# Survodutide in adults with obesity and metabolic dysfunction-associated steatotic liver disease: SYNCHRONIZE-MASLD, a randomized, double-blind, placebo-controlled phase 3 trial

**DOI:** 10.1038/s41591-026-04479-3

**Published:** 2026-06-07

**Authors:** Lee M. Kaplan, Elena Startseva, Carel W. le Roux, Sean Wharton, Biykem Bozkurt, Daniel F. Mazo, Jessica von Schlippenbach, Sandra González Maldonado, Samina Ajaz Hussain, Guy W. Neff, Yaneicy Gonzalez Rojas, Charles Smith, Ramy Younes, Arun J. Sanyal

**Affiliations:** 1 The Obesity and Metabolism Institute, Boston, MA USA; 2 Geisel School of Medicine at Dartmouth, Hanover, NH USA; 3 Boehringer Ingelheim International GmbH, Ingelheim am Rhein, Germany; 4 University College Dublin School of Medicine, Dublin, Ireland; 5 Ulster University, Coleraine, UK; 6 McMaster University, Hamilton, Ontario Canada; 7 University of Toronto, Toronto, Ontario Canada; 8 Winters Center for Heart Failure Research, Cardiovascular Research Institute, Baylor College of Medicine, Houston, TX USA; 9 Boehringer Ingelheim International GmbH, Biberach, Germany; 10 Boehringer Ingelheim Pharma GmbH & Co. KG, Biberach, Germany; 11 Covenant Metabolic Specialists, LLC, Sarasota and Fort Myers, FL USA; 12 Optimus U Corp., Miami, FL USA; 13 Stravitz Sanyal Institute for Liver Disease and Metabolic Health, Division of Gastroenterology, Hepatology and Nutrition, Virginia Commonwealth University School of Medicine, Richmond, VA USA; 14 Hospital Universitario La Paz, Madrid, Spain; 15 Liver Unit, Vall d’Hebron University Hospital, VHIR, Universitat Autònoma de Barcelona, CIBERehd, Barcelona, Spain; 16 Velocity Clinical Research, Gardena, CA USA; 17 Clinical Trials of Texas, LLC, San Antonio, dba Flourish Research, San Antonio, TX USA; 18 Washington University School of Medicine, St. Louis, MO USA; 19 Fleming Island Center for Clinical Research, Fleming Island, FL USA; 20 Kansas Medical Clinic, Topeka, KS USA; 21 Excel Medical Clinical Trials, LLC, Boca Raton, dba Flourish Research, Boca Raton, FL USA; 22 Amel Med, LLC, Georgetown, TX USA; 23 Ark Clinical Research – PARENT, Long Beach, CA USA; 24 Louisiana Research Center, LLC, Shreveport, LA USA; 25 Indiana University, Indianapolis, IN USA; 26 Biopharma Informatic, LLC, McAllen, TX USA; 27 Health Awareness, Inc., Jupiter, FL USA; 28 DSI Research Northridge, LLC, Dayton, OH USA; 29 University of Iowa Health Care, Iowa City, IA USA; 30 Velocity Clinical Research, North Hollywood, CA USA; 31 Velocity Clinical Research, Hallandale Beach, FL USA; 32 Panax Clinical Research, LLC, Miami Lakes, FL USA; 33 Ark Clinical Research, Fountain Valley, CA USA; 34 GI Select Health Research, LLC, Richmond, VA USA; 35 Catalina Research Institute, Montclair, CA USA; 36 Velocity Clinical Research, Austin, TX USA; 37 Velocity Clinical Research, Santa Ana, CA USA; 38 Velocity Clinical Research, Waco, TX USA; 39 Accurate Clinical Research, Inc., Richmond, TX USA; 40 Springfield Clinic, Springfield, IL USA; 41 Segal Drug Trials, Delray Beach, FL USA; 42 Nature Coast Clinical Research, Inverness, FL USA

**Keywords:** Outcomes research, Diagnostic markers

## Abstract

Survodutide is a glucagon receptor/glucagon-like peptide-1 receptor dual agonist under investigation for treating obesity and related diseases. The SYNCHRONIZE-MASLD phase 3, randomized, double-blind, placebo-controlled trial included 216 adults (131 female and 85 male) with obesity (defined as a body mass index ≥30 kg m^−^^2^ or ≥27 kg m^−^^2^ with at least one obesity complication) and at-risk metabolic dysfunction-associated steatotic liver disease (MASLD), defined by MASLD with evidence of liver inflammation and/or fibrosis by noninvasive tests (NITs) or biopsy-confirmed metabolic dysfunction-associated steatohepatitis (MASH). Participants were randomized (2:1) and treated with once-weekly subcutaneous injections of survodutide 6.0 mg (*n* = 146) or placebo (*n* = 70). The co-primary endpoints, ≥30% reduction in magnetic resonance imaging-proton density fat fraction (MRI-PDFF)-assessed liver fat content (LFC) and percentage change in body weight (both baseline to week 48), were met. In total, 84.2% of survodutide-treated patients versus 24.3% of placebo-treated patients had ≥30% reduction in LFC using the efficacy estimand (*P* < 0.0001; treatment regimen estimand: 68.5% versus 28.6%, respectively; *P* < 0.0001). Mean percentage change in body weight was −12.2% with survodutide and −1.0% with placebo using the efficacy estimand (*P* < 0.0001; treatment regimen estimand: −8.7% versus −1.4%, respectively; *P* < 0.0001). The most frequently reported adverse events with survodutide were gastrointestinal, commonly occurring during dose escalation, and were generally of mild-to-moderate severity. In adults with obesity and at-risk MASLD, survodutide treatment was statistically and clinically superior to placebo for reductions in MRI-PDFF-assessed LFC and body weight. Limitations included short trial duration (48 weeks) and limited global reach (participants recruited in the United States and Spain).

## Abstract (web summary)

As presented at the American Diabetes Association Meeting, in this phase 3 trial, weekly subcutaneous injections of survodutide, a glucagon receptor/GLP-1R dual agonist, lowered liver fat content and body weight compared to placebo in adults with obesity and at-risk metabolic dysfunction-associated steatotic liver disease (MASLD).

## Main

Obesity is a multifaceted, chronic disease driven by metabolic dysfunction that can contribute to the development and progression of MASLD^1–5^. MASLD is defined by hepatic steatosis identified by imaging or biopsy together with at least one cardiometabolic risk factor^2^. The progressive form of MASLD, MASH, is defined by steatosis, inflammation and hepatic cell injury^2^. Lipotoxicity, chronic inflammation and metabolic stress associated with MASH can, in turn, drive the development of liver fibrosis and cirrhosis, increasing the risk of adverse liver outcomes^2,5,6^. A recent meta-analysis estimated that the global prevalence of MASLD in adults with obesity is approximately 75%, and approximately one-third have MASH^7^.

Timely identification and treatment of MASLD and MASH is important to halt the disease course before the development of advanced fibrosis and improve long-term outcomes, quality of life and economic burden associated early in the course of MASLD^8^. Hepatic outcomes are linked to fibrosis and steatohepatitis, the latter defined by hepatic inflammation and hepatocyte injury, which are not easily assessed in routine clinical settings. Various NITs reflective of different aspects of metabolic liver disease have been developed and validated. Several of them are incorporated in MASLD screening algorithms developed by professional organizations representing primary care and specialty physicians to identify people with MASLD at heightened risk of disease progression to MASH^2,9,10^. Nonetheless, limited disease awareness and a lack of routine, noninvasive screening in the primary care environment limits screening and detection, resulting in missed opportunities for timely lifestyle and pharmacological management^11^. Formal diagnosis and staging of MASH requires assessment of a liver biopsy; however, blood-based and imaging-based NITs, including MRI-PDFF to quantify LFC, are becoming increasingly more common in routine clinical practice to diagnose people with MASLD or MASH and to identify people who could benefit from treatment^12–14^.

For people who are diagnosed with obesity and MASLD, current pharmacologic treatment options include interventions for obesity treatment, including recently approved medications with glucagon-like peptide-1 receptor (GLP-1R) agonist activity (semaglutide and tirzepatide)^15,16^ and medications that are conditionally approved by the US Food and Drug Administration for the treatment of MASH with moderate-to-advanced liver fibrosis (stage F2/F3; semaglutide and resmetirom)^5,15,17–19^. Nonetheless, the wide patient-to-patient variability in response to current medications to treat MASH suggests a substantial unmet need for additional treatment options for patients with obesity and MASH, with or without fibrosis^20^.

Survodutide is a glucagon receptor/GLP-1R dual agonist, designed to replicate the actions of oxyntomodulin, an endogenous glucagon receptor/GLP-1R dual agonist that can influence body weight and liver health. Survodutide, distinguished by its enhanced receptor agonist activity and long half-life^21–23^, is currently under investigation for the treatment of obesity and related metabolic diseases, including MASH. In phase 2 trials, survodutide treatment was shown to improve histological MASH and liver fibrosis and reduce LFC in participants with biopsy-confirmed MASH^24^ and to induce significant weight reduction in participants with obesity with or without type 2 diabetes (T2D)^25,26^. The SYNCHRONIZE phase 3 clinical trial program was designed to further evaluate the effects of survodutide treatment in people with obesity. In this report, we describe the design and primary results from the SYNCHRONIZE-MASLD phase 3 trial of survodutide treatment in adults with obesity and ‘at-risk MASLD’, defined as MASLD with evidence of inflammation and/or fibrosis using NITs or a recent history of biopsy-confirmed MASH.

## Results

### SYNCHRONIZE-MASLD trial design

We conducted a 48-week placebo-controlled trial (that included a 24-week dose-escalation period for uptitration of survodutide to 6.0 mg and a 24-week dose-maintenance period) in participants aged ≥18 years, with body mass index (BMI) ≥30 kg m^−^^2^ or BMI ≥27 kg m^−^^2^ and at least one metabolic obesity complication (hypertension, dyslipidemia, obstructive sleep apnea, cardiovascular disease and/or T2D), a history of at least one patient-reported unsuccessful dietary effort to lose body weight and at-risk MASLD as defined by hepatic steatosis (MRI-PDFF ≥8% at screening) with the exclusion of secondary causes of hepatic fat accumulation and one or more elevated NITs (see Methods for full list) or a history of biopsy-confirmed MASH within the past 3 years.

The co-primary endpoints were the percentage change in body weight and the proportion of participants with a ≥30% reduction in LFC as assessed by MRI-PDFF, each from baseline to week 48. The secondary and further endpoints (each assessed from baseline to week 48) included absolute and percentage change in LFC (%) and change in MRI-assessed liver volume (ml); hepatic and systemic inflammatory activity as assessed by circulating alanine aminotransferase (ALT; U l^−1^) and high-sensitivity C-reactive protein (hsCRP), respectively; metabolic function as predicted by waist circumference (cm) and homeostatic model assessment of insulin resistance (HOMA-IR); liver stiffness, a marker of liver fibrosis, as assessed by vibration-controlled transient elastography (VCTE; kPa) and magnetic resonance elastography (MRE; kPa); and changes in liver fibroinflammatory activity as determined by corrected T1 (cT1) levels and the proportion of participants with a ≥80-ms reduction in cT1.

### Participants

**Fig. 1:**
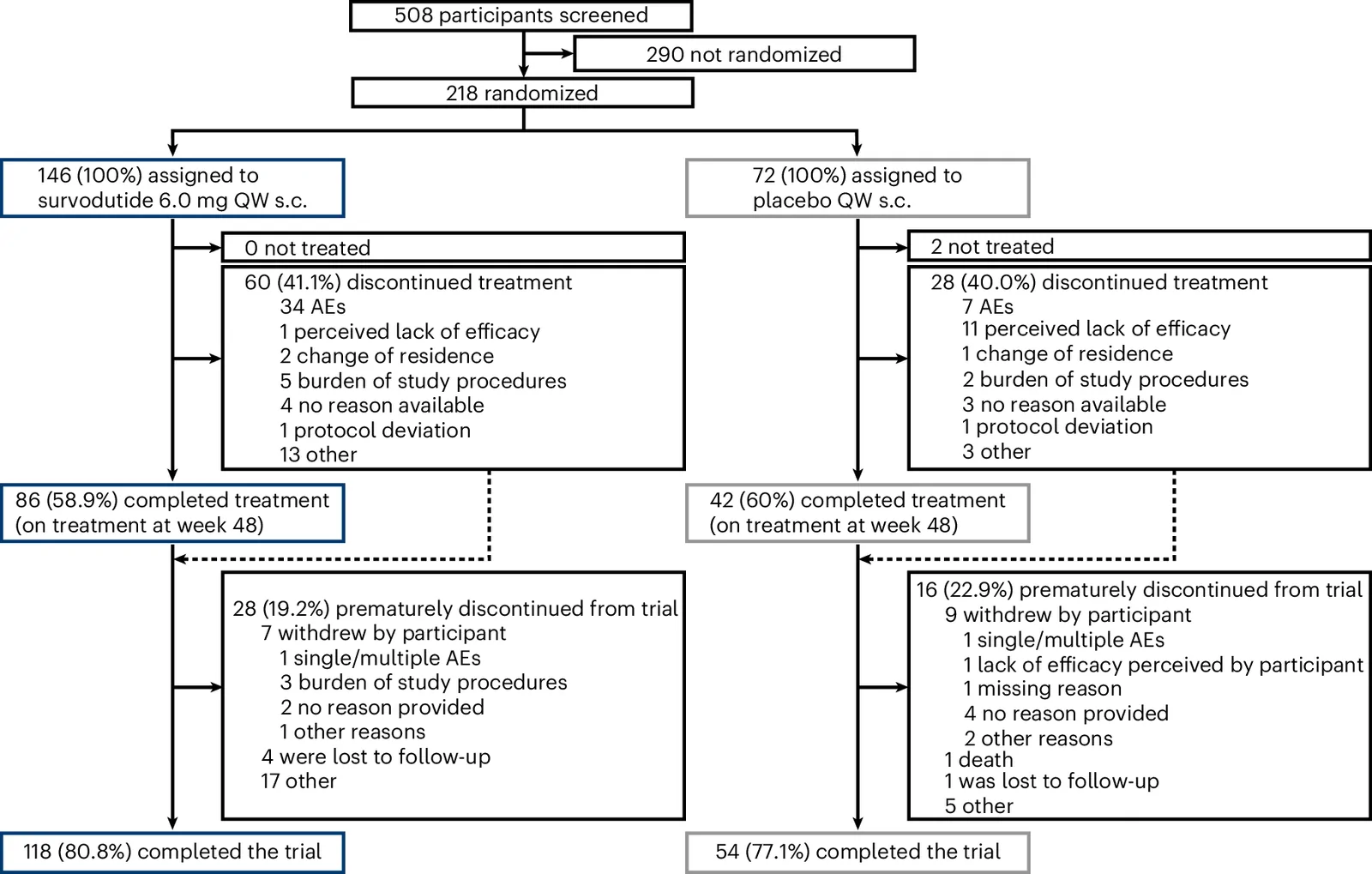
Participant disposition. AE, adverse event; QW, once-weekly; s.c., subcutaneous.

**Extended Data Fig. 1:**
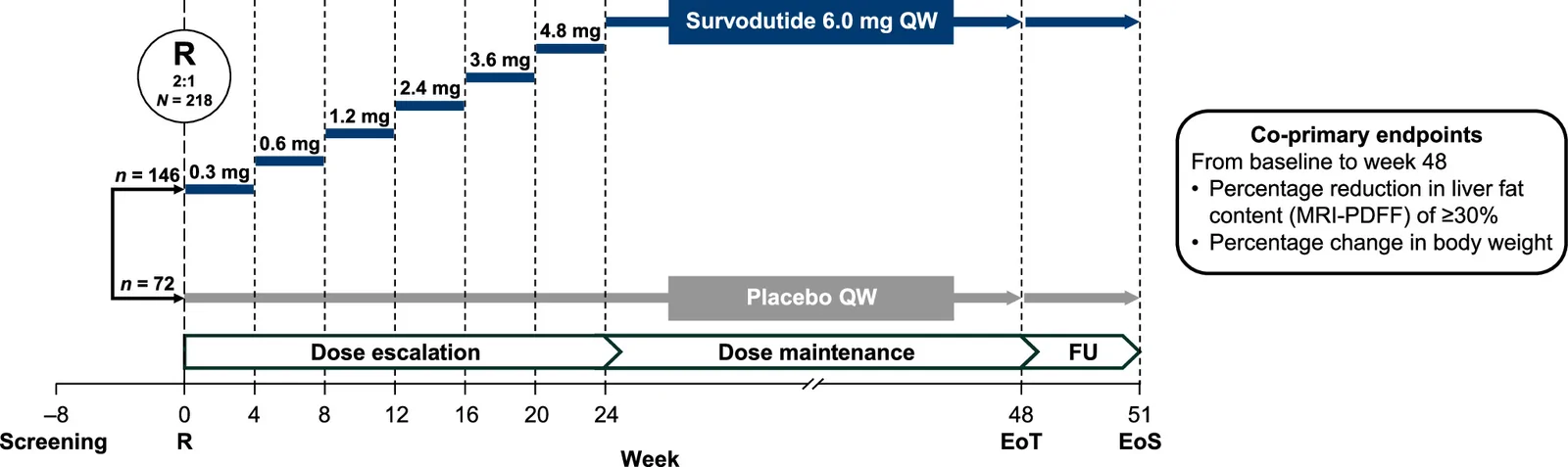
Trial design. EOS, end of study; EOT, end of treatment; FU, follow-up; MRI-PDFF, magnetic resonance imaging-proton density fat fraction; QW, once weekly; R, randomization.

The trial was conducted between 2 April 2024 and 2 December 2025. Of the 508 participants assessed for eligibility, 218 with obesity and at-risk MASLD underwent randomization (2:1) to weekly survodutide 6.0 mg or placebo, and 216 received at least one dose of treatment (Fig. 1 and Extended Data Fig. 1). Overall, 172 of 216 participants (79.6%) completed the trial. Treatment discontinuation occurred at a similar frequency in the survodutide (41.1% (60/146)) and placebo (40.0% (28/70)) arms. Among participants randomized to survodutide who did not prematurely discontinue study treatment (*n* = 86), 80.2% maintained the target dose of 6.0 mg until the end of the treatment period; six participants (7.0%) ended the trial on a dose of 4.8 mg, five participants (5.8%) on a dose of 3.6 mg and six participants (7.0%) on a dose of 2.4 mg.

Participant characteristics at baseline were similar across treatment groups (Table 1 and Extended Data Table 1). Mean age was 55.8 years, 60.6% were female, mean BMI was 39.6 kg m^−^^2^, mean body weight was 108.3 kg and mean waist circumference was 120.9 cm. Nearly all participants (94.4%) had at least one extra-hepatic metabolic complication, including 38.4% with T2D. At baseline, mean MRI-PDFF-assessed LFC was 16.9%.

**Table 1:** Participant demographic and clinical characteristics at baseline

	Survodutide 6.0 mg (*n* = 146)	Placebo (*n* = 70)	Total (*n* = 216)
Demographic characteristics			
Age, mean ± s.d., years	56.0 ± 12.4	55.4 ± 12.9	55.8 ± 12.5
Female sex, no. (%)	92 (63.0)	39 (55.7)	131 (60.6)
Race, no. (%)			
White	132 (90.4)	64 (91.4)	196 (90.7)
Black or African American	13 (8.9)	3 (4.3)	16 (7.4)
Asian	1 (0.7)	1 (1.4)	2 (0.9)
American Indian or Alaska Native	0 (0.0)	1 (1.4)	1 (0.5)
Native Hawaiian or Other Pacific Islander	0 (0.0)	0 (0.0)	0 (0.0)
Multiple	0 (0.0)	1 (1.4)	1 (0.5)
Ethnicity, no. (%)			
Hispanic or Latino	59 (40.4)	29 (41.4)	88 (40.7)
Not Hispanic or Latino	87 (59.6)	41 (58.6)	128 (59.3)
Clinical characteristics			
Body weight, mean ± s.d., kg	106.6 ± 23.3	111.9 ± 20.5	108.3 ± 22.5
BMI, mean ± s.d., kg m^−^^2^	39.0 ± 7.9	40.7 ± 7.7	39.6 ± 7.9
BMI category, kg m^−^^2^, no. (%)			
27 to <30	10 (6.8)	3 (4.3)	13 (6.0)
30 to <35	36 (24.7)	15 (21.4)	51 (23.6)
35 to <40	43 (29.5)	18 (25.7)	61 (28.2)
≥40	57 (39.0)	34 (48.6)	91 (42.1)
Obesity complications, no. (%)			
Dyslipidemia	107 (73.3)	46 (65.7)	153 (70.8)
Hypertension	102 (69.9)	50 (71.4)	152 (70.4)
T2D	56 (38.4)	27 (38.6)	83 (38.4)
Obstructive sleep apnea	50 (34.2)	27 (38.6)	77 (35.6)
Cardiovascular disease	7 (4.8)	2 (2.9)	9 (4.2)
≥1 obesity complications, no. (%)	137 (93.8)	67 (95.7)	204 (94.4)
Biopsy-confirmed MASH (fibrosis stage F0–F3), no. (%)	12 (8.2)	9 (12.9)	21 (9.7)
NIT-determined MASH^a^, no. (%)	134 (91.8)	61 (87.1)	195 (90.3)
NITs			
Liver fat, fibrosis and inflammation			
LFC by MRI-PDFF^b^, mean ± s.d., %	16.9 ± 7.0 *n* = 146	16.9 ± 6.6 *n* = 70	16.9 ± 6.9 *n* = 216
Liver stiffness by MRE, mean ± s.d., kPa	2.8 ± 0.6 *n* = 142	2.8 ± 0.7 *n* = 70	2.8 ± 0.7 *n* = 212
MAST score, mean ± s.d., units on a scale	0.06 ± 0.08 *n* = 144	0.07 ± 0.10 *n* = 70	0.06 ± 0.08 *n* = 214
Liver volume by MRI, mean ± s.d., ml	2,234.5 ± 537.5 *n* = 144	2,274.8 ± 518.8 *n* = 70	2,247.7 ± 530.6 *n* = 214
CAP by FibroScan^c^, mean ± s.d., dB m^−1^	327.7 ± 38.6 *n* = 128	343.5 ± 32.5 *n* = 62	332.9 ± 37.4 *n* = 190
Liver stiffness by FibroScan^c^, mean ± s.d., kPa	10.2 ± 3.4 *n* = 127	10.7 ± 3.1 *n* = 59	10.3 ± 3.3 *n* = 186
FAST score^c^, mean ± s.d., units on a scale	0.4 ± 0.2 *n* = 124	0.4 ± 0.2 *n* = 58	0.4 ± 0.2 *n* = 182
ELF score, mean ± s.d., units on a scale	9.4 ± 0.7 *n* = 146	9.4 ± 0.8 *n* = 70	9.4 ± 0.8 *n* = 216
FIB-4 index, mean ±s.d., units on a scale	1.3 ± 0.7 *n* = 146	1.2 ± 0.6 *n* = 70	1.3 ± 0.7 *n* = 216
cT1 by MRI, mean ± s.d., ms	923.3 ± 109.4 *n* = 141	926.4 ± 136.2 *n* = 68	924.3 ± 118.5 *n* = 209
ALT, geometric mean (geometric s.d.), U l^−1^	35.9 (1.8) *n* = 146	34.4 (1.8) *n* = 70	35.4 (1.8) *n* = 216
AST, geometric mean (geometric s.d.), U l^−1^	29.7 (1.7) *n* = 146	28.5 (1.7) *n* = 70	29.3 (1.7) *n* = 216
Cardiometabolic factors			
Waist circumference, mean ± s.d., cm	119.8 ± 13.7 *n* = 146	123.3 ± 14.3 *n* = 70	120.9 ± 14.0 *n* = 216
SBP, mean ± s.d., mmHg	127.7 ± 12.4 *n* = 146	128.9 ± 14.3 *n* = 70	128.1 ± 13.0 *n* = 216
DBP, mean ± s.d., mmHg	79.9 ± 7.2 *n* = 146	80.1 ± 8.4 *n* = 70	80.0 ± 7.6 *n* = 216
HbA1c, mean ± s.d., %	6.2 ± 1.1 *n* = 146	6.2 ± 1.1 *n* = 69	6.2 ± 1.1 *n* = 215
Triglycerides, geometric mean (geometric s.d.), mg dl^−1^	160.3 (1.6) *n* = 146	155.9 (1.5) *n* = 68	158.9 (1.6) *n* = 214
Total cholesterol, geometric mean (geometric s.d.), mg dl^−1^	180.9 (1.3) *n* = 146	182.3 (1.3) *n* = 70	181.3 (1.3) *n* = 216
LDL cholesterol, geometric mean (geometric s.d.), mg dl^−1^	96.8 (1.4) *n* = 140	98.0 (1.6) *n* = 66	97.2 (1.5) *n* = 206
HDL cholesterol, geometric mean (geometric s.d.), mg dl^−1^	44.6 (1.3) *n* = 146	45.0 (1.2) *n* = 68	44.7 (1.3) *n* = 214
VLDL cholesterol, geometric mean (geometric s.d.), mg dl^−1^	26.7 (1.6) *n* = 146	26.0 (1.5) *n* = 67	26.5 (1.6) *n* = 213
hsCRP, mean ± s.d., mg l^−1^	5.9 ± 6.4 *n* = 146	6.3 ± 6.4 *n* = 69	6.0 ± 6.4 *n* = 215
Uric acid, mean ± s.d., μmol l^−1^	345.9 ± 79.6 *n* = 146	335.2 ± 84.5 *n* = 70	342.4 ± 81.2 *n* = 216
HOMA-IR^d^, geometric mean (geometric s.d.), units on a scale	5.4 (2.2) *n* = 132	5.5 (2.2) *n* = 63	5.4 (2.2) *n* = 195

Additional baseline characteristics are provided in Extended Data Table 1.

^a^ Based on NITs at screening: MRE ≥2.61 kPa and <4.68 kPa or FibroScan VCTE ≥8 kPa and <20 kPa or ELF score >7.7 and <11.3 or FIB-4 score ≥2.67 and <3.48 or MAST score ≥0.242 or FAST score ≥0.5 or cT1 ≥875 ms.

^b^ Inclusion criteria for NIT-determined or biopsy-confirmed MASH included MRI-PDFF ≥8%.

^c^ Excluding participants who had at least one measurement with SmartExam.

^d^ Participants without background insulin.

ALT, alanine aminotransferase; AST, aspartate aminotransferase; BMI, body mass index; CAP, controlled attenuation parameter; cT1, iron-corrected T1; DBP, diastolic blood pressure; ELF, enhanced liver fibrosis; FAST, FibroScan-aspartate aminotransferase; FIB-4, fibrosis-4; HbA1c, glycated hemoglobin; HDL, high-density lipoprotein; HOMA-IR, homeostasis model assessment-insulin resistance; hsCRP, high sensitivity C-reactive protein; LDL, low-density lipoprotein; LFC, liver fat content; MASH, metabolic dysfunction-associated steatohepatitis; MAST, magnetic resonance imaging-aspartate aminotransferase; MRE, magnetic resonance elastography; MRI, magnetic resonance imaging; NIT, noninvasive test; PDFF, proton density fat fraction; SBP, systolic blood pressure; s.d., standard deviation; VCTE, vibration-controlled transient elastography; VLDL, very low-density lipoprotein.

Most trial participants (90.3%) had at-risk MASLD with NIT-based evidence of either no or mild-to-moderate fibrosis at baseline, and the remaining 9.7% had a history of biopsy-confirmed MASH within the past 3 years. The mean baseline liver stiffness as assessed by MRE was 2.8 kPa (stage F0 or F1), and the mean fibrosis-4 (FIB-4) score was 1.3. The mean baseline liver stiffness as assessed by VCTE was 10.3 kPa, and the enhanced liver fibrosis (ELF) score was 9.4, suggesting that the spectrum of liver fibrosis likely ranged from stage F1 to F2. The mean cT1 at baseline was 924.3 ms, consistent with fibroinflammation.

### Change in LFC assessed by MRI-PDFF

**Fig. 2:**
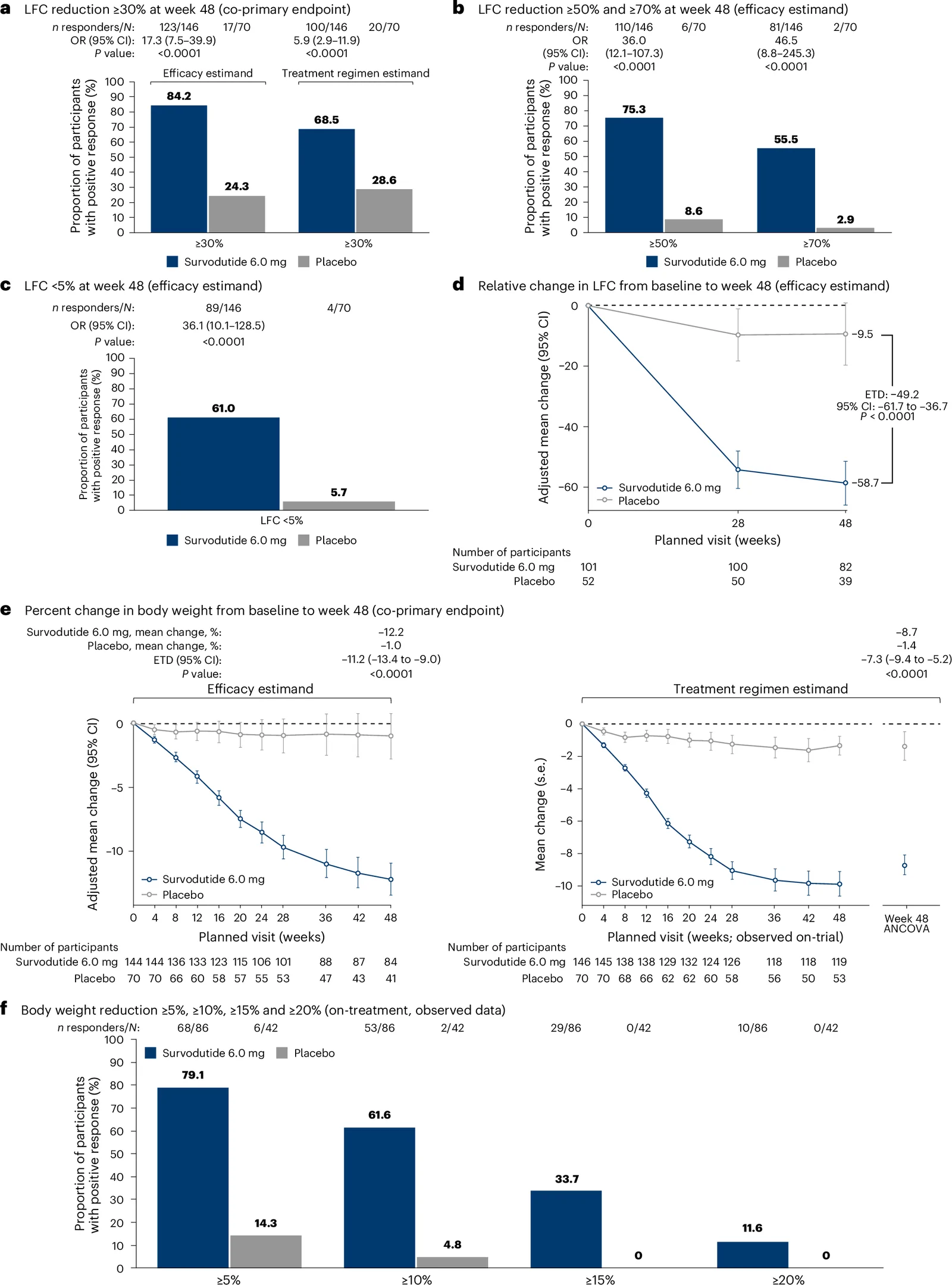
Change in LFC by MRI-PDFF and body weight from baseline to week 48. **a**, Estimated proportion of participants who had an LFC (MRI-PDFF) reduction of ≥30% from baseline to week 48 (co-primary endpoint), derived from logistic regression (Rubin-based Wald *t*-test, two-sided *P* value). **b**, Estimated proportion of participants who had an LFC (MRI-PDFF) reduction of ≥50% and ≥70% from baseline to week 48, derived from logistic regression (Rubin-based Wald *t*-test, two-sided *P* value). **c**, LFC (MRI-PDFF) <5% at week 48, derived from logistic regression (Rubin-based Wald *t*-test, two-sided *P* value). **d**, Relative change in LFC (MRI-PDFF) from baseline to week 48, derived from MMRM (two-sided test). **e**, Percentage change in body weight over time (co-primary endpoint) (Rubin-based Wald *t*-test, two-sided test). **f**, Body weight reduction ≥5%, ≥10%, ≥15% and ≥20% from baseline to week 48, derived from logistic regression (Rubin-based Wald *t*-test, two-sided *P* value). No adjustments for multiple comparisons were done. In **a**, estimates for the treatment regimen estimand used multiple imputation based on the ‘follow the reference’ strategy, where trial participants in the survodutide arm were assumed to have an effect similar to that of the placebo arm while preserving information of any on-treatment and off-treatment data. In **a**–**c**, for the efficacy estimand, missing values of LFC (MRI-PDFF) were multiply imputed using a MAR assumption and dichotomized. In **d**, absolute change over time was derived from an MMRM analysis. In **e**, for the efficacy estimand, percentage body weight reduction was derived from an MMRM analysis. For the treatment regimen estimand, the left-hand side of the chart shows the observed mean percentage change in body weight over time; estimates on the right-hand side were derived from an ANCOVA model. ANCOVA, analysis of covariance; CI, confidence interval; ETD, estimated treatment difference; LFC, liver fat content; MAR, missing at random; MMRM, mixed models for repeated measures; MRI-PDFF, magnetic resonance imaging derived proton density fat fraction; OR, odds ratio. Source data

Based on the efficacy estimand, 84.2% of participants treated with survodutide 6.0 mg weekly had a ≥30% reduction in LFC assessed by MRI-PDFF (co-primary endpoint) at week 48 compared to 24.3% receiving placebo (odds ratio = 17.3, 95% confidence interval: 7.5−39.9, *P* < 0.0001) (Fig. 2a). Using the treatment regimen estimand, 68.5% of participants in the survodutide 6.0 mg treatment arm had a ≥30% reduction in LFC compared to 28.6% of participants in the placebo arm (odds ratio = 5.9, 95% confidence interval: 2.9−11.9, *P* < 0.0001) (Fig. 2a).

Using the efficacy estimand, 75.3% of participants had an MRI-PDFF-assessed LFC reduction of ≥50% with survodutide 6.0 mg versus 8.6% with placebo, and 55.5% of participants in the survodutide arm had an LFC reduction of ≥70% versus 2.9% in the placebo arm (Fig. 2b). In addition, 61.0% of participants treated with survodutide had an LFC <5% at week 48 versus 5.7% treated with placebo (Fig. 2c). The mean relative change in LFC was −58.7% with survodutide 6.0 mg versus −9.5% with placebo (estimated treatment difference (ETD) −49.2%, 95% confidence interval: −61.7 to −36.7, *P* < 0.0001) (Fig. 2d). Descriptive subgroup analysis of MRI-PDFF-assessed LFC reduction of ≥30% by T2D, BMI, sex and age is shown in Extended Data Table 2.

### Change in body weight

At week 48, the mean change in body weight (co-primary endpoint) was −12.2% with survodutide treatment versus −1.0% with placebo using the efficacy estimand, with an ETD of −11.2% (95% confidence interval: −13.4 to −9.0, *P* < 0.0001) (Fig. 2e). Using the treatment regimen estimand, the respective mean changes in body weight were −8.7% and −1.4% for the survodutide and placebo groups, respectively, with an ETD of −7.3% (95% confidence interval: −9.4 to −5.2, *P* < 0.0001) (Fig. 2e). More than three-fourths of participants (79.1%) had a body weight reduction of ≥5% with survodutide treatment versus 14.3% in the placebo-treated group (Fig. 2f). Descriptive subgroup analysis of percentage change in body weight by T2D, BMI, sex, and age is presented in Extended Data Table 3.

### Additional measures of liver fat, fibrosis and inflammation

**Extended Data Fig. 2:**
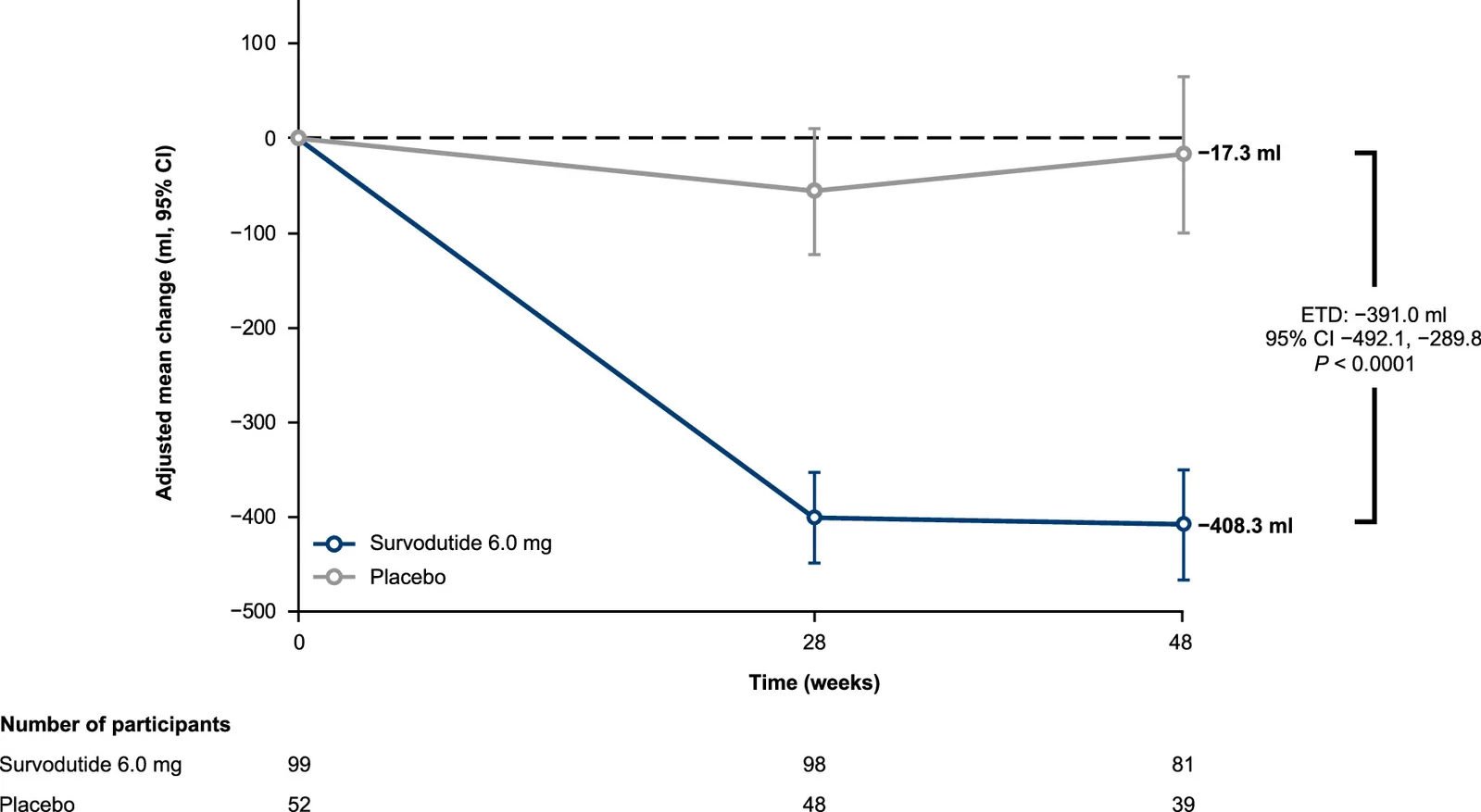
Absolute change in liver volume (MRI) from baseline to week 48 (efficacy estimand)^a^. ^a^Absolute change in liver volume assessed by MRI from baseline to week 52, derived from an MMRM analysis (two-sided test). CI, confidence interval; ETD, estimated treatment difference; MMRM, mixed models for repeated measures; MRI, magnetic resonance imaging. Source data

**Extended Data Fig. 3:**
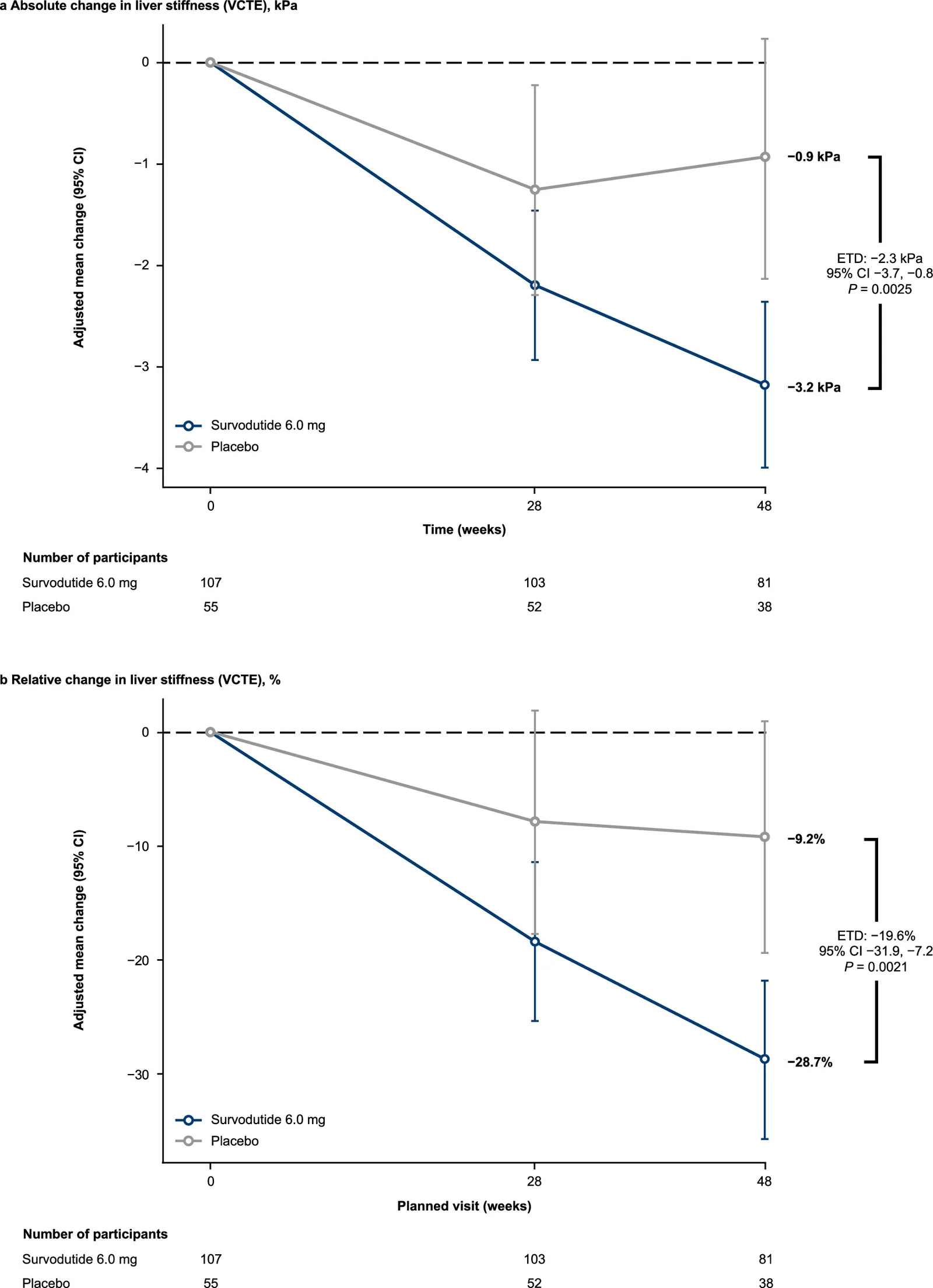
Change in liver stiffness (VCTE) from baseline to week 48 (MMRM analysis; on-treatment)^a^. **a**. Absolute change in liver stiffness (VCTE). **b**. Relative change in liver stiffness (VCTE). ^a^Absolute and relative change in liver stiffness from baseline to week 48, derived from an MMRM analysis (two-sided test). Participants who had at least one measurement with SmartExam were excluded. CI, confidence interval; ETD, estimated treatment difference; MMRM, mixed models for repeated measures; VCTE, vibration-controlled transient elastography. Source data

Additional measures determined at baseline and 48 weeks included liver volume assessed by MRI, which decreased by 408.3 ml with survodutide treatment compared to 17.3 ml with placebo (ETD −391.0 ml, 95% confidence interval: −492.1 to −289.8, *P* < 0.0001) (Extended Data Fig. 2). Liver stiffness assessed by VCTE was reduced by 28.7% after survodutide treatment versus by 9.2% in the placebo-treated group (ETD −19.6%, 95% confidence interval: −31.9 to −7.2, *P* = 0.0021) (Extended Data Fig. 3). Changes in liver stiffness assessed by MRE did not show a difference between the survodutide 6.0 mg and placebo groups (ETD −0.05, 95% confidence interval: −0.25 to 0.15, *P* = 0.6235). The ELF score decreased by 0.34 in the survodutide arm versus a 0.06 decrease with placebo (ETD −0.28, 95% confidence interval: −0.50 to −0.05, *P* = 0.0161).

**Fig. 3:**
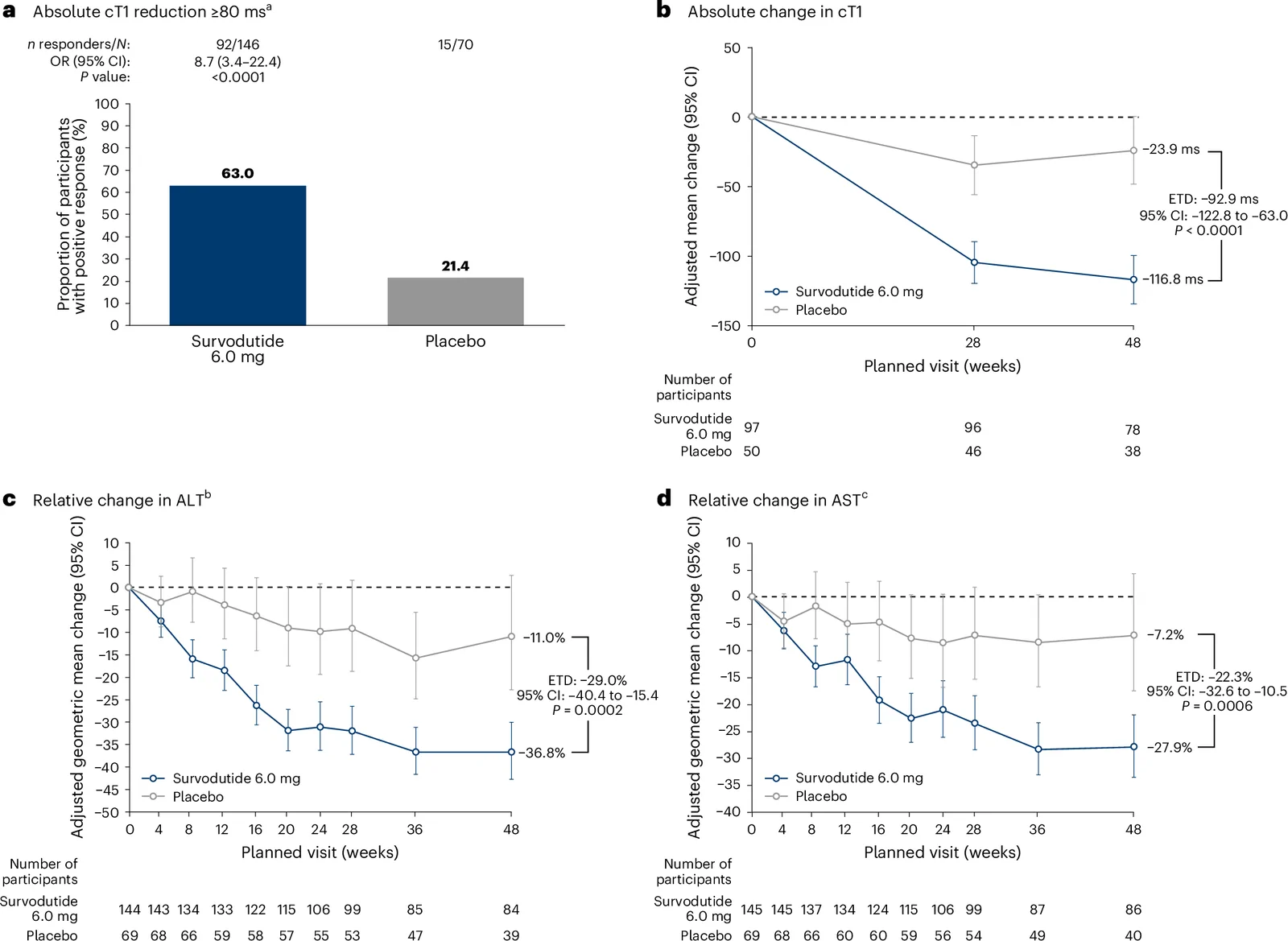
Change in biomarkers of liver inflammation from baseline to week 48 (efficacy estimand). **a**, Estimated proportion of participants who had an absolute cT1 reduction ≥80 ms from baseline to week 48, derived from logistic regression (Rubin-based Wald *t*-test, two-sided *P* value). **b**, Absolute change in cT1 over time, derived from an MMRM analysis (two-sided test). **c**, Relative change in ALT over time, derived from an MMRM analysis (two-sided test). **d**, Relative change in AST over time, derived from an MMRM analysis (on-treatment) (two-sided test). ^a^ 80 ms is considered as a meaningful clinical response that corresponds to a histological 2-point decrease in the NAS score with no worsening in fibrosis^34^. ^b^ Measurements were analyzed on a log scale; therefore, only relative changes (as percentages) are reported. Descriptive statistics for absolute changes in ALT are mean ± s.d., −16.6 ± 25.9 U l^−1^ with survodutide 6.0 mg (*n* = 86) and −6.4 ± 23.5 U l^−1^ with placebo (*n* = 40) at week 48 (on-treatment). ^c^ Absolute change in AST −9.7 U l^−1^ with survodutide 6.0 mg and −4.5 U l^−1^ with placebo at week 48; ETD (95% CI) −5.1 (−10.5 to 0.2) (MMRM estimates, on-treatment). ALT, alanine aminotransferase; AST, aspartate aminotransferase; CI, confidence interval; cT1, iron-corrected T1; ETD, estimated treatment difference; MMRM, mixed models for repeated measures; NAS, nonalcoholic fatty liver disease activity score; OR, odds ratio. Source data

There were greater improvements in markers of liver inflammation (cT1, ALT and aspartate aminotransferase (AST)) with survodutide treatment than with placebo (Fig. 3). At week 48, 63.0% of participants treated with survodutide had an absolute reduction in cT1 of ≥80 ms from baseline compared to 21.4% with placebo (Fig. 3a). The mean absolute change in cT1 was −116.8 ms with survodutide 6.0 mg versus −23.9 ms with placebo (Fig. 3b). The mean relative change in ALT from baseline to week 48 was −36.8% with survodutide treatment versus −11.0% with placebo, and the analogous changes in AST were −27.9% and −7.2%, respectively (Fig. 3c,d). Among participants with elevated ALT levels at baseline, 82.1% in the survodutide arm exhibited normalization of their ALT (<43 for females, <48 for males) at 48 weeks compared to 27.3% in the placebo group.

### Cardiometabolic risk factors and biomarkers

**Extended Data Fig. 4:**
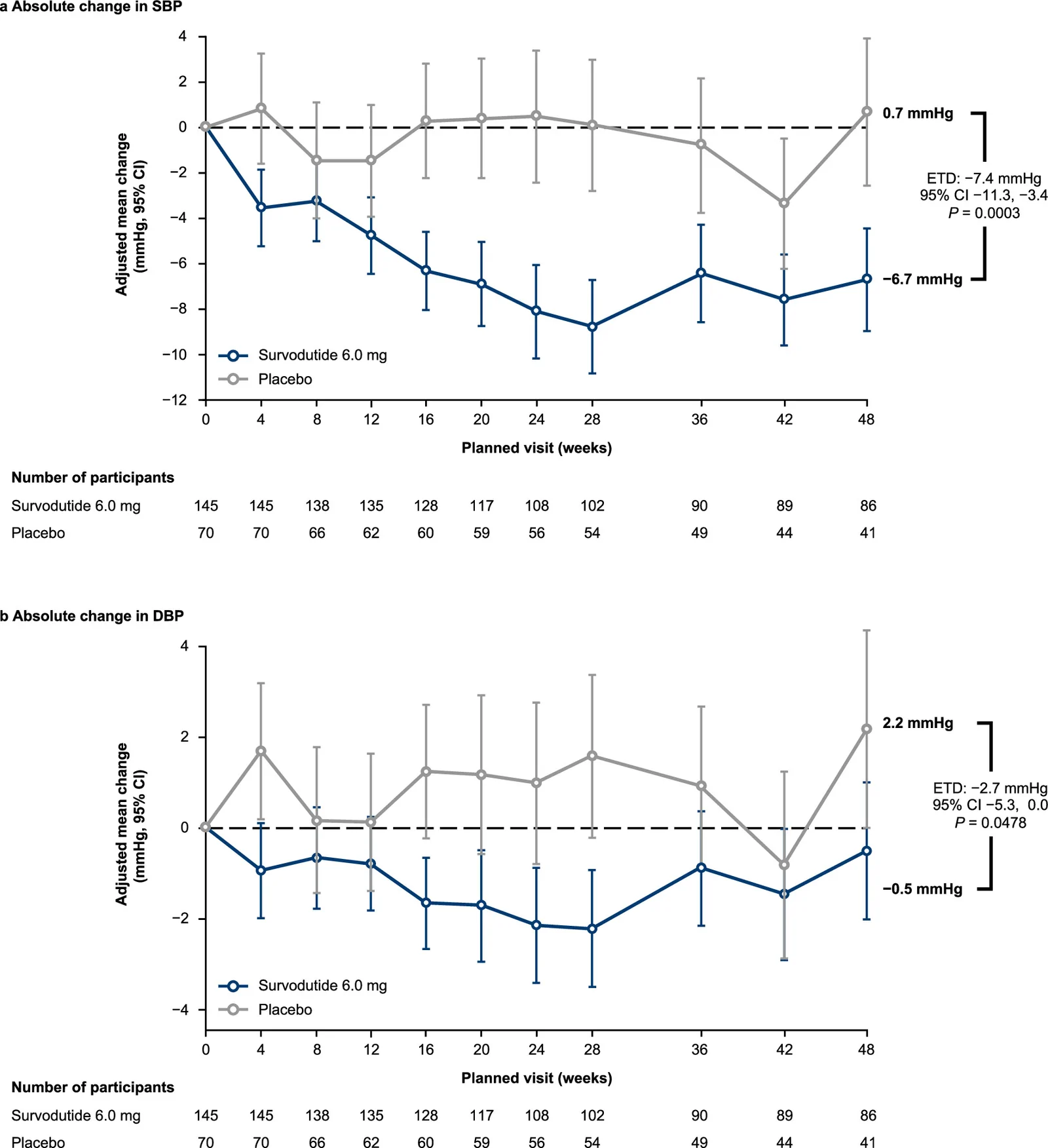
Change in SBP and DBP from baseline to week 48 (MMRM analysis; on-treatment)^a^. **a**. Absolute change in SBP. **b**. Absolute change in DBP. ^a^Absolute change in SBP and DBP from baseline to week 48, derived from an MMRM analysis (two-sided test). CI, confidence interval; DBP, diastolic blood pressure; ETD, estimated treatment difference; MMRM, mixed models for repeated measures; SBP, systolic blood pressure. Source data

At week 48, mean waist circumference was reduced by 11.1 cm after survodutide compared to 1.9 cm after placebo (ETD −9.3, 95% confidence interval: −11.8 to −6.8, *P* < 0.0001) using the efficacy estimand. Significantly greater reductions in systolic blood pressure (SBP) and diastolic blood pressure (DBP) were seen with survodutide than with placebo, with a treatment difference of −7.4 mmHg (95% confidence interval: −11.3 to −3.4, *P* = 0.0003) for SBP and −2.7 mmHg (95% confidence interval: −5.3 to −0.03, *P* = 0.0478) for DBP (Table 2 and Extended Data Fig. 4). Improvements were also observed in glycated hemoglobin (HbA1c) and lipid levels (triglycerides, total cholesterol and very-low-density lipoprotein (VLDL) cholesterol) with survodutide 6.0 mg versus placebo (Table 2). Other cardiometabolic biomarkers, including HOMA-IR, uric acid and hsCRP, also improved after survodutide treatment to a greater degree than after placebo treatment (Table 2).

**Table 2:** Change in cardiometabolic parameters from baseline to week 48

	Survodutide 6.0 mg (*n* = 146)	Placebo (*n* = 70)	ETD (95% CI)	*P* value
Waist circumference, absolute change, cm	−11.1 *n* = 84	−1.9 *n* = 39	−9.3 (−11.8 to −6.8)	<0.0001
SBP, absolute change, mmHg	−6.7 *n* = 86	0.7 *n* = 41	−7.4 (−11.3 to −3.4)	0.0003
DBP, absolute change, mmHg	−0.5 *n* = 86	2.2 *n* = 41	−2.7 (−5.3 to −0.03)	0.0478
HbA1c, absolute change, %	−0.6 *n* = 86	−0.02 *n* = 39	−0.6 (−0.8 to −0.3)	<0.0001
Without T2D	−0.6 *n* = 53	−0.1 *n* = 23	−0.4 (−0.7 to −0.2)	0.0019
With T2D	−0.6 *n* = 33	0.2 *n* = 16	−0.8 (−1.1 to −0.4)	<0.0001
Triglycerides^a^, relative change, %	−30.3 *n* = 86	0.7 *n* = 37	−30.8 (−39.1 to −21.4)	<0.0001
Total cholesterol^a^, relative change, %	−10.5 *n* = 86	−1.8 *n* = 40	−8.9 (−14.4 to −3.0)	0.0039
LDL cholesterol^a^, relative change, %	−10.8 *n* = 81	−2.4 *n* = 36	−8.6 (−17.4 to 1.2)	0.0834
HDL cholesterol^a^, relative change, %	5.0 *n* = 86	1.4 *n* = 37	3.6 (−1.7 to 9.2)	0.1834
VLDL cholesterol^a^, relative change, %	−32.8 *n* = 86	−0.6 *n* = 36	−32.5 (−41.0 to −22.7)	<0.0001
HOMA-IR^a,b^, relative change, %	−46.3 *n* = 72	−12.4 *n* = 31	−38.7 (−53.6 to −19.2)	0.0007
hsCRP, relative change, %	−46.2 *n* = 86	−7.2 *n* = 41	−42.1 (−56.6 to −22.7)	0.0003
Uric acid, relative change, %	−14.2 *n* = 86	2.8 *n* = 40	−16.9 (−22.8 to −11.1)	<0.0001

Data reported are derived from MMRM estimates. On-treatment, observed data are reported except for waist circumference and HOMA-IR, which reflect the efficacy estimand. ETD values may differ from those calculated due to rounding.

^a^ Measurements were analyzed on a log scale; therefore, only relative change (as percentages) are reported.

^b^ Participants without background insulin at baseline.

CI, confidence interval; DBP, diastolic blood pressure; ETD, estimated treatment difference; HbA1c, glycated hemoglobin; HDL, high-density lipoprotein; HOMA-IR, homeostatic model assessment for insulin resistance; hsCRP, high sensitivity C-reactive protein; LDL, low-density lipoprotein; MMRM, mixed model for repeated measures; SBP, systolic blood pressure; VLDL, very low-density lipoprotein.

### Safety

Overall, 87.0% of participants treated with survodutide and 78.6% of participants treated with placebo experienced at least one adverse event during the treatment period (Table 3). The most common adverse events were gastrointestinal in nature (predominantly nausea, vomiting, diarrhea and constipation) and were reported more frequently among participants treated with survodutide than with placebo (Table 3). These events occurred primarily during the dose-escalation period, were largely mild-to-moderate in intensity and resulted in treatment discontinuation in 19.9% of participants receiving survodutide versus 4.3% of those receiving placebo (Table 3). The incidence of serious adverse events was higher in the placebo group than in the survodutide treatment group.

**Table 3:** Adverse events and safety^a^

Participants with adverse events, no. (%)	Survodutide 6.0 mg (*n* = 146)	Placebo (*n* = 70)
Any adverse event	127 (87.0)	55 (78.6)
Adverse event leading to treatment discontinuation	34 (23.3)	7 (10.0)
Gastrointestinal disorders	29 (19.9)	3 (4.3)
Adverse event occurring in ≥10% of participants in any treatment group^b^		
Nausea	82 (56.2)	10 (14.3)
Vomiting	62 (42.5)	5 (7.1)
Diarrhea	43 (29.5)	7 (10.0)
Constipation	41 (28.1)	7 (10.0)
Decreased appetite	17 (11.6)	7 (10.0)
Upper respiratory tract infection	16 (11.0)	4 (5.7)
Fatigue	15 (10.3)	4 (5.7)
Headache	11 (7.5)	9 (12.9)
Abdominal distension	11 (7.5)	8 (11.4)
Serious adverse event	14 (9.6)	8 (11.4)
Adverse event leading to death	0 (0.0)	1 (1.4)^c^
Specific safety topics of interest		
Gastrointestinal disorders^d^	113 (77.4)	30 (42.9)
Liver-related events^e^	2 (1.4)	0 (0.0)
Cholelithiasis^b,f^	1 (0.7)	0 (0.0)
Cholecystitis^b,f^	1 (0.7)	0 (0.0)
Hypersensitivity	12 (8.2)	9 (12.9)
Acute kidney injury^b^	0 (0.0)	3 (4.3)
Hypotension and syncope^e^	1 (0.7)	1 (1.4)
Hypoglycemia^e^	0 (0.0)	2 (2.9)
Hyperglycemia^e^	2 (1.4)	6 (8.6)
Suicidal ideation and behavior^e^	0 (0.0)	2 (2.9)
Major depressive disorder^e^	4 (2.7)	0 (0.0)
Diabetic retinopathy^e^	2 (1.4)	0 (0.0)
Adjudication-confirmed events		
5P-MACE	2 (1.4)	1 (1.4)
Drug-induced liver injury	0 (0.0)	0 (0.0)
Pancreatic cancer	0 (0.0)	1 (1.4)^c^
Medullary thyroid cancer	0 (0.0)	0 (0.0)
Acute pancreatitis	0 (0.0)	0 (0.0)
Asymptomatic pancreatic hyperenzymemia	1 (0.7)	0 (0.0)

^a^ Includes adverse events that occurred on-treatment (onset on or after the first dose of study medication and up to 28 days after the last dose, corresponding to the predefined residual effect period for survodutide).

^b^ Preferred terms in the Medical Dictionary for Regulatory Activities, version 28.1.

^c^ Metastatic pancreatic cancer.

^d^ Based on SOC gastrointestinal disorders.

^e^ Based on grouped terms; includes nonserious events within grouped terms.

^f^ Reported in the same participant.

5P-MACE, 5-point major adverse cardiovascular events; SOC, system organ class.

**Extended Data Fig. 5:**
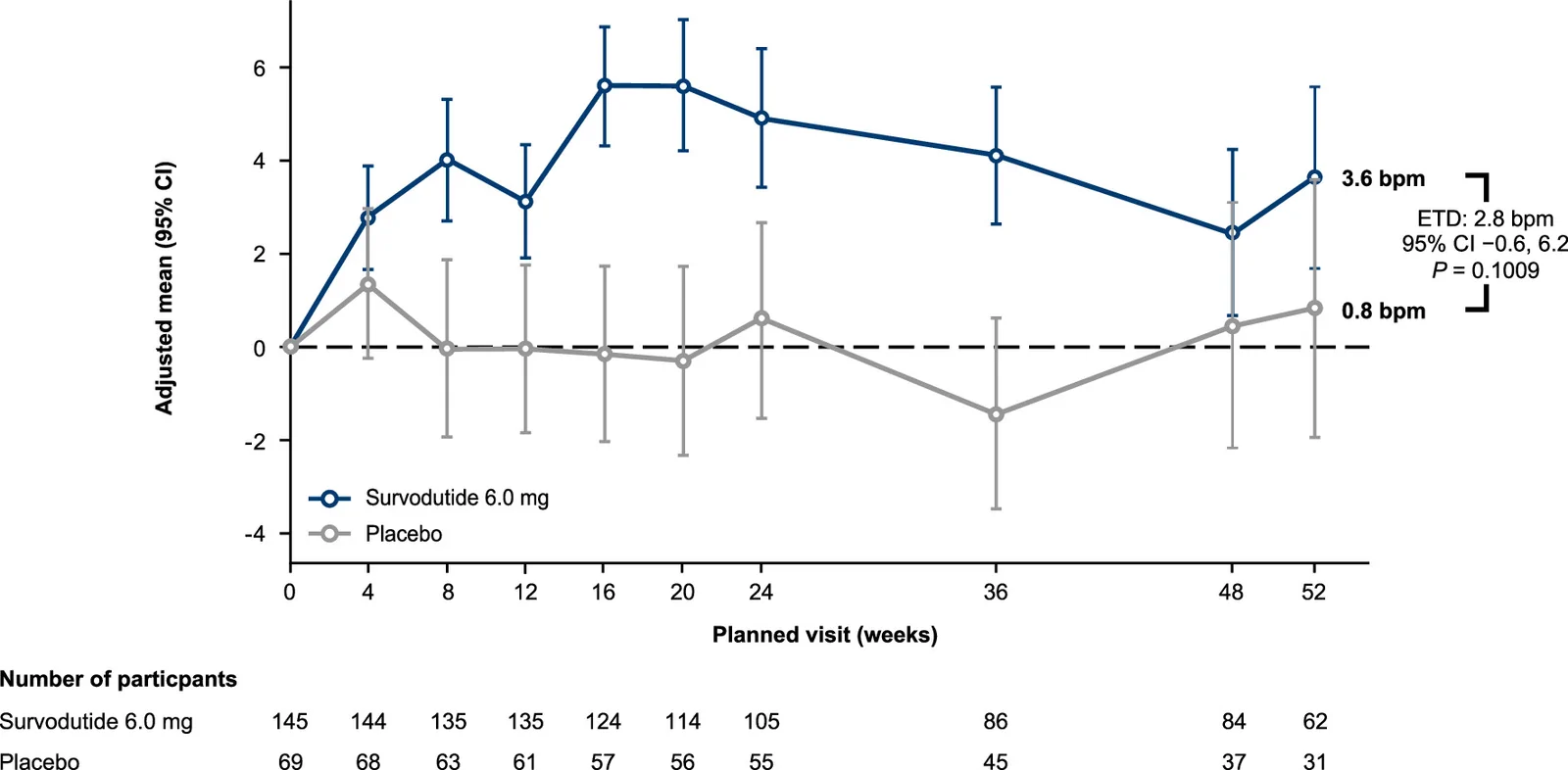
Absolute change in heart rate from baseline to week 52 (MMRM analysis; on-treatment)^a^. ^a^Absolute change in heart rate from baseline to week 52, derived from an MMRM analysis (two-sided test). CI, confidence interval; ETD, estimated treatment difference. Source data

Among the safety topics of interest (Table 3), there were no cases of adjudication-confirmed drug-induced liver injury, acute pancreatitis, pancreatic cancer or thyroid cancer in the survodutide group. Hypoglycemia and hyperglycemia events were reported in a higher frequency in the placebo group. At week 52, the mean change in heart rate from baseline was an increase of 3.6 beats per minute (BPM) in participants receiving survodutide compared to a 0.8-BPM increase in the placebo group (Extended Data Fig. 5). No participants experienced a newly occurring corrected QT using Fridericiaʼs formula (QTcF)^27^ interval >500 ms or a QTcF increase from baseline exceeding 60 ms.

## Discussion

This multicenter phase 3 trial evaluated the glucagon receptor/GLP-1R dual agonist survodutide in participants with obesity and at-risk MASLD as determined by MASLD and evidence of liver inflammation and/or fibrosis using NITs or liver biopsy-confirmed MASH. In most participants, baseline values of NITs were most consistent with early disease characterized by excess liver fat and inflammation, with no or only mild-to-moderate fibrosis. In this population, survodutide resulted in a greater proportion of participants achieving ≥30% reduction in LFC assessed by MRI-PDFF than placebo, with six in 10 participants achieving normalization of LFC (<5%). Survodutide also resulted in statistically and clinically significant reductions in body weight as well as improvements in multiple liver-related NITs and cardiometabolic parameters.

A relative reduction of ≥30% in MRI-PDFF-assessed LFC has been shown to be associated with a higher likelihood of histologic improvement and MASH resolution^28^, and, for people with MASH and fibrosis stage F2–F3, a ≥50% reduction in LFC has correlated with fibrosis regression^29^. In the present trial, a substantial proportion of participants experienced these levels of reduction in MRI-PDFF-assessed LFC with survodutide: 84.2% had a reduction of ≥30%, 75.3% had a reduction of ≥50% and 55.5% had a reduction of ≥70%. These findings are consistent with the previously reported phase 2 trial of survodutide in people with biopsy-confirmed MASH and fibrosis stage 1–3, in which 87.0% of participants treated with survodutide (6.0 mg) versus 19.7% treated with placebo had a ≥30% relative reduction in MRI-PDFF-assessed LFC over 48 weeks^24^. Furthermore, 61.0% of participants in the present trial had LFC <5% with survodutide at week 48 compared to 5.7% with placebo. Given that lipotoxicity is a driver of inflammation and fibrosis^30^, these data suggest the potential for survodutide treatment to support meaningful improvement in several of the proposed drivers of MASH and hepatic fibrosis, addressing an area of high unmet need^11,20^.

Based on mean values of liver fibrosis biomarkers at baseline, the study population could be classified as having fibrosis ranging from absent to mild or moderate at baseline. The mean baseline FIB-4 score was 1.3, which, in published clinical practice guidelines, is a requirement for further testing, and liver stiffness by MRE (mean 2.8 kPa) reflected a very low likelihood of fibrosis (advanced fibrosis is likely at ≥3.63 kPa and unlikely at <2.55 kPa)^2,9^. There were no differences in liver stiffness assessed by MRE between the survodutide and placebo groups at week 48. Although the lack of improvement in MRE could reflect a lack of biological effect of the treatment, the low baseline value of liver stiffness as assessed by MRE likely generated a ‘floor effect’ that hampered the ability of MRE to detect meaningful changes in this endpoint^31^. Similar findings were reported in a phase 1 trial of semaglutide in people with MASH who had a mean MRE-assessed liver stiffness of 2.95−3.08 kPa at baseline. The authors of that report also hypothesized that the sensitivity of MRE may be reduced in people with less severe liver fibrosis^32^. By contrast, liver stiffness as assessed by VCTE, which was indicative of mild-to-moderate fibrosis at baseline (advanced fibrosis is likely at ≥12 kPa and unlikely at <8 kPa)^2,9^, was reduced after survodutide treatment compared to placebo at week 48. BMI is an important determinant of discordance between MRE and VCTE for fibrosis staging, raising the possibility of fibrosis overestimation by VCTE^33^. However, the ELF score, which is a marker of extracellular matrix turnover and fibrogenesis, at baseline also reflected mild-to-moderate fibrosis (advanced fibrosis is likely at ≥9.8 and unlikely at <7.7)^2,9^ and showed an absolute reduction of 0.34 after 48 weeks of treatment with survodutide. Improvements in NITs related to liver inflammation, including cT1, ALT and AST, and in liver volume as assessed by MRI were observed after survodutide treatment. An absolute reduction in cT1 of ≥80 ms from baseline was previously shown to correspond to a histological 2-point decrease in the nonalcoholic fatty liver disease activity score (NAS)^34^. Collectively, these findings suggest that early and effective treatment may reduce LFC in people with obesity and at-risk MASLD, with the potential for a direct effect of survodutide on the liver to reduce inflammation and either suppress fibrosis progression or promote fibrosis regression.

The broader range of pharmacological actions resulting from dual receptor agonism with survodutide could provide clinical advantages over GLP-1R monoagonist therapies for MASH^35^. In a previous analysis of a phase 2 trial in MASH and fibrosis, survodutide demonstrated early and sustained improvements in serological biomarkers versus placebo^36,37^. In a mediation analysis from a MASH phase 2 trial with survodutide, NIT endpoints related to improvements in liver inflammation and fibrosis (for example, ELF score, ALT and AST) were shown to be predominantly weight loss independent, consistent with the potential for complementary additive or synergistic effects of glucagon receptor agonism on the liver^36^. The ongoing LIVERAGE phase 3 clinical trial program of survodutide for the treatment of MASH with moderate-to-advanced fibrosis (stage F2–F3) and for the treatment of MASH-associated compensated cirrhosis (stage F4) will assess the effects of survodutide on a more advanced MASH population^38,39^.

Because people with MASLD/MASH have a substantially increased risk of major adverse cardiovascular outcomes, it is noteworthy that survodutide treatment in this population is associated with improvements in several key markers of cardiovascular risk, including SBP, DBP, lipids, HOMA-IR, hsCRP, uric acid and HbA1c^40^. The reduction in body weight in this trial is similar to other MASLD/MASH trials with a mixed T2D population^24,32^. An improvement in LFC may have led to these improved metabolic functions and decreased cardiometabolic risk^40^. In ASCOT, a randomized controlled trial in the UK, participants with hypertension treated with blood pressure-lowering and lipid-lowering medications had fewer cardiovascular events 10 years after the trial ended^41^. This suggests that effective treatment of MASLD early in the disease course, along with improvement in cardiometabolic parameters, may prevent progression of cardiovascular disease and reduce the risk of future cardiac events. The mechanisms through which survodutide mediates its effects on liver and cardiometabolic health remain to be fully elucidated. However, the results of this study suggest that survodutide has the potential to exert beneficial effects on obesity and liver disease through multiple mechanisms. Direct actions of glucagon receptor agonism in the liver include increased fatty acid oxidation, which may enhance liver fat clearance; there is also a potential for liver-targeted effects that enhance the beneficial effect on obesity and body fat regulation^23,35,42–45^.

Glucagon receptor agonism and GLP-1R agonism have opposing effects on blood glucose levels; therefore, the optimal balancing of these receptors in dual agonists is important for the maintenance of glucose homeostasis. Survodutide has an eight-fold greater activation of the human GLP-1R than the glucagon receptor in vitro^23^. A previous phase 2 trial in participants with T2D found an improvement in HbA1c with survodutide^26^. In the present study, we demonstrate a reduction in body weight and improvements in liver and cardiometabolic health, including glucose-related parameters, with no imbalance of hyperglycemic events overall, a pattern consistent with survodutide having an optimized balance of glucagon receptor and GLP-1R activation that supports metabolic benefits without compromising glucose regulation.

Survodutide treatment was associated with a safety profile consistent with other medications that include GLP-1R agonist activity. Adverse gastrointestinal events were the most frequently reported and were the most common cause of treatment discontinuation. These adverse effects occurred most commonly during the dose-escalation period and resolved with longer exposure to the drug. Because tolerability early in the treatment course could be a key determinant of treatment persistence, efforts to further decrease gastrointestinal intolerance would be beneficial. The protocol for this study initially allowed limited flexibility in dose escalation. Although the protocol was amended to enhance flexibility of dosing, the change likely took place too late in this study to have a meaningful effect on participant tolerance. The variability in tolerance to the prescribed dose-escalation protocol underscores the importance of individualized dose management during the early phase of treatment to achieve optimal therapeutic benefit, the approach most commonly taken with other medications containing GLP-1R agonist activity in real-world clinical practice^46–48^. Other ongoing studies in the phase 3 SYNCHRONIZE obesity program, including SYNCHRONIZE-CVOT, and studies in the ongoing LIVERAGE program have allowed for greater dose flexibility. There were no adjudication-confirmed cases of drug-induced liver injury, acute pancreatitis, pancreatic cancer or thyroid cancer in the survodutide group. At week 52, survodutide was associated with a modest mean increase in heart rate relative to placebo, consistent with previous reports for other drugs containing GLP-1R agonist activity^24,25^, without evidence of clinically relevant QTcF prolongation.

The present study was conducted in participants with obesity and what we have defined as at-risk MASLD, as determined by liver steatosis in the absence of secondary causes of hepatic fat accumulation and evidence of inflammation and/or fibrosis by one or more widely used NITs. Although most participants did not meet formal criteria for MASH, they are representative of patients in clinical practice likely to be considered for treatment based primarily on NITs. A recent US real-world study reported that liver biopsy was performed in only 10% of patients with newly diagnosed MASH^12^, similar to the proportion with biopsy-confirmed MASH in this trial. The inclusion of participants based on NITs without a requirement for biopsy, and the beneficial effects of survodutide in the population studied, provide support for the efficacy and safety of this treatment for people with MASLD who have not yet progressed to advanced fibrosis or cirrhosis, which is the profile of most patients with MASLD/MASH seen in the broader medical community^11^.

Limitations of this study include its relatively short duration (48 weeks), which precluded assessment of longer-term efficacy and safety outcomes. Additionally, the global reach of the trial was limited because of rapid recruitment among research sites in the United States, restricting participating sites to only two countries (United States and Spain), where recruited participants were mostly White, limiting the generalizability. Other ongoing studies in the SYNCHRONIZE program are evaluating the effects of survodutide in people with broader ethnic and racial representation.

In conclusion, this phase 3 trial demonstrated clinically meaningful reductions in LFC and body weight after 48 weeks of treatment with weekly survodutide 6.0 mg in participants with obesity and at-risk MASLD. These results in participants whose disease was predominantly defined by NITs have high relevance for clinical practice. Improvements in LFC, body weight and multiple markers of liver and cardiometabolic health with survodutide treatment in participants with early-stage MASLD suggest that this therapeutic agent has important benefits for reducing liver inflammation and cardiometabolic risk and underscore its potential for improving long-term outcomes in people with obesity and metabolic liver disease.

## Methods

### Trial design

This phase 3, randomized, double-blind, placebo-controlled trial was conducted at 31 sites in the United States and one in Spain to examine the efficacy and safety of once-weekly survodutide 6.0 mg together with dietary and exercise counseling in participants with obesity and at-risk MASLD (NCT06309992). The protocol was approved by the Advarra central institutional review board and the European Medicines Agency, and the trial was conducted in accordance with the principles of the Declaration of Helsinki and Good Clinical Practice guidelines of the International Council for Harmonisation. All participants provided written informed consent before enrollment. The sponsor (Boehringer Ingelheim) designed the trial with the assistance of outside consultants; trial site investigators were responsible for data collection, IQVIA performed site monitoring and data management and the sponsor conducted the data analysis. Recruitment took place between April and November 2024.

### Participants

Eligibility criteria included age ≥18 years, BMI ≥30 kg m^−^^2^ or BMI ≥27 kg m^−^^2^ with at least one obesity complication (hypertension, dyslipidemia, obstructive sleep apnea, cardiovascular disease and/or T2D), a history of at least one patient-reported unsuccessful dietary effort to lose body weight and at-risk MASLD, defined by hepatic steatosis (MRI-PDFF ≥8%) in the absence of secondary causes of hepatic fat accumulation and at least one of the following: (1) MRE ≥2.61 kPa and <4.68 kPa; (2) MRI-AST (MAST) score ≥0.242; (3) FibroScan-AST (FAST) score ≥0.5; (4) FibroScan-assessed VCTE ≥8 kPa and <20 kPa; (5) FIB-4 score ≥2.67 and <3.48; (6) ELF score >7.7 and <11.3; (7) cT1 ≥875 ms; or (8) recent liver biopsy (within 3 years of screening) consistent with MASH (presence of hepatic steatosis, hepatic inflammation and hepatocyte ballooning) with stage 0–3 fibrosis according to the NASH Clinical Research Network classification^49^. Overall, the inclusion criteria were meant to identify MASLD at risk of disease progression based on NITs related to liver inflammation and/or fibrosis that is commonly associated with obesity and seen in many nonspecialized clinical practice environments. Key exclusion criteria included current or history of significant alcohol consumption; history of other chronic liver diseases; intake of medications associated with liver injury, hepatic steatosis or steatohepatitis; current or history of cirrhosis; self-reported body weight variation of >5% within 3 months before screening; and medications for obesity within 3 months before screening. A full list of eligibility criteria is provided in the Supplementary Information (Appendix B). To ensure that enough male and female trial participants were enrolled and randomized, a cap of approximately 70% was set for the number of female trial participants randomized.

### Randomization and treatment

Participants were randomly assigned in a 2:1 ratio to receive once-weekly subcutaneous injections of survodutide 6.0 mg (maintenance dose) or placebo (Extended Data Fig. 1). The randomization list was generated using validated software, which involved a pseudorandom number generator, and was stratified according to the presence or absence of T2D.

The treatment duration was 48 weeks, which included a dose-escalation period of 24 weeks for uptitration of survodutide to 6.0 mg. After escalation, the dose was maintained for 24 weeks until the end of treatment (dose-maintenance period); dose reduction was permitted, and 2.4 mg was the lowest maintenance dose allowed. After completion of the main treatment period of 48 weeks, participants had the option to enter an extended treatment period, which included an additional 4 weeks at the previously assigned dose for the assessment of safety. All participants received counseling for diet (aiming for a reduction of approximately 500 kcal per day) and physical activity (with a recommendation of at least 150 minutes of exercise per week).

### Endpoints

The co-primary endpoints were the proportion of participants with ≥30% reduction in LFC assessed by MRI-PDFF and percentage change in body weight, each from baseline to week 48. The secondary endpoints (assessed from baseline to week 48) were absolute and percentage change in MRI-PDFF-assessed LFC (%), ALT levels (U l^−1^), waist circumference (cm), HOMA-IR, MRE-assessed liver stiffness (kPa), MRI-assessed liver volume (ml) and the proportion of participants with absolute reduction in cT1 levels of ≥80 ms. Additional endpoints included changes (from baseline to week 48) in other imaging-based and serum-based NITs, including measures of liver fat, liver fibrosis, liver inflammation and cardiometabolic factors.

Safety assessments included adverse events, changes in physical examination, vital signs, safety laboratory parameters, 12-lead electrocardiograms, Columbia-Suicide Severity Rating Scale (C-SSRS) and the Patient Health Questionnaire 9-item depression module (PHQ-9).

The trial used MRI scanners of either 1.5-Tesla or 3-Tesla field strength (manufactured by GE, Philips or Siemens), all equipped with standard thoracic MRI coils. The MRI-PDFF values were derived from the multiecho Dixon sequence, a well-established method for robust water–fat separation and quantitative fat assessment. cT1 values were derived from a MOLLI-type sequence and analyzed using Perspectum’s proprietary LiverMultiScan post-processing medical device (LMS version 5) software. Standardized T1-weighted sequence was used for assessing liver volume. MRE was performed using standardized spin echo or gradient echo sequences with Resoundantʼs active and passive drivers. A 4-hour fasting period was required prior to the MRE scan. The MRI component of the trial was conducted in collaboration with an external imaging vendor (Perspectum Ltd.) and included blinded centralized reads to maintain objectivity. A Trial Imaging Manual was provided to harmonize imaging acquisition protocols, and an Imaging Charter was developed to guide the blinded central read process and analysis.

FibroScan (Echosens) was used to assess liver stiffness via VCTE (a surrogate of fibrosis) and LFC via the controlled attenuation parameter (CAP; a surrogate of steatosis). Only measurements meeting predefined quality criteria were included: (1) ≥3 hours of fasting; (2) ≥10 valid measurements; and (3) VCTE interquartile range / median ratio ≤30%.

### Statistical analysis

A sample size of 156 participants (104 randomized to survodutide and 52 to placebo) was calculated to provide 90% overall power to demonstrate superiority of survodutide 6.0 mg over placebo for both co-primary endpoints, each at a two-sided significance level of 0.05, assuming an early treatment discontinuation rate of 15%. During the course of the trial, the number of participants increased to approximately 220 owing to an unexpected sharp drop in the screening failure percentage after recruitment closure. For the co-primary endpoint of percentage reduction in LFC of ≥30% from baseline to week 48, a placebo effect of 30% and a survodutide effect of 60% were assumed, whereas participants with early treatment discontinuation were assumed to be nonresponders. For the co-primary endpoint of percentage change in body weight from baseline to week 48, power was determined using a predicted placebo effect of 3% and a survodutide effect of 10% (assumed in a mixed population 1:1 ratio with and without T2D; 12% in participants without T2D, 8% in participants with T2D), and standard deviations of 6% for placebo and 10% for survodutide were assumed, whereas participants with early treatment discontinuation were assumed to respond the same as those completing the trial on placebo.

Data from all participants who received at least one dose of the trial drug were used to analyze the efficacy and safety data. A primary and a supplementary estimand were prespecified: the treatment regimen estimand reflected the treatment effect in the presence of predefined intercurrent events, including intake of obesity management medications prohibited by the protocol and early treatment discontinuation, and the efficacy estimand reflected the treatment effect as if all trial participants had adhered to treatment and had not received any obesity management medications prohibited by the protocol. Co-primary endpoints were evaluated primarily for the treatment regimen estimand and additionally for the supplementary efficacy estimand. Secondary and further endpoints were evaluated for the efficacy estimand or based on observed, on-treatment data, as prespecified in the statistical analysis plan. Additional details are provided in the Supplementary Information (Appendix B). SAS version 9.4 or later was used for all analysis. Where statistical analyses require the use of R instead of SAS, R version 4.2.2 or later was used.

Adverse events are reported descriptively and coded using the Medical Dictionary for Regulatory Activities (https://www.meddra.org/).

### Reporting summary

Further information on research design is available in the Nature Portfolio Reporting Summary linked to this article.

## Online content

Any methods, additional references, Nature Portfolio reporting summaries, source data, extended data, supplementary information, acknowledgements, peer review information; details of author contributions and competing interests; and statements of data and code availability are available at 10.1038/s41591-026-04479-3.

## Supplementary information

Supplementary Information Trial committees and investigators, Supplementary Methods, clinical trial protocols and statistical analysis plan.

Reporting Summary

## Source data

Source Data Fig. 2 Statistical Source Data

Source Data Fig. 3 Statistical Source Data

Source Data Extended Data Fig. 2 Statistical Source Data

Source Data Extended Data Fig. 3 Statistical Source Data

Source Data Extended Data Fig. 4 Statistical Source Data

Source Data Extended Data Fig. 5 Statistical Source Data

## Data Availability

To ensure independent interpretation of clinical study results and enable authors to fulfill their role and obligations under ICMJE criteria, Boehringer Ingelheim grants all external authors access to relevant clinical study data. In adherence with the Boehringer Ingelheim policy on transparency and publication of clinical study data, scientific and medical researchers can request access to clinical study data, typically 1 year after the approval has been granted by major regulatory authorities or after termination of the development program. Initial feedback is provided within 21 days of receipt of the data-sharing request. Upon approval, and governed by a legal agreement, data are shared in a secured data access system for a limited period of 1 year, which may be extended upon request. Prior to providing access, clinical study documents and data will be examined and, if necessary, redacted and deidentified to protect the personal data of study participants and personnel and to respect the boundaries of the informed consent of the study participants. Researchers should use the https://vivli.org/ link to request access to study data and visit www.mystudywindow.com/msw/datasharing for further information. Source data are provided with this paper.

## Extended data

**Extended Data Table 1:** Additional baseline characteristics

	Survodutide 6.0 mg (*N* = 146)	Placebo (*N* = 70)	Total (*N* = 216)
**Demographic characteristics**			
Country, no. (%)			
United States of America	145 (99.3)	70 (100.0)	215 (99.5)
Spain	1 (0.7)	0 (0.0)	1 (0.5)
**Clinical characteristics**			
Edmonton Obesity Staging System, no. (%)			
Stage 0	3 (2.1)	1 (1.4)	4 (1.9)
Stage 1	32 (21.9)	11 (15.7)	43 (19.9)
Stage 2	111 (76.0)	55 (78.6)	166 (76.9)
Stage 3	0 (0.0)	3 (4.3)	3 (1.4)
Type of cardiovascular disease, no. (%)			
NYHA class II–III	0 (0.0)	2 (2.9)	2 (0.9)
History of ischemic stroke	0 (0.0)	0 (0.0)	0 (0.0)
History of hemorrhagic stroke	0 (0.0)	0 (0.0)	0 (0.0)
History of cerebrovascular revascularization procedure	0 (0.0)	0 (0.0)	0 (0.0)
MI	2 (1.4)	0 (0.0)	2 (0.9)
Coronary artery disease	5 (3.4)	1 (1.4)	6 (2.8)
Peripheral vascular disease	3 (2.1)	1 (1.4)	4 (1.9)
Fasting plasma glucose, mean ± s.d., mg/dl	112.9 ± 39.1 *n* = 142	115.0 ± 39.6 *n* = 67	113.6 ± 39.1 *n* = 209
Estimated GFR, mean ± s.d., ml/min/1.73 m^2^	85.8 ± 18.1 *n* = 146	82.8 ± 18.0 *n* = 70	84.8 ± 18.0 *n* = 216

GFR, glomerular filtration rate, MI, myocardial infarction; NYHA, New York Heart Association; s.d., standard deviation.

**Extended Data Table 2:** MRI-PDFF-assessed LFC reduction of ≥ 30% by subgroup at Week 48

Subgroup	*N*	Survodutide 6.0 mg	*N*	Placebo
T2D, no. (%)				
No	53	42 (79.2%)	25	6 (24.0%)
Yes	31	26 (83.9%)	15	4 (26.7%)
Age, no. (%), years				
<65	64	50 (78.1%)	31	5 (16.1%)
≥65	20	18 (90.0%)	9	5 (55.6%)
Sex, no. (%)				
Male	34	27 (79.4%)	20	4 (20.0%)
Female	50	41 (82.0%)	20	6 (30.0%)
BMI, no. (%), kg/m^2^				
<27	0		0	
≥27 to <30	6	5 (83.3%)	2	0
≥30 to <35	19	15 (78.9%)	10	2 (20%)
≥35 to <40	26	23 (88.5%)	11	5 (45.5%)
≥40	33	25 (75.8%)	17	3 (17.6%)

Treated set, on-treatment; observed data. BMI, body mass index; LFC, liver fat content; MRI-PDFF, magnetic resonance imaging-proton density fat fraction; T2D, type 2 diabetes.

**Extended Data Table 3:** Percentage change in body weight by subgroup at Week 48

Subgroup	*N*	Survodutide 6.0 mg	*N*	Placebo
T2D, mean ± s.d.				
No	53	−13.4 ± 7.0	26	−1.2 ± 4.0
Yes	33	−9.9 ± 8.2	16	−2.0 ± 4.3
Age, mean ± s.d., years				
<65	65	−12.2 ± 7.7	32	−1.0 ± 3.7
≥65	21	−11.6 ± 7.4	10	−2.9 ± 5.2
Sex, mean ± s.d.				
Male	34	−11.2 ± 6.0	21	−1.5 ± 3.4
Female	52	−12.6 ± 8.5	21	−1.5 ± 4.8
BMI, mean ± s.d., kg/m^2^				
<27	0		0	
≥27 to <30	6	−16.0 ± 8.3	2	1.3 ± 0.1
≥30 to <35	19	−11.2 ± 8.3	10	−1.4 ± 5.2
≥35 to <40	27	−13.0 ± 7.5	11	−0.4 ± 4.3
≥40	34	−11.1 ± 7.1	19	−2.5 ± 3.4

Treated set, on-treatment; observed data. BMI, body mass index; s.d, standard deviation; T2D, type 2 diabetes.
