# Author Correction: Survodutide in adults with obesity and metabolic dysfunction-associated steatotic liver disease: SYNCHRONIZE-MASLD, a randomized, double-blind, placebo-controlled phase 3 trial

**DOI:** 10.1038/s41591-026-04650-w

**Published:** 2026-08-25

**Authors:** Lee M. Kaplan, Elena Startseva, Carel W. le Roux, Sean Wharton, Biykem Bozkurt, Daniel F. Mazo, Jessica von Schlippenbach, Sandra González Maldonado, Samina Ajaz Hussain, Guy W. Neff, Yaneicy Gonzalez Rojas, Charles Smith, Ramy Younes, Arun J. Sanyal

**Affiliations:** 1 The Obesity and Metabolism Institute, Boston, MA USA; 2 Geisel School of Medicine at Dartmouth, Hanover, NH USA; 3 Boehringer Ingelheim International GmbH, Ingelheim am Rhein, Germany; 4 University College Dublin School of Medicine, Dublin, Ireland; 5 Ulster University, Coleraine, UK; 6 McMaster University, Hamilton, Ontario Canada; 7 University of Toronto, Toronto, Ontario Canada; 8 Winters Center for Heart Failure Research, Cardiovascular Research Institute, Baylor College of Medicine, Houston, TX USA; 9 Boehringer Ingelheim International GmbH, Biberach, Germany; 10 Boehringer Ingelheim Pharma GmbH & Co. KG, Biberach, Germany; 11 Covenant Metabolic Specialists, LLC, Sarasota and Fort Myers, FL USA; 12 Optimus U Corp., Miami, FL USA; 13 Stravitz Sanyal Institute for Liver Disease and Metabolic Health, Division of Gastroenterology, Hepatology and Nutrition, Virginia Commonwealth University School of Medicine, Richmond, VA USA; 14 Hospital Universitario La Paz, Madrid, Spain; 15 Liver Unit, Vall d’Hebron University Hospital, VHIR, Universitat Autònoma de Barcelona, CIBERehd, Barcelona, Spain; 16 Velocity Clinical Research, Gardena, CA USA; 17 Clinical Trials of Texas, LLC, San Antonio, dba Flourish Research, San Antonio, TX USA; 18 Washington University School of Medicine, St. Louis, MO USA; 19 Fleming Island Center for Clinical Research, Fleming Island, FL USA; 20 Kansas Medical Clinic, Topeka, KS USA; 21 Excel Medical Clinical Trials, LLC, Boca Raton, dba Flourish Research, Boca Raton, FL USA; 22 Amel Med, LLC, Georgetown, TX USA; 23 Ark Clinical Research – PARENT, Long Beach, CA USA; 24 Louisiana Research Center, LLC, Shreveport, LA USA; 25 Indiana University, Indianapolis, IN USA; 26 Biopharma Informatic, LLC, McAllen, TX USA; 27 Health Awareness, Inc., Jupiter, FL USA; 28 DSI Research Northridge, LLC, Dayton, OH USA; 29 University of Iowa Health Care, Iowa City, IA USA; 30 Velocity Clinical Research, North Hollywood, CA USA; 31 Velocity Clinical Research, Hallandale Beach, FL USA; 32 Panax Clinical Research, LLC, Miami Lakes, FL USA; 33 Ark Clinical Research, Fountain Valley, CA USA; 34 GI Select Health Research, LLC, Richmond, VA USA; 35 Catalina Research Institute, Montclair, CA USA; 36 Velocity Clinical Research, Austin, TX USA; 37 Velocity Clinical Research, Santa Ana, CA USA; 38 Velocity Clinical Research, Waco, TX USA; 39 Accurate Clinical Research, Inc., Richmond, TX USA; 40 Springfield Clinic, Springfield, IL USA; 41 Segal Drug Trials, Delray Beach, FL USA; 42 Nature Coast Clinical Research, Inverness, FL USA

**Keywords:** Outcomes research, Diagnostic markers

Correction to: *Nature Medicine* https://doi.org/10.1038/s41591-026-04479-3, published online 7 June 2026

In the version of the article initially published, among the SYNCHRONIZE-MASLD Investigators, Juan M. Pericás (Liver Unit, Vall d’Hebron University Hospital, VHIR, Universitat Autònoma de Barcelona, CIBERehd, Barcelona, Spain) and Michael Zimmerman (Velocity Clinical Research, Austin, TX, USA) were not included in the author list; Antonio Olveira (Hospital Universitario La Paz, Madrid, Spain) was listed with an incorrect surname (Antonio O. Martin) and an incorrect additional affiliation; and Rizwana Mohseni was listed with an incorrect second affiliation. In the “Participants” section of Results, there was a typographical error: in the sentence now reading “Treatment discontinuation occurred at a similar frequency in the survodutide (41.1% (60/146)) and placebo (40.0% (28/70)) arms,” “70” originally appeared as “72.” Additionally, in the caption to Extended Data Fig. 5, “week 52” now replaces the original “week 48.” The changes have been made in the HTML and PDF versions of the article.

