# Supplementary material for: Survodutide in adults with obesity and metabolic dysfunction-associated steatotic liver disease: SYNCHRONIZE-MASLD, a randomized, double-blind, placebo-controlled phase 3 trial
Source: Nat Med. 2026 Jun 7;32(8):2948–58. doi: 10.1038/s41591-026-04479-3 (PMC13472939; doi:10.1038/s41591-026-04479-3)
Supplement: Supplementary file 1 [file 41591_2026_4479_MOESM1_ESM.pdf]

nature medicine

Supplementary information https://doi.org/10.1038/s41591-026-04479-3

Survodutide in adults with obesity and metabolic dysfunction-associated steatotic liver disease: SYNCHRONIZE-MASLD, a randomized, double-blind, placebo-controlled phase 3 trial

In the format provided by the authors and unedited

CONTENTS

Trial committees and investigators (page 2)

Supplementary methods (page 5)

BI Trial No: 1404-0056 clinical trial protocol (CTP), version 1 (page 14)

BI Trial No: 1404-0056 CTP, final version including a summary of changes (page 132)

BI Trial No: 1404-0056 trial statistical analysis plan (TSAP), version 1, there have been no changes (page 287)

TRIAL COMMITTEES AND INVESTIGATORS

Trial committees

- *Executive Committee*
    - Carel W. leRoux, M.B., Ch.B., Ph.D (PI of SYNCHRONIZE global phase 3 program), University College Dublin, Dublin, Ireland and Ulster University, Coleraine, UK
    - Lee M. Kaplan, M.D., Ph.D (Chairman of SYNCHRONIZE global phase 3 program), Geisel School of Medicine at Dartmouth, Hanover, NH, USA
    - Sean Wharton, M.D., McMaster University, Hamilton, ON, Canada and University of Toronto, ON, Canada
    - Biykem Bozkurt, M.D., Ph.D, Baylor College of Medicine, Houston, TX, USA

- *Data Monitoring Committee*
    - Matthias Blüher, M.D., Ph.D (DMC chair), Leipzig University, Leipzig, Germany
    - Timothy Garvey, M.D. (DMC member), The Kirklin Clinic of UAB Hospital, Birmingham, AL, USA
    - Darren K. McGuire, M.D. (DMC member), PLLC, University of Texas Southwestern Medical Center, Dallas, TX, USA
    - Andreea Ciudin Mihai, M.D., Ph.D (DMC member), Hospital Universitari Vall d'Hebron, Barcelona, Spain
    - Javier Ampuero, M.D., Ph.D, M.Sc (DMC member), The Institute of Biomedicine of Seville, Seville, Spain
    - Tim Clayton, M.Sc (DMC statistician), London School of Hygiene & Tropical Medicine, London, UK
    - Detlef Schuppan, M.D. (DMC member), University Medical Center Mainz, Germany and Beth Israel Medical Center, Harvard Medical School, Boston, MA, USA

- *Clinical Event Committee*
    - Mark Petrie, M.D. (Chair, Cardiology), University of Glasgow, Glasgow, UK
    - Wolfram Doehner, M.D., Ph.D (Cardiology), Charité - Universitätsmedizin Berlin, Germany
    - Markus Haass, M.D. (Cardiology), Theresienkrankenhaus, Mannheim, Germany
    - Inder Anand, M.D., Ph.D (Cardiology), University of Minnesota Medical School, Minneapolis, MN, USA and VA Medical Center La Jolla, CA, USA
    - Steen Pehrson, M.D. (Cardiology), Copenhagen University Hospital-Rigshospitalet, Copenhagen, Denmark
    - Johannes Mann, M.D. (Cardiology), University of Erlangen-Nürnberg, Munich General Hospitals, Munich, Germany
    - Ninian Lang, M.D., Ch.B., Ph.D (Cardiology), University of Glasgow, Glasgow, UK
    - John Petrie, M.D. (Cardiology), Robertson Centre for Biostatistics and Clinical Trials Unit, University of Glasgow, Glasgow, UK
    - Michel Komajda, M.D. (Cardiology), Groupe Hospitalier Paris Saint Joseph, Paris, France

- Alejandro Rabinstein, M.D. (Neurology), Mayo Clinic College of Medicine, Rochester, MN, USA
- Hooman Kamel, M.D. (Neurology), Presbyterian Hospital/Weill Cornell Medical Center, New York, NY, USA
- Thorsten Steiner, M.D. (Neurology), Klinikum Frankfurt Höchst, Frankfurt, Germany
- Matthew Flaherty, M.D. (Neurology), University of Cincinnati College of Medicine, Cincinnati, OH, USA
- Pamela Munster, M.D. (Oncology), University of California San Francisco, San Francisco, CA, USA
- Brandon Smaglo, M.D. (Oncology), The University of Texas MD Anderson Cancer Center, Houston, TX, USA
- Daniel Palmer, M.D., Ph.D, (Oncology), University of Liverpool, Liverpool, UK
- Syed Hussain, M.D. (Oncology), University of Sheffield, Sheffield, UK
- James Lewis, M.D. (Gastroenterology & Hepatology), Medstar Georgetown University Hospital, Washington, D.C., USA
- Paola Casciato, M.D. (Gastroenterology & Hepatology), Hospital Italiano de Buenos Aires, Buenos Aires, Argentina
- Hannes Hagström, M.D. (Gastroenterology & Hepatology) Karolinska Institutet, Stockholm, Sweden and Karolinska University Hospital, Stockholm, Sweden
- Stephen Pappas, M.D. (Gastroenterology & Hepatology), Baylor College of Medicine Houston, TX, USA
- Raul Andrade, M.D. (Hepatology), University of Malaga, Malaga, Spain and University Hospital of Málaga, Malaga, Spain
- Laurent Castera, M.D. (Hepatology), University of Paris-Cité (Beaujon Hospital), Paris, France

List of investigators

- *Spain*
    - A. Olveira Martin, Hospital Universitario La Paz, Madrid; J.M. Pericas Pulido, Hospital Universitari Vall d'Hebron - VHIR.
- *United States of America*
    - M. Leibowitz, Velocity Clinical Research, Gardena, CA, USA; D. Denham, Clinical Trials of Texas, LLC San Antonio dba Flourish Research, San Antonio, TX, USA; K. Korenblat, Washington University School of Medicine, St. Louis. MO, USA; M. Herman, Fleming Island Center for Clinical Research, Fleming Island, FL, USA; S. Challa, Kansas Medical Clinic, Topeka, KS, USA; R. Youssef, Excel Medical Clinical Trials, LLC Boca Raton dba Flourish Research, Boca Raton, FL, USA; M. Khaja, Amel Med LLC, Georgetown, TX, USA; A. Vutikullird, Ark Clinical Research – PARENT, Long Beach, CA, USA; H. Aguilar, Louisiana Research Center, LLC, Shreveport, LA, USA; N. Chalasani, Indiana University, Indianapolis, IN, USA; J. Maher, Biopharma Informatic, LLC, McAllen, TX, USA; R. Surowitz, Health Awareness, Inc., Jupiter, FL, USA; C. Steimle, DSI Research Northridge LLC, Dayton, OH, USA; Y. Gonzalez Rojas, Optimus U Corp., Miami, FL, USA; A. Sanchez, University of Iowa Health Care, Iowa City, IA, USA; R. Espinoza Kavcioglu, Velocity Clinical Research, North Hollywood, CA, USA; J. Vijil, Velocity Clinical Research, Hallandale Beach, FL, USA; R. Perry, Panax Clinical Research, LLC, Miami Lakes, FL, USA; G. Neff, Covenant Metabolic Specialists, LLC, Fort Myers, FL, USA; M. Garcia Navarro, Ark Clinical Research, Fountain Valley, CA, USA; O. Khalid, GI Select Health Research LLC, Richmond, VA, USA; R. Mohseni, Catalina Research Institute, Montclair, CA, USA; M.P. Zimmerman, Velocity Clinical Research, Austin, TX, USA; J. Vu, Velocity Clinical Research, Santa Ana, CA, USA; G. Lucksinger, Velocity Clinical Research, Waco, TX, USA; C. Smith, Covenant Metabolic Specialists, LLC, Fort Myers, FL, USA; S. Iduru, Accurate Clinical Research, Inc., Richmond, TX, USA; D. Shuster, Springfield Clinic, Springfield, IL, USA; B. Segal, Segal Drug Trials, Delray Beach, FL, USA; P. Hellstern, Nature Coast Clinical Research, Inverness, FL, USA.

SUPPLEMENTARY METHODS

Inclusion and exclusion criteria

<u>Inclusion criteria</u>

For an eligible participant, all inclusion criteria must be answered 'yes' at screening and reconfirmed at baseline (i.e. before randomization), except for laboratory tests for which screening results are used for randomization.

1. Participants aged ≥18 years at the time of signing informed consent, and at least the legal age of consent in countries where it is >18 years.
2. Signed and dated written informed consent in accordance with International Council of Harmonisation-Good Clinical Practice and local legislation prior to admission to the trial.
3. Body mass index (BMI) ≥30 kg/m^2, or BMI ≥27 kg/m^2 and at least one obesity complication at screening:
    a. Hypertension (defined as repeated, i.e. at least three measurements in resting condition, systolic blood pressure [SBP] values of ≥140 mmHg and/or diastolic blood pressure [DBP] values of ≥90 mmHg in the absence of antihypertensive treatment, or intake of at least one anti-hypertensive drug to maintain normotensive blood pressure).
    b. Dyslipidemia (defined as at least one lipid-lowering treatment required to maintain normal blood lipid levels, or low-density lipoprotein (LDL) cholesterol ≥160 mg/dl (≥4.1 mmol/L) or triglycerides ≥150 mg/dl (≥1.7 mmol/L), or high-density lipoprotein (HDL) cholesterol <40 mg/dl (<1.0 mmol/L) for men or HDL cholesterol <50 mg/dl (<1.3 mmol/L) for women.
    c. Obstructive sleep apnea.
    d. Cardiovascular disease (e.g. heart failure [HF] with New York Heart Association [NYHA] functional class II–III, history of ischemic or hemorrhagic stroke or cerebrovascular revascularization procedure [e.g. carotid endarterectomy and/or stent], myocardial infarction [MI], coronary artery disease, or peripheral vascular disease).
    e. Type 2 diabetes (T2D; diagnosed at least 180 days prior to screening, with glycated hemoglobin [HbA1c] ≥6.5% [≥48 mmol/mol] and <10% [<86 mmol/mol] as measured by the central laboratory at screening).
4. History of at least one self-reported unsuccessful dietary effort to lose body weight.
5. Presumed/confirmed MASH: Evidence of hepatic steatosis (defined by a magnetic resonance imaging-proton density fat fraction ≥8% at screening) with the exclusion of secondary causes of hepatic fat accumulation such as significant alcohol consumption, other chronic liver diseases and/or steatogenic medications, AND at least one of the following:
    a. Magnetic resonance elastography (MRE) ≥2.61 and <4.68 kPa at screening, OR
    b. Magnetic resonance imaging (MRI)-aspartate aminotransferase (AST) (MAST) score ≥0.242 at screening, when MAST score = exp(MAST)/(1+exp[MAST])
    where MAST = –12.17 + 7.07 logMRE + 0.037 PDFF + 3.55 log AST, OR
    c. FibroScan®-AST (FAST) score ≥0.5 at screening, OR

d. FibroScan® vibration-controlled transient elastography (VCTE™) ≥8 kPa and <20 kPa at screening, OR
e. Fibrosis-4 (FIB-4) score ≥2.67 and <3.48 at screening, OR
f. Enhanced liver fibrosis (ELF™) score >7.7 and <11.3 at screening, OR
g. Iron-corrected T1 (cT1) ≥875 ms at screening, OR
h. Recent liver biopsy (within 3 years of screening) consistent with MASH (defined as the presence of steatosis, inflammation and ballooning) with stage ≤3 fibrosis according to the NASH Clinical Research Network classification.

6. Women of childbearing potential (WOCBP[a]) must be ready and able to use highly effective methods of birth control per ICH M3 (R2) that result in a low failure rate of less than 1% per year when used consistently and correctly. A list of contraception methods meeting these criteria was provided in the participant information.
7. In the investigator's opinion, participants are well-motivated, capable and willing to:
    a. Learn how to self-inject the investigational medicinal product (IMP), as required for this protocol (persons with physical limitations who are not able to perform the injections must have the assistance of an individual trained to inject the IMP) OR
    b. Inject the IMP or accept injection from a designated person
    c. Follow study procedures for the duration of the study, including, but not limited to: follow lifestyle advice (for example, dietary restrictions and exercise, and exercise plan), maintain a diary, complete required questionnaires, and handle the IMP as described in the instructions for use.

[a]A woman is considered of childbearing potential, i.e. fertile, following menarche and until becoming postmenopausal unless permanently sterile. Permanent sterilization methods include hysterectomy, bilateral salpingectomy and bilateral oophorectomy. Tubal ligation is NOT a method of permanent sterilization. A postmenopausal state is defined as no menses for 12 months without an alternative medical cause.

<u>Exclusion criteria</u>

For an eligible participant, all the following exclusion criteria must be answered 'no' at screening and reconfirmed at baseline (i.e. before randomization), except for laboratory tests for which screening results are used for randomization.

Liver

1. Current or history of significant alcohol consumption (defined as intake of >210 g/week in men and >140 g/week in women on average over a consecutive period of more than 3 months) or inability to reliably quantify alcohol consumption based on the investigator's judgement within the past 5 years.
2. Intake of medications associated with liver injury, hepatic steatosis or steatohepatitis.
3. History of other chronic liver diseases (e.g. viral hepatitis, autoimmune liver disease, primary biliary cholangitis, primary sclerosing cholangitis, Wilson's disease, hemochromatosis, alpha 1 antitrypsin deficiency, history of liver transplantation). Hepatitis B and C testing were done at visit 1. Participants with positive hepatitis B surface antigen should be excluded. Participants treated for hepatitis C must have a negative ribonucleic acid (RNA) test at screening and also be hepatitis C virus (HCV) RNA negative for at least 3 years prior to screening to be eligible for the trial. Trial

participants with positive HCV antibody and no history of HCV treatment require a negative HCV RNA test at screening to be eligible for the trial.

4. Cirrhosis based on clinical assessment, abdominal imaging, liver histology or noninvasive tests assessed at screening (ELF™ ≥11.3, FIB-4 ≥3.48, FibroScan® VCTE™ ≥20 kPa or MRE ≥4.68 kPa) or a history of cirrhosis.
5. Current decompensated liver disease or previous hepatic decompensation (ascites, spontaneous bacterial peritonitis, portal hypertension bleeding, hepatic encephalopathy, hepatorenal syndrome).
6. Previous or current evidence of portal hypertension (e.g. splenomegaly, esophageal varices or other portosystemic collateral pathways).
7. Any of the following liver laboratory test abnormalities at screening:
    a. Serum AST and/or alanine aminotransferase (ALT) elevation ≥5× upper limit of normal (ULN).
    b. Total serum bilirubin concentration ≥1.2× ULN (except for cases of known Gilbert's Syndrome).
    c. Alkaline phosphatase >2× ULN.
    d. International normalized ratio ≥1.3 (unless participant is on anticoagulants).

Obesity

8. Body weight variation (self-reported) >5% within 3 months before screening.
9. Medications for obesity within 3 months before screening.
10. Previous or planned (during the trial period) treatment for obesity with surgery or a weight loss device, or prior surgery of the GI tract that could interfere with body weight. The following are allowed: (1) liposuction and/or abdominoplasty, if performed >1 year before screening, (2) lap banding, if the band has been removed >1 year before screening, (3) intragastric balloon, if the balloon has been removed >1 year before screening, (4) duodenal-jejunal bypass sleeve, if the sleeve has been removed >1 year before screening, (5) appendectomy, (6) simple hernia repair, or (7) cholecystectomy.
11. Obesity induced by other endocrinologic disorders (i.e. Cushing Syndrome) or diagnosed monogenetic or syndromic forms of obesity (i.e. melanocortin 4 receptor deficiency, leptin deficiency, or Prader Willi Syndrome).

Diabetes

12. Treatment with medications for the indication of T2D.
13. Uncontrolled and potentially unstable diabetic retinopathy or maculopathy, verified by an eye examination within 3 months before screening or in the period between screening and randomization.

Mental health

14. Answered 'yes' to any of the suicide-related behaviors (actual attempt, interrupted attempt, aborted attempt, preparatory act or behavior) or to the nonsuicidal self-injurious behavior question on the 'Suicidal Behavior' section of the Columbia-Suicide Severity Rating Scale (C-SSRS) related to the past 2 years before screening visit up to and including the randomization visit.

15. Answered ‘yes’ to Question 4 (active suicidal ideation with some intent to act, without specific plan) or to Question 5 (active suicidal ideation with specific plan and intent) on the ‘Suicidal Ideation’ section of the C-SSRS from 3 months before screening visit up to and including the randomization visit.
16. Reported a history of psychiatric inpatient hospitalization (due to significant active or unstable major depressive disorder or other severe psychiatric disorder, e.g. schizophrenia, bipolar disorder, or other serious mood or anxiety disorder) within the past 1 year before screening, OR are not stabilized on their psychiatric medications (i.e. change of medication type or dosage adjustment within 8 weeks prior to screening visit or until randomization), OR major depressive symptoms defined as a Participant Health Questionnaire-9 score of ≥15 at screening or during the screening period (based on investigator’s judgement, this assessment can be repeated once within the screening window).
17. Participants with major depressive disorder or generalized anxiety disorder whose disease state is considered stable and expected to remain stable throughout the course of the study, in the opinion of the investigator, may be considered for inclusion if they are not on excluded medications.

Other medical

18. Any treatment with medications which may cause significant weight change started within 3 months prior to screening (included but not limited to systemic glucocorticoid therapy, tricyclic antidepressants, atypical antipsychotic, mood stabilizers, antihypertensive, antihistamines and anti-epileptics). Participants taking these medications for more than 3 months prior to screening with stable body weight are allowed.
19. Known clinically significant gastric emptying abnormality (e.g. severe diabetic gastroparesis or gastric outlet obstruction).
20. Uncontrolled hypertension (mean SBP ≥160 mmHg and/or mean DBP ≥100 mmHg, measured in at least two measurements performed 5 minutes apart) at screening.
21. Uncontrolled (as per investigators assessment) hypo- or hyperthyroidism at screening.
22. Renal impairment measured as estimated glomerular filtration rate <30 ml/min/1.73 m^2 by the central laboratory at screening.
23. History of either chronic or acute pancreatitis or elevation of serum lipase or serum pancreatic amylase >2× ULN as measured by the central laboratory at screening.
24. Major surgery performed within 3 months prior to randomization or planned for the duration of the trial.
25. Personal or family history of medullary thyroid carcinoma or multiple endocrine neoplasia syndrome type 2.
26. Calcitonin ≥100 ng/L (29.26 pmol/L) as measured by the central laboratory at screening.
27. Recent evidence (within 3 months prior to screening) of acute or unstable cardiovascular events, e.g. hospitalization for HF, acute coronary syndrome, unstable angina, MI, ischemic or hemorrhagic stroke, transient ischemic attack, and/or acute peripheral vascular event.
28. HF with NYHA functional class IV.

29. QTc (Fridericia) mean interval that is greater than 500 ms at screening (triplicate electrocardiogram) or personal or family history of long QT syndrome.
30. History of infection with human immunodeficiency virus (HIV) or positive HIV test at screening.
31. History of organ transplantation (corneal transplants [keratoplasty] allowed) or awaiting an organ transplant.
32. Hematological condition that may interfere with HbA1c measurement (e.g. hemolytic anemias, sickle cell disease).
33. Women who are pregnant, nursing or who plan to become pregnant while in the trial.
34. Known or suspected hypersensitivity to the IMP or related products.
35. Contraindication to MRI including, but not limited to, severe claustrophobia, inner ear implant, pacemakers or other implanted cardiac rhythm management devices, intracranial aneurism clips incompatible with MRI, any other metallic, non-MRI compatible implanted devices (e.g. hip joint replacement), a history of intra-orbital metal fragments that have not been removed, and weight or girth that exceeds scanner capabilities.
36. Confirmed diagnosis of malignancy (including remission) within 5 years prior to screening, except for basal- or squamous-cell carcinoma of the skin that has been treated successfully. Trial participants under evaluation for malignant disease currently are not eligible for trial participation.
37. Platelet count <140,000/mm^3.

General

38. Participants not expected to comply with the protocol requirements or not expected to complete the trial as scheduled (e.g. chronic alcohol or drug abuse or any other condition that, in the investigator's opinion, makes the participant an unreliable trial participant).
39. Participants who must or wish to continue the intake of prohibited medication or any drug considered likely to interfere with the safe conduct of the trial.
40. WOCBP who are using oral contraceptives during the screening visit and are:
    a. Not willing to change to nonoral contraceptives at least 7 days prior to first dose of IMP, OR
    b. Not willing to add an additional barrier method of contraception during the entire treatment period with IMP and 28 days thereafter.
41. Currently enrolled in another investigational device or drug trial, or less than 30 days or five half-lives, whichever is longer, since ending another investigational device or drug trial or receiving other investigational treatment(s); current or previous enrollment in strictly observational trials is allowed.
42. Previous randomization in this trial.
43. Any condition not covered by any of the other exclusion criteria, which in the investigator's opinion, might jeopardize the participant's safety or compliance with the protocol, or may interfere with the trial objectives.

Statistical analysis methods

<u>Treatment-regimen estimand</u>

- *Treatment:* the randomized treatment taken for up to 48 weeks as an adjunct to a reduced-calorie diet and increased physical activity and including certain anti-hyperglycemia medication (rescue therapy), certain glucose-lowering, anti-hypertensive, and lipid-lowering medication, and obesity management medications.
- *Population:* participants with a BMI ≥30 kg/m^2 or a BMI ≥27 kg/m^2 with one or more obesity complication and presumed or confirmed NASH.
- *Endpoints:* efficacy was evaluated using the co-primary endpoints, the relative reduction in liver fat content of at least 30% assessed by MRI-PDFF from baseline to week 48 (yes/no) and relative change in body weight from baseline to week 48, therefore no adjustments for multiple comparisons were required.
- *Intercurrent events:*
    - For trial participants who initiated a prohibited obesity management medication other than a GLP-1R agonist containing medication during treatment with IMP or after discontinuation from IMP, who initiated a prohibited medication containing GLP-1R agonists, or who discontinued prematurely from IMP for any reason, observed endpoint values through week 48 was used for the analysis.
    - For trial participants who died, a hypothetical strategy was used.
- *Population-level summary:*
    - Difference of adjusted means were compared between the survodutide group and the placebo group for relative change in body weight. The null hypothesis of the difference in adjusted means (survodutide versus placebo) being greater than or equal to zero was tested.
    - Odds ratios were estimated to compare the proportion of participants in the survodutide group versus the placebo group with a relative reduction in liver fat content of at least 30% (yes/no). The null hypothesis of the odds ratio (survodutide/placebo) being less than or equal to one between survodutide and placebo group was tested.

<u>Efficacy estimand</u>

- *Treatment:* the randomized treatment taken for up to 48 weeks as an adjunct to a reduced-calorie diet and increased physical activity, but excluding certain anti-hyperglycemia medication (rescue therapy), certain glucose-lowering, anti-hypertensive, and lipid-lowering medication, and obesity management medications.
- *Population:* participants with a BMI ≥30 kg/m^2 or a BMI ≥27 kg/m^2 with one or more obesity complication and presumed or confirmed NASH.
- *Endpoints:* efficacy was evaluated using the co-primary endpoints, the relative reduction in liver fat content of at least 30% assessed by MRI-PDFF from baseline to week 48 (yes/no) and relative change in body weight from baseline to week 48.
- *Intercurrent events:*
    - For trial participants who initiated an obesity management medication other than GLP-1R agonists containing medication during treatment with IMP or after discontinuation from IMP, all body weight measurements during intake of the obesity management medications were excluded from the analysis. However, the endpoint measurement at week 48 was used in the analysis if the trial participant continued randomized treatment through week 48 and

stopped intake of any obesity management medications other than GLP-1R agonist containing medication before week 48.

  - For trial participants who discontinued prematurely from IMP for any reason, all body weight measurements and MRI-PDFF liver fat data after discontinuation +28 days (residual effect period of survodutide) were excluded from the analysis. That is, a hypothetical strategy was used to estimate what the treatment effect was at week 48 if trial participants had continued their randomized treatment through week 48.
  - For trial participants who initiated prohibited medication containing GLP-1R agonists, all body weight measurements and MRI-PDFF liver fat data after start of medication containing GLP-1R agonists were excluded from the analysis. That is, a hypothetical strategy was used to estimate what the treatment effect was at week 48 if trial participants had not started the prohibited medication containing GLP-1R agonists.
  - For trial participants who died, a hypothetical strategy was used.
- *Population-level summary:*
  - Difference of adjusted means between the survodutide group and the placebo group for relative change in body weight. The null hypothesis of the difference in adjusted means (survodutide versus placebo) being greater than or equal to zero was tested.
  - Odds ratios were estimated to compare the proportion of participants in the survodutide group versus the placebo group with a relative reduction in liver fat content of at least 30% (yes/no). The null hypothesis of the odds ratio (survodutide/placebo) being less than or equal to one between survodutide and placebo group was tested.

Analysis relative to the treatment-regimen estimand

Analysis of covariance (ANCOVA) after multiple imputation of missing endpoint data guided by Rubin[1] was performed for the continuous primary endpoint ‘relative (%) change in body weight from baseline to week 48’. If preceded by the intercurrent event ‘treatment discontinuation’, missing body weight data at week 48 were imputed based on ‘retrieved dropouts’, defined as participants in the same treatment arm, who had their value measured at week 48 but who prematurely discontinued the study drug. For each of the imputed datasets, ANCOVA was applied to the relative change in body weight from baseline to week 48 as response variable and included treatment and T2D status at baseline as fixed effects, and baseline body weight as a continuous covariate. Model estimates were combined over imputed datasets using Rubin’s rules.

A logistic regression with fixed categorical effects of treatment and T2D status at baseline and baseline liver fat content as continuous covariate was used to analyze the occurrence of ‘relative reduction in liver fat content of at least 30% from baseline to week 48 assessed by MRI-PDFF (yes/no)’. The responder/nonresponder (yes/no) status was based on the continuous liver fat measurements, or, in the case of missing liver fat values, on the continuous values imputed with the methods described in the previous paragraph, in combination with the predictive mean matching (PMM) method to avoid imputation of out-of-range values. Model estimates were combined over imputation datasets guided by Rubin’s rules and the odds ratio was reported.[1]

1. Rubin DB. Multiple Imputation for Nonresponse in Surveys. New York: John Wiley & Sons Inc.; 1987.

For each of the two endpoints, if the proportion of off-treatment data collected at week 48 was below 40% for any of the treatment arms, then the 'follow the reference' strategy (that is, after the intercurrent event 'treatment discontinuation', trial participants in the survodutide arm were assumed to follow a trajectory which gradually approached that of the placebo arm while preserving information of any on-treatment and off-treatment data) was instead adopted as the primary analysis (main estimation) and the retrieved dropout analysis was used as sensitivity analysis.

Table 1 │ Conclusion on main imputation method – liver fat content assessed by MRI-PDFF

	Survodutide 6.0 mg (*N* = 146)	Placebo (*N* = 70)	Conclusion for main analysis
No baseline value	0 (0.0)	0 (0.0)	
Premature treatment discontinuation	60 (100%)	28 (100%)	
Week 48 value available (retrieved dropouts)	30 (50.0%)	11 (39.3%)	Follow the reference
No week 48 value available	30 (50.0%)	17 (60.7%)	
No premature treatment discontinuation	86 (100%)	42 (100%)	
Week 48 value available (completers)	85 (98.8%)	42 (100%)	
No week 48 value available	1 (1.2%)	0 (0.0)	

MRI-PDFF, magnetic resonance imaging-proton density fat fraction.

Table 2 │ Conclusion on main imputation method – weight

	Survodutide 6.0 mg (*N* = 146)	Placebo (*N* = 70)	Conclusion for main analysis
No baseline value	0 (0.0)	0 (0.0)	
Premature treatment discontinuation	60 (100%)	28 (100%)	
Week 48 value available (retrieved dropouts)	33 (55.0%)	11 (39.3%)	Follow the reference
No week 48 value available	27 (45.0%)	17 (60.7%)	
No premature treatment discontinuation	86 (100%)	42 (100%)	
Week 48 value available (completers)	86 (100%)	42 (100%)	
No week 48 value available	0 (0.0)	0 (0.0)	

Analysis relative to the efficacy estimand

For the continuous primary endpoint 'relative (%) change in body weight from baseline to week 48', missing values were handled using an MMRM under the MAR (missing at random) assumption. The MMRM used fixed categorical effects for treatment each week and presence of T2D (yes/no), and the fixed continuous (linear) effect of baseline body weight (kg) each week.

The Kenward-Roger approximation was used to estimate denominator degrees of freedom and adjust standard errors. Significance tests were based on contrasts at the primary endpoint time point (week 48) using a two-sided α = 0.05 (two-sided 95% confidence intervals). Unstructured covariance was assumed to model the relationship between pairs of measurements taken at different weeks on the same patient.

A logistic regression with fixed categorical effects T2D status, treatment and the fixed continuous effect of baseline liver fat content assessed by MRI-PDFF were used to analyze the occurrence of 'relative reduction in liver fat content of at least 30% assessed by MRI-PDFF (yes/no)'. The responder/nonresponder (yes/no) status was based on the continuous liver fat measurements. Missing values of the continuous liver fat content (MRI-PDFF) variable were multiply imputed using a MAR assumption.

Clinical Trial Protocol

	Document Number:	c43133006-01
**EU Clinical Trial No.**<br>**Universal Trial Number**	2023-505303-23-00<br>U1111-1299-9925	
**BI Trial No.**	1404-0056	
**BI Investigational Medicinal Product**	Survodutide (BI 456906)	
**Title**	Multicenter, randomised, double-blind, placebo-controlled, 48-week, Phase III trial to evaluate the efficacy and safety of survodutide administered subcutaneously in participants with overweight or obesity and presumed or confirmed non-alcoholic steatohepatitis (NASH)	
**Lay Title**	A study to test whether survodutide helps people living with obesity or overweight and with a confirmed or presumed liver disease called non-alcoholic steatohepatitis (NASH) to reduce liver fat and to lose weight	
**Clinical Phase**	III	
**Clinical Trial Leader**	[redacted]<br>Telephone: [redacted]	
**Coordinating Investigator**	[redacted]<br>Telephone: [redacted]	
**Current Version and Date**	Version 1.0, 01 Dec 2023	
**Original Protocol Date**	01 Dec 2023	Page 1 of **116**

Proprietary confidential information.
© 2023 Boehringer Ingelheim International GmbH or one or more of its affiliated companies. All rights reserved.
This document may not - in full or in part - be passed on, reproduced, published or otherwise used without prior written permission.

Proprietary confidential information © 2023 Boehringer Ingelheim International GmbH or one or more of its affiliated companies

CLINICAL TRIAL PROTOCOL SYNOPSIS

**Company name**	Boehringer Ingelheim
**Original protocol date**<br>**Latest revision date**	01 Dec 2023
**Boehringer Ingelheim trial number**<br>**EU CT number**<br>**Universal Trial Number**	1404-0056<br>2023-505303-23-00<br>U1111-1299-9925
**Title of trial**	Multicentre, randomised, double-blind, placebo-controlled, 48-week, Phase III trial to evaluate the efficacy and safety of survodutide administered subcutaneously in participants with overweight or obesity and presumed or confirmed non-alcoholic steatohepatitis (NASH)
**Coordinating Investigator**	[redacted]<br>Telephone: [redacted]
**Trial site(s)**	Multi-centre trial conducted in approximately 4-8 countries
**Clinical phase**	III
**Trial rationale**	Non-alcoholic fatty liver disease (NAFLD) and non-alcoholic steatohepatitis (NASH) are associated with obesity, dyslipidaemia, hypertension, insulin resistance, diabetes mellitus, and chronic kidney disease. Currently, there is no approved pharmacological treatment for NASH at any disease stage, representing a huge unmet medical need. Weight loss is one of the most critical interventions for obesity and NAFLD/NASH, with a dose-response relationship between the magnitude of weight loss and improvement of liver inflammation, ballooning, and resolution of NAFLD or NASH. This trial investigates the effect of survodutide once weekly versus placebo on liver fat and body weight as an adjunct to a reduced-calorie diet and increased physical activity in participants with presumed or confirmed NASH and obesity (BMI ≥30 kg/m^2) or overweight (BMI ≥27 kg/m^2) with one or more weight-related co-morbidities.

Proprietary confidential information © 2023 Boehringer Ingelheim International GmbH or one or more of its affiliated companies

**Benefit-risk assessment and ethical considerations**	Trial participants treated with survodutide are expected to achieve larger body weight reduction and larger associated reduction in liver fat content, compared with those treated with placebo. The risks for participants caused by participation in the trial, including the trial procedures and exposure to the trial drug, are reasonably low and do not outweigh the potential benefits. The inclusion of placebo helps to better determine the difference between survodutide with lifestyle modification vs. lifestyle modification alone. Overall, the potential benefits coupled with an acceptable safety profile and a well-controlled clinical trial environment support the initiation of the trial.
**Trial objectives**	The primary objective is to demonstrate superiority of survodutide vs. placebo in:<br>• Reduction in liver fat content assessed by MRI-PDFF, defined as ≥30% from baseline at Week 48 (odds ratio, survodutide vs. placebo), AND<br>• The difference in adjusted means of relative change (%) in body weight from baseline to Week 48 (survodutide vs. placebo).<br>Secondary (exploratory) objectives are to investigate the superiority of survodutide vs. placebo in terms of the difference in adjusted means of changes from baseline to Week 48 in liver fat content assessed by MRI-PDFF, iron corrected T1 mapping (cT1), alanine aminotransferase (ALT) levels, waist circumference, Homeostasis Model Assessment - Insulin Resistance (HOMA-IR), liver stiffness assessed by magnetic resonance elastography (MRE), and liver volume assessed by MRI.<br>All treatment comparisons will be as randomised, including the effects of any early treatment discontinuation or any prohibited (anti-obesity) treatment including the effects of anti-obesity treatments, including GLP-1R agonists. In addition, treatments will be compared as randomised, but excluding effects of any early treatment discontinuation and any prohibited (anti-obesity) treatments.
**Trial endpoints**	Co-primary endpoints:<br>• Relative reduction in liver fat content of at least 30% from baseline to Week 48 (yes/no) assessed by MRI-PDFF, AND<br>• Relative change (%) in body weight from baseline to Week 48<br><br>Secondary endpoints:<br>• Absolute and relative change (%) from baseline to Week 48 in liver fat content assessed by MRI-PDFF<br>• Reduction from baseline to Week 48 in cT1 levels of ≥80 ms (yes/no)<br>• Absolute and relative change from baseline to Week 48 in ALT levels

Proprietary confidential information © 2023 Boehringer Ingelheim International GmbH or one or more of its affiliated companies

	• Absolute and relative change from baseline to Week 48 in waist circumference [cm]<br>• Absolute change from baseline to Week 48 in HOMA-IR (FPI [mlU/L]·FPG [mmol/L]/22.5)<br>• Absolute and relative change from baseline to Week 48 in liver stiffness assessed by MRE<br>• Absolute and relative change in liver volume [mL] from baseline to Week 48 measured using MRI
**Trial design**	Randomised, double-blind, placebo-controlled, 48-week, Phase III clinical trial
**Total number of trial participants randomised**	Approximately 160
**Number of trial participants per treatment group**	Survodutide: approximately 106 participants<br>Placebo: approximately 53 participants
**Diagnosis, main inclusion and exclusion criteria**	Main diagnosis: presumed or confirmed NASH and overweight or obesity<br><br>Key inclusion criteria:<br>• Male or female, age ≥18 years at the time of signing informed consent, and at least the legal age of consent in countries where it is >18 years<br>• BMI ≥30 kg/m^2, OR BMI ≥27 kg/m^2 and at least one of the following weight-related comorbidities at screening:<br>o Hypertension (defined as repeated, i.e. at least 3 measurements in resting condition, SBP values of ≥140 mmHg and/or DBP values of ≥90 mmHg in the absence of anti-hypertensive treatment, or intake of at least 1 anti-hypertensive drug to maintain a normotensive blood pressure)<br>o Dyslipidaemia (defined as at least 1 lipid-lowering treatment required to maintain normal blood lipid levels, or low-density lipoprotein [LDL] ≥160 mg/dL [≥4.1 mmol/L] or triglycerides ≥150 mg/dL [≥1.7 mmol/L], or high-density lipoprotein (HDL] <40 mg/dL (<1.0 mmol/L] for men or HDL<50 mg/dL (<1.3 mmol/L) for women<br>o Obstructive sleep apnoea<br>o Cardiovascular disease (e.g. heart failure with New York Heart Association [NYHA] functional class II-III, history of ischaemic or haemorrhagic stroke or cerebrovascular revascularisation procedure [e.g. carotid endarterectomy and/or stent], MI, coronary artery disease, or peripheral vascular disease)

Proprietary confidential information © 2023 Boehringer Ingelheim International GmbH or one or more of its affiliated companies

	o T2DM (diagnosed at least 180 days prior to screening, with glycated haemoglobin [HbA_{1c}] ≥6.5% [48 mmol/mol] and <10% [86 mmol/mol] as measured by the central laboratory at screening)<br>• Presumed/confirmed NASH: Evidence of hepatic steatosis (defined by an MRI-PDFF ≥8% at screening) with the exclusion of secondary causes of hepatic fat accumulation, such as significant alcohol consumption, other chronic liver diseases and/or steatogenic medications AND at least one of the following:<br>o MRE ≥2.61 and <4.68 kPa at screening, OR<br>o MAST score ≥0.242 at screening, OR<br>o FAST score ≥0.5 at screening, OR<br>o FibroScan® Vibration-Controlled Transient Elastography (VCTE™) ≥8 kPa and <20 kPa at screening, OR<br>o FIB-4 score ≥2.67 and <3.48 at screening, OR<br>o ELF score >7.7 and <11.3 at screening, OR<br>o cT1 ≥875 ms at screening, OR<br>o Recent liver biopsy (within 3 years of screening) consistent with NASH (defined as the presence of steatosis, inflammation, and ballooning) with stage ≤3 fibrosis according to the NASH Clinical Research Network classification<br><br>Key exclusion criteria:<br>• Current or history of significant alcohol consumption (defined as intake of >210 g/week in men and >140 g/week in women on average over a consecutive period of more than 3 months) or inability to reliably quantify alcohol consumption based on the investigator's judgement within the last 5 years.<br>• Intake of medications associated with liver injury, hepatic steatosis or steatohepatitis.<br>• History of other chronic liver diseases (e.g. viral hepatitis, autoimmune liver disease, primary biliary cholangitis , primary sclerosing cholangitis, Wilson's disease, hemochromatosis, A1At deficiency, history of liver transplantation). Hepatitis B and C testing will be done at Visit 1. Participants with positive HBsAg should be excluded. Participants treated for hepatitis C must have a negative RNA test at screening and also be HCV RNA negative for at least 3 years prior to screening in order to be eligible for the trial.<br>• Cirrhosis based on clinical assessment, abdominal imaging, liver histology or non-invasive tests assessed at screening (ELF ≥11.3 or FIB-4 ≥3.48 or FibroScan® VCTE™ ≥20 kPa or MRE ≥4.68 kPa)<br>• Current decompensated liver disease or previous hepatic decompensation (ascites, spontaneous bacterial peritonitis, portal

Proprietary confidential information © 2023 Boehringer Ingelheim International GmbH or one or more of its affiliated companies

	hypertension bleeding, hepatic encephalopathy, hepatorenal syndrome).<br>• Evidence of portal hypertension (e.g. splenomegaly, oesophageal varices, or other portosystemic collateral pathways).<br>• Any of the following liver laboratory test abnormalities at screening:<br>• Serum AST and/or ALT elevation ≥5x ULN<br>• Total serum bilirubin concentration ≥1.2x ULN (except for cases of known Gilbert's Syndrome)<br>• Alkaline phosphatase >2x ULN<br>• INR ≥1.3 (unless patient is on anticoagulants)<br>• Body weight variation (self-reported) >5% within 3 months before screening<br>• Medications for obesity within 3 months before screening<br>• Previous or planned (during the trial period) treatment for obesity with surgery or a weight loss device, or prior surgery of the gastrointestinal (GI) tract that could interfere with body weight. The following are allowed: (1) liposuction and/or abdominoplasty, if performed >1 year before screening, (2) lap banding, if the band has been removed >1 year before screening, (3) intragastric balloon, if the balloon has been removed >1 year before screening, (4) duodenal-jejunal bypass sleeve, if the sleeve has been removed >1 year before screening (5) appendectomy, (6) simple hernia repair, or (7) cholecystectomy.<br>• Obesity induced by other endocrinologic disorders (i.e. Cushing Syndrome) or diagnosed monogenetic or syndromic forms of obesity (i.e. melanocortin 4 receptor deficiency, leptin deficiency, or Prader Willi Syndrome)
**Trial intervention and test product**	Survodutide (BI 456906)
**Dose and mode of administration**	6.0 mg survodutide once weekly per subcutaneous (s.c.) injection
**Comparator product**	Placebo
**Dose and mode of administration**	Matching s.c. injection
**Duration of treatment**	48 weeks
**Statistical methods**	The primary objective will be addressed by the treatment-regimen estimand together with the efficacy estimand. Contrary to the efficacy estimand, the treatment-regimen estimand tolerates certain anti-hyperglycaemia medication (rescue therapy), certain glucose-

Proprietary confidential information © 2023 Boehringer Ingelheim International GmbH or one or more of its affiliated companies

	lowering, anti-hypertensive, and lipid-lowering medication, and possibly anti-obesity medication.<br>Relative to the treatment-regimen estimand:<br>Analysis of covariance with multiple imputation of missing measure and statistical inference over multiple imputation of missing data will be performed for the continuous primary endpoint of "relative (%) change in body weight from baseline to Week 48". Missing body weight data at Week 48 will be imputed based on retrieved dropouts, i.e. participants with Week 48 value in the same treatment arm who prematurely discontinued trial drug. If retrieved dropouts are insufficient to provide a reliable imputation model, multiple imputation will be based on the follow-the-reference strategy (i.e. participants in the survodutide arm who discontinue treatment are assumed to have a similar effect as the placebo arm while preserving information of any on-treatment and off-treatment data. The analysis will include treatment and region and T2DM as fixed effects, and baseline body weight as a covariate.<br>A logistic regression with fixed categorical effects of region, T2DM status, and treatment will be used to analyse the primary endpoint of "Relative reduction in liver fat content of at least 30% from baseline to Week 48 assessed by MRI-PDFF (yes/no)". Missing continuous values of MRI-PDFF liver fat at Week 48 will be imputed using the multiple imputation method from Week 28 as described above and then dichotomized. If Week 28 is missing, the participant will be imputed as non-responder.<br>Relative to the efficacy estimand:<br>MMRM with treatment, visit, treatment by visit interaction, region, T2DM status, and region by visit interaction, as well as baseline by visit interaction as fixed effects, and baseline as a covariate will be used for analysis of the continuous primary endpoint.<br>A logistic regression with fixed categorical effects of region, T2DM status, treatment, and the fixed continuous effect of baseline will be used to analyse the occurrence of "Relative reduction in liver fat content of at least 30% from baseline to Week 48 assessed by MRI-PDFF (yes/no)". Missing values will be imputed using the imputed values from MMRM analysis and then dichotomized.

Proprietary confidential information © 2023 Boehringer Ingelheim International GmbH or one or more of its affiliated companies

SCHEDULE OF ASSESSMENTS

			**Dose escalation**								**Maintenance**					**FU**
	**Screening** (in-clinic)	**Randomisation** (in-clinic)	Remote	In-clinic	Remote	In-clinic	In-clinic	In-clinic	In-clinic	In-clinic	In-clinic	Remote	In-clinic	In-clinic	In-clinic (EOT)[10]	In-clinic[11]
**Visit**[1]	**V1**	**V2**	**V3**	**V4**	**V5**	**V6**	**V7**	**V8**	**V9**	**V10**	**V11**	**V12**	**V13**	**V14**	**V15**	**FU/ EOS**
**Timing of visits (weeks)**	Up to -8	0	2	4	6	8	12	16	20	24	28	32	36	42	48	51
**Timing of visits (days)**	Up to -56	1	15	29	43	57	85	113	141	169	197	225	253	295	337	358
**Visit window (days)**		+/-0	+/-3	+/-3	+/-3	+/-3	+/-3	+/-3	+/-3	+/-3	+/-7	+/-7	+/-7	+/-7	+/-7	+7
Informed consent[2]	X															
Review of inclusion/exclusion criteria	X	X														
Demographics	X															
Medical and weight history, baseline conditions[3]	X															
Height	X															
Weight[4]	X	X		X		X	X	X	X	X	X		X	X	X	X
Waist and hip circumference		X		X		X	X	X	X	X	X		X	X	X	X
Physical examination[5]	X	X								X					X	
Eye examination[6]	X									X					X	
Vital signs (SBP, DBP, pulse)[7]	X	X		X		X	X	X	X	X	X		X	X	X	X
12-lead ECG[8]	X	X		X		X	X	X	X	X			X		X	X
Concomitant therapy[9]	X	X	X	X	X	X	X	X	X	X	X	X	X	X	X	X
Assessment of obesity staging	X															
Evaluation of lipid lowering treatment[12]		X		X		X	X	X	X	X	X		X	X	X	X
Evaluation of antihypertensive treatment[12]		X		X		X	X	X	X	X	X		X	X	X	X
All AEs/SAEs/AESIs[13] and check criteria for discontinuation	X	X	X	X	X	X	X	X	X	X	X	X	X	X	X	X

Proprietary confidential information © 2023 Boehringer Ingelheim International GmbH or one or more of its affiliated companies

			Dose escalation								Maintenance					FU
	**Screening** (in-clinic)	**Randomisation** (in-clinic)	Remote	In-clinic	Remote	In-clinic	In-clinic	In-clinic	In-clinic	In-clinic	In-clinic	Remote	In-clinic	In-clinic	In-clinic (EOT)[10]	In-clinic[11]
**Visit[1]**	**V1**	**V2**	**V3**	**V4**	**V5**	**V6**	**V7**	**V8**	**V9**	**V10**	**V11**	**V12**	**V13**	**V14**	**V15**	**FU/ EOS**
**Timing of visits (weeks)**	Up to -8	0	2	4	6	8	12	16	20	24	28	32	36	42	48	51
**Timing of visits (days)**	Up to -56	1	15	29	43	57	85	113	141	169	197	225	253	295	337	358
**Visit window (days)**		+/-0	+/-3	+/-3	+/-3	+/-3	+/-3	+/-3	+/-3	+/-3	+/-7	+/-7	+/-7	+/-7	+/-7	+7
Randomisation		X														
**Liver outcome assessments**																
MRI-PDFF[14]	X										X				X	
cT1[14]	X										X				X	
MRE[14]	X										X				X	
FibroScan® VCTE™ + CAP™[15]	X										X				X	
**Laboratory tests[16]**																
Safety laboratory tests[17]	X	X		X		X	X	X	X	X	X		X		X	X
HBV, HCV, HIV	X															
HbA_{1c}	X						X				X		X		X	
FPG, FPI, C-peptide		X					X				X		X		X	
HOMA-IR		X					X				X		X		X	
Lipids		X					X				X		X		X	
Urinalysis[18]		X		X			X				X		X		X	
Sampling for biomarkers[19]		X					X				X				X	
Sampling for biobanking (optional)[2, 20]		X													X	
Sampling for PK, ADA, and NAb[21]		X		X		X	X			X			X		X	X
FSH[22]	X															
Pregnancy testing[23]	X	X		X		X	X	X	X	X	X		X	X	X	X
**Questionnaires[24]**																

[redacted]

Proprietary confidential information © 2023 Boehringer Ingelheim International GmbH or one or more of its affiliated companies

			**Dose escalation**								**Maintenance**					**FU**
	**Screening** (in-clinic)	**Randomisation** (in-clinic)	Remote	In-clinic	Remote	In-clinic	In-clinic	In-clinic	In-clinic	In-clinic	In-clinic	Remote	In-clinic	In-clinic	In-clinic (EOT)[10]	In-clinic[11]
**Visit**[1]	**V1**	**V2**	**V3**	**V4**	**V5**	**V6**	**V7**	**V8**	**V9**	**V10**	**V11**	**V12**	**V13**	**V14**	**V15**	**FU/ EOS**
**Timing of visits (weeks)**	Up to -8	0	2	4	6	8	12	16	20	24	28	32	36	42	48	51
**Timing of visits (days)**	Up to -56	1	15	29	43	57	85	113	141	169	197	225	253	295	337	358
**Visit window (days)**		+/-0	+/-3	+/-3	+/-3	+/-3	+/-3	+/-3	+/-3	+/-3	+/-7	+/-7	+/-7	+/-7	+/-7	+7
[redacted]																
PHQ-9	X	X					X			X			X		X	
C-SSRS	X	X	X	X	X	X	X	X	X	X	X	X	X	X	X	X
**Other assessments and procedures**																
Training in/observe prefilled syringe administration[25]		X	X	X	X			X			X					
Hand out IFU		X														
IRT call and dispense IMP[26]	X[27]	X		X		X	X	X	X	X	X		X			
eDiary review (IMP administration, injection site reactions, and compliance check)[28]			X	X	X	X	X	X	X	X	X	X	X	X	X	
Paper diary or local app review		X	X	X	X	X	X	X	X	X	X	X	X	X	X	
Diet and physical activity counselling[29]		X		X		X	X	X	X	X	X		X	X	X	
Hand out glucose monitoring device (if applicable) and participant materials[30]	X[31]	X														
Hyper-/hypoglycaemic episode review (eDiary), if applicable [32]			X	X	X	X	X	X	X	X	X	X	X	X	X	X
[redacted]																

Proprietary confidential information © 2023 Boehringer Ingelheim International GmbH or one or more of its affiliated companies

			Dose escalation								Maintenance					FU
	**Screening** (in-clinic)	**Randomisation** (in-clinic)	Remote	In-clinic	Remote	In-clinic	In-clinic	In-clinic	In-clinic	In-clinic	In-clinic	Remote	In-clinic	In-clinic	In-clinic (EOT)[10]	In-clinic[11]
**Visit[1]**	**V1**	**V2**	**V3**	**V4**	**V5**	**V6**	**V7**	**V8**	**V9**	**V10**	**V11**	**V12**	**V13**	**V14**	**V15**	**FU/ EOS**
**Timing of visits (weeks)**	Up to -8	0	2	4	6	8	12	16	20	24	28	32	36	42	48	51
**Timing of visits (days)**	Up to -56	1	15	29	43	57	85	113	141	169	197	225	253	295	337	358
**Visit window (days)**		+/-0	+/-3	+/-3	+/-3	+/-3	+/-3	+/-3	+/-3	+/-3	+/-7	+/-7	+/-7	+/-7	+/-7	+7
Vital status																X

1. If the IMP administration is scheduled on the day of the visit, the trial participant has to be informed to come to the study site prior to the IMP administration so that the PK, ADA, and NAb sample is obtained prior to IMP administration.
2. A separate informed consent is required for biobanking samples.
3. Medical history includes assessment of pre-existing conditions (special attention to be paid on medical conditions requested by in-/exclusion criteria and other important medical conditions, e.g. history of gallbladder disease, cardiovascular disease, history of psychiatric disorder, MTC, and history of any cancer other than MTC) and substance usage (such as alcohol). In addition, if a mammogram or colonoscopy was performed within 2 years prior to screening as part of routine clinical practice, data should be recorded in the eCRF.
4. Body weight must be measured in fasting state (except the screening visit). If the trial participant is not fasted, the participant should be called in for a new visit within the visit window. See Section 5.1.3.1.
5. The physical examination should be repeated if the baseline visit (Visit 2) is performed more than 2 weeks after the screening visit.
6. The eye examination has to be performed in the period between screening and randomization, if not performed in the past 3 months before screening, to verify uncontrolled and potentially unstable diabetic retinopathy or maculopathy (Section 5.2.5.2). The eye examination at Visit 10 can be performed within a time window of +/- 1 month of the visit, and at the EOT Visit within a time window of -2 months of the visit. Unless the eye examination at Visit 10 was performed within 3 months before the EOT Visit, the EOT eye examination must be performed on the EOT Visit with a time window of -2 months of the visit.
7. Vital sign measurements should be taken before obtaining an ECG tracing and before collection of blood samples for laboratory testing per the instructions in Section 5.2.2.
8. ECG at screening will be done in triplicate. ECGs should be collected prior to collection of blood samples for laboratory testing, including PK samples. For the schedule of PK sampling see Section 10.3.
9. Special attention has to be paid if medication with a narrow therapeutic index is used. Refer to Section 4.2.2.1.
10. If a trial participant has been prematurely discontinued from IMP, the EOT visit will be scheduled 7 days after the last IMP dose or if more than 7 days, then as soon as possible.

Proprietary confidential information © 2023 Boehringer Ingelheim International GmbH or one or more of its affiliated companies

11. The last regular visit is the FU/ EOS Visit. Trial participants who discontinue the IMP prematurely should undergo the EOT Visit 7 days after the last IMP dose or if more than 7 days then as soon as possible and the FU Visit 21 days after the EOT Visit and at least 28 days after the last IMP dose. For trial participants who discontinue the IMP prematurely, the FU Visit does not need to be the EOS Visit if they follow the visit schedule. The FU/EOS Visit should be in-clinic, and a remote visit (if allowed per local regulations) should be done only as an exemption. Trial participants who discontinue the IMP prematurely should still follow the trial schedule. If possible, body weight, AEs, and concomitant therapy changes should be recorded.
12. Changes of the medication including dose in anti-hypertension and lipid-lowering medications have to be assessed for increase/decrease/no change and captured in the eCRF.
13. After the EOS Visit (i.e. the individual trial participant's EOS), the investigator should only report any occurrence of cancer of new histology, IMP related SAEs, and IMP related AESIs which the investigator may become aware of. Refer to Section 5.2.6.2.2.
14. MRI to be scheduled prior to Visit 2 (randomisation visit) only for trial participants who have met all inclusion criteria and none of the exclusion criteria. Refer to Section 6.2.1. The MRI assessment at EOT may be scheduled on a different day than the EOT day; however, it must still fall within the +/-7-day time window.
15. VCTE™ to be performed only if CAP™ is not available
16. All in-clinic visits should be on fasting state (except for screening). Trial participants should be instructed to come after at least 8 h without food or liquid intake, except water, and without administering anti-hyperglycaemia medications (as applicable), which will be taken in the clinic. The IMP dose administration (if scheduled within the day of the visit) should be withheld in the morning of the clinic visit until blood sampling has been performed. If a lab parameter measured at screening in non-fasting state is out of normal range and thus prevents the participant from inclusion into the trial as per eligibility criteria, measurement can be repeated in fasting state within the screening period window.
17. All laboratory assessments will be performed by a central laboratory. Instruction regarding sample collection, sample handling/processing, and sample shipments will be provided in the respective laboratory manuals.
18. Urine sample should be collected in the clinic from the second morning void (preferably) and if not possible, from spot urine. Refer to Section 5.2.3 for details.
19. Refer to Section 5.4.1 for details [redacted]
20. DNA sample for biobanking will only be collected at Visit 2 while plasma/serum samples for biobanking will be collected at Visit 2 and at the Week 48/EOT visit. Collection of DNA samples at Visit 2 is preferred but also possible at later visits.
21. If the IMP administration is scheduled during the day of the visit, then the trial participant will come to the study site prior to IMP administration and sampling for PK, ADA, and NAb will be done prior to IMP administration. IMP administration prior to PK sampling should be documented in trial participant's eDiary. Refer to Section 10.3 for the detailed sampling schedule. If a participant has discontinued the IMP prematurely, sampling is performed for ADA and NAb testing, but not for PK testing.
22. For women age >40 years with amenorrhea for 12 months, to confirm if they are menopausal.
23. For WOCBP only. A urine pregnancy test will be done during screening. If it is positive, the participant will be considered a screen failure. If it is negative, a serum pregnancy test will be done. Urine pregnancy tests will be performed at all other in-clinic visits. If the urine pregnancy test is positive, IMP should be paused, and a serum pregnancy test will be done. If the serum pregnancy test is positive, the participant needs to be discontinued from IMP treatment. Pregnancy tests can be done more frequently if required by local regulations. If a female participant discontinues the IMP prematurely, no further pregnancy testing is required.

Proprietary confidential information © 2023 Boehringer Ingelheim International GmbH or one or more of its affiliated companies

24. Questionnaires should be performed prior to any other assessment scheduled for the in-clinic visit and in the following order: [redacted] 5) PHQ-9, 6) C-SSRS. If PHQ-9 score is ≥15 and/or type 4 or 5 of suicidal ideation in C-SSRS, the trial participant needs to be referred to a mental health professional (MHP) visit. If the trial participant refuses to be referred to an MHP, this needs to be documented in the medical records and the investigator has to assess, whether it is safe for the participant to continue the trial or if the participant should be discontinued.
25. Each trial participant will receive training in the administration of the prefilled syringes. Site staff will observe the trial participant administering the prefilled syringe in the clinic at Visit 2. The remote visits (if allowed per local regulations) are to be performed as video phone calls (refer to Section 6.2). IMP administration may be observed by site staff during Visits 3, 4, 5, 8 and 11. Site staff and the trial participant might plan to schedule Visits 3, 4, 5, 8 and 11 on the day of planned IMP administration to allow site staff to observe the injection at these visits if needed.
26. Trial participants will receive enough IMP to cover the time period until the next scheduled visit. The investigator should instruct the trial participants about the IMP storage temperature requirements. Trial participants should be instructed about the necessity to return all IMP related packaging used and unused. Refer to Section 4.1.4.
27. At the screening visit, no IMP will be dispensed.
28. Refer to Section 4.1.4 for details.
29. The counselling on diet and physical activity should be performed by a dietician or equivalent qualified delegate. It should include the calculation of the individualized energy requirement, total energy expenditure (TEE), and methods to change the dietary composition and amount of physical activity. The counselling on diet and physical activity may be performed on a separate day from the rest of the visit's trial procedures but must occur within the visit window. The study personnel should provide reinforcement and encouragement for lifestyle modifications. The counselling includes the review of diet and exercise goals. Refer to Section 4.2.1.2 for details.
30. Trial participant materials include: 1) Instructions and eDiary to record IMP dosing information, 2) Trial participant ID card, 3) Instructions and trial participant brochure for control of sickness, and 4) Dietary materials and a paper diary to record body weight measurements, physical activity, and dietary compliance. In addition, for trial participants with T2DM or who develop T2DM during the trial, the blood glucose monitoring device and IFU will be provided.
31. At screening, only the paper diary will be given to the trial participants.
32. Participants with T2DM or who develop T2DM must record any hypo- or hyperglycaemic episodes in the eDiary which then must regularly be reviewed. Weekly testing is recommended. Additional measurements should be taken in case of hypo- or hyperglycaemia-related symptoms.

[redacted]

Proprietary confidential information © 2023 Boehringer Ingelheim International GmbH or one or more of its affiliated companies

TABLE OF CONTENTS

TITLE PAGE 1
CLINICAL TRIAL PROTOCOL SYNOPSIS 2
SCHEDULE OF ASSESSMENTS 8
TABLE OF CONTENTS 14
ABBREVIATIONS AND DEFINITIONS 18
1. INTRODUCTION 20
1.1 MEDICAL BACKGROUND 20
1.2 DRUG PROFILE 21
1.3 RATIONALE FOR PERFORMING THE TRIAL 24
1.4 BENEFIT - RISK ASSESSMENT 25
1.4.1 Benefits 25
1.4.2 Risks 25
1.4.3 Discussion 29
2. TRIAL OBJECTIVES AND ENDPOINTS 30
2.1 MAIN OBJECTIVES, PRIMARY AND SECONDARY ENDPOINTS 30
2.1.1 Main objectives 30
2.1.2 Primary endpoints 30
2.1.3 Secondary endpoints 30
2.2 FURTHER OBJECTIVES AND FURTHER ENDPOINTS 31
2.2.1 Further objectives 31
2.2.2 Further endpoints 31
3. DESCRIPTION OF DESIGN AND TRIAL POPULATION 34
3.1 OVERALL TRIAL DESIGN 34
3.2 DISCUSSION OF TRIAL DESIGN, INCLUDING THE CHOICE OF CONTROL GROUP(S) 35
3.3 SELECTION OF TRIAL POPULATION 35
3.3.1 Main diagnosis for trial entry 35
3.3.2 Inclusion criteria 35
3.3.3 Exclusion criteria 37
3.3.4 Discontinuation of trial participants from treatment or assessments 40
3.3.4.1 Discontinuation of trial treatment 40
3.3.4.2 Withdrawal of consent to trial participation 42
3.3.4.3 Discontinuation of the trial by the sponsor 43
3.3.4.4 Lost to follow-up 43
4. TREATMENTS 45
4.1 INVESTIGATIONAL TREATMENTS 45
4.1.1 Identity of the Investigational Medicinal Products 45
4.1.2 Selection of doses in the trial and dose modifications 45
4.1.3 Method of assigning trial participants to treatment groups 46
4.1.4 Drug assignment and administration of doses for each trial participant 46

Proprietary confidential information © 2023 Boehringer Ingelheim International GmbH or one or more of its affiliated companies

4.1.4.1 Management of gastrointestinal symptoms 49
4.1.5 Blinding and procedures for unblinding 49
4.1.5.1 Blinding 49
4.1.5.2 Emergency unblinding and breaking the code 50
4.1.6 Packaging, labelling, and re-supply 50
4.1.7 Storage conditions 50
4.1.8 Drug accountability 51
4.2 OTHER TREATMENTS, EMERGENCY PROCEDURES, RESTRICTIONS 52
4.2.1 Other treatments and emergency procedures 52
4.2.1.1 Concomitant medications 52
4.2.1.1.1 Home glucose monitoring 52
4.2.1.1.2 Rescue medication for treatment of hyperglycaemia 52
4.2.1.1.3 Hypoglycaemic episodes 53
4.2.1.2 Dietary and physical activity counselling 53
4.2.1.3 Severe and/or systemic hypersensitivity including injection reaction and anaphylactic reaction 54
4.2.1.4 Elective surgery/procedures that require general anaesthesia or deep sedation 55
4.2.2 Restrictions 55
4.2.2.1 Restrictions regarding concomitant treatment 55
4.2.2.2 Restrictions on diet and lifestyle 57
4.2.2.3 Contraception requirements 57
4.3 TREATMENT COMPLIANCE 58
5. ASSESSMENTS 59
5.1 ASSESSMENT OF EFFICACY 59
5.1.1 MRI 59
5.1.2 Fibroscan® 59
5.1.3 Body measurements 59
5.1.3.1 Weight measurement 59
5.1.3.2 Height 60
5.1.3.3 Body mass index 60
5.1.3.4 Waist and hip circumference measurement 60
5.1.4 Blood pressure measurements 60
[redacted]
5.1.6 Efficacy laboratory parameters 61
5.2 ASSESSMENT OF SAFETY 61
5.2.1 Physical examination 61
5.2.2 Vital signs 62
5.2.3 Safety laboratory parameters 62
5.2.4 Electrocardiogram 64
5.2.5 Other safety parameters 65
5.2.5.1 Mental health questionnaires 65
5.2.5.2 Eye examination for participants with T2DM 66
5.2.6 Assessment of adverse events 67
5.2.6.1 Definitions of AEs 67
5.2.6.1.1 Adverse event 67
5.2.6.1.2 Serious adverse event 67

Proprietary confidential information © 2023 Boehringer Ingelheim International GmbH or one or more of its affiliated companies

5.2.6.1.3 AEs considered "Always Serious" 68
5.2.6.1.4 Adverse events of special interest 68
5.2.6.1.5 Intensity (severity) of AEs 71
5.2.6.1.6 Causal relationship of AEs 71
5.2.6.1.7 Adverse events requiring additional data collection 72
5.2.6.2 Adverse event collection and reporting 74
5.2.6.2.1 AE Collection 74
5.2.6.2.2 AE reporting to the sponsor and timelines 75
5.2.6.2.3 Pregnancy 75
5.2.6.2.4 Other safety topics 75
5.3 DRUG CONCENTRATION MEASUREMENTS AND PHARMACOKINETICS 76
5.3.1 Assessment of pharmacokinetics 76
5.3.2 Methods of sample collection 76
[redacted]
5.3.4 Pharmacokinetic - pharmacodynamic relationship 76
[redacted]
5.5 BIOBANKING 78
5.5.1 Methods and timing of sample collection 78
5.6 OTHER ASSESSMENTS 78
5.6.1 Immunogenicity 78
5.6.1.1 Timing of immunogenicity measures 78
5.6.1.2 Methods of sample collection 79
5.6.1.3 Immunogenicity analysis 79
5.6.2 Clinical staging of obesity 79
[redacted]
5.7 APPROPRIATENESS OF MEASUREMENTS 80
6. INVESTIGATIONAL PLAN 81
6.1 VISIT SCHEDULE 81
6.2 DETAILS OF TRIAL PROCEDURES AT SELECTED VISITS 81
6.2.1 Screening period 82
6.2.2 Treatment period 86
6.2.3 Follow-up period and trial completion 88
6.2.4 Exceptional conditions 88
7. STATISTICAL METHODS AND DETERMINATION OF SAMPLE SIZE 90
7.1 NULL AND ALTERNATIVE HYPOTHESES 90
7.2 PLANNED ANALYSES 90
7.2.1 General considerations 90
7.2.2 Handling of Intercurrent Events 91
7.2.3 Primary objective analyses 93
7.2.3.1 Main analyses 94
7.2.3.2 Sensitivity Analyses 95
[redacted]

Proprietary confidential information © 2023 Boehringer Ingelheim International GmbH or one or more of its affiliated companies

7.2.3.4 Supplementary Analyses 95
7.2.4 Secondary objective analyses 95
7.2.5 Further objective analyses 95
7.2.6 Safety analyses 95
7.2.7 Other Analyses 96
[redacted]
7.3 HANDLING OF MISSING DATA 96
7.4 RANDOMISATION 96
7.5 DETERMINATION OF SAMPLE SIZE 97
8. INFORMED CONSENT, TRIAL RECORDS, DATA PROTECTION, PUBLICATION POLICY, AND ADMINISTRATIVE STRUCTURE 99
8.1 TRIAL APPROVAL, PATIENT INFORMATION, INFORMED CONSENT 99
8.2 DATA QUALITY ASSURANCE 100
8.3 RECORDS 100
8.3.1 Source documents 100
8.3.2 Direct access to source data and documents 101
8.3.3 Storage period of records 101
8.4 EXPEDITED REPORTING OF ADVERSE EVENTS 102
8.5 STATEMENT OF CONFIDENTIALITY AND TRIAL PARTICIPANT PRIVACY 102
8.5.1 Collection, storage, and future use of biological samples and corresponding data 102
8.6 TRIAL MILESTONES 103
8.7 ADMINISTRATIVE STRUCTURE OF THE TRIAL 103
9. REFERENCES 105
9.1 PUBLISHED REFERENCES 105
[redacted]
10. APPENDICES 110
10.1 MANAGEMENT OF GASTROINTESTINAL SYMPTOMS 110
10.2 CONTRACEPTION METHODS ALLOWED IN THIS TRIAL 113
10.3 TIME SCHEDULE FOR PHARMACOKINETIC (PK) AND ANTI-DRUG ANTIBODY (ADA) / NEUTRALISING ANTIBODY (NAB) BLOOD SAMPLING 114
10.4 CLINICAL STAGING OF OBESITY 115
11. DESCRIPTION OF GLOBAL AMENDMENT(S) 116

Proprietary confidential information © 2023 Boehringer Ingelheim International GmbH or one or more of its affiliated companies

ABBREVIATIONS AND DEFINITIONS

A1At	Alpha-1 Antitrypsin
ADA	Anti-Drug Antibodies
AE	Adverse Event
AESI	Adverse Event of Special Interest
ALT	Alanine amino transferase
ANCOVA	Analysis of Covariance
ApoB100	Apolipoprotein B100
[redacted]	
AST	Aspartate amino transferase
AUC	Area under the Curve
BMI	Body Mass Index
CAP™	Controlled Attenuation Parameter
CEC	Clinical Event Committee
CI	Confidence interval
CK	Creatine kinase
CKD-EPIcr	Chronic Kidney Disease Epidemiology Collaboration creatinine equation
[redacted]	
CRF	Case Report Form
CRO	Contract Research Organisation
C-SSRS	Columbia-Suicide Severity Rating Scale
CTCAE	Common Terminology Criteria for Adverse Events
cT1	Iron corrected T1
[redacted]	
CV	Cardiovascular
DBP	Diastolic Blood Pressure
DILI	Drug induced liver injury
DMC	Data Monitoring Committee
DPP-4i	Dipeptidyl peptidase 4 inhibitor
ECG	Electrocardiogram
eCOA	Electronic Clinical Outcome Assessment
eCRF	Electronic case report form
EDC	Electronic Data Capture/Collection
eGFR	Estimated glomerular filtration rate
ELF	Enhanced liver fibrosis
EOS	End of Study
EOT	End of Treatment
EQ-5D	EuroQoL-5 Dimensions
FAST	FibroScan-AST
FDA	Food and Drug Administration
FIB-4	Fibrosis-4
[redacted]	
FPG	Fasting Plasma Glucose
FPI	Fasting Plasma Insulin
FU	Follow-up
GCG	Glucagon
GCGR	Glucagon receptor
GCP	Good Clinical Practice
GGT	Gamma glutamyltransferase
GI	Gastrointestinal
GIP	Glucose-dependent insulinotropic polypeptide
GLP-1R	Glucagon-like-peptide (GLP) 1 receptor
[redacted]	
HbA_{1c}	Glycosylated haemoglobin A1c
hCG	Human chorionic gonadotropin
HCV	Hepatitis C virus

Proprietary confidential information © 2023 Boehringer Ingelheim International GmbH or one or more of its affiliated companies

HDL	High-density lipoprotein
HEOR	Health Economics and Outcomes Research
HF	Heart Failure
HIV	Human Immunodeficiency Virus
HOMA-IR	Homeostasis Model Assessment -Insulin Resistance
HRQoL	Health-related quality of life
[redacted]	
hsCRP	High sensitivity C-reactive protein
ICE	Intercurrent Event
ICH	International Conference of Harmonisation
IEC	Independent Ethics Committee
IFU	Instructions for use
IMP	Investigational Medicinal Product
INR	International Normalized Ratio
IRB	Institutional Review Board
ISF	Investigator Site File
[redacted]	
LDL	Low-density lipoprotein
LDL-C	Low-density lipoprotein C
MASLD	Metabolic dysfunction-associated steatotic liver disease
MASH	Metabolic dysfunction-associated steatohepatitis
MAST	MRI-AST
MedDRA	Medical Dictionary for Regulatory Activities
MEN-2	Multiple endocrine neoplasia syndrome type 2
MMRM	Mixed Model Repeated Measures
MRE	Magnetic Resonance Elastography
MRI	Magnetic Resonance Imaging
MRI-PDFF	MRI proton density fat fraction
MTC	Medullary thyroid carcinoma
NAb	Neutralizing Antibody
NAFL	Non-alcoholic fatty liver
NAFLD	Non-alcoholic fatty liver disease
NAS	NAFLD (Non-Alcoholic Fatty Liver Disease) Activity Score
NASH	Non-Alcoholic SteatoHepatitis
NYHA	New York Heart Association
PHQ-9	Patient Health Questionnaire 9
[redacted]	
PK/PD	Pharmacokinetics/Pharmacodynamics
PRO	Patient Reported Outcome
[redacted]	
PYY3-36	Peptide YY 3-36
QTcF	QT interval corrected for heart rate according to Fridericia's formula
SAE	Serious adverse event
s.c.	Subcutaneous
SBP	Systolic Blood Pressure
SD	Standard deviation
[redacted]	
SGLT2i	Sodium-glucose co-transporter-2 (SGLT2) inhibitors
SMBG	Self-monitoring blood glucose
SoA	Schedule of Assessments
T2DM	Type 2 diabetes mellitus
TSAP	Trial Statistical Analysis Plan
[redacted]	
ULN	Upper Limit of Normal
VCTE™	Vibration-Controlled Transient Elastography
VLDL	Very-low-density lipoprotein
WOCBP	Women of childbearing potential

Proprietary confidential information © 2023 Boehringer Ingelheim International GmbH or one or more of its affiliated companies

1. INTRODUCTION

1.1 MEDICAL BACKGROUND

NAFLD (also known as metabolic dysfunction-associated steatotic liver disease, MASLD) is a metabolic dysfunction that represents a range of liver damage conditions from steatosis alone (non-alcoholic fatty liver, NAFL, also known as metabolic dysfunction associated steatosis, MAS) to the inflammatory phenotype, NASH (also known as metabolic dysfunction-associated steatohepatitis, MASH) [R23-2769]. The latter involves persistent liver inflammation and may result in progressive fibrosis and loss of liver function [R23-1199].

The global prevalence of NAFLD is increasing and closely related to the worldwide growth of obesity and type 2 diabetes mellitus (T2DM), which are major drivers of NAFLD severity and progression [R23-1196]. It affects approximately 30% of the general population and is the most common liver disease in Western countries [R22-2292]. In a recent meta-analysis of 151 studies (N=101,028) [R23-1416], the prevalence of NAFLD in the overweight and obese population is 69.9% (95% confidence interval [CI] 65.4, 74.2) and 75.3% (95% CI 70.9, 79.2), respectively. Significant fibrosis (stages F2-4) presents in 20.3% (95% CI 11.3, 33.6) of overweight individuals with NAFLD and in 21.6% (95% CI 11.5, 36.9) of obese individuals with NAFLD. Advanced fibrosis (stages F3-F4) presents in 6.7% (95% CI 4.4, 10.0) of overweight individuals with NAFLD and 6.9% (95% CI 3.9, 11.9) of obese individuals with NAFLD. The meta-analysis included patients with obesity who were at risk of procedures (e.g. bariatric surgery); thus the actual prevalence of NAFLD in the overall population with obesity might be lower.

A recent pooled analysis of biopsy studies suggests that the transition from NAFLD to NASH is dynamic (Figure 1) [R22-2295]. It is estimated that 20% of individuals with NAFLD have NASH, and 20% of those may progress to cirrhosis over 3-4 decades. A limitation of this study was the lack of estimates for regression rates. Progression and regression rates were reported by a meta-analysis including both observational studies and randomised controlled trials (54 studies involving 26,738 patients using liver biopsy or imaging to diagnose NAFLD during a follow-up period of ≥48 weeks), which confirmed that the transition/progression from NAFL to NASH or more advanced stages of the disease is dynamic. Among NAFL patients, 31% developed NASH after 4.7 years (incidence rate 7.4/100 person-years). In 29% of patients with NASH, resolution occurred after a median of 3.5 years (incidence rate 5.1/100 person-years). Time to progress by one fibrosis stage for F0, F1, F2, and F3 was 9.9, 10.3, 13.3, and 22.2 years, respectively [R23-2419]. In another study in 733 patients from United States, United Kingdom, and Australia with clinical diagnosis and liver biopsy-confirmed NAFLD, patients with overweight or obesity had 2.35 and 5.70 greater odds of advanced fibrosis (stages F3-F4), respectively, versus those with normal weight [R17-3207].

Proprietary confidential information © 2023 Boehringer Ingelheim International GmbH or one or more of its affiliated companies

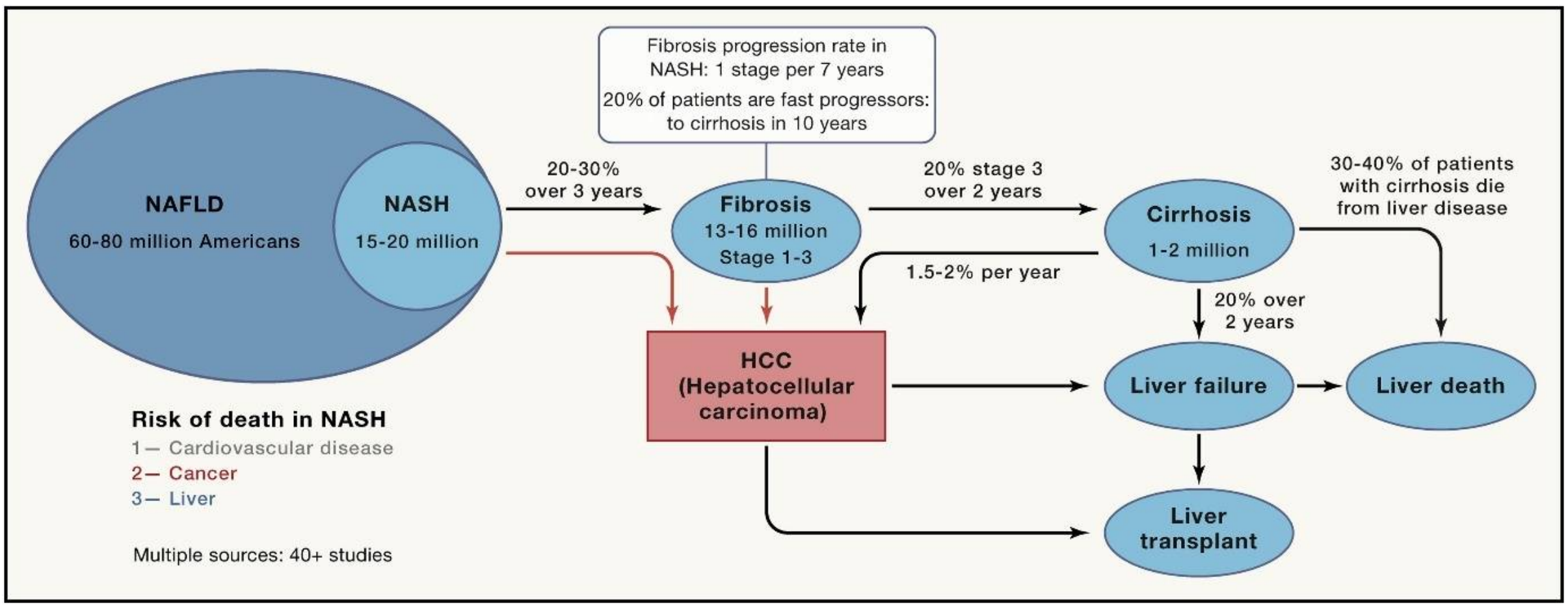

Figure 1 NAFLD natural history in the United States

Source data: [R22-2295]

NAFLD is associated with obesity, dyslipidemia, hypertension, insulin resistance, diabetes mellitus, and chronic kidney disease [R23-1199]. It is also associated with an increased risk of all-cause, cardiovascular, and cancer-related mortality [R23-1197]. Patients with NASH and fibrosis stages ≥F2 are at higher risk for liver-related events and mortality [R23-0537]. NASH also adversely impacts patients’ health-related quality of life (HRQoL) [R23-1198].

The identification and stratification of patients with at-risk NASH or advanced fibrosis can be performed using non-invasive tests such as FIB-4, ELF score, MRI-PDFF, cT1, MRI, FibroScan® VCTE, MRE and their derived scores (FAST and MAST score) [R23-0537, R23-1377]. It is thought that non-invasive imaging modalities are needed to replace liver biopsy for the diagnosis and monitoring of NASH in clinical trials [R23-1383]. For example, MRI-PDFF is a reliable, highly reproducible, and accurate non-invasive biomarker for histological improvement in disease activity [R23-1379]. A meta-analysis demonstrated that a ≥30% relative decline in MRI-PDFF is associated with higher odds of histologic response (2-point improvement in NAFLD Activity Score (NAS) with at least 1-point improvement in lobular inflammation or ballooning) and NASH resolution [R23-1379]. In addition, MRI-PDFF response (≥30% relative decline in MRI-PDFF) was an independent predictor of fibrosis regression with an adjusted odds ratio of 6.46 in a recent US study [R23-1380]. Importantly, the 2023 AASLD Guidance on NAFLD stated that improvement in alanine aminotransferase (ALT) and reduction in liver fat content by imaging in response to an intervention could be used as a surrogate for histological improvement in disease activity [R23-0537].

1.2 DRUG PROFILE

Mode of action

Survodutide is a dual agonist of glucagon-like-peptide (GLP) 1 receptor (GLP-1R) and glucagon receptor (GCGR). Targeting both GLP-1 and glucagon signalling pathways is expected to provide synergistic clinical benefit for chronic weight management and obesity.

Simultaneous dual activation of GLP-1R and GCGR by survodutide is anticipated to achieve improved glycaemic control, reduction in body weight, and improvement in NASH.

Proprietary confidential information © 2023 Boehringer Ingelheim International GmbH or one or more of its affiliated companies

Survodutide leads to full activation of the GLP-1R at the predicted therapeutic exposure, but only to a partial activation of the GCGR, which seems sufficient to achieve greater weight loss than GLP-1R activation alone, without activation of hyperglycaemic pathways.

Key PK characteristics

PK of survodutide was investigated after single s.c. doses of 0.3, 0.5, and 1.2 mg in trial 1404-0001 [c22991258-01]. C_{max} was 7, 13, and 34 nM, respectively. t_{max} was reached between 28 and 34 h. $t_{1/2}$ ranged from 109 to 115 h. Dose proportionality was demonstrated for C_{max}, $AUC_{0\text{-}168\text{ h}}$, and $AUC_{0\text{-}\infty}$.

In trial 1404-0021 [c33609485-01], a slightly higher average exposure was observed in Japanese compared with Caucasians. In a population analysis including data from trials 1404-0001, 1404-0003, and 1404-0021 [c22991258-01, c26759941-01, c33609485-01], survodutide PK was not different in Japanese and non-Japanese participants after accounting for body weight effects [c37765403].

Further details, also on multiple dosing, can be found in the investigator's brochure [c14085752].

Drug interactions

GLP-1R agonism reduces gastric emptying. This was assessed by measuring the effect of survodutide on the absorption of paracetamol in trial 1404-0001 [c22991258-01]. Paracetamol C_{max} and $AUC_{0\text{-}10}$ were slightly lower after a single dose of 0.3 mg survodutide, and strongly decreased after a single dose of 1.2 mg survodutide compared with administration of paracetamol alone. The effect of survodutide on gastric emptying decreases with multiple dosing in trial 1404-0003 [c26759941-01].

A population PK/PD analysis based on data from Japanese and non-Japanese subjects did not indicate meaningful ethnic differences in the survodutide effect on gastric emptying, as measured with paracetamol absorption test [c37765403].

Residual effect period

The residual effect period of survodutide is 28 days (half-life 112 h). This is the period after the last dose with measurable drug levels and/or PD effects still likely to be present.

Data from non-clinical studies

In a 4-week study in mice with diet-induced obesity, survodutide induced a body weight loss greater than the pure GLP-1R agonist liraglutide. Survodutide dose-dependently increased energy expenditure while liraglutide had no effect even at supra-therapeutic exposure. The increased energy expenditure contributes to a longer lasting negative energy balance than GLP-1R agonism alone, while achieving a favourable benefit/risk profile [c14085752].

In toxicity studies of up to 26 weeks in mice and up to 39 weeks in Cynomolgus monkeys, survodutide induced reduced food consumption and body weight loss or reduced body weight gain. These intended pharmacological effects limited the highest dose assessed in the toxicity studies performed in healthy (non-obese) animals and, therefore, only low exposure multiples to clinical use could be tested. All findings seen in the toxicity studies have been recorded in studies where food has been intentionally restricted or with treatments inducing unspecific

Proprietary confidential information © 2023 Boehringer Ingelheim International GmbH or one or more of its affiliated companies

stress, and therefore were interpreted as secondary effects. These observations included signs of metabolic stress for the liver, as indicated by hepatocellular vacuolation, slight increase of plasma bilirubin or changes in hepatocellular glycogen content, or changes of haematopoiesis in bone marrow, spleen, and thymus, as well as atrophy of the ovary. Cynomolgus monkeys, especially female monkeys, are considered oversensitive with regard to limiting body weight effects due to their low total body fat content (5.6% ± 3.81%), compared with humans and even more to patients with obesity.

In the non-clinical studies on embryofoetal development, there were no adverse effects on maternal toxicity and embryofoetal development up to and including the high dosage levels of 1498 µg/kg/week in mice and 252 µg/kg/week in rabbits, indicating that the compound is not teratogenic.

Data from clinical studies

Survodutide was studied in healthy volunteers in the single rising dose trial 1404-0001 [c22991258-01]. Similar to other GLP-R1 agonists, nausea and vomiting were seen with increasing doses up to 1.2 mg, and planned dose escalation to further levels was stopped due to severe nausea and vomiting in a number of healthy volunteers.

Survodutide has also been studied in otherwise healthy volunteers with obesity or overweight in the multiple rising dose trial 1404-0003 [c26759941-01] with dose escalation to investigate the safety and tolerability of different dose escalation schemes to determine a dose escalation scheme that minimise GI AEs. In Part A, survodutide was administered for 6 weeks in 4 different titration schemes (1 once-daily dose group and 3 once-weekly dose groups with weekly dose escalation). The most frequent AEs were GI disorders, such as nausea, dyspepsia, abdominal distention, and vomiting. Cardiac disorders were reported in the survodutide treated group with 9% AV block first-degree, 9% second-degree, 8% sinus tachycardia, and 6% ventricular extrasystoles. All of the cardiac AEs were considered mild to moderate in intensity, none was reported as severe. An independent review of 24 h Holter recordings of trial participants in Part A of the trial with ECG findings noted at the site using a 12-lead telemetry system was conducted by an independent cardiologist at a central ECG lab. It was concluded that the IMP given at 4 different doses did not show a notable increase in the findings reported by the cardiologist. The incidences in the categories were similar to those occurring at baseline and also not significantly different from those seen in the placebo-treated participants. In Part B of this trial, survodutide was administered for 16 weeks in 3 different titration schemes. As in Part A, the most frequent AEs were GI disorders, in this case nausea, vomiting, dyspepsia, diarrhoea, and eructation. No deaths, or protocol specified AESIs were reported in this trial.

In trial 1404-0021 in healthy Japanese volunteers [c33609485-01], GI disorders (decreased appetite, dyspepsia, diarrhoea, and nausea) were the most commonly reported type of AEs. There were no clinically relevant cardiac disorders reported. No unexpected safety or tolerability concerns were noted. The most frequently reported AEs were GI disorders.

In the Phase II trial 1404-0002 in 411 participants with T2DM and obesity/overweight [c36750061-01], survodutide showed an overall good safety profile during the trial with GI events (nausea, vomiting, and diarrhoea) being the most common AEs. Dose-dependent HbA_{1c} reductions were observed with survodutide treatment in patients with T2DM and overweight/obesity. Weekly injections with survodutide effectively lowered absolute HbA_{1c} by up to –1.88% (Week 16) compared with the reduction of 1.47% with open-label

Proprietary confidential information © 2023 Boehringer Ingelheim International GmbH or one or more of its affiliated companies

semaglutide [P22-07379]. Treatment with survodutide induced greater body weight loss than open-label semaglutide after 16 weeks of treatment: −8.95% with the highest 3.6 mg dose vs. −5.40% with semaglutide 1.0 mg [P22-08755]. Severe AEs and SAEs were reported at low frequencies, 5.3% and 3.6% in all participants treated with survodutide, respectively, with similar frequencies reported in the placebo group. There were no cases of pancreatitis. There were no serious or severe cardiac AEs. Of note, 11.9% of participants treated with survodutide discontinued treatment due to GI AEs.

[redacted]

For a more detailed description of the survodutide profile, please refer to the current investigator's brochure [c14085752].

1.3 RATIONALE FOR PERFORMING THE TRIAL

Currently, there is no approved pharmacological treatment for NASH at any disease stage, representing a huge unmet medical need. Non-liver-specific management includes weight loss, lifestyle changes and treatment of associated metabolic syndromes [P23-02853, R23-1381]. Weight loss is one of the most critical interventions for obesity and NAFLD/NASH. The hepatic histological beneficial effect of weight loss was demonstrated for both surgical interventions (bariatric surgery) and lifestyle modifications [R23-1378, R23-1382]. In addition, there is evidence of a dose-response relationship between the magnitude of weight loss and improvement of liver inflammation, ballooning, and resolution of NAFLD or NASH [R23-1384]. A paired liver biopsy study showed that 25% of the subjects achieved resolution of steatohepatitis after lifestyle modifications for 52 weeks. A higher proportion of subjects with ≥5% weight loss had NASH resolution than those with <5% weight loss. Among patients who lost ≥10% of their weight, 90% had resolution of NASH, and 45% had regression of liver fibrosis [R23-1382].

The Phase II trial 1404-0036 in participants with obesity [c31754142-05] demonstrated that s.c. survodutide as an adjunct to a reduced-calorie diet and increased physical activity was effective for weight loss, while displaying a satisfactory tolerability profile. The weight loss was accompanied by a consistent improvement in weight-related complications, indicated by CV risk factors, lipid profile, and glycaemic factors as well as improvements in clinical outcome assessments.

This Phase III trial investigates the effect of survodutide once weekly versus placebo on liver fat and body weight as an adjunct to a reduced-calorie diet and increased physical activity in participants with presumed or confirmed NASH and obesity (BMI ≥30 kg/m^2) or overweight (BMI ≥27 kg/m^2) with one or more weight-related co-morbidities.

Proprietary confidential information © 2023 Boehringer Ingelheim International GmbH or one or more of its affiliated companies

The study of exploratory and probable valid biomarkers will mostly be hypothesis-generating or supportive to endpoints. These findings will be used to expand the understanding of NAFLD/NASH and survodutide, e.g. by learning more about the mechanism of action of the IMP or the biology of the disease, thereby allowing correlation of patient subgroups with differential responses to treatment and/or prognosis and supporting subsequent trial designs regarding dose and schedule selection.

[redacted]

In order to be able to address future scientific questions, trial participants will be asked to voluntarily donate biospecimens for banking (see Section 5.5). If the trial participant agrees, banked samples may be used for future biomarker research and drug development projects, e.g. to identify patients that are more likely to benefit from a treatment or experience an AE, or to gain a mechanistic or genetic understanding of the product's effects and thereby better match patients with therapies.

1.4 BENEFIT - RISK ASSESSMENT

The overall benefits and safety profile of survodutide are outlined in previous sections. More information on the benefits, risks, and known AEs is presented in the latest version of the investigator's brochure [c14085752].

1.4.1 Benefits

Trial participants treated with survodutide, together with the close contact with the trial site and counselling by a dietician or a similar qualified healthcare professional, are expected to achieve significant reduction in liver fat content and body weight, as well as improvement of the weight-related complications [redacted]

1.4.2 Risks

There are no important identified risks specific for survodutide, based on the toxicology programme or any clinical trials conducted for this product to date.

There are 4 important potential risks based on other GLP-1R agonists currently approved (class effect), and data from the completed Phase II studies of survodutide. The 4 important

Proprietary confidential information © 2023 Boehringer Ingelheim International GmbH or one or more of its affiliated companies

potential risks include MTC (C-cell carcinogenicity), acute pancreatitis, pancreatic cancer, and pre-renal acute kidney injury due to dehydration.

The risks for participants caused by participation in the trial, including the trial procedures and exposure to the trial drug, are reasonably low and do not outweigh the potential benefits. The expected side effects are known to be temporary, dose dependent, easy to monitor, and manageable in clinical trials. An overview of trial related risks is presented in Table 1.

Proprietary confidential information © 2023 Boehringer Ingelheim International GmbH or one or more of its affiliated companies

Table 1 Overview of trial related risks

**Possible or known risks of clinical relevance for this trial**	**Summary of data, rationale for the risk**	**Mitigation strategy**
**Survodutide**		
GI disorders (nausea, vomiting, and diarrhoea)	Well-known side effects for GLP-1R agonist-based therapies, mostly during dose escalation phase	Slow and careful dose escalation. Instructions to be given on management of GI symptoms (Section 4.1.4.1)
Acute pancreatitis and pancreatic cancer	Acute pancreatitis is a known side effect for GLP-1R agonist-based therapies.<br>Pancreatic cancer is not a recognised risk in humans.	An extensive safety laboratory will be performed.<br>Timely detection, evaluation, and follow-up of laboratory alterations in selected laboratory parameters to ensure trial participants' safety.
MTC	To date there is no evidence that survodutide causes such AE.<br>MTC is a known side effect for GLP-1R agonist-based therapies in rodents.<br>GLP-1R agonist-based therapies are contraindicated in patients with a personal or family history of MTC.	An extensive safety laboratory will be performed.<br>Timely detection, evaluation, and follow-up of laboratory alterations in selected laboratory parameters to ensure trial participants' safety.
Acute kidney injury (pre-renal) due to dehydration	Related to severe GI AEs of GLP-1R agonist class (severe vomiting may cause dehydration)	In case of vomiting, nausea, and diarrhoea, fluid intake should be increased.<br>Decreased renal function will be monitored.
Cardiac disorders (tachycardia and conduction disorders)	Tachycardia is a known side effect for GLP-1R agonist-based therapies.<br>Cardiac conduction disorders were described for GLP-1R agonists in clinical trials and in the prescribing information for liraglutide (Saxenda) [R22-0131], reported as first degree atrioventricular block, right bundle branch block, or left bundle branch block.<br>Survodutide has a low pro-arrhythmic potential based on animal studies.	Centralised ECG monitoring will be performed at screening, randomisation and at all in-clinic visits during the dose escalation and maintenance periods, and criteria for QT prolongation are defined.
Acute gallbladder disease	Well-known side effect for GLP-1R agonist-based therapies, and well-known AE of rapid significant weight loss with any modalities	An extensive safety laboratory will be performed.<br>Timely detection, evaluation, and follow-up of laboratory alterations in selected laboratory parameters to ensure trial participants' safety.

Table 1 (cont'd) Overview of trial related risks

**Possible or known risks of clinical relevance for this trial**	**Summary of data, rationale for the risk**	**Mitigation strategy**
Systemic hypersensitivity reaction	After administration of any biologic agent or protein, there is a possibility of occurrence of adverse immune reactions which can be local (e.g. redness, pruritus, and or swelling at the injection site) or systemic (e.g. anaphylactic reactions).	Trial participants with a history of allergy/hypersensitivity to the systemically administered trial medication agent or its excipients are excluded from the trial.<br>In case of systemic hypersensitivity reactions including anaphylactic reaction emerging during or after administration of trial medication, the investigator should consider in accordance with severity of the reaction and local standard of care to interrupt and treat the condition.
**Trial procedures**		
Insufficient weight loss	Trial participants who will receive placebo may not achieve sufficient weight loss during the trial, but there may also be non-responders to survodutide who may not lose weight.	Trial participants in the placebo group will remain on standard of care that includes diet and exercise counselling.
Injection site reactions	Well-known side effects for injectables	Instructions will be given.<br>Injection site will be monitored during every in-clinic visit.
**Other risks**		
Drug-induced liver injury	Rare but severe event, thus under constant surveillance by sponsors and regulators	Timely detection, evaluation, and follow-up of laboratory alterations in selected liver laboratory parameters to ensure trial participants' safety.<br>Section 5.2.6.1.4 provides guidance for the investigators on how to evaluate and treat trial participants with suspected DILI.
Suicide risk, depression, and mood disorder	Survodutide could act on central GLP-1R (limited data on survodutide).	C-SSRS will be applied at every visit as required by regulatory agencies.

In this trial, the following safety measures will be applied to minimise the risk for trial participants:

- Trial participants in all groups, including placebo group will receive standard of care that includes diet and physical activity counselling.
- There are no risks expected from stopping the IMP during the trial.
- Centralised ECG monitoring will be performed at screening, randomisation and at in-clinic visits during the dose escalation and maintenance periods, and criteria for QT prolongation are defined.

Proprietary confidential information © 2023 Boehringer Ingelheim International GmbH or one or more of its affiliated companies

- Instructions will be given to the trial participants to help improve the tolerability especially during the escalation phase. For trial participants who do not tolerate the assigned escalation schedule, the dose escalation can be modified according to the predefined process. Details are described in Section 4.1.4.1 and Section 10.1.
- During the trial, participants will be under medical observation and thoroughly monitored for both expected and unexpected AEs including administration and evaluation of the C-SSRS, at every visit as required by regulatory agencies for compounds acting on CNS located receptors.
- Paediatric patients (less than 18 years old) are excluded from the trial.
- Participants who develop T2DM will be educated about signs and symptoms of hypoglycaemia, how to treat hypoglycaemia, and how to collect information for each episode of hypoglycaemia. Hypoglycaemic events should be treated, and additional glucose monitoring should be implemented per investigator discretion and medical judgement. In cases of hyperglycaemia, glycaemic rescue treatment should be initiated at the discretion of the investigator.
- WOCBP who are pregnant, nursing or intend to become pregnant or are not using an adequate contraceptive method (Section 4.2.2.3) are excluded from the trial.

To continue the assessment of the safety of survodutide, adjudication of some events (see Section 5.2.6.1.7) will be performed in this trial by a Clinical Event Committee (CEC). The progress of the trial will also be assessed at regular intervals by an independent Data Monitoring Committee (DMC). Further details are described in Section 8.7, the CEC Charter, and the DMC Charter.

1.4.3 Discussion

Based on the currently available data, in trial participants with a BMI ≥30 kg/m² or a BMI ≥27 kg/m² with at least one weight-related complication, dual agonism at the GLP-1R and GCGR is expected to result in superior body weight loss and associated reduction in liver fat content. Nausea, vomiting, diarrhoea, constipation, abdominal pain, dyspepsia, and dizziness are considered ADRs for survodutide. There are no important identified risks defined for survodutide. Important potential risks for survodutide are MTC (C-cell carcinogenicity), pancreatic cancer, acute pancreatitis, and pre-renal acute kidney injury due to dehydration. In the context of the anticipated benefit of survodutide, the benefit risk evaluation of the compound based on the available preclinical and clinical data, is favourable. Overall, the potential benefits, coupled with an acceptable safety profile and a well-controlled clinical trial environment support the initiation of the trial.

Proprietary confidential information © 2023 Boehringer Ingelheim International GmbH or one or more of its affiliated companies

2. TRIAL OBJECTIVES AND ENDPOINTS

2.1 MAIN OBJECTIVES, PRIMARY AND SECONDARY ENDPOINTS

2.1.1 Main objectives

This trial aims to provide evidence of the efficacy, safety, and tolerability of survodutide administered once weekly as an adjunct to a reduced-calorie diet and increased physical activity, in comparison with placebo, in participants with presumed or confirmed NASH and obesity (BMI ≥30 kg/m^2) or overweight (BMI ≥27 kg/m^2) with at least one of the following weight-related complications (treated or untreated): hypertension, dyslipidaemia, obstructive sleep apnoea, cardiovascular disease or T2DM.

The primary objective is to demonstrate superiority of survodutide in:

- Reduction in liver fat content assessed by MRI-PDFF, defined as ≥30% from baseline at Week 48 (odds ratio, survodutide vs. placebo), AND
- The difference in adjusted means of relative change (%) in body weight from baseline to Week 48 (survodutide vs. placebo)

The secondary (exploratory) objectives are to demonstrate the superiority of survodutide vs. placebo in terms of the difference in adjusted means of changes from baseline to Week 48 in liver fat content assessed by MRI-PDFF, cT1, ALT levels, waist circumference, HOMA-IR, liver stiffness assessed by MRE, and liver volume assessed by MRI.

All treatment comparisons will be as randomised, including the effects of any early treatment discontinuation or any prohibited (anti-obesity) treatment including the effects of anti-obesity treatments, including GLP-1R agonists. In addition, treatments will be compared as randomised, but excluding effects of any early treatment discontinuation and any prohibited (anti-obesity) treatments.

2.1.2 Primary endpoints

The co-primary endpoints are:

- Relative reduction in liver fat content of at least 30% from baseline to Week 48 (yes/no) assessed by MRI-PDFF, AND
- Relative change (%) in body weight from baseline to Week 48

2.1.3 Secondary endpoints

The secondary endpoints are:

- Absolute and relative change (%) from baseline to Week 48 in liver fat content assessed by MRI-PDFF
- Reduction from baseline to Week 48 in cT1 levels of ≥80 ms (yes/no)
- Absolute and relative change from baseline to Week 48 in ALT levels
- Absolute and relative change from baseline to Week 48 in waist circumference [cm]
- Absolute change from baseline to Week 48 in HOMA-IR (FPI [mlU/L]·FPG [mmol/L]/22.5)

Proprietary confidential information © 2023 Boehringer Ingelheim International GmbH or one or more of its affiliated companies

- Absolute and relative change from baseline to Week 48 in liver stiffness assessed by MRE
- Absolute and relative change in liver volume [mL] from baseline to Week 48 measured using MRI

2.2 FURTHER OBJECTIVES AND FURTHER ENDPOINTS

2.2.1 Further objectives

Further objectives include evaluation of safety and tolerability, liver fat content, population PK of survodutide, biochemical/[redacted]biomarkers related to obesity and NAFLD activity.

2.2.2 Further endpoints

[redacted]

Absolute change from baseline to Week 48 in:

[redacted]
- Systolic blood pressure [mmHg]
- Diastolic blood pressure [mmHg]

[redacted]
- HbA_{1c} [%, mmol/mol], overall and per diabetes status

[redacted]

Proprietary confidential information © 2023 Boehringer Ingelheim International GmbH or one or more of its affiliated companies

[redacted]

- ELF score

[redacted]

- cT1 values [ms]

[redacted]

- High-sensitivity C-reactive protein (hsCRP)

Proprietary confidential information © 2023 Boehringer Ingelheim International GmbH or one or more of its affiliated companies

- Uric acid [µmol/L]

[redacted]

Categorical based on relative reduction from baseline to Week 48 in:

- Liver fat content of at least 50% (yes/no) assessed by MRI-PDFF
- Liver fat content of at least 70% (yes/no) assessed by MRI-PDFF

Miscellaneous

[redacted]

- Liver fat content (<5%) assessed by MRI-PDFF at Week 48 (yes/no)

[redacted]

Proprietary confidential information © 2023 Boehringer Ingelheim International GmbH or one or more of its affiliated companies

3. DESCRIPTION OF DESIGN AND TRIAL POPULATION

3.1 OVERALL TRIAL DESIGN

This is a randomised, double-blind, placebo-controlled 48-week Phase III clinical trial comparing survodutide 6.0 mg once weekly to placebo once weekly, as an adjunct to a reduced-calorie diet and increased physical activity, in participants with presumed or confirmed NASH and obesity (BMI ≥30 kg/m^2) or overweight (BMI ≥27 kg/m^2) and at least one weight-related comorbidity. The overall trial design and dose escalation schedule are depicted in Figure 2.

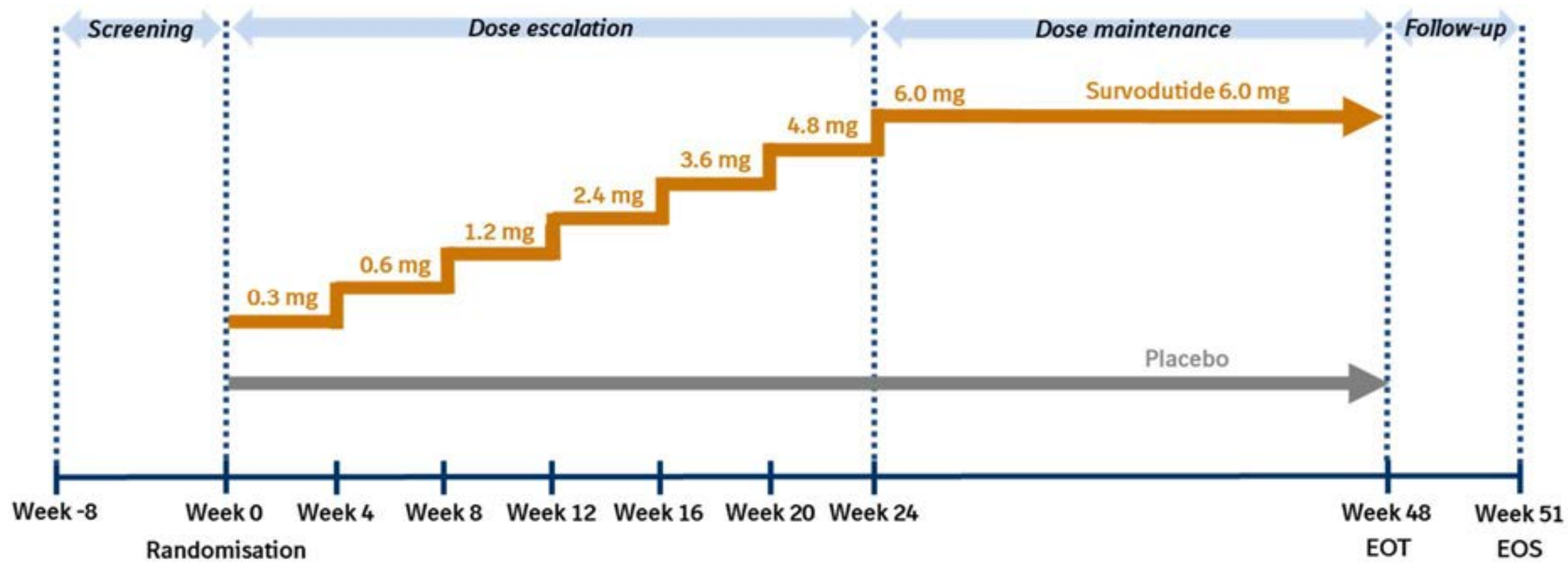

Figure 2 Trial design

The trial has a screening period of up to 8 weeks to assess participant eligibility criteria. Re-screening of previously screen-failed participants will be permitted providing the reasons for screen failure were reversible and have been resolved, based on the investigator's judgement. Re-screening is only allowed once.

Approximately 160 eligible participants will be randomised to survodutide or matching placebo in a 2:1 ratio (survodutide: approximately 106 participants, placebo: approximately 53 participants) as an adjunct to a reduced-calorie diet and increased physical activity. Randomisation will be stratified by the presence of T2DM.

The treatment duration is 48 weeks in total, including a dose escalation period of up to 24 weeks (approximately 4 weeks between each dose escalation; up to a maximum of 32 weeks if 1 or 2 re-escalation attempts are needed due to GI effects) and a maintenance period of up to 24 weeks. Remote (telemedicine, if allowed per local regulations) contacts are [redacted] The last administration of IMP is at Week 47. A Follow-up/End-of-Study (FU/EOS) Visit for safety assessments is scheduled at Week 51 (i.e. 4 weeks after the last IMP administration or 3 weeks after Week 48/End of Treatment [EOT] Visit). The end of trial is defined as the date the last trial participant completes the last FU/EOS visit (Week 51).

Participants who discontinue IMP prematurely will be asked to continue the trial visits as planned. All efforts should be made to conduct the Week 48 visit in clinic, in particular the MRI-PDFF measurement, for all participants, including those who discontinued IMP

Proprietary confidential information © 2023 Boehringer Ingelheim International GmbH or one or more of its affiliated companies

prematurely. However, if this is not possible, a phone or telemedicine contact should be made at Week 48 to obtain vital status. If possible, weight, adverse events (AEs), and concomitant therapy changes should be recorded.

All trial participants will receive counselling for diet and physical activity (see Section 4.2.1.2).

During the trial, participants should not initiate any anti-obesity medication. GLP-1R agonists, GLP-1R agonist/insulin/GIP combinations, amylin analogues, and DPP4i are not allowed while on study treatment (see also Section 4.2.2.1).

3.2 DISCUSSION OF TRIAL DESIGN, INCLUDING THE CHOICE OF CONTROL GROUP(S)

A randomised, double blind, placebo-controlled design was chosen for this trial as an accepted standard trial design for a Phase III trial. The treatment duration of 48 weeks was chosen as sufficient to potentially show changes in liver fat and body weight. A placebo control was included to evaluate the absolute effects of survodutide on efficacy and safety. This is acceptable as no standard treatments for NASH and/or fibrosis are available and in accordance with the guidelines provided by regulatory authorities.

3.3 SELECTION OF TRIAL POPULATION

A sufficient number of subjects will be screened in approximately 56 sites and approximately 4-8 countries to randomise approximately 160 trial participants.

3.3.1 Main diagnosis for trial entry

The main diagnosis required for trial entry is presumed or confirmed NASH and overweight or obesity.

Please refer to Section 8.3.1 (Source Documents) for the documentation requirements pertaining to the inclusion and exclusion criteria.

3.3.2 Inclusion criteria

Participants are eligible to be included if all the following criteria apply:

1. Male or female, age ≥18 years at the time of signing informed consent, and at least the legal age of consent in countries where it is >18 years
2. Signed and dated written informed consent in accordance with ICH-GCP and local legislation prior to admission to the trial
3. BMI ≥30 kg/m^2, OR BMI ≥27 kg/m^2 and at least one weight-related comorbidity at screening:
   - Hypertension (defined as repeated, i.e. at least 3 measurements in resting condition, SBP values of ≥140 mmHg and/or DBP values of ≥90 mmHg in the absence of anti-hypertensive treatment, or intake of at least 1 anti-hypertensive drug to maintain a normotensive blood pressure)
   - Dyslipidaemia (defined as at least 1 lipid-lowering treatment required to maintain normal blood lipid levels, or low-density lipoprotein [LDL] ≥160 mg/dL [≥4.1

Proprietary confidential information © 2023 Boehringer Ingelheim International GmbH or one or more of its affiliated companies

mmol/L] or triglycerides ≥150 mg/dL [≥1.7 mmol/L], or high-density lipoprotein (HDL] <40 mg/dL (<1.0 mmol/L] for men or HDL<50 mg/dL (<1.3 mmol/L) for women

- Obstructive sleep apnoea
- Cardiovascular disease (e.g. heart failure with New York Heart Association [NYHA] functional class II-III, history of ischaemic or haemorrhagic stroke or cerebrovascular revascularisation procedure [e.g. carotid endarterectomy and/or stent], MI, coronary artery disease, or peripheral vascular disease)
- T2DM (diagnosed at least 180 days prior to screening, with glycated haemoglobin [HbA_{1c}] ≥6.5% [48 mmol/mol] and <10% [86 mmol/mol] as measured by the central laboratory at screening)

4. Presumed/confirmed NASH: Evidence of hepatic steatosis (defined by an MRI-PDFF ≥8% at screening) with the exclusion of secondary causes of hepatic fat accumulation such as significant alcohol consumption, other chronic liver diseases and/or steatogenic medications, AND at least one of the following:
   - MRE ≥2.61 and <4.68 kPa at screening, OR
   - MAST score ≥0.242 at screening, OR
   - FAST score ≥0.5 at screening, OR
   - FibroScan® VCTE™ ≥8 kPa and <20 kPa at screening, OR
   - FIB-4 score ≥2.67 and <3.48 at screening, OR
   - ELF score >7.7 and <11.3 at screening, OR
   - cT1 ≥875 ms at screening, OR
   - Recent liver biopsy (within 3 years of screening) consistent with NASH (defined as the presence of steatosis, inflammation, and ballooning) with stage ≤3 fibrosis according to the NASH Clinical Research Network classification [R16-0241].
5. WOCBP[1] must be ready and able to use highly effective methods of birth control per ICH M3 (R2) that result in a low failure rate of less than 1% per year when used consistently and correctly. A list of contraception methods meeting these criteria and instructions on the duration of their use will be provided in the participant information and Section 10.2.
6. In the investigator's opinion, participants are well-motivated, capable, and willing to:
   - Learn how to self-inject the IMP, as required for this protocol (persons with physical limitations who are not able to perform the injections must have the assistance of an individual trained to inject the IMP) OR
   - Inject the IMP or accept injection from a designated person
   - Follow study procedures for the duration of the study, including, but not limited to: follow lifestyle advice (for example, dietary restrictions and exercise, and exercise plan), maintain a diary, complete required questionnaires, and handle the IMP as described in the IFU

[1] A woman is considered of childbearing potential, i.e. fertile, following menarche and until becoming post-menopausal unless permanently sterile. Permanent sterilisation methods include hysterectomy, bilateral salpingectomy, and bilateral oophorectomy. Tubal ligation is NOT a method of permanent sterilisation. A postmenopausal state is defined as no menses for 12 months without an alternative medical cause.

Proprietary confidential information © 2023 Boehringer Ingelheim International GmbH or one or more of its affiliated companies

3.3.3 Exclusion criteria

Participants are excluded if any of the following criteria apply:

Liver:

1. Current or history of significant alcohol consumption (defined as intake of >210 g/week in men and >140 g/week in women on average over a consecutive period of more than 3 months) or inability to reliably quantify alcohol consumption based on the investigator's judgement within the last 5 years.
2. Intake of medications associated with liver injury, hepatic steatosis or steatohepatitis (refer to Section 4.2.2.1).
3. History of other chronic liver diseases (e.g. viral hepatitis, autoimmune liver disease, primary biliary cholangitis , primary sclerosing cholangitis, Wilson's disease, hemochromatosis, A1At deficiency, history of liver transplantation). Hepatitis B and C testing will be done at Visit 1. Participants with positive HBsAg should be excluded. Participants treated for hepatitis C must have a negative RNA test at screening and also be HCV RNA negative for at least 3 years prior to screening in order to be eligible for the trial.
4. Cirrhosis based on clinical assessment, abdominal imaging, liver histology or non-invasive tests assessed at screening (ELF ≥11.3, FIB-4 ≥3.48, FibroScan® VCTE™ ≥20 kPa, or MRE ≥4.68 kPa)
5. Current decompensated liver disease or previous hepatic decompensation (ascites, spontaneous bacterial peritonitis, portal hypertension bleeding, hepatic encephalopathy, hepatorenal syndrome).
6. Previous or current evidence of portal hypertension (e.g. splenomegaly, oesophageal varices, or other portosystemic collateral pathways).
7. Any of the following liver laboratory test abnormalities at screening:
    - Serum AST and/or ALT elevation ≥5x ULN
    - Total serum bilirubin concentration ≥1.2x ULN (except for cases of known Gilbert's Syndrome)
    - Alkaline phosphatase >2x ULN
    - INR ≥1.3 (unless patient is on anticoagulants)

Obesity:

8. Body weight variation (self-reported) >5% within 3 months before screening
9. Medications for obesity within 3 months before screening (see Section 4.2.2.1)
10. Previous or planned (during the trial period) treatment for obesity with surgery or a weight loss device, or prior surgery of the GI tract that could interfere with body weight. The following are allowed: (1) liposuction and/or abdominoplasty, if performed >1 year before screening, (2) lap banding, if the band has been removed >1 year before screening, (3) intragastric balloon, if the balloon has been removed >1 year before screening, (4) duodenal-jejunal bypass sleeve, if the sleeve has been removed >1 year before screening (5) appendectomy, (6) simple hernia repair, or (7) cholecystectomy.
11. Obesity induced by other endocrinologic disorders (i.e. Cushing Syndrome) or diagnosed monogenetic or syndromic forms of obesity (i.e. melanocortin 4 receptor deficiency, leptin deficiency, or Prader Willi Syndrome)

Proprietary confidential information © 2023 Boehringer Ingelheim International GmbH or one or more of its affiliated companies

Diabetes:

12. Treatment with medications for the indication of T2DM as described in Section 4.2.2.1
13. Uncontrolled and potentially unstable diabetic retinopathy or maculopathy, verified by an eye examination within 3 months before screening or in the period between screening and randomisation (for details, see Section 5.2.5.2)

Mental health:

14. Answered “yes” to any of the suicide-related behaviours (actual attempt, interrupted attempt, aborted attempt, preparatory act or behaviour) or to the non-suicidal self-injurious behaviour question on the “Suicidal Behaviour” section of the C-SSRS related to the past 2 years or during the screening period.
15. Answered “yes” to Question 4 (active suicidal ideation with some intent to act, without specific plan) or to Question 5 (active suicidal ideation with specific plan and intent) on the “Suicidal Ideation” section of the C-SSRS related to the past 3 months prior to screening or during the screening period.)
16. Reported a history of psychiatric inpatient hospitalisation (due to significant active or unstable major depressive disorder or other severe psychiatric disorder, e.g. schizophrenia, bipolar disorder, or other serious mood or anxiety disorder) within the past 1 year before screening, OR are not stabilised on their psychiatric medications (i.e. change of medication type or dosage adjustment within 8 weeks prior to screening), OR major depressive symptoms defined as a PHQ-9 score of ≥15 at screening or during the screening period.
17. Participants with major depressive disorder or generalized anxiety disorder whose disease state is considered stable and expected to remain stable throughout the course of the study, in the opinion of the investigator, may be considered for inclusion if they are not on excluded medications.

Other medical:

18. Any treatment with medications which may cause significant weight change started within 3 months prior to screening (included but not limited to systemic glucocorticoid therapy, tricyclic antidepressants, atypical antipsychotic, mood stabilisers, anti-hypertensive, antihistamines, and anti-epileptics; refer to Table 8). Participants taking these medications for more than 3 months prior to screening with stable body weight are allowed.
19. Known clinically significant gastric emptying abnormality (e.g. severe diabetic gastroparesis or gastric outlet obstruction)
20. Uncontrolled hypertension (mean SBP ≥160 mmHg and/or mean DBP ≥100 mmHg, measured in at least 2 measurements performed 5 min apart) at screening (assessed as described in Section 5.2.2).
21. Uncontrolled (as per investigators assessment) hypo- or hyperthyroidism at screening
22. Renal impairment measured as eGFR <30 mL/min/1.73 m^2 by the central laboratory at screening
23. History of either chronic or acute pancreatitis or elevation of serum lipase or amylase >2x ULN as measured by the central laboratory at screening
24. Major surgery performed within 3 months prior to randomisation or planned for the duration of the trial

Proprietary confidential information © 2023 Boehringer Ingelheim International GmbH or one or more of its affiliated companies

25. Personal or family history of medullary thyroid carcinoma or multiple endocrine neoplasia syndrome type 2
26. Calcitonin ≥100 ng/L (29.26 pmol/L) as measured by the central laboratory at screening
27. Recent evidence (within 3 months prior to screening) of acute or unstable cardiovascular events, e.g. hospitalisation for HF, acute coronary syndrome, unstable angina, MI, ischaemic or haemorrhagic stroke, transient ischaemic attack, and/or acute peripheral vascular event
28. HF with NYHA functional class IV
29. QTc (Fridericia) mean interval that is greater than 500 ms at screening (triplicate ECG) or personal or family history of long QT syndrome
30. History of infection with HIV or positive HIV test at screening
31. History of organ transplantation (corneal transplants [keratoplasty] allowed) or awaiting an organ transplant
32. Haematological condition that may interfere with HbA_{1c} measurement (e.g. haemolytic anaemias, sickle cell disease)
33. Women who are pregnant, nursing or who plan to become pregnant while in the trial
34. Known or suspected hypersensitivity to the IMP or related products
35. Contraindication to MRI including, but not limited to, severe claustrophobia, inner ear implant, pacemakers or other implanted cardiac rhythm management devices, intracranial aneurism clips incompatible with MRI, any other metallic, non-MRI compatible implanted devices (e.g. hip joint replacement), a history of intra-orbital metal fragments that have not been removed, and weight or girth that exceeds scanner capabilities.
36. Confirmed diagnosis of malignancy within 5 years prior to screening, except for basal or squamous-cell carcinoma of the skin that has been treated successfully. Trial participants with a history of other malignancies that have been treated with curative intent and who have no demonstrable disease recurrence within 5 years may also be eligible if approved following discussion with the sponsor. Trial participants under evaluation for malignant disease currently are not eligible for study participation.
37. Platelet count <140 000/mm^3

General:

38. Participants not expected to comply with the protocol requirements or not expected to complete the trial as scheduled (e.g. chronic alcohol or drug abuse or any other condition that, in the investigator's opinion, makes the participant an unreliable trial participant)
39. Participants who must or wish to continue the intake of prohibited medication (see Section 4.2.2.1) or any drug considered likely to interfere with the safe conduct of the trial
40. WOCBP who are using oral contraceptives during the screening visit and are
    - Not willing to change to non-oral contraceptives at least 7 days prior to first dose of IMP

    or

    - Not willing to add an additional barrier method of contraception during the entire treatment period with IMP and 28 days thereafter (see Appendix 10.2)

Proprietary confidential information © 2023 Boehringer Ingelheim International GmbH or one or more of its affiliated companies

41. Currently enrolled in another investigational device or drug trial, or less than 30 days or 5 half-lives, whichever is longer, since ending another investigational device or drug trial or receiving other investigational treatment(s); current or previous enrolment in strictly observational trials is allowed
42. Previous randomisation in this trial
43. Any condition not covered by any of the other exclusion criteria, which in the investigator's opinion, might jeopardise the participant's safety or compliance with the protocol.

3.3.4 Discontinuation of trial participants from treatment or assessments

Trial participants may discontinue trial IMP or withdraw consent to trial participation as a whole ("withdrawal of consent") with very different implications; please see Sections 3.3.4.1 and 3.3.4.2.

Measures to control the withdrawal rate include careful trial participant selection, appropriate explanation of the trial requirements and procedures prior to trial enrolment, as well as the explanation of the consequences of withdrawal.

The decision to discontinue trial IMP or withdraw consent to trial participation and the reason (if provided) must be documented in the trial participant files and CRF. If applicable, consider the requirements for AE collection and reporting (see Section 5.2.6.1.7).

3.3.4.1 Discontinuation of trial treatment

Participants may be discontinued at any time during the trial. A participant who discontinues treatment will have an EOT Visit 7 days after the last dose or if more than 7 days, then as soon as possible. Efforts must be made to have participants who discontinue IMP to continue in the trial with the scheduled visits and assessments. Visits may be converted to remote visits or phone calls if the participant is not willing to attend the clinic visits. At minimum, a contact at the Week 51 visit will be requested to obtain vital status and body weight.

Participants should be informed of anticipated GI side effects, and investigators should provide guidance on how to avoid or overcome them.

The participant must be discontinued from IMP if the following applies:

- The trial participant wants to discontinue the IMP. The trial participant will be asked to explain the reasons but has the right to refuse to answer.
- The trial participant has repeatedly shown to be non-compliant with important trial procedures and, in the opinion of both the investigator and sponsor representative, the safety of the trial participant cannot be guaranteed as he/she is not willing or able to adhere to the trial requirements in the future.
- Pregnancy or intention of becoming pregnant of female trial participant. In case of pregnancy, the trial participant will be expected to attend the regularly scheduled study visits as outlined above. She will be followed up during the trial and until birth or termination of the pregnancy.
- When pancreatitis is suspected, IMP should be stopped. If pancreatitis is confirmed, IMP should be discontinued.

Proprietary confidential information © 2023 Boehringer Ingelheim International GmbH or one or more of its affiliated companies

- In case of evidence of QTcF interval >500 ms or an increase in QTcF interval more than 60 ms AND in QTcF interval above ULN (470 ms for women, 450 ms for men) compared with the last ECG recording performed prior to the first IMP administration obtained from central ECG reading, IMP should be stopped and then discontinued, if this finding is confirmed by central ECG reading. If any local ECG is needed due to any acute symptom/event (e.g. syncope), QTcF should be measured.
- Suicidality:
  - Answered “yes” to either Question 4 (Active Suicidal Ideation with Some Intent to Act, Without Specific Plan) or to Question 5 (Active Suicidal Ideation with Specific Plan and Intent) on the “Suicidal Ideation” section of the C-SSRS since last visit
  - Answered “yes” to any of the suicide-related behaviours (Actual attempt, Interrupted attempt, Aborted attempt, Preparatory act or behaviour) or to the non-suicidal self-injurious behaviour question on the “Suicidal Behaviour” section of the C-SSRS since last visit
- PHQ-9 score ≥15

  Trial participants should be referred to an MHP to assist with participant’s condition evaluation. If a trial participant’s psychiatric disorder can be adequately treated with psycho- and/or pharmacotherapy, then the participant, at the discretion of the investigator (in agreement with the MHP), may be continued in the trial on randomised therapy. If the trial participant refuses to be referred to an MHP, the investigator will decide if the participant can remain in the trial.
- Initiation of prohibited medications as per Section 4.2.2 if the trial participant will not or cannot discontinue them
- Bariatric surgery
- Intolerable GI symptoms despite management as described in Section 4.1.4.1.
- >3 doses are missed (see Table 6)
- BMI ≤18.5 kg/m^2 is reached at any time during the trial. If a trial participant with any BMI above 18.5 kg/m^2 wants to discontinue the IMP because the trial participant does not want to lose any more weight, the investigator should propose a dose reduction to prevent the discontinuation from IMP treatment, but not lower than 2.4 mg
- Diagnosis of T1DM
- Diagnosis of any malignancy (other than basal or squamous-cell carcinoma of the skin) after randomisation
- Elevation of calcitonin ≥100 pg/L (29.26 pmol/L)
- The trial participant develops a clinically significant elevation of liver enzymes. Refer to Section 5.2.6.1.4.
- Development of any significant IMP related hypersensitivity reaction, as per investigator’s opinion
- Severe HS reaction or anaphylactic reaction (see Section 4.2.1.3)
- Occurrence of any other treatment-emergent AE, SAE, or clinically significant finding for which the investigator believes that permanent IMP discontinuation is the appropriate measure to be taken

Proprietary confidential information © 2023 Boehringer Ingelheim International GmbH or one or more of its affiliated companies

In case a trial participant was mistakenly enrolled in the study (did not meet inclusion criteria or met exclusion criteria at screening/ randomisation), the participant's continuation on the trial treatment will be discussed with the Sponsor Study Physician.

For participants who prematurely discontinue the IMP, all efforts should be made to observe these participants and ask them to continue to attend the scheduled visits until the end of the trial, or at minimum, to conduct the Week 51 visit. However, if a participant who prematurely discontinues the IMP is not willing to return for the predefined trial visits, a remote visit or phone call should be made at Week 51 to obtain vital status. If possible, weight, AEs, and concomitant therapy changes should be recorded.

Every attempt must be made by the investigator to ensure a participant continues participating in the trial during IMP interruptions and after discontinuation of the IMP. Participants that are not actively taking IMP may be less motivated to adhere to the scheduled trial visits. Investigators and site staff should work to detect early signs of losing interest and readily present such participants (not actively taking IMP) with the following options to encourage continued participation:

- Option 1: Continue to attend regularly scheduled trial visits at the centre until the trial ends
- Option 2: Conduct all remaining trial visits via remote visits or over the phone
- Option 3: Discontinue participation in remaining trial activities but permit collection of vital status at the end of the trial through the participant or alternative person designated by the participant (e.g. family, spouse, partner, or physician) even if only by telephone. If possible, other AEs and concomitant therapy changes are to be recorded. Sites should encourage the participant to return to the clinic for the final FU visit.
- Option 4: Discontinue participation in remaining trial activities but permit collection of vital status at the end of the trial through the participant, alternative person designated by the participant, or through review of participant's medical information from alternative sources (e.g. doctor's notes, hospital records, etc.)

Participants will be asked to choose the most rigorous form of follow-up that they are willing to comply with.

In case of a temporary reason, IMP should be restarted if medically justified; please see Section 4.1.4. Any planned temporary discontinuation due to a medical condition requires discussion with the Sponsor Study Physician.

If new efficacy/safety information becomes available, BI will review the benefit-risk assessment and, if needed, pause or discontinue the IMP treatment for all trial participants or take any other appropriate action to guarantee the safety of the trial participants.

3.3.4.2 Withdrawal of consent to trial participation

A participant has the right to withdraw informed consent for participation at any time for any reason. However, withdrawal of consent from trial participation should be very rare and unusual. Because of this, the investigator must be involved in the discussions with the

Proprietary confidential information © 2023 Boehringer Ingelheim International GmbH or one or more of its affiliated companies

participant regarding a withdrawal of consent. Early discontinuation of IMP is not a criterion for withdrawal of consent for participation in the trial.

If the participant withdraws informed consent for participation in the trial, the trial will end for that participant. The participant should stop taking IMP and should be asked to complete the EOT visit and follow-up procedures as described in the SoA. Completing these procedures is strongly recommended for the participant's safety. Participants that withdraw informed consent will not be replaced.

Vital status must be collected at the end of trial for participants who withdraw consent from trial participation, if allowed by local regulations.

3.3.4.3 Discontinuation of the trial by the sponsor

Boehringer Ingelheim reserves the right to discontinue the trial overall or at a particular trial site at any time for the following reasons:

1. Failure to meet expected enrolment goals overall or at a particular trial site
2. New efficacy or safety information invalidating the earlier positive benefit-risk assessment; please see Section 3.3.4.1
3. Deviations from GCP, the trial protocol, or the contract impairing the appropriate conduct of the trial

Further treatment and follow up of trial participants affected will occur as described in Section 3.3.4.1.

The investigator/trial site will be reimbursed for reasonable expenses incurred in case of trial termination (except in case of the third reason).

3.3.4.4 Lost to follow-up

If a participant is lost, every effort will be made by the investigator and site staff to contact and locate the participant before the participant is declared lost to follow-up. Investigators and site staff must use every possible allowable means, according to local regulations, to locate participants who have missed visits. Efforts to contact the participant may include but are not limited to:

- Calling all numbers for participant and listed contacts
- Calling primary care physician, referring specialist and/or other listed physicians for more recent information, date of last office visit or to determine vital status
- Sending an email and follow up with mailing certified letters (return receipt requested) to all known participant addresses and all listed contacts that were provided by the participant
- Reviewing participants records and medical notes for any details of a hospitalisation doctor's visit or other procedure that may indicate location or status of participant
- Use internet to search for possible contact information for the participant
- Try reverse directory for phone numbers to get possible addresses and/or new contact details

Proprietary confidential information © 2023 Boehringer Ingelheim International GmbH or one or more of its affiliated companies

- Check local, regional, and national public records to locate the participant or search for vital status in accordance with local law
- Consider home visit
- Use third party vendor in compliance with local legislation to contact participant

The following actions must be taken if a participant fails to return to the clinic for a required study visit:

- The site must attempt to contact the participant and reschedule the missed visit as soon as possible and counsel the participant on the importance of maintaining the assigned visit schedule and ascertain whether or not the participant wishes to and/or should continue in the trial.

- Before a participant is deemed lost to follow-up, the investigator or designee must make every effort to regain contact with the participant (where possible, 3 telephone calls and, if necessary, a certified letter to the participant's last known mailing address or local equivalent methods). These contact attempts should be documented in the participant's medical record.

- Should the participant continue to be unreachable, they will be considered lost to follow-up.

Proprietary confidential information © 2023 Boehringer Ingelheim International GmbH or one or more of its affiliated companies

4. TREATMENTS

4.1 INVESTIGATIONAL TREATMENTS

4.1.1 Identity of the Investigational Medicinal Products

Table 2 Test product

Substance:	Survodutide
Pharmaceutical formulation:	Solution for injection
Source:	Boehringer Ingelheim Pharma GmbH & Co. KG, Biberach Germany
Unit strength:	Filling volume: 0.5 mL<br>Concentrations: 0.3 mg/0.5 mL, 0.6 mg/0.5 mL, 1.2 mg/0.5 mL, 2.4 mg/0.5 mL, 3.6 mg/0.5 mL, 4.8 mg/0.5 mL, and 6.0 mg/0.5 mL
Posology:	Once weekly (one pre-filled syringe)
Method and route of administration:	s.c. injections

Table 3 Comparator product

Substance:	Placebo
Pharmaceutical formulation:	Solution for injection
Source:	Boehringer Ingelheim Pharma GmbH & Co. KG, Biberach Germany
Unit strength:	Filling volume: 0.5 mL
Posology:	Once weekly (one pre-filled syringe)
Method and route of administration:	s.c. injections

4.1.2 Selection of doses in the trial and dose modifications

Maintenance dose of 6.0 mg survodutide will be investigated. Dose escalation will be performed over 24 weeks after randomisation and will consist of stepwise dose increases every 4 weeks from the starting dose of 0.3 mg and consecutive escalation doses of 0.6, 1.2, 2.4, 3.6, 4.8, and 6.0 mg.

The dosing regimens were selected based on the following considerations:

- Data from trial participants with obesity or overweight up to 6 weeks for doses ranging from 0.7 to 3.15 mg/week (daily dosing) and 0.3 to 3 mg/week (weekly dosing), and later up to 16 weeks for doses ranging from 0.6 to 4.8 mg/week (weekly or twice weekly dosing from trial 1404-0003 [c26759941-01].
- Safety and tolerability of survodutide evaluated in a dose-finding trial 1404-0036 [c31754142-05] in 387 trial participants with overweight or obesity using once weekly injections of 0.6, 2.4, 3.6, and 4.8 mg or placebo.

Proprietary confidential information © 2023 Boehringer Ingelheim International GmbH or one or more of its affiliated companies

- The selected up-titration scheme is more spaced-out (every 4 weeks) compared with earlier studies where the dose was escalated every 2 weeks. This is expected to improve the overall tolerability profile. In particular, simulations with exposure-response models developed based on Phase I and Phase II (data on file) predict decreased frequency of nausea and vomiting with the selected dosing regimen compared with earlier studies, especially during the early dose escalation phase, which is known to be associated with the highest discontinuation rate due to GI tolerability. Additionally, more flexibility has been included (e.g. the dose escalation period in Phase III will be extended up to a maximum of 32 weeks if needed to mitigate GI side effects; participants will have the option of temporary interruption of IMP; they will also be able to reduce the IMP dose to the next lower dose with the possibility to re-escalate to the randomised dose). Planned up-titration scheme and flexibility to interrupt or reduce the dose is expected to improve the overall tolerability profile in Phase III program and enable more trial participants to reach and stay on the target dose.
- The model-predicted average heart rate increase in the 6.0 mg group for this Phase III study is comparable with that simulated for the dose groups tested in the Phase II study 1404-0036 (data on file), where no cardiac safety signals were detected.
- Based on the no observed adverse effect level in male cynomolgus monkeys in a 39-week study, the 6.0 mg dose had a safety margin of 3.03 for $C_{max,ss}$ and 2.98 for $AUC_{\tau,ss}$ (data on file).
- The 6.0 mg dose is expected to enhance the effect of GCGR agonism in lipid and fatty acid oxidation in the liver [c14085752].

4.1.3 Method of assigning trial participants to treatment groups

After the assessment of all inclusion and exclusion criteria, each eligible trial participant will be randomised to a treatment group according to a randomisation plan (see Section 7.4). Randomisation codes will be generated through a validated software and kept blinded to the trial team, sites, and trial participants. Access to the codes will be controlled and documented. An Interactive Response Technology (IRT) system will be used to screen participants, create a participant number, perform treatment assignment, manage initial/re-supply ordering of IMP supplies, and handle emergency unblinding.

Randomisation will be stratified by the presence of T2DM.

In addition, an adjustable screening cap will be implemented in the IRT system to ensure that enough male and female trial participants are enrolled and randomised. Cap for number of female participants is approximately 70% of all randomised participants.

4.1.4 Drug assignment and administration of doses for each trial participant

Dose escalation of survodutide/placebo takes place up to 24 weeks after randomisation as displayed in Table 4 (approximately 4 weeks between each dose escalation; up to a maximum of 32 weeks, if 1 or 2 re-escalation attempts are needed due to GI symptoms). Administration of survodutide/placebo at Week 24 marks the end of the dose escalation period and the start of the maintenance period.

Proprietary confidential information © 2023 Boehringer Ingelheim International GmbH or one or more of its affiliated companies

[redacted]

Participants will receive the IMP until Week 47. Participants will be instructed to inject the IMP once weekly at the same day of the week to the extent possible (±1 day). A weekly reminder for IMP administration will be sent to the trial participants through a digital support platform to allow trial participants to follow their weekly dosing schedule closely. Trial participants who are not willing to use the digital platform should be closely followed up and monitored for compliance by the site.

The injection day may not match the visit day. If the injection date is planned during the in-clinic site visit, the trial participant should not administer the IMP prior to the visit but after the actual visit to allow PK sampling to be done prior to the injection. The first IMP dose administered at Visit 2 should be taken at the site. For all consequent PK visits, the IMP for injections at the site should be taken from the newly dispensed kit.

During the in-clinic visits, the trial participants will receive IMP to cover the time period until the next scheduled visit. Instr[redacted]to the participants, and participants will receive the IFU. [redacted]some countries to support training at the in-clinic visit. Training [redacted]specified in the SoA.

Injections should be administered in the [redacted]pective of meals as described in the IFU.

Participants should record the date, time of all dose administrations in their eDiary.

Participants should aim to reach the randomised maintenance dose. Between Weeks 16 and 32 of treatment, a participant who does not tolerate the randomised maintenance dose once weekly may stay at a lower dose level as described in Table 5. A dose reduction should only be allowed after consultation with a sponsor representative if the management of GI symptoms was not successful and the participant would otherwise discontinue the IMP, and if considered safe to continue the IMP, as per the investigator’s discretion. Thorough documentation of all measures taken to improve tolerability, including counselling on dietary behaviours, symptomatic medication (antiemetic or antidiarrheal), temporary interruption of IMP, and outcomes of the discussion with sponsor representative, is required in a specific eCRF page in case of a dose reduction. For further details, please refer to Sections 4.1.4.1 and 10.1. For trial participants with GI symptoms, the IMP may be dispensed during an unscheduled visit.

Proprietary confidential information © 2023 Boehringer Ingelheim International GmbH or one or more of its affiliated companies

Table 5 Dose reduction scheme (only applicable between Weeks 16 and 32)

**Last dose of survodutide or placebo**	**If not tolerated, reduce the dose to:**	**If not tolerated after re-escalation, reduce the dose to:**
2.4 mg or less	Discontinue IMP	Not applicable
3.6 mg	2.4 mg	2.4 mg (until EOT)
4.8 mg	3.6 mg	3.6 mg (until EOT)
6.0 mg	4.8 mg	4.8 mg (until EOT)

In case 1 or more IMP doses are missed, the instruction in Table 6 should be followed. A missed dose should not affect the dose escalation scheme or scheduled dosing day of the next week. A minimum of 5 days between weekly doses is required.

Table 6 Instructions in case of missed IMP dose(s)

**Number of missed doses**	**What to do**
1 dose of IMP	IMP administration as soon as noticed, but within 2 days after the original scheduled time point<br>If noticed more than 2 days after the original scheduled injection time point, no IMP administration until the next scheduled IMP injection
2 consecutive doses of IMP	Recommencement of IMP treatment if considered safe as per the investigator's discretion and if the participant does not meet any of the discontinuation criteria
3 consecutive doses of IMP	Consideration of treatment discontinuation. If the trial participant is willing to continue the treatment, the sponsor must be contacted to find an alternative option.
>3 consecutive doses of IMP	Treatment discontinuation<br>In some exceptional circumstances (e.g. COVID-19-like pandemic situation), the sponsor is to be contacted for an alternative option.

The residual effect period of survodutide is 28 days (half-life 112 h); therefore, a period of 28 days after last administration of the IMP will be assigned to the on-treatment period for evaluation.

In the event of force majeure or other disrupting circumstances (e.g. pandemic, war; see Section 6), in-clinic visits may not be feasible or may need to be restricted to ensure participant safety. Based on a thorough assessment of the benefits and risks, the investigator may still decide to continue IMP treatment. Where permitted by local law and regulations, the IMP may be shipped directly to the participants' home. In agreement with the sponsor, a home visit service or remote visit may assist the participant with IMP administration.

In addition, home delivery of IMP is allowed upon special request of the trial participant, e.g. due to limited storage space at home. A specialised courier service has been contracted for the direct to participant IMP shipment. If requested, and if allowed by country regulations, this courier service will deliver IMP dispensed by site pharmacy directly to trial participants' home address. The courier service has special procedures in place to protect trial participant privacy data.

Proprietary confidential information © 2023 Boehringer Ingelheim International GmbH or one or more of its affiliated companies

4.1.4.1 Management of gastrointestinal symptoms

Participants who experience intolerable GI symptoms, for example nausea, vomiting, or diarrhoea, at any time during the trial should:

- First be counselled on dietary behaviours that may help mitigate nausea and vomiting. This may include eating smaller meals, splitting 3 daily meals into 4 or more smaller ones, stopping eating when they feel full, reducing fat intake, and not eating for 2-3 h before going to sleep. In case of vomiting or diarrhoea, the participant should be instructed to stay well hydrated.
- If symptoms persist, the participant should be prescribed symptomatic medication (antiemetic or antidiarrheal medication) at the investigator's discretion and kept hydrated.
- A temporary interruption of IMP for 1 dose is permitted.
- A temporary interruption of a second dose of IMP may be allowed at the investigator's discretion.
- The IMP should be resumed at the assigned dose immediately, alone or in combination with symptomatic medication, which can be utilised to manage symptoms.

[redacted]

4.1.5 Blinding and procedures for unblinding

4.1.5.1 Blinding

Trial participants, investigators, central reviewers, and everyone involved in the trial conduct or analysis or with any other interest in this double-blind trial will remain blinded regarding the randomised treatment assignments until the database is declared ready for analysis. Further details regarding the timepoint of unblinding the database for analysis are provided in the TSAP.

Proprietary confidential information © 2023 Boehringer Ingelheim International GmbH or one or more of its affiliated companies

Access to the randomisation code will be kept restricted until its documented release per sponsor SOP. The DMC will be provided with unblinded data to allow them to review safety and to fulfil their tasks as outlined in the DMC charter. An independent team, not otherwise involved in the conduct of the trial, will provide the unblinded results to the DMC.

Randomisation codes will be provided to the external bioanalytical laboratory during the trial to allow PK samples taken from participants receiving placebo / comparator to be excluded from the analyses. In preparation for data transfer and bioanalytical report writing, the trial bioanalyst may receive unblinded data from the external bioanalytical laboratory after the last trial participant has completed the last visit but prior to official unblinding of the analysis database. The trial bioanalyst will not disclose the randomisation codes or bioanalytical results until the trial is unblinded after database lock.

4.1.5.2 Emergency unblinding and breaking the code

Emergency unblinding will be available to the investigator via IRT. It must only be used in an emergency when the identity of the trial drug must be known to the investigator in order to provide appropriate medical treatment or otherwise assure safety of trial participants. The treatment allocation should not be disclosed to the sponsor unless this is explicitly requested. The reason for unblinding must be documented in the source documents and/or appropriate eCRF page.

Due to the requirements to report SUSARs, it may be necessary for a representative from BI's Pharmacovigilance group to access the randomisation code for individual trial participants during trial conduct. The access to the code will only be given to authorised PSPV representatives for processing in the Pharmacovigilance database and not be shared further.

4.1.6 Packaging, labelling, and re-supply

The IMP will be provided by Boehringer Ingelheim or a designated Contract Research Organisation (CRO). The IMP will be packaged and labelled in accordance with the principles of Good Manufacturing Practice (GMP). Re-supply to the sites will be managed via an IRT system, which will also monitor expiry dates of supplies available at the sites.

The label will be prepared according to EU Regulation No. 536/2014, Annex 6, Section D.8., omitting certain particulars with the following justifications:

- The investigator name was omitted from the label due to the IRT system.
- Should local regulations outside EU require these particulars they will be added to the country specific label text.

For details of packaging and the description of the label, refer to the ISF.

4.1.7 Storage conditions

IMP supplies will be kept in their original packaging to protect them from light, in a secure limited access storage area, and refrigerated according to the recommended storage conditions on the IMP label. A temperature log must be maintained for documentation.

If the storage conditions are found to be outside the specified range, the CRA (as provided in the list of contacts) must be contacted immediately.

Proprietary confidential information © 2023 Boehringer Ingelheim International GmbH or one or more of its affiliated companies

The IMP will be provided in pre-filled syringes at specific visits (see SoA). An IFU with step-by-step instructions on how to use the pre-filled syringe will be made available to the investigator. A copy will be placed in the ISF. The trial participant will also receive a copy of the IFU when pre-filled syringes are dispensed to the participant for self-administration. Moreover, the IFU will contain important safety information and storage instructions.

4.1.8 Drug accountability

The investigator or designee will receive the IMP delivered by the sponsor or delegate when the following requirements are fulfilled:

- Approval of the clinical trial protocol by the IRB / ethics committee
- Availability of a signed and dated clinical trial contract between the sponsor or delegate and the investigational site
- Approval / notification of the regulatory authority, e.g. competent authority
- Availability of the *curriculum vitae* of the Principal Investigator
- Availability of a signed and dated clinical trial protocol
- Availability of the proof of a medical license for the Principal Investigator
- Availability of FDA Form 1572 (if applicable)

The IMP is not allowed to be used outside the context of this protocol. It must not be forwarded to other investigators or clinics, except if specifically authorised by the sponsor.

Trial participants should be instructed to bring all unused IMP, empty cartons, and sharps container with used syringes with them to their next in-clinic visit (scheduled and unscheduled).

The investigator or designee must maintain records of the product's delivery to the trial site, the inventory at the site, the use by each trial participant, and the return to the sponsor or warehouse/drug distribution centre or alternative disposal of unused products using the IRT. If applicable, the sponsor or warehouse/drug distribution centre will maintain records of the disposal.

These records via IRT will include dates, quantities, batch/serial numbers, expiry ('use by') dates, and the unique code numbers assigned to the IMP and trial participants. The investigator or designee will maintain records that document adequately that the trial participants were provided the doses specified by the clinical trial protocol and reconcile all IMPs received from the sponsor. At the time of return to the sponsor or appointed CRO, the investigator or designee must verify that all unused or partially used IMP supplies have been returned by the trial participant and that no remaining supplies are in the investigator's possession.

Proprietary confidential information © 2023 Boehringer Ingelheim International GmbH or one or more of its affiliated companies

4.2 OTHER TREATMENTS, EMERGENCY PROCEDURES, RESTRICTIONS

4.2.1 Other treatments and emergency procedures

4.2.1.1 Concomitant medications

During the trial, participants are expected to be treated according to the best standard of care for baseline medical conditions. Investigators will be asked to confirm in the eCRF during each visit if this condition is met.

As survodutide is expected to improve certain obesity-related complications (e.g. lipid levels, blood pressure, HbA_{1c}), the evaluation of existing treatment should be done during each in-clinic visit and eventual adjustment of treatment should be recorded in eCRF. All concomitant medications, including anti-hyperglycaemia medications (see Section 4.2.2.1), should be recorded on the appropriate pages of the eCRF. For the management of trial participants with GI symptoms, refer to Section 4.1.4.1.

Participants who develop diabetes during the trial may initiate medication for glucose control (see Section 4.2.2.1). They will be provided and trained to use a glucometer and provided an eDiary to record hypoglycaemic and hyperglycaemic episodes.

4.2.1.1.1 Home glucose monitoring

All participants with diabetes mellitus will be provided with a glucose monitoring device and supplies for use at home during the trial for self-measurement of blood glucose. Instruction on the use of the glucose monitoring device will be provided by the site staff. Trial participants may also use their own glucose monitoring device.

Weekly home measurements are recommended. Additionally, throughout the trial, blood glucose measurements should be performed at any time the participant is symptomatic, i.e. experiences signs/symptoms of hyper- or hypoglycaemia. More frequent testing may be done if deemed necessary by the investigator or required by local authorities.

Blood glucose measurements should be taken fasted (at least 8 h) and prior to taking any anti-hyperglycaemic medication.

At the time of signing consent, all trial participants with T2DM will be educated about signs and symptoms of both hyper- and hypoglycaemia, how to treat both conditions, and how to collect information for each episode of hyper- or hypoglycaemia in the eDiary. Both conditions may be identified by spontaneous reporting of symptoms from trial participants, by plasma glucose samples collected during in-clinic visits, or by home glucose monitoring device.

4.2.1.1.2 Rescue medication for treatment of hyperglycaemia

Hyperglycaemia should be identified in case of persistent and unacceptable hyperglycaemia, if any of the FPG values (including fasting SMBG values) exceeds 15 mmol/L (270 mg/dL) and no intercurrent cause of the hyperglycaemia can be confirmed. In such cases, glycaemic rescue treatment should be initiated at the discretion of the investigator.

Proprietary confidential information © 2023 Boehringer Ingelheim International GmbH or one or more of its affiliated companies

- If any of the FPG values (including fasting SMBG values) exceeds 15 mmol/L (270 mg/dL), a confirmatory FPG and HbA_{1c} value (at the central laboratory) should be obtained by calling the trial participant for an unscheduled visit.
- If the confirmatory measurement exceeds 15 mmol/L (270 mg/dL), the trial participant should be offered rescue medication, at the discretion of the investigator, according to the ADA/EASD guidelines [P22-05567] (excluding prohibited medications as stated in Section 4.2.2.1)

The following scenarios can be foreseen within the rescue treatment start and should be documented in the eCRF as rescue medications:

- Additional anti-hyperglycaemia medication used for 7 days or more or until treatment discontinuation
- Increase in the dose of background medication above the baseline dose for 7 days or more or until treatment discontinuation

If the trial participant does not have persistent and unacceptable hyperglycaemia >15 mmol/L (270 mg/dL) but the investigator deems necessary to adjust the anti-hyperglycaemia medications, these changes will not be considered as rescue therapy but as the therapy adjustment. Therapy adjustment has to be documented on the respective pages of the eCRF.

Trial participants that are started on rescue medication should continue to follow the protocol-specified visit schedule and stay on randomised treatment unless the investigator judges that it jeopardizes safety.

Rescue medication should be documented in medical records and reported in the eCRF. Rescue medication will not be supplied but reimbursed as long as the trial participant remains in the trial, if required according to local regulations.

4.2.1.1.3 Hypoglycaemic episodes

When a trial participant experiences a hypoglycaemic episode, the participant should call the site and record the general information in relation to the hypoglycaemia (date, time, blood glucose measurements, and any symptoms) in the eDiary. Blood glucose should always be measured and recorded when a hypoglycaemic episode is suspected.

Hypoglycaemic events will be recorded in the eCRF using the definitions as stated in Section 5.2.6.1.4. They should be treated and additional glucose monitoring should be implemented per investigator discretion and medical judgement.

All plasma glucose values ≤3.9 mmol/L (70 mg/dL, with or without hypoglycaemic symptoms) should be reported as a hypoglycaemic episode.

4.2.1.2 Dietary and physical activity counselling

Participants will receive diet and physical activity counselling.

Participants will be given a paper Diary in which they will track their level of activity daily as well as their daily dietary compliance in order to assist and evaluate their lifestyle intervention (alternatively local apps can be used if already in use by participant prior to

Proprietary confidential information © 2023 Boehringer Ingelheim International GmbH or one or more of its affiliated companies

joining the study). The participant dietary compliance and the average daily level of physical activity will be evaluated and recorded in the eCRF at every visit by the site staff.

All participants will receive dietary guidance materials. At the randomisation visit, participants will receive diet counselling by a dietician or equivalent qualified delegate according to local standards with a recommended energy deficit of approximately 500 kcal/day compared with the participant's estimated total energy expenditure [R20-2315].

The total energy expenditure is calculated at the randomisation visit by multiplying the estimated basal metabolic rate (Table 7) with a physical activity level value of 1.3. The hypocaloric diet is to be continued after randomisation and throughout the planned treatment period. If a BMI of ≤22 kg/m^2 is reached, the recommended energy intake should be recalculated with no kcal deficit (maintenance diet) for the remainder of the trial.

Table 7 Equations for estimating basal metabolic rate

**Sex**	**Age [years]**	**Basal metabolic rate [kcal/day]**
Men	18-30	15.057 × actual weight in kg + 692.2
	31-60	11.472 × actual weight in kg + 873.1
	>60	11.711 × actual weight in kg + 587.7
Women	18-30	14.818 × actual weight in kg + 486.6
	31-60	8.126 × actual weight in kg + 845.6
	>60	9.082 × actual weight in kg + 658.5

An increase in physical activity to at least 150 to 300 min a week of moderate intensity, combining aerobic and muscle strengthening exercises, will be encouraged and re-enforced.

4.2.1.3 Severe and/or systemic hypersensitivity including injection reaction and anaphylactic reaction

In case of severe and/or systemic hypersensitivity including anaphylactic reaction emerging during or after injection(s) of the IMP, the investigator should consider in accordance with the severity of the reaction and local standard of care to immediately stop further injections and treat with systemic antihistamines, i.v. steroids, and in case of a severe allergic reaction (e.g. anaphylactic reaction) with epinephrine.

In case of systemic hypersensitivity, based on the trial participant's clinical course and medical judgment, the injection(s) may be continued in case of mild or moderate systemic hypersensitivity.

In case of an anaphylactic reaction based on the criteria discussed in the statement paper by Sampson HA *et al.* [R11-4890] suspected to be caused by the IMP, the investigator should permanently discontinue the treatment with IMP.

The following laboratory tests should be performed:

- A blood sample should be drawn for immunoglobulin E, PK, ADA, and NAb assessment as detailed in the Lab Manual (ISF).
- In addition, the evaluation of histamine, serum tryptase, and complement components (C3 and C4) should be included.

Proprietary confidential information © 2023 Boehringer Ingelheim International GmbH or one or more of its affiliated companies

4.2.1.4 Elective surgery/procedures that require general anaesthesia or deep sedation

For trial participants who undergo elective surgery/procedures that require general anaesthesia or deep sedation, the following recommendations are made based on the American Society of Anaesthesiologists Consensus-Based Guidance on Preoperative Management of Patients (Adults and Children) on GLP-1R agonists [R23-2688]:

- Consider holding the IMP one week prior to elective surgery/procedure that requires general anaesthesia or deep sedation
- On the day of the procedure, if GI symptoms such as severe nausea/vomiting/ retching, abdominal bloating, or abdominal pain are present, consider delaying elective procedure
- On the day of the procedure, if the patient has no GI symptoms, and the IMP has been held as advised, proceed as usual.

4.2.2 Restrictions

4.2.2.1 Restrictions regarding concomitant treatment

While enrolled in this trial, trial participants must not participate in another investigational drug or device trial or receive other investigational treatment(s).

<u>GLP-1R agonists</u>

GLP-1R agonists including GLP-1R agonist/insulin/GIP combinations are not allowed 3 months before screening visit and during the trial period from screening until end of follow-up. If such treatment is initiated during the trial, the participant should be instructed to discontinue the treatment with GLP-1R agonists including GLP-1R agonist/insulin/GIP combinations, and if the treatment is discontinued immediately, the participant should remain in the trial. If no discontinuation of such a drug is possible, the participant needs to stop IMP administration.

<u>NASH medications</u>

Initiation or change in dose of Vitamin E exceeding 400 UI per day is prohibited 6 months prior to randomization and during the entire duration of the trial.

Any off label, investigational (or subsequently approved) medications used for the purpose of treatment of NAFLD/NASH (including but not limited to GLP-1R agonists) are prohibited 6 months prior to the trial and for the trial duration up to EOS visit.

Treatment with drugs that may cause <u>hepatic steatosis or steatohepatitis</u> administered for at least 2 weeks within 12 months prior to screening is prohibited (e.g. valproic acid, tamoxifen, methotrexate, amiodarone, chronic treatment with oral corticosteroids >5 mg/day of prednisone equivalent [one short [<2 weeks] course of oral corticosteroids more than 3 months before screening is allowed], or oestrogens at doses greater than those used for contraception or hormone replacement).

<u>Anti-obesity medications</u>

All anti-obesity medications including bupropion/naltrexone, orlistat, phentermine, and phentermine/topiramate are not allowed 3 months before screening and during the whole trial

Proprietary confidential information © 2023 Boehringer Ingelheim International GmbH or one or more of its affiliated companies

period (screening, treatment, and follow-up periods including trial participants who discontinue the IMP).

If such treatment is initiated during the trial, the participant should be instructed to discontinue the treatment, and if the treatment is discontinued, the participant should remain in the trial. If no discontinuation of such a drug is possible, the participant needs to stop IMP administration.

Body weight change inducing medication

Initiation of treatment with medications that may cause body weight change is not allowed 3 months before screening and during the trial (Table 8). Participants who were taking these medications for >3 months prior to screening with stable body weight are allowed to participate in the trial. If it is necessary to start such a treatment during the trial, alternative medications without potential to change body weight should be considered. In case there is no alternative possible, the sponsor should be contacted. If possible, the same approach should be applied to participants who discontinued the IMP and are in the follow-up period.

Table 8 Body weight change inducing medication

**Medication class**	**Weight gain**	**Weight loss**
Anti-hypertensives	α-adrenergic blockers (doxazosin, prazosin, terazosin)<br>β-blockers (atenolol, metoprolol, nadolol, propranolol)	
Anti-depressants	TCA (amitriptyline, doxepin, imipramine, nortriptyline), MAOIs, SSRIs (mirtazapine, paroxetine), lithium	Bupropion, fluoxetine, triple reuptake inhibitors (tesofensine)
Anti-psychotics	FGA (thorazine, thioridazine, haldol, fluphenazine), SGA (clozapine, olanzapine, risperidone, asenapine)	
Anti-epileptics	Carbamazepine, gabapentin, pregabalin, valproic acid	Topiramate, zonisamide
Anti-histamines[1]	First generation antihistamines (diphenhydramine)	
Systemic steroids[1]	Glucocorticoids (dexamethasone, methylprednisolone, prednisolone, prednisone)	
Opioid antagonists		Naltrexone

[1]Short use up to 14 days is allowed. If longer use is needed, the sponsor should be contacted.

Anti-hyperglycaemia medications

Only trial participants who develop T2DM during the trial may initiate anti-hyperglycaemia medications for glucose control, with the exception of GLP-1R agonists, GLP-1R agonist/ GIP combinations, amylin analogues, and DPP-4i (Table 9).

During the trial the following medication for the treatment of diabetes is allowed as per local guideline: metformin, SGLT2i, thiazolidinediones, sulfonylurea, insulin or acarbose (Table 9). Metformin should not be initiated during the trial for the treatment of other metabolic conditions (e.g. polycystic ovary syndrome, diabetes prevention). For participants on pioglitazone, the dose should be stable for 6 months before enrolment. For participants on stable sulfonylurea or insulin treatment, it is recommended to lower the dose when starting the IMP treatment to avoid hypoglycaemic events. All concomitant medications should be recorded on the appropriate pages of the eCRF.

Proprietary confidential information © 2023 Boehringer Ingelheim International GmbH or one or more of its affiliated companies

Table 9 Anti-hyperglycaemia medications

**Allowed medication**	**Prohibited medication**
Metformin	GLP-1R agonists
SGLT2i	GLP-1R agonist/GIP combinations
Thiazolidinediones[1]	Amylin analogues
Sulfonylureas[2]	DPP-4i
Insulin[3]	
Acarbose	

[1] For participants on pioglitazone use, the dose should be stable for 6 months before enrolment

[2] Allowed for participants on stable treatment (i.e. for ≥3 months prior to screening), but not allowed for treatment initiation during screening and the whole trial period

[3] HOMA-IR collection and evaluation should not be performed in subjects on current insulin therapy.

Oral medications with a narrow therapeutic index
Due to the effect of survodutide on gastric emptying which may affect the absorption of orally administered drugs, increased clinical or laboratory monitoring for orally administered co-medications that have a narrow therapeutic index is recommended, and if not possible, drugs with NTIs are not permitted during treatment with survodutide.

CYP3A sensitive medications with a narrow therapeutic index
As survodutide has the potential to down regulate CYP3A4 expression, which might affect safety and efficacy of CYP3A sensitive drug substances with a narrow therapeutic index, all CYP3A sensitive medications with NTIs (e.g. alfentanil, fentanyl, carbamazepine, cyclosporine, tacrolimus, sirolimus) are not permitted during the trial.

Oral contraceptives
Due to the effect of survodutide on gastric emptying which may affect the absorption of orally administered drugs and the effect of oral contraceptives may no longer be guaranteed, increased precautions are recommended. Women who use oral contraceptives at screening should be advised to change to non-oral contraceptives at least 7 days prior to first dose of IMP or to add additional barrier method of contraception during the entire treatment period with IMP and 28 days thereafter (see Section 4.2.2.3).

4.2.2.2 Restrictions on diet and lifestyle

Please refer to Section 4.2.1.2.

4.2.2.3 Contraception requirements

Contraception of male participants is not needed.

WOCBP (for the definition please refer to Section 3.3.2) must use a medically approved method of birth control throughout the trial and for a period of at least 28 days after last IMP intake. A list of contraception methods meeting these criteria is provided in the trial participant information and Section 10.2.

Proprietary confidential information © 2023 Boehringer Ingelheim International GmbH or one or more of its affiliated companies

4.3 TREATMENT COMPLIANCE

Trial participants will be provided with an eDiary to record all the injections of the IMP (at home or at the clinic during in-clinic visits).

Trial participants should be encouraged to be fully compliant with the medication dosing schedule. A weekly reminder for recording the date and time of IMP administration will be sent to the trial participants through a digital support platform to allow trial participants to follow closely their weekly dosing schedule. Trial participants who are not willing to use the digital platform should be closely followed up and monitored for compliance by the site.

Trial participants should be compliant on clinic visits within the protocol allowed time window and on the dosing schedule when they self-administer the IMP at home with pre-filled syringes. An IFU with instructions on how to use the pre-filled syringes including important safety information and storage instructions will be provided to the investigators and trial participants.

A compliance check of the IMP administration will be performed at each clinic visit to ensure the weekly injection is being administered correctly at both the trial site and by the trial participant at home. In case of missed doses, the site should instruct the trial participant as indicated in Section 4.1.4. The investigator/site staff should explain to the trial participant the importance of treatment compliance and discuss with the trial participant any tolerability issue.

Trial participants will be provided with a medication bag to store empty cartons of the IMP and a sharp bin to store all used syringes. Unused IMP should be kept in the refrigerator. Trial participants are requested to bring all unused IMP, empty cartons, and sharps container with used syringes with them to their next in-clinic visit (scheduled and unscheduled) for the compliance check. The sharps container with used syringes should be returned to the site. Any discrepancies should be discussed with the investigator or the designee.

Proprietary confidential information © 2023 Boehringer Ingelheim International GmbH or one or more of its affiliated companies

5. ASSESSMENTS

5.1 ASSESSMENT OF EFFICACY

5.1.1 MRI

MRI will be used to assess liver fat content using PDFF, liver stiffness using MRE, fibro-inflammation using cT1, and liver volume. To participate in the trial, sites must have an MRI scanner and MRE acoustic driver from one of the following manufacturers: GE, Philips, or Siemens, and have access to manufacturer-specific MRE sequences. Ideally, the MRI scanner should be equipped with a manufacturer-specific MRI-PDFF package, also known as multi-echo, multi-peak Dixon techniques. The cT1 analysis requires proprietary algorithms that must be installed on the site MRI device by the patent holder (Perspectum) using their software package "LiverMultiScan®." All imaging sites will undergo a quality assessment process before the trial begins, which includes a review of their MRI hardware and software. Sites may collaborate with other sites that meet these requirements.

To calculate the liver fat volume, MR images will be collected from each participant during a single MRI session, utilising 3D image acquisition to determine the complete liver volume. Other MR image sequences may also be collected for exploratory analyses if required. The MR image acquisition process, including preparation time, is estimated to take no longer than 30 min. All MR images will be sent to an external vendor for centralised reading.

5.1.2 Fibroscan®

Liver stiffness and steatosis will be assessed using the FibroScan® device (Echosens). Liver stiffness is a measurement of fibrosis and will be assessed using VCTE™. The liver steatosis will be assessed using CAP™. Both measurements are performed using a standard FibroScan® device using the patient specific ultrasound probes which are tailored to the morphology of the patient.

5.1.3 Body measurements

For time points, refer to the SoA.

5.1.3.1 Weight measurement

It is important to minimise the variability in body weight measurement. The sponsor will provide medically graded weight scales to the sites which must be used throughout the course of the trial for all measurements. The equipment will be certified and calibrated according to manufacturer's specifications. Weight should be recorded in kilograms up to 1 decimal.

The body weight must be measured in fasting state (except the screening visit). If the trial participant is not fasted, the participant should be called in for a new visit within the visit window. In order to get comparable body weight values, shoes, coats/jackets, and any headgear should be taken off, and pockets should be emptied of heavy objects (i.e. keys, coins, etc.). Headgear worn for religious reasons are acceptable, but this should be consistently worn for all weight measurements in the trial. The trial participant should empty the bladder before weight is measured.

Proprietary confidential information © 2023 Boehringer Ingelheim International GmbH or one or more of its affiliated companies

5.1.3.2 Height

Height should be measured without shoes in centimetres.

5.1.3.3 Body mass index

BMI will be calculated by the eCRF from the height and weight measured at the time points specified in the SoA.

5.1.3.4 Waist and hip circumference measurement

Waist circumference measurements should be made around the bare midriff, i.e. the abdominal circumference located midway between the lower rib margin and the iliac crest. Hip circumference measurements should be taken around the widest portion of the buttocks without thick clothes (e.g. coats, jackets). Measures must be obtained in standing position with a non-stretchable measuring tape and to the nearest centimetre or inch. The tape should touch the skin but not compress soft tissue, and twists in the tape should be avoided. The trial participant should be asked to breathe normally. The same measuring tape should be used throughout the trial. The measuring tape will be provided by the sponsor.

5.1.4 Blood pressure measurements

Vital signs will be evaluated at the time points specified in the SoA, prior to blood sampling.

Initially, blood pressure should be taken in both arms. If the pressures differ by more than 10 mmHg (as in the presence of a subclavian steal syndrome), the arm with the highest pressure (either systolic or diastolic) should be used for all subsequent measurements.

Blood pressure measurements should be performed on the same arm. The same method and device must be used throughout the trial for a participant. After participants have rested quietly in the seated position for 5 min, 3 blood pressure measurements will be taken 2 min apart, and all 3 results will be entered in the CRF. The seated pulse rate will be taken during the 2-minute interval between the second and third blood pressure reading. Blood pressure measurements can be repeated at the discretion of the investigator, if the values are out of normal range. Caffeine, inhalation of nicotine, and physical activity should be avoided within 30 min prior to the blood pressure measurement.

[redacted]

Proprietary confidential information © 2023 Boehringer Ingelheim International GmbH or one or more of its affiliated companies

[redacted]

5.1.6 Efficacy laboratory parameters

Some of the lab parameters will be analysed as part of efficacy criteria (see Sections 2.1.3, 2.2.2, 5.2.3, and 5.4.1).

5.2 ASSESSMENT OF SAFETY

Safety is monitored during the trial conduct as per the below procedures. Investigators are encouraged to repeat ECG and/or additional imaging and laboratory testing if any clinically significant abnormal finding is observed as required.

5.2.1 Physical examination

A complete physical examination will be performed at the time points specified in the SoA, including at a minimum general appearance, neck (including thyroid gland), lungs, cardiovascular system, abdomen, extremities, and skin. The physical examination should be repeated if the baseline visit (Visit 2) is performed more than 2 weeks after the screening visit.

The results must be included in the source documents available at the site.

Proprietary confidential information © 2023 Boehringer Ingelheim International GmbH or one or more of its affiliated companies

5.2.2 Vital signs

Vital signs will be evaluated at the time points specified in the SoA, prior to blood sampling. This includes SBP, DBP, and pulse rate (electronically or by palpation count for 1 minute) in a seated position after at least 5 min of rest. See also Section 5.1.4.

5.2.3 Safety laboratory parameters

Safety laboratory parameters to be assessed are listed in Table 10, and the sampling time points are shown in the SoA.

The total amount of blood collected during this study will not exceed 570 mL (39 tablespoons), including optional biobanking.

All analyses will be performed by a central laboratory, and the respective reference ranges will be provided in the ISF. Instructions regarding sample collection, sample handling/processing, and sample shipping are provided in the laboratory manual.

All in-clinic visits should be on fasting state (except for screening). Trial participants should be instructed to come in the fasting state after at least 8 h without food or liquid intake, except water. The IMP dose (if applicable) should be withheld in the morning of the clinic visit until blood sampling has been performed. If a trial participant has come to the in-clinic visit in non-fasting state, procedures that require fasting (blood sampling for safety, FPG, FPI, fasting C-peptide, lipids) should be performed on another day within the visit window.

The central laboratory will send reports and alerts to the investigator. It is the responsibility of the investigator to evaluate the laboratory reports. Clinically relevant abnormal findings as judged by the investigator will be reported as AEs (refer to Section 5.2.6).

In case the criteria for hepatic injury are fulfilled, a number of additional measures will be performed as per the DILI Checklist (Section 5.2.6.1.4). The amount of blood taken from the trial participant concerned will be increased due to this additional sampling. The investigator will use the exclusionary lab test results for the assessment of the participants' eligibility for the trial.

The central laboratory will transfer the results of the analysis to the sponsor periodically.

Proprietary confidential information © 2023 Boehringer Ingelheim International GmbH or one or more of its affiliated companies

Table 10 Laboratory tests

**Category**	**Test name**
Safety laboratory test Haematology	Haematocrit<br>Haemoglobin<br>Reticulocyte count (reflex test if haemoglobin outside normal range)<br>Erythrocytes (including morphology)<br>Leukocytes<br>Thrombocytes<br>Neutrophils<br>Eosinophils<br>Basophils<br>Monocytes<br>Lymphocytes<br>INR
Safety laboratory test Clinical chemistry	Albumin<br>Alkaline phosphatase<br>Amylase<br>ALT<br>AST<br>Bicarbonate<br>Bilirubin total, fractionated if increased<br>Calcium<br>Calcitonin<br>Chloride<br>Creatine kinase (CK)<br>CK-MB, troponin (reflex tests if CK is elevated)<br>eGFR (CKD-EPIcr equation for adults)<br>Enzymatic creatinine<br>Gamma-glutamyl transferase<br>hsCRP<br>Lactate dehydrogenase<br>Lipase<br>Magnesium<br>Phosphate<br>Potassium<br>Protein total<br>Sodium<br>Urea (blood urea nitrogen)<br>Uric acid<br>TSH
Glucose metabolism	Fasting plasma glucose (FPG)<br>Fasting Insulin (FPI)<br>HbA_{1c}<br>C-peptide
Lipids	Cholesterol<br>HDL<br>LDL<br>VLDL<br>Triglycerides<br>Free fatty acids<br>ApoB100

Proprietary confidential information © 2023 Boehringer Ingelheim International GmbH or one or more of its affiliated companies

Table 10 (cont'd) Laboratory tests

Category	Test name
Menopausal testing	FSH: for women age >40 years with amenorrhea for 12 months (screening only)
Pregnancy testing	Serum and urine human chorionic gonadotropin (hCG) tests will be performed at screening for women of childbearing potential.<br>Urine hCG test will be performed at all other in-clinic visits using tests provided by the central laboratory. If the urine test is positive, repeat with a serum test.
Urinalysis	Semiquantitative analyses: nitrite, protein, ketone, pH value, leukocyte esterase, erythrocytes<br>Quantitative analyses: urinary albumin, urinary creatinine, [redacted]<br>Urine sample should be collected void in the clinic from the second morning (preferably) and if not possible, from spot urine.
Severe and/or systemic hypersensitivity:	Tryptase<br>Complement C3<br>Complement C4
Infectious serology	Hepatitis B surface antigen (screening only)<br>Hepatitis C antibodies (screening only)<br>HCV RNA (screening only)[1]<br>HIV-1/2 combination (screening only)

[1]Will only be performed in case of Hepatitis C antibodies positivity or history of hepatitis C treatment.

5.2.4 Electrocardiogram

12-lead ECGs should be collected prior to the dosing at the time points specified in the SoA.

At screening, a triplicate ECG will be collected. Centralised ECG services will be provided by an external vendor. ECGs should be collected according to the trial-specific recommendations, using the standardised equipment provided by the vendor. ECGs may be repeated for quality or safety reasons. Trial participants must be supine for approximately 5 to 10 min before ECG collection. Trial participants should remain supine, but awake, during the ECG collection process.

The investigator has the responsibility to complete an initial review as soon as the ECG recordings are obtained at the site visit. At any time during the trial, the investigator may decide to place a hold on further dosing of the trial participant if there is an indication of significant abnormalities in the ECG and may prefer to wait until the results from the central reading are available.

The digital ECG recordings will be transmitted to the vendor for central reading. The ECG recordings will be centrally evaluated and rated as normal, abnormal, or unable to evaluate, and the results will be reported to the site. The site investigator must review the report. If the ECG is rated as abnormal, the investigator will have to determine if the abnormal findings are clinically significant. Clinically significant abnormal findings based on central reading will be reported either as baseline condition (if identified at the screening visit) or otherwise as AEs and will be followed up and/or treated as medically appropriate.

After the screening visit, the investigator must complete a review of the ECG results from central reading. Any pre-existing conditions should be recorded as baseline conditions. ECGs recorded at the randomisation visit should be evaluated by the investigator before the trial participant receives the first dose. If abnormalities are observed by the investigator in the ECG reading at the randomisation visit, it is recommended that the investigator waits until

Proprietary confidential information © 2023 Boehringer Ingelheim International GmbH or one or more of its affiliated companies

the results from central reading are available, and the randomisation visit should be rescheduled.

The site investigator will have the responsibility of following up with the trial participant if there are any clinically significant findings in the ECG report. It is recommended to repeat the 12-lead ECG recording and/or refer to a cardiologist and/or perform additional cardiac tests (i.e. cardiac enzymes) if there is an indication of significant abnormalities or in case of doubts.

QTcF will be applied as the QT correction method.

If a clinically relevant increase (according to investigator judgement) in the QTcF interval or any other clinically significant quantitative or qualitative change from last ECG recording performed prior to the first IMP administration is identified after the trial participant is randomised, the investigator will assess the symptoms (e.g. palpitations, near syncope, and syncope) and decide if the participant will continue taking the IMP. In case automated QTcF on local ECG interval is informed as >500 ms, IMP should be stopped and then, discontinued, if central ECG reading confirms this finding (see also Section 3.3.4.1). Any new findings or deterioration of previous findings observed during the trial, assessed by investigator as clinically significant, will be recorded as AEs/SAEs and will be followed up and/or treated as medically appropriate.

All ECGs that are read in the central location will be stored in the vendor's database and transmitted to the sponsor periodically. If there is any clinically significant finding in an ECG performed locally due to presence of acute symptoms, this ECG will be transmitted to the vendor for central reading.

5.2.5 Other safety parameters

5.2.5.1 Mental health questionnaires

The mental health status of the trial participants will be assessed by the C-SSRS and PHQ-9 questionnaires. The questionnaires will be administered to all trial participants (electronically) in local language (linguistically validated version). Details to the (electronic) use can be found in the ISF.

The C-SSRS is a semi-structured, investigator-rated interview, developed by clinical experts in cooperation with the FDA and requested by regulatory agencies, assessing both suicidal behaviour and suicidal ideation. It does not give a global score but provides some categorical and some severity information specifically for behaviour and ideation.

The C-SSRS interview may be administered by any type of physician, psychologist, clinical social worker, mental health counsellor, nurse, or research coordinator with C-SSRS training. It has a typical duration of 5 min and causes only a low burden on trial participants. At a minimum, the interview consists of 2 screening questions related to suicidal ideation and 4 related to suicidal behaviour and may be expanded to up to 17 items in case of positive responses.

The C-SSRS has been widely used in large multinational clinical trials. The C-SSRS will be administered at the screening visit (using the "baseline/screening" version) with the aim to exclude trial participants with a history of suicidal ideation and behaviour. After the baseline

Proprietary confidential information © 2023 Boehringer Ingelheim International GmbH or one or more of its affiliated companies

visit, the assessment "since last visit" will be performed at clinic and remote visits (if allowed per local regulations) as specified in the SoA.

The investigator will review positive and negative reports for plausibility and clinical relevance. Doubtful reports may be repeated, or reports may be validated by a consulting psychiatrist. If there is an answer "yes" to either Question 4 (Active Suicidal Ideation with Some Intent to Act, Without Specific Plan) on the "Suicidal Ideation" portion of the C-SSRS, or an answer "yes" to Question 5 (Active Suicidal Ideation with Specific Plan and Intent) on the "Suicidal Ideation" portion of the C-SSRS, or an answer "yes" to any of the suicide-related behaviours (Actual attempt, Interrupted attempt, Aborted attempt, Preparatory act or behaviour) on the "Suicidal Behaviour" portion of the C-SSRS, the investigator has to immediately interview the trial participant during the clinic visit and recommended the participant to consult with an MHP. Treatment with the IMP should be stopped, and the trial participant should remain in the trial. Additionally, all C-SSRS reports of suicidal ideation type 4 or 5 and all reports of suicidal behaviour must be reported as separate SAEs by the investigator.

For 'Self-injurious behaviour, no suicidal intent' (Type 11), standard AE/SAE reporting rules are to be applied. For each negative report (suicidal ideation Types 1, 2, or 3) after start of the trial, the investigator will decide based on clinical judgment whether it represents an AE as defined in this protocol, and if it is considered an AE then it must be reported accordingly.

The PHQ-9 is the 9-item depression module of the patient health questionnaire, which is a self-administered diagnostic tool used for assessment of mental disorders. It takes approximately 5 to 10 min to complete the questionnaire and it should be completed together with the other PROs. If the PHQ-9 score is >15, the trial participants should be referred to an MHP to assist in deciding whether the participant may continue in the trial or should be discontinued.

The C-SSRS and the PHQ-9 will be administered to all trial participants electronically where the digital approach is approved. At sites where these cannot be administered electronically, a paper back up will be available.

5.2.5.2 Eye examination for participants with T2DM

Uncontrolled and potentially unstable diabetic retinopathy or maculopathy is defined as an exclusion criterion (Section 3.3, exclusion criterion 13) and a trial participant presenting these conditions during screening is not eligible to participate in the trial. Testing is required via one of these options prior to participant randomisation within the screening period or if such an examination has been performed within the past 3 months prior to screening, the results can be used for eligibility evaluation:

- The pharmacologically pupil-dilated fundus eye examination performed by an ophthalmologist or an equally qualified healthcare provider (e.g. optometrist) OR
- Other ophthalmologist examination of diabetic retinopathy (as recommended by country T2DM management guidelines).

During the trial conduct, the eye examination needs to be performed at time points specified in the SoA or whenever the trial participant has experienced a worsening of vision.

Respective findings will be reported as AEs (refer to Section 5.2.6).

Proprietary confidential information © 2023 Boehringer Ingelheim International GmbH or one or more of its affiliated companies

5.2.6 Assessment of adverse events

Data and information necessary for the thorough assessment of AEs, SAEs, and AESIs will be reported to the sponsor via eCRF. This may include specific data and information not prospectively specified in this protocol.

5.2.6.1 Definitions of AEs

5.2.6.1.1 Adverse event

An AE is defined as any untoward medical occurrence in a trial participant or clinical investigation subject administered a medicinal product and which does not necessarily have to have a causal relationship with this treatment.

An AE can therefore be any unfavourable and unintended sign (including an abnormal laboratory finding), symptom, or disease temporally associated with the use of a medicinal product, whether considered related or not.

The following should also be recorded on the appropriate eCRF(s):

- Worsening of the underlying disease or of other pre-existing conditions
- Changes in vital signs, ECG, physical examination and laboratory test results, if they are judged clinically relevant by the investigator

If such abnormalities already exist prior to trial inclusion, they will be considered as baseline conditions and should be collected in the eCRF only.

5.2.6.1.2 Serious adverse event

An SAE is defined as any AE that fulfils at least one of the following criteria:

- Results in death
- Is life-threatening, which refers to an event in which the participant was at risk of death at the time of the event; it does not refer to an event that hypothetically might have caused death if more severe
- Requires inpatient hospitalisation or prolongation of existing hospitalisation
- Results in persistent or significant disability or incapacity
- Is a congenital anomaly / birth defect
- Is deemed serious for any other reason if it is an important medical event when based on appropriate medical judgement which may jeopardise the participant and may require medical or surgical intervention to prevent one of the other outcomes listed in the above definitions. Examples of such events are intensive treatment in an emergency room or at home for allergic bronchospasm, blood dyscrasias or convulsions that do not result in hospitalisation or development of dependency or abuse

Proprietary confidential information © 2023 Boehringer Ingelheim International GmbH or one or more of its affiliated companies

5.2.6.1.3 AEs considered "Always Serious"

In accordance with the European Medicines Agency initiative on Important Medical Events, Boehringer Ingelheim has set up a list of AEs, which by their nature, can always be considered to be "serious" even though they may not have met the criteria of an SAE as defined in Section 5.2.6.1.2. The latest list of "Always Serious AEs" can be found in the eDC system. A copy of the latest list of "Always Serious AEs" will be provided upon request. These events should always be reported as SAEs as described in Section 5.2.6.2.

Cancers of new histology and exacerbations of existing cancer must be classified as a serious event regardless of the time since discontinuation of the drug and must be reported as described in Section 5.2.6.2, subsections "AE Collection" and "AE reporting to sponsor and timelines".

All C-SSRS reports of suicidal ideation Type 4 or 5 and all reports of suicidal behaviour must be reported as separate SAEs by the investigator.

5.2.6.1.4 Adverse events of special interest

The term "adverse event of special interest" (AESI) relates to any specific AE that has been identified at the project level as being of particular concern for prospective safety monitoring and safety assessment within this trial, e.g. the potential for AEs based on knowledge from other compounds in the same class. AESIs need to be reported to the sponsor's Pharmacovigilance Department within the same timeframe that applies to SAEs; see Section 5.2.6.2.2.

The following are considered as AESIs:

Potential severe drug-induced liver injury

A potential severe DILI that requires follow-up is defined by any of the alerts (alterations) of hepatic laboratory parameters shown in Table 11. Liver enzymes should be repeated or IMP interrupted as described.

These laboratory findings constitute a hepatic injury alert, and trial participants showing any of these lab abnormalities need to be followed up according to the DILI checklist provided in the EDC system and the ISF.

In case of clinical signs or symptoms of hepatic injury (icterus, unexplained encephalopathy, unexplained coagulopathy, right upper quadrant abdominal pain, etc.) without lab results (ALT, AST, total bilirubin) available, the investigator should make sure these parameters are analysed, if necessary, in an unscheduled blood test. Should the results meet the criteria of hepatic injury alert, the procedures described in the DILI checklist should be followed.

Trial participants should be followed up until resolution of symptoms or signs in the above stated situations [P09-12413]. Treatment with the IMP may be restarted only if another aetiology is clearly identified and the liver values have returned to baseline [R20-1981]. If DILI is confirmed, after resolution or stabilisation, the trial participant should complete the procedures for the EOT and FU/EOS visits as outlined in the SoA.

Following completion of the DILI checklist, if the IMP cannot be excluded as a possible cause of DILI event, then discontinuation should be made permanent without rechallenge. If

Proprietary confidential information © 2023 Boehringer Ingelheim International GmbH or one or more of its affiliated companies

an alternate causality is confirmed by the DILI checklist evaluation, then IMP may be re-started, if warranted.

Table 11 Algorithm for monitoring and management of possible hepatocellular DILI in trial participants with normal or elevated baseline ALT[1]

**Treatment emergent ALT**	**Treatment emergent total bilirubin**	**Liver related symptoms**	**Action**[2]
Normal/near normal baseline: ALT ≥5x ULN<br>Elevated baseline[1]: ALT ≥3x baseline or ≥300 U/L (whichever occurred first)	Normal<br><br>Patients with Gilbert's syndrome: no change in total bilirubin	None	Repeat ALT, AST, alkaline phosphatase, and total bilirubin in 2-5 days. Initiate evaluation for other aetiologies of abnormal liver tests.
Normal/near normal baseline: ALT ≥3x ULN<br>Elevated baseline[1]: ALT ≥2x baseline or ≥300 U/L (whichever occurred first)	Normal<br><br>Patients with Gilbert's syndrome: no change in total bilirubin	Severe fatigue, nausea, vomiting, right upper quadrant pain	Repeat ALT, AST, alkaline phosphatase, and total bilirubin in 2-5 days. Initiate evaluation for other aetiologies of abnormal liver tests.
Normal/near normal baseline: ALT ≥8x ULN<br>Elevated baseline[1]: ALT ≥5x baseline or ≥500 U/L (whichever occurred first)	Normal<br><br>Patients with Gilbert's syndrome: no change in total bilirubin	None	Interrupt IMP. Initiate close monitoring and workup for competing aetiologies. IMP can be restarted only if another aetiology is identified, and liver enzymes return to baseline.
Normal/near normal baseline: ALT ≥3x ULN<br>Elevated baseline[1]: ALT ≥2x baseline or ≥300 U/L (whichever occurred first)	Total bilirubin ≥2x ULN<br><br>Patients with Gilbert's syndrome: doubling of direct bilirubin or increased INR to >1.5	None	Interrupt IMP. Initiate close monitoring and workup for competing aetiologies. IMP can be restarted only if another aetiology is identified, and liver enzymes return to baseline.
Normal/near normal baseline: ALT ≥5x ULN<br>Elevated baseline[1]: ALT ≥3x baseline or ≥300 U/L (whichever occurred first)	Normal or elevated	Severe fatigue, nausea, vomiting, right upper quadrant pain	Interrupt IMP. Initiate close monitoring and workup for competing aetiologies. IMP can be restarted only if another aetiology is identified, and liver enzymes return to baseline.

[1]Elevated baseline is defined as ALT ≥1.5x ULN.

[2]The actions of close observation, monitoring, and IMP interruption often overlap. Occasionally, workup is initiated after IMP interruption.

Source data: [R20-1981]

Thyroid malignancies and C-cell hyperplasia

Individuals with personal or family history of MTC and/or MEN-2 will be excluded from the study. Trial participants who are diagnosed with MTC during the study will have the IMP stopped.

Proprietary confidential information © 2023 Boehringer Ingelheim International GmbH or one or more of its affiliated companies

The assessment of thyroid safety during the trial will include reporting of any case of thyroid malignancy (including MTC and papillary carcinoma) and measurements of calcitonin.

Trial participants with calcitonin levels ≥100 pg/mL or 29.26 pmol/L will be excluded from the trial. If an increased calcitonin value (≥100 pg/mL or 29.26 pmol/L) is observed during the trial, IMP should be discontinued, and follow-up by an endocrinologist should be done.

This data will be captured in specific eCRFs. All cases of thyroid cancer will be adjudicated.

Systemic and serious cutaneous hypersensitivity or anaphylactic reactions

All systemic and serious cutaneous hypersensitivity or anaphylactic reactions will be reported as AESIs. Further details are described in Section 4.2.1.3.

Acute gallbladder disease

All events of treatment emergent biliary colic, cholecystitis, or other suspected events related to gallbladder disease should be evaluated and additional diagnostic tests performed, as needed. In cases of elevated liver markers, hepatic monitoring should be initiated as outlined in Table 11. Additional data should be recorded on the specific eCRF page.

Severe hypoglycaemia requiring assistance (Level 3)

At the time of signing consent, all trial participants with T2DM will be educated about signs and symptoms of hypoglycaemia, how to treat hypoglycaemia, and how to collect information for each episode of hypoglycaemia. Hypoglycaemia may be identified by spontaneous reporting of symptoms from trial participants, by plasma glucose samples collected during in-clinic visits, or by home glucose monitoring device. The trial participants will be informed about the need to notify the investigator about all cases when they experience a hypoglycaemic episode. Upon onset of a hypoglycaemic episode, the trial participant is recommended to measure and record blood glucose every 15 min until the value is >3.9 mmol/L (70 mg/dL) and/or symptoms have been resolved in accordance with current guidelines. When a trial participant experiences a hypoglycaemic episode, the participant should record the general information in relation to the hypoglycaemia (date, time, blood glucose measurements, and any symptoms in the eDiary). Blood glucose should always be measured and recorded when a hypoglycaemic episode is suspected. Hypoglycaemic events will be recorded in the eCRF using the definitions in Table 12.

Table 12 Classification of hypoglycaemia

**Level**	**Glycaemic criteria/description**
Level 1	Glucose <70 mg/dL (3.9 mmol/L) and ≥54 mg/dL (3.0 mmol/L)
Level 2	Glucose <54 mg/dL (3.0 mmol/L)
Level 3	A severe event characterised by altered mental and/or physical status requiring assistance for treatment of hypoglycaemia

Source data: [R22-4277]

Hypoglycaemic events should be treated, and additional glucose monitoring should be implemented per investigator discretion and medical judgement. All hypoglycaemic episodes will be recorded in a specific eCRF page. All symptomatic hypoglycaemic events should be

Proprietary confidential information © 2023 Boehringer Ingelheim International GmbH or one or more of its affiliated companies

reported as an AE. All Level 3 hypoglycaemic events (severe events characterised by altered mental and/or physical status requiring assistance) should be reported as an AESI.

If the hypoglycaemic episode fulfils the criteria for an SAE, then reporting should be done as described in Section 5.2.6.2.2. One AE entry can cover several hypoglycaemic episodes, if the trial participant has not recovered between the episodes. All blood glucose values ≤3.9 mmol/L (70 mg/dL, with or without hypoglycaemic symptoms) should be reported as a hypoglycaemic episode.

5.2.6.1.5 Intensity (severity) of AEs

The intensity (severity) of adverse events will be classified and recorded in the eCRF according to the Common Terminology Criteria for Adverse Events (CTCAE) Version 5.0 released on 27 Nov 2017 [R18-1357].

5.2.6.1.6 Causal relationship of AEs

Medical judgement should be used to determine the relationship between the AE and the Boehringer Ingelheim IMP, considering all relevant factors, including pattern of reaction, temporal relationship, de-challenge or re-challenge, confounding factors such as concomitant medication, concomitant diseases, and relevant history.

Arguments that may suggest a reasonable possibility of a causal relationship could be:

- The event is consistent with the known pharmacology of the IMP
- The event is known to be caused by or attributed to the drug class
- A plausible time to onset of the event relative to the time of IMP exposure
- Evidence that the event is reproducible when the IMP is reintroduced
- No medically sound alternative aetiologies that could explain the event (e.g. pre-existing or concomitant diseases, or comedications)
- The event is typically drug-related and infrequent in the general population not exposed to drugs (e.g. Stevens-Johnson syndrome)
- An indication of dose-response (i.e. greater effect size if the dose is increased, smaller effect size if dose is reduced)

Arguments that may suggest no reasonable possibility of a causal relationship could be:

- No plausible time to onset of the event relative to the time of IMP exposure is evident (e.g. pretreatment cases, diagnosis of cancer or chronic disease within days / weeks of IMP administration; an allergic reaction weeks after discontinuation of the IMP concerned)
- Continuation of the event despite the withdrawal of the IMP, considering the pharmacological properties of the compound (e.g. after 5 half-lives).
  Of note, this criterion may not be applicable to events whose time course is prolonged despite removing the original trigger
- There is an alternative explanation, e.g. situations where other drugs or underlying diseases appear to provide a more likely explanation for the observed event than the IMP concerned

Proprietary confidential information © 2023 Boehringer Ingelheim International GmbH or one or more of its affiliated companies

- Disappearance of the event even though trial IMP treatment continues or remains unchanged

5.2.6.1.7 Adverse events requiring additional data collection

The adverse events listed below are considered critical for the assessment of the safety profile of survodutide and will be recorded in a separate eCRF page. Some of them will be centrally adjudicated in a blinded fashion by an external clinical event committee, independent from the sponsor.

Events requiring adjudication:

- Major adverse CV events (5P-MACE), including
  - non-fatal MI
  - non-fatal stroke
  - ischaemia related coronary revascularisation
  - HF event (including hospitalisation for HF, emergency room visit, urgent care visit, or urgent outpatient HF visit)
  - CV death
- Non-CV death
- Acute pancreatitis
- Thyroid malignancies and C-cell hyperplasia
- Pancreatic cancer

<u>Acute pancreatitis</u>

Acute pancreatitis is an acute inflammatory process of the pancreas that may also involve peripancreatic tissues and/or remote organ systems.

If acute pancreatitis is suspected, IMP should be stopped and appropriate laboratory tests (including levels of pancreatic amylase and lipase) should be obtained via the central laboratory (and locally, if needed). If there is evidence of increase in amylase or lipase (3x ULN), an abdominal CT scan with contrast should be performed to confirm the diagnosis. If a CT scan with contrast is contraindicated, a CT without contrast or MRI should be performed. If imaging does not support the diagnosis of acute pancreatitis, the IMP can be restarted as per investigator's judgment. If abdominal imaging supports the diagnosis of acute pancreatitis, IMP should be discontinued, and the participant should be requested to continue in the study (see also Section 3.3.4.1). A review of the participant's concomitant medications should be conducted to assess any potential causal relationship with pancreatitis.

If typical signs and/or symptoms of pancreatitis are present and confirmed by laboratory values (lipase or amylase [total and/or pancreatic]) and imaging studies, the event must be reported as an SAE. For a potential case that does not meet all of these criteria, it is up to the investigator to determine the seriousness of the case (AE or SAE) and the relatedness of the event to the IMP.

As a part of safety monitoring each trial participant will have measurements of amylase and lipase (assessed at the central laboratory) as shown in SoA the to assess the effects of the

Proprietary confidential information © 2023 Boehringer Ingelheim International GmbH or one or more of its affiliated companies

investigational doses of survodutide on pancreatic enzyme levels. Relevant data from participants with acute or chronic pancreatitis will be entered into a specifically designed eCRF page.

All cases of pancreatitis will be adjudicated.

Pancreatic cancer

Based on data from experimental studies in animal models, it has been speculated that the chronic over-stimulation of GLP-1R in exocrine pancreatic cells could induce pancreatitis, ultimately leading to an increased risk of pancreatic cancer. Cases of pancreatic cancer will be adjudicated, and details entered into a specifically designed eCRF page.

Events requiring additional data collection (these events will not be adjudicated):

Local tolerability and injection site reactions

Local tolerability at the administration site of survodutide will be assessed by the investigator during the IMP administration visits and at the time of AE assessment by questioning retrospectively since the last visit. Trial participants will answer the site reaction question in the eDiary after each home injection. The eDiary will be reviewed during the in-clinic visit. Any observed local tolerability reaction, e.g. “swelling”, “induration”, “heat”, or “redness” should be collected on the specific eCRF page, and reported as AE if clinically significant.

Acute severe renal events

Renal safety will be assessed based on repeated renal functional assessment as well as assessment of AEs suggestive of acute or worsening of chronic renal failure. Survodutide has not shown to be nephrotoxic, however, consistent with other GLP-1R agonists, GI AEs have been reported with survodutide, including nausea, diarrhoea, and vomiting. The events may lead to dehydration, which could cause a deterioration in renal function, including acute renal failure. Participants should be advised to notify investigators in case of severe nausea, frequent vomiting, diarrhoea, or symptoms of dehydration. Renal function should be closely monitored in participants with GFR <60 mL/min/1.73 m^2 who develop severe vomiting and diarrhoea. Additional data should be recorded on the specific eCRF page.

Persistent and unacceptable hyperglycaemia

Hyperglycaemia can be identified in case of persistent and unacceptable hyperglycaemia, if any of the FPG values (including fasting SMBG values) exceeds 15 mmol/L (270 mg/dL) and no intercurrent cause of the hyperglycaemia can be confirmed (refer to Sections 4.2.1.1.1 and 4.2.1.1.2). Persistent and unacceptable episodes of hyperglycaemia should be recorded as AEs and in the specific eCRF page.

Worsening of diabetic retinopathy or maculopathy

Fast improvements in glucose levels may lead to a temporary worsening of diabetic eye disease. Trial participants with uncontrolled and potentially unstable diabetic retinopathy or maculopathy within the past 3 months before screening or in the period between screening and randomisation are excluded. All eye examinations including worsening of diabetic retinopathy or maculopathy should be recorded in the specific eCRF page (refer to Section 5.2.5.2).

Proprietary confidential information © 2023 Boehringer Ingelheim International GmbH or one or more of its affiliated companies

Hypotension, orthostatic hypotension, and syncopal events

Clinically significant hypotension, orthostatic hypotension, and syncopal events should be recorded in the specific eCRF page.

Arrhythmias and cardiac conduction disorders

Treatment-emergent clinically significant cardiac conduction disorders will be recorded as an AE. Events that meet criteria for serious conditions must be reported as SAEs. All clinically significant arrhythmias and cardiac conduction disorders should be recorded in the specific eCRF page.

Major depressive disorder, requiring hospitalisation

Participants will be monitored for depression through AE collection and by using the PHQ-9. For further details, refer to Section 5.2.5.1.

Suicidal ideation and behaviour

Participants will be monitored for suicidal ideation and behaviour through AE collection and by using the C-SSRS. For further details, refer to Section 5.2.5.1.

5.2.6.2 Adverse event collection and reporting

5.2.6.2.1 AE Collection

The investigator shall maintain and keep detailed records of all AEs in the participant files.

The following must be collected and documented:

All AEs (serious and non-serious) and all AESIs from signing the informed consent onwards until the individual trial participant's EOS (usually the FU/EOS visit), see Section 5.2.6.2.2.

After the individual trial participant's EOS visit, the investigator does not need to actively monitor the trial participant for new AEs. However, the following events should be reported:

- Any occurrence of cancer of new histology
- IMP related SAEs
- IMP related AESIs of which the investigator may become aware of by any means of communication, e.g. phone call.

Vital status data collection

Trial participants who discontinue IMP prematurely, and agree to be contacted further, should be followed up as described in Section 3.3.4.1. The above-described AE reporting requirements apply as far as this is possible. If a trial participant only agrees to be contacted for vital status data collection, the investigator must report until the individual participant's EOS visit any occurrence of cancer, all deaths/fatal AEs regardless of relationship, IMP related SAEs, and IMP related AESIs the investigator becomes aware of.

Proprietary confidential information © 2023 Boehringer Ingelheim International GmbH or one or more of its affiliated companies

5.2.6.2.2 AE reporting to the sponsor and timelines

The investigator must report SAEs, AESIs, and non-serious AEs that are relevant for the reported SAE or AESI immediately (within 24 h of becoming aware of the event). SAE will be reported using paper SAE forms. The country-specific process will be described in the ISF. On specific occasions, the investigator could inform the sponsor upfront via telephone in addition.

With receipt of any further information on these events, follow-up reports must be provided. For follow-up information, the same rules and timelines apply as for initial information. All AEs / SAEs, including those persisting after an individual participant's end of trial must be followed up until they have resolved, have been assessed as "chronic" or "stable", or no further information can be obtained.

5.2.6.2.3 Pregnancy

In rare cases, pregnancy might occur in a clinical trial. Once a trial participant has been enrolled in the clinical trial and has taken trial IMP, the investigator must report any IMP exposure during pregnancy in a trial participant immediately (within 24 h) by means of Part A of the Pregnancy Monitoring Form to the sponsor's unique entry point.

It is not required to report the pregnancy of a partner of a male trial participant.

The outcome of the pregnancy associated with the IMP exposure during pregnancy must be followed up and reported to the sponsor's unique entry point on the Pregnancy Monitoring Form for Studies (Part B).

The ISF will contain the Pregnancy Monitoring Form for Studies (Parts A and B).

As pregnancy itself is not to be reported as an AE, in the absence of an accompanying SAE and/or AESI, only the Pregnancy Monitoring Form for Studies is to be completed. However, an SAE and / or AESI associated with the pregnancy must be reported as described in Section 5.2.6.2.2.

5.2.6.2.4 Other safety topics

Severe and serious gastrointestinal events

Survodutide may cause severe GI AEs, such as nausea, vomiting, and diarrhoea. Information about severe and serious GI AEs will be collected in the AE form and information regarding antiemetic/antidiarrheal medication use will be collected in the Concomitant Medication form. For detailed information concerning the management of GI AEs, please refer to Section 4.1.4.1.

Calcitonin monitoring

Trial participants with calcitonin levels ≥100 pg/mL or 29.26 pmol/L during screening will be excluded from the trial. If an increased calcitonin value (≥100 pg/mL or 29.26 pmol/L) is observed during the course of the trial, IMP should be discontinued, and follow-up by an endocrinologist should be done.

Proprietary confidential information © 2023 Boehringer Ingelheim International GmbH or one or more of its affiliated companies

5.3 DRUG CONCENTRATION MEASUREMENTS AND PHARMACOKINETICS

5.3.1 Assessment of pharmacokinetics

Date and clock time of drug administration as well as actual date and clock time of PK sampling will be captured in the database.

For PK analysis, trough plasma concentration of survodutide will be used for the evaluation of the influence of intrinsic and extrinsic factors on survodutide.

A population PK analysis will be undertaken using appropriate recognised software to analyse PK sampling data. The trough plasma concentrations will be used to refine an already existing population PK model. Exposure predictions based on the population PK model may subsequently be used for characterizing the exposure-response relationships for selected efficacy and/or safety endpoints.

5.3.2 Methods of sample collection

Detailed instructions on sampling, preparation, processing, shipment, and storage are provided in the laboratory manual.

For quantification of survodutide plasma concentrations, blood samples will be taken at the time points described in the SoA. Blood will be withdrawn by means of either an indwelling venous catheter or by venepuncture with a metal needle.

At a minimum, the sample tube labels should list the following information: BI trial number, trial participant number, and visit. Further information such as matrix and analyte may also be provided.

Plasma samples will be discarded at the latest 6 months after the final clinical trial report has been signed.

All lab kit supplies will be provided by the central lab vendor designated by the sponsor.

[redacted]

5.3.4 Pharmacokinetic - pharmacodynamic relationship

An analysis of the relationship between PK and explorative biomarker parameters may be investigated in an exploratory manner. Results will be reported in a separate report.

Proprietary confidential information © 2023 Boehringer Ingelheim International GmbH or one or more of its affiliated companies

[redacted]

Proprietary confidential information © 2023 Boehringer Ingelheim International GmbH or one or more of its affiliated companies

[redacted]

5.5 BIOBANKING

For sampling timepoints, see the SoA. Biobanking will be performed only in countries where allowed by local laws and regulations. Participation in biobanking is voluntary and not a prerequisite for participation in the trial. Biobanking will occur only after a separate biobanking informed consent has been given in accordance with local ethical and regulatory requirements.

To be able to address future scientific questions, trial participants will be asked to voluntarily donate biospecimens for banking. If the trial participant agrees, banked samples may be used for future biomarker research and drug development projects, e.g. to identify patients that are more likely to benefit from a treatment or experience an AE, or to gain a mechanistic or genetic understanding of drug effects and thereby better match patients with therapies. Samples will be stored for testing for a period consistent with local regulations.

5.5.1 Methods and timing of sample collection

Detailed instructions on sampling, preparation, processing, shipment, and storage are provided in the laboratory manual. Samples will be collected prior to the IMP administration. For sampling time points, see SoA.

Blood will be drawn for DNA, plasma and serum banking purposes. Any analyses on DNA or plasma/serum samples will not be reported in the main clinical trial report. Samples will be collected only in countries where all applicable local regulatory and ethics approvals have been obtained for biobanking.

5.6 OTHER ASSESSMENTS

5.6.1 Immunogenicity

5.6.1.1 Timing of immunogenicity measures

Blood samples will be obtained from trial participants at the time points detailed in the SoA. Serum samples will be analysed for the presence of ADAs and NAbs. ADAs will be

Proprietary confidential information © 2023 Boehringer Ingelheim International GmbH or one or more of its affiliated companies

evaluated for their specificity towards survodutide and their ability to cross-react with GLP-1 or glucagon. Similarly, NAbs will be evaluated towards their ability to neutralise survodutide, and may also be evaluated for their ability to cross-react and neutralise endogenous GLP-1 or glucagon. The impact of ADAs/NAbs on PK, clinical safety and efficacy will be evaluated.

Additionally, blood for ADA and NAb assessment will be collected upon observation of any immunologically related AEs, and the serum concentration of survodutide will be assessed.

5.6.1.2 Methods of sample collection

Detailed instructions on sampling, preparation, processing, shipment, and storage are provided in the laboratory manual.

For both, ADA (incl. cross-reactive binding antidrug antibodies) and NAb (incl. cross-neutralization of GLP-1 and glucagon) assessments, blood will be taken for each time point described in the SoA via venipuncture or indwelling catheter into serum blood-drawing tubes at the time points listed in the Flow Chart, and additionally in the event of any severe hypersensitivity reaction as mentioned above.

At a minimum, the sample tube labels should list the following information: BI trial number, trial participant number, visit, matrix, and analyte (ADA or NAb).

Serum samples will be shipped on dry ice and stored in a freezer set at -70°C until and after analysis. The samples may be used for further ADA or NAb characterization or methodological investigations (e.g. for stability testing, isotyping, cross-reactivity, cross-neutralization), however, only data related to ADAs or NAbs will be generated by these additional investigations. The trial samples will be discarded after completion of the additional investigations but not later than 5 years after the final clinical trial report has been signed.

All lab kit supplies will be provided by the central lab vendor designated by the sponsor.

5.6.1.3 Immunogenicity analysis

The presence of ADAs to survodutide will be assessed via a tiered approach using validated assays. All pre- and post-dose samples will be tested in the screening assay and positive samples in the screening assay will be tested in the confirmatory assay. Trial samples that are confirmed positive may be further characterised for titer assessment, cross-reactivity to GLP-1, cross-reactivity to glucagon, neutralization of survodutide activity on GLP-1R and neutralization of survodutide activity on GCGR. Samples that are positive for cross-reactivity to GLP-1 will be tested for cross-neutralization of GLP-1. Samples that are positive for cross-reactivity to glucagon will be tested for cross-neutralization of glucagon.

5.6.2 Clinical staging of obesity

Staging of obesity will be assessed by the investigator at screening using the Edmonton obesity staging, based on the medical history. The Edmonton obesity staging system is used for clinical prioritisation and management of obesity independent of BMI [R20-1982]. For details, refer to Section 10.4.

Proprietary confidential information © 2023 Boehringer Ingelheim International GmbH or one or more of its affiliated companies

[redacted]

5.7 APPROPRIATENESS OF MEASUREMENTS

All measurements performed during this trial are standard measurements and will be performed in order to evaluate efficacy and tolerability of survodutide and to determine PK and PD parameters in an appropriate way. The scheduled measurements will allow monitoring of changes in vital signs, standard laboratory values, and ECG parameters that might occur as a result of administration of the trial drug. The safety assessments are standard, are accepted for evaluation of safety and tolerability of an s.c. administered drug and are widely used in clinical trials. The PK parameters and measurements outlined are generally used assessments of drug exposure.

Proprietary confidential information © 2023 Boehringer Ingelheim International GmbH or one or more of its affiliated companies

6. INVESTIGATIONAL PLAN

6.1 VISIT SCHEDULE

For a detailed overview of the trial procedures, in-clinic and remote visits (telemedicine, if allowed per local regulations), and time windows for visits, please refer to the SoA.

The trial consists of a screening visit followed by a dose escalation period of up to 24 weeks (or up to a maximum of 32 weeks if 1 or 2 re-escalation attempts are needed due to occurrence of GI symptoms), a maintenance period of 24 weeks, and an FU/EOS Visit. The FU Visit for safety assessments is scheduled 28 days after the last administration of the IMP (21 days after the Week 48/EOT Visit).

A pre-screening approach is recommended in this trial. The study site will be able to review their records in a structured process to identify potential subjects who would most likely qualify for the trial (e.g. overweight/ obesity, presence of metabolic comorbidities, previous NAFLD/NASH diagnosis, biochemical liver tests and non-invasive liver fibrosis tests). Time windows of ±3 days (dose escalation period) or ±7 days (maintenance period and follow-up) for scheduled visits are allowed to accommodate scheduling problems. These time windows apply to scheduled visits, not to IMP administration, which may not occur on the same day as a visit. Time windows for IMP administration are outlined in Section 4.1.4. If a delay is observed for a particular visit, the original calendar schedule should be kept for subsequent visits (delays should not accumulate).

If a visit is missed, the trial participant should be instructed to reach out to the investigator as soon as possible. Handling of missed IMP injections is described in Section 4.1.4. The investigator should contact the CRO monitor to discuss the further schedule.

Trial participants who completed the trial treatment as planned will have an FU/EOS Visit 21 days after the EOT visit following the completion of the treatment period. For more details (e.g. for trial participants who discontinued prematurely), refer to Section 6.2.3. Procedures to be completed at the FU Visit can be found in the SoA. The sequence of visit procedures will be the same as in the treatment period.

6.2 DETAILS OF TRIAL PROCEDURES AT SELECTED VISITS

Please refer to Section 5 for explanations of procedures or observations and to the SoA for corresponding time points.

An electronic Study Hub with a secure online platform from an external CRO will be used in this study. This Study Hub is the place where the study site staff can communicate with the trial participant (including video conference), complete study tasks, and receive important information about the study. Trial participants will access the Study Hub using a secure app on a smartphone or tablet or by using a secure website through any computer. Trial participant will have “Study Hub Technical Support” that is accessible 24 h/day, 7 days/week.

The eCOA platform will be integrated with the Study Hub. The eCOA platform will include electronic PROs as well as the eDiary. The e-consent process will not be integrated with the Study Hub, but with the “Complete Consent“ platform.

Proprietary confidential information © 2023 Boehringer Ingelheim International GmbH or one or more of its affiliated companies

Electronic tools will be implemented only in participating countries where this is permitted based on applicable approvals from local regulatory authorities and ethics committees. Otherwise, a traditional non-electronic approach will be used. In exceptional cases, if a trial participant is unable to use the electronic platforms, a back-up solution with paper will be made available upon sponsor approval.

6.2.1 Screening period

Screening of participants for this trial is competitive, i.e. screening for the trial will stop at all sites at the same time once a sufficient number of participants has been screened. Investigators will be notified about screening completion and will then not be allowed to screen additional participants for this trial. Participants already in screening at this time will be allowed to continue to randomisation if eligible.

Whilst the information provided below is not an exhaustive list, it provides some guidance as to when such re-screening and/or re-testing would be considered appropriate.

Re-testing for eligibility criteria (within the defined screening window period):

- Is only to be performed once for ECG, vital signs, and a laboratory result which is obviously received beyond stability at the central laboratory, thought to be a spurious result based on previously available laboratory results, or if the Investigator believes the ineligible result is caused by an error or extenuating circumstance.
- The re-test should be carried out as soon as possible so that the laboratory test results can be available prior to the randomisation visit.

Re-screening

- Re-screening of the same trial participant is only allowed once.
- The trial participant should be declared a screening failure in the eCRF and IRT with their original trial participant number.
- Upon re-screening, the IRT system will allocate a new screening number for the trial participant.
- All procedures of Visit 1 must be repeated except for FibroScan® and MRI. FibroScan® and MRI from the initial screening can be used if the assessments were performed within 1 month prior to re-screening. Data for FibroScan® and MRI from the initial screening failure will be transferred to the corresponding re-screening number.
- Trial participants who were considered ineligible because of uncontrolled hypertension (see Section 3.3.3), can be re-screened at the discretion of the investigator after good blood pressure control has been achieved.

Re-screening of a previously screen failed patient will be permitted providing the reasons for screen failure were reversible and have been resolved, based on Investigator's judgement. A log of all participants enrolled in the trial (i.e. who have signed informed consent) will be maintained in the ISF, irrespective of whether or not IMP was administered to the participant. If retrospectively it is found that a trial participant has been randomised in error (i.e. did not meet all inclusion criteria or met one or more exclusion criteria), the sponsor or delegate

Proprietary confidential information © 2023 Boehringer Ingelheim International GmbH or one or more of its affiliated companies

should be contacted immediately. Based on an individual benefit-risk assessment, a decision will be made as to whether continued trial participation is possible.

<u>Screening</u>

During the screening visit, demographics and baseline information will be collected:

- Age (in years) on the day of informed consent
- Sex (male or female, in order to describe the trial participant's sex at birth)
- Gender identity (male, female, non-binary, not answered, or other in order to describe how the trial participant self-identifies, regardless of their genotypic or phenotypic sex) if 1) locally accepted (i.e. based on HA/EC/IRB acceptance, independent of acceptance by investigators or participants), 2) investigators are willing to ask, and 3) participants are willing to reply
- For women of childbearing potential, yes/no to characterize the patient population and as a basis for contraception requirements
- Ethnicity and race to sufficiently characterise the patient population, to support possible subgroup analyses if needed, unless not acceptable according to local regulations
- Medical and weight history, baseline conditions and concomitant therapy
- Height and weight (for obesity staging)
- Physical examination, vital signs (SBP, DBP, pulse), and a 12-lead ECG
- PHQ-9 and C-SSRS
- Eye examination for T2DM subjects
- Blood samples (for safety laboratory tests and infectious serology)
- FIB-4 score
- Review of inclusion and exclusion criteria

If the trial participant meets no exclusion criteria and fulfils at least one of the three conditions below:

- Recent liver biopsy (within 3 years) consistent with NASH (defined as the presence of steatosis, inflammation, and ballooning) with stage ≤3 fibrosis according to the NASH Clinical Research Network classification OR
- FIB-4 score ≥2.67 and <3.48 at screening OR
- Non-invasive test results in the previous 6 months: MRE ≥2.61 and <4.68 kPa, OR MAST score ≥0.242, OR FAST score ≥0.5, OR FibroScan® VCTE ≥8 kPa and <20 kPa), OR ELF score >7.7 and <11.3, OR cT1 ≥875 ms

then the FibroScan® VCTE™ + CAP™ (VCTE™ only if CAP™ not available) should be performed. In case VCTE <20 kPa, MRI should be performed. In case VCTE ≥20 kPa, the participant should be registered as a screen failure in the IRT and eCRF screen failure page should be recorded.

Proprietary confidential information © 2023 Boehringer Ingelheim International GmbH or one or more of its affiliated companies

Participants not fulfilling at least one of the three conditions mentioned above and FIB-4 score <1.3 should be registered as screen failures in the IRT and eCRF screen failure page should be recorded.

For the remaining participants with FIB-4 score ≥1.3, the FibroScan® VCTE™ + CAP™ (VCTE™ only if CAP™ not available) should be performed to assess the FAST score and/or the VCTE results for eligibility criteria. In case FAST ≥0.5 or VCTE ≥8 kPa (and VCTE <20 kPa), MRI should be performed. In case FAST score/VCTE inclusion criteria are not met (or VCTE ≥20 kPa), the participant should be registered as a screen failure in the IRT and eCRF screen failure page should be recorded. Figure 3 shows the flowchart of assessments at Visit 1.

Proprietary confidential information © 2023 Boehringer Ingelheim International GmbH or one or more of its affiliated companies

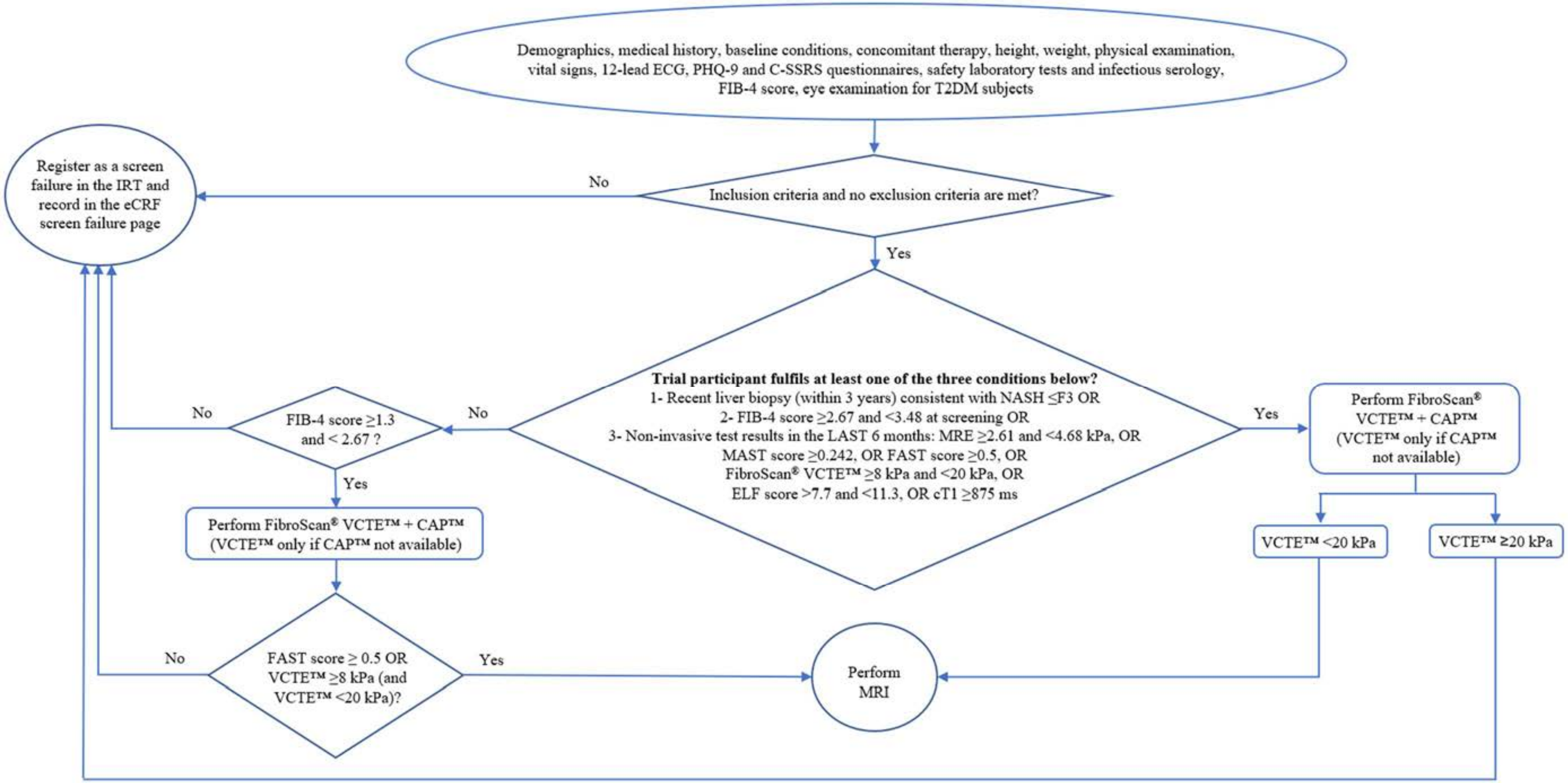

Figure 3 Flowchart of assessments at Visit 1

Proprietary confidential information © 2023 Boehringer Ingelheim International GmbH or one or more of its affiliated companies

6.2.2 Treatment period

[redacted]

Trial participants who meet all protocol criteria will be randomised at Visit 2. After randomisation, trial participants will start the treatment period which includes a dose escalation phase, followed by the dose maintenance phase as shown in Figure 2.

[redacted]

Procedures to be completed at each visit can be found in the SoA.

At visits specified in the SoA, trial participants should be instructed to come in the fasting state after at least 8 h without food or liquid intake, except water. If the IMP injection date is scheduled during the date of the visit, the trial participant needs to wait with injection of the IMP as this will be done on site after blood sampling.

The in-clinic visit should be scheduled in the mornings, if this is convenient for the trial participant.

Sequence of assessments

For in-clinic visits after randomisation and review of inclusion/exclusion criteria, the suggested sequence of assessments (as applicable) is:

1. Completion of questionnaires
2. Vital signs and other applicable assessments
3. Physical examination
4. ECG
5. Blood draws and urine collection
6. Dosing with IMP

[redacted]

5. PHQ-9
6. C-SSRS

At each visit, trial participants should be advised to follow the recommended diet and exercise plan and to record their compliance regularly (at least weekly) on the provided food, exercise, and weight diary. The trial participants can also use their own tools (e.g. paper or local applications) that they have been using prior to enrolment for tracking their diet, body

Proprietary confidential information © 2023 Boehringer Ingelheim International GmbH or one or more of its affiliated companies

weight, and physical activity. Diet and exercise counselling sessions are held at visits indicated in the SoA. When necessary, from a logistic point of view, it is possible to have the diet/exercise counselling on a different day than the actual in-clinic visit, but within the time window of the visit.

Unscheduled visits may be arranged if necessary. Procedures completed during an unscheduled visit will depend on the circumstances under which the visit was scheduled, and at the discretion of the investigator.

During the remote visits (if allowed per local regulations) the trial participants will be contacted by the site staff. They will be asked about their well-being, occurrence of AEs, and intake of concomitant medications (including standard of care for the weight-related complications). Site staff will conduct the C-SSRS assessment, ask trial participants if they have performed the IMP administration and whether it was documented in the eDiary. Trial participants will be asked if they have followed the diet and physical activity recommendations and have documented it in the paper food, exercise, and weight diary or their own tools. When applicable, site staff will also ask trial participants if they experienced any hypo/hyperglycaemic episodes and if so, whether they documented the glucose measurements for these episodes in the eDiary.

Remote visit responsibilities within the study team will be defined in the study delegation log on site level (e.g. assessment of concomitant therapy and AEs by a physician).

If remote visits are not allowed per local regulations, they will be performed as in-clinic visits.

Exceptionally, and after consultation with the sponsor, one visit could be completed a few days in advance to accommodate for trial participant's imperatives. The call to IRT and IMP allocation would be made, but the IMP injection would occur as per the initial schedule and allowed time window.

During the remote Visits 3 and 5 and 12 the staff will also observe the IMP administration and if needed, perform retraining of the trial participants on the administration technique. Remote visits should occur, by default, via video conferencing within the Study Hub application. In rare cases, the remote visits may be done by phone call. The trial participants will be trained by the study staff how to access the video conference application. All procedures to be performed during the remote visits are specified in the SoA.

After completion of the treatment period, the trial participants will have a Week 48/EOT Visit 7 days after last IMP administration. Trial participants who discontinue trial treatment will have an EOT Visit 7 days after the last IMP dose or as soon as possible.

Safety lab and other laboratory tests:
The analysis will be performed by a central laboratory. The local lab would only be allowed to be documented in exceptional cases and should be discussed with the sponsor.

Minimum required safety lab parameters are:
Haematology: Haematocrit, haemoglobin, erythrocytes, leukocytes, thrombocytes
Clinical chemistry: ALT, AST, bilirubin total, amylase, lipase

Proprietary confidential information © 2023 Boehringer Ingelheim International GmbH or one or more of its affiliated companies

6.2.3 Follow-up period and trial completion

Trial participants who completed the trial treatment as planned will have an FU/EOS Visit 21 days after the EOT visit after the completion of the treatment period. Procedures to be completed at the FU Visit can be found in the SoA. The sequence of visit procedures will be the same as in the treatment period.

All efforts should be made to follow-up on all trial participants until trial end.

Trial participants who discontinue the IMP prematurely and are willing to participate in trial-related activities will be asked to continue the trial visits as planned, including the FU/EOS Visit at Week 51. The Week 51 and EOS eCRF pages must be completed at this visit, because for these participants no follow-up will be performed separately as they will be monitored through the regular scheduled visits.

Trial participants who discontinue the IMP prematurely and are not willing to continue with trial activities should have an FU/EOS Visit 21 days after their EOT Visit.

If a trial participant is not willing to return to the predefined trial visits, at minimum a remote visit (if allowed per local regulations) or phone call at the FU/EOS Visit (that should occur 21 days after EOT visit) will be required to document the occurrence of outcome events, vital status, and body weight (self-reported). Trial participants who withdraw consent should stop taking IMP and should be asked to complete the EOT visit and follow-up procedures as described in the SoA and Section 3.3.4.1.

A trial participant is considered to have completed the trial in case any of the following applies:

- Completion of planned follow-up period (= FU/EOS Visit completed)
- Lost to follow-up
- Refusal to be followed-up
- Death

If needed in the opinion of the investigator, after the FU/EOS Visit additional visits may be scheduled for continued safety monitoring. Abnormal assessments or lab values judged clinically relevant by the investigator will be monitored until they returned to a medically acceptable level.

6.2.4 Exceptional conditions

In the event of force majeure or other disruptive circumstances (e.g. pandemic, war) the execution of the investigational plan as per this clinical trial protocol may not be feasible. With the consent of the participant, the sponsor and investigator may agree on alternative, back-up or rescue methodology which may include but will not be limited to virtual trial participant visits and assessments, direct-to-participant/direct-from-participant shipments of trial treatment or bio-sample pick up from the participant's home. The implementation of these measures will depend on participant's consent, operational feasibility, local law and regulations. If alternative methodology is implemented, the deviations from the original plan will be precisely documented.

Trial participants with obesity are in general at higher risk for severe illness from COVID-19. In exceptional cases, if standard visits at the trial sites are impossible because of COVID-19

Proprietary confidential information © 2023 Boehringer Ingelheim International GmbH or one or more of its affiliated companies

related safety risks, the trial conduct may need to be adjusted, strictly consistent with local public health guidance and regulations. If, in order to minimize the risk of exposure to COVID-19, physical visits should be avoided, the use of remote visits (via phone and/or internet-based means of communication) and local laboratory services for safety and efficacy parameters measurements should be considered if possible. However, IMP shipment to trial participants might not be possible in all countries to be included. In such situations, picking up the IMP could be done by the trial participant's delegate or by the trial participant at a sterile pharmacy (e.g. "drive -through", "isolated" pharmacy). Investigators should complete trial procedures according to the clinical trial protocol to the extent possible. The maximum number of missing doses of IMP is defined in Section 4.1.4. Considering all aspects, the investigator will decide upon each trial participant's (continued) participation in the planned trial. BI as the sponsor, where required, will support the investigator in their decision finding. It is acknowledged that the investigator may decide to implement protocol deviations where this protects the safety, wellbeing and/or is in the best interest of the trial participant. All home/remote visits need to be discussed with and approved by the sponsor`s trial team. Local regulatory and legal requirements of the participating country still apply. All COVID-19 related deviations from the original schedule of visits and procedures will be documented and the implications considered for the analysis of the trial data.

Proprietary confidential information © 2023 Boehringer Ingelheim International GmbH or one or more of its affiliated companies

7. STATISTICAL METHODS AND DETERMINATION OF SAMPLE SIZE

7.1 NULL AND ALTERNATIVE HYPOTHESES

The hypotheses reflecting the primary objective are as follows:

Let OR1_ survodutide/placebo denote the true odds ratio of achievement of MRI-PDFF response ≥30% from baseline to Week 48 between survodutide and placebo.

- $H0_1$: OR1_ survodutide/placebo ≤1 versus HA_1: OR1_ survodutide/placebo >1

Let µ1_ survodutide and µ1_placebo denote the true mean of percentage change in body weight from baseline to Week 48 for survodutide and placebo, respectively.

- $H0_2$: µ1_ survodutide ≤ µ1_placebo versus HA_2: µ1_ survodutide >µ1_placebo

The trial will be considered positive if based on the treatment-regimen estimand both hypotheses $H0_1$ and $H0_2$ are rejected. The overall two-sided significance level is α=5%.

7.2 PLANNED ANALYSES

7.2.1 General considerations

Analysis sets

- Randomised set (RS): the RS includes all randomised trial participants.
- Treated set (TS): the TS is defined as all randomised trial participants who received at least one dose of IMP.

All efficacy and safety analyses will be based on the treated set.

Important protocol deviations
IPD categories will be defined in the IPD specification document.

IPDs will be identified no later than in the Report Planning Meeting. Identification of patients with IPDs will be confirmed at RPM and the patients will be listed in the CTR.

IPDs may include (but not necessarily be limited to) the following:

- Violation of inclusion or exclusion criteria
- Major deviations from scheduled timing of assessments
- Non-compliance with IMP
- Treatment dispensing errors (handling of such participants will be decided on case-by-case basis by the sponsor)
- Use of prohibited or restricted concomitant medication

Proprietary confidential information © 2023 Boehringer Ingelheim International GmbH or one or more of its affiliated companies

Definition of baseline
In general, unless otherwise specified in this document or in the TSAP, the last non-missing measurement on or prior to the date of first dose of IMP will be used as baseline for efficacy and safety variables.

For the purposes of on-treatment safety analyses, an assessment (or AE start date) will be considered "on-treatment" if the assessment date (or AE start date) is between the date of first dose and 28 days after the date of last dose of the IMP.

7.2.2 Handling of Intercurrent Events

The expected ICEs of interest in this trial are listed in Table 13 together with their respective handling. The handling of intercurrent events, that are not listed, will be described in the TSAP.

Table 13 Handling of intercurrent events

**Intercurrent event (ICE)**	**"Treatment-Regimen" Estimand (ICE handling)**	**"Efficacy" Estimand (ICE handling)**
Premature treatment discontinuation	Include all available data<br>("Treatment policy" strategy)	Exclude data after treatment discontinuation + 28 days (residual effect period of survodutide)<br>("Hypothetical" strategy)
Intake of prohibited anti-obesity medication (other than GLP-1R agonist containing medication) while on treatment	Include all available data<br>("Treatment policy" strategy)	Exclude data during intake of any prohibited anti-obesity medication<br>("Hypothetical" strategy)
Intake of prohibited anti-obesity medication (other than GLP-1R agonist containing medication) after premature discontinuation of study drug	Include all available data<br>("Treatment policy" strategy)	Exclude data during intake of any prohibited anti-obesity medication<br>("Hypothetical" strategy)
Intake of prohibited medication containing GLP-1R agonists	Include all available data<br>("Treatment policy" strategy)	Exclude all data after first intake of GLP-1R agonist containing medication<br>("Hypothetical" strategy)
Death from any cause	Include data prior to death ("Hypothetical" strategy for both continuous and binary endpoints)	Include data prior to death ("Hypothetical" strategy for both continuous and binary endpoints)

There will be 2 estimands of interest addressing the primary and secondary objectives: Treatment-Regimen Estimand and Efficacy Estimand.

Treatment-Regimen Estimand

T.1) Treatment regimen under evaluation will be the randomised treatment taken for up to 48 weeks (with a dose escalation of survodutide/placebo during the first 24 weeks after randomisation) as an adjunct to a reduced-calorie diet and increased physical activity and

Proprietary confidential information © 2023 Boehringer Ingelheim International GmbH or one or more of its affiliated companies

including certain anti-hyperglycaemia medication (rescue therapy), certain glucose-lowering, anti-hypertensive, and lipid-lowering medication, and possibly anti-obesity medication.

T.2) Target population: The treatment effect will be estimated for trial participants with a BMI ≥30 kg/m^2 or a BMI ≥27 kg/m^2 with one or more weight-related co-morbidities and presumed or confirmed NASH.

T.3) Endpoint(s): Efficacy will be evaluated using the co-primary endpoints "Relative reduction in liver fat content of at least 30% from baseline to Week 48 assessed by MRI-PDFF (yes/no)" and "Relative (%) change in body weight from baseline to Week 48".

T.4) Intercurrent events:

- For trial participants who initiate a prohibited anti-obesity medication other than GLP-1R agonist containing medication during treatment with IMP or after discontinuation from IMP, who initiate a prohibited medication containing GLP-1R agonists, or who discontinue prematurely from IMP for any reason, observed endpoint values through Week 48 (in T.3) will be used for the analysis.
- For trial participants who die, a hypothetical strategy will be used for the endpoint "Relative change (%) in body weight from baseline to Week 48" to estimate what the treatment effect would be at Week 48 if the trial participants had not died. The binary endpoint "Relative reduction in liver fat content of at least 30% from baseline to Week 48 assessed by MRI-PDFF (yes/no)" will be derived from the continuous endpoint MRI-PDFF liver fat, if available, hence, a hypothetical strategy will be used as well.

T.5) Population-level summary: Adjusted mean "Percentage of change in body weight from baseline to Week 48" will be computed for each randomised treatment group. The survodutide group will be compared with the placebo group using difference of adjusted means. A hypothesis of the difference in adjusted means being less or equal to zero, in favour of survodutide, will be tested.

The proportion of trial participants with "Relative reduction in liver fat content of at least 30% from baseline to Week 48 assessed by MRI-PDFF (yes/no)" will be compared between survodutide and placebo treatment in terms of odds ratios. A hypothesis of the odds ratio being ≤1 between survodutide and placebo group will be tested.

Efficacy Estimand

E.1) Treatment regimen under evaluation will be the randomised treatment taken for up to 48 weeks (with a dose escalation of survodutide/placebo during the first 24 weeks after randomisation) as an adjunct to a reduced-calorie diet and increased physical activity, but excluding certain anti-hyperglycaemia medication (rescue therapy), certain glucose-lowering, anti-hypertensive and lipid-lowering medication, and possibly anti-obesity medication.

E.2) Target population: The treatment effect will be estimated for trial participants with a BMI ≥30 kg/m^2 or a BMI ≥27 kg/m^2 with one or more weight-related co-morbidities and presumed or confirmed NASH.

E.3) Endpoint(s): Efficacy will be evaluated using the co-primary endpoints "Relative reduction in liver fat content of at least 30% from baseline to Week 48 assessed by MRI-PDFF (yes/no)" and "Relative (%) change in body weight from baseline to Week 48".

Proprietary confidential information © 2023 Boehringer Ingelheim International GmbH or one or more of its affiliated companies

E.4) Intercurrent events:

- For trial participants who initiate an anti-obesity medication other than GLP-1R agonist containing medication during treatment with IMP or after discontinuation from IMP, all body weight measurements during intake of the anti-obesity medication will be excluded from the analysis. However, the endpoint measurement at Week 48 will be used in the analysis if the trial participant continued randomised treatment through Week 48 and stopped intake of any anti-obesity medication other than GLP-1R agonist containing medication before Week 48.
- For trial participants who discontinue prematurely from IMP for any reason, all body weight measurements and MRI-PDFF liver fat data after discontinuation + 28 days (residual effect period of survodutide) will be excluded from the analysis. That is, a hypothetical strategy will be used to estimate what the treatment effect would be at Week 48 if trial participants had continued on their randomised treatment through Week 48.
- For trial participants who initiate prohibited medication containing GLP-1R agonists, all body weight measurements and MRI-PDFF liver fat data after start of medication containing GLP-1R agonists will be excluded from the analysis. That is, a hypothetical strategy will be used to estimate what the treatment effect would be at Week 48 if trial participants had not started the prohibited medication containing GLP-1R agonists.
- For trial participants who die, a hypothetical strategy will be used for the endpoint "Relative (%) change in body weight from baseline to Week 48" and endpoint "Relative reduction in liver fat content of at least 30% from baseline to Week 48 assessed by MRI-PDFF (yes/no)" to estimate what the treatment effect would be at Week 48 if the trial participants had not died.

E.5) Population-level summary: Adjusted mean "Percentage change from baseline in body weight from baseline to Week 48" will be computed for each randomised treatment group. The survodutide group will be compared with the placebo group using difference of adjusted means. A hypothesis of the difference in adjusted means being less or equal to zero, in favour of survodutide, will be tested.

The proportion of trial participants with "Relative reduction in liver fat content of at least 30% from baseline to Week 48 assessed by MRI-PDFF (yes/no)" will be compared between survodutide and placebo treatment in terms of odds ratios. A hypothesis of the odds ratio being ≤1 between each of survodutide and the placebo group will be tested.

No multiplicity adjustment will be made for conducting separate analyses relative to Efficacy and Treatment-Regimen estimands, estimands as these two estimands are intended for different purposes.

7.2.3 Primary objective analyses

The "treatment regimen" estimand reflects the treatment effect which includes the effects of any early treatment discontinuation and any prohibited anti-obesity treatment. In addition, the "efficacy" estimand reflects the treatment effect if all trial participants had adhered to

Proprietary confidential information © 2023 Boehringer Ingelheim International GmbH or one or more of its affiliated companies

treatment and had not received any prohibited anti-obesity therapy. Both estimands evaluate the co-primary endpoints in all randomised and treated trial participants (treated set).

7.2.3.1 Main analyses

Primary analyses relative to the estimands as defined above:

Analysis relative to the Treatment-Regimen Estimand:

Analysis of covariance (ANCOVA) with multiple imputation of missing measure and statistical inference over multiple imputation of missing data guided by Rubin [R12-2378] will be performed for the continuous primary endpoint. Missing body weight (and continuous liver fat) data at Week 48 will be imputed based on 'retrieved dropouts', defined as participants who had their value measured at Week 48 in the same treatment arm who prematurely discontinued study drug. In cases where there are not enough retrieved dropouts to provide a reliable imputation model (i.e. the model does not converge), multiple imputation will be based on the 'follow the reference' strategy. That is, after ICE 'treatment discontinuation', trial participants in the survodutide arm are assumed to have an effect similar to that of the placebo arm while preserving information of any on-treatment and off-treatment data. ANCOVA will include treatment and region and T2DM as fixed effects, and baseline body weight as a covariate.

A logistic regression with fixed categorical effects of region, T2DM status and treatment, will be used to analyse the occurrence of "Relative reduction in liver fat content of at least 30% from baseline to Week 48 assessed by MRI-PDFF (yes/no)". Missing continuous values of MRI-PDFF liver fat at Week 48 will be imputed using the multiple imputation method from Week 28 as described above and then dichotomized. If Week 28 is missing as well, the participant will be imputed as non-responder.

The ability to run, and the variance of the pre-specified retrieved dropout primary analysis model in the first step is extremely dependent upon the proportion of off-treatment measurements at Week 48 that are missing. Consequently, although every effort will be made to ensure that as much off-treatment data as possible is collected, if the proportion of off-treatment data at Week 48, that is collected, is below 40%, then the 'follow the reference' strategy will instead be adopted as the primary analysis (main estimation) and the retrieved dropout analysis will be used as sensitivity analysis. According to the 'follow the reference' strategy, after ICE 'treatment discontinuation', trial participants in the experimental arms are assumed to have an effect similar to that of the placebo arm while preserving information of any on-treatment and off-treatment data.

Analysis relative to the Efficacy Estimand:

MMRM with treatment, visit, treatment by visit interaction, region, T2DM status, and region by visit interaction, as well as baseline by visit interaction as fixed effects, and baseline as a covariate will be used for analysis of the continuous primary endpoint.

A logistic regression with fixed categorical effects of region, T2DM status, treatment, and the fixed continuous effect of baseline will be used to analyse the occurrence of "Relative reduction in liver fat content of at least 30% from baseline to Week 48 assessed by MRI-PDFF (yes/no)". Missing values will be imputed using the imputed values from MMRM analysis and then dichotomized.

Proprietary confidential information © 2023 Boehringer Ingelheim International GmbH or one or more of its affiliated companies

7.2.3.2 Sensitivity Analyses

Sensitivity analyses will be specified in the TSAP.

[redacted]

7.2.3.4 Supplementary Analyses

Two estimands have been defined in this protocol. Supplementary estimands will be specified in the TSAP, if requested by regulatory authorities.

7.2.4 Secondary objective analyses

Secondary objectives are listed in Section 2.1.3. Analyses for secondary efficacy endpoints follow those for the co-primary endpoints described above.

7.2.5 Further objective analyses

Further objectives are listed in Section 2.2.1. Analyses for further efficacy endpoints follow those for the co-primary endpoints described above.

[redacted]

7.2.6 Safety analyses

AEs will be coded using the Medical Dictionary for Drug Regulatory Activities (MedDRA). Standard Boehringer Ingelheim summary tables and listings will be produced. All AEs with an onset between start of treatment and end of the REP, a period of 28 days after the last dose of trial IMP, will be assigned to the on-treatment period for evaluation.

All treated trial participants will be included in the safety analysis. In general, safety analyses will be descriptive in nature and will be based on Boehringer Ingelheim standards. No hypothesis testing is planned.

Frequency, severity, and causal relationship of AEs will be tabulated by system organ class and preferred term after coding according to the current version of the Medical Dictionary for Drug Regulatory Activities (MedDRA) at database lock.

Proprietary confidential information © 2023 Boehringer Ingelheim International GmbH or one or more of its affiliated companies

Laboratory data will be analysed both quantitatively as well as qualitatively. The latter will be done via comparison of laboratory data to their reference ranges. Values outside the reference range as well as values defined as clinically relevant will be summarised. Treatment groups will be compared descriptively with regard to distribution parameters as well as with regard to frequency and percentage of trial participants with abnormal values or clinically relevant abnormal values.

Vital signs, physical examinations, or other safety-relevant data observed at screening, baseline, during the course of the trial, and at the end-of-trial evaluation will be assessed with regard to possible changes compared with findings before the start of treatment.

Further details will be provided in the TSAP.

7.2.7 Other Analyses

Details on other analyses will be described in TSAP, if needed.

[redacted]

7.3 HANDLING OF MISSING DATA

There will be no imputation for safety data. For trial participants who discontinue the IMP prematurely, all efforts will be made to follow trial participants for vital status and for any other endpoints including the primary endpoint, until EOS.

For the "treatment-regimen" estimand, missing data will be multiply imputed by a 'retrieved dropout' approach as described in Section 7.2.3.

For the "efficacy" estimand, missing data will be imputed using an MMRM as the imputation model.

7.4 RANDOMISATION

Boehringer Ingelheim will arrange for the randomisation and the packaging and labelling of trial IMP. The randomisation list will be generated using a validated system, which involves a pseudo-random number generator so that the resulting treatment will be both reproducible and non-predictable. Specific parameters used for the creation of the randomization schedule (e.g. block size or biasing coin probabilities) will be documented in the Clinical Trial Report. Access to the codes will be controlled and documented.

Trial participants will be randomised in blocks using a 2:1 allocation to 1 of 2 double-blind treatment groups. Randomisation will be performed at the start of the dose escalation period at Visit 2 (starting dose and timing of dose escalation as outlined in Table 4), with a 2:1 allocation to survodutide 6.0 mg once weekly or placebo once weekly.

Randomisation will be stratified by the presence of T2DM.

In addition, an adjustable screening cap will be implemented in the IRT system to ensure that enough male and female trial participants are enrolled and randomised. Cap for number of female trial participants is approximately 70%.

Proprietary confidential information © 2023 Boehringer Ingelheim International GmbH or one or more of its affiliated companies

7.5 DETERMINATION OF SAMPLE SIZE

Participants will be randomised in a 2:1 ratio to survodutide 6.0 mg or placebo. It is planned to randomise approximately 160 participants. The sample size needed to show superiority of survodutide over placebo for the co-primary endpoints was calculated under the following assumptions:

- Relative reduction in liver fat content of at least 30% from baseline to Week 48 assessed by MRI-PDFF (yes/no)
- Placebo effect: 30%
- Survodutide effect: 60%
- 95% power
- Alpha = 0.05 two-sided

The assumed response rates include a 10-15% non-responder rate for participants that prematurely discontinue and MRI-PDFF data cannot be imputed otherwise, which are therefore handled as non-responders.

On the basis of these assumptions, randomising 156 participants (104 to survodutide and 52 to placebo) will provide 95% power to establish superiority for survodutide compared with placebo at a 2-sided significance level of 0.05.

- Relative (%) change in body weight from baseline to Week 48
- Placebo effect: 3%
- Survodutide effect: 10% (assumed in a mixed population with and without T2DM; 12% in participants without T2DM, 8% in participants with T2DM, assumed ratio 1:1)
- SD=10% for survodutide and SD=6% for placebo
- 95% power
- Alpha=0.05
- 15% of participants discontinue treatment permanently and assumed to have same effect as participants who completed the trial on placebo

[redacted]

On the basis of these assumptions, randomising 96 participants (64 to survodutide and 32 to placebo) will provide 95% power to establish superiority for a survodutide dose compared with placebo at a 2-sided significance level of 0.05. The t-test on the mean difference assuming unequal variances and unequal allocation ratio was used for calculation.

Proprietary confidential information © 2023 Boehringer Ingelheim International GmbH or one or more of its affiliated companies

Given these assumptions, a sample size of approximately 160 participants (at least 156; 104 randomised to survodutide and 52 to placebo) would be required to test both co-primary endpoints. Both sample sizes were done on 95% to have an overall power of 90%.

Calculations were performed using nQuery Advisor® 6.1 statistical package by Statistical Solutions Ltd. The assumptions for the sample size calculations are based on the results from the STEP 1 trial [R21-1460], STEP 2 trial [R21-1465], Phase II trial 1404-0036 [c31754142-05] and [R23-4361].

Proprietary confidential information © 2023 Boehringer Ingelheim International GmbH or one or more of its affiliated companies

8. INFORMED CONSENT, TRIAL RECORDS, DATA PROTECTION, PUBLICATION POLICY, AND ADMINISTRATIVE STRUCTURE

The trial will be carried out in compliance with the protocol, the ethical principles laid down in the Declaration of Helsinki, in accordance with the ICH Harmonized Guideline for GCP, relevant Boehringer Ingelheim SOPs, EU regulation 536/2014, and other relevant regulations. Investigators and site staff must adhere to these principles. Deviation from the protocol, the principles of ICH GCP, or applicable regulations will be treated as 'protocol deviation'.

Standard medical care (prophylactic, diagnostic, and therapeutic procedures) remains the responsibility of the treating physician of the trial participant.

The investigator will inform the sponsor or delegate immediately of any urgent safety measures taken to protect the trial participants against any immediate hazard, as well as of any serious breaches of the protocol or of ICH GCP.

The Boehringer Ingelheim transparency and publication policy can be found on the following web page: trials.boehringer-ingelheim.com. The rights of the investigator and of the sponsor with regard to publication of the results of this trial are described in the investigator contract. As a rule, no trial results should be published prior to finalisation of the clinical trial report.

The certificate of insurance cover is made available to the investigator and the trial participants and is stored in the ISF.

8.1 TRIAL APPROVAL, PATIENT INFORMATION, INFORMED CONSENT

This trial will be initiated only after all required legal documentation has been reviewed and approved by the respective Institutional Review Board (IRB)/Independent Ethics Committee (IEC) and competent authority (CA) according to national and international regulations. The same applies for the implementation of changes introduced by amendments.

Prior to participation in the trial, written informed consent must be obtained from each participant according to ICH GCP and the regulatory and legal requirements of the participating country. Electronic systems will be used to support the consent process (eConsent) where it is permissible. Each signature must be personally dated by each signatory and the informed consent form and any additional participant-information form retained by the investigator as part of the trial records. A signed copy of the informed consent and any additional participant information must be given to each participant.

The trial participant must be given sufficient time to consider participation in the trial. The investigator or delegate obtains written consent of the trial participant's own free will with the informed consent form after confirming that the trial participant understands the contents. The investigator or delegate must sign (or place a seal on) and date the informed consent form. If a trial collaborator has given a supplementary explanation, the trial collaborator also signs (or places a seal on) and dates the informed consent.

Re-consenting may be necessary when new relevant information becomes available and should be conducted according to the sponsor's instructions.

The consent and re-consenting process should be properly documented in the source documentation.

Proprietary confidential information © 2023 Boehringer Ingelheim International GmbH or one or more of its affiliated companies

8.2 DATA QUALITY ASSURANCE

A risk-based approach is used for trial quality management. It is initiated by the assessment of critical data and processes for trial participant protection and reliability of the results, as well as identification and assessment of associated risks. An Integrated Quality and Risk Management Plan or alternative plan, in line with the guidance provided by ICH Q9 and ICH GCP E6, documents the rationale and strategies for risk management during trial conduct including monitoring approaches, vendor management, and other processes focusing on areas of greatest risk.

Continuous risk review and assessment may lead to adjustments in trial conduct, trial design, or monitoring approaches.

A quality assurance audit/inspection of this trial may be conducted by the sponsor, sponsor's designee, IRB/IEC, or regulatory authorities. The quality assurance auditor will have access to all medical records, the investigator's trial-related files and correspondence, and the informed consent documentation of this clinical trial.

8.3 RECORDS

CRFs for individual trial participants will be provided by the sponsor. See Section 4.1.5.2 for rules about emergency code breaks. For drug accountability, refer to Section 4.1.8.

8.3.1 Source documents

A detailed description of the transmission of electronic data (i.e. data flow) is provided in the data management plan.

In accordance with regulatory requirements, the investigator should prepare and maintain adequate and accurate source documents and trial records that include all observations and other data pertinent to the investigation on each trial participant. Source data as well as reported data should follow the "ALCOA principles" and be **a**ttributable, **l**egible, **c**ontemporaneous, **o**riginal, and **a**ccurate. Changes to the data should be traceable (audit trail).

Data reported on the CRF must be consistent with the source data, or the discrepancies must be explained.

The current medical history of the participant may not be sufficient to confirm eligibility for the trial, and the investigator may need to request previous medical histories and evidence of any diagnostic tests. In this case, the investigator must make at least one documented attempt to retrieve previous medical records. If this fails, a verbal history from the participant, documented in their medical records, would be acceptable.

Copies of source documents necessary (e.g. for adjudication of events and ECG central reading) will be provided to the corresponding vendor qualified by the sponsor for these assessments. Before sending or uploading those copies, the investigator must ensure that all participant identifiers (e.g. participant's name, initials, address, phone number, social security number) have been properly removed or redacted from all copies of the participant's source documents.

If the trial participant is not compliant with the protocol, any corrective action (e.g. re-training) must be documented in the participant file.

Proprietary confidential information © 2023 Boehringer Ingelheim International GmbH or one or more of its affiliated companies

For the CRF, data must be derived from source documents, for example:

- Participant identification: sex, year of birth (in accordance with local laws and regulations)
- Participant participation in the trial (substance, trial number, participant number, date participant was informed)
- Dates of participant's visits, including dispensing of trial IMP
- Medical history (including trial indication and concomitant diseases, if applicable)
- Medication history
- AEs and outcome events (onset date [mandatory] and end date [if available])
- SAEs (onset date [mandatory] and end date [if available])
- Concomitant therapy (start date, changes)
- Originals or copies of laboratory results and other imaging or testing results, with proper documented medical evaluation (in validated electronic format, if available)
- Completion of participant's participation in the trial (end date; in case of premature discontinuation, document the reason)
- Prior to allocation of a trial participant to a treatment in a clinical trial, there must be documented evidence in the source data (e.g. medical records) that the trial participant meets all inclusion criteria and does not meet any exclusion criteria. The absence of records (either medical records, verbal documented feedback of the participant, or testing conducted specific for a protocol) to support inclusion/exclusion criteria does not make the participant eligible for the clinical trial

8.3.2 Direct access to source data and documents

The investigator/institution will allow site trial-related monitoring, audits, IRB/IEC review, and regulatory inspections. Direct access must be provided to the CRF and all source documents/data, including progress notes and copies of laboratory and medical test results, which must always be available for review by the CRA, auditor, and regulatory inspector (e.g. FDA). They may review all CRFs and informed consent forms. The accuracy of the data will be verified by direct comparison with the source documents described in Section 8.3.1. The sponsor or delegate will also monitor compliance with the protocol and GCP.

In the event of force majeure or other disrupting circumstance (e.g. pandemic or war; see Section 6), site access may be restricted, thus limiting the ability to perform standard site monitoring activities on site, such as source data review and source data verification. Therefore, some of these activities may be performed remotely or replaced by centralized monitoring to the extent possible, based on a documented risk assessment and in alignment with local regulations.

8.3.3 Storage period of records

Trial sites: The trial sites must retain the source and essential documents (including ISF) according to the contract or local requirements valid at the time of the end of the trial (whichever is longer).

Sponsor: The sponsor must retain the essential documents according to the sponsor's SOPs.

Proprietary confidential information © 2023 Boehringer Ingelheim International GmbH or one or more of its affiliated companies

8.4 EXPEDITED REPORTING OF ADVERSE EVENTS

Boehringer Ingelheim is responsible for fulfilling their legal and regulatory reporting obligation in accordance with regulatory requirements.

8.5 STATEMENT OF CONFIDENTIALITY AND TRIAL PARTICIPANT PRIVACY

Data protection and data security measures are implemented for the collection, storage and processing of trial participant data in accordance with the principles 7 and 12 of the WHO GCP handbook.

To ensure confidentiality of records and personal data, only pseudonymised data will be transferred to the sponsor by using a participant identification number instead of the trial participant's name. The code is only available at the site and must not be forwarded to the sponsor. In case participant's records will be forwarded e.g. for SAE processing or adjudication committees, personal data that can identify the trial participant will be redacted by the site prior to forwarding. Access to the participant files and clinical data is strictly limited: personalised treatment data may be given to the trial participant's personal physician or to other appropriate medical personnel responsible for the trial participant's welfare. Data generated at the site as a result of the trial need to be available for inspection on request by the participating physicians, the sponsor's representatives, by the IRB/IEC and the regulatory authorities.

A potential data security breach will be assessed regarding the implications for rights and privacy of the affected person(s). Immediate actions as well as corrective and preventive actions will be implemented. Respective regulatory authorities, IRBs/IECs and trial participants will be informed as appropriate.

8.5.1 Collection, storage, and future use of biological samples and corresponding data

Measures are in place to comply with the applicable rules for the collection, biobanking, and future use of biological samples and clinical data, in particular:

- Sample and data usage must be in accordance with the separate biobanking informed consent
- The Boehringer Ingelheim internal facilities storing biological samples from clinical trial participants, as well as the external banking facility, are qualified for the storage of biological samples collected in clinical trials
- An appropriate sample and data management system, including audit trail for clinical data and samples to identify and destroy such samples according to ICF, is in place
- A fit for the purpose documentation (biomarker proposal, analysis plan and report) ensures compliant usage
- A fit for purpose approach will be used for assay/equipment validation depending on the intended use of the biomarker data

Proprietary confidential information © 2023 Boehringer Ingelheim International GmbH or one or more of its affiliated companies

- Samples and/or data may be transferred to third parties and other countries as specified in the biobanking ICF

8.6 TRIAL MILESTONES

The **first act of recruitment** represents the **start of the trial** and is defined as the date when the first trial participant in the whole trial signs informed consent.

The end of the trial is defined as the date of the last visit of the last trial participant in the whole trial ("last participant completed").

The "**last participant last treatment"** (LPLT) date is defined as the date on which the last trial participant in the whole trial is administered the last dose of trial IMP (as scheduled per protocol or prematurely). Individual investigators will be notified of SUSARs occurring with the trial IMP until 30 days after LPLT at their site.

Early termination of the trial is defined as the premature termination of the trial due to any reason before the end of the trial as specified in this protocol.

Temporary halt of the trial is defined as any unplanned interruption of the trial by the sponsor with the intention to resume it.

Suspension of the trial is defined as an interruption of the trial based on a Health Authority request.

The sponsor will prepare a clinical trial report within 1 year from the end of trial (6 months for paediatric trials).

The sponsor will notify the IEC/competent authority in each participating EU member state about all required trial milestones according to the respective laws.

A final report of the clinical trial data will be written only after all trial participants have completed the trial in all countries (EU or non-EU) to incorporate and consider all data in the report.

The sponsor will submit to the EU database a summary of the final trial results within 1 year from the end of a clinical trial as a whole, regardless of the country of the last trial participant (EU or non-EU).

8.7 ADMINISTRATIVE STRUCTURE OF THE TRIAL

The trial is sponsored by Boehringer Ingelheim.

A Coordinating Investigator is responsible to coordinate investigators at the different sites participating in this trial. Tasks and responsibilities are defined in a contract.

An Executive Committee consisting of independent experts and sponsor representatives will be established to support the sponsor in designing the trial and successful execution. A separate Executive Committee Charter will describe the tasks and responsibilities of the Committee.

A DMC will be established. Members of the DMC are independent of BI, they are physicians experienced in the treatment of the disease under investigation and a statistician. The DMC will assess the progress of the trial, including an unblinded safety assessment at specified intervals. The DMC will receive urgent significant safety concerns for immediate evaluation.

Proprietary confidential information © 2023 Boehringer Ingelheim International GmbH or one or more of its affiliated companies

While DMC members are unblinded, measures are in place to ensure the blinding for everyone else involved in the trial. Regular DMC meetings will be held at specified intervals. The DMC will recommend to the sponsor continuation, modification, or termination of the trial as detailed in the DMC charter. DMC recommendations as well as the final BI decision will be reported to the appropriate RAs/HAs, IRBs/IECs, and to investigators as requested by local law. The tasks and responsibilities of the DMC are specified in a DMC Charter.

Relevant documentation on the participating (Principal) Investigators (e.g. their *curricula vitae*) will be filed in the ISF.

An independent (external) and blinded CEC will be established for prospective adjudication of major adverse CV events (including non-fatal MI, non-fatal stroke, ischaemia related coronary revascularisation, and CV death), non-CV death, HF events (including hospitalisation for HF, emergency room visit, urgent care visit, or urgent outpatient HF visit), hospitalisation for unstable angina leading to coronary artery revascularisation, hospitalisation for coronary artery revascularisation, pancreatitis, and thyroid and pancreatic cancer. Composition of the CEC, its procedures, and interactions will be clarified in a CEC Charter.

The trial will be managed by a FOT vendor based on a contract which specifies the delegated responsibilities and duties. Boehringer Ingelheim has appointed a Clinical Trial Leader responsible for coordinating all required activities, in order to:

- Oversee and manage the trial in accordance with applicable regulations and SOPs
- Direct the clinical trial team and oversee the FOT vendor in the preparation, conduct, and reporting of the trial
- Ensure appropriate training

In the participating countries, the trial will be performed by a CRO based on a contract. The CRO will perform project management, clinical field monitoring, medical monitoring, reporting, and data management.

Statistical evaluation will be done by Boehringer Ingelheim according to Boehringer Ingelheim SOPs.

Tasks and functions assigned in order to organise, manage, and evaluate the trial are defined according to Boehringer Ingelheim SOPs. A list of responsible persons and relevant local information can be found in the ISF.

A central laboratory service, PK and immunogenicity laboratory services, a central ECG service, an IRT vendor, an MRI vendor, [redacted] will be used in this trial. Details will be provided in the IRT manual and central laboratory manual, available in the ISF.

Proprietary confidential information © 2023 Boehringer Ingelheim International GmbH or one or more of its affiliated companies

9. REFERENCES

9.1 PUBLISHED REFERENCES

P09-12413 Guidance for industry: drug-induced liver injury: premarketing clinical evaluation (July 2009).

P22-05567 Katsiki N, Ferrannini E, Mantzoros C. New American Diabetes Association (ADA)/European Association for the Study of Diabetes (EASD) guidelines for the pharmacotherapy of type 2 diabetes: placing them into a practicing physician's perspective. Metabolism; 2020; 107; 154218

P22-07379 Rosenstock J, Blueher M, Schmid B, Hoefler J, Hennige A. Multiple dose-ranging study of the novel glucagon/GLP-1 receptor dual agonist BI 456906 vs. placebo and open-label weekly semaglutide reference control in type 2 diabetes. Diabetologia; 2022; 65 (Suppl 1); Abstr 613; S314-S315.

P22-08755 Rosenstock J, Blueher M, Schoelch C, Hoefler J, Hennige A. Glucagon/GLP-1 Receptor Dual Agonist BI 456906 Reduces Body Weight in Patients with Type 2 Diabetes. Obesity (Silver Spring) 2022; 30 (Suppl 1): Abstr 30.

P23-02853 Yin X, Guo X, Liu Z, Wang J. Advances in the diagnosis and treatment of non-alcoholic fatty liver disease. Int J Mol Sci; 2023; 24; 2844

R00-0292 Buysse DJ, Reynolds CF, Monk TH, Berman SR, Kupfer DJ. The Pittsburgh sleep quality_index: a new instrument for psychiatric practice and research. Psychiatry Res 1989; 28, p. 193-213.

R11-4890 Sampson HA, Muñoz-Furlong A, Campbell RL, *et al.* Second symposium on the definition and management of anaphylaxis: summary report - second National Institute of Allergy and Infectious Disease/Food Allergy and Anaphylaxis Network symposium. J Allergy Clin Immunol 2006; 117(2):391-397.

R12-2378 Rubin DB. Multiple imputation for nonresponse in surveys. 1987

R16-0241 Kleiner DE, Brunt EM, Natta N van, Nonalcoholic Steatohepatitis Clinical Research Network. Design and validation of a histological scoring system for nonalcoholic fatty liver disease. Hepatology; 2005; 41(6); 1313-1321.

R17-3205 Lin ZH, Xin YN, Dong QJ, *et al.* Performance of the aspartate aminotransferase-to-platelet ratio index for the staging of hepatitis C-related fibrosis: an updated meta-analysis. Hepatology 2011; 53:726-36.

R17-3206 Sterling RK, Lissen E, Clumeck N, *et al.* Development of a simple noninvasive index to predict significant fibrosis patients with HIV/HCV co-infection. Hepatology 2006; 43:1317-1325.

[redacted]

Proprietary confidential information © 2023 Boehringer Ingelheim International GmbH or one or more of its affiliated companies

R18-1357 U.S. Department of Health and Human Services. Common terminology criteria for adverse events (CTCAE) version 5.0 (published 27 Nov 2017).

R20-1981 Regev A, Palmer M, Avigan MI, Dimick-Santos L, Treem WR, Marcinak JF, *et al.* Consensus: guidelines: best practices for detection, assessment and management of suspected acute drug-induced liver injury during clinical trials in patients with nonalcoholic steatohepatitis. Aliment Pharmacol Ther; 2019; 49; 702-713.

R20-1982 AM Sharma and RF Kushner. A proposed clinical staging system for obesity International Journal of Obesity 2009; (33):289–295.

R20-2315 Human energy requirements. Report of a joint FAO/WHO/UNU expert consultation. FAO: food and nutrition technical report

R21-1460 Wilding JPH, Batterham RL, Calanna S, Davies M, Gaal LF, van Lingvay I, *et al*. STEP 1 Study Group. Once-weekly semaglutide in adults with overweight or obesity. N Engl J Med 2021; 384(11), p. 989-1002.

R21-1465 Davies M, Faerch L, Jeppesen OK, Pakseresht A, Pedersen SD, Perreault L, *et al* STEP 2 Study Group. Semaglutide 2.4 mg once a week in adults with overweight or obesity, and type 2 diabetes (STEP 2): a randomised, double-blind, double-dummy, placebo-controlled, phase 3 trial. Lancet 2021; 397, p. 971-984.

R22-0131 Saxenda (liraglutide) injection, for subcutaneous use (Novo Nordisk) (U.S. prescribing information, revised: 12/2020).

R22-2292 Riazi K, Azhari H, Charette JH, *et al.* The prevalence and incidence of NAFLD worldwide: a systematic review and meta-analysis. Lancet Gastroenterol Hepatol; 2022

R22-2295 Loomba R, Friedman SL, Shulman GI. Mechanisms and disease consequences of nonalcoholic fatty liver disease. Cell; 2021; 184(10); 2537-2564.

R22-4277 American Diabetes Association Professional Practice Committee. Glycemic targets: standards of medical care in diabetes - 2022. Diabetes Care; 2022; 45(Suppl 1); S83-S96.

R23-0537 Rinella ME, Neuschwander-Tetri BA, *et al.* AASLD practice guidance on the clinical assessment and management of nonalcoholic fatty liver disease. Hepatology; 2023

R23-1195 Younossi ZM, Golabi P, Paik JM, Henry A, Dongen C van, Henry L. The global epidemiology of nonalcoholic fatty liver disease (NAFLD) and nonalcoholic steatohepatitis (NASH): a systematic review. Hepatology; 2023; 77(4); 1335-1347.

R23-1196 Lu FB, Hu ED, Xu LM, *et al.* The relationship between obesity and the severity of non-alcoholic fatty liver disease: systematic review and meta-analysis. Expert Rev Gastroenterol Hepatol; 2018; 12(5); 491-502.

Proprietary confidential information © 2023 Boehringer Ingelheim International GmbH or one or more of its affiliated companies

R23-1197 Fu CE, Ng CH, Yong JN, *et al.* A meta-analysis on associated risk of mortality in nonalcoholic fatty liver disease. Endocr Pract; 2023; 29; 33-39.

R23-1198 Younossi Z, Aggarwal P, Shrestha I, *et al.* The burden of non-alcoholic steatohepatitis: a systematic review of health-related quality of life and patient-reported outcomes. JHEP Rep; 2022; 4(9); 100525

R23-1199 Mantovani A, Petracca G, Beatrice G, *et al.* Non-alcoholic fatty liver disease and risk of incident chronic kidney disease: an updated meta-analysis. Gut; 2022; 71; 156-162.

R23-1377 Younossi Z, Alkhouri N, Cusi K, *et al.* A practical use of noninvasive tests in clinical practice to identify high-risk patients with nonalcoholic steatohepatitis. Aliment Pharmacol Ther; 2023; 57; 304-312.

R23-1378 Zhou H, Luo P, Li P, *et al.* Bariatric surgery improves nonalcoholic fatty liver disease: systematic review and meta-analysis. Obes Surg; 2022; 32; 1872-1883.

R23-1379 Stine JG, Munaganuru N, Barnard A, *et al.* Change in MRI-PDFF and histologic response in patients with nonalcoholic steatohepatitis: a systematic review and meta-analysis. Clin Gastroenterol Hepatol; 2021; 19(11); 2274-2283.

R23-1380 Tamaki N, Munaganuru N, Jung J, *et al.* Clinical utility of 30% relative decline in MRI-PDFF in predicting fibrosis regression in non-alcoholic fatty liver disease. Gut; 2022; 71; 983-990.

R23-1381 Albert SG, Wood EM. Meta-analysis of trials in non-alcoholic fatty liver disease with therapeutic interventions for metabolic syndrome. Diabetes Metab Syndr; 2021; 15; 102232

R23-1382 Vilar-Gomez E, Martinez-Perez Y, Calzadilla-Bertot L, *et al.* Weight loss through lifestyle modification significantly reduces features of nonalcoholic steatohepatitis. Gastroenterology; 2015; 149(2); 367-378.e5.

R23-1383 Rinella ME, Tacke F, Sanyal AJ, Anstee QM, AASLD/EASL Workshop Report on the AASLD/EASL joint workshop on clinical trial endpoints in NAFLD. Hepatology; 2019; 70(4); 1424-1436.

R23-1384 Koutoukidis DA, Koshiaris C, Henry JA, *et al.* The effect of the magnitude of weight loss on non-alcoholic fatty liver disease: a systematic review and meta-analysis. Metabolism; 2021; 115; 154455

R23-1416 Quek J, Chan KE, Wong ZY, *et al.* Global prevalence of non-alcoholic fatty liver disease and non-alcoholic steatohepatitis in the overweight and obese population: a systematic review and meta-analysis. Lancet Gastroenterol Hepatol; 2023; 8; 20-30.

[redacted]

Proprietary confidential information © 2023 Boehringer Ingelheim International GmbH or one or more of its affiliated companies

[redacted]

R23-2419 Le P, Payne JY, Zhang L, *et al.* Disease State Transition Probabilities Across the Spectrum of NAFLD: A Systematic Review and Meta-Analysis of Paired Biopsy or Imaging Studies. Clin Gastroenterol Hepatol. 2023; 21 (5): 1154-1168.

[redacted]

R23-2688 Joshi GP, Abdelmalak BB, Weigel WA, *et al.* American Society of Anesthesiologists consensus-based guidance on preoperative management of patients (adults and children) on glucagon-like peptide-1 (GLP-1) receptor agonists. 09 Jun 2023. Available at: Website: asahq.org/about-asa/newsroom/news-releases/2023/06/american-society-of-anesthesiologists-consensus-based-guidance-on-preoperative.

R23-2769 Rinella ME, Lazarus JV, Ratziu V, et al, NAFLD Nomenclature Consensus Group. A multi-society Delphi consensus statement on new fatty liver disease nomenclature. Hepatology; 2023

R23-3760 Newsome PN, Sasso M, Deeks JJ, *et al.* FibroScan-AST (FAST) score for the non-invasive identification of patients with non-alcoholic steatohepatitis with significant activity and fibrosis: a prospective derivation and global validation study. Lancet Gastroenterol Hepatol; 2020; 5(4); 362-373.

R23-4361 Flint A, Andersen G, Hockings P, Johansson L, Morsing A, Palle MS, *et al*. Randomised clinical trial: semaglutide versus placebo reduced liver steatosis but not liver stiffness in subjects with non-alcoholic fatty liver disease assessed by magnetic resonance imaging. Aliment Pharmacol Ther, 2021; 54, p. 1150-1161; 2021.

Proprietary confidential information © 2023 Boehringer Ingelheim International GmbH or one or more of its affiliated companies

[redacted]

Proprietary confidential information © 2023 Boehringer Ingelheim International GmbH or one or more of its affiliated companies

10. APPENDICES

10.1 MANAGEMENT OF GASTROINTESTINAL SYMPTOMS

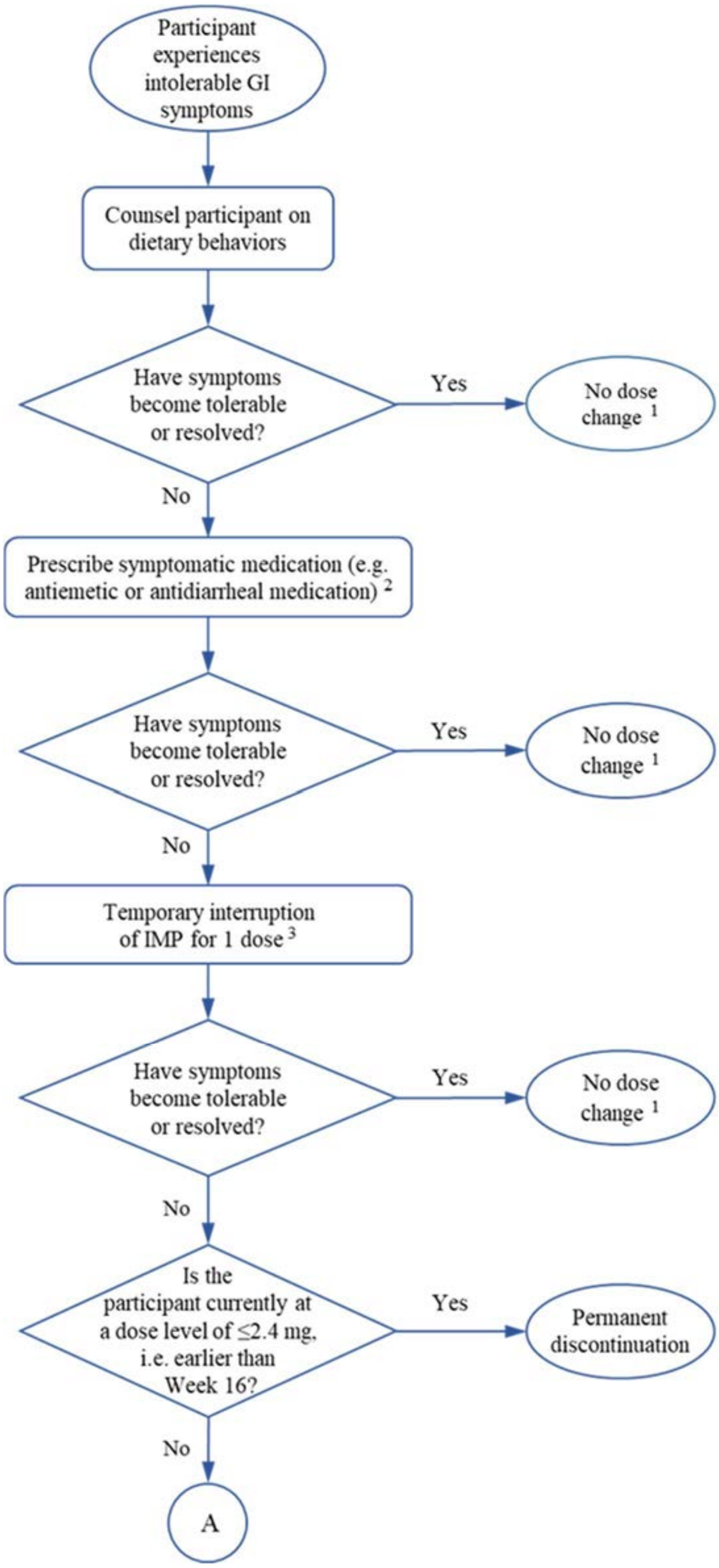

Figure 4 Management of GI symptoms

Proprietary confidential information © 2023 Boehringer Ingelheim International GmbH or one or more of its affiliated companies

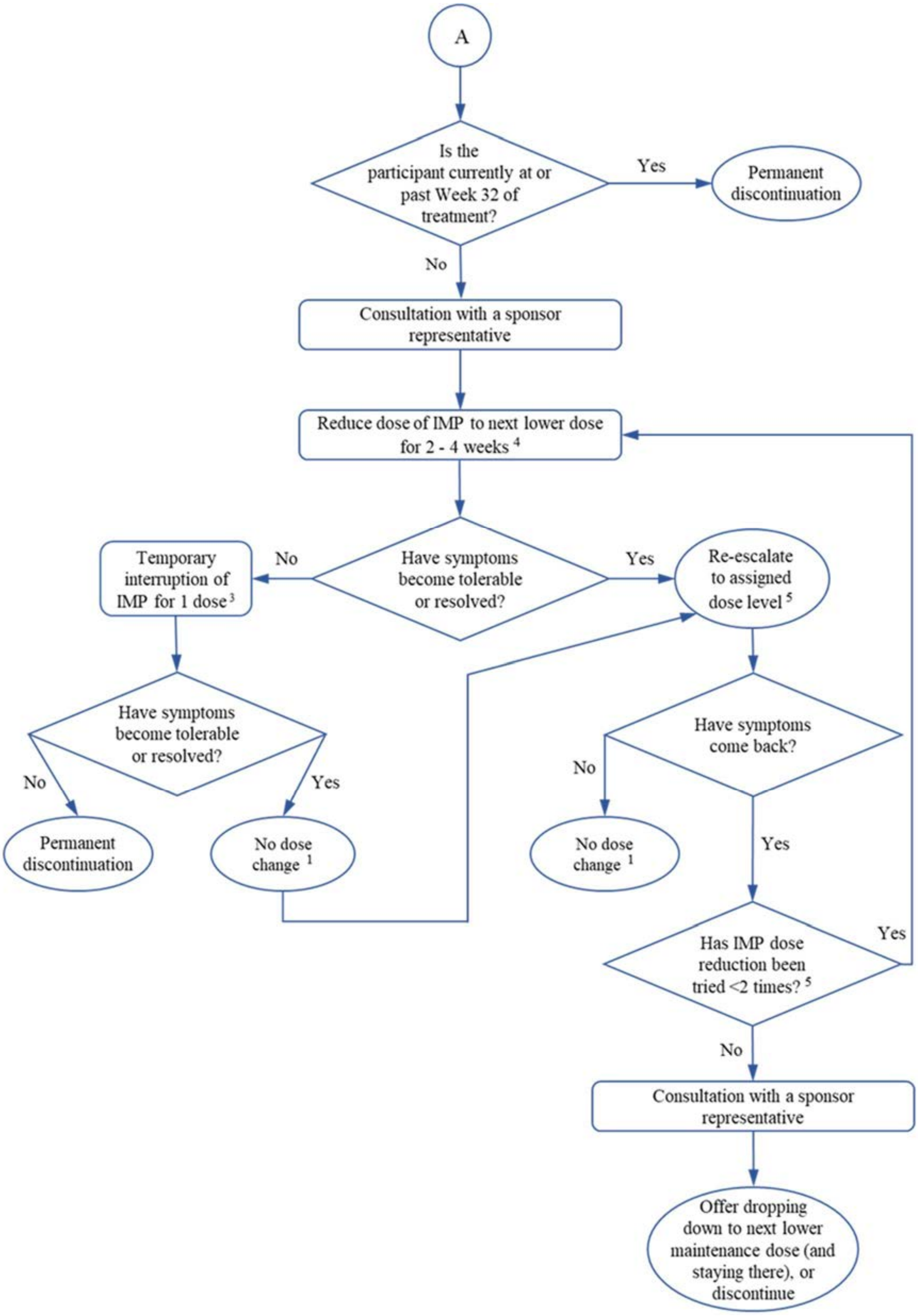

Figure 4 (cont'd) Management of GI symptoms

Proprietary confidential information © 2023 Boehringer Ingelheim International GmbH or one or more of its affiliated companies

[1]Continue with dose escalation if in the escalation period or continue in the same dose if in the maintenance period.

[2]Symptomatic medications (e.g. antiemetic or antidiarrheal medication) can be prescribed at any time point during escalation and maintenance period as deemed necessary by investigator.

[3]A temporary interruption of a second dose of the IMP may be allowed at the investigator's discretion, i.e. 2 consecutive doses are skipped.

[4] Between Weeks 16 and 32 of treatment, for participants who are not able to attain or stay at the maintenance dose of 6.0 mg due to GI symptoms, a dose reduction to 4.8 mg or 3.6 mg (or to 2.4 mg in exceptional circumstances) is possible **after consultation with a sponsor representative**. A dose reduction below 2.4 mg once per week is not allowed. A participant at the 2.4 mg dose or lower who requires a dose reduction will be discontinued from treatment. Thorough documentation of all measures taken, including outcomes of the discussion with sponsor representative, is required in a specific eCRF page in case of a dose reduction.

[5]As per investigator's discretion, it is recommended that the trial participant makes up to 2 attempts to re-escalate per dose escalation scheme to his/her assigned dose level.

Proprietary confidential information © 2023 Boehringer Ingelheim International GmbH or one or more of its affiliated companies

10.2 CONTRACEPTION METHODS ALLOWED IN THIS TRIAL

The following birth control methods (oral[1] and non-oral[2] contraceptives) may be considered as highly effective and allowed in this trial.

- Combined (oestrogen and progestogen containing) hormonal contraception associated with inhibition of ovulation
    - Oral[3]
    - Intravaginal
    - Transdermal (only for trial participants weighing less than 90 kg or 198 pounds)
- Progestogen-only hormonal contraception associated with inhibition of ovulation
    - Oral[3]
    - Injectable
    - Implantable
- Intrauterine device
- Intrauterine hormone-releasing system
- Bilateral tubal occlusion
- Vasectomised partner[4]
- Sexual abstinence[5]

[1]Oral contraceptives include combined (oestrogen and progestogen containing) or progesterone-only hormonal contraceptive pills associated with inhibition of ovulation.

[2]Non-oral contraceptives include combined hormonal contraception intravaginal or transdermal, progestogenonly hormonal contraception injectable or implantable, IUD, IUS, bilateral tubal occlusion, vasectomized partner, and sexual abstinence.

[3]Women who use oral contraceptives at screening should be advised to change to non-oral contraceptives (hormonal intravaginal, transdermal, injectable or implantable) at least 7 days prior to first dose of IMP or to add a barrier method of contraception during the entire treatment period with IMP and 28 days thereafter (preferably male condom). Women who initiate oral contraceptives at or after screening should be advised to continue using the previously applied contraception method for at least 7 days and use a barrier method of contraception in addition to the oral contraceptives during the entire treatment period with IMP and 28 days thereafter (preferably male condom).

[4]Vasectomised partner is a highly effective birth control method provided that partner is the sole sexual partner of the WOCBP trial participant and there is documented absence of sperm

[5]Sexual abstinence is considered a highly effective method only if defined as refraining from heterosexual intercourse. The reliability of sexual abstinence needs to be evaluated in relation to the duration of the clinical trial and the preferred and usual lifestyle of the trial participant. Periodic abstinence e.g. calendar, ovulation, symptothermal, post-ovulation methods; declaration of abstinence for the duration of exposure to IMP; and withdrawal are not acceptable.

Proprietary confidential information © 2023 Boehringer Ingelheim International GmbH or one or more of its affiliated companies

10.3 TIME SCHEDULE FOR PHARMACOKINETIC (PK) AND ANTI-DRUG ANTIBODY (ADA) / NEUTRALISING ANTIBODY (NAB) BLOOD SAMPLING

Table 14 Time Schedule for PK and ADA/NAb Sampling

Visit	Day	Time Point [hh:min]	PK Sampling	ADA/NAb Sampling
2	1 (+/-0)	- 00:30<br><br>Within 30 min before IMP administration	X	X
4	29 (+/-3)		X	X
6	57 (+/-3)		X	X
7	85 (+/-3)		X	X
10	169 (+/-3)		X	X
13	253 (+/-7)		X	X
End of Treatment	337 (+/-7)	168:00<br><br>Approximately 168 h (1 week) after last IMP administration	X	X
Follow Up	358 (+7)	672:00<br><br>Approximately 672 h (4 weeks) after last IMP administration	X	X

Proprietary confidential information © 2023 Boehringer Ingelheim International GmbH or one or more of its affiliated companies

10.4 CLINICAL STAGING OF OBESITY

Staging of obesity will be assessed by the investigator using the following staging system based on the medical history at screening.

Edmonton obesity staging system

Stage	Short description
**Stage 0**	**No apparent risk factors**
**Stage 1**	**Subclinical risk factors**: Presence of obesity-related subclinical risk factors (e.g. borderline hypertension, impaired fasting glucose, elevated liver enzymes, etc.), mild physical symptoms (e.g. dyspnoea on moderate exertion, occasional aches and pains, fatigue, etc.), mild psychopathology, mild functional limitations and/or mild impairment of well being
**Stage 2**	**Established complications**: Presence of established obesity-related chronic disease (e.g. hypertension, T2DM, sleep apnoea, osteoarthritis, reflux disease, polycystic ovary syndrome, anxiety disorder, etc.), moderate limitations in activities of daily living and/or well being
**Stage 3**	**End organ damage:** Presence of end organ damage such as MI, HF, diabetic complications, incapacitating osteoarthritis, significant psychopathology, significant functional limitations and/or impairment of well being
**Stage 4**	**End stage**: Having disabilities from obesity-related chronic diseases, severe disabling psychopathology, severe functional limitations and/or severe impairment of well being

Proprietary confidential information © 2023 Boehringer Ingelheim International GmbH or one or more of its affiliated companies

11. DESCRIPTION OF GLOBAL AMENDMENT(S)

This is the original protocol.

APPROVAL / SIGNATURE PAGE

Document Number: c43133006 **Technical Version Number:1.0**

Document Name: clinical-trial-protocol-version-01

Title: Multicenter, randomised, double-blind, placebo-controlled, 48 week, Phase III trial to evaluate the efficacy and safety of survodutide administered subcutaneously in participants with overweight or obesity and presumed or confirmed non-alcoholic steatohepatitis (NASH)

Signatures (obtained electronically)

Meaning of Signature	Signed by	Date Signed
Approval-Biostatistics	[redacted]	01 Dec 2023 14:10 CET
Approval-Team Member Medicine		01 Dec 2023 14:10 CET
Approval-Clinical Trial Leader		01 Dec 2023 14:11 CET
Verification-Paper Signature Completion		06 Dec 2023 17:26 CET

(Continued) Signatures (obtained electronically)

Meaning of Signature	Signed by	Date Signed

Boehringer Ingelheim

Clinical Trial Protocol

	**Document Number:**	**c43133006<br>228892_981335_5.0**
**EU Clinical Trial No.**<br>**Universal Trial Number**	2023-505303-23-00<br>U1111-1299-9925	
**BI Trial No.**	1404-0056	
**BI Investigational Medicinal Product**	Survodutide (BI 456906)	
**Title**	Multicentre, randomised, double-blind, placebo-controlled, 48-week, Phase III trial to evaluate the efficacy and safety of survodutide administered subcutaneously in participants with overweight or obesity and presumed or confirmed non-alcoholic steatohepatitis (NASH)	
**Lay Title**	A study to test whether survodutide helps people living with obesity or overweight and with a confirmed or presumed liver disease called non-alcoholic steatohepatitis (NASH) to reduce liver fat and to lose weight	
**Clinical Phase**	III	
**Clinical Trial Leader**	[redacted]<br>Telephone: [redacted]	
**Coordinating Investigator**	[redacted]<br>Telephone: [redacted]	
**Current Version and Date**	Version 5.0, 03 Jul 2025	
**Original Protocol Date**	01 Dec 2023	Page 1 of 154

Proprietary confidential information.
© 2025 Boehringer Ingelheim International GmbH or one or more of its affiliated companies. All rights reserved.
This document may not - in full or in part - be passed on, reproduced, published or otherwise used without prior written permission.

Proprietary confidential information © 2025 Boehringer Ingelheim International GmbH or one or more of its affiliated companies

CLINICAL TRIAL PROTOCOL SYNOPSIS

**Company name**	Boehringer Ingelheim
**Original protocol date**<br>**Latest revision date**	01 Dec 2023<br>03 Jul 2025
**Boehringer Ingelheim trial number**<br>**EU CT number**<br>**Universal Trial Number**	1404-0056<br>2023-505303-23-00<br>U1111-1299-9925
**Title of trial**	Multicentre, randomised, double-blind, placebo-controlled, 48-week, Phase III trial to evaluate the efficacy and safety of survodutide administered subcutaneously in participants with overweight or obesity and presumed or confirmed non-alcoholic steatohepatitis (NASH)
**Coordinating Investigator**	[redacted]<br>Telephone: [redacted]
**Trial site(s)**	Multicentre trial conducted in approximately 2-8 countries
**Clinical phase**	III
**Trial rationale**	Non-alcoholic fatty liver disease (NAFLD) and non-alcoholic steatohepatitis (NASH) are associated with obesity, dyslipidaemia, hypertension, insulin resistance, diabetes mellitus, and chronic kidney disease; its treatment represents a large unmet medical need. Weight loss is one of the most critical interventions for obesity and NAFLD/NASH, with a dose-response relationship between the magnitude of weight loss and improvement of liver inflammation, ballooning, and resolution of NAFLD or NASH. This trial investigates the effect of survodutide once weekly versus placebo on liver fat and body weight as an adjunct to a reduced-calorie diet and increased physical activity in participants with presumed or confirmed NASH and obesity (BMI ≥30 kg/m^2) or overweight (BMI ≥27 kg/m^2) with one or more weight-related co-morbidities.

Proprietary confidential information © 2025 Boehringer Ingelheim International GmbH or one or more of its affiliated companies

**Benefit-risk assessment and ethical considerations**	Trial participants treated with survodutide are expected to achieve larger body weight reduction and larger associated reduction in liver fat content, compared with those treated with placebo. The risks for participants caused by participation in the trial, including the trial procedures and exposure to the trial drug, are reasonably low and do not outweigh the potential benefits. The inclusion of placebo helps to better determine the difference between survodutide with lifestyle modification vs. lifestyle modification alone. Overall, the potential benefits coupled with an acceptable safety profile and a well-controlled clinical trial environment support the initiation of the trial.
**Trial objectives**	The primary objective is to demonstrate superiority of survodutide vs. placebo in:<br>• Reduction in liver fat content assessed by MRI-PDFF, defined as ≥30% from baseline at Week 48 (odds ratio, survodutide vs. placebo), AND<br>• The difference in adjusted means of relative change (%) in body weight from baseline to Week 48 (survodutide vs. placebo).<br>Secondary (exploratory) objectives are to demonstrate the superiority of survodutide vs. placebo in terms of the difference in adjusted means of changes from baseline to Week 48 in liver fat content assessed by MRI-PDFF, iron corrected T1 mapping (cT1), alanine aminotransferase (ALT) levels, waist circumference, Homeostasis Model Assessment - Insulin Resistance (HOMA-IR), liver stiffness assessed by magnetic resonance elastography (MRE), and liver volume assessed by MRI.<br>All treatment comparisons will be as randomised, including the effects of any early treatment discontinuation or any prohibited (anti-obesity) treatment, including GLP-1R agonists. In addition, treatments will be compared as randomised, but excluding effects of any early treatment discontinuation and any prohibited (anti-obesity) treatments.
**Trial endpoints**	Co-primary endpoints:<br>• Relative reduction in liver fat content of at least 30% from baseline to Week 48 (yes/no) assessed by MRI-PDFF, AND<br>• Relative change (%) in body weight from baseline to Week 48<br><br>Secondary endpoints:<br>• Absolute and relative change (%) from baseline to Week 48 in liver fat content assessed by MRI-PDFF [%]<br>• Reduction from baseline to Week 48 in cT1 [ms] levels of ≥80 ms (yes/no)<br>• Absolute and relative change from baseline to Week 48 in ALT [U/L] levels<br>• Absolute and relative change from baseline to Week 48 in waist circumference [cm]

Proprietary confidential information © 2025 Boehringer Ingelheim International GmbH or one or more of its affiliated companies

	• Absolute and relative change from baseline to Week 48 in HOMA-IR (FPI [mlU/L]·FPG [mmol/L]/22.5)<br>• Absolute and relative change from baseline to Week 48 in liver stiffness [kPa] assessed by MRE<br>• Absolute and relative change in liver volume [mL] from baseline to Week 48 measured using MRI
**Trial design**	Randomised, double-blind, placebo-controlled, 48-week, Phase III clinical trial with an optional extended treatment period of 4 weeks
**Total number of trial participants randomised**	Planned: Approximately 160<br>During the course of the trial, the number of participants increased to approx. 220 due to an unexpected sharp drop in the screening failure percentage following recruitment closure.
**Number of trial participants per treatment group**	Survodutide: at least 104 participants<br>Placebo: at least 52 participants
**Diagnosis, main inclusion and exclusion criteria**	Main diagnosis: presumed or confirmed NASH and overweight or obesity<br><br>Key inclusion criteria:<br>• Age ≥18 years at the time of signing informed consent, and at least the legal age of consent in countries where it is >18 years<br>• BMI ≥30 kg/m^2, OR BMI ≥27 kg/m^2 and at least one of the following weight-related comorbidities at screening:<br>o Hypertension (defined as repeated, i.e. at least 3 measurements in resting condition, SBP values of ≥140 mmHg and/or DBP values of ≥90 mmHg in the absence of anti-hypertensive treatment, or intake of at least 1 anti-hypertensive drug to maintain a normotensive blood pressure)<br>o Dyslipidaemia (defined as at least 1 lipid-lowering treatment required to maintain normal blood lipid levels, or low-density lipoprotein [LDL] cholesterol ≥160 mg/dL [≥4.1 mmol/L] or triglycerides ≥150 mg/dL [≥1.7 mmol/L], or high-density lipoprotein [HDL] cholesterol <40 mg/dL [<1.0 mmol/L] for men or HDL cholesterol <50 mg/dL [<1.3 mmol/L] for women)<br>o Obstructive sleep apnoea<br>o Cardiovascular disease (e.g. heart failure with New York Heart Association [NYHA] functional class II-III, history of ischaemic or haemorrhagic stroke or cerebrovascular revascularisation procedure [e.g. carotid endarterectomy and/or stent], MI, coronary artery disease, or peripheral vascular disease)<br>o T2DM (diagnosed at least 180 days prior to screening, with glycated haemoglobin [HbA_{1c}] ≥6.5% [48 mmol/mol] and

Proprietary confidential information © 2025 Boehringer Ingelheim International GmbH or one or more of its affiliated companies

	<10% [86 mmol/mol] as measured by the central laboratory at screening)<br>• Presumed/confirmed NASH: Evidence of hepatic steatosis (defined by an MRI-PDFF ≥8% at screening) with the exclusion of secondary causes of hepatic fat accumulation, such as significant alcohol consumption, other chronic liver diseases and/or steatogenic medications AND at least one of the following:<br>o MRE ≥2.61 and <4.68 kPa at screening, OR<br>o MAST score ≥0.242 at screening, OR<br>o FAST score ≥0.5 at screening, OR<br>o FibroScan® Vibration-Controlled Transient Elastography (VCTE™) ≥8 kPa and <20 kPa at screening, OR<br>o FIB-4 score ≥2.67 and <3.48 at screening, OR<br>o ELF score >7.7 and <11.3 at screening, OR<br>o cT1 ≥875 ms at screening, OR<br>o Recent liver biopsy (within 3 years of screening) consistent with NASH (defined as the presence of steatosis, inflammation, and ballooning) with stage ≤3 fibrosis according to the NASH Clinical Research Network classification<br>• History of at least one self-reported unsuccessful dietary effort to lose body weight<br><br>Key exclusion criteria:<br>• Current or history of significant alcohol consumption (defined as intake of >210 g/week in men and >140 g/week in women on average over a consecutive period of more than 3 months) or inability to reliably quantify alcohol consumption based on the investigator's judgement within the last 5 years.<br>• Intake of medications associated with liver injury, hepatic steatosis or steatohepatitis.<br>• History of other chronic liver diseases (e.g. viral hepatitis, autoimmune liver disease, primary biliary cholangitis, primary sclerosing cholangitis, Wilson's disease, hemochromatosis, A1At deficiency, history of liver transplantation). Hepatitis B and C testing will be done at Visit 1. Participants with positive HBsAg should be excluded. Participants treated for hepatitis C must have a negative RNA test at screening and also be HCV RNA negative for at least 3 years prior to screening in order to be eligible for the trial. Trial participants with positive HCV antibody and no history of HCV treatment require a negative HCV RNA test at screening to be eligible for the trial<br>• Cirrhosis based on clinical assessment, abdominal imaging, liver histology or non-invasive tests assessed at screening (ELF ≥11.3 or FIB4 ≥3.48 or FibroScan® VCTE™ ≥20 kPa or MRE ≥4.68 kPa), or history of cirrhosis

Proprietary confidential information © 2025 Boehringer Ingelheim International GmbH or one or more of its affiliated companies

	• Current decompensated liver disease or previous hepatic decompensation (ascites, spontaneous bacterial peritonitis, portal hypertension bleeding, hepatic encephalopathy, hepatorenal syndrome).<br>• Evidence of portal hypertension (e.g. splenomegaly, oesophageal varices, or other portosystemic collateral pathways).<br>• Any of the following liver laboratory test abnormalities at screening:<br>o Serum AST and/or ALT elevation ≥5x ULN<br>o Total serum bilirubin concentration ≥1.2x ULN (except for cases of known Gilbert's Syndrome)<br>o Alkaline phosphatase >2x ULN<br>o INR ≥1.3 (unless participant is on anticoagulants)<br>• Body weight variation (self-reported) >5% within 3 months before screening<br>• Medications for obesity within 3 months before screening<br>• Previous or planned (during the trial period) treatment for obesity with surgery or a weight loss device, or prior surgery of the gastrointestinal (GI) tract that could interfere with body weight. The following are allowed: (1) liposuction and/or abdominoplasty, if performed >1 year before screening, (2) lap banding, if the band has been removed >1 year before screening, (3) intragastric balloon, if the balloon has been removed >1 year before screening, (4) duodenal-jejunal bypass sleeve, if the sleeve has been removed >1 year before screening (5) appendectomy, (6) simple hernia repair, or (7) cholecystectomy.<br>• Obesity induced by other endocrinologic disorders (i.e. Cushing Syndrome) or diagnosed monogenetic or syndromic forms of obesity (i.e. melanocortin 4 receptor deficiency, leptin deficiency, or Prader Willi Syndrome)
**Trial intervention and test product**	Survodutide (BI 456906)
**Dose and mode of administration**	6.0 mg survodutide once weekly per subcutaneous (s.c.) injection
**Comparator product**	Placebo
**Dose and mode of administration**	Matching s.c. injection
**Duration of treatment**	Up to 52 weeks consisting of a main treatment period of 48 weeks with an optional extended treatment period of 4 weeks
**Statistical methods**	The primary objective will be addressed by the treatment-regimen estimand. The efficacy estimand is included for supplementary analyses. Contrary to the efficacy estimand, the treatment-regimen estimand tolerates certain anti-hyperglycaemia medication (rescue

Proprietary confidential information © 2025 Boehringer Ingelheim International GmbH or one or more of its affiliated companies

	therapy), certain glucose-lowering, anti-hypertensive, and lipid-lowering medication, and possibly anti-obesity medication.<br>Relative to the treatment-regimen estimand:<br>Analysis of covariance with multiple imputation of missing endpoint data and statistical inference over multiple imputation of missing data will be performed for the continuous primary endpoint of "relative (%) change in body weight from baseline to Week 48". Missing body weight data at Week 48 will be imputed based on 'retrieved dropouts', i.e. participants with Week 48 value in the same treatment arm who prematurely discontinued trial drug. If retrieved dropouts are insufficient to provide a reliable imputation model, multiple imputation will be based on the "follow the reference" strategy (i.e. participants in the survodutide arm who discontinue treatment are assumed to have a similar effect as the placebo arm while preserving information of any on-treatment and off-treatment data). The analysis will include treatment and region and T2DM as fixed effects, and baseline body weight as a covariate.<br>A logistic regression with fixed categorical effects of treatment, region and T2DM status at baseline will be used to analyse the primary endpoint of "Relative reduction in liver fat content of at least 30% from baseline to Week 48 assessed by MRI-PDFF (yes/no)". The responder/non-responder (yes/no) status will be based on the continuous liver fat measurements, or, in the case of missing liver fat values, on the continuous values imputed with the methods described in the previous paragraph.<br>Relative to the efficacy estimand:<br>MMRM with region, T2DM status at baseline, baseline body weight by visit interaction, and treatment by visit interaction, as fixed effects will be used for analysis of the continuous primary endpoint.<br>A logistic regression with fixed categorical effects of region, T2DM status at baseline, treatment, and the fixed continuous effect of baseline liver fat content assessed by MRI-PDFF will be used to analyse the occurrence of "Relative reduction in liver fat content of at least 30% from baseline to Week 48 assessed by MRI-PDFF (yes/no)". The responder/non-responder (yes/no) status will be based on the continuous liver fat measurements. Missing liver fat values will be addressed via multiple imputation and then dichotomized.

Proprietary confidential information © 2025 Boehringer Ingelheim International GmbH or one or more of its affiliated companies

SCHEDULE OF ASSESSMENTS

			**Dose escalation**								**Maintenance**					**Optional ETP**	**FU**	
	**Screening** (in-clinic)	**Randomisation** (in-clinic)	Remote	In-clinic	Remote	In-clinic	In-clinic	In-clinic	In-clinic	In-clinic	In-clinic	Remote	In-clinic	In-clinic	In-clinic	In-Clinic	In-clinic	In-clinic
**Visit[1]**	**V1**	**V2**	**V3**	**V4**	**V5**	**V6**	**V7**	**V8**	**V9**	**V10**	**V11**	**V12**	**V13**	**V14**	**V15/ EOT[10]**	**V16/ EOT[36]**	**ETD[10]**	**FU/ EOS[11]**
**Timing of visits (weeks)**	Up to -8	0	2	4	6	8	12	16	20	24	28	32	36	42	48	52		51 (55)
**Timing of visits (days)**	Up to -56	1	15	29	43	57	85	113	141	169	197	225	253	295	337	365		358 (386)
**Visit window (days)**		+/-0	+/-3	+/-3	+/-3	+/-3	+/-3	+/-3	+/-3	+/-3	+/-7	+/-7	+/-7	+/-7	+/-7	+/-7		+7
Informed consent[2]	X																	
Review of inclusion/exclusion criteria	X	X																
Demographics	X																	
Medical and weight history, baseline conditions[3]	X																	
Height	X																	
Weight[4]	X	X		X		X	X	X	X	X	X		X	X	X	X	X	X
Waist and hip circumference		X		X		X	X	X	X	X	X		X	X	X	X	X	X
Physical examination[5]	X	X[5]								X					X	X	X	
Substance usage	X	X								X					X		X	
Eye examination[6]	X									X					X		X	
Vital signs (SBP, DBP, pulse)[7]	X	X		X		X	X	X	X	X	X		X	X	X	X	X	X
12-lead ECG[8]	X	X		X		X	X	X	X	X			X		X	X	X	X
Concomitant therapy[9]	X	X	X	X	X	X	X	X	X	X	X	X	X	X	X	X	X	X
Assessment of obesity staging	X																	
Evaluation of lipid lowering treatment[12]		X		X		X	X	X	X	X	X		X	X	X		X	X
Evaluation of antihypertensive treatment[12]		X		X		X	X	X	X	X	X		X	X	X		X	X
All AEs/SAEs/AESIs[13] and check criteria for discontinuation	X	X	X	X	X	X	X	X	X	X	X	X	X	X	X	X	X	X

Proprietary confidential information © 2025 Boehringer Ingelheim International GmbH or one or more of its affiliated companies

			**Dose escalation**								**Maintenance**					**Optional ETP**	**FU**	
	**Screening** (in-clinic)	**Randomisation** (in-clinic)	Remote	In-clinic	Remote	In-clinic	In-clinic	In-clinic	In-clinic	In-clinic	In-clinic	Remote	In-clinic	In-clinic	In-clinic	In-Clinic	In-clinic	In-clinic
**Visit[1]**	**V1**	**V2**	**V3**	**V4**	**V5**	**V6**	**V7**	**V8**	**V9**	**V10**	**V11**	**V12**	**V13**	**V14**	**V15/ EOT[10]**	**V16/ EOT[36]**	**ETD[10]**	**FU/ EOS[11]**
**Timing of visits (weeks)**	Up to -8	0	2	4	6	8	12	16	20	24	28	32	36	42	48	52		51 (55)
**Timing of visits (days)**	Up to -56	1	15	29	43	57	85	113	141	169	197	225	253	295	337	365		358 (386)
**Visit window (days)**		+/-0	+/-3	+/-3	+/-3	+/-3	+/-3	+/-3	+/-3	+/-3	+/-7	+/-7	+/-7	+/-7	+/-7	+/-7		+7
Randomisation		X																
**Liver outcome assessments**																		
MRI-PDFF[14]	X										X				X			(X)
cT1[14]	X										X				X			(X)
MRE[14]	X										X				X			(X)
FibroScan® VCTE™ + CAP™[15]	X										X				X			(X)
**Laboratory tests[16]**																		
Safety laboratory tests[17]	X	X		X		X	X	X	X	X	X		X		X	X	X	X
Hs Troponin T		X					X				X				X		X	
HBV, HCV, HIV	X																	
HbA_{1c}	X	X					X				X		X		X		X	
FPG, FPI, C-peptide		X					X				X		X		X		X	
HOMA-IR		X					X				X		X		X		X	
[redacted]																		
Lipid tests: total cholesterol, HDL, LDL, VLDL, triglycerides, free fatty acids, ApoB		X					X				X		X		X		X	
Urinalysis[18]		X		X			X				X		X		X	X	X	
ELF score	X	X					X				X				X		X	
[redacted]																		

Proprietary confidential information © 2025 Boehringer Ingelheim International GmbH or one or more of its affiliated companies

			**Dose escalation**								**Maintenance**					**Optional ETP**	**FU**	
	**Screening** (in-clinic)	**Randomisation** (in-clinic)	Remote	In-clinic	Remote	In-clinic	In-clinic	In-clinic	In-clinic	In-clinic	In-clinic	Remote	In-clinic	In-clinic	In-clinic	In-Clinic	In-clinic	In-clinic
**Visit[1]**	**V1**	**V2**	**V3**	**V4**	**V5**	**V6**	**V7**	**V8**	**V9**	**V10**	**V11**	**V12**	**V13**	**V14**	**V15/ EOT[10]**	**V16/ EOT[36]**	**ETD[10]**	**FU/ EOS[11]**
**Timing of visits (weeks)**	Up to -8	0	2	4	6	8	12	16	20	24	28	32	36	42	48	52		51 (55)
**Timing of visits (days)**	Up to -56	1	15	29	43	57	85	113	141	169	197	225	253	295	337	365		358 (386)
**Visit window (days)**		+/-0	+/-3	+/-3	+/-3	+/-3	+/-3	+/-3	+/-3	+/-3	+/-7	+/-7	+/-7	+/-7	+/-7	+/-7		+7
Other biomarkers[20]		X					X				X				X			
Sampling for biobanking (optional)[2, 21]		X													X		X	
Sampling for PK, ADA, and NAb[22]		X		X		X	X			X			X		X	X	X	X
FSH[23]	X																	
Pregnancy testing[24]	X	X		X		X	X	X	X	X	X		X	X	X	X	X	X
**Questionnaires[25]**																		
[redacted]																		
PHQ-9	X	X					X			X			X		X		X	
C-SSRS	X	X	X	X	X	X	X	X	X	X	X	X	X	X	X		X	X
**Other assessments and procedures**																		
Training in/observe prefilled syringe administration[26]		X	X	X	X			X			X							
Hand out IFU		X																
IRT call and dispense IMP[27]	X[28]	X		X		X	X	X	X	X	X		X	X	(X)[35]			
eDiary review (IMP administration, injection site reactions, and compliance check)[29]			X	X	X	X	X	X	X	X	X	X	X	X	X	X	X	
Paper diary or local app review		X	X	X	X	X	X	X	X	X	X	X	X	X	X	X	X	
Diet and physical activity counselling[30]		X		X		X	X	X	X	X	X		X	X	X	X	X	
Hand out glucose monitoring device (if applicable) and participant materials[31]	X[32]	X																

Proprietary confidential information © 2025 Boehringer Ingelheim International GmbH or one or more of its affiliated companies

			**Dose escalation**								**Maintenance**					**Optional ETP**	**FU**	
	**Screening** (in-clinic)	**Randomisation** (in-clinic)	Remote	In-clinic	Remote	In-clinic	In-clinic	In-clinic	In-clinic	In-clinic	In-clinic	Remote	In-clinic	In-clinic	In-clinic	In-Clinic	In-clinic	In-clinic
**Visit[1]**	**V1**	**V2**	**V3**	**V4**	**V5**	**V6**	**V7**	**V8**	**V9**	**V10**	**V11**	**V12**	**V13**	**V14**	**V15/ EOT[10]**	**V16/ EOT[36]**	**ETD[10]**	**FU/ EOS[11]**
**Timing of visits (weeks)**	Up to -8	0	2	4	6	8	12	16	20	24	28	32	36	42	48	52		51 (55)
**Timing of visits (days)**	Up to -56	1	15	29	43	57	85	113	141	169	197	225	253	295	337	365		358 (386)
**Visit window (days)**		+/-0	+/-3	+/-3	+/-3	+/-3	+/-3	+/-3	+/-3	+/-3	+/-7	+/-7	+/-7	+/-7	+/-7	+/-7		+7
Hyper-/hypoglycaemic episode review (eDiary), if applicable[33]			X	X	X	X	X	X	X	X	X	X	X	X	X	X	X	X
[redacted]																		
Vital status																		X

Note: For in-clinic visits after randomisation and review of inclusion/exclusion criteria, the suggested order of assessments is described in Section 6.2.2. If remote visits are not allowed per local regulations, they will be performed as in-clinic visits. If remote visits are completed as in-clinic visits, only procedures designated for the remote visit should be completed in the clinic. EOT is 7 days after last dose, FU is 28 days after last dose.

1. If the IMP administration is scheduled on the day of the visit, the trial participant has to be informed to come to the study site prior to the IMP administration so that the PK, ADA, and NAb sample is obtained prior to IMP administration.
2. Separate informed consent forms are required for biobanking samples [redacted]
3. Medical history includes assessment of pre-existing conditions (special attention to be paid on medical conditions requested by in-/exclusion criteria and other important medical conditions, e.g. history of gallbladder disease, cardiovascular disease, history of psychiatric disorder, MTC, and history of any cancer other than MTC) In addition, if a mammogram or colonoscopy was performed within 2 years prior to screening as part of routine clinical practice, data should be recorded in the eCRF. Substance usage such as smoking/nicotine and alcohol will be captured as indicated.
4. Body weight must be measured in fasting state (except the screening visit). If the trial participant has not fasted, the participant should be called in for a new visit within the visit window. See Section 5.1.3.1.
5. The physical examination should be repeated if Visit 2 is performed more than 2 weeks after the screening visit.
6. The eye examination has to be performed in participants with diabetes (see Section 3.3.2 for diabetes criteria) in the period between screening and randomization, if not performed in the past 3 months before screening, to verify uncontrolled and potentially unstable diabetic retinopathy or maculopathy (Section 5.2.5.2). The eye examination at Visit 10 can be performed within a time window of +/- 1 month of the visit, and at the EOT/ETD Visit within a time

Proprietary confidential information © 2025 Boehringer Ingelheim International GmbH or one or more of its affiliated companies

window of -2 months/+14 days of the visit. Unless the eye examination at Visit 10 was performed within 3 months before the EOT/ETD Visit, the EOT eye examination must be performed on the EOT/ETD Visit with a time window of -2 months/+14 days of the visit.

7. Vital sign measurements should be taken before obtaining an ECG tracing and before collection of blood samples for laboratory testing per the instructions in Section 5.2.2.
8. ECG at screening should be recorded in triplicate (i.e three single ECGs recorded within 180 s). ECGs should be collected prior to collection of blood samples for laboratory testing, including PK samples. For the schedule of PK sampling see Section 10.4.
9. Special attention has to be paid if medication with a narrow therapeutic index is used. Refer to Section 4.2.2.1.
10. For trial participants who completed the planned treatment schedule up to Week 48/Visit 15, the EOT and Visit 15 should be combined in one visit (no duplicate assessments to be performed). Trial participants prematurely discontinuing IMP will have an ETD Visit 7 days after the last dose or as soon as possible. Both the ETD visit and Week 48/Visit 15 should be in-clinic; however as an exception they may also be performed by a site's HCP or vendor at the participant's home, if available and acceptable according to local law and regulations. Refer to Section 3.3.4.1 for more details. For trial participants who agree to enter an optional extended treatment period of 4 weeks after completion of the main treatment period of 48 weeks, the EOT visit page in the eCRF should be completed at Week 52/Visit 16 only.
11. The last regular visit is the FU/ EOS Visit. The FU/EOS Visit should be in-clinic, and a remote visit (if allowed per local regulations) should be done only as an exemption. For participants who discontinued the IMP prematurely and are willing to continue in the trial, the EOS at W51 should only include collection of vital status, AEs/AESIs/SAEs, concomitant medication and samples for ADA/Nab (also refer to Section 3.3.4.1).
12. Changes of the medication including dose in anti-hypertension and lipid-lowering medications have to be assessed for increase/decrease/no change and captured in the eCRF.
13. After the EOS Visit (i.e. the individual trial participant's EOS), the investigator should only report any occurrence of cancer of new histology, IMP related SAEs, and IMP related AESIs which the investigator may become aware of. Refer to Section 5.2.6.2.2.
14. MRI to assess MRI-PDFF, cT1 and MRE to be scheduled prior to Visit 2 (randomisation visit) only for trial participants who have met all inclusion criteria and none of the exclusion criteria. Refer to Section 6.2.1. The MRI assessment at EOT may be scheduled on a different day than the EOT day; however, it must still fall within the +/-7-day time window. Prior to MRE 4 h of fasting are required. The MRI assessment at FU/EOS Visit applies only to participants who discontinued treatment prematurely, according to the options presented in Section 3.3.4.1. Participants who discontinued IMP due to pregnancy are not to undergo any imaging assessments for trial purposes.
15. If CAP™ is not available, VCTE™ only to be performed. Prior to FibroScan® measurements, 3 h of fasting are required. The MRI assessment at FU/EOS Visit applies only to participants who discontinued treatment prematurely, according to the options presented in Section 3.3.4.1. Participants who discontinued IMP due to pregnancy are not to undergo any imaging assessments for trial purposes.
16. All in-clinic visits should be on fasting state (except for screening). Trial participants should be instructed to come after at least 8 h without food or liquid intake, except water, and without administering anti-hyperglycaemia medications (as applicable), which will be taken in the clinic. The IMP dose administration (if scheduled within the day of the visit) should be withheld in the morning of the clinic visit until blood sampling has been performed. If a lab parameter measured at screening in non-fasting state is out of normal range and thus prevents the participant from inclusion into the trial as per eligibility criteria, measurement can be repeated in fasting state within the screening period window.
17. All laboratory assessments will be performed by a central laboratory. Instruction regarding sample collection, sample handling/processing, and sample shipments will be provided in the respective laboratory manuals.

Proprietary confidential information © 2025 Boehringer Ingelheim International GmbH or one or more of its affiliated companies

18. Urine sample should be collected in the clinic from the second morning void (preferably) and if not possible, from spot urine. Refer to Section 5.2.3 for details.
19. [redacted].
20. Biomarker samples (refer to Section 5.4.1 for details) are to be taken before IMP administration and in a fasted state. If a participant has discontinued IMP prematurely biomarkers samples will not be taken after IMP discontinuation. If a sample is not taken as scheduled, this sample will be omitted and defined schedule of assessment should be followed.
21. A DNA sample for biobanking will only be collected once, preferably at Visit 2, while plasma/serum samples for biobanking will be collected at Visit 2 and at the Week 48/EOT (or ETD) visit. Samples will be taken prior to IMP administration. Refer to Section 5.5 for details.
22. If the IMP administration is scheduled during the day of the visit, then the trial participant will come to the study site prior to IMP administration and sampling for PK, ADA, and NAb will be done prior to IMP administration. IMP administration prior to PK sampling should be documented in trial participant's eDiary. Refer to Section 10.4 for the detailed sampling schedule. If a participant has discontinued the IMP prematurely, sampling is performed for ADA and NAb testing, but not for PK testing, except for ETD and FU/EOS Visits (if scheduled 28 days after the last IMP dose).
23. For women between age of 40 and 55 years with amenorrhea for 12 months, perform FSH test to confirm their menopausal status. If the participant is on HRT, HRT must be withheld intermittently to confirm the menopausal status via FSH testing before enrolment. If pausing HRT for this purpose is not possible or not accepted by the participant, this participant will have to use non-oestrogen hormonal highly effective contraception methods compatible with HRT. FSH testing is not required for women who are permanently sterile (for definition of being permanently sterile, refer to Section 3.3.2) or for women ≥55 years old with amenorrhea for at least 12 months without alternative medical cause other than being postmenopausal.
24. For WOCBP only. A urine pregnancy test will be done during screening. If it is positive, the participant will be considered a screen failure. If it is negative, a serum pregnancy test will be done. Urine pregnancy tests will be performed at all other in-clinic visits. If the urine pregnancy test is positive, IMP should be paused, and a serum pregnancy test will be done. If the serum pregnancy test is positive, the participant needs to be discontinued from IMP treatment. Pregnancy tests can be done more frequently if required by local regulations. Once a female participant discontinues the IMP prematurely, pregnancy testing will only be required during their EOT and FU visit.
25. Completion of questionnaires should be performed prior to any other assessment scheduled for the in-clinic visit and in the following order: [redacted], 5) PHQ-9, 6) C-SSRS. Refer to Sections 3.3.4.1 and 5.2.5.1 for PHQ-9 and C-SSRS criteria requiring discontinuation and safety measures to be followed.

Proprietary confidential information © 2025 Boehringer Ingelheim International GmbH or one or more of its affiliated companies

26. Each trial participant will receive training in the administration of the prefilled syringes. Site staff will observe the trial participant administering the prefilled syringe in the clinic at Visit 2. The remote visits (if allowed per local regulations) may be performed as video phone calls (refer to Section 6.2). IMP administration may be observed by site staff during Visits 3, 4, 5, 8 and 11. Site staff and the trial participant might plan to schedule Visits 3, 4, 5, 8 and 11 on the day of planned IMP administration to allow site staff to observe the injection at these visits if needed.
27. Trial participants will receive enough IMP to cover the time period until the next scheduled visit. The investigator should instruct the trial participants about the IMP storage temperature requirements. Trial participants should be instructed about the necessity to return all IMP related packaging used and unused. Refer to Section 4.1.4.
28. At the screening visit, no IMP will be dispensed.
29. Refer to Section 4.1.4 for details.
30. The counselling on diet and physical activity should be performed by a dietician or equivalent qualified delegate. It should include the calculation of the individualized energy requirement, total energy expenditure (TEE), and methods to change the dietary composition and amount of physical activity. The counselling on diet and physical activity may be performed on a separate day from the rest of the visit's trial procedures but must occur within the visit window. The study personnel should provide reinforcement and encouragement for lifestyle modifications. The counselling includes the review of diet and exercise goals. Refer to Section 4.2.1.2 for details.
31. Trial participant materials include: 1) Instructions and eDiary to record IMP dosing information, 2) Trial participant ID card, 3) Instructions and trial participant brochure for control of sickness, and 4) Dietary materials and a paper diary to record body weight measurements, physical activity, and dietary compliance. In addition, for trial participants with T2DM or who develop T2DM during the trial, the blood glucose monitoring device and IFU will be provided.
32. At screening, only the paper diary will be given to the trial participants.
33. Participants with T2DM or who develop T2DM must record any hypo- or hyperglycaemic episodes in the eDiary which then must regularly be reviewed. Weekly testing is recommended. Additional measurements should be taken in case of hypo- or hyperglycaemia-related symptoms.

[redacted]

35. IRT call and dispense of IMP only applicable for participants who agreed to enter the optional extended treatment period of 4 weeks.
36. Optional extended treatment period (ETP): After completion of the main treatment period of 48 weeks, participants may agree to enter an optional extended treatment period, which includes an additional 4 weeks at the previously assigned dose. The EOT and W52/Visit 16 should be combined in one visit (no duplicate assessments to be performed).

Proprietary confidential information © 2025 Boehringer Ingelheim International GmbH or one or more of its affiliated companies

TABLE OF CONTENTS

TITLE PAGE 1
CLINICAL TRIAL PROTOCOL SYNOPSIS 2
SCHEDULE OF ASSESSMENTS 8
TABLE OF CONTENTS 15
ABBREVIATIONS AND DEFINITIONS 19
1. INTRODUCTION 22
1.1 MEDICAL BACKGROUND 22
1.2 DRUG PROFILE 23
1.3 RATIONALE FOR PERFORMING THE TRIAL 26
1.4 BENEFIT - RISK ASSESSMENT 27
1.4.1 Benefits 27
1.4.2 Risks 28
1.4.3 Discussion 32
2. TRIAL OBJECTIVES AND ENDPOINTS 33
2.1 MAIN OBJECTIVES, PRIMARY AND SECONDARY ENDPOINTS 33
2.1.1 Main objectives 33
2.1.2 Primary endpoints 33
2.1.3 Secondary endpoints 33
2.2 FURTHER OBJECTIVES AND FURTHER ENDPOINTS 34
2.2.1 Further objectives 34
2.2.2 Further endpoints 34
3. DESCRIPTION OF DESIGN AND TRIAL POPULATION 38
3.1 OVERALL TRIAL DESIGN 38
3.2 DISCUSSION OF TRIAL DESIGN, INCLUDING THE CHOICE OF CONTROL GROUP(S) 39
3.3 SELECTION OF TRIAL POPULATION 39
3.3.1 Main diagnosis for trial entry 39
3.3.2 Inclusion criteria 40
3.3.3 Exclusion criteria 41
3.3.4 Discontinuation of trial participants from treatment or assessments 44
3.3.4.1 Discontinuation of trial treatment 45
3.3.4.2 Withdrawal of consent to trial participation 49
3.3.4.3 Discontinuation of the trial by the sponsor 50
3.3.4.4 Lost to follow-up 50
4. TREATMENTS 52
4.1 INVESTIGATIONAL TREATMENTS 52
4.1.1 Identity of the Investigational Medicinal Products 52
4.1.2 Selection of doses in the trial and dose modifications 52
4.1.3 Method of assigning trial participants to treatment groups 53
4.1.4 Drug assignment and administration of doses for each trial participant 53

Proprietary confidential information © 2025 Boehringer Ingelheim International GmbH or one or more of its affiliated companies

4.1.4.1 Management of gastrointestinal symptoms ... 56
4.1.5 Blinding and procedures for unblinding ... 57
4.1.5.1 Blinding ... 57
4.1.5.2 Emergency unblinding and breaking the code ... 57
4.1.6 Packaging, labelling, and re-supply ... 58
4.1.7 Storage conditions ... 58
4.1.8 Drug accountability ... 58
4.2 OTHER TREATMENTS, EMERGENCY PROCEDURES, RESTRICTIONS ... 59
4.2.1 Other treatments and emergency procedures ... 59
4.2.1.1 Concomitant medications ... 59
4.2.1.1.1 Home glucose monitoring ... 59
4.2.1.1.2 Rescue medication for treatment of hyperglycaemia ... 60
4.2.1.1.3 Hypoglycaemic episodes ... 60
4.2.1.2 Dietary and physical activity counselling ... 61
4.2.1.3 Severe and/or systemic hypersensitivity including injection reaction and anaphylactic reaction ... 62
4.2.1.4 Elective surgery/procedures that require general anaesthesia or deep sedation ... 62
4.2.2 Restrictions ... 63
4.2.2.1 Restrictions regarding concomitant treatment ... 63
4.2.2.2 Restrictions on diet and lifestyle ... 65
4.2.2.3 Contraception requirements ... 66
4.3 TREATMENT COMPLIANCE ... 66

5. ASSESSMENTS ... 67

5.1 ASSESSMENT OF EFFICACY ... 67
5.1.1 MRI ... 67
5.1.2 Fibroscan® ... 67
5.1.3 Body measurements ... 68
5.1.3.1 Weight measurement ... 68
5.1.3.2 Height ... 68
5.1.3.3 Body mass index ... 68
5.1.3.4 Waist and hip circumference measurement ... 68
5.1.4 Blood pressure measurements ... 68
[redacted]
5.1.6 Efficacy laboratory parameters ... 69
5.2 ASSESSMENT OF SAFETY ... 70
5.2.1 Physical examination ... 70
5.2.2 Vital signs ... 70
5.2.3 Safety laboratory parameters ... 70
5.2.4 Electrocardiogram ... 72
5.2.5 Other safety parameters ... 74
5.2.5.1 Mental health questionnaires ... 74
5.2.5.2 Eye examination for participants with T2DM ... 75
5.2.6 Assessment of adverse events ... 75
5.2.6.1 Definitions of AEs ... 75
5.2.6.1.1 Adverse event ... 75
5.2.6.1.2 Serious adverse event ... 76

Proprietary confidential information © 2025 Boehringer Ingelheim International GmbH or one or more of its affiliated companies

5.2.6.1.3 AEs considered "Always Serious" ... 76
5.2.6.1.4 Adverse events of special interest ... 76
5.2.6.1.5 Intensity (severity) of AEs ... 80
5.2.6.1.6 Causal relationship of AEs ... 80
5.2.6.1.7 Adverse events requiring additional data collection ... 81
5.2.6.2 Adverse event collection and reporting ... 84
5.2.6.2.1 AE Collection ... 84
5.2.6.2.2 AE reporting to the sponsor and timelines ... 85
5.2.6.2.3 Pregnancy ... 85
5.2.6.2.4 Other safety topics ... 85
5.3 DRUG CONCENTRATION MEASUREMENTS AND PHARMACOKINETICS ... 86
5.3.1 Assessment of pharmacokinetics ... 86
5.3.2 Methods of sample collection ... 86
[redacted]
5.3.4 Pharmacokinetic - pharmacodynamic relationship ... 86
[redacted]
5.5 BIOBANKING ... 88
5.5.1 Methods and timing of sample collection ... 88
5.6 OTHER ASSESSMENTS ... 88
5.6.1 Immunogenicity ... 88
5.6.1.1 Timing of immunogenicity measures ... 88
5.6.1.2 Methods of sample collection ... 89
5.6.1.3 Immunogenicity analysis ... 89
5.6.2 Clinical staging of obesity ... 89
[redacted]
5.7 APPROPRIATENESS OF MEASUREMENTS ... 90
6. INVESTIGATIONAL PLAN ... 91
6.1 VISIT SCHEDULE ... 91
6.2 DETAILS OF TRIAL PROCEDURES AT SELECTED VISITS ... 91
6.2.1 Screening period ... 92
6.2.2 Treatment period ... 96
6.2.3 Follow-up period and trial completion ... 98
6.2.4 Exceptional conditions ... 99
7. STATISTICAL METHODS AND DETERMINATION OF SAMPLE SIZE ... 100
7.1 NULL AND ALTERNATIVE HYPOTHESES ... 100
7.2 PLANNED ANALYSES ... 100
7.2.1 General considerations ... 100
7.2.2 Handling of Intercurrent Events ... 101
7.2.3 Primary objective analyses ... 104
7.2.3.1 Main analyses ... 104
7.2.3.2 Sensitivity Analyses ... 105
[redacted]

Proprietary confidential information © 2025 Boehringer Ingelheim International GmbH or one or more of its affiliated companies

7.2.3.4 Supplementary Analyses 105
7.2.4 Secondary objective analyses 105
7.2.5 Further objective analyses 106
7.2.6 Safety analyses 106
7.2.7 Other Analyses 106
[redacted]
7.3 HANDLING OF MISSING DATA 107
7.4 RANDOMISATION 107
7.5 DETERMINATION OF SAMPLE SIZE 107
8. INFORMED CONSENT, TRIAL RECORDS, DATA PROTECTION, PUBLICATION POLICY, AND ADMINISTRATIVE STRUCTURE 109
8.1 TRIAL APPROVAL, PATIENT INFORMATION, INFORMED CONSENT 109
8.2 DATA QUALITY ASSURANCE 110
8.3 RECORDS 110
8.3.1 Source documents 110
8.3.2 Direct access to source data and documents 111
8.3.3 Storage period of records 111
8.4 EXPEDITED REPORTING OF ADVERSE EVENTS 112
8.5 STATEMENT OF CONFIDENTIALITY AND TRIAL PARTICIPANT PRIVACY 112
8.5.1 Collection, storage, and future use of biological samples and corresponding data 112
8.6 TRIAL MILESTONES 113
8.7 ADMINISTRATIVE STRUCTURE OF THE TRIAL 113
9. REFERENCES 115
9.1 PUBLISHED REFERENCES 115
[redacted]
10. APPENDICES 120
10.1 TRIAL PARTICIPANT FEEDBACK 120
10.2 MANAGEMENT OF GASTROINTESTINAL SYMPTOMS 121
10.3 CONTRACEPTION METHODS ALLOWED IN THIS TRIAL 124
10.4 TIME SCHEDULE FOR PHARMACOKINETIC (PK) AND ANTI-DRUG ANTIBODY (ADA) / NEUTRALISING ANTIBODY (NAB) BLOOD SAMPLING 125
10.5 CLINICAL STAGING OF OBESITY 126
11. DESCRIPTION OF GLOBAL AMENDMENT(S) 127
11.1 GLOBAL AMENDMENT 1 127
11.2 GLOBAL AMENDMENT 2 130
11.3 GLOBAL AMENDMENT 3 141
11.4 GLOBAL AMENDMENT 4 150

Proprietary confidential information © 2025 Boehringer Ingelheim International GmbH or one or more of its affiliated companies

ABBREVIATIONS AND DEFINITIONS

A1At	Alpha-1 Antitrypsin
ADA	Anti-Drug Antibodies
ADR	Adverse drug reactions
AE	Adverse Event
AESI	Adverse Event of Special Interest
AKI	Acute kidney injury
ALT	Alanine amino transferase
ANCOVA	Analysis of Covariance
ApoB	Apolipoprotein B
[redacted]	[redacted]
AST	Aspartate amino transferase
AUC	Area under the Curve
BMI	Body Mass Index
CAP™	Controlled Attenuation Parameter
CEC	Clinical Event Committee
CI	Confidence interval
CK	Creatine kinase
CKD-EPIcr	Chronic Kidney Disease Epidemiology Collaboration creatinine equation
[redacted]	[redacted]
COA	Clinical outcome assessment
CRF	Case Report Form
CRO	Contract Research Organisation
C-SSRS	Columbia-Suicide Severity Rating Scale
cT1	Iron corrected T1
CTCAE	Common Terminology Criteria for Adverse Events
[redacted]	[redacted]
CV	Cardiovascular
DBP	Diastolic Blood Pressure
DILI	Drug induced liver injury
DMC	Data Monitoring Committee
DPP-4i	Dipeptidyl peptidase 4 inhibitor
ECG	Electrocardiogram
eCOA	Electronic Clinical Outcome Assessment
eCRF	Electronic case report form
EDC	Electronic Data Capture/Collection
eGFR	Estimated glomerular filtration rate
ELF	Enhanced liver fibrosis
EOS	End of Study
EOT	End of Treatment
ETD	Early Treatment Discontinuation
ETP	Extended Treatment Period
FAST	FibroScan-AST
FDA	Food and Drug Administration
FIB-4	Fibrosis-4
[redacted]	[redacted]
FPG	Fasting Plasma Glucose
FPI	Fasting Plasma Insulin
FU	Follow-up
GCGR	Glucagon receptor
GCP	Good Clinical Practice
GGT	Gamma glutamyltransferase
GI	Gastrointestinal
GIP	Glucose-dependent insulinotropic polypeptide

Proprietary confidential information © 2025 Boehringer Ingelheim International GmbH or one or more of its affiliated companies

GLP-1R	Glucagon-like-peptide (GLP) 1 receptor
[redacted]	[redacted]
HbA_{1c}	Glycosylated haemoglobin A1c
hCG	Human chorionic gonadotropin
HCP	Health care professional
HCV	Hepatitis C virus
HDL	High-density lipoprotein
HF	Heart Failure
HIV	Human Immunodeficiency Virus
HOMA-IR	Homeostasis Model Assessment -Insulin Resistance
HOMA-ß	Homeostasis Model Assessment of Beta-cell function
HRQoL	Health-related quality of life
hsCRP	High sensitivity C-reactive protein
[redacted]	[redacted]
ICE	Intercurrent Event
ICH	International Conference of Harmonisation
IEC	Independent Ethics Committee
IFU	Instructions for use
IMP	Investigational Medicinal Product
INR	International Normalized Ratio
IRB	Institutional Review Board
ISF	Investigator Site File
[redacted]	[redacted]
KDIGO	Kidney Disease Improving Global Outcomes
LDL	Low-density lipoprotein
LDL-C	Low-density lipoprotein C
MASH	Metabolic dysfunction-associated steatohepatitis
MASLD	Metabolic dysfunction-associated steatotic liver disease
MAST	MRI-AST
MedDRA	Medical Dictionary for Regulatory Activities
MEN-2	Multiple endocrine neoplasia syndrome type 2
MMRM	Mixed Model Repeated Measures
MAR	Missing at random
MRE	Magnetic Resonance Elastography
MRI	Magnetic Resonance Imaging
MRI-PDFF	MRI proton density fat fraction
MTC	Medullary thyroid carcinoma
NAb	Neutralising Antibody
NAFL	Non-alcoholic fatty liver
NAFLD	Non-alcoholic fatty liver disease
NAS	NAFLD (Non-Alcoholic Fatty Liver Disease) Activity Score
NASH	Non-Alcoholic SteatoHepatitis
NYHA	New York Heart Association
PHQ-9	Participant Health Questionnaire 9
PK/PD	Pharmacokinetics/Pharmacodynamics
PRO	Participant Reported Outcome
[redacted]	
QTcF	QT interval corrected for heart rate according to Fridericia's formula
s.c.	Subcutaneous
SAE	Serious adverse event
SBP	Systolic Blood Pressure
SD	Standard deviation
[redacted]	[redacted]
SGLT2i	Sodium-glucose co-transporter-2 (SGLT2) inhibitors
SMBG	Self-monitoring blood glucose

Proprietary confidential information © 2025 Boehringer Ingelheim International GmbH or one or more of its affiliated companies

SoA	Schedule of Assessments
T2DM	Type 2 diabetes mellitus
TSAP	Trial Statistical Analysis Plan
[redacted]	[redacted]
ULN	Upper Limit of Normal
VCTE™	Vibration-Controlled Transient Elastography
VLDL	Very-low-density lipoprotein
WOCBP	Women of childbearing potential

Proprietary confidential information © 2025 Boehringer Ingelheim International GmbH or one or more of its affiliated companies

1. INTRODUCTION

1.1 MEDICAL BACKGROUND

NAFLD (also known as metabolic dysfunction-associated steatotic liver disease, MASLD) is a metabolic dysfunction that represents a range of liver damage conditions from steatosis alone (non-alcoholic fatty liver, NAFL, also known as metabolic dysfunction associated steatosis, MAS) to the inflammatory phenotype, NASH (also known as metabolic dysfunction-associated steatohepatitis, MASH) [R23-2769][1]. The latter involves persistent liver inflammation and may result in progressive fibrosis and loss of liver function [R23-1199].

The global prevalence of NAFLD is increasing and closely related to the worldwide growth of obesity and type 2 diabetes mellitus (T2DM), which are major drivers of NAFLD severity and progression [R23-1196]. It affects approximately 30% of the general population and is the most common liver disease in Western countries [R22-2292]. In a recent meta-analysis of 151 studies (N=101,028) [R23-1416], the prevalence of NAFLD in the overweight and obese population is 69.9% (95% confidence interval [CI] 65.4, 74.2) and 75.3% (95% CI 70.9, 79.2), respectively. Significant fibrosis (stages F2-4) presents in 20.3% (95% CI 11.3, 33.6) of overweight individuals with NAFLD and in 21.6% (95% CI 11.5, 36.9) of obese individuals with NAFLD. Advanced fibrosis (stages F3-F4) presents in 6.7% (95% CI 4.4, 10.0) of overweight individuals with NAFLD and 6.9% (95% CI 3.9, 11.9) of obese individuals with NAFLD. The meta-analysis included participants with obesity who were at risk of procedures (e.g. bariatric surgery); thus the actual prevalence of NAFLD in the overall population with obesity might be lower.

A recent pooled analysis of biopsy studies suggests that the transition from NAFLD to NASH is dynamic (Figure 1) [R22-2295]. It is estimated that 20% of individuals with NAFLD have NASH, and 20% of those may progress to cirrhosis over 3-4 decades. A limitation of this study was the lack of estimates for regression rates. Progression and regression rates were reported by a meta-analysis including both observational studies and randomised controlled trials (54 studies involving 26,738 participants using liver biopsy or imaging to diagnose NAFLD during a follow-up period of ≥48 weeks), which confirmed that the transition/progression from NAFL to NASH or more advanced stages of the disease is dynamic. Among NAFL participants, 31% developed NASH after 4.7 years (incidence rate 7.4/100 person-years). In 29% of participants with NASH, resolution occurred after a median of 3.5 years (incidence rate 5.1/100 person-years). Time to progress by one fibrosis stage for F0, F1, F2, and F3 was 9.9, 10.3, 13.3, and 22.2 years, respectively [R23-2419]. In another study in 733 participants from United States, United Kingdom, and Australia with clinical diagnosis and liver biopsy-confirmed NAFLD, participants with overweight or obesity had 2.35 and 5.70 greater odds of advanced fibrosis (stages F3-F4), respectively, versus those with normal weight [R17-3207].

[1]Within the context of trial 1404-0056 the terms NAFLD, NAFL and NASH are to be considered interchangeable to MASLD, MASL and MASH, respectively.

Proprietary confidential information © 2025 Boehringer Ingelheim International GmbH or one or more of its affiliated companies

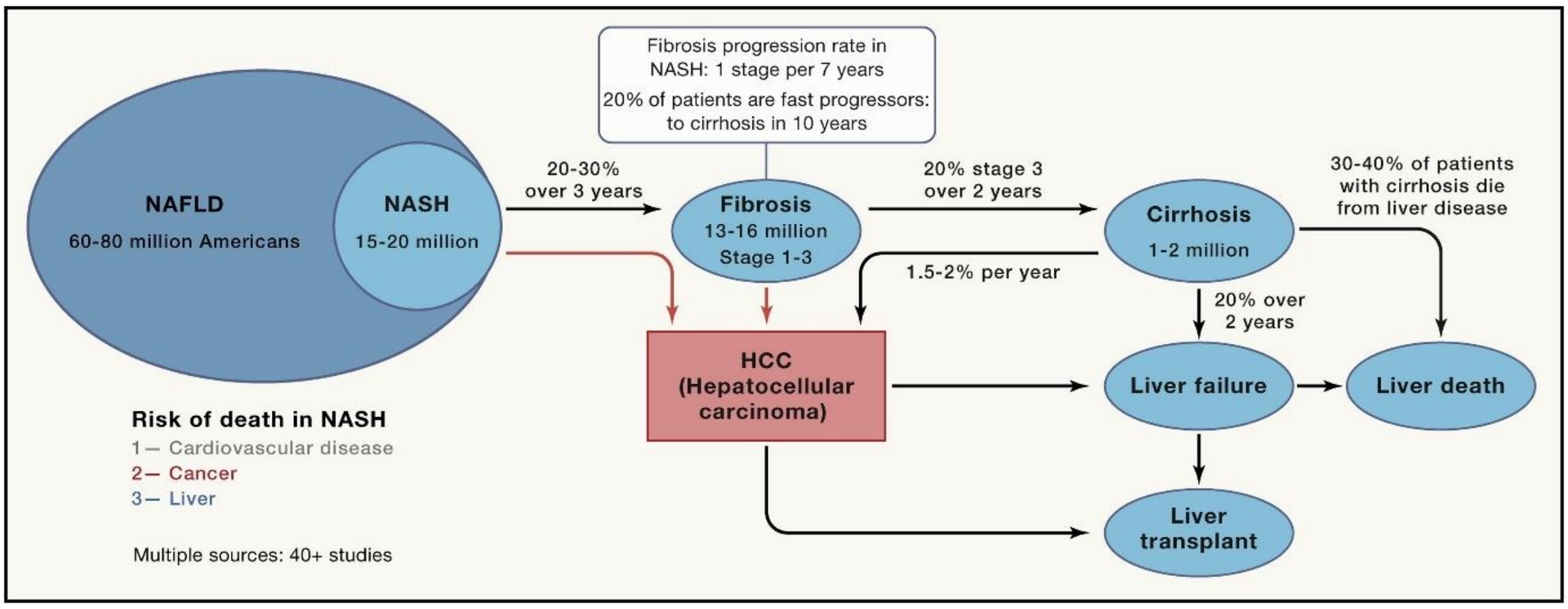

Figure 1 NAFLD natural history in the United States

Source data: [R22-2295]

NAFLD is associated with obesity, dyslipidemia, hypertension, insulin resistance, diabetes mellitus, and chronic kidney disease [R23-1199]. It is also associated with an increased risk of all-cause, cardiovascular, and cancer-related mortality [R23-1197]. Patients with NASH and fibrosis stages ≥F2 are at higher risk for liver-related events and mortality [R23-0537]. NASH also adversely impacts patients' health-related quality of life (HRQoL) [R23-1198].

The identification and stratification of patients with at-risk NASH or advanced fibrosis can be performed using non-invasive tests such as FIB-4, ELF score, MRI-PDFF, cT1, MRI, FibroScan® VCTE, MRE and their derived scores (FAST and MAST score) [R23-0537, R23-1377]. It is thought that non-invasive imaging modalities are needed to replace liver biopsy for the diagnosis and monitoring of NASH in clinical trials [R23-1383]. For example, MRI-PDFF is a reliable, highly reproducible, and accurate non-invasive biomarker for histological improvement in disease activity [R23-1379]. A meta-analysis demonstrated that a ≥30% relative decline in MRI-PDFF is associated with higher odds of histologic response (2-point improvement in NAFLD Activity Score (NAS) with at least 1-point improvement in lobular inflammation or ballooning) and NASH resolution [R23-1379]. In addition, MRI-PDFF response (≥30% relative decline in MRI-PDFF) was an independent predictor of fibrosis regression with an adjusted odds ratio of 6.46 in a recent US study [R23-1380]. Importantly, the 2023 AASLD Guidance on NAFLD stated that improvement in alanine aminotransferase (ALT) and reduction in liver fat content by imaging in response to an intervention could be used as a surrogate for histological improvement in disease activity [R23-0537].

1.2 DRUG PROFILE

Mode of action

Survodutide is a dual agonist of glucagon-like-peptide (GLP) 1 receptor (GLP1R) and glucagon receptor (GCGR). Targeting both GLP1 and glucagon signalling pathways is expected to provide synergistic clinical benefit for chronic weight management and obesity.

Simultaneous dual activation of GLP1R and GCGR by survodutide is anticipated to achieve improved glycaemic control, reduction in body weight, and improvement in NASH.

Proprietary confidential information © 2025 Boehringer Ingelheim International GmbH or one or more of its affiliated companies

Survodutide leads to full activation of the GLP1R at the predicted therapeutic exposure, but only to a partial activation of the GCGR, which seems sufficient to achieve greater weight loss than GLP1R activation alone, without activation of hyperglycaemic pathways.

Key PK characteristics

PK of survodutide was investigated after single s.c. doses of 0.3, 0.5, and 1.2 mg in trial 1404-0001 [c22991258]. C_{max} was 7, 13, and 34 nM, respectively. t_{max} was reached between 28 and 34 h. $t_{1/2}$ ranged from 109 to 115 h. Dose proportionality was demonstrated for C_{max}, $AUC_{0\text{-}168\,h}$, and $AUC_{0\text{-}\infty}$.

In trial 1404-0021 [c33609485], a slightly higher average exposure was observed in Japanese compared with Caucasians. In a population analysis including data from trials 1404-0001, 1404-0003, and 1404-0021 [c22991258, c26759941, c33609485], survodutide PK was not different in Japanese and non-Japanese participants after accounting for body weight effects [c37765403].

Further details, also on multiple dosing, can be found in the investigator's brochure [c14085752].

Drug interactions

GLP-1R agonism reduces gastric emptying. This was assessed by measuring the effect of survodutide on the absorption of paracetamol in trial 1404-0001 [c22991258]. Paracetamol C_{max} and $AUC_{0\text{-}10}$ were slightly lower after a single dose of 0.3 mg survodutide, and strongly decreased after a single dose of 1.2 mg survodutide compared with administration of paracetamol alone. The effect of survodutide on gastric emptying decreases with multiple dosing in trial 1404-0003 [c26759941].

A population PK/PD analysis based on data from Japanese and non-Japanese subjects did not indicate meaningful ethnic differences in the survodutide effect on gastric emptying, as measured with paracetamol absorption test [c37765403].

Residual effect period

The residual effect period of survodutide is 28 days (half-life 112 h). This is the period after the last dose with measurable drug levels and/or PD effects still likely to be present.

Data from non-clinical studies

In a 4-week study in mice with diet-induced obesity, survodutide induced a body weight loss greater than the pure GLP-1R agonist liraglutide. Survodutide dose-dependently increased energy expenditure while liraglutide had no effect even at supra-therapeutic exposure. The increased energy expenditure contributes to a longer lasting negative energy balance than GLP-1R agonism alone, while achieving a favourable benefit/risk profile [c14085752].

In toxicity studies of up to 26 weeks in mice and up to 39 weeks in Cynomolgus monkeys, survodutide induced reduced food consumption and body weight loss or reduced body weight gain. These intended pharmacological effects limited the highest dose assessed in the toxicity studies performed in healthy (non-obese) animals and, therefore, only low exposure multiples to clinical use could be tested. All findings seen in the toxicity studies have been recorded in studies where food has been intentionally restricted or with treatments inducing unspecific

Proprietary confidential information © 2025 Boehringer Ingelheim International GmbH or one or more of its affiliated companies

stress, and therefore were interpreted as secondary effects. These observations included signs of metabolic stress for the liver, as indicated by hepatocellular vacuolation, slight increase of plasma bilirubin or changes in hepatocellular glycogen content, or changes of haematopoiesis in bone marrow, spleen, and thymus, as well as atrophy of the ovary. Cynomolgus monkeys, especially female monkeys, are considered oversensitive with regard to limiting body weight effects due to their low total body fat content (5.6% ± 3.81%), compared with humans and even more to patients with obesity.

In the non-clinical studies on embryofoetal development, there were no adverse effects on maternal toxicity and embryofoetal development up to and including the high dosage levels of 1498 µg/kg/week in mice and 252 µg/kg/week in rabbits, indicating that the compound is not teratogenic.

Data from clinical studies

Survodutide was studied in healthy volunteers in the single rising dose trial 1404-0001 [c22991258]. Similar to other GLP-R1 agonists, nausea and vomiting were seen with increasing doses up to 1.2 mg, and planned dose escalation to further levels was stopped due to severe nausea and vomiting in a number of healthy volunteers.

Survodutide has also been studied in otherwise healthy volunteers with obesity or overweight in the multiple rising dose trial 1404-0003 [c26759941] with dose escalation to investigate the safety and tolerability of different dose escalation schemes to determine a dose escalation scheme that minimise GI AEs. In Part A, survodutide was administered for 6 weeks in 4 different titration schemes (1 once-daily dose group and 3 once-weekly dose groups with weekly dose escalation). The most frequent AEs were GI disorders, such as nausea, dyspepsia, abdominal distention, and vomiting. Cardiac disorders were reported in the survodutide treated group with 9% AV block first-degree, 9% second-degree, 8% sinus tachycardia, and 6% ventricular extrasystoles. All of the cardiac AEs were considered mild to moderate in intensity, none was reported as severe. An independent review of 24 h Holter recordings of trial participants in Part A of the trial with ECG findings noted at the site using a 12-lead telemetry system was conducted by an independent cardiologist at a central ECG lab. It was concluded that the IMP given at 4 different doses did not show a notable increase in the findings reported by the cardiologist. The incidences in the categories were similar to those occurring at baseline and also not significantly different from those seen in the placebo-treated participants. In Part B of this trial, survodutide was administered for 16 weeks in 3 different titration schemes. As in Part A, the most frequent AEs were GI disorders, in this case nausea, vomiting, dyspepsia, diarrhoea, and eructation. No deaths, or protocol specified AESIs were reported in this trial.

In trial 1404-0021 in healthy Japanese volunteers [c33609485], GI disorders (decreased appetite, dyspepsia, diarrhoea, and nausea) were the most commonly reported type of AEs. There were no clinically relevant cardiac disorders reported. No unexpected safety or tolerability concerns were noted. The most frequently reported AEs were GI disorders.

In the Phase II trial 1404-0002 in 411 participants with T2DM and obesity/overweight [c36750061], survodutide showed an overall good safety profile during the trial with GI events (nausea, vomiting, and diarrhoea) being the most common AEs. Dose-dependent HbA_{1c} reductions were observed with survodutide treatment in participants with T2DM and overweight/obesity. Weekly injections with survodutide effectively lowered absolute HbA_{1c} by up to –1.88% (Week 16) compared with the reduction of 1.47% with open-label

Proprietary confidential information © 2025 Boehringer Ingelheim International GmbH or one or more of its affiliated companies

semaglutide [P22-07379]. Treatment with survodutide induced greater body weight loss than open-label semaglutide after 16 weeks of treatment: −8.95% with the highest 3.6 mg dose vs. −5.40% with semaglutide 1.0 mg [P22-08755]. Severe AEs and SAEs were reported at low frequencies, 5.3% and 3.6% in all participants treated with survodutide, respectively, with similar frequencies reported in the placebo group. There were no cases of pancreatitis. There were no serious or severe cardiac AEs. Of note, 11.9% of participants treated with survodutide discontinued treatment due to GI AEs.

[redacted]

For a more detailed description of the survodutide profile, please refer to the current investigator's brochure [c14085752].

1.3 RATIONALE FOR PERFORMING THE TRIAL

Treatment for NASH represents a large unmet need. Currently there is a very limited number of available pharmacotherapies; their long-term clinical benefits for NASH are still to be proven.

Non-liver-specific management of NASH includes weight loss, lifestyle changes and treatment of associated metabolic syndromes [P23-02853, R23-1381]. Weight loss is one of the most critical interventions for obesity and NAFLD/NASH. The hepatic histological beneficial effect of weight loss was demonstrated for both surgical interventions (bariatric surgery) and lifestyle modifications [R23-1378, R23-1382]. In addition, there is evidence of a dose-response relationship between the magnitude of weight loss and improvement of liver inflammation, ballooning, and resolution of NAFLD or NASH [R23-1384]. A paired liver biopsy study showed that 25% of the subjects achieved resolution of steatohepatitis after lifestyle modifications for 52 weeks. A higher proportion of subjects with ≥5% weight loss had NASH resolution than those with <5% weight loss. Among participants who lost ≥10% of their weight, 90% had resolution of NASH, and 45% had regression of liver fibrosis [R23-1382].

The Phase II trial 1404-0036 in participants with obesity [c31754142] demonstrated that s.c. survodutide as an adjunct to a reduced-calorie diet and increased physical activity was effective for weight loss, while displaying a satisfactory tolerability profile. The weight loss was accompanied by a consistent improvement in weight-related complications, indicated by CV risk factors, lipid profile, and glycaemic factors as well as improvements in clinical outcome assessments.

Proprietary confidential information © 2025 Boehringer Ingelheim International GmbH or one or more of its affiliated companies

This Phase III trial investigates the effect of survodutide once weekly versus placebo on liver fat and body weight as an adjunct to a reduced-calorie diet and increased physical activity in participants with presumed or confirmed NASH and obesity (BMI ≥30 kg/m^2) or overweight (BMI ≥27 kg/m^2) with one or more weight-related co-morbidities.

The study of exploratory and probable valid biomarkers will mostly be hypothesis-generating or supportive to endpoints. These findings will be used to expand the understanding of NAFLD/NASH and survodutide, e.g. by learning more about the mechanism of action of the IMP or the biology of the disease, thereby allowing correlation of patient subgroups with differential responses to treatment and/or prognosis and supporting subsequent trial designs regarding dose and schedule selection.

[redacted]

To be able to address future scientific questions, trial participants will be asked to voluntarily donate biospecimens for banking (see Section 5.5). If the trial participant agrees, banked samples may be used for future biomarker research and drug development projects, e.g. to identify patients that are more likely to benefit from a treatment or experience an AE, or to gain a mechanistic or genetic understanding of the product's effects and thereby better match patients with therapies.

1.4 BENEFIT - RISK ASSESSMENT

The overall benefits and safety profile of survodutide are outlined in previous sections. More information on the benefits, risks, and known AEs is presented in the latest version of the investigator's brochure [c14085752].

1.4.1 Benefits

Trial participants treated with survodutide, together with the close contact with the trial site and counselling by a dietician or a similar qualified healthcare professional, are expected to achieve significant reduction in liver fat content and body weight, as well as improvement of the weight-related complications [redacted]

Proprietary confidential information © 2025 Boehringer Ingelheim International GmbH or one or more of its affiliated companies

1.4.2 Risks

There are no important identified risks specific for survodutide, based on the toxicology programme or any clinical trials conducted for this product to date.

There are 4 important potential risks based on other GLP1R agonists currently approved (class effect), and data from the completed Phase II studies of survodutide. The 4 important potential risks include MTC (C-cell carcinogenicity), acute pancreatitis, pancreatic cancer, and pre-renal acute kidney injury due to dehydration.

The risks for participants caused by participation in the trial, including the trial procedures and exposure to the trial drug, are reasonably low and do not outweigh the potential benefits. The expected side effects are known to be temporary, dose dependent, easy to monitor, and manageable in clinical trials. An overview of trial related risks is presented in Table 1.

Proprietary confidential information © 2025 Boehringer Ingelheim International GmbH or one or more of its affiliated companies

Table 1 Overview of trial related risks

**Possible or known risks of clinical relevance for this trial**	**Summary of data, rationale for the risk**	**Mitigation strategy**
**Survodutide**		
GI disorders (e.g. nausea, vomiting, diarrhoea)	Well-known side effects for GLP-1R agonist-based therapies. In completed survodutide trials, GI AEs occurred mostly during dose escalation phase, and were mild to moderate in intensity and reversible.	Slow and careful dose escalation. Instructions to be given on management of GI symptoms (Section 4.1.4.1)
Acute pancreatitis and pancreatic cancer	Acute pancreatitis is a known side effect for GLP-1R agonist-based therapies.<br>Pancreatic cancer is not a recognised risk in humans.	Participants with a history of either chronic or acute pancreatitis or elevation of serum lipase or serum pancreatic amylase >2x ULN as measured by the central laboratory at screening are excluded from the trial.<br>An extensive safety laboratory will be performed.<br>Timely detection, evaluation, and follow-up of laboratory alterations in selected laboratory parameters to ensure trial participants' safety.
MTC	To date there is no evidence that survodutide causes such AE.<br>MTC is a known side effect for GLP-1R agonist-based therapies in rodents.<br>GLP-1R agonist-based therapies are contraindicated in participants with a personal or family history of MTC.	Participants with personal or family history of medullary thyroid carcinoma or MEN 2 are excluded from the trial.<br>An extensive safety laboratory will be performed.<br>Timely detection, evaluation, and follow-up of laboratory alterations in selected laboratory parameters to ensure trial participants' safety.
Acute kidney injury (pre-renal) due to dehydration	Related to severe GI AEs of GLP-1R agonist class (e.g. severe vomiting or diarrhoea may cause dehydration)	In case of severe GI AEs, such as vomiting, nausea, and diarrhoea, adequate fluid substitution (oral or intravenous) will be initiated. Modification in dose of concomitant medications, in particular diuretics, should be initiated to prevent dehydration or exacerbation of dehydration.<br>Renal function will be routinely monitored. See Section 5.2.6.1.7 for further details.

Proprietary confidential information © 2025 Boehringer Ingelheim International GmbH or one or more of its affiliated companies

Table 1 (cont'd) Overview of trial related risks

**Possible or known risks of clinical relevance for this trial**	**Summary of data, rationale for the risk**	**Mitigation strategy**
Cardiac disorders (increased heart rate and conduction disorders)	Increased heart rate is a known side effect for GLP-1R agonist-based therapies including survodutide.<br>Cardiac conduction disorders were described for GLP-1R agonists in clinical trials and in the prescribing information for liraglutide (Saxenda) [R22-0131], reported as first degree atrioventricular block, right bundle branch block, or left bundle branch block.<br>Survodutide has a low pro-arrhythmic potential based on animal and clinical studies.	Centralised ECG monitoring will be performed at screening, randomisation and at all in-clinic visits during the dose escalation and maintenance periods, and criteria for QT prolongation are defined.
Acute gallbladder disease	Well-known side effect for GLP-1R agonist-based therapies, and well-known AE of rapid significant weight loss with any modalities	An extensive safety laboratory will be performed.<br>Timely detection, evaluation, and follow-up of laboratory alterations in selected laboratory parameters to ensure trial participants' safety.
Systemic hypersensitivity reaction	After administration of any biologic agent or protein, there is a possibility of occurrence of adverse immune reactions which can be local (e.g. redness, pruritus, and or swelling at the injection site) or systemic (e.g. anaphylactic reactions).	Trial participants with a history of allergy/hypersensitivity to the systemically administered trial medication agent or its excipients are excluded from the trial.<br>In case of systemic hypersensitivity reactions including anaphylactic reaction emerging during or after administration of trial medication, the investigator should consider in accordance with severity of the reaction and local standard of care to interrupt and treat the condition.
**Trial procedures**		
Insufficient weight loss	Trial participants who will receive placebo may not achieve sufficient weight loss during the trial, but there may also be non-responders to survodutide who may not lose weight.	Trial participants in the placebo group will remain on standard of care that includes diet and exercise counselling.
Injection site reactions	Well-known side effects for injectables	Instructions will be given.<br>Injection site will be monitored during every in-clinic visit.

Proprietary confidential information © 2025 Boehringer Ingelheim International GmbH or one or more of its affiliated companies

Table 1 (cont'd) Overview of trial related risks

**Possible or known risks of clinical relevance for this trial**	**Summary of data, rationale for the risk**	**Mitigation strategy**
**Other risks**		
Drug-induced liver injury	Rare but severe event, thus under constant surveillance by sponsors and regulators	Timely detection, evaluation, and follow-up of laboratory alterations in selected liver laboratory parameters to ensure trial participants' safety.<br>Section 5.2.6.1.4 provides guidance for the investigators on how to evaluate and treat trial participants with suspected DILI.
Suicide risk, depression, and mood disorder	Survodutide could act on central GLP1R (limited data on survodutide).	C-SSRS will be applied at every visit as required by regulatory agencies.

In this trial, the following safety measures will be applied to minimise the risk for trial participants:

- Trial participants in all groups, including placebo group will receive standard of care that includes diet and physical activity counselling.
- There are no risks expected from stopping the IMP during the trial.
- Centralised ECG monitoring will be performed at screening, randomisation and at in-clinic visits during the dose escalation and maintenance periods, and criteria for QT prolongation are defined.
- Instructions will be given to the trial participants to help improve the tolerability especially during the escalation phase. For trial participants who do not tolerate the assigned escalation schedule, the dose escalation can be modified according to the predefined process. Details are described in Section 4.1.4.1 and Section 10.2.
- During the trial, participants will be under medical observation and thoroughly monitored for both expected and unexpected AEs including administration and evaluation of the C-SSRS, at every visit as required by regulatory agencies for compounds acting on CNS located receptors.
- Paediatric participants (less than 18 years old) are excluded from the trial.
- Participants who develop T2DM will be educated about signs and symptoms of hypoglycaemia, how to treat hypoglycaemia, and how to collect information for each episode of hypoglycaemia. Hypoglycaemic events should be treated, and additional glucose monitoring should be implemented per investigator discretion and medical judgement. In cases of hyperglycaemia, glycaemic rescue treatment should be initiated at the discretion of the investigator.
- WOCBP who are pregnant, nursing or intend to become pregnant or are not using an adequate contraceptive method (Section 4.2.2.3) are excluded from the trial.

To continue the assessment of the safety of survodutide, adjudication of some events (see Section 5.2.6.1.7) will be performed in this trial by a Clinical Event Committee (CEC). The progress of the trial will also be assessed at regular intervals by an independent Data

Proprietary confidential information © 2025 Boehringer Ingelheim International GmbH or one or more of its affiliated companies

Monitoring Committee (DMC). Further details are described in Section 8.7, the CEC Charter, and the DMC Charter.

1.4.3 Discussion

Based on the currently available data, in trial participants with a BMI ≥30 kg/m² or a BMI ≥27 kg/m² with at least one weight-related complication, dual agonism at the GLP1R and GCGR is expected to result in superior body weight loss and associated reduction in liver fat content. Known ADRs (adverse drug reactions) of survodutide include, but not limited to, nausea, vomiting, diarrhoea, constipation, abdominal pain, dizziness, and dyspepsia. Further ADRs are included in the current version of the IB and will be updated on an ongoing basis.

There are no important identified risks defined for survodutide. Important potential risks for survodutide are MTC (C-cell carcinogenicity), pancreatic cancer, acute pancreatitis, and pre-renal acute kidney injury due to dehydration.

In the context of the anticipated benefit of survodutide, the benefit risk evaluation of the compound based on the available preclinical and clinical data, is favourable.

Overall, the potential benefits, coupled with an acceptable safety profile and a well-controlled clinical trial environment support the initiation of the trial.

Proprietary confidential information © 2025 Boehringer Ingelheim International GmbH or one or more of its affiliated companies

2. TRIAL OBJECTIVES AND ENDPOINTS

2.1 MAIN OBJECTIVES, PRIMARY AND SECONDARY ENDPOINTS

2.1.1 Main objectives

This trial aims to provide evidence of the efficacy, safety, and tolerability of survodutide administered once weekly as an adjunct to a reduced-calorie diet and increased physical activity, in comparison with placebo, in participants with presumed or confirmed NASH and obesity (BMI ≥30 kg/m^2) or overweight (BMI ≥27 kg/m^2) with at least one of the following weight-related complications (treated or untreated): hypertension, dyslipidaemia, obstructive sleep apnoea, cardiovascular disease or T2DM.

The primary objective is to demonstrate superiority of survodutide in:

- Reduction in liver fat content assessed by MRI-PDFF, defined as ≥30% from baseline at Week 48 (odds ratio, survodutide vs. placebo), AND
- The difference in adjusted means of relative change (%) in body weight from baseline to Week 48 (survodutide vs. placebo)

The secondary (exploratory) objectives are to demonstrate the superiority of survodutide vs. placebo in terms of the difference in adjusted means of changes from baseline to Week 48 in liver fat content assessed by MRI-PDFF, cT1, ALT levels, waist circumference, HOMA-IR, liver stiffness assessed by MRE, and liver volume assessed by MRI.

All treatment comparisons will be as randomised, including the effects of any early treatment discontinuation or any prohibited (anti-obesity) treatment including the effects of anti-obesity treatments, including GLP1R agonists. In addition, treatments will be compared as randomised, but excluding effects of any early treatment discontinuation and any prohibited (anti-obesity) treatments.

2.1.2 Primary endpoints

The co-primary endpoints are:

- Relative reduction in liver fat content of at least 30% from baseline to Week 48 (yes/no) assessed by MRI-PDFF [%], AND
- Relative change (%) in body weight [kg] from baseline to Week 48

2.1.3 Secondary endpoints

The secondary endpoints are:

- Absolute change from baseline to Week 48 in liver fat content assessed by MRI-PDFF [%]
- Relative change (%) from baseline to Week 48 in liver fat content assessed by MRI-PDFF [%]
- Reduction from baseline to Week 48 in cT1 [ms] levels of ≥80 ms (yes/no)
- Absolute and relative change from baseline to Week 48 in ALT [U/L] levels
- Absolute and relative change from baseline to Week 48 in waist circumference [cm]

Proprietary confidential information © 2025 Boehringer Ingelheim International GmbH or one or more of its affiliated companies

- Absolute and relative change from baseline to Week 48 in HOMA-IR (FPI [mlU/L]·FPG [mmol/L]/22.5)
- Absolute and relative change from baseline to Week 48 in liver stiffness [kPa] assessed by MRE
- Absolute and relative change in liver volume [mL] from baseline to Week 48 measured using MRI

2.2 FURTHER OBJECTIVES AND FURTHER ENDPOINTS

2.2.1 Further objectives

Further objectives may include but are not limited to evaluation of safety and tolerability, efficacy, population PK of survodutide, biochemical/[redacted] biomarkers related to obesity and NAFLD activity.

2.2.2 Further endpoints

[redacted]

Absolute and/or relative change from baseline to Week 48 in:

[redacted]

- Body weight [kg]
- Systolic blood pressure [mmHg]
- Diastolic blood pressure [mmHg]
- Total cholesterol [mg/dL]
- HDL cholesterol [mg/dL]
- LDL cholesterol [mg/dL]
- Triglycerides [mg/dL]
- VLDL cholesterol [mg/dL]

Proprietary confidential information © 2025 Boehringer Ingelheim International GmbH or one or more of its affiliated companies

[redacted]

- HbA_{1c} [%, mmol/mol], overall and per diabetes status
- Fasting plasma glucose (FPG) [mmol/L], overall and per diabetes status
- Fasting plasma insulin (FPI) [mIU/L], overall and per diabetes status

[redacted]

  - ELF score [units on a scale], [redacted]

[redacted]

Proprietary confidential information © 2025 Boehringer Ingelheim International GmbH or one or more of its affiliated companies

[redacted]

- Liver stiffness [kPa] assessed by FibroScan®
- cT1 values [ms]
- Aspartate aminotransferase (AST) levels [U/L]

[redacted]

- Uric acid [µmol/L]

[redacted]

Categorical based on relative reduction from baseline to Week 48 in:

- Liver fat content [%] of at least 50% (yes/no) assessed by MRI-PDFF
- Liver fat content [%] of at least 70% (yes/no) assessed by MRI-PDFF
- Body weight [kg] of at least 5% (yes/no)

Proprietary confidential information © 2025 Boehringer Ingelheim International GmbH or one or more of its affiliated companies

- Body weight [kg] of at least 10% (yes/no)
- Body weight [kg] of at least 15% (yes/no)
- Body weight [kg] of at least 20% (yes/no)

[redacted]

Proprietary confidential information © 2025 Boehringer Ingelheim International GmbH or one or more of its affiliated companies

3. DESCRIPTION OF DESIGN AND TRIAL POPULATION

3.1 OVERALL TRIAL DESIGN

This is a randomised, double-blind, placebo-controlled Phase III clinical trial comparing survodutide 6.0 mg once weekly to placebo once weekly, as an adjunct to a reduced-calorie diet and increased physical activity, in participants with presumed or confirmed NASH and obesity (BMI ≥30 kg/m^2) or overweight (BMI ≥27 kg/m^2) and at least one weight-related comorbidity. The overall trial design and dose escalation schedule are depicted in Figure 2.

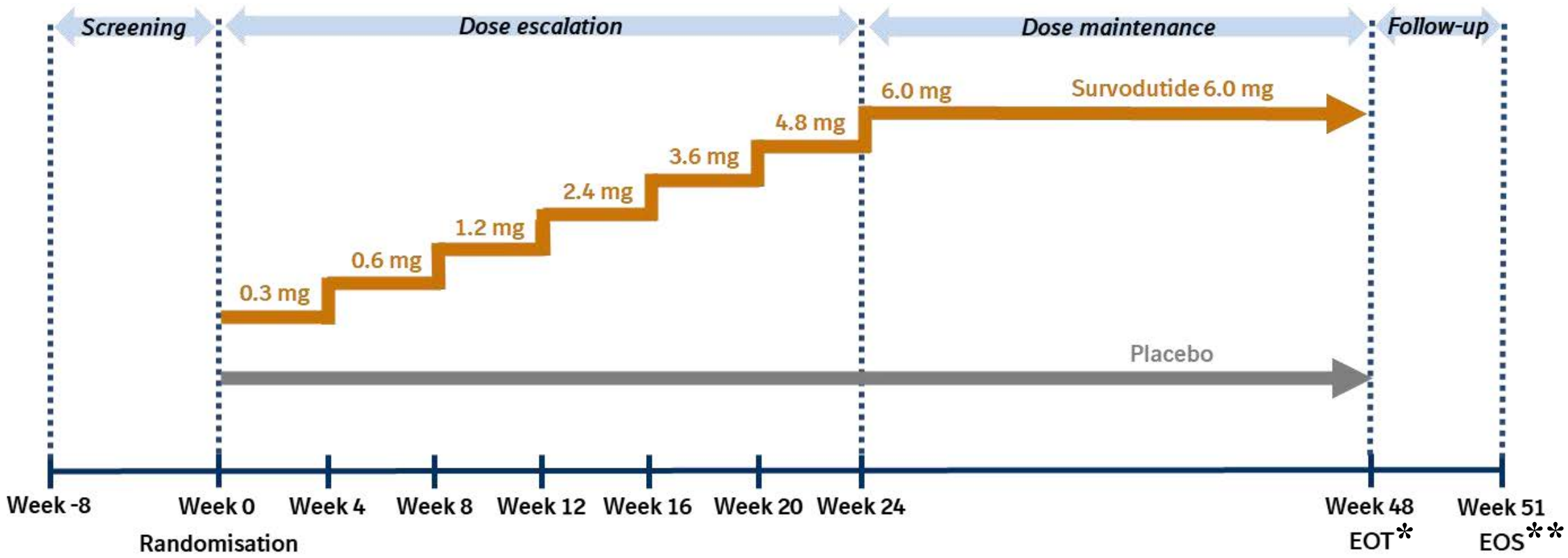

* EOT will take place at Week 52 for participants with ETP
** EOS will take place at Week 55 for participants with ETP

Figure 2 Trial design

The trial has a screening period of up to 8 weeks to assess participant eligibility criteria. Re-screening of previously screen-failed participants will be permitted providing the reasons for screen failure were reversible and have been resolved, based on the investigator's judgement. Re-screening is only allowed once.

Approximately 160 eligible participants will be randomised to survodutide or matching placebo in a 2:1 ratio (survodutide: at least 104 participants, placebo: at least 52 participants) as an adjunct to a reduced-calorie diet and increased physical activity. Randomisation will be stratified by the presence of T2DM.

During the course of the trial, the number of participants increased to approx. 220 due to an unexpected sharp drop in the screening failure percentage following recruitment closure.

The main treatment duration is 48 weeks in total, including a dose escalation period of up to 24 weeks (approximately 4 weeks between each dose escalation; up to a maximum of 32 weeks if 1 or 2 re-escalation attempts are needed due to GI effects) and a maintenance [redacted]. After completion of the main treatment period of 48 weeks, participants may agree to enter an optional extended treatment period, which includes an additional 4 weeks at the previously assigned dose. [redacted] The last administration of IMP is at Week 47 or at Week 51 for participants, who agreed to the

Proprietary confidential information © 2025 Boehringer Ingelheim International GmbH or one or more of its affiliated companies

optional extended treatment period. A Follow-up/End-of-Study (FU/EOS) Visit for safety assessments is scheduled at Week 51 (i.e. 28 days after the last IMP administration or 21 days after Week 48/End of Treatment [EOT] Visit). For participants, who agreed to the optional extended treatment, a Follow-up/End-of-Study (FU/EOS) Visit for safety assessments is scheduled at Week 55 (i.e. 28 days after the last IMP administration or 21 days after Week 52/End of Treatment [EOT]). The end of trial is defined as the date the last trial participant completes the last FU/EOS visit (at Week 51 or Week 55). If participants discontinue IMP prematurely, they will return to the site for an early treatment discontinuation (ETD) Visit within 7 days after last dose of IMP and will be asked to continue the trial visits as planned until end of the trial (see Section 3.3.4.1 for details).

Participants who discontinue IMP prematurely will be asked to continue the trial visits as planned (see also Section 3.3.4.1). All efforts should be made to conduct the Week 48 visit in clinic, in particular the MRI-PDFF and body weight measurements, for all participants, including those who discontinued IMP prematurely. However, if this is not possible, a phone or telemedicine contact should be made at Week 51 to obtain vital status. If possible, adverse events (AEs), and concomitant therapy changes should be recorded.

All trial participants will receive counselling for diet and physical activity (see Section 4.2.1.2).

During the trial, participants should not initiate any anti-obesity medication. GLP-1R agonists, GLP-1R agonist/insulin/GIP combinations, amylin analogues, and DPP4i are not allowed while on study treatment (see also Section 4.2.2.1).

3.2 DISCUSSION OF TRIAL DESIGN, INCLUDING THE CHOICE OF CONTROL GROUP(S)

A randomised, double blind, placebo-controlled design was chosen for this trial as an accepted standard trial design for a Phase III trial. The treatment duration of 48 weeks was chosen as sufficient to potentially show changes in liver fat and body weight. A placebo control was included to evaluate the absolute effects of survodutide on efficacy and safety. This is acceptable as no standard treatments for NASH and/or fibrosis are available and in accordance with the guidelines provided by regulatory authorities.

3.3 SELECTION OF TRIAL POPULATION

A sufficient number of subjects will be screened in approximately 56 sites and approximately 2-8 countries to randomise approximately 160 trial participants.

3.3.1 Main diagnosis for trial entry

The main diagnosis required for trial entry is presumed or confirmed NASH and overweight or obesity.

Please refer to Section 8.3.1 (Source Documents) for the documentation requirements pertaining to the inclusion and exclusion criteria.

Proprietary confidential information © 2025 Boehringer Ingelheim International GmbH or one or more of its affiliated companies

3.3.2 Inclusion criteria

For an eligible participant, all inclusion criteria must be answered "yes" at screening and re-confirmed at baseline (i.e. before randomisation), except for laboratory tests for which screening results are used for randomisation.

1. Age ≥18 years at the time of signing informed consent, and at least the legal age of consent in countries where it is >18 years
2. Signed and dated written informed consent in accordance with ICH-GCP and local legislation prior to admission to the trial
3. BMI ≥30 kg/m^2, OR BMI ≥27 kg/m^2 and at least one weight-related comorbidity at screening:
   - Hypertension (defined as repeated, i.e. at least 3 measurements in resting condition, SBP values of ≥140 mmHg and/or DBP values of ≥90 mmHg in the absence of anti-hypertensive treatment, or intake of at least 1 anti-hypertensive drug to maintain a normotensive blood pressure)
   - Dyslipidaemia (defined as at least 1 lipid-lowering treatment required to maintain normal blood lipid levels, or LDL cholesterol ≥160 mg/dL (≥4.1 mmol/L) or triglycerides ≥150 mg/dL (≥1.7 mmol/L), or HDL cholesterol <40 mg/dL (<1.0 mmol/L) for men or HDL cholesterol<50 mg/dL (<1.3 mmol/L) for women
   - Obstructive sleep apnoea
   - Cardiovascular disease (e.g. heart failure with New York Heart Association [NYHA] functional class II-III, history of ischaemic or haemorrhagic stroke or cerebrovascular revascularisation procedure [e.g. carotid endarterectomy and/or stent], MI, coronary artery disease, or peripheral vascular disease)
   - T2DM (diagnosed at least 180 days prior to screening, with glycated haemoglobin [HbA_{1c}] ≥6.5% [≥48 mmol/mol] and <10% [<86 mmol/mol] as measured by the central laboratory at screening)
4. History of at least one self-reported unsuccessful dietary effort to lose body weight
5. Presumed/confirmed NASH: Evidence of hepatic steatosis (defined by an MRI-PDFF ≥8% at screening) with the exclusion of secondary causes of hepatic fat accumulation such as significant alcohol consumption, other chronic liver diseases and/or steatogenic medications, AND at least one of the following:
   - MRE ≥2.61 and <4.68 kPa at screening, OR
   - MAST score ≥0.242 at screening, when MAST score = exp(MAST)/(1+exp[MAST]) where MAST = -12.17 + 7.07 logMRE + 0.037 PDFF + 3.55 log AST [R24-0245], OR
   - FAST score ≥0.5 at screening (see Section 2.2.2 for calculation), OR
   - FibroScan® VCTE™ ≥8 kPa and <20 kPa at screening, OR
   - FIB-4 score ≥2.67 and <3.48 at screening, OR
   - ELF score >7.7 and <11.3 at screening, OR
   - cT1 ≥875 ms at screening, OR

Proprietary confidential information © 2025 Boehringer Ingelheim International GmbH or one or more of its affiliated companies

- Recent liver biopsy (within 3 years of screening) consistent with NASH (defined as the presence of steatosis, inflammation, and ballooning) with stage ≤3 fibrosis according to the NASH Clinical Research Network classification [R16-0241].

6. WOCBP[1] must be ready and able to use highly effective methods of birth control per ICH M3 (R2) that result in a low failure rate of less than 1% per year when used consistently and correctly. A list of contraception methods meeting these criteria and instructions on the duration of their use will be provided in the participant information and Section 10.3.
7. In the investigator's opinion, participants are well-motivated, capable and willing to:
   - Learn how to self-inject the IMP, as required for this protocol (persons with physical limitations who are not able to perform the injections must have the assistance of an individual trained to inject the IMP) OR
   - Inject the IMP or accept injection from a designated person
   - Follow study procedures for the duration of the study, including, but not limited to: follow lifestyle advice (for example, dietary restrictions and exercise, and exercise plan), maintain a diary, complete required questionnaires, and handle the IMP as described in the IFU

3.3.3 Exclusion criteria

For an eligible participant, all the following exclusion criteria must be answered "no" at screening and re-confirmed at baseline (i.e. before randomisation), except for laboratory tests for which screening results are used for randomization.

Liver:

1. Current or history of significant alcohol consumption (defined as intake of >210 g/week in men and >140 g/week in women on average over a consecutive period of more than 3 months) or inability to reliably quantify alcohol consumption based on the investigator's judgement within the last 5 years.
2. Intake of medications associated with liver injury, hepatic steatosis or steatohepatitis (refer to Section 4.2.2.1).
3. History of other chronic liver diseases (e.g. viral hepatitis, autoimmune liver disease, primary biliary cholangitis, primary sclerosing cholangitis, Wilson's disease, hemochromatosis, A1At deficiency, history of liver transplantation). Hepatitis B and C testing will be done at Visit 1. Participants with positive HBsAg should be excluded. Participants treated for hepatitis C must have a negative RNA test at screening and also be HCV RNA negative for at least 3 years prior to screening in order to be eligible for the trial. Trial participants with positive HCV antibody and no history of HCV treatment require a negative HCV RNA test at screening to be eligible for the trial.
4. Cirrhosis based on clinical assessment, abdominal imaging, liver histology or non-invasive tests assessed at screening (ELF ≥11.3, FIB4 ≥3.48, FibroScan® VCTE™ ≥20 kPa, or MRE ≥4.68 kPa), or a history of cirrhosis.

---

[1] A woman is considered of childbearing potential, i.e. fertile, following menarche and until becoming post-menopausal unless permanently sterile. Permanent sterilisation methods include hysterectomy, bilateral salpingectomy, and bilateral oophorectomy. Tubal ligation is NOT a method of permanent sterilisation. A postmenopausal state is defined as no menses for 12 months without an alternative medical cause.

Proprietary confidential information © 2025 Boehringer Ingelheim International GmbH or one or more of its affiliated companies

5. Current decompensated liver disease or previous hepatic decompensation (ascites, spontaneous bacterial peritonitis, portal hypertension bleeding, hepatic encephalopathy, hepatorenal syndrome).
6. Previous or current evidence of portal hypertension (e.g. splenomegaly, oesophageal varices, or other portosystemic collateral pathways).
7. Any of the following liver laboratory test abnormalities at screening:
   - Serum AST and/or ALT elevation ≥5x ULN
   - Total serum bilirubin concentration ≥1.2x ULN (except for cases of known Gilbert's Syndrome)
   - Alkaline phosphatase >2x ULN
   - INR ≥1.3 (unless participant is on anticoagulants)

Obesity:

8. Body weight variation (self-reported) >5% within 3 months before screening
9. Medications for obesity within 3 months before screening (see Section 4.2.2.1)
10. Previous or planned (during the trial period) treatment for obesity with surgery or a weight loss device, or prior surgery of the GI tract that could interfere with body weight. The following are allowed: (1) liposuction and/or abdominoplasty, if performed >1 year before screening, (2) lap banding, if the band has been removed >1 year before screening, (3) intragastric balloon, if the balloon has been removed >1 year before screening, (4) duodenal-jejunal bypass sleeve, if the sleeve has been removed >1 year before screening (5) appendectomy, (6) simple hernia repair, or (7) cholecystectomy.
11. Obesity induced by other endocrinologic disorders (i.e. Cushing Syndrome) or diagnosed monogenetic or syndromic forms of obesity (i.e. melanocortin 4 receptor deficiency, leptin deficiency, or Prader Willi Syndrome)

Diabetes:

12. Treatment with medications for the indication of T2DM as described in Section 4.2.2.1
13. Uncontrolled and potentially unstable diabetic retinopathy or maculopathy, verified by an eye examination within 3 months before screening or in the period between screening and randomisation (for details, see Section 5.2.5.2)

Mental health:

14. Answered "yes" to any of the suicide-related behaviours (actual attempt, interrupted attempt, aborted attempt, preparatory act or behaviour) or to the non-suicidal self-injurious behaviour question on the "Suicidal Behaviour" section of the C-SSRS related to the past 2 years before screening visit up to and including the randomisation visit
15. Answered "yes" to Question 4 (active suicidal ideation with some intent to act, without specific plan) or to Question 5 (active suicidal ideation with specific plan and intent) on the "Suicidal Ideation" section of the C-SSRS from 3 months before screening visit up to and including the randomisation visit.
16. Reported a history of psychiatric inpatient hospitalisation (due to significant active or unstable major depressive disorder or other severe psychiatric disorder, e.g. schizophrenia, bipolar disorder, or other serious mood or anxiety disorder) within the past 1 year before screening, OR are not stabilised on their psychiatric medications (i.e. change of medication type or dosage adjustment within 8 weeks prior to screening visit or until randomisation), OR major depressive symptoms defined as a PHQ9 score of ≥15 at

Proprietary confidential information © 2025 Boehringer Ingelheim International GmbH or one or more of its affiliated companies

screening or during the screening period (based on investigator's judgement, this assessment can be repeated once within the screening window).

17. Participants with major depressive disorder or generalized anxiety disorder whose disease state is considered stable and expected to remain stable throughout the course of the study, in the opinion of the investigator, may be considered for inclusion if they are not on excluded medications.

Other medical:

18. Any treatment with medications which may cause significant weight change started within 3 months prior to screening (included but not limited to systemic glucocorticoid therapy, tricyclic antidepressants, atypical antipsychotic, mood stabilisers, anti-hypertensive, antihistamines, and anti-epileptics; refer to Table 8). Participants taking these medications for more than 3 months prior to screening with stable body weight are allowed.
19. Known clinically significant gastric emptying abnormality (e.g. severe diabetic gastroparesis or gastric outlet obstruction)
20. Uncontrolled hypertension (mean SBP ≥160 mmHg and/or mean DBP ≥100 mmHg, measured in at least 2 measurements performed 5 min apart) at screening (assessed as described in Section 5.2.2).
21. Uncontrolled (as per investigators assessment) hypo- or hyperthyroidism at screening
22. Renal impairment measured as eGFR <30 mL/min/1.73 m^2 by the central laboratory at screening
23. History of either chronic or acute pancreatitis or elevation of serum lipase or serum pancreatic amylase >2x ULN as measured by the central laboratory at screening
24. Major surgery performed within 3 months prior to randomisation or planned for the duration of the trial
25. Personal or family history of medullary thyroid carcinoma or multiple endocrine neoplasia syndrome type 2
26. Calcitonin ≥100 ng/L (29.26 pmol/L) as measured by the central laboratory at screening
27. Recent evidence (within 3 months prior to screening) of acute or unstable cardiovascular events, e.g. hospitalisation for HF, acute coronary syndrome, unstable angina, MI, ischaemic or haemorrhagic stroke, transient ischaemic attack, and/or acute peripheral vascular event
28. HF with NYHA functional class IV
29. QTc (Fridericia) mean interval that is greater than 500 ms at screening (triplicate ECG) or personal or family history of long QT syndrome
30. History of infection with HIV or positive HIV test at screening
31. History of organ transplantation (corneal transplants [keratoplasty] allowed) or awaiting an organ transplant
32. Haematological condition that may interfere with HbA_{1c} measurement (e.g. haemolytic anaemias, sickle cell disease)
33. Women who are pregnant, nursing or who plan to become pregnant while in the trial
34. Known or suspected hypersensitivity to the IMP or related products

Proprietary confidential information © 2025 Boehringer Ingelheim International GmbH or one or more of its affiliated companies

35. Contraindication to MRI including, but not limited to, severe claustrophobia, inner ear implant, pacemakers or other implanted cardiac rhythm management devices, intracranial aneurism clips incompatible with MRI, any other metallic, non-MRI compatible implanted devices (e.g. hip joint replacement), a history of intra-orbital metal fragments that have not been removed, and weight or girth that exceeds scanner capabilities.
36. Confirmed diagnosis of malignancy (including remission) within 5 years prior to screening, except for basal- or squamous-cell carcinoma of the skin that has been treated successfully. Trial participants under evaluation for malignant disease currently are not eligible for trial participation
37. Platelet count <140 000/mm^3

General:

38. Participants not expected to comply with the protocol requirements or not expected to complete the trial as scheduled (e.g. chronic alcohol or drug abuse or any other condition that, in the investigator's opinion, makes the participant an unreliable trial participant)
39. Participants who must or wish to continue the intake of prohibited medication (see Section 4.2.2.1) or any drug considered likely to interfere with the safe conduct of the trial
40. WOCBP who are using oral contraceptives during the screening visit and are
    - Not willing to change to non-oral contraceptives at least 7 days prior to first dose of IMP

    or

    - Not willing to add an additional barrier method of contraception during the entire treatment period with IMP and 28 days thereafter (see Appendix 10.3)
41. Currently enrolled in another investigational device or drug trial, or less than 30 days or 5 half-lives, whichever is longer, since ending another investigational device or drug trial or receiving other investigational treatment(s); current or previous enrolment in strictly observational trials is allowed
42. Previous randomisation in this trial
43. Any condition not covered by any of the other exclusion criteria, which in the investigator's opinion, might jeopardise the participant's safety or compliance with the protocol, or may interfere with the trial objectives

3.3.4 Discontinuation of trial participants from treatment or assessments

Trial participants may discontinue trial IMP or withdraw consent to trial participation as a whole ("withdrawal of consent") with very different implications; please see Sections 3.3.4.1 and 3.3.4.2.

Trial participants should be informed of anticipated GI side effects (see Section 4.1.4.1), and investigators should provide guidance on how to avoid or overcome them.

Measures to control the withdrawal rate include careful trial participant selection, appropriate explanation of the trial requirements and procedures prior to trial enrolment, as well as the explanation of the consequences of withdrawal.

Proprietary confidential information © 2025 Boehringer Ingelheim International GmbH or one or more of its affiliated companies

The decision to discontinue trial IMP or withdraw consent to trial participation and the reason (if provided) must be documented in the trial participant files and CRF. If applicable, consider the requirements for AE collection and reporting (see Section 5.2.6.2).

3.3.4.1 Discontinuation of trial treatment

The participant must be discontinued from IMP if the following applies:

- The trial participant wants to discontinue the IMP. The trial participant will be asked to explain the reasons but has the right to refuse to answer.
- The trial participant has repeatedly shown to be non-compliant with important trial procedures and, in the opinion of both the investigator and sponsor representative, the safety of the trial participant cannot be guaranteed as he/she is not willing or able to adhere to the trial requirements in the future.
- Pregnancy or intention of becoming pregnant of female trial participant. In case of pregnancy, the trial participant will be expected to attend the regularly scheduled study visits as outlined above, with the exception of any imaging assessments. She will be followed up during the trial and until birth or termination of the pregnancy.
- When pancreatitis is suspected, IMP should be stopped. If pancreatitis is confirmed, IMP should be discontinued.
- In case of evidence of mean QTc (Fridericia) interval >500 ms or an increase in mean QTcF interval more than 60 ms AND mean QTcF interval above the ULN (470 ms for women, 450 ms for men) compared with the last ECG recording performed prior to the first IMP administration obtained from central ECG reading. If these changes are seen in a local ECG reading, IMP should be temporarily interrupted and then discontinued, if this finding is confirmed by central ECG reading. If any local ECG is needed due to any acute symptom/event (e.g. syncope), QTcF should be measured. In the case of conditions with broad QRS complex, such as newly developed bundle branch block (BBB) or ventricular pacing, an increase in QTcF interval >60 ms compared to baseline should be evaluated by the Investigator after consultation with a cardiologist to determine whether there is a true QTcF prolongation.
- Suicidality (see Section 5.2.5.1 for further safety measures):
    - Answered “yes” to either Question 4 (Active Suicidal Ideation with Some Intent to Act, Without Specific Plan) or to Question 5 (Active Suicidal Ideation with Specific Plan and Intent) on the “Suicidal Ideation” section of the C-SSRS since last visit
    - Answered “yes” to any of the suicide-related behaviours (Actual attempt, Interrupted attempt, Aborted attempt, Preparatory act or behaviour) or to the non-suicidal self-injurious behaviour question on the “Suicidal Behaviour” section of the C-SSRS since last visit

Proprietary confidential information © 2025 Boehringer Ingelheim International GmbH or one or more of its affiliated companies

- PHQ-9 score ≥15

  Trial participants should be referred to an MHP to assist with participant's condition evaluation. If a trial participant's psychiatric disorder can be adequately treated with psycho- and/or pharmacotherapy, then the participant, at the discretion of the investigator (in agreement with the MHP), may be continued in the trial on randomised therapy. If the trial participant refuses to be referred to an MHP, the investigator will decide if the participant can remain in the trial.
- Initiation of prohibited medications as per Section 4.2.2 if the trial participant will not or cannot discontinue them
- Bariatric surgery
- Intolerable GI symptoms despite management as described in Section 4.1.4.1.
- >3 doses are missed (see Table 6)
- BMI ≤18.5 kg/m^2 is reached at any time during the trial. If a trial participant with any BMI above 18.5 kg/m^2 wants to discontinue the IMP because the trial participant does not want to lose any more weight, the investigator should propose a dose reduction to prevent the discontinuation from IMP treatment, but not lower than 2.4 mg
- Diagnosis of T1DM
- Worsening or new onset of diabetic retinopathy or maculopathy, <u>if deemed clinically significant by the Investigator</u>
- Diagnosis of any malignancy (other than basal or squamous-cell skin cancer, in-situ cancers) after randomisation
  - <u>Cancers that can be considered cured after surgical resection, e.g. prostate cancer should be discussed with the sponsor.</u>
- Elevation of calcitonin ≥100 pg/L (29.26 pmol/L)
- The trial participant develops a clinically significant elevation of liver enzymes. Refer to Section 5.2.6.1.4.
- Development of any significant IMP related hypersensitivity reaction, as per investigator's opinion
- Severe HS reaction or anaphylactic reaction (see Section 4.2.1.3)
- Occurrence of any other treatment-emergent AE, SAE, or clinically significant finding for which the investigator believes that permanent IMP discontinuation is the appropriate measure to be taken
- Emergency unblinding of participant´s treatment assignment by the investigator

In case a trial participant was mistakenly enrolled in the study (did not meet inclusion criteria or met exclusion criteria at screening/ randomisation), the participant's continuation on the trial treatment will be discussed with the Sponsor Study Physician.

Early treatment discontinuation (ETD) and follow-up (FU) Visits

A participant who discontinues treatment prematurely during the trial should undergo an ETD Visit 7 days after the last IMP dose or, if more than 7 days passed, as soon as possible. For participants who are willing to continue with trial assessments, the next scheduled trial visit

Proprietary confidential information © 2025 Boehringer Ingelheim International GmbH or one or more of its affiliated companies

may be skipped if the ETD visit is completed within 14 days (for participants with ETD up to Visit 6) or within 28 days (for participants with ETD between Visit 6 to Visit 15) prior to the scheduled visit.

21 days after the ETD Visit and at least 28 days after the last IMP dose a FU Visit should be conducted. For trial participants who are willing to continue with scheduled trial assessments, the FU Visit may be combined with EOS Visit if FU visit took place less than 28 days before the scheduled EOS visit. Otherwise, the FU and EOS are two separate visits. If the FU visit is completed within 14 days (for participants with ETD up to Visit 6) or within 28 days (for participants with ETD between Visit 6 to Visit 15) prior to the next regular scheduled on-site visit, the visits can be combined. Sites should encourage the participant to return to the clinic for the FU visit.

Trial participation after IMP discontinuation

For participants who prematurely discontinue the IMP, all efforts should be made to observe these participants and ask them to continue to attend the scheduled visits until the end of the trial. If possible, at least all applicable imaging assessments, body weight, AEs, and concomitant therapy changes should be recorded at clinic visits. At a minimum the ETD visit 7 days after the last IMP dose or, if more than 7 days passed, as soon as possible, and the FU visit should be done. Preferably, these visits should be in-clinic to allow for safety monitoring . If the participant is not willing or able to attend the clinic, the ETD and/or Week 48 visit can be performed by a site’s HCP or vendor at the participant’s home, if available and acceptable according to local law and regulations. In this case the assessments will be restricted to collection of weight, waist/hip circumference, vital signs, information on AE and substance usage. The FU/EOS Visit may be conducted via a remote visit (if allowed per local regulations) or over the phone for review of medical records. For participants who prematurely discontinued the IMP and are willing to continue in the trial and attend clinic visits, the FU/EOS at Week 51 should only include collection of vital status, AEs/AESIs/SAEs, concomitant medication and samples for ADA/Nab.

For primary endpoint data analysis every effort should be made to obtain MRI-PDFF data as close to the scheduled time points as possible, and participants should be encouraged to return for the MRI-PDFF assessments, especially for the assessment at Week 48.

For participants who discontinue after Week 28 and who are not sure that they will return at Week 48, an MRI should be scheduled at their FU Visit (which takes place 21 days after their individual ETD). For participants who had an MRI-PDFF at their FU Visit and are willing to come back at Week 48, an MRI-PDFF should also be scheduled at Week 48.

For participants who discontinue before Week 20 and who are not sure that they will return at Week 28 and Week 48, an MRI should be scheduled at their FU Visit (which takes place 21 days after their individual ETD). For participants who had an MRI-PDFF at their FU Visit and are willing to come back at Week 28 and Week 48, an MRI-PDFF should be scheduled also at Week 28 and Week 48. Week 48 MRI-PDFF has always highest priority due to its relevance for primary endpoint data analysis.

Proprietary confidential information © 2025 Boehringer Ingelheim International GmbH or one or more of its affiliated companies

For participants who discontinue after Week 20 but before Week 28 and who are not sure that they will return at Week 28 and Week 48, an MRI should be scheduled at their FU Visit (which takes place 21 days after their individual ETD). For participants who had an MRI-PDFF at their FU Visit and are willing to come back at Week 28 and Week 48, an additional MRI-PDFF should be scheduled only at Week 48.

Please note that not only MRI-PDFF, but also liver volume, MRE, and cT1 data should be collected for all MRI examinations.

The data collection strategy for MRI for participants who prematurely discontinue the IMP is also valid for FibroScan®.

Note that participants who prematurely discontinue IMP due to pregnancy are not to undergo any imaging assessments for trial purposes.

Participants should be asked to choose the most rigorous form of follow-up that they are willing to comply with.

Every attempt must be made by the investigator to ensure a participant continues participating in the trial during IMP interruptions and after discontinuation of the IMP. At a minimum the ETD visit 7 days after the last IMP dose or, if more than 7 days passed, as soon as possible, and the FU visit should be done. Participants that are not actively taking IMP may be less motivated to adhere to the scheduled trial visits. In potential cases, when a trial participant is no longer willing to complete all follow-up measures, the following sequence of options should be considered and discussed if allowed by local regulations:

- Option 1: Continue to attend as many regularly scheduled trial visits as possible at the centre until the trial ends, but definitely have Week 48 visit performed on site, including the MRI scan. It is recommended to complete all assessments during these visits. If the participant declines to come to the site, collection of Week 48 body weight data by a medically qualified and protocol-trained person using site-supplied equipment should be attempted.
- Option 2: Have Week 48 visit performed on site, including the MRI scan. Conduct as many of the remaining trial visits via remote visits (if allowed per local regulations) or over the phone. It is recommended to complete all assessments, that can be conducted remotely, during these visits. If the participant declines to come to the site, collection of Week 48 body weight data by a medically qualified and protocol-trained person using trial-specific and site-supplied equipment should be attempted.
- Option 3: Conduct only the Week 48 visit on site, including the MRI scan, but discontinue participation in any other trial visits. If the participant declines to come to the site, collection of Week 48 body weight data by a medically qualified and protocol-trained person using trial-specific and site-supplied equipment should be attempted.
- Option 4: Discontinue participation in remaining trial activities but provide vital status at the end of the trial (Week 51) through the participant, alternative person designated by the participant, or through review of participant's medical information from alternative sources (e.g. doctor's notes, hospital records, etc.).

Proprietary confidential information © 2025 Boehringer Ingelheim International GmbH or one or more of its affiliated companies

- Option 5: Discontinue participation in remaining trial activities; only ETD and FU visits will be done.

Please note that with Options 4 and 5 no primary endpoint data at Week 48 will be obtainable for the participant; this should be avoided as much as possible.

Please note that any imaging assessments are not to be conducted in pregnant participants for trial purposes, who would have discontinued IMP.

Participants should be asked to choose the most rigorous form of follow-up that they are willing to comply with. Participants who choose Option 3 or Option 4 should be contacted over the phone approximately every 2 months to maintain contact until Week 48. Any information on safety and outcome events collected during this contact should be documented in medical records.

In case of a temporary reason, IMP should be restarted if medically justified; please see Section 4.1.4. Any planned temporary discontinuation due to a medical condition requires discussion with the Sponsor Study Physician.

Hepatic injury

If DILI adjudication reveals that Survodutide is responsible for hepatic injury in 3 or more subjects, Boehringer Ingelheim will pause enrolment of new participants in the trial and, taking into consideration the DMC recommendations, discontinue the trial treatment for all participants, if needed.

If new efficacy/safety information becomes available, BI will review the benefit-risk assessment and, if needed, pause or discontinue the IMP treatment for all trial participants or take any other appropriate action to guarantee the safety of the trial participants.

3.3.4.2 Withdrawal of consent to trial participation

A participant has the right to withdraw informed consent for participation at any time without the need to justify the decision.

Withdrawal of consent to trial participation means:

- Permanently stopping the trial treatment
- No further participation in ANY further study visits or study assessments
- No further contact with the participant
- No further data collection (e.g. through participant's representative or via participant's medical records, as permitted by local regulations)

The participant should stop taking IMP and will be asked to complete the ETD Visit and follow-up procedures as described in the SoA and Section 3.3.4.1. Completing these procedures is strongly recommended for the participant's safety.

Proprietary confidential information © 2025 Boehringer Ingelheim International GmbH or one or more of its affiliated companies

If a trial participant wants to withdraw consent, the investigator should be involved in the discussions with the participant and explain the difference between trial treatment discontinuation and withdrawal of consent to trial participation, as well as explain the participant's options for continued follow-up after trial treatment discontinuation described at in Section 3.3.4.1. If the participant withdraws informed consent for participation in the trial, the trial will end for that participant. Participants that withdraw informed consent will not be replaced.

3.3.4.3 Discontinuation of the trial by the sponsor

Boehringer Ingelheim reserves the right to discontinue the trial overall or at a particular trial site at any time for the following reasons:

1. Failure to meet expected enrolment goals overall or at a particular trial site
2. New efficacy or safety information invalidating the earlier positive benefit-risk assessment; please see Section 3.3.4.1
3. Deviations from GCP, the trial protocol, or the contract impairing the appropriate conduct of the trial

Further treatment and follow up of trial participants affected will occur as described in Section 3.3.4.1.

The investigator/trial site will be reimbursed for reasonable expenses incurred in case of trial termination (except in case of the third reason).

3.3.4.4 Lost to follow-up

If a participant is lost, every effort will be made by the investigator and site staff to contact and locate the participant before the participant is declared lost to follow-up. Investigators and site staff must use every possible allowable means, according to local regulations, to locate participants who have missed visits. Efforts to contact the participant may include but are not limited to:

- Calling all numbers for participant and listed contacts
- Calling primary care physician, referring specialist and/or other listed physicians for more recent information, date of last office visit or to determine vital status
- Sending an email and follow up with mailing certified letters (return receipt requested) to all known participant addresses and all listed contacts that were provided by the participant
- Reviewing participants records and medical notes for any details of a hospitalisation doctor's visit or other procedure that may indicate location or status of participant
- Use internet to search for possible contact information for the participant
- Try reverse directory for phone numbers to get possible addresses and/or new contact details
- Check local, regional, and national public records to locate the participant or search for vital status in accordance with local law
- Consider home visit

Proprietary confidential information © 2025 Boehringer Ingelheim International GmbH or one or more of its affiliated companies

- Use third party vendor in compliance with local legislation to contact participant

The following actions must be taken if a participant fails to return to the clinic for a required study visit:

- The site must attempt to contact the participant and reschedule the missed visit as soon as possible and counsel the participant on the importance of maintaining the assigned visit schedule and ascertain whether or not the participant wishes to and/or should continue in the trial.
- Before a participant is deemed lost to follow-up, the investigator or designee must make every effort to regain contact with the participant (where possible, 3 telephone calls and, if necessary, a certified letter to the participant's last known mailing address or local equivalent methods). These contact attempts should be documented in the participant's medical record.
- Should the participant continue to be unreachable, they will be considered lost to follow-up.

Proprietary confidential information © 2025 Boehringer Ingelheim International GmbH or one or more of its affiliated companies

4. TREATMENTS

4.1 INVESTIGATIONAL TREATMENTS

4.1.1 Identity of the Investigational Medicinal Products

Table 2 Test product

Substance:	Survodutide
Pharmaceutical formulation:	Solution for injection
Source:	Boehringer Ingelheim Pharma GmbH & Co. KG, Biberach Germany
Unit strength:	Filling volume: 0.5 mL<br>Concentrations: 0.3 mg/0.5 mL, 0.6 mg/0.5 mL, 1.2 mg/0.5 mL, 2.4 mg/0.5 mL, 3.6 mg/0.5 mL, 4.8 mg/0.5 mL, and 6.0 mg/0.5 mL
Posology:	Once weekly (one pre-filled syringe)
Method and route of administration:	s.c. injections

Table 3 Comparator product

Substance:	Placebo
Pharmaceutical formulation:	Solution for injection
Source:	Boehringer Ingelheim Pharma GmbH & Co. KG, Biberach Germany
Unit strength:	Filling volume: 0.5 mL
Posology:	Once weekly (one pre-filled syringe)
Method and route of administration:	s.c. injections

4.1.2 Selection of doses in the trial and dose modifications

Maintenance dose of 6.0 mg survodutide will be investigated. Dose escalation will be performed over 24 weeks after randomisation and will consist of stepwise dose increases every 4 weeks from the starting dose of 0.3 mg and consecutive escalation doses of 0.6, 1.2, 2.4, 3.6, 4.8, and 6.0 mg.

The dosing regimens were selected based on the following considerations:

- Data from trial participants with obesity or overweight up to 6 weeks for doses ranging from 0.7 to 3.15 mg/week (daily dosing) and 0.3 to 3 mg/week (weekly dosing), and later up to 16 weeks for doses ranging from 0.6 to 4.8 mg/week (weekly or twice weekly dosing from trial 1404-0003 [c26759941].
- Safety and tolerability of survodutide evaluated in a dose-finding trial 1404-0036 [c31754142] in 387 trial participants with overweight or obesity using once weekly injections of 0.6, 2.4, 3.6, and 4.8 mg or placebo.

Proprietary confidential information © 2025 Boehringer Ingelheim International GmbH or one or more of its affiliated companies

- The selected up-titration scheme is more spaced-out (every 4 weeks) compared with earlier studies where the dose was escalated every 2 weeks. This is expected to improve the overall tolerability profile. In particular, simulations with exposure-response models developed based on Phase I and Phase II (data on file) predict decreased frequency of nausea and vomiting with the selected dosing regimen compared with earlier studies, especially during the early dose escalation phase, which is known to be associated with the highest discontinuation rate due to GI tolerability. Additionally, more flexibility has been included (e.g. the dose escalation period in Phase III will be extended up to a maximum of 32 weeks if needed to mitigate GI side effects; participants will have the option of temporary interruption of IMP; they will also be able to reduce the IMP dose to the next lower dose with the possibility to re-escalate to the randomised dose). Planned up-titration scheme and flexibility to interrupt or reduce the dose is expected to improve the overall tolerability profile in Phase III program and enable more trial participants to reach and stay on the target dose.
- The model-predicted average heart rate increase in the 6.0 mg group for this Phase III study is comparable with that simulated for the dose groups tested in the Phase II study 1404-0036 (data on file), where no cardiac safety signals were detected.
- Based on the no observed adverse effect level in male cynomolgus monkeys in a 39-week study, the 6.0 mg dose had a safety margin of 3.03 for $C_{max,ss}$ and 2.98 for $AUC_{\tau,ss}$ (data on file).
- The 6.0 mg dose is expected to enhance the effect of GCGR agonism in lipid and fatty acid oxidation in the liver [c14085752].

4.1.3 Method of assigning trial participants to treatment groups

After the assessment of all inclusion and exclusion criteria, each eligible trial participant will be randomised to a treatment group according to a randomisation plan (see Section 7.4). Randomisation codes will be generated through a validated software and kept blinded to the trial team, sites, and trial participants. Access to the codes will be controlled and documented. An Interactive Response Technology (IRT) system will be used to screen participants, create a participant number, perform treatment assignment, manage initial/re-supply ordering of IMP supplies, and handle emergency unblinding.

Randomisation will be stratified by the presence of T2DM.

In addition, an adjustable screening cap will be implemented in the IRT system to ensure that enough male and female trial participants are enrolled and randomised. Cap for number of female participants is approximately 70% of all randomised participants.

4.1.4 Drug assignment and administration of doses for each trial participant

Planned dose escalation of survodutide/placebo takes place up to 24 weeks after randomisation as displayed in Table 4 (approximately 4 weeks between each dose escalation; up to a maximum of 32 weeks, if 1 or 2 re-escalation attempts are needed due to GI symptoms). Administration of survodutide/placebo at Week 24 marks the end of the planned dose escalation period and the start of the maintenance period.

Proprietary confidential information © 2025 Boehringer Ingelheim International GmbH or one or more of its affiliated companies

Table 4 Dose escalation scheme

**Treatment week**	**0**	**1**	**2**	**3**	**4**	**5**	**6**	**7**	**8**	**9**	**10**	**11**	**12**	**13**	**14**	**15**	**16**	**17**	**18**	**19**	**20**	**21**	**22**	**23**	**End of dose escalation**
**Visit number**	**V2**		**V3**		**V4**		**V5**		**V6**				**V7**				**V8**				**V9**				
**Escalation**	**Starting dose**				**1 st**				**2nd**				**3rd**				**4th**				**5th**				**6th**
Survodutide	0.3 mg				0.6 mg				1.2 mg				2.4 mg				3.6 mg				4.8 mg				6.0 mg (final)
Placebo	Placebo																								

Participants will receive the IMP until Week 47 or, if they have agreed to the optional extended treatment period, until Week 51. Participants will be instructed to inject the IMP once weekly at the same day of the week to the extent possible (±1 day). A weekly reminder for IMP administration will be sent to the trial participants through a digital support platform to allow trial participants to follow their weekly dosing schedule closely. Trial participants who are not willing to use the digital platform should be closely followed up and monitored for compliance by the site.

The injection day may not match the visit day. If the injection date is planned during the in-clinic site visit, the trial participant should not administer the IMP prior to the visit but after the actual visit to allow PK sampling to be done prior to the injection. The first IMP dose administered at Visit 2 should be taken at the site. For all consequent PK visits, the IMP for injections at the site should be taken from the newly dispensed kit.

During the in-clinic visits, the trial participants will receive IMP to cover the time period until the next scheduled visit. The participants may receive spare kits as a back-up in case of failed injection or damage to injector. The spare kits should not be used unless instructed otherwise by the site. Participants should record the date and time of all dose administrations in their eDiary. Any unused kit should be returned to the site.

Instructions and training will be given to the participants, and participants will receive the IFU. Handling kits will be provided in some countries to support training at the in-clinic visit. Training will be repeated at time points specified in the SoA.

Injections should be administered in the abdomen at any time of day irrespective of meals as described in the IFU.

Participants should aim to reach the randomised maintenance dose. [redacted]

Proprietary confidential information © 2025 Boehringer Ingelheim International GmbH or one or more of its affiliated companies

[redacted]. Thorough documentation of all measures taken to improve tolerability, including counselling on dietary behaviours, symptomatic medication (antiemetic or antidiarrheal), temporary interruption of IMP, and outcomes of the discussion with sponsor representative, is required in a specific eCRF page in case of a dose reduction. For further details, please refer to Sections 4.1.4.1 and 10.2. For trial participants with GI symptoms, the IMP may be dispensed during an unscheduled visit.

Table 5 Dose reduction scheme (only applicable between Weeks 16 and 32)

**Last dose of survodutide or placebo**	**If not tolerated, reduce the dose to:**	**If not tolerated after re-escalation, reduce the dose to:**
2.4 mg or less	Discontinue IMP	Not applicable
3.6 mg	2.4 mg	2.4 mg (until EOT)
4.8 mg	3.6 mg	3.6 mg (until EOT)
6.0 mg	4.8 mg	4.8 mg (until EOT)

In case 1 or more IMP doses are missed, the instruction in Table 6 should be followed. A missed dose should not affect the dose escalation scheme or scheduled dosing day of the next week. A minimum of 5 days between weekly doses is required.

Table 6 Instructions in case of missed IMP dose(s)

**Number of missed doses**	**What to do**
1 dose of IMP	IMP administration as soon as noticed, but within 2 days after the original scheduled time point<br>If noticed more than 2 days after the original scheduled injection time point, no IMP administration until the next scheduled IMP injection
2 consecutive doses of IMP	Recommencement of IMP treatment if considered safe as per the investigator's discretion and if the participant does not meet any of the discontinuation criteria
3 consecutive doses of IMP	Consideration of treatment discontinuation. If the trial participant is willing to continue the treatment, and the investigator feels it is safe, the participant may resume dosing.
>3 consecutive doses of IMP	Treatment discontinuation<br>In some exceptional circumstances (e.g. COVID-19-like pandemic situation), the sponsor is to be contacted for an alternative option.

The residual effect period of survodutide is 28 days (half-life 112 h); therefore, a period of 28 days after last administration of the IMP will be assigned to the on-treatment period for evaluation.

In the event of force majeure or other disrupting circumstances (e.g. pandemic, war; see Section 6), in-clinic visits may not be feasible or may need to be restricted to ensure participant safety. Based on a thorough assessment of the benefits and risks, the investigator may still decide to continue IMP treatment. Where permitted by local law and regulations, the IMP may be shipped directly to the participants' home. In agreement with the sponsor, a home visit service or remote visit may assist the participant with IMP administration and/or

Proprietary confidential information © 2025 Boehringer Ingelheim International GmbH or one or more of its affiliated companies

completion of assessments performed by a specialized vendor/HCP, if available and acceptable according to local law and regulations.

In addition, home delivery of IMP is allowed upon special request of the trial participant, e.g. due to limited storage space at home. A specialised courier service has been contracted for the direct to participant IMP shipment. If requested, and if allowed by country regulations, this courier service will deliver IMP dispensed by site pharmacy directly to trial participants' home address. The courier service has special procedures in place to protect trial participant privacy data.

4.1.4.1 Management of gastrointestinal symptoms

For trial participants who experience intolerable GI symptoms, for example nausea, vomiting, or diarrhoea, the following steps should be taken:

- First, the trial participant should be counselled on dietary behaviours that may help mitigate nausea and vomiting. This may include eating smaller meals, splitting 3 daily meals into 4 or more smaller ones, stopping eating when they feel full, reducing fat intake, and not eating for 2-3 h before going to sleep. In case of vomiting or diarrhoea, the participant should be instructed to stay well hydrated.
- If symptoms persist, the participant should be prescribed symptomatic medication (antiemetic or antidiarrheal medication) at the investigator's discretion and kept hydrated.
- A temporary interruption of IMP for 1 dose is permitted.
- A temporary interruption of a second dose of IMP may be allowed at the investigator's discretion.
- The IMP should be resumed at the assigned dose immediately, alone or in combination with symptomatic medication, which can be utilised to manage symptoms.
- If, between Weeks 16 and 32 of treatment, the symptoms persist in spite of these measures, down-titration and subsequent re-escalation is allowed (see Table 5), after consultation with a sponsor representative. This should only be allowed if the participant would otherwise discontinue the IMP and if considered safe to continue the IMP, as per the investigator's discretion. Down-titration in this case means either reducing the dose to the next lower dose for 2 – 4 weeks (see Section 10.2) or delaying a scheduled up-titration by 2 – 4 weeks; both with the intent of returning to the planned dose escalation scheme once the participant's GI symptoms have become tolerable, and with an overall delay to the dose escalation not exceeding 8 weeks beyond the regular dose escalation's end at Week 24. If the last dose was 2.4 mg or less, the IMP has to be discontinued (Table 5 and Section 10.2).
- If the symptoms become tolerable or are completely resolved, it is recommended that the trial participant makes up to 2 attempts to re-escalate per dose escalation scheme to the assigned dose level, at the investigator's discretion. In such cases, the entire dose escalation period must not be longer than 32 weeks.
- If a trial participant does not tolerate the higher dose level after re-escalation or in case re-escalation is not considered safe as per investigator's discretion, the trial participant may stay at a lower dose level of 2.4 mg, 3.6 mg, or 4.8 mg once weekly until EOT/ETD.

Proprietary confidential information © 2025 Boehringer Ingelheim International GmbH or one or more of its affiliated companies

- For any changes in dose outside of the regularly scheduled steps, IMP of the new dose is dispensed during an unscheduled visit, and the participant must return all kits of the old dose.
- Thorough documentation of all measures taken to improve tolerability, including counselling on dietary behaviours, symptomatic medication (antiemetic or antidiarrheal), temporary interruption of IMP, and outcomes of the discussion with sponsor representative, is required in a specific eCRF page in case of a dose reduction.

For further details, please refer to Table 5 and Section 10.2.

4.1.5 Blinding and procedures for unblinding

4.1.5.1 Blinding

Trial participants, investigators, central reviewers, and everyone involved in the trial conduct or analysis or with any other interest in this double-blind trial will remain blinded regarding the randomised treatment assignments until the database is declared ready for analysis. Further details regarding the timepoint of unblinding the database for analysis are provided in the TSAP.

Access to the randomisation code will be kept restricted until its documented release per sponsor SOP. The DMC will be provided with unblinded data to allow them to review safety and to fulfil their tasks as outlined in the DMC charter. An independent team, not otherwise involved in the conduct of the trial, will provide the unblinded results to the DMC.

Randomisation codes will be provided to the external bioanalytical laboratory during the trial to allow PK samples and ADA/Nab samples taken from participants receiving placebo / comparator to be excluded from the analyses. In preparation for data transfer and bioanalytical report writing, the trial bioanalyst may receive unblinded data from the external bioanalytical laboratory after the last trial participant has completed the last visit but prior to official unblinding of the analysis database. The trial bioanalyst will not disclose the randomisation codes or bioanalytical results until the trial is unblinded after database lock.

4.1.5.2 Emergency unblinding and breaking the code

Emergency unblinding will be available to the investigator via IRT. It must only be used in an emergency when the identity of the trial drug must be known to the investigator in order to provide appropriate medical treatment or otherwise assure safety of trial participants. The treatment allocation should not be disclosed to the sponsor unless this is explicitly requested. The reason for unblinding must be documented in the source documents and/or appropriate eCRF page. Once unblinded, the trial treatment needs to be discontinued for the participant. However, the participant should remain in the trial if willing to continue follow up.

Due to the requirements to report SUSARs, it may be necessary for a representative from BI's Pharmacovigilance group to access the randomisation code for individual trial participants during trial conduct. The access to the code will only be given to authorised PSPV representatives for processing in the Pharmacovigilance database and not be shared further.

Proprietary confidential information © 2025 Boehringer Ingelheim International GmbH or one or more of its affiliated companies

4.1.6 Packaging, labelling, and re-supply

The IMP will be provided by Boehringer Ingelheim or a designated Contract Research Organisation (CRO). The IMP will be packaged and labelled in accordance with the principles of Good Manufacturing Practice (GMP). Re-supply to the sites will be managed via an IRT system, which will also monitor expiry dates of supplies available at the sites.

The label will be prepared according to EU Regulation No. 536/2014, Annex 6, Section D.8., omitting certain particulars with the following justifications:

- The investigator name was omitted from the label due to the IRT system.
- Should local regulations outside EU require these particulars they will be added to the country specific label text.

For details of packaging and the description of the label, refer to the ISF.

4.1.7 Storage conditions

IMP supplies will be kept in their original packaging to protect them from light, in a secure limited access storage area, and refrigerated according to the recommended storage conditions on the IMP label. A temperature log must be maintained for documentation.

If the storage conditions are found to be outside the specified range, the CRA (as provided in the list of contacts) must be contacted immediately.

The IMP will be provided in pre-filled syringes at specific visits (see SoA). An IFU with step-by-step instructions on how to use the pre-filled syringe will be made available to the investigator. A copy will be placed in the ISF. The trial participant will also receive a copy of the IFU when pre-filled syringes are dispensed to the participant for self-administration. Moreover, the IFU will contain important safety information and storage instructions.

4.1.8 Drug accountability

The investigator or designee will receive the IMP delivered by the sponsor or delegate when the following requirements are fulfilled:

- Approval of the clinical trial protocol by the IRB / ethics committee
- Availability of a signed and dated clinical trial contract between the sponsor or delegate and the investigational site
- Approval / notification of the regulatory authority, e.g. competent authority
- Availability of the *curriculum vitae* of the Principal Investigator
- Availability of a signed and dated clinical trial protocol
- Availability of the proof of a medical license for the Principal Investigator
- Availability of FDA Form 1572 (if applicable)

The IMP is not allowed to be used outside the context of this protocol. It must not be forwarded to other investigators or clinics, except if specifically authorised by the sponsor.

Trial participants should be instructed to bring all unused IMP and empty cartons with them to their next in-clinic visit (scheduled and unscheduled). Sharps container with used syringes

Proprietary confidential information © 2025 Boehringer Ingelheim International GmbH or one or more of its affiliated companies

should be returned to the site whenever a container replacement is needed and latest at EOT visit.

The investigator or designee must maintain records of the product's delivery to the trial site, the inventory at the site, the use by each trial participant, and the return to the sponsor or warehouse/drug distribution centre or alternative disposal of unused products using the IRT. If applicable, the sponsor or warehouse/drug distribution centre will maintain records of the disposal.

These records via IRT will include dates, quantities, batch/serial numbers, expiry ('use by') dates, and the unique code numbers assigned to the IMP and trial participants. The investigator or designee will maintain records that document adequately that the trial participants were provided the doses specified by the clinical trial protocol and reconcile all IMPs received from the sponsor. At the time of return to the sponsor or appointed CRO, the investigator or designee must verify that all unused or partially used IMP supplies have been returned by the trial participant and that no remaining supplies are in the investigator's possession.

4.2 OTHER TREATMENTS, EMERGENCY PROCEDURES, RESTRICTIONS

4.2.1 Other treatments and emergency procedures

4.2.1.1 Concomitant medications

During the trial, participants are expected to be treated according to the best standard of care for baseline medical conditions. Investigators will be asked to confirm in the eCRF during each visit if this condition is met.

As survodutide is expected to improve certain obesity-related complications (e.g. lipid levels, blood pressure, HbA_{1c}), the evaluation of existing treatment should be done during each in-clinic visit and eventual adjustment of treatment should be recorded in eCRF. All concomitant medications, including anti-hyperglycaemia medications (see Section 4.2.2.1), should be recorded on the appropriate pages of the eCRF. For the management of trial participants with GI symptoms, refer to Section 4.1.4.1.

Participants who develop diabetes during the trial may initiate medication for glucose control (see Section 4.2.2.1). They will be provided and trained to use a glucometer and provided an eDiary to record hypoglycaemic and hyperglycaemic episodes.

4.2.1.1.1 Home glucose monitoring

All participants with diabetes mellitus will be provided with a glucose monitoring device and supplies for use at home during the trial for self-measurement of blood glucose. Instruction on the use of the glucose monitoring device will be provided by the site staff. Trial participants may also use their own glucose monitoring device.

Weekly home measurements are recommended. Additionally, throughout the trial, blood glucose measurements should be performed at any time the participant is symptomatic, i.e. experiences signs/symptoms of hyper- or hypoglycaemia. More frequent testing may be done if deemed necessary by the investigator or required by local authorities.

Proprietary confidential information © 2025 Boehringer Ingelheim International GmbH or one or more of its affiliated companies

Blood glucose measurements should be taken fasted (at least 8 h) and prior to taking any anti-hyperglycaemic medication.

At the time of signing consent, all trial participants with T2DM will be educated about signs and symptoms of both hyper- and hypoglycaemia, how to treat both conditions, and how to collect information for each episode of hyper- or hypoglycaemia in the eDiary. Both conditions may be identified by spontaneous reporting of symptoms from trial participants, by plasma glucose samples collected during in-clinic visits, or by home glucose monitoring device.

4.2.1.1.2 Rescue medication for treatment of hyperglycaemia

Hyperglycaemia should be identified in case of persistent and unacceptable hyperglycaemia, if any of the FPG values (including fasting SMBG values) exceeds 15 mmol/L (270 mg/dL) and no intercurrent cause of the hyperglycaemia can be confirmed. In such cases, glycaemic rescue treatment should be initiated at the discretion of the investigator.

- If any of the FPG values (including fasting SMBG values) exceeds 15 mmol/L (270 mg/dL), a confirmatory FPG and HbA_{1c} value (at the central laboratory) should be obtained by calling the trial participant for an unscheduled visit.
- If the confirmatory measurement exceeds 15 mmol/L (270 mg/dL), the trial participant should be offered rescue medication, at the discretion of the investigator, according to the ADA/EASD guidelines [P22-05567] (excluding prohibited medications as stated in Section 4.2.2.1)

The following scenarios can be foreseen within the rescue treatment start and should be documented in the eCRF as rescue medications:

- Additional anti-hyperglycaemia medication used for 7 days or more or until treatment discontinuation
- Increase in the dose of background medication above the baseline dose for 7 days or more or until treatment discontinuation

If the trial participant does not have persistent and unacceptable hyperglycaemia >15 mmol/L (270 mg/dL) but the investigator deems necessary to adjust the anti-hyperglycaemia medications, these changes will not be considered as rescue therapy but as the therapy adjustment. Therapy adjustment has to be documented on the respective pages of the eCRF.

Trial participants that are started on rescue medication should continue to follow the protocol-specified visit schedule and stay on randomised treatment unless the investigator judges that it jeopardizes safety.

Rescue medication should be documented in medical records and reported in the eCRF. Rescue medication will not be supplied but reimbursed as long as the trial participant remains in the trial, if required according to local regulations.

4.2.1.1.3 Hypoglycaemic episodes

When a trial participant experiences a hypoglycaemic episode, the participant should call the site and record the general information in relation to the hypoglycaemia (date, time, blood

Proprietary confidential information © 2025 Boehringer Ingelheim International GmbH or one or more of its affiliated companies

glucose measurements, and any symptoms) in the eDiary. Blood glucose should always be measured and recorded when a hypoglycaemic episode is suspected.

Hypoglycaemic events will be recorded in the eCRF using the definitions as stated in Section 5.2.6.1.4. They should be treated and additional glucose monitoring should be implemented per investigator discretion and medical judgement.

All plasma glucose values <3.9 mmol/L (<70 mg/dL, with or without hypoglycaemic symptoms) should be reported as a hypoglycaemic episode.

4.2.1.2 Dietary and physical activity counselling

Participants will receive diet and physical activity counselling.

Participants will be given a paper Diary in which they will track their level of activity daily as well as their daily dietary compliance in order to assist and evaluate their lifestyle intervention (alternatively local apps can be used if already in use by participant prior to joining the study). The participant dietary compliance and the average daily level of physical activity will be evaluated and recorded in the eCRF at every visit by the site staff.

To assess dietary compliance, sites will be asked to record in the three-day food section of the eCRF everything that the individual participant has eaten and drunk for 3 days (preferably 2 weekdays and 1 weekend day, to be selected from the participant's daily diary entries that are typical for their current eating pattern). Additionally, the total daily energy intake and caloric breakdown (to be assessed by a dietician or equally qualified person) needs to be recorded.

All participants will receive dietary guidance materials. At the randomisation visit, participants will receive diet counselling by a dietician or equivalent qualified delegate according to local standards with a recommended energy deficit of approximately 500 kcal/day compared with the participant's estimated total energy expenditure [R20-2315].

The total energy expenditure is calculated at the randomisation visit by multiplying the estimated basal metabolic rate (Table 7) with a physical activity level value of 1.3 by the site staff (dietician or equally qualified person). The hypocaloric diet is to be continued after randomisation and throughout the planned treatment period. If a BMI of ≤22 kg/m^2 is reached, the recommended energy intake should be recalculated with no kcal deficit (maintenance diet) for the remainder of the trial.

Proprietary confidential information © 2025 Boehringer Ingelheim International GmbH or one or more of its affiliated companies

Table 7 Equations for estimating basal metabolic rate

Sex	Age [years]	Basal metabolic rate [kcal/day]
Men	18-30	15.057 × actual weight in kg + 692.2
	31-60	11.472 × actual weight in kg + 873.1
	>60	11.711 × actual weight in kg + 587.7
Women	18-30	14.818 × actual weight in kg + 486.6
	31-60	8.126 × actual weight in kg + 845.6
	>60	9.082 × actual weight in kg + 658.5

An increase in physical activity to at least 150 to 300 min a week of moderate intensity, combining aerobic and muscle strengthening exercises, will be encouraged and re-enforced.

4.2.1.3 Severe and/or systemic hypersensitivity including injection reaction and anaphylactic reaction

In case of severe and/or systemic hypersensitivity including anaphylactic reaction emerging during or after injection(s) of the IMP, the investigator should consider in accordance with the severity of the reaction and local standard of care to immediately stop further injections and treat with systemic antihistamines, i.v. steroids, and in case of a severe allergic reaction (e.g. anaphylactic reaction) with epinephrine.

In case of systemic hypersensitivity, based on the trial participant's clinical course and medical judgment, the injection(s) may be continued in case of mild or moderate systemic hypersensitivity.

In case of an anaphylactic reaction based on the criteria discussed in the statement paper by Sampson HA *et al*. [R11-4890] suspected to be caused by IMP, the investigator should permanently discontinue the treatment with IMP.

The following laboratory tests should be performed:

- A blood sample should be drawn for immunoglobulin E, PK, ADA, and NAb assessment as detailed in the Lab Manual (ISF).
- In addition, the evaluation of histamine, serum tryptase, and complement components (C3 and C4) should be included.

4.2.1.4 Elective surgery/procedures that require general anaesthesia or deep sedation

For trial participants who undergo elective surgery/procedures that require general anaesthesia or deep sedation, the following recommendations are made based on the American Society of Anaesthesiologists Consensus-Based Guidance on Preoperative Management of Patients (Adults and Children) on GLP-1R agonists [R23-2688]:

- Consider holding the IMP one week prior to elective surgery/procedure that requires general anaesthesia or deep sedation
- On the day of the procedure, if GI symptoms such as severe nausea/vomiting/ retching, abdominal bloating, or abdominal pain are present, consider delaying elective procedure

Proprietary confidential information © 2025 Boehringer Ingelheim International GmbH or one or more of its affiliated companies

- On the day of the procedure, if the participant has no GI symptoms, and the IMP has been held as advised, proceed as usual.

4.2.2 Restrictions

4.2.2.1 Restrictions regarding concomitant treatment

While enrolled in this trial, trial participants must not participate in another investigational drug or device trial or receive other investigational treatment(s).

GLP-1R agonists

GLP1R agonists including GLP1R agonist/insulin/GIP combinations are not allowed 3 months before screening visit and during the trial period from screening until end of follow-up. If such treatment is initiated during the trial, the participant should be instructed to discontinue the treatment with GLP1R agonists including GLP1R agonist/insulin/GIP combinations, and if the treatment is discontinued immediately, the participant should remain in the trial. If no discontinuation of such a drug is possible, the participant needs to stop IMP administration.

NASH medications

Vitamin E at the dose of less than 400 IU per day is allowed. If Vitamin E dose is ≥400 IU or more per day, the dose must be stable for 6 months prior to randomisation and during the entire duration of the trial. Initiation of more than 400 IU or change in dose of Vitamin E exceeding 400 UI per day is prohibited 6 months prior to randomization and during the entire duration of the trial.

Any off label, investigational (or subsequently approved) medications used for the purpose of treatment of NAFLD/NASH (including but not limited to GLP1R agonists) are prohibited 6 months prior to screening and for the trial duration up to EOS visit.

Treatment with drugs that may cause hepatic steatosis or steatohepatitis administered for at least 2 weeks within 12 months prior to screening is prohibited (e.g. valproic acid, tamoxifen, methotrexate, amiodarone, chronic treatment with oral corticosteroids >5 mg/day of prednisone equivalent [one short [<2 weeks] course of oral corticosteroids more than 3 months before screening is allowed], or oestrogens at doses greater than those used for contraception or hormone replacement).

Due to possible liver effects, the use of ursodeoxycholic acid during entire duration the trial, starting 3 months prior to screening, is not permitted.

Anti-obesity medications or supplements or participation in a weight reduction program
All anti-obesity medications including bupropion/naltrexone, orlistat, phentermine, and phentermine/topiramate, anti-obesity supplements or participating in an organised weight loss program (e.g. Weight Watchers® or Noom®), or any medication, including over-the-counter medication (in the opinion of the investigator) that could enable weight change are not allowed 3 months before screening and during the whole trial period (screening, treatment, and follow-up periods including trial participants who discontinue the IMP).

If such treatment is initiated during the trial, the participant should be instructed to discontinue the treatment, and if the treatment is discontinued, the participant should remain

Proprietary confidential information © 2025 Boehringer Ingelheim International GmbH or one or more of its affiliated companies

in the trial. If no discontinuation of such a drug is possible, the participant needs to stop IMP administration.

Body weight change inducing medication

Initiation of treatment with medications that may cause body weight change is not allowed 3 months before screening and during the trial (see examples in Table 8). Participants who were taking these medications for >3 months prior to screening with stable body weight are allowed to participate in the trial. If it is necessary to start such a treatment during the trial, alternative medications without potential to change body weight should be considered. In case there is no alternative possible, the sponsor should be contacted. If possible, the same approach should be applied to participants who discontinued the IMP and are in the follow-up period.

Table 8 Body weight change inducing medication[1]

**Medication class**	**Weight gain**	**Weight loss**
Anti-hypertensives	α-adrenergic blockers (e.g. doxazosin, prazosin, terazosin)<br>β-blockers (e.g. atenolol, metoprolol, nadolol, propranolol)	
Anti-depressants	TCA (e.g. amitriptyline, doxepin, imipramine, nortriptyline), MAOIs, SSRIs (e.g. paroxetine), mirtazapine, lithium	Bupropion, fluoxetine, triple reuptake inhibitors (tesofensine)
Anti-psychotics	FGA (e.g. thorazine, thioridazine, haldol, fluphenazine), SGA (e.g. clozapine, olanzapine, risperidone, asenapine)	
Anti-epileptics	Carbamazepine, gabapentin, pregabalin, valproic acid	Topiramate, zonisamide
Anti-histamines[2]	First generation antihistamines (e.g. diphenhydramine)	
Systemic steroids[2]	Glucocorticoids (e.g. dexamethasone, methylprednisolone, prednisolone, prednisone)	
Opioid antagonists		Naltrexone

[1]This is not an exhaustive list.
[2]Short use up to 14 days is allowed. If longer use is needed, the sponsor should be contacted.

Anti-hyperglycaemia medications

Only trial participants who develop T2DM during the trial may initiate anti-hyperglycaemia medications for glucose control, with the exception of GLP-1R agonists, GLP-1R agonist/ GIP combinations, amylin analogues, and DPP-4i (Table 9).

During the trial the following medication for the treatment of diabetes is allowed as per local guideline: metformin, SGLT2i, thiazolidinediones, sulfonylurea, insulin or acarbose (Table 9). Metformin should not be initiated during the trial for the treatment of other metabolic conditions (e.g. polycystic ovary syndrome, diabetes prevention). For participants on pioglitazone, the dose should be stable for 6 months before enrolment. For participants on stable sulfonylurea or insulin treatment, it is recommended to lower the dose when starting the IMP treatment to avoid hypoglycaemic events. All concomitant medications should be recorded on the appropriate pages of the eCRF.

Proprietary confidential information © 2025 Boehringer Ingelheim International GmbH or one or more of its affiliated companies

Table 9 Anti-hyperglycaemia medications

**Allowed medication**	**Prohibited medication**
Metformin	GLP-1R agonists
SGLT2i	GLP-1R agonist/GIP combinations
Thiazolidinediones[1]	Amylin analogues
Sulfonylureas[2]	DPP-4i
Insulin[3]	
Acarbose	

[1] For participants on pioglitazone use, the dose should be stable for 6 months before enrolment

[2] Allowed for participants on stable treatment (i.e. for ≥3 months prior to screening), but not allowed for treatment initiation during screening and the whole trial period

[3] HOMA-IR, HOMA-ß, and fasting plasma insulin collection and evaluation should not be performed in subjects on current insulin therapy.

Oral medications with a narrow therapeutic index
Due to the effect of survodutide on gastric emptying which may affect the absorption of orally administered drugs, increased clinical or laboratory monitoring for orally administered co-medications that have a narrow therapeutic index is recommended, and if not possible, drugs with NTIs are not permitted during the IMP treatment period until at least 28 days after last dose.

CYP3A sensitive medications with a narrow therapeutic index
As survodutide has the potential to down regulate CYP3A4 expression, which might affect safety and efficacy of CYP3A sensitive drug substances with a narrow therapeutic index, all CYP3A sensitive medications with NTIs (e.g. alfentanil, fentanyl, carbamazepine, cyclosporine, tacrolimus, sirolimus) are not permitted during the during the IMP treatment period until at least 28 days after last dose.

Oral contraceptives
Due to the effect of survodutide on gastric emptying which may affect the absorption of orally administered drugs and the effect of oral contraceptives may no longer be guaranteed, increased precautions are recommended. Women who use oral contraceptives at screening should be advised to change to non-oral contraceptives at least 7 days prior to first dose of IMP or to add additional barrier method of contraception during the entire treatment period with IMP and 28 days thereafter (see Section 4.2.2.3).

4.2.2.2 Restrictions on diet and lifestyle

It is recommended that participants abstain from alcohol consumption during the trial.

Please refer to Section 4.2.1.2. for dietary and physical activity counselling.

Proprietary confidential information © 2025 Boehringer Ingelheim International GmbH or one or more of its affiliated companies

4.2.2.3 Contraception requirements

Contraception of male participants is not needed.

WOCBP (for the definition please refer to Section 3.3.2) must use a medically approved method of birth control throughout the trial and for a period of at least 28 days after last IMP intake. A list of contraception methods meeting these criteria is provided in the trial participant information and Section 10.3.

4.3 TREATMENT COMPLIANCE

Trial participants will be provided with an eDiary to record all the injections of the IMP (at home or at the clinic during in-clinic visits).

Trial participants should be encouraged to be fully compliant with the medication dosing schedule. A weekly reminder for recording the date and time of IMP administration will be sent to the trial participants through a digital support platform to allow trial participants to follow closely their weekly dosing schedule. Trial participants who are not willing to use the digital platform should be closely followed up and monitored for compliance by the site.

Trial participants should be compliant on clinic visits within the protocol allowed time window and on the dosing schedule when they self-administer the IMP at home with pre-filled syringes. An IFU with instructions on how to use the pre-filled syringes including important safety information and storage instructions will be provided to the investigators and trial participants.

A compliance check of the IMP administration will be performed at each clinic visit to ensure the weekly injection is being administered correctly at both the trial site and by the trial participant at home. In case of missed doses, the site should instruct the trial participant as indicated in Section 4.1.4. The investigator/site staff should explain to the trial participant the importance of treatment compliance and discuss with the trial participant any tolerability issue.

Trial participants will be provided with a medication bag to store empty cartons of the IMP and a sharp bin to store all used syringes. Unused IMP should be kept in the refrigerator. Trial participants are requested to bring all unused IMP, empty cartons, and sharps container with used syringes with them to their next in-clinic visit (scheduled and unscheduled) for the compliance check. The sharps container with used syringes should be returned to the site. Any discrepancies should be discussed with the investigator or the designee.

Proprietary confidential information © 2025 Boehringer Ingelheim International GmbH or one or more of its affiliated companies

5. ASSESSMENTS

5.1 ASSESSMENT OF EFFICACY

5.1.1 MRI

MRI will be used to assess liver fat content using PDFF, liver stiffness using MRE, fibro-inflammation using cT1, and liver volume. To participate in the trial, sites must have an MRI scanner and MRE acoustic driver from one of the following manufacturers: GE, Philips, or Siemens, and have access to manufacturer-specific MRE sequences. Ideally, the MRI scanner should be equipped with a manufacturer-specific MRI-PDFF package, also known as multi-echo, multi-peak Dixon techniques. The cT1 analysis requires proprietary algorithms that must be configured on the site MRI device by the patent holder (Perspectum). The cT1 and PDFF will be calculated using the "LiverMultiScan®" package. All imaging sites will undergo a quality assessment process before the trial begins, which includes a review of their MRI and MRE hardware and software. Sites may collaborate with other sites that meet these requirements. Prior to MRE measurements, 4 h of fasting are required.

To calculate the liver fat volume, MR images will be collected from each participant during a single MRI session, utilising 3D image acquisition to determine the complete liver volume. Other MR image sequences may also be collected for exploratory analyses if required. The MR image acquisition process, including preparation time, is estimated to take no longer than 30 min. All MR images will be sent to an external vendor for centralised reading. In case of technical issues or inadequate quality of individual scans, MRI including MRE assessments may be repeated with approval from the sponsor.

5.1.2 Fibroscan®

Liver stiffness and steatosis will be assessed using the FibroScan® device (Echosens). Liver stiffness is a measurement of fibrosis and will be assessed using VCTE™. The liver steatosis will be assessed using CAP™. Both measurements are performed using a standard FibroScan® device using the participant specific ultrasound probes which are tailored to the morphology of the participant. Prior to FibroScan® measurements, 3 h of fasting are required.

In case the FibroScan® device supports the SmartExam computation method (1. SmartExam: E. Bardou-Jacquet et al, Validation of the Continuous Controlled Attenuation Parameter (CAPc) using the MRI-PDFF as reference. EASL 2022 poster #FRI-228), this option should be disabled (turned off) for all measurements and for all study participants.

Should a participant be measured with SmartExam turned on, all subsequent measurements should be made with SmartExam turned off.

In case of technical issues or inadequate quality of individual scans, Fibroscan assessments may be repeated with approval from the sponsor.

Note that participants who prematurely discontinue IMP due to pregnancy are not to undergo any imaging assessments for the purpose of the trial.

Proprietary confidential information © 2025 Boehringer Ingelheim International GmbH or one or more of its affiliated companies

5.1.3 Body measurements

For time points, refer to the SoA.

5.1.3.1 Weight measurement

It is important to minimise the variability in body weight measurement. The sponsor will provide medically graded weight scales to the sites which must be used throughout the course of the trial for all measurements. The equipment will be certified and calibrated according to manufacturer's specifications. Weight should be recorded in kilograms up to 1 decimal.

The body weight must be measured in fasting state (except the screening visit). If the trial participant is not fasted, the participant should be called in for a new visit within the visit window. In order to get comparable body weight values, shoes, coats/jackets, and any headgear should be taken off, and pockets should be emptied of heavy objects (i.e. keys, coins, etc.). Headgear worn for religious reasons are acceptable, but this should be consistently worn for all weight measurements in the trial. The trial participant should empty the bladder before weight is measured.

5.1.3.2 Height

Height should be measured without shoes in centimetres.

5.1.3.3 Body mass index

BMI will be calculated by the eCRF from the height and weight measured at the time points specified in the SoA.

5.1.3.4 Waist and hip circumference measurement

Waist circumference measurements should be made around the bare midriff, i.e. the abdominal circumference located midway between the lower rib margin and the iliac crest. Hip circumference measurements should be taken around the widest portion of the buttocks without thick clothes (e.g. coats, jackets). Measures must be obtained in standing position with a non-stretchable measuring tape and to the nearest centimetre or inch. The tape should touch the skin but not compress soft tissue, and twists in the tape should be avoided. The trial participant should be asked to breathe normally. The same measuring tape should be used throughout the trial. The measuring tape will be provided by the sponsor.

5.1.4 Blood pressure measurements

Vital signs will be evaluated at the time points specified in the SoA, prior to blood sampling.

Initially, blood pressure should be taken in both arms. If the pressures differ by more than 10 mmHg (as in the presence of a subclavian steal syndrome), the arm with the highest pressure (either systolic or diastolic) should be used for all subsequent measurements.

Blood pressure measurements should be performed on the same arm. On-site, the same method and device must be used throughout the trial for a participant. After participants have rested quietly in the seated position for 5 min, 3 blood pressure measurements will be taken

Proprietary confidential information © 2025 Boehringer Ingelheim International GmbH or one or more of its affiliated companies

2 min apart, and all 3 results will be entered in the CRF. The seated pulse rate will be taken during the 2 minute interval between the second and third blood pressure reading. Blood pressure measurements can be repeated at the discretion of the investigator, if the values are out of normal range. Caffeine, inhalation of nicotine, and physical activity should be avoided within 30 min prior to the blood pressure measurement.

[redacted]

5.1.6 Efficacy laboratory parameters

Some of the lab parameters will be analysed as part of efficacy criteria (see Sections 2.1.3, 2.2.2, 5.2.3, and 5.4.1).

Proprietary confidential information © 2025 Boehringer Ingelheim International GmbH or one or more of its affiliated companies

5.2 ASSESSMENT OF SAFETY

Safety is monitored during the trial conduct as per the below procedures. Investigators are encouraged to repeat ECG and/or additional imaging and laboratory testing if any clinically significant abnormal finding is observed as required.

5.2.1 Physical examination

A complete physical examination will be performed at the time points specified in the SoA, including at a minimum general appearance, neck (including thyroid gland), lungs, cardiovascular system, abdomen, extremities, and skin. The physical examination should be repeated if the baseline visit (Visit 2) is performed more than 2 weeks after the screening visit.

The results must be included in the source documents available at the site.

5.2.2 Vital signs

Vital signs will be evaluated at the time points specified in the SoA, prior to blood sampling. This includes SBP, DBP, and pulse rate (electronically or by palpation count for 1 minute) in a seated position after at least 5 min of rest. See also Section 5.1.4.

5.2.3 Safety laboratory parameters

Safety laboratory parameters to be assessed are listed in Table 10, and the sampling time points are shown in the SoA.

The total amount of blood collected during this study will not exceed 570 mL (39 tablespoons), including optional biobanking.

All analyses will be performed by a central laboratory, and the respective reference ranges will be provided in the ISF. Instructions regarding sample collection, sample handling/processing, and sample shipping are provided in the laboratory manual.

All in-clinic visits should be on fasting state (except for screening). Trial participants should be instructed to come in the fasting state after at least 8 h without food or liquid intake, except water. The IMP dose (if applicable) should be withheld in the morning of the clinic visit until blood sampling has been performed. If a trial participant has come to the in-clinic visit in non-fasting state, procedures that require fasting (blood sampling for safety, FPG, FPI, fasting C-peptide, lipids) should be performed on another day within the visit window.

The central laboratory will send reports and alerts to the investigator. It is the responsibility of the investigator to evaluate the laboratory reports. Clinically relevant abnormal findings as judged by the investigator will be reported as AEs (refer to Section 5.2.6).

In case the criteria for hepatic injury are fulfilled, a number of additional measures will be performed as per the DILI Checklist (Section 5.2.6.1.4). The amount of blood taken from the trial participant concerned will be increased due to this additional sampling. The investigator will use the exclusionary lab test results for the assessment of the participants' eligibility for the trial.The central laboratory will transfer the results of the analysis to the sponsor periodically.

Proprietary confidential information © 2025 Boehringer Ingelheim International GmbH or one or more of its affiliated companies

Table 10 Laboratory tests

Category	Test name
Safety laboratory test Haematology	Haematocrit<br>Haemoglobin<br>Reticulocyte count (reflex test if haemoglobin outside normal range)<br>Erythrocytes (including morphology)<br>Leukocytes<br>Thrombocytes<br>Neutrophils (segmented when the differential is abnormal)<br>Eosinophils<br>Basophils<br>Monocytes<br>Lymphocytes<br>INR
Safety laboratory test Clinical chemistry	Albumin<br>Alkaline phosphatase<br>Amylase<br>Pancreatic amylase (reflex test if amylase is elevated)<br>ALT<br>AST<br>Bicarbonate<br>Bilirubin total, fractionated if increased<br>Calcium<br>Calcitonin<br>Chloride<br>Creatine kinase (CK)<br>CK-MB (reflex test if CK is elevated)<br>INR (reflex test if DILI suspected)<br>High-sensitivity Troponin T at V2, V7, V11, V15 and ETD; at other visits reflex tests if CK is elevated<br>eGFR (CKD-EPIcr equation for adults)<br>Enzymatic creatinine<br>Gamma-glutamyl transferase<br>hsCRP<br>Lactate dehydrogenase<br>Lipase<br>Magnesium<br>Phosphate<br>Potassium<br>Protein total<br>Sodium<br>Urea (blood urea nitrogen)<br>Uric acid<br>TSH
Glucose metabolism	Fasting plasma glucose (FPG)<br>Fasting Insulin (FPI)<br>HbA_{1c}<br>C-peptide

Proprietary confidential information © 2025 Boehringer Ingelheim International GmbH or one or more of its affiliated companies

Table 10 (cont'd) Laboratory tests

Category	Test name
Lipids	Cholesterol<br>HDL cholesterol<br>LDL cholesterol<br>VLDL cholesterol<br>Triglycerides<br>Free fatty acids<br>ApoB
Menopausal testing	FSH: For women between age of 40 and 55 years with amenorrhea for 12 months. If the participant is on HRT, HRT must be withheld intermittently to confirm the menopausal status via FSH testing before enrolment. If pausing HRT for this purpose is not possible or not accepted by the participant, this participant will have to use non-oestrogen hormonal highly effective contraception methods compatible with HRT.<br>FSH testing is not required for women who are permanently sterile (for definition of being permanently sterile, refer to Section 3.3.2) or for women ≥55 years old with amenorrhea for at least 12 months without alternative medical cause other than being postmenopausal.
Pregnancy testing	Serum and urine human chorionic gonadotropin (hCG) tests will be performed at screening for women of childbearing potential.<br>Urine hCG test will be performed at all other in-clinic visits using tests provided by the central laboratory. If the urine test is positive, repeat with a serum test.
Urinalysis	Semiquantitative analyses: nitrite, protein, ketone, pH value, leukocyte esterase, erythrocytes<br>Quantitative analyses: urinary albumin, urinary creatinine, [redacted]<br>Urine sample should be collected void in the clinic from the second morning (preferably) and if not possible, from spot urine.
Severe and/or systemic hypersensitivity:	Tryptase<br>Complement C3<br>Complement C4
Infectious serology	Hepatitis B surface antigen (screening only)<br>Hepatitis C antibodies (screening only)<br>HCV RNA (screening only)[1]<br>HIV-1/2 combination (screening only)

[1]Will only be performed in case of Hepatitis C antibodies positivity or history of hepatitis C treatment.

5.2.4 Electrocardiogram

12-lead ECGs should be collected prior to the dosing at the time points specified in the SoA.

At screening, a triplicate ECG will be collected (i.e. three single ECGs recorded within 180 s). Centralised ECG services will be provided by an external vendor. ECGs should be collected according to the trial-specific recommendations, using the standardised equipment provided by the vendor. ECGs may be repeated for quality or safety reasons. Trial participants must be supine for approximately 5 to 10 min before ECG collection. Trial participants should remain supine, but awake, during the ECG collection process.

The investigator has the responsibility to complete an initial review as soon as the ECG recordings are obtained at the site visit. At any time during the trial, the investigator may decide to place a hold on further dosing of the trial participant if there is an indication of

Proprietary confidential information © 2025 Boehringer Ingelheim International GmbH or one or more of its affiliated companies

significant abnormalities in the ECG and may prefer to wait until the results from the central reading are available.

The digital ECG recordings will be transmitted to the vendor for central reading. The ECG recordings will be centrally evaluated and rated as normal, abnormal, or unable to evaluate, and the results will be reported to the site. The site investigator must review the report. If the ECG is rated as abnormal, the investigator will have to determine if the abnormal findings are clinically significant. Clinically significant abnormal findings based on central reading will be reported either as baseline condition (if identified prior to first IMP administration) or otherwise as AEs and will be followed up and/or treated as medically appropriate.

After the screening visit, the investigator must complete a review of the ECG results from central reading. Any pre-existing conditions should be recorded as baseline conditions. ECGs recorded at the randomisation visit should be evaluated by the investigator before the trial participant receives the first dose. If abnormalities are observed by the investigator in the ECG reading at the randomisation visit, it is recommended that the investigator waits until the results from central reading are available, and the randomisation visit should be rescheduled.

The site investigator will have the responsibility of following up with the trial participant if there are any clinically significant findings in the ECG report. It is recommended to repeat the 12lead ECG recording and/or refer to a cardiologist and/or perform additional cardiac tests (i.e. cardiac enzymes) if there is an indication of significant abnormalities or in case of doubts.

QTcF will be applied as the QT correction method.

If a clinically relevant increase (according to investigator judgement) in the QTcF interval or any other clinically significant quantitative or qualitative change from last ECG recording performed prior to the first IMP administration is identified after the trial participant is randomised, the investigator will assess the symptoms (e.g. palpitations, near syncope, and syncope) and decide if the participant will continue taking the IMP. In case automated QTcF on local ECG interval is informed as >500 ms, or a mean increase in QTcF interval more than 60 ms AND in mean QTcF interval above ULN (470 ms for women, 450 ms for men) compared with the last ECG recording performed prior to the first IMP administration obtained from central ECG reading, IMP should be temporarily interrupted and then, discontinued permanently, if central ECG reading confirms this finding (see also Section 3.3.4.1). Any new findings or deterioration of previous findings observed during the trial, assessed by investigator as clinically significant, will be recorded as AEs/SAEs and will be followed up and/or treated as medically appropriate.

All ECGs that are read in the central location will be stored in the vendor's database and transmitted to the sponsor periodically. If there is any clinically significant finding in an ECG performed locally due to presence of acute symptoms, this ECG will be transmitted to the vendor for central reading.

Proprietary confidential information © 2025 Boehringer Ingelheim International GmbH or one or more of its affiliated companies

5.2.5 Other safety parameters

5.2.5.1 Mental health questionnaires

The mental health status of the trial participants will be assessed by the C-SSRS and PHQ9 questionnaires. The questionnaires will be administered to all trial participants (electronically) in local language (linguistically validated version). Details to the (electronic) use can be found in the ISF.

The C-SSRS is a semi-structured, investigator-rated interview, developed by clinical experts in cooperation with the FDA and requested by regulatory agencies, assessing both suicidal behaviour and suicidal ideation. It does not give a global score but provides some categorical and some severity information specifically for behaviour and ideation.

The C-SSRS interview may be administered by any type of physician, psychologist, clinical social worker, mental health counsellor, nurse, or research coordinator with C-SSRS training. It has a typical duration of 5 min and causes only a low burden on trial participants. At a minimum, the interview consists of 2 screening questions related to suicidal ideation and 4 related to suicidal behaviour and may be expanded to up to 17 items in case of positive responses.

The C-SSRS has been widely used in large multinational clinical trials. The C-SSRS will be administered at the screening visit (using the "baseline/screening" version) with the aim to exclude trial participants with a history of suicidal ideation and behaviour. After the baseline visit, the assessment "since last visit" will be performed at clinic and remote visits (if allowed per local regulations) as specified in the SoA.

The investigator will review positive and negative reports for plausibility and clinical relevance. Doubtful reports may be repeated, or reports may be validated by a consulting psychiatrist. If there is an answer "yes" to either Question 4 (Active Suicidal Ideation with Some Intent to Act, Without Specific Plan) on the "Suicidal Ideation" portion of the C-SSRS, or an answer "yes" to Question 5 (Active Suicidal Ideation with Specific Plan and Intent) on the "Suicidal Ideation" portion of the C-SSRS, or an answer "yes" to any of the suicide-related behaviours (Actual attempt, Interrupted attempt, Aborted attempt, Preparatory act or behaviour) on the "Suicidal Behaviour" portion of the C-SSRS, the investigator has to immediately interview the trial participant during the clinic visit and recommended the participant to consult with an MHP. Treatment with the IMP should be stopped, and the trial participant should remain in the trial. Additionally, all C-SSRS reports of suicidal ideation type 4 or 5 and all reports of suicidal behaviour must be reported as separate SAEs by the investigator.

For 'Self-injurious behaviour, no suicidal intent' (Type 11), standard AE/SAE reporting rules are to be applied. For each negative report (suicidal ideation Types 1, 2, or 3) after start of the trial, the investigator will decide based on clinical judgment whether it represents an AE as defined in this protocol, and if it is considered an AE then it must be reported accordingly.

The PHQ9 is the 9item depression module of the patient health questionnaire, which is a self-administered diagnostic tool used for assessment of mental disorders. It takes approximately 5 to 10 min to complete the questionnaire and it should be completed together with the other PROs. If the PHQ9 score is >15, the trial participants should be referred to an MHP to assist in deciding whether the participant may continue in the trial or should be discontinued. If the

Proprietary confidential information © 2025 Boehringer Ingelheim International GmbH or one or more of its affiliated companies

trial participant refuses to be referred to an MHP, the investigator will decide if the participant can remain in the trial.

The C-SSRS and the PHQ9 will be administered to all trial participants electronically where the digital approach is approved. At sites where these cannot be administered electronically, a paper back up will be available.

5.2.5.2 Eye examination for participants with T2DM

Uncontrolled and potentially unstable diabetic retinopathy or maculopathy is defined as an exclusion criterion (Section 3.3, exclusion criterion 13) and a trial participant presenting these conditions during screening is not eligible to participate in the trial. Testing is required via one of these options prior to participant randomisation within the screening period or if such an examination has been performed within the past 3 months prior to screening, the results can be used for eligibility evaluation:

- The pharmacologically pupil-dilated fundus eye examination performed by an ophthalmologist or an equally qualified healthcare provider (e.g. optometrist) OR
- Other ophthalmologist examination of diabetic retinopathy (as recommended by country T2DM management guidelines).

During the trial conduct, the eye examination needs to be performed at time points specified in the SoA or whenever the trial participant has experienced a worsening of vision.

Respective findings will be reported as AEs (refer to Section 5.2.6).

5.2.6 Assessment of adverse events

Data and information necessary for the thorough assessment of AEs, SAEs, and AESIs will be reported to the sponsor via eCRF. This may include specific data and information not prospectively specified in this protocol.

5.2.6.1 Definitions of AEs

5.2.6.1.1 Adverse event

An AE is defined as any untoward medical occurrence in a trial participant or clinical investigation subject administered a medicinal product and which does not necessarily have to have a causal relationship with this treatment.

An AE can therefore be any unfavourable and unintended sign (including an abnormal laboratory finding), symptom, or disease temporally associated with the use of a medicinal product, whether considered related or not.

Proprietary confidential information © 2025 Boehringer Ingelheim International GmbH or one or more of its affiliated companies

The following should also be recorded on the appropriate eCRF(s):

- Worsening of the underlying disease or of other pre-existing conditions
- Changes in vital signs, ECG, physical examination and laboratory test results, if they are judged clinically relevant by the investigator

If such abnormalities already exist prior to first IMP administration, they will be considered as baseline conditions and should be collected in the eCRF only.

5.2.6.1.2 Serious adverse event

An SAE is defined as any AE that fulfils at least one of the following criteria:

- Results in death
- Is life-threatening, which refers to an event in which the participant was at risk of death at the time of the event; it does not refer to an event that hypothetically might have caused death if more severe
- Requires inpatient hospitalisation or prolongation of existing hospitalisation
- Results in persistent or significant disability or incapacity
- Is a congenital anomaly / birth defect
- Is deemed serious for any other reason if it is an important medical event when based on appropriate medical judgement which may jeopardise the participant and may require medical or surgical intervention to prevent one of the other outcomes listed in the above definitions. Examples of such events are intensive treatment in an emergency room or at home for allergic bronchospasm, blood dyscrasias or convulsions that do not result in hospitalisation or development of dependency or abuse

5.2.6.1.3 AEs considered "Always Serious"

In accordance with the European Medicines Agency initiative on Important Medical Events, Boehringer Ingelheim has set up a list of AEs, which by their nature, can always be considered to be "serious" even though they may not have met the criteria of an SAE as defined in Section 5.2.6.1.2. The latest list of "Always Serious AEs" can be found in the eDC system. A copy of the latest list of "Always Serious AEs" will be provided upon request. These events should always be reported as SAEs as described in Section 5.2.6.2.

Cancers of new histology and exacerbations of existing cancer must be classified as a serious event regardless of the time since discontinuation of the drug and must be reported as described in Section 5.2.6.2, subsections "AE Collection" and "AE reporting to sponsor and timelines".

All C-SSRS reports of suicidal ideation Type 4 or 5 and all reports of suicidal behaviour must be reported as separate SAEs by the investigator.

5.2.6.1.4 Adverse events of special interest

The term "adverse event of special interest" (AESI) relates to any specific AE that has been identified at the project level as being of particular concern for prospective safety monitoring

Proprietary confidential information © 2025 Boehringer Ingelheim International GmbH or one or more of its affiliated companies

and safety assessment within this trial, e.g. the potential for AEs based on knowledge from other compounds in the same class. AESIs need to be reported to the sponsor's Pharmacovigilance Department within the same timeframe that applies to SAEs; see Section 5.2.6.2.2.

The following are considered as AESIs:

Potential severe drug-induced liver injury

A potential DILI that requires follow-up is defined by any of the combinations of treatment emergent ALT assessed together with the corresponding treatment-emergent bilirubin, and new onset of liver-related symptoms, as described in each row of Table 11. Respective actions, including repetition of liver enzyme tests or interruption of IMP as described in the specific row/scenario of Table 11, should be performed.

These laboratory findings with or without new onset of liver-related symptoms (as described in each row of Table 11) constitute a hepatic injury alert, and trial participants showing any of these lab abnormalities need to be followed up according to the DILI checklist provided in the EDC system and the ISF.

In case of clinical signs or symptoms of hepatic injury (icterus, unexplained encephalopathy, unexplained coagulopathy, right upper quadrant abdominal pain, etc.) without lab results (ALT, AST, total bilirubin) available, the investigator should make sure these parameters are analysed, if necessary, in an unscheduled blood test. Should the results meet the criteria of hepatic injury alert, the procedures described in the DILI checklist should be followed.

Trial participants should be followed up until resolution of symptoms or signs in the above stated situations [P09-12413]. Treatment with the IMP may be restarted only if another aetiology is clearly identified and the liver values have returned to baseline [R20-1981]. If DILI is confirmed, after resolution or stabilisation, the trial participant should complete the procedures for the EOT and FU/EOS visits as outlined in the SoA.

Following completion of the DILI checklist, if the IMP cannot be excluded as a possible cause of DILI event, then discontinuation should be made permanent without rechallenge. If an alternate causality is confirmed by the DILI checklist evaluation, then IMP may be re-started, if warranted.

Proprietary confidential information © 2025 Boehringer Ingelheim International GmbH or one or more of its affiliated companies

Table 11 Algorithm for monitoring and management of possible hepatocellular DILI in trial participants with normal or elevated baseline ALT[1]

**Treatment emergent ALT**	**Treatment emergent total bilirubin**	**New onset of liver related symptoms**	**Action**[2] Initiate DILI checklist evaluation for any of the 5 scenarios mentioned below.
Normal/near normal baseline: ALT ≥5x ULN<br>Elevated baseline[1]: ALT ≥3x baseline or ≥300 U/L (whichever occurred first)	Normal<br><br>Participants with Gilbert's syndrome: no change in total bilirubin	None	Repeat ALT, AST, alkaline phosphatase, and total bilirubin in 2-3 days. Interrupt IMP if ALT remains elevated on repeat testing. Initiate evaluation for other aetiologies of abnormal liver tests.
Normal/near normal baseline: ALT ≥3x ULN<br>Elevated baseline[1]: ALT ≥2x baseline or ≥300 U/L (whichever occurred first)	Normal<br><br>Participants with Gilbert's syndrome: no change in total bilirubin	Severe fatigue, nausea, vomiting, right upper quadrant pain, rash, eosinophilia (>5%)	Repeat ALT, AST, alkaline phosphatase, and total bilirubin in 2-3 days. Interrupt IMP if ALT remains elevated on repeat testing. Initiate evaluation for other aetiologies of abnormal liver tests.
Normal/near normal baseline: ALT ≥8x ULN<br>Elevated baseline[1]: ALT ≥5x baseline or ≥500 U/L (whichever occurred first)	Normal<br><br>Participants with Gilbert's syndrome: no change in total bilirubin	None	Interrupt IMP. Initiate close monitoring and workup for competing aetiologies. IMP can be restarted only if another aetiology is identified, and liver enzymes return to baseline.
Normal/near normal baseline: ALT[1] or AST[3] ≥3x ULN<br>Elevated baseline[1]: ALT ≥2x baseline or ≥300 U/L (whichever occurred first)	Total bilirubin ≥2x ULN<br><br>Participants with Gilbert's syndrome: doubling of direct bilirubin or increased INR to >1.5	None	Interrupt IMP. Initiate close monitoring and workup for competing aetiologies. IMP can be restarted only if another aetiology is identified, and liver enzymes return to baseline.
Normal/near normal baseline: ALT ≥5x ULN<br>Elevated baseline[1]: ALT ≥3x baseline or ≥300 U/L (whichever occurred first)	Normal or elevated	Severe fatigue, nausea, vomiting, right upper quadrant pain, rash, eosinophilia (>5%)	Interrupt IMP. Initiate close monitoring and workup for competing aetiologies. IMP can be restarted only if another aetiology is identified, and liver enzymes return to baseline.

[1]Elevated baseline is defined as ALT ≥1.5x ULN.

[2]The actions of close observation, monitoring, and IMP interruption often overlap. Occasionally, workup is initiated after IMP interruption.

[3] Only applicable for participants with baseline AST ≤ ULN.

Source data: [R20-1981]

Proprietary confidential information © 2025 Boehringer Ingelheim International GmbH or one or more of its affiliated companies

Thyroid malignancies and C-cell hyperplasia

Individuals with personal or family history of MTC and/or MEN-2 will be excluded from the study. Trial participants who are diagnosed with MTC during the study will have the IMP stopped.

The assessment of thyroid safety during the trial will include reporting of any case of thyroid malignancy (including MTC and papillary carcinoma) and measurements of calcitonin.

Trial participants with calcitonin levels ≥100 pg/mL or 29.26 pmol/L will be excluded from the trial. If an increased calcitonin value (≥100 pg/mL or 29.26 pmol/L) is observed during the trial, IMP should be discontinued, and follow-up by an endocrinologist should be done.

This data will be captured in specific eCRFs. All cases of thyroid cancer will be adjudicated.

Systemic and serious cutaneous hypersensitivity or anaphylactic reactions

All systemic and serious cutaneous hypersensitivity or anaphylactic reactions will be reported as AESIs. Further details are described in Section 4.2.1.3.

Acute gallbladder disease

All events of treatment emergent biliary colic, cholecystitis, or other suspected events related to gallbladder disease should be evaluated and additional diagnostic tests performed, as needed. In cases of elevated liver markers, hepatic monitoring should be initiated as outlined in Table 11. Additional data should be recorded on the specific eCRF page.

Severe hypoglycaemia requiring assistance (Level 3)

At the time of signing consent, all trial participants with T2DM will be educated about signs and symptoms of hypoglycaemia, how to treat hypoglycaemia, and how to collect information for each episode of hypoglycaemia. Hypoglycaemia may be identified by spontaneous reporting of symptoms from trial participants, by plasma glucose samples collected during in-clinic visits, or by home glucose monitoring device. The trial participants will be informed about the need to notify the investigator about all cases when they experience a hypoglycaemic episode. Upon onset of a hypoglycaemic episode, the trial participant is recommended to measure and record blood glucose every 15 min until the value is ≥3.9 mmol/L (≥70 mg/dL) and/or symptoms have been resolved in accordance with current guidelines. When a trial participant experiences a hypoglycaemic episode, the participant should record the general information in relation to the hypoglycaemia (date, time, blood glucose measurements, and any symptoms in the eDiary). Blood glucose should always be measured and recorded when a hypoglycaemic episode is suspected. Hypoglycaemic events will be recorded in the eCRF using the definitions in Table 12.

Table 12 Classification of hypoglycaemia

**Level**	**Glycaemic criteria/description**
Level 1	Glucose <70 mg/dL (<3.9 mmol/L) and ≥54 mg/dL (≥3.0 mmol/L)
Level 2	Glucose <54 mg/dL (3.0 mmol/L)
Level 3	A severe event characterised by altered mental and/or physical status requiring assistance for treatment of hypoglycaemia

Source data: [R22-4277]

Proprietary confidential information © 2025 Boehringer Ingelheim International GmbH or one or more of its affiliated companies

Hypoglycaemic events should be treated, and additional glucose monitoring should be implemented per investigator discretion and medical judgement. All hypoglycaemic episodes will be recorded in a specific eCRF page. All symptomatic hypoglycaemic events should be reported as an AE. All Level 3 hypoglycaemic events (severe events characterised by altered mental and/or physical status requiring assistance) should be reported as an AESI.

If the hypoglycaemic episode fulfils the criteria for an SAE, then reporting should be done as described in Section 5.2.6.2.2. One AE entry can cover several hypoglycaemic episodes, if the trial participant has not recovered between the episodes. All blood glucose values <3.9 mmol/L (<70 mg/dL, with or without hypoglycaemic symptoms) should be reported as a hypoglycaemic episode.

5.2.6.1.5 Intensity (severity) of AEs

The intensity (severity) of adverse events will be classified and recorded in the eCRF according to the Common Terminology Criteria for Adverse Events (CTCAE) Version 5.0 released on 27 Nov 2017 [R18-1357].

5.2.6.1.6 Causal relationship of AEs

Medical judgement should be used to determine the relationship between the AE and the Boehringer Ingelheim IMP, considering all relevant factors, including pattern of reaction, temporal relationship, de-challenge or re-challenge, confounding factors such as concomitant medication, concomitant diseases, and relevant history.

Arguments that may suggest a reasonable possibility of a causal relationship could be:

- The event is consistent with the known pharmacology of the IMP
- The event is known to be caused by or attributed to the drug class
- A plausible time to onset of the event relative to the time of IMP exposure
- Evidence that the event is reproducible when the IMP is reintroduced
- No medically sound alternative aetiologies that could explain the event (e.g. pre-existing or concomitant diseases, or comedications)
- The event is typically drug-related and infrequent in the general population not exposed to drugs (e.g. Stevens-Johnson syndrome)
- An indication of dose-response (i.e. greater effect size if the dose is increased, smaller effect size if dose is reduced)

Proprietary confidential information © 2025 Boehringer Ingelheim International GmbH or one or more of its affiliated companies

Arguments that may suggest no reasonable possibility of a causal relationship could be:

- No plausible time to onset of the event relative to the time of IMP exposure is evident (e.g. pretreatment cases, diagnosis of cancer or chronic disease within days / weeks of IMP administration; an allergic reaction weeks after discontinuation of the IMP concerned)
- Continuation of the event despite the withdrawal of the IMP, considering the pharmacological properties of the compound (e.g. after 5 half-lives).
  Of note, this criterion may not be applicable to events whose time course is prolonged despite removing the original trigger
- There is an alternative explanation, e.g. situations where other drugs or underlying diseases appear to provide a more likely explanation for the observed event than the IMP concerned
- Disappearance of the event even though trial IMP treatment continues or remains unchanged

5.2.6.1.7 Adverse events requiring additional data collection

The adverse events listed below are considered critical for the assessment of the safety profile of survodutide and will be recorded in a separate eCRF page. Some of them will be centrally adjudicated in a blinded fashion by an external clinical event committee, independent from the sponsor.

Events requiring adjudication:

- Major adverse CV events (5P-MACE), including
  - non-fatal MI
  - non-fatal stroke
  - ischaemia related coronary revascularisation
  - HF event (including hospitalisation for HF, emergency room visit, urgent care visit, or urgent outpatient HF visit)
  - CV death
- Non-CV death
- Acute pancreatitis
- Thyroid malignancies and C-cell hyperplasia
- Pancreatic cancer
- DILI

<u>Acute pancreatitis</u>

Acute pancreatitis is an acute inflammatory process of the pancreas that may also involve peripancreatic tissues and/or remote organ systems.

If acute pancreatitis is suspected, IMP should be stopped and appropriate laboratory tests (including levels of serum pancreatic amylase and lipase) should be obtained via the central laboratory (or locally, if needed). If there is evidence of increase in pancreatic amylase or

Proprietary confidential information © 2025 Boehringer Ingelheim International GmbH or one or more of its affiliated companies

lipase (3x ULN) and suspicion of acute pancreatitis, an abdominal CT scan (or MRI) with contrast should be performed to confirm the diagnosis. If contrast for both CT scan and MRI is contraindicated, a CT scan or MRI should be performed without contrast. If imaging does not support the diagnosis of acute pancreatitis, the IMP can be restarted as per investigator's judgment. If abdominal imaging supports the diagnosis of acute pancreatitis, IMP should be discontinued, and the participant should be requested to continue in the study (see also Section 3.3.4.1). A review of the participant's concomitant medications should be conducted to assess any potential causal relationship with pancreatitis.

If typical signs and/or symptoms of pancreatitis are present and confirmed by laboratory values (lipase or amylase [total and/or pancreatic]) and imaging studies, the event must be reported as an SAE. For a potential case that does not meet all of these criteria, it is up to the investigator to determine the seriousness of the case (AE or SAE) and the relatedness of the event to the IMP.

As a part of safety monitoring each trial participant will have measurements of amylase and lipase (assessed at the central laboratory) as shown in SoA to assess the effects of the investigational doses of survodutide on pancreatic enzyme levels. Relevant data from participants with acute or chronic pancreatitis will be entered into a specifically designed eCRF page.

All cases of pancreatitis will be adjudicated.

Pancreatic cancer

Based on data from experimental studies in animal models, it has been speculated that the chronic over-stimulation of GLP1R in exocrine pancreatic cells could induce pancreatitis, ultimately leading to an increased risk of pancreatic cancer. Cases of pancreatic cancer will be adjudicated, and details entered into a specifically designed eCRF page.

Events requiring additional data collection (these events will not be adjudicated):

Local tolerability and injection site reactions

Local tolerability at the administration site of survodutide will be assessed by the investigator during the IMP administration visits and at the time of AE assessment by questioning retrospectively since the last visit. Trial participants will answer the site reaction question in the eDiary after each home injection. The eDiary will be reviewed during the in-clinic visit. Any observed local tolerability reaction, e.g. "swelling", "induration", "heat", or "redness" should be collected on the specific eCRF page, and reported as AE if clinically significant.

Acute severe renal events

Acute severe renal event is defined as any stage 3 acute kidney injury (based on KDIGO definitions, see below), any acute kidney injury requiring hospital admission for treatment, or any stage AKI with secondary complications (eg. severe hyperkalaemia [serum potassium ≥6.5 or >6.0 with relevant ECG changes], pulmonary oedema, uraemic pericarditis, metabolic acidosis).

Renal function should be closely monitored in participants with GFR <60 mL/min/1.73 m^2 who develop severe vomiting and diarrhoea and/or signs and symptoms of dehydration.

Proprietary confidential information © 2025 Boehringer Ingelheim International GmbH or one or more of its affiliated companies

Renal safety will be assessed based on repeated renal functional assessment as well as assessment of AEs suggestive of acute or worsening of chronic renal failure. Survodutide has not shown to be nephrotoxic, however, consistent with other GLP1R agonists, GI AEs have been reported with survodutide, including nausea, diarrhoea, and vomiting. The events may lead to dehydration, which could cause a deterioration in renal function, including acute renal failure. Participants should be advised to notify investigators in case of severe nausea, frequent vomiting, diarrhoea, poor oral intake, or symptoms of dehydration. Adequate fluid substitution (oral or intravenous) must be initiated. Modification in dose of concomitant medications, in particular diuretics and anti-hypertensives, should be initiated to prevent dehydration or exacerbation of dehydration as medically indicated.

The diagnosis of acute kidney injury (AKI), based on Kidney Disease Improving Global Outcomes (KDIGO) recommendations, is shown below.

AKI grades will be stratified based on serum creatinine values compared with the last known measurement in patients:

- AKI stage 1: increase in creatinine ≥0.3 mg/dL (26.5 µmol/L) or 1.5 to 1.9 times the last known measurement

- AKI stage 2: serum creatinine 2.0-2.9 times the last known measurement

- AKI stage 3: Serum creatinine 3 times the last known measurement OR increase in creatinine ≥4.0 mg/dL (353.6 µmol/L) OR initiation of renal replacement therapy

Clinically significant increases in creatinine, as judged by the investigator, should be reported as an AE/SAE.

Additional details in the specific eCRF page should be included for reported AE/SAE of AKI.

Persistent and unacceptable hyperglycaemia

Hyperglycaemia can be identified in case of persistent and unacceptable hyperglycaemia, if any of the FPG values (including fasting SMBG values) exceeds 15 mmol/L (270 mg/dL) and no intercurrent cause of the hyperglycaemia can be confirmed (refer to Sections 4.2.1.1.1 and 4.2.1.1.2). Persistent and unacceptable episodes of hyperglycaemia should be recorded as AEs and in the specific eCRF page.

Worsening of diabetic retinopathy or maculopathy

Fast improvements in glucose levels may lead to a temporary worsening of diabetic eye disease. Trial participants with uncontrolled and potentially unstable diabetic retinopathy or maculopathy within the past 3 months before screening or in the period between screening and randomisation are excluded. All eye examinations including worsening of diabetic retinopathy or maculopathy should be recorded in the specific eCRF page (refer to Section 5.2.5.2).

Proprietary confidential information © 2025 Boehringer Ingelheim International GmbH or one or more of its affiliated companies

Hypotension, orthostatic hypotension, and syncopal events

Clinically significant hypotension, orthostatic hypotension, and syncopal events should be recorded in the specific eCRF page.

Arrhythmias and cardiac conduction disorders

Treatment-emergent clinically significant cardiac conduction disorders will be recorded as an AE. Events that meet criteria for serious conditions must be reported as SAEs. All clinically significant arrhythmias and cardiac conduction disorders should be recorded in the specific eCRF page.

Major depressive disorder, requiring hospitalisation

Participants will be monitored for depression through AE collection and by using the PHQ-9. For further details, refer to Section 5.2.5.1.

Suicidal ideation and behaviour

Participants will be monitored for suicidal ideation and behaviour through AE collection and by using the C-SSRS. For further details, refer to Section 5.2.5.1.

5.2.6.2 Adverse event collection and reporting

5.2.6.2.1 AE Collection

The investigator shall maintain and keep detailed records of all AEs in the participant files.

The following must be collected and documented:

All AEs (serious and non-serious) and all AESIs from signing the informed consent onwards until the individual trial participant's EOS (usually the FU/EOS visit), see Section 5.2.6.2.2.

After the individual trial participant's EOS visit, the investigator does not need to actively monitor the trial participant for new AEs. However, the following events should be reported:

- Any occurrence of cancer of new histology
- IMP related SAEs
- IMP related AESIs of which the investigator may become aware of by any means of communication, e.g. phone call.

Vital status data collection

Trial participants who discontinue IMP prematurely, and agree to be contacted further after the ETD visit, should be followed up as described in Section 3.3.4.1. The above-described AE reporting requirements apply as far as this is possible. If a trial participant only agrees to be contacted for vital status data collection, the investigator must report until the individual participant's EOS visit any occurrence of cancer, all deaths/fatal AEs regardless of relationship, IMP related SAEs, and IMP related AESIs the investigator becomes aware of.

Proprietary confidential information © 2025 Boehringer Ingelheim International GmbH or one or more of its affiliated companies

5.2.6.2.2 AE reporting to the sponsor and timelines

The investigator must report SAEs, AESIs, and non-serious AEs that are relevant for the reported SAE or AESI immediately (within 24 h of becoming aware of the event). SAE will be reported using paper SAE forms. The country-specific process will be described in the ISF. On specific occasions, the investigator could inform the sponsor upfront via telephone in addition.

With receipt of any further information on these events, follow-up reports must be provided. For follow-up information, the same rules and timelines apply as for initial information. All AEs / SAEs, including those persisting after an individual participant's end of trial must be followed up until they have resolved, have been assessed as "chronic" or "stable", or no further information can be obtained.

5.2.6.2.3 Pregnancy

In rare cases, pregnancy might occur in a clinical trial. Once a trial participant has been enrolled in the clinical trial and has taken trial IMP, the investigator must report any IMP exposure during pregnancy in a trial participant immediately (within 24 h) by means of Part A of the Pregnancy Monitoring Form to the sponsor's unique entry point.

It is not required to report the pregnancy of a partner of a male trial participant.

The outcome of the pregnancy associated with the IMP exposure during pregnancy must be followed up and reported to the sponsor's unique entry point on the Pregnancy Monitoring Form for Studies (Part B). If required by local Ethics Committees or regulatory authorities, this will only occur after a separate pregnancy informed consent has been given.

The ISF will contain the Pregnancy Monitoring Form for Studies (Parts A and B).

As pregnancy itself is not to be reported as an AE, in the absence of an accompanying SAE and/or AESI, only the Pregnancy Monitoring Form for Studies is to be completed. However, an SAE and / or AESI associated with the pregnancy must be reported as described in Section 5.2.6.2.2.

5.2.6.2.4 Other safety topics

Severe and serious gastrointestinal events

Survodutide may cause severe GI AEs, such as nausea, vomiting, and diarrhoea. Information about severe and serious GI AEs will be collected in the AE form and information regarding antiemetic/antidiarrheal medication use will be collected in the Concomitant Medication form. For detailed information concerning the management of GI AEs, please refer to Section 4.1.4.1.

Calcitonin monitoring

Trial participants with calcitonin levels ≥100 pg/mL or 29.26 pmol/L during screening will be excluded from the trial. If an increased calcitonin value (≥100 pg/mL or 29.26 pmol/L) is observed during the course of the trial, IMP should be discontinued, and follow-up by an endocrinologist should be done.

Proprietary confidential information © 2025 Boehringer Ingelheim International GmbH or one or more of its affiliated companies

5.3 DRUG CONCENTRATION MEASUREMENTS AND PHARMACOKINETICS

5.3.1 Assessment of pharmacokinetics

Date and clock time of drug administration as well as actual date and clock time of PK sampling will be captured in the database.

For PK analysis, trough plasma concentration of survodutide will be used for the evaluation of the influence of intrinsic and extrinsic factors on survodutide.

A population PK analysis will be undertaken using appropriate recognised software to analyse PK sampling data. The trough plasma concentrations will be used to refine an already existing population PK model. Exposure predictions based on the population PK model may subsequently be used for characterizing the exposure-response relationships for selected efficacy and/or safety endpoints.

5.3.2 Methods of sample collection

Detailed instructions on sampling, preparation, processing, shipment, and storage are provided in the laboratory manual.

For quantification of survodutide plasma concentrations, blood samples will be taken at the time points described in the SoA. Blood will be withdrawn by means of either an indwelling venous catheter or by venepuncture with a metal needle.

At a minimum, the sample tube labels should list the following information: BI trial number, trial participant number, and visit. Further information such as matrix and analyte may also be provided.

Plasma samples will be discarded at the latest 6 months after the final clinical trial report has been signed.

All lab kit supplies will be provided by the central lab vendor designated by the sponsor.

[redacted]

5.3.4 Pharmacokinetic - pharmacodynamic relationship

An analysis of the relationship between PK and explorative biomarker parameters may be investigated in an exploratory manner. Results will be reported in a separate report.

Proprietary confidential information © 2025 Boehringer Ingelheim International GmbH or one or more of its affiliated companies

[redacted]

Proprietary confidential information © 2025 Boehringer Ingelheim International GmbH or one or more of its affiliated companies

[redacted]

5.5 BIOBANKING

For sampling timepoints, see the SoA. Biobanking will be performed only in countries where allowed by local laws and regulations. Participation in biobanking is voluntary and not a prerequisite for participation in the trial. Biobanking will occur only after a separate biobanking informed consent has been given in accordance with local ethical and regulatory requirements.

To be able to address future scientific questions, trial participants will be asked to voluntarily donate biospecimens for banking. If the trial participant agrees, banked samples may be used for future biomarker research and drug development projects, e.g. to identify patients that are more likely to benefit from a treatment or experience an AE, or to gain a mechanistic or genetic understanding of drug effects and thereby better match patients with therapies. Samples will be stored for testing for a period consistent with local regulations.

5.5.1 Methods and timing of sample collection

Detailed instructions on sampling, preparation, processing, shipment, and storage are provided in the laboratory manual. Samples will be taken prior to the IMP administration. For sampling time points, see SoA.

Blood will be drawn for DNA, plasma and serum banking purposes. Any analyses on DNA or plasma/serum samples will not be reported in the main clinical trial report. Samples will be collected only in countries where all applicable local regulatory and ethics approvals have been obtained for biobanking.

5.6 OTHER ASSESSMENTS

5.6.1 Immunogenicity

5.6.1.1 Timing of immunogenicity measures

Blood samples will be obtained from trial participants at the time points detailed in the SoA. Serum samples will be analysed for the presence of ADAs and NAbs. ADAs will be

Proprietary confidential information © 2025 Boehringer Ingelheim International GmbH or one or more of its affiliated companies

evaluated for their specificity towards survodutide and their ability to cross-react with GLP-1 or glucagon. Similarly, NAbs will be evaluated towards their ability to neutralise survodutide, and may also be evaluated for their ability to cross-react and neutralise endogenous GLP-1 or glucagon. The impact of ADAs/NAbs on PK, clinical safety and efficacy will be evaluated.

Additionally, blood for ADA and NAb assessment will be collected upon observation of severe or systemic hypersensitivity AEs, and the serum concentration of survodutide will be assessed.

5.6.1.2 Methods of sample collection

Detailed instructions on sampling, preparation, processing, shipment, and storage are provided in the laboratory manual.

For both, ADA (incl. cross-reactive binding antidrug antibodies) and NAb (incl. cross-neutralization of GLP-1 and glucagon) assessments, blood will be taken for each time point described in the SoA via venipuncture or indwelling catheter into serum blood-drawing tubes at the time points listed in the Flow Chart, and additionally in the event of any severe hypersensitivity reaction as mentioned above.

At a minimum, the sample tube labels should list the following information: BI trial number, trial participant number, visit, matrix, and analyte (ADA or NAb).

Serum samples will be shipped on dry ice and stored in a freezer set at -70°C until and after analysis. The samples may be used for further ADA or NAb characterization or methodological investigations (e.g. for stability testing, isotyping, cross-reactivity, cross-neutralization), however, only data related to ADAs or NAbs will be generated by these additional investigations. The trial samples will be discarded after completion of the additional investigations but not later than 5 years after the final clinical trial report has been signed.

All lab kit supplies will be provided by the central lab vendor designated by the sponsor.

5.6.1.3 Immunogenicity analysis

The presence of ADAs to survodutide will be assessed via a tiered approach using validated assays. All pre- and post-dose samples will be tested in the screening assay and positive samples in the screening assay will be tested in the confirmatory assay. Trial samples that are confirmed positive may be further characterised for titer assessment, cross-reactivity to GLP1, cross-reactivity to glucagon, neutralization of survodutide activity on GLP1R and neutralization of survodutide activity on GCGR. Samples that are positive for cross-reactivity to GLP1 will be tested for cross-neutralization of GLP1. Samples that are positive for cross-reactivity to glucagon will be tested for cross-neutralization of glucagon.

5.6.2 Clinical staging of obesity

Staging of obesity will be assessed by the investigator at screening using the Edmonton obesity staging, based on the medical history. The Edmonton obesity staging system is used for clinical prioritisation and management of obesity independent of BMI [R20-1982]. For details, refer to Section 10.5.

Proprietary confidential information © 2025 Boehringer Ingelheim International GmbH or one or more of its affiliated companies

[redacted]

5.7 APPROPRIATENESS OF MEASUREMENTS

All measurements performed during this trial are standard measurements and will be performed in order to evaluate efficacy and tolerability of survodutide and to determine PK and PD parameters in an appropriate way. The scheduled measurements will allow monitoring of changes in vital signs, standard laboratory values, and ECG parameters that might occur as a result of administration of the trial drug. The safety assessments are standard, are accepted for evaluation of safety and tolerability of an s.c. administered drug and are widely used in clinical trials. The PK parameters and measurements outlined are generally used assessments of drug exposure.

Proprietary confidential information © 2025 Boehringer Ingelheim International GmbH or one or more of its affiliated companies

6. INVESTIGATIONAL PLAN

6.1 VISIT SCHEDULE

For a detailed overview of the trial procedures, in-clinic and remote visits (telemedicine, if allowed per local regulations), and time windows for visits, please refer to the SoA.

The trial consists of a screening visit followed by a dose escalation period of up to 24 weeks (or up to a maximum of 32 weeks if 1 or 2 re-escalation attempts are needed due to occurrence of GI symptoms), a maintenance period of 24 weeks, and an FU/EOS Visit. After completion of the main treatment period of 48 weeks, participants may agree to an optional extended treatment period, which includes an additional 4 weeks at the previously assigned dose. The FU Visit for safety assessments is scheduled 28 days after the last administration of the IMP (21 days after the Week 48/EOT Visit or after Week 52/EOT for patients who agreed to the optional extended treatment period).

A pre-screening approach is recommended in this trial. The study site will be able to review their records in a structured process to identify potential subjects who would most likely qualify for the trial (e.g. overweight/ obesity, presence of metabolic comorbidities, previous NAFLD/NASH diagnosis, biochemical liver tests and non-invasive liver fibrosis tests). Time windows of ±3 days (dose escalation period) or ±7 days (maintenance period and follow-up) for scheduled visits are allowed to accommodate scheduling problems. These time windows apply to scheduled visits, not to IMP administration, which may not occur on the same day as a visit. Time windows for IMP administration are outlined in Section 4.1.4. If a delay is observed for a particular visit, the original calendar schedule should be kept for subsequent visits (delays should not accumulate).

If a visit is missed, the trial participant should be instructed to reach out to the investigator as soon as possible. Handling of missed IMP injections is described in Section 4.1.4. The investigator should contact the CRO monitor to discuss the further schedule.

Trial participants who completed the trial treatment as planned will have an FU/EOS Visit 21 days after the EOT visit following the completion of the treatment period. For more details (e.g. for trial participants who discontinued prematurely), refer to Section 6.2.3. Procedures to be completed at the FU Visit can be found in the SoA. The sequence of visit procedures will be the same as in the treatment period.

6.2 DETAILS OF TRIAL PROCEDURES AT SELECTED VISITS

Please refer to Section 5 for explanations of procedures or observations and to the SoA for corresponding time points.

An electronic Study Hub with a secure online platform from an external CRO will be used in this study. This Study Hub is the place where the study site staff can communicate with the trial participant (including video conference), complete study tasks, and receive important information about the study. Trial participants will access the Study Hub using a secure app on a smartphone or tablet or by using a secure website through any computer. Trial participant will have "Study Hub Technical Support" that is accessible 24 h/day, 7 days/week.

Proprietary confidential information © 2025 Boehringer Ingelheim International GmbH or one or more of its affiliated companies

The eCOA platform will be integrated with the Study Hub. The eCOA platform will include electronic PROs as well as the eDiary. The e-consent process will not be integrated with the Study Hub, but with the “Complete Consent“ platform.

Electronic tools will be implemented only in participating countries where this is permitted based on applicable approvals from local regulatory authorities and ethics committees. Otherwise, a traditional non-electronic approach will be used. In exceptional cases, if a trial participant is unable to use the electronic platforms, a back-up solution with paper will be made available upon sponsor approval.

6.2.1 Screening period

Screening of participants for this trial is competitive, i.e. screening for the trial will stop at all sites at the same time once a sufficient number of participants has been screened. Investigators will be notified about screening completion and will then not be allowed to screen additional participants for this trial. Participants already in screening at this time will be allowed to continue to randomisation if eligible.

Whilst the information provided below is not an exhaustive list, it provides some guidance as to when such re-screening and/or re-testing would be considered appropriate.

Re-testing for eligibility criteria (within the defined screening window period):

- Is only to be performed once for ECG, vital signs, and a laboratory result which is obviously received beyond stability at the central laboratory, thought to be a spurious result based on previously available laboratory results, or if the Investigator believes the ineligible result is caused by an error or extenuating circumstance.
- The re-test should be carried out as soon as possible so that the laboratory test results can be available prior to the randomisation visit.

Re-screening

- Re-screening of the same trial participant is only allowed once.
- The trial participant should be declared a screening failure in the eCRF and IRT with their original trial participant number.
- Upon re-screening, the IRT system will allocate a new screening number for the trial participant.
- All procedures of Visit 1 must be repeated except for FibroScan® and MRI. FibroScan® and MRI from the initial screening can be used if the assessments were performed within 1 month prior to re-screening. Data for FibroScan® and MRI from the initial screening failure will be transferred to the corresponding re-screening number.
- Trial participants who were considered ineligible because of uncontrolled hypertension (see Section 3.3.3), can be re-screened at the discretion of the investigator after good blood pressure control has been achieved.

Re-screening of a previously screen failed patient will be permitted providing the reasons for screen failure were reversible and have been resolved, based on Investigator’s judgement. A log of all participants enrolled in the trial (i.e. who have signed informed consent) will be

Proprietary confidential information © 2025 Boehringer Ingelheim International GmbH or one or more of its affiliated companies

maintained in the ISF, irrespective of whether or not IMP was administered to the participant. If retrospectively it is found that a trial participant has been randomised in error (i.e. did not meet all inclusion criteria or met one or more exclusion criteria), the sponsor or delegate should be contacted immediately. Based on an individual benefit-risk assessment, a decision will be made as to whether continued trial participation is possible.

Screening

During the screening visit, demographics and baseline information will be collected:

- Age (in years) on the day of informed consent
- Sex (male or female, in order to describe the trial participant's sex at birth)
- Gender identity (male, female, non-binary, not answered, or other in order to describe how the trial participant self-identifies, regardless of their genotypic or phenotypic sex) if 1) locally accepted (i.e. based on HA/EC/IRB acceptance, independent of acceptance by investigators or participants), 2) investigators are willing to ask, and 3) participants are willing to reply
- For women of childbearing potential, yes/no to characterize the patient population and as a basis for contraception requirements
- Ethnicity and race to sufficiently characterise the patient population, to support possible subgroup analyses if needed, unless not acceptable according to local regulations
- Medical and weight history, baseline conditions and concomitant therapy
- Height and weight (for obesity staging)
- Physical examination, vital signs (SBP, DBP, pulse), and a 12-lead ECG
- PHQ-9 and C-SSRS
- Eye examination for T2DM subjects
- Blood samples (for safety laboratory tests and infectious serology)
- FIB-4 score
- Review of inclusion and exclusion criteria
- MAST score (for calculation see Section 3.3.2)
- FAST score (for calculation see Section 2.2.2)
- ELF score

If the trial participant meets no exclusion criteria and fulfils at least one of the three conditions below:

- Recent liver biopsy (within 3 years) consistent with NASH (defined as the presence of steatosis, inflammation, and ballooning) with stage ≤3 fibrosis according to the NASH Clinical Research Network classification OR
- FIB-4 score ≥2.67 and <3.48 at screening OR
- Non-invasive test results in the previous 6 months: MRE ≥2.61 and <4.68 kPa, OR MAST score ≥0.242, OR FAST score ≥0.5, OR FibroScan® VCTE ≥8 kPa and <20 kPa), OR ELF score >7.7 and <11.3, OR cT1 ≥875 ms

Proprietary confidential information © 2025 Boehringer Ingelheim International GmbH or one or more of its affiliated companies

then the FibroScan® VCTE™ + CAP™ (VCTE™ only if CAP™ not available) should be performed. In case VCTE <20 kPa, MRI to measure of MRI-PDFF, cT1 and MRE should be performed. In case VCTE ≥20 kPa, the participant should be registered as a screen failure in the IRT and eCRF screen failure page should be recorded.

Participants not fulfilling at least one of the three conditions mentioned above and FIB-4 score <1.3 should be registered as screen failures in the IRT and eCRF screen failure page should be recorded.

For the remaining participants with FIB-4 score ≥1.3 and <2.67, the FibroScan® VCTE™ + CAP™ (VCTE™ only if CAP™ not available) should be performed to assess the FAST score and/or the VCTE results for eligibility criteria. In case FAST ≥0.5 or VCTE ≥8 kPa (and VCTE <20 kPa), MRI should be performed. In case FAST score/VCTE inclusion criteria are not met (or VCTE ≥20 kPa), the participant should be registered as a screen failure in the IRT and eCRF screen failure page should be recorded. Figure 3 shows the flowchart of assessments at Visit 1.

Proprietary confidential information © 2025 Boehringer Ingelheim International GmbH or one or more of its affiliated companies

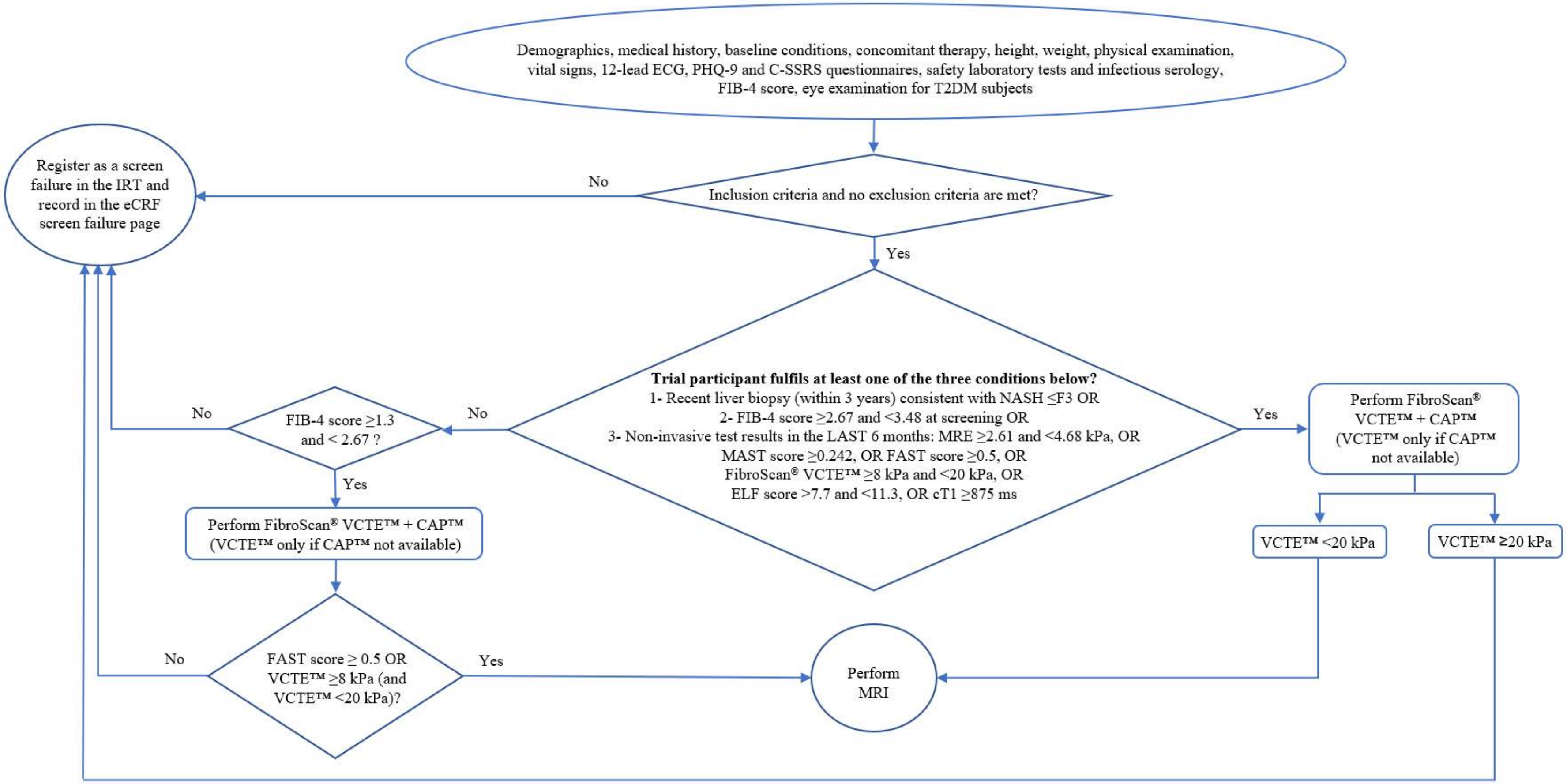

Figure 3 Flowchart of assessments at Visit 1

Proprietary confidential information © 2025 Boehringer Ingelheim International GmbH or one or more of its affiliated companies

6.2.2 Treatment period

[redacted]

Trial participants who meet all protocol criteria will be randomised at Visit 2. After randomisation, trial participants will start the treatment period which includes a dose escalation phase, followed by the dose maintenance phase as shown in Figure 2.

[redacted]

Procedures to be completed at each visit can be found in the SoA.

At visits specified in the SoA, trial participants should be instructed to come in the fasting state after at least 8 h without food or liquid intake, except water. If the IMP injection date is scheduled during the date of the visit, the trial participant needs to wait with injection of the IMP as this will be done on site after blood sampling.

The in-clinic visit should be scheduled in the mornings, if this is convenient for the trial participant.

Sequence of assessments

For in-clinic visits after randomisation and review of inclusion/exclusion criteria, the suggested sequence of assessments (as applicable) is:

1. Completion of questionnaires (to be performed in a fasted state, should be the first assessment)
2. Vital signs and other applicable assessments, including weight (to be performed in a fasted state)
3. ECG (to be performed in a fasted state and always before blood draws)
4. Blood draws (to be performed in a fasted state) and urine collection
5. Physical examination
6. Dosing with IMP

[redacted]

5. PHQ-9
6. C-SSRS

Proprietary confidential information © 2025 Boehringer Ingelheim International GmbH or one or more of its affiliated companies

At each visit, trial participants should be advised to follow the recommended diet and exercise plan and to record their compliance regularly (at least weekly) on the provided food, exercise, and weight diary. The trial participants can also use their own tools (e.g. paper or local applications) that they have been using prior to enrolment for tracking their diet, body weight, and physical activity. Diet and exercise counselling sessions are held at visits indicated in the SoA. When necessary, from a logistic point of view, it is possible to have the diet/exercise counselling on a different day than the actual in-clinic visit, but within the time window of the visit.

Unscheduled visits may be arranged if necessary. Procedures completed during an unscheduled visit will depend on the circumstances under which the visit was scheduled, and at the discretion of the investigator.

During the remote visits (if allowed per local regulations) the trial participants will be contacted by the site staff. They will be asked about their well-being, occurrence of AEs, and intake of concomitant medications (including standard of care for the weight-related complications). Site staff will conduct the C-SSRS assessment, ask trial participants if they have performed the IMP administration and whether it was documented in the eDiary. Trial participants will be asked if they have followed the diet and physical activity recommendations and have documented it in the paper food, exercise, and weight diary or their own tools. When applicable, site staff will also ask trial participants if they experienced any hypo/hyperglycaemic episodes and if so, whether they documented the glucose measurements for these episodes in the eDiary.

Remote visit responsibilities within the study team will be defined in the study delegation log on site level (e.g. assessment of concomitant therapy and AEs by a physician).

If remote visits are not allowed per local regulations, they will be performed as in-clinic visits.

Exceptionally, and after consultation with the sponsor, one visit could be completed a few days in advance to accommodate for trial participant's imperatives. The call to IRT and IMP allocation would be made, but the IMP injection would occur as per the initial schedule and allowed time window.

During the remote Visits 3 and 5 and 12 the staff may also observe the IMP administration and if needed, perform retraining of the trial participants on the administration technique. Remote visits could occur, by default, via video calls. In rare cases, the remote visits may be done by phone call. The trial participants will be trained by the study staff how to access the video conference application. All procedures to be performed during the remote visits are specified in the SoA.

After completion of the main treatment period, the trial participants will have a Week 48/EOT Visit 7 days after last IMP administration. Alternatively, participants may agree to enter an optional extended treatment period, which includes an additional 4 weeks at the previously assigned dose.

Trial participants who discontinue trial treatment will have an ETD Visit 7 days after the last IMP dose or as soon as possible.

Proprietary confidential information © 2025 Boehringer Ingelheim International GmbH or one or more of its affiliated companies

Safety lab and other laboratory tests:
The analysis will be performed by a central laboratory. The local lab would only be allowed to be documented in exceptional cases and should be discussed with the sponsor.

Minimum required safety lab parameters are:
Haematology: Haematocrit, haemoglobin, erythrocytes, leukocytes, thrombocytes
Clinical chemistry: ALT, AST, bilirubin total, amylase, lipase

6.2.3 Follow-up period and trial completion

Trial participants who completed the trial treatment as planned will have an FU/EOS Visit at least 21 days after the EOT visit after the completion of the treatment period. Procedures to be completed at the FU Visit can be found in the SoA. The sequence of visit procedures will be the same as in the treatment period.

All efforts should be made to follow-up on all trial participants until trial end.

Trial participants who discontinue the IMP prematurely and are willing to participate in trial-related activities will have the FU visit scheduled 21 days after the ETD visit and will be asked to continue the trial visits as planned, including the EOS Visit at Week 51 (see Section 3.3.4.1 for more details). The Week 51 and EOS eCRF pages must be completed at this visit, because for these participants no EOS will be performed separately as they will be monitored through the regular scheduled visits.

Trial participants who discontinue the IMP prematurely and are not willing to continue with trial activities should have an FU/EOS Visit 21 days after their ETD Visit.

If a trial participant is not willing to return to the predefined trial visits, at minimum a remote visit (if allowed per local regulations) or phone call at the FU/EOS Visit (that should occur 21 days after ETD visit) will be required to review the medical records.

Trial participants who withdraw consent should stop taking IMP and should be asked to complete the ETD Visit and follow-up procedures as described in the SoA and Section 3.3.4.1.

A trial participant is considered to have completed the trial if

- The trial participant did not discontinue treatment and completed scheduled visits until Week 48 Visit, and FU Visit

or

- The trial participant prematurely discontinued treatment but completed at a minimum Week 48 Visit, and (if applicable[1]) FU Visit

If needed in the opinion of the investigator, after the FU/EOS Visit additional visits may be scheduled for continued safety monitoring. Abnormal assessments or lab values judged clinically relevant by the investigator will be monitored until they returned to a medically acceptable level.

[1] If applicable per the CTP version in effect at time of the discontinuation.

Proprietary confidential information © 2025 Boehringer Ingelheim International GmbH or one or more of its affiliated companies

6.2.4 Exceptional conditions

In the event of force majeure or other disruptive circumstances (e.g. pandemic, war) the execution of the investigational plan as per this clinical trial protocol may not be feasible. With the consent of the participant, the sponsor and investigator may agree on alternative, back-up or rescue methodology which may include but will not be limited to virtual trial participant visits and assessments, direct-to-participant/direct-from-participant shipments of trial treatment or bio-sample pick up from the participant's home. The implementation of these measures will depend on participant's consent, operational feasibility, local law and regulations. If alternative methodology is implemented, the deviations from the original plan will be precisely documented.

Trial participants with obesity are in general at higher risk for severe illness from COVID-19. In exceptional cases, if standard visits at the trial sites are impossible because of COVID-19 related safety risks, the trial conduct may need to be adjusted, strictly consistent with local public health guidance and regulations. If, in order to minimize the risk of exposure to COVID-19, physical visits should be avoided, the use of remote visits (via phone and/or internet-based means of communication) and local laboratory services for safety and efficacy parameters measurements should be considered if possible. However, IMP shipment to trial participants might not be possible in all countries to be included. In such situations, picking up the IMP could be done by the trial participant's delegate or by the trial participant at a sterile pharmacy (e.g. "drive -through", "isolated" pharmacy). Investigators should complete trial procedures according to the clinical trial protocol to the extent possible. The maximum number of missing doses of IMP is defined in Section 4.1.4. Considering all aspects, the investigator will decide upon each trial participant's (continued) participation in the planned trial. BI as the sponsor, where required, will support the investigator in their decision finding. It is acknowledged that the investigator may decide to implement protocol deviations where this protects the safety, wellbeing and/or is in the best interest of the trial participant. All home/remote visits need to be discussed with and approved by the sponsor`s trial team. Local regulatory and legal requirements of the participating country still apply. All COVID-19 related deviations from the original schedule of visits and procedures will be documented and the implications considered for the analysis of the trial data.

Proprietary confidential information © 2025 Boehringer Ingelheim International GmbH or one or more of its affiliated companies

7. STATISTICAL METHODS AND DETERMINATION OF SAMPLE SIZE

7.1 NULL AND ALTERNATIVE HYPOTHESES

The hypotheses reflecting the primary objective are as follows:

Let OR1_ survodutide/placebo denote the true odds ratio of achievement of MRI-PDFF response ≥30% from baseline to Week 48 between survodutide and placebo.

- $H0_1$: OR1_ survodutide/placebo ≤1 versus HA_1: OR1_ survodutide/placebo >1

Let µ1_ survodutide and µ1_placebo denote the true mean of percentage change in body weight from baseline to Week 48 for survodutide and placebo, respectively.

- $H0_2$: µ1_ survodutide ≥ µ1_placebo versus HA_2: µ1_ survodutide <µ1_placebo

The trial will be considered positive if based on the treatment-regimen estimand both hypotheses $H0_1$ and $H0_2$ are rejected. The overall two-sided significance level is α=5%.

7.2 PLANNED ANALYSES

7.2.1 General considerations

Analysis sets

- Randomised set (RS): the RS includes all randomised trial participants.
- Treated set (TS): the TS is defined as all randomised trial participants who received at least one dose of IMP.

All efficacy and safety analyses will be based on the treated set.

Important protocol deviations
IPD categories will be defined in the IPD specification document.

IPDs will be identified no later than in the Report Planning Meeting. Identification of patients with IPDs will be confirmed at RPM and the patients will be listed in the CTR.

IPDs may include (but not necessarily be limited to) the following:

- Violation of inclusion or exclusion criteria
- Major deviations from scheduled timing of assessments
- Non-compliance with IMP
- Treatment dispensing errors (handling of such participants will be decided on case-by-case basis by the sponsor)
- Use of prohibited or restricted concomitant medication

Proprietary confidential information © 2025 Boehringer Ingelheim International GmbH or one or more of its affiliated companies

Definition of baseline

In general, unless otherwise specified in this document or in the TSAP, the last non-missing measurement on or prior to the date of first dose of IMP will be used as baseline for efficacy and safety variables.

For the purposes of on-treatment safety analyses, an assessment (or AE start date) will be considered "on-treatment" if the assessment date (or AE start date) is between the date of first dose and 28 days after the date of last dose of the IMP.

7.2.2 Handling of Intercurrent Events

The expected ICEs of interest in this trial are listed in Table 13 together with their respective handling. The handling of intercurrent events, that are not listed, will be described in the TSAP.

Table 13 Handling of intercurrent events

**Intercurrent event (ICE)**	**"Treatment-regimen" estimand (ICE handling)**	**"Efficacy" estimand (ICE handling)**
Premature treatment discontinuation	Include all available data<br>("Treatment policy" strategy)	Exclude data after treatment discontinuation + 28 days (residual effect period of survodutide)<br>("Hypothetical" strategy)
Intake of prohibited anti-obesity medication (other than GLP1R agonist containing medication) while on treatment	Include all available data<br>("Treatment policy" strategy)	Exclude data during intake of any prohibited anti-obesity medication<br>("Hypothetical" strategy)
Intake of prohibited anti-obesity medication (other than GLP1R agonist containing medication) after premature discontinuation of study drug	Include all available data<br>("Treatment policy" strategy)	Exclude data during intake of any prohibited anti-obesity medication<br>("Hypothetical" strategy)
Intake of prohibited medication containing GLP1R agonists	Include all available data<br>("Treatment policy" strategy)	Exclude all data after first intake of GLP1R agonist containing medication<br>("Hypothetical" strategy)
Death from any cause	Include data prior to death ("Hypothetical" strategy for both continuous and binary endpoints)	Include data prior to death ("Hypothetical" strategy for both continuous and binary endpoints)

There will be 2 estimands of interest addressing the primary and secondary objectives: treatment-regimen estimand and efficacy estimand.

Proprietary confidential information © 2025 Boehringer Ingelheim International GmbH or one or more of its affiliated companies

Treatment-regimen estimand

T.1) Treatment regimen under evaluation will be the randomised treatment taken for up to 48 weeks (with a dose escalation of survodutide/placebo during the first 24 weeks after randomisation) as an adjunct to a reduced-calorie diet and increased physical activity and including certain anti-hyperglycaemia medication (rescue therapy), certain glucose-lowering, anti-hypertensive, and lipid-lowering medication, and possibly anti-obesity medication.

T.2) Target population: The treatment effect will be estimated for trial participants with a BMI ≥30 kg/m^2 or a BMI ≥27 kg/m^2 with one or more weight-related co-morbidities and presumed or confirmed NASH.

T.3) Endpoint(s): Efficacy will be evaluated using the co-primary endpoints "Relative reduction in liver fat content of at least 30% from baseline to Week 48 assessed by MRI-PDFF (yes/no)" and "Relative (%) change in body weight from baseline to Week 48".

T.4) Intercurrent events:

- For trial participants who initiate a prohibited anti-obesity medication other than GLP1R agonist containing medication during treatment with IMP or after discontinuation from IMP, who initiate a prohibited medication containing GLP1R agonists, or who discontinue prematurely from IMP for any reason, observed endpoint values through Week 48 (in T.3) will be used for the analysis.
- For trial participants who die, a hypothetical strategy will be used for the endpoint "Relative change (%) in body weight from baseline to Week 48" to estimate what the treatment effect would be at Week 48 if the trial participants had not died. The binary endpoint "Relative reduction in liver fat content of at least 30% from baseline to Week 48 assessed by MRI-PDFF (yes/no)" will be derived from the continuous endpoint MRI-PDFF liver fat, if available, hence, a hypothetical strategy will be used as well.

T.5) Population-level summary: Adjusted mean "Percentage of change in body weight from baseline to Week 48" will be computed for each randomised treatment group. The survodutide group will be compared with the placebo group using difference of adjusted means. The null hypothesis of the difference in adjusted means (survodutide - placebo) being greater than or equal to zero will be tested.

The proportion of trial participants with "Relative reduction in liver fat content of at least 30% from baseline to Week 48 assessed by MRI-PDFF (yes/no)" will be compared between survodutide and placebo treatment in terms of odds ratios. The null hypothesis of the odds ratio (survodutide/placebo) being less than or equal to one between survodutide and placebo group will be tested.

Efficacy estimand

E.1) Treatment regimen under evaluation will be the randomised treatment taken for up to 48 weeks (with a dose escalation of survodutide/placebo during the first 24 weeks after randomisation) as an adjunct to a reduced-calorie diet and increased physical activity, but excluding certain anti-hyperglycaemia medication (rescue therapy), certain glucose-lowering, anti-hypertensive and lipid-lowering medication, and possibly anti-obesity medication.

Proprietary confidential information © 2025 Boehringer Ingelheim International GmbH or one or more of its affiliated companies

E.2) Target population: The treatment effect will be estimated for trial participants with a BMI ≥30 kg/m^2 or a BMI ≥27 kg/m^2 with one or more weight-related co-morbidities and presumed or confirmed NASH.

E.3) Endpoint(s): Efficacy will be evaluated using the co-primary endpoints "Relative reduction in liver fat content of at least 30% from baseline to Week 48 assessed by MRI-PDFF (yes/no)" and "Relative (%) change in body weight from baseline to Week 48".

E.4) Intercurrent events:

- For trial participants who initiate an anti-obesity medication other than GLP1R agonist containing medication during treatment with IMP or after discontinuation from IMP, all body weight measurements during intake of the anti-obesity medication will be excluded from the analysis. However, the endpoint measurement at Week 48 will be used in the analysis if the trial participant continued randomised treatment through Week 48 and stopped intake of any anti-obesity medication other than GLP1R agonist containing medication before Week 48.
- For trial participants who discontinue prematurely from IMP for any reason, all body weight measurements and MRI-PDFF liver fat data after discontinuation + 28 days (residual effect period of survodutide) will be excluded from the analysis. That is, a hypothetical strategy will be used to estimate what the treatment effect would be at Week 48 if trial participants had continued on their randomised treatment through Week 48.
- For trial participants who initiate prohibited medication containing GLP1R agonists, all body weight measurements and MRI-PDFF liver fat data after start of medication containing GLP1R agonists will be excluded from the analysis. That is, a hypothetical strategy will be used to estimate what the treatment effect would be at Week 48 if trial participants had not started the prohibited medication containing GLP1R agonists.
- For trial participants who die, a hypothetical strategy will be used for the endpoint "Relative (%) change in body weight from baseline to Week 48" and endpoint "Relative reduction in liver fat content of at least 30% from baseline to Week 48 assessed by MRI-PDFF (yes/no)" to estimate what the treatment effect would be at Week 48 if the trial participants had not died.

E.5) Population-level summary: Adjusted mean "Percentage change from baseline in body weight from baseline to Week 48" will be computed for each randomised treatment group. The survodutide group will be compared with the placebo group using difference of adjusted means. The null hypothesis of the difference in adjusted means (survodutide – placebo) being greater than or equal to zero, will be tested.

The proportion of trial participants with "Relative reduction in liver fat content of at least 30% from baseline to Week 48 assessed by MRI-PDFF (yes/no)" will be compared between survodutide and placebo treatment in terms of odds ratios. The null hypothesis of the odds ratio (survodutide/placebo) being less than or equal to one between survodutide and the placebo group will be tested.

Proprietary confidential information © 2025 Boehringer Ingelheim International GmbH or one or more of its affiliated companies

The primary efficacy analysis will be conducted using treatment-regimen estimand. No multiplicity adjustment will be made for conducting separate analyses relative to efficacy and treatment-regimen estimands, as these two estimands are intended for different purposes.

7.2.3 Primary objective analyses

The treatment-regimen estimand reflects the treatment effect which includes the effects of any early treatment discontinuation and any prohibited anti-obesity treatment. In addition, the efficacy estimand reflects the treatment effect if all trial participants had adhered to treatment and had not received any prohibited anti-obesity therapy. Both estimands evaluate the co-primary endpoints in all randomised and treated trial participants (treated set).

7.2.3.1 Main analyses

Primary analyses relative to the estimands as defined above:

Analysis relative to the treatment-regimen estimand:

Analysis of covariance (ANCOVA) with multiple imputation of missing endpoint data guided by Rubin [R12-2378] will be performed for the continuous primary endpoint “relative (%) change in body weight from baseline to Week 48”. Missing body weight data at Week 48 will be imputed based on ‘retrieved dropouts’, defined as participants who had their value measured at Week 48 in the same treatment arm who prematurely discontinued study drug. In cases where there are not enough retrieved dropouts to provide a reliable imputation model (i.e. the model does not converge), multiple imputation will be based on the ‘follow the reference’ strategy. That is, after ICE ‘treatment discontinuation’, trial participants in the survodutide arm are assumed to have an effect similar to that of the placebo arm while preserving information of any on-treatment and off-treatment data. ANCOVA will be applied to the relative change in body weight from baseline to Week as response variable and will include treatment,region and T2DM status at baseline as fixed effects, and baseline body weight as a covariate.

A logistic regression with fixed categorical effects of treatment, region and T2DM status at baseline, will be used to analyse the occurrence of “Relative reduction in liver fat content of at least 30% from baseline to Week 48 assessed by MRI-PDFF (yes/no)”. Odds ratios will be reported. The responder/non-responder (yes/no) status will be based on the continuous liver fat measurements, or, in the case of missing liver fat values, on the continuous values imputed with the methods described in the previous paragraph. Statistical inference over imputed data will also be guided by Rubin’s rule.

The feasibility (and the variance) of the pre-specified retrieved dropout primary analysis model in the first step is extremely dependent upon the proportion of off-treatment measurements at Week 48 that are missing. Consequently, although every effort will be made to ensure that as much off-treatment data as possible is collected, if the proportion of off-treatment data at Week 48, that is collected, is below 40%, then the ‘follow the reference’ strategy will instead be adopted as the primary analysis (main estimation) and the retrieved dropout analysis will be used as sensitivity analysis. According to the ‘follow the reference’ strategy, after ICE ‘treatment discontinuation’, trial participants in the experimental arms are assumed to have an effect similar to that of the placebo arm while preserving information of any on-treatment and off-treatment data.

Proprietary confidential information © 2025 Boehringer Ingelheim International GmbH or one or more of its affiliated companies

Further details will be provided in the TSAP. The TSAP will be finalized prior to database lock.

7.2.3.2 Sensitivity Analyses

Sensitivity analyses will be specified in the TSAP.

[redacted]

7.2.3.4 Supplementary Analyses

Analysis relative to the efficacy estimand:

For the continuous primary endpoint "relative (%) change in body weight from baseline to Week an MMRM will be used with region, T2DM status at baseline, baseline body weight by visit interaction, and treatment by visit interaction, as fixed effects. Missing values will be implicitly handled by the MMRM under the MAR (Missing at random) assumption.

The Kenward-Roger approximation will be used to estimate denominator degrees of freedom and adjust standard errors. Significance tests will be based on contrasts at the primary endpoint time point (Week 48) using a two-sided $\alpha = 0.05$ (two-sided 95% confidence intervals). Unstructured covariance will be assumed to model the relationship between pairs of measurements taken at different weeks on the same patient. Strategies for handling model convergence issues will be specified in the TSAP.

A logistic regression with fixed categorical effects of region, T2DM status, treatment, and the fixed continuous effect of baseline liver fat content assessed by MRI-PDFF will be used to analyse the occurrence of "Relative reduction in liver fat content of at least 30% from baseline to Week 48 assessed by MRI-PDFF (yes/no)". The responder/non-responder (yes/no) status will be based on the continuous liver fat measurements. Missing liver fat values will be adressed via multiple imputation and then dichotomized. More details are provided in the TSAP.

Additionally, and following the FDA's guidance for industry "Adjusting for Covariates in Randomized Clinical Trials for Drugs and Biological Products" an estimate of the unconditional treatment effect on the binary endpoint "Relative reduction in liver fat content of at least 30% from baseline to Week 48 assessed by MRI-PDFF (yes/no)" will be provided. Details on the selected method and its implementation will be provided in the TSAP.

Further supplementary estimands will be specified in the TSAP, if requested by regulatory authorities.

7.2.4 Secondary objective analyses

Secondary objectives are listed in Section 2.1.3. Analyses for secondary efficacy endpoints will be described in the TSAP.

Proprietary confidential information © 2025 Boehringer Ingelheim International GmbH or one or more of its affiliated companies

7.2.5 Further objective analyses

Further objectives are listed in Section 2.2.1. Analyses for further efficacy endpoints will be described in the TSAP.

[redacted]

7.2.6 Safety analyses

AEs will be coded using the Medical Dictionary for Drug Regulatory Activities (MedDRA). Standard Boehringer Ingelheim summary tables and listings will be produced. All AEs with an onset between start of treatment and end of the REP, a period of 28 days after the last dose of trial IMP, will be assigned to the on-treatment period for evaluation.

All treated trial participants will be included in the safety analysis. In general, safety analyses will be descriptive in nature and will be based on Boehringer Ingelheim standards. No hypothesis testing is planned.

Frequency, severity, and causal relationship of AEs will be tabulated by system organ class and preferred term after coding according to the current version of the Medical Dictionary for Drug Regulatory Activities (MedDRA) at database lock.

Laboratory data will be analysed both quantitatively as well as qualitatively. The latter will be done via comparison of laboratory data to their reference ranges. Values outside the reference range as well as values defined as clinically relevant will be summarised. Treatment groups will be compared descriptively with regard to distribution parameters as well as with regard to frequency and percentage of trial participants with abnormal values or clinically relevant abnormal values.

Vital signs, physical examinations, or other safety-relevant data observed at screening, baseline, during the course of the trial, and at the end-of-trial evaluation will be assessed with regard to possible changes compared with findings before the start of treatment.

Further details will be provided in the TSAP.

7.2.7 Other Analyses

Details on other analyses will be described in TSAP, if needed.

[redacted]

Proprietary confidential information © 2025 Boehringer Ingelheim International GmbH or one or more of its affiliated companies

7.3 HANDLING OF MISSING DATA

There will be no imputation for safety data. For trial participants who discontinue the IMP prematurely, all efforts will be made to follow trial participants for vital status and for any other endpoints including the primary endpoints, until EOS.

For the treatment-regimen estimand, missing data will be multiply imputed by a 'retrieved dropout' approach as described in Section 7.2.3.

For the efficacy estimand, missing data will be implicitly handled by an MMRM model for the continuous endpoint, and multiply imputed (and dichotomized) for the responder (binary) endpoint. More details are provided in the TSAP.

7.4 RANDOMISATION

Boehringer Ingelheim will arrange for the randomisation and the packaging and labelling of trial IMP. The randomisation list will be generated using a validated system, which involves a pseudo-random number generator so that the resulting treatment will be both reproducible and non-predictable. Specific parameters used for the creation of the randomization schedule (e.g. block size or biasing coin probabilities) will be documented in the Clinical Trial Report. Access to the codes will be controlled and documented.

Trial participants will be randomised in blocks using a 2:1 allocation to 1 of 2 double-blind treatment groups. Randomisation will be performed at the start of the dose escalation period at Visit 2 (starting dose and timing of dose escalation as outlined in Table 4), with a 2:1 allocation to survodutide 6.0 mg once weekly or placebo once weekly.

Randomisation will be stratified by the presence of T2DM.

In addition, an adjustable screening cap will be implemented in the IRT system to ensure that enough male and female trial participants are enrolled and randomised. Cap for number of female trial participants is approximately 70%.

7.5 DETERMINATION OF SAMPLE SIZE

Participants will be randomised in a 2:1 ratio to survodutide 6.0 mg or placebo. It is planned to randomise approximately 160 participants. The sample size needed to show superiority of survodutide over placebo for the co-primary endpoints was calculated under the following assumptions:

- Relative reduction in liver fat content of at least 30% from baseline to Week 48 assessed by MRI-PDFF (yes/no)
- Placebo effect: 30%
- Survodutide effect: 60%
- 95% power
- Alpha = 0.05 two-sided

The assumed response rates include a 10-15% non-responder rate for participants that prematurely discontinue and MRI-PDFF data cannot be imputed otherwise, which are therefore handled as non-responders.

Proprietary confidential information © 2025 Boehringer Ingelheim International GmbH or one or more of its affiliated companies

On the basis of these assumptions, randomising 156 participants (104 to survodutide and 52 to placebo) will provide 95% power to establish superiority for survodutide compared with placebo at a 2-sided significance level of 0.05.

- Relative (%) change in body weight from baseline to Week 48
- Placebo effect: 3%
- Survodutide effect: 10% (assumed in a mixed population with and without T2DM; 12% in participants without T2DM, 8% in participants with T2DM, assumed ratio 1:1)
- SD=10% for survodutide and SD=6% for placebo
- 95% power
- Alpha=0.05
- 15% of participants discontinue treatment permanently and assumed to have same effect as participants who completed the trial on placebo

[redacted]

On the basis of these assumptions, randomising 96 participants (64 to survodutide and 32 to placebo) will provide 95% power to establish superiority for a survodutide dose compared with placebo at a 2-sided significance level of 0.05. The t-test on the mean difference assuming unequal variances and unequal allocation ratio was used for calculation.

Given these assumptions, a sample size of approximately 160 participants (at least 156; 104 randomised to survodutide and 52 to placebo) would be required to test both co-primary endpoints. Both sample sizes were done on 95% to have an overall power of 90%.

Calculations were performed using nQuery Advisor® 6.1 statistical package by Statistical Solutions Ltd. The assumptions for the sample size calculations are based on the results from the STEP 1 trial [R21-1460], STEP 2 trial [R21-1465], Phase II trial 1404-0036 [c31754142] and [R23-4361].

Proprietary confidential information © 2025 Boehringer Ingelheim International GmbH or one or more of its affiliated companies

8. INFORMED CONSENT, TRIAL RECORDS, DATA PROTECTION, PUBLICATION POLICY, AND ADMINISTRATIVE STRUCTURE

The trial will be carried out in compliance with the protocol, the ethical principles laid down in the Declaration of Helsinki, in accordance with the ICH Harmonized Guideline for GCP, relevant Boehringer Ingelheim SOPs, EU regulation 536/2014, and other relevant regulations. Investigators and site staff must adhere to these principles. Deviation from the protocol, the principles of ICH GCP, or applicable regulations will be treated as 'protocol deviation'.

Standard medical care (prophylactic, diagnostic, and therapeutic procedures) remains the responsibility of the treating physician of the trial participant.

The investigator will inform the sponsor or delegate immediately of any urgent safety measures taken to protect the trial participants against any immediate hazard, as well as of any serious breaches of the protocol or of ICH GCP.

The Boehringer Ingelheim transparency and publication policy can be found on the following web page: trials.boehringer-ingelheim.com. The rights of the investigator and of the sponsor with regard to publication of the results of this trial are described in the investigator contract. As a rule, no trial results should be published prior to finalisation of the clinical trial report.

The certificate of insurance cover is made available to the investigator and the trial participants and is stored in the ISF.

8.1 TRIAL APPROVAL, PATIENT INFORMATION, INFORMED CONSENT

This trial will be initiated only after all required legal documentation has been reviewed and approved by the respective Institutional Review Board (IRB)/Independent Ethics Committee (IEC) and competent authority (CA) according to national and international regulations. The same applies for the implementation of changes introduced by amendments.

Prior to participation in the trial, written informed consent must be obtained from each participant according to ICH GCP and the regulatory and legal requirements of the participating country. Electronic systems will be used to support the consent process (eConsent) where it is permissible. Each signature must be personally dated by each signatory and the informed consent form and any additional participant-information form retained by the investigator as part of the trial records. A signed copy of the informed consent and any additional participant information must be given to each participant.

The trial participant must be given sufficient time to consider participation in the trial. The investigator or delegate obtains written consent of the trial participant's own free will with the informed consent form after confirming that the trial participant understands the contents. The investigator or delegate must sign (or place a seal on) and date the informed consent form. If a trial collaborator has given a supplementary explanation, the trial collaborator also signs (or places a seal on) and dates the informed consent.

Re-consenting may be necessary when new relevant information becomes available and should be conducted according to the sponsor's instructions.

The consent and re-consenting process should be properly documented in the source documentation.

Proprietary confidential information © 2025 Boehringer Ingelheim International GmbH or one or more of its affiliated companies

8.2 DATA QUALITY ASSURANCE

A risk-based approach is used for trial quality management. It is initiated by the assessment of critical data and processes for trial participant protection and reliability of the results, as well as identification and assessment of associated risks. An Integrated Quality and Risk Management Plan or alternative plan, in line with the guidance provided by ICH Q9 and ICH GCP E6, documents the rationale and strategies for risk management during trial conduct including monitoring approaches, vendor management, and other processes focusing on areas of greatest risk.

Continuous risk review and assessment may lead to adjustments in trial conduct, trial design, or monitoring approaches.

A quality assurance audit/inspection of this trial may be conducted by the sponsor, sponsor's designee, IRB/IEC, or regulatory authorities. The quality assurance auditor will have access to all medical records, the investigator's trial-related files and correspondence, and the informed consent documentation of this clinical trial.

8.3 RECORDS

CRFs for individual trial participants will be provided by the sponsor. See Section 4.1.5.2 for rules about emergency code breaks. For drug accountability, refer to Section 4.1.8.

8.3.1 Source documents

A detailed description of the transmission of electronic data (i.e. data flow) is provided in the data management plan.

In accordance with regulatory requirements, the investigator should prepare and maintain adequate and accurate source documents and trial records that include all observations and other data pertinent to the investigation on each trial participant. Source data as well as reported data should follow the "ALCOA principles" and be **a**ttributable, **l**egible, **c**ontemporaneous, **o**riginal, and **a**ccurate. Changes to the data should be traceable (audit trail).

Data reported on the CRF must be consistent with the source data, or the discrepancies must be explained.

The current medical history of the participant may not be sufficient to confirm eligibility for the trial, and the investigator may need to request previous medical histories and evidence of any diagnostic tests. In this case, the investigator must make at least one documented attempt to retrieve previous medical records. If this fails, a verbal history from the participant, documented in their medical records, would be acceptable.

Copies of source documents necessary (e.g. for adjudication of events and ECG central reading) will be provided to the corresponding vendor qualified by the sponsor for these assessments. Before sending or uploading those copies, the investigator must ensure that all participant identifiers (e.g. participant's name, initials, address, phone number, social security number) have been properly removed or redacted from all copies of the participant's source documents.

If the trial participant is not compliant with the protocol, any corrective action (e.g. re-training) must be documented in the participant file.

Proprietary confidential information © 2025 Boehringer Ingelheim International GmbH or one or more of its affiliated companies

For the CRF, data must be derived from source documents, for example:

- Participant identification: sex, year of birth (in accordance with local laws and regulations)
- Participant participation in the trial (substance, trial number, participant number, date participant was informed)
- Dates of participant's visits, including dispensing of trial IMP
- Medical history (including trial indication and concomitant diseases, if applicable)
- Medication history
- AEs and outcome events (onset date [mandatory] and end date [if available])
- SAEs (onset date [mandatory] and end date [if available])
- Concomitant therapy (start date, changes)
- Originals or copies of laboratory results and other imaging or testing results, with proper documented medical evaluation (in validated electronic format, if available)
- Completion of participant's participation in the trial (end date; in case of premature discontinuation, document the reason)
- Prior to allocation of a trial participant to a treatment in a clinical trial, there must be documented evidence in the source data (e.g. medical records) that the trial participant meets all inclusion criteria and does not meet any exclusion criteria. The absence of records (either medical records, verbal documented feedback of the participant, or testing conducted specific for a protocol) to support inclusion/exclusion criteria does not make the participant eligible for the clinical trial

8.3.2 Direct access to source data and documents

The investigator/institution will allow site trial-related monitoring, audits, IRB/IEC review, and regulatory inspections. Direct access must be provided to the CRF and all source documents/data, including progress notes and copies of laboratory and medical test results, which must always be available for review by the CRA, auditor, and regulatory inspector (e.g. FDA). They may review all CRFs and informed consent forms. The accuracy of the data will be verified by direct comparison with the source documents described in Section 8.3.1. The sponsor or delegate will also monitor compliance with the protocol and GCP.

In the event of force majeure or other disrupting circumstance (e.g. pandemic or war; see Section 6), site access may be restricted, thus limiting the ability to perform standard site monitoring activities on site, such as source data review and source data verification. Therefore, some of these activities may be performed remotely or replaced by centralized monitoring to the extent possible, based on a documented risk assessment and in alignment with local regulations.

8.3.3 Storage period of records

Trial sites: The trial sites must retain the source and essential documents (including ISF) according to the contract or local requirements valid at the time of the end of the trial (whichever is longer).

Sponsor: The sponsor must retain the essential documents according to the sponsor's SOPs.

Proprietary confidential information © 2025 Boehringer Ingelheim International GmbH or one or more of its affiliated companies

8.4 EXPEDITED REPORTING OF ADVERSE EVENTS

Boehringer Ingelheim is responsible for fulfilling their legal and regulatory reporting obligation in accordance with regulatory requirements.

8.5 STATEMENT OF CONFIDENTIALITY AND TRIAL PARTICIPANT PRIVACY

Data protection and data security measures are implemented for the collection, storage and processing of trial participant data in accordance with the principles 7 and 12 of the WHO GCP handbook.

To ensure confidentiality of records and personal data, only pseudonymised data will be transferred to the sponsor by using a participant identification number instead of the trial participant's name. The code is only available at the site and must not be forwarded to the sponsor. In case participant's records will be forwarded e.g. for SAE processing or adjudication committees, personal data that can identify the trial participant will be redacted by the site prior to forwarding. Access to the participant files and clinical data is strictly limited: personalised treatment data may be given to the trial participant's personal physician or to other appropriate medical personnel responsible for the trial participant's welfare. Data generated at the site as a result of the trial need to be available for inspection on request by the participating physicians, the sponsor's representatives, by the IRB/IEC and the regulatory authorities.

A potential data security breach will be assessed regarding the implications for rights and privacy of the affected person(s). Immediate actions as well as corrective and preventive actions will be implemented. Respective regulatory authorities, IRBs/IECs and trial participants will be informed as appropriate.

8.5.1 Collection, storage, and future use of biological samples and corresponding data

Measures are in place to comply with the applicable rules for the collection, biobanking, and future use of biological samples and clinical data, in particular:

- Sample and data usage must be in accordance with the separate biobanking informed consent
- The Boehringer Ingelheim internal facilities storing biological samples from clinical trial participants, as well as the external banking facility, are qualified for the storage of biological samples collected in clinical trials
- An appropriate sample and data management system, including audit trail for clinical data and samples to identify and destroy such samples according to ICF, is in place
- A fit for the purpose documentation (biomarker proposal, analysis plan and report) ensures compliant usage
- A fit for purpose approach will be used for assay/equipment validation depending on the intended use of the biomarker data

Proprietary confidential information © 2025 Boehringer Ingelheim International GmbH or one or more of its affiliated companies

- Samples and/or data may be transferred to third parties and other countries as specified in the biobanking ICF

8.6 TRIAL MILESTONES

The **first act of recruitment** represents the **start of the trial** and is defined as the date when the first trial participant in the whole trial signs informed consent.

The end of the trial is defined as the date of the last visit of the last trial participant in the whole trial ("last participant completed").

The "**last participant last treatment"** (LPLT) date is defined as the date on which the last trial participant in the whole trial is administered the last dose of trial IMP (as scheduled per protocol or prematurely). Individual investigators will be notified of SUSARs occurring with the trial IMP until 30 days after LPLT at their site.

Early termination of the trial is defined as the premature termination of the trial due to any reason before the end of the trial as specified in this protocol.

Temporary halt of the trial is defined as any unplanned interruption of the trial by the sponsor with the intention to resume it.

Suspension of the trial is defined as an interruption of the trial based on a Health Authority request.

The sponsor will prepare a clinical trial report within 1 year from the end of trial (6 months for paediatric trials).

The sponsor will notify the IEC/competent authority in each participating EU member state about all required trial milestones according to the respective laws.

A final report of the clinical trial data will be written only after all trial participants have completed the trial in all countries (EU or non-EU) to incorporate and consider all data in the report.

The sponsor will submit to the EU database a summary of the final trial results within 1 year from the end of a clinical trial as a whole, regardless of the country of the last trial participant (EU or non-EU).

8.7 ADMINISTRATIVE STRUCTURE OF THE TRIAL

The trial is sponsored by Boehringer Ingelheim.

A Coordinating Investigator is responsible to coordinate investigators at the different sites participating in this trial. Tasks and responsibilities are defined in a contract.

An Executive Committee consisting of independent experts and sponsor representatives will be established to support the sponsor in designing the trial and successful execution. A separate Executive Committee Charter will describe the tasks and responsibilities of the Committee.

A DMC will be established. Members of the DMC are independent of BI, they are physicians experienced in the treatment of the disease under investigation and a statistician. The DMC will assess the progress of the trial, including an unblinded safety assessment at specified intervals. The DMC will receive urgent significant safety concerns for immediate evaluation.

Proprietary confidential information © 2025 Boehringer Ingelheim International GmbH or one or more of its affiliated companies

While DMC members are unblinded, measures are in place to ensure the blinding for everyone else involved in the trial. Regular DMC meetings will be held at specified intervals. The DMC will recommend to the sponsor continuation, modification, or termination of the trial as detailed in the DMC charter. DMC recommendations as well as the final BI decision will be reported to the appropriate RAs/HAs, IRBs/IECs, and to investigators as requested by local law. The tasks and responsibilities of the DMC are specified in a DMC Charter.

Relevant documentation on the participating (Principal) Investigators (e.g. their *curricula vitae*) will be filed in the ISF.

An independent (external) and blinded CEC will be established for prospective adjudication of major adverse CV events (including non-fatal MI, non-fatal stroke, ischaemia related coronary revascularisation, and CV death), non-CV death, HF events (including hospitalisation for HF, emergency room visit, urgent care visit, or urgent outpatient HF visit), hospitalisation for unstable angina leading to coronary artery revascularisation, hospitalisation for coronary artery revascularisation, pancreatitis, and thyroid and pancreatic cancer. Composition of the CEC, its procedures, and interactions will be clarified in a CEC Charter.

The trial will be managed by a FOT vendor based on a contract which specifies the delegated responsibilities and duties. Boehringer Ingelheim has appointed a Clinical Trial Leader responsible for coordinating all required activities, in order to:

- Oversee and manage the trial in accordance with applicable regulations and SOPs
- Direct the clinical trial team and oversee the FOT vendor in the preparation, conduct, and reporting of the trial
- Ensure appropriate training

In the participating countries, the trial will be performed by a CRO based on a contract. The CRO will perform project management, clinical field monitoring, medical monitoring, reporting, and data management.

Statistical evaluation will be done by Boehringer Ingelheim according to Boehringer Ingelheim SOPs.

Tasks and functions assigned in order to organise, manage, and evaluate the trial are defined according to Boehringer Ingelheim SOPs. A list of responsible persons and relevant local information can be found in the ISF.

A central laboratory service, PK and immunogenicity laboratory services, a central ECG service, an IRT vendor, an MRI vendor,[redacted] will be used in this trial. Details will be provided in the IRT manual and central laboratory manual, available in the ISF.

Proprietary confidential information © 2025 Boehringer Ingelheim International GmbH or one or more of its affiliated companies

9. REFERENCES

9.1 PUBLISHED REFERENCES

P09-12413 Guidance for industry: drug-induced liver injury: premarketing clinical evaluation (July 2009).

P22-05567 Katsiki N, Ferrannini E, Mantzoros C. New American Diabetes Association (ADA)/European Association for the Study of Diabetes (EASD) guidelines for the pharmacotherapy of type 2 diabetes: placing them into a practicing physician's perspective. Metabolism; 2020; 107; 154218.

P22-07379 Rosenstock J, Blueher M, Schmid B, et al. Multiple dose-ranging study of the novel glucagon/GLP1 receptor dual agonist BI 456906 vs. placebo and open-label weekly semaglutide reference control in type 2 diabetes. Diabetologia; 2022; 65 (Suppl 1); Abstr 613; S314S315.

P22-08755 Rosenstock J, Blueher M, Schoelch C, et al. Glucagon/GLP1 Receptor Dual Agonist BI 456906 Reduces Body Weight in Patients with Type 2 Diabetes. Obesity (Silver Spring) 2022; 30 (Suppl 1): Abstr 30.

P23-02853 Yin X, Guo X, Liu Z, et al. Advances in the diagnosis and treatment of non-alcoholic fatty liver disease. Int J Mol Sci; 2023; 24; 2844.

P24-00984 Roux CW le, Steen O, Lucas KJ, et al. Glucagon and GLP-1_receptor dual agonist survodutide for obesity: a randomised, double-blind, placebo-controlled, dose-finding Phase II trial. Lancet Diabetes Endocrinol; 2024; 12, p. 162-173.

P24-04081 Sanyal AJ, Bedossa P, Fraessdorf M, et al. A Phase II randomized trial of survodutide in MASH and fibrosis. N Engl J Med; 2024.

R00-0292 Buysse DJ, Reynolds CF, Monk TH, et al. The Pittsburgh sleep quality_index: a new instrument for psychiatric practice and research. Psychiatry Res 1989; 28, p. 193-213.

R11-4890 Sampson HA, Muñoz-Furlong A, Campbell RL, et al. Second symposium on the definition and management of anaphylaxis: summary report - second National Institute of Allergy and Infectious Disease/Food Allergy and Anaphylaxis Network symposium. J Allergy Clin Immunol 2006; 117(2):391-397.

R12-2378 Rubin DB. Multiple imputation for nonresponse in surveys. 1987.

R16-0241 Kleiner DE, Brunt EM, Natta N van, Nonalcoholic Steatohepatitis Clinical Research Network. Design and validation of a histological scoring system for nonalcoholic fatty liver disease. Hepatology; 2005; 41(6); 1313-1321.

R17-3205 Lin ZH, Xin YN, Dong QJ, et al. Performance of the aspartate aminotransferase-to-platelet ratio index for the staging of hepatitis C-related fibrosis: an updated meta-analysis. Hepatology 2011; 53:726-36.

Proprietary confidential information © 2025 Boehringer Ingelheim International GmbH or one or more of its affiliated companies

R17-3206 Sterling RK, Lissen E, Clumeck N, et al. Development of a simple noninvasive index to predict significant fibrosis patients with HIV/HCV co-infection. Hepatology 2006; 43:13171325.

[redacted]

R18-1357 U.S. Department of Health and Human Services. Common terminology criteria for adverse events (CTCAE) version 5.0 (published 27 Nov 2017).

R20-1981 Regev A, Palmer M, Avigan MI, et al. Consensus: guidelines: best practices for detection, assessment and management of suspected acute drug-induced liver injury during clinical trials in patients with nonalcoholic steatohepatitis. Aliment Pharmacol Ther; 2019; 49; 702-713.

R20-1982 AM Sharma and RF Kushner. A proposed clinical staging system for obesity International Journal of Obesity 2009; (33):289–295.

R20-2315 Human energy requirements. Report of a joint FAO/WHO/UNU expert consultation. FAO: food and nutrition technical report.

R21-1460 Wilding JPH, Batterham RL, Calanna S, et al. STEP 1 Study Group. Once-weekly semaglutide in adults with overweight or obesity. N Engl J Med 2021; 384(11), p. 989-1002.

R21-1465 Davies M, Faerch L, Jeppesen OK, et al. STEP 2 Study Group. Semaglutide 2.4 mg once a week in adults with overweight or obesity, and type 2 diabetes (STEP 2): a randomised, double-blind, double-dummy, placebo-controlled, Phase III trial. Lancet 2021; 397, p. 971-984.

R22-0131 Saxenda (liraglutide) injection, for subcutaneous use (Novo Nordisk) (U.S. prescribing information, revised: 12/2020).

R22-2292 Riazi K, Azhari H, Charette JH, et al. The prevalence and incidence of NAFLD worldwide: a systematic review and meta-analysis. Lancet Gastroenterol Hepatol; 2022.

R22-2295 Loomba R, Friedman SL, Shulman GI. Mechanisms and disease consequences of nonalcoholic fatty liver disease. Cell; 2021; 184(10); 2537-2564.

R22-4277 American Diabetes Association Professional Practice Committee. Glycemic targets: standards of medical care in diabetes - 2022. Diabetes Care; 2022; 45(Suppl 1); S83-S96.

R23-0537 Rinella ME, Neuschwander-Tetri BA, et al. AASLD practice guidance on the clinical assessment and management of nonalcoholic fatty liver disease. Hepatology; 2023.

R23-1196 Lu FB, Hu ED, Xu LM, et al. The relationship between obesity and the severity of non-alcoholic fatty liver disease: systematic review and meta-analysis. Expert Rev Gastroenterol Hepatol; 2018; 12(5); 491-502.

Proprietary confidential information © 2025 Boehringer Ingelheim International GmbH or one or more of its affiliated companies

R23-1197 Fu CE, Ng CH, Yong JN, et al. A meta-analysis on associated risk of mortality in nonalcoholic fatty liver disease. Endocr Pract; 2023; 29; 33-39.

R23-1198 Younossi Z, Aggarwal P, Shrestha I, et al. The burden of non-alcoholic steatohepatitis: a systematic review of health-related quality of life and patient-reported outcomes. JHEP Rep; 2022; 4(9); 100525.

R23-1199 Mantovani A, Petracca G, Beatrice G, et al. Non-alcoholic fatty liver disease and risk of incident chronic kidney disease: an updated meta-analysis. Gut; 2022; 71; 156-162.

R23-1377 Younossi Z, Alkhouri N, Cusi K, et al. A practical use of noninvasive tests in clinical practice to identify high-risk patients with nonalcoholic steatohepatitis. Aliment Pharmacol Ther; 2023; 57; 304-312.

R23-1378 Zhou H, Luo P, Li P, et al. Bariatric surgery improves nonalcoholic fatty liver disease: systematic review and meta-analysis. Obes Surg; 2022; 32; 1872-1883.

R23-1379 Stine JG, Munaganuru N, Barnard A, et al. Change in MRI-PDFF and histologic response in patients with nonalcoholic steatohepatitis: a systematic review and meta-analysis. Clin Gastroenterol Hepatol; 2021; 19(11); 2274-2283.

R23-1380 Tamaki N, Munaganuru N, Jung J, et al. Clinical utility of 30% relative decline in MRI-PDFF in predicting fibrosis regression in non-alcoholic fatty liver disease. Gut; 2022; 71; 983-990.

R23-1381 Albert SG, Wood EM. Meta-analysis of trials in non-alcoholic fatty liver disease with therapeutic interventions for metabolic syndrome. Diabetes Metab Syndr; 2021; 15; 102232.

R23-1382 Vilar-Gomez E, Martinez-Perez Y, Calzadilla-Bertot L, et al. Weight loss through lifestyle modification significantly reduces features of nonalcoholic steatohepatitis. Gastroenterology; 2015; 149(2); 367-378.e5.

R23-1383 Rinella ME, Tacke F, Sanyal AJ, et al. AASLD/EASL Workshop Report on the AASLD/EASL joint workshop on clinical trial endpoints in NAFLD. Hepatology; 2019; 70(4); 1424-1436.

R23-1384 Koutoukidis DA, Koshiaris C, Henry JA, et al. The effect of the magnitude of weight loss on non-alcoholic fatty liver disease: a systematic review and meta-analysis. Metabolism; 2021; 115; 154455.

R23-1416 Quek J, Chan KE, Wong ZY, et al. Global prevalence of non-alcoholic fatty liver disease and non-alcoholic steatohepatitis in the overweight and obese population: a systematic review and meta-analysis. Lancet Gastroenterol Hepatol; 2023; 8; 20-30.

[redacted]

Proprietary confidential information © 2025 Boehringer Ingelheim International GmbH or one or more of its affiliated companies

[redacted]

R23-2419 Le P, Payne JY, Zhang L, et al. Disease State Transition Probabilities Across the Spectrum of NAFLD: A Systematic Review and Meta-Analysis of Paired Biopsy or Imaging Studies. Clin Gastroenterol Hepatol. 2023; 21 (5): 1154-1168.

[redacted]

R23-2688 Joshi GP, Abdelmalak BB, Weigel WA, et al. American Society of Anesthesiologists consensus-based guidance on preoperative management of patients (adults and children) on glucagon-like peptide-1 (GLP-1) receptor agonists. 09 Jun 2023. Available at: Website: asahq.org/about-asa/newsroom/news-releases/2023/06/american-society-of-anesthesiologists-consensus-based-guidance-on-preoperative.

R23-2769 Rinella ME, Lazarus JV, Ratziu V, et al, NAFLD Nomenclature Consensus Group. A multi-society Delphi consensus statement on new fatty liver disease nomenclature. Hepatology; 2023.

R23-3760 Newsome PN, Sasso M, Deeks JJ, et al. FibroScan-AST (FAST) score for the non-invasive identification of patients with non-alcoholic steatohepatitis with significant activity and fibrosis: a prospective derivation and global validation study. Lancet Gastroenterol Hepatol; 2020; 5(4); 362373.

R23-4361 Flint A, Andersen G, Hockings P, et al. Randomised clinical trial: semaglutide versus placebo reduced liver steatosis but not liver stiffness in subjects with non-alcoholic fatty liver disease assessed by magnetic resonance imaging. Aliment Pharmacol Ther, 2021; 54, p. 1150-1161; 2021.

R24-0245 Noureddin M, Truong E, Gornbein JA, et al. MRI-based (MAST) score accurately identifies patients with NASH and significant fibrosis. J Hepatol; 76(4), p. 781-787; 2022.

Proprietary confidential information © 2025 Boehringer Ingelheim International GmbH or one or more of its affiliated companies

[redacted]

Proprietary confidential information © 2025 Boehringer Ingelheim International GmbH or one or more of its affiliated companies

10. APPENDICES

10.1 TRIAL PARTICIPANT FEEDBACK

This trial will include an option for participants to complete questionnaires, 'Trial Participant Feedback Questionnaire', to provide feedback on their clinical trial experience. Individual participant level responses will not be reviewed by investigators. Responses will be used by the sponsor to understand where improvements can be made in the clinical trial process. These questionnaires will not collect data about the participant's disease, symptoms, treatment effect, or AEs and therefore will not be part of the trial data or CTR. The questionnaires will be implemented after local regulatory approval, if required, and after consent of the trial participant. Providing feedback is optional and not required for participation in the trial.

Proprietary confidential information © 2025 Boehringer Ingelheim International GmbH or one or more of its affiliated companies

10.2 MANAGEMENT OF GASTROINTESTINAL SYMPTOMS

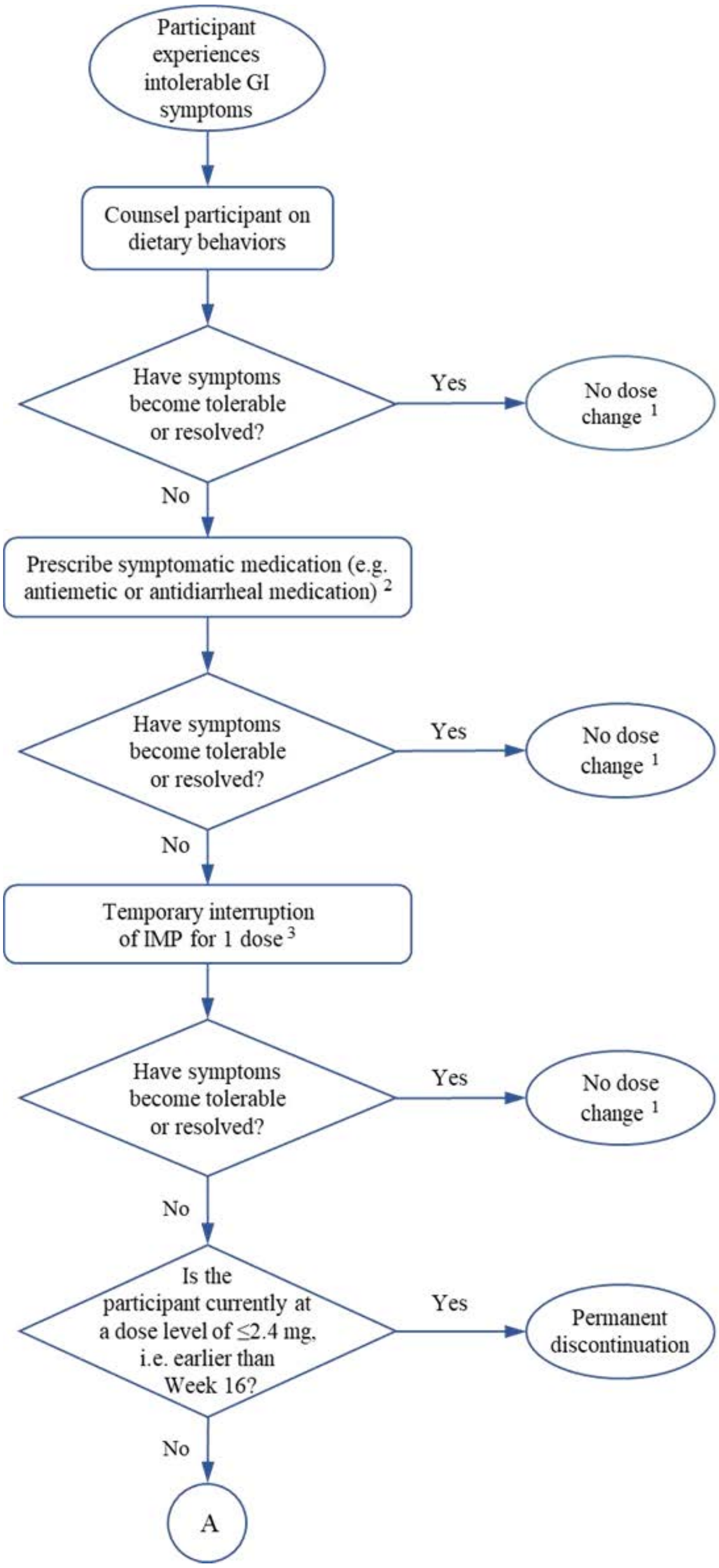

Figure 4 Management of GI symptoms

Proprietary confidential information © 2025 Boehringer Ingelheim International GmbH or one or more of its affiliated companies

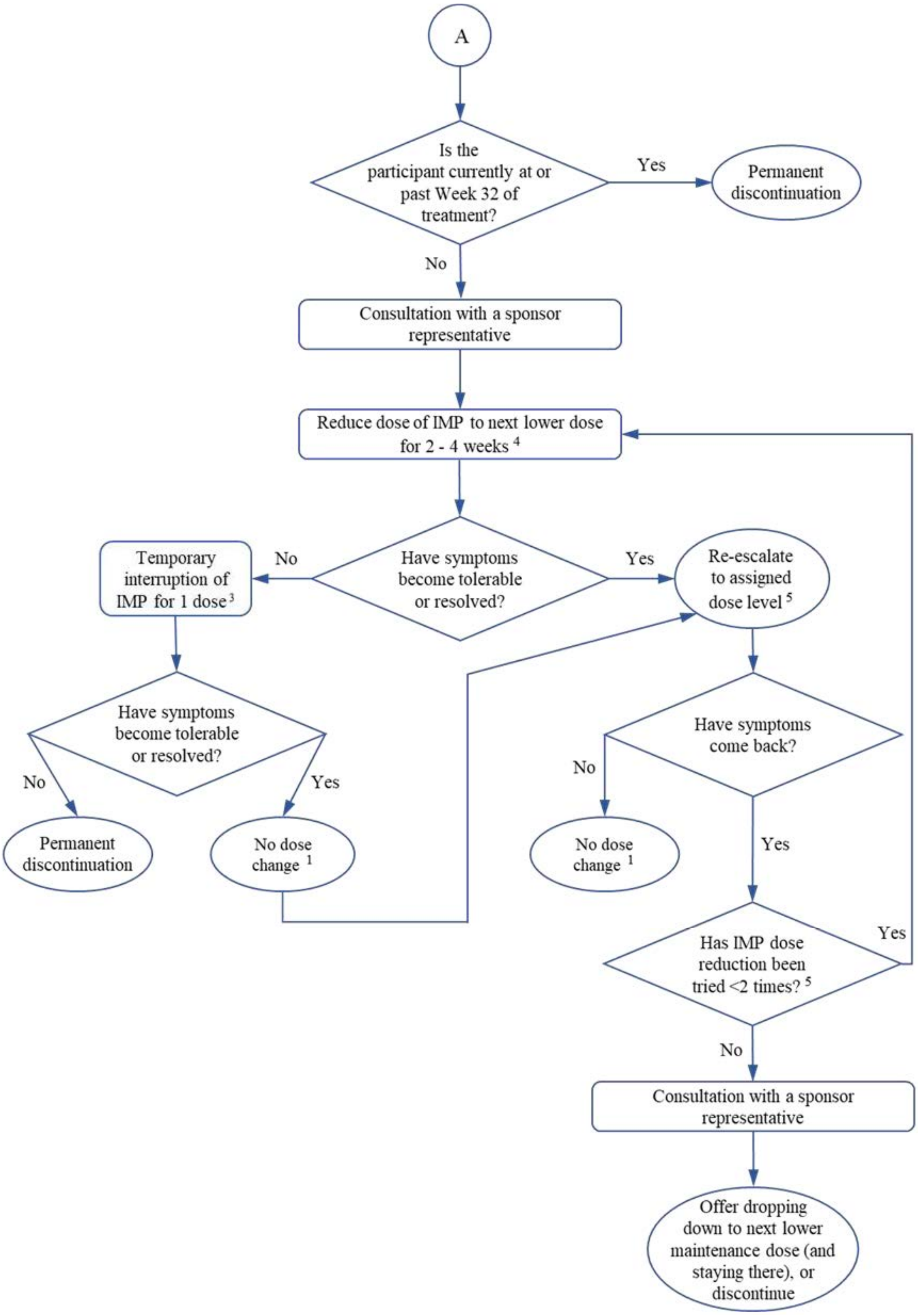

Figure 4 (cont'd) Management of GI symptoms

[1]Continue with dose escalation if in the escalation period or continue in the same dose if in the maintenance period.

[2]Symptomatic medications (e.g. antiemetic or antidiarrheal medication) can be prescribed at any time point during escalation and maintenance period as deemed necessary by investigator.

Proprietary confidential information © 2025 Boehringer Ingelheim International GmbH or one or more of its affiliated companies

[3]A temporary interruption of a second dose of the IMP may be allowed at the investigator's discretion, i.e. 2 consecutive doses are skipped.

[4] Between Weeks 16 and 32 of treatment, for participants who are not able to attain or stay at the maintenance dose of 6.0 mg due to GI symptoms, a dose reduction to 4.8 mg or 3.6 mg (or to 2.4 mg in exceptional circumstances) is possible **after consultation with a sponsor representative**. A dose reduction below 2.4 mg once per week is not allowed. A participant at the 2.4 mg dose or lower who requires a dose reduction will be discontinued from treatment. Thorough documentation of all measures taken, including outcomes of the discussion with sponsor representative, is required in a specific eCRF page in case of a dose reduction.

[5]As per investigator's discretion, it is recommended that the trial participant makes up to 2 attempts to re-escalate per dose escalation scheme to his/her assigned dose level.

Proprietary confidential information © 2025 Boehringer Ingelheim International GmbH or one or more of its affiliated companies

10.3 CONTRACEPTION METHODS ALLOWED IN THIS TRIAL

The following birth control methods (oral[1] and non-oral[2] contraceptives) may be considered as highly effective and allowed in this trial.

- Combined (oestrogen and progestogen containing) hormonal contraception associated with inhibition of ovulation
  - Oral[3]
  - Intravaginal
  - Transdermal (only for trial participants weighing less than 90 kg or 198 pounds)
- Progestogen-only hormonal contraception associated with inhibition of ovulation
  - Oral[3]
  - Injectable
  - Implantable
- Intrauterine device
- Intrauterine hormone-releasing system
- Bilateral tubal occlusion
- Vasectomised partner[4]
- Sexual abstinence[5]

[1]Oral contraceptives include combined (oestrogen and progestogen containing) or progesterone-only hormonal contraceptive pills associated with inhibition of ovulation.

[2]Non-oral contraceptives include combined hormonal contraception intravaginal or transdermal, progestogen only hormonal contraception injectable or implantable, IUD, IUS, bilateral tubal occlusion, vasectomized partner, and sexual abstinence.

[3]Women who use oral contraceptives at screening should be advised to change to non-oral contraceptives (hormonal intravaginal, transdermal, injectable or implantable) at least 7 days prior to first dose of IMP or to add a barrier method of contraception during the entire treatment period with IMP and 28 days thereafter (preferably male condom). Women who initiate oral contraceptives at or after screening should be advised to continue using the previously applied contraception method for at least 7 days and use a barrier method of contraception in addition to the oral contraceptives during the entire treatment period with IMP and 28 days thereafter (preferably male condom).

[4]Vasectomised partner is a highly effective birth control method provided that partner is the sole sexual partner of the WOCBP trial participant and there is documented absence of sperm

[5]Sexual abstinence is considered a highly effective method only if defined as refraining from heterosexual intercourse. The reliability of sexual abstinence needs to be evaluated in relation to the duration of the clinical trial and the preferred and usual lifestyle of the trial participant. Periodic abstinence e.g. calendar, ovulation, symptothermal, post-ovulation methods; declaration of abstinence for the duration of exposure to IMP; and withdrawal are not acceptable.

Proprietary confidential information © 2025 Boehringer Ingelheim International GmbH or one or more of its affiliated companies

10.4 TIME SCHEDULE FOR PHARMACOKINETIC (PK) AND ANTI-DRUG ANTIBODY (ADA) / NEUTRALISING ANTIBODY (NAB) BLOOD SAMPLING

Table 14 Time Schedule for PK and ADA/NAb Sampling

Visit	Day	Time Point [hh:min]	PK Sampling	ADA/NAb Sampling
2	1 (+/-0)	- 00:30<br>Approximately 30 min before IMP administration	X	X
4	29 (+/-3)		X	X
6	57 (+/-3)		X	X
7	85 (+/-3)		X	X
10	169 (+/-3)		X	X
13	253 (+/-7)		X	X
15/EOT	337 (+/-7)	168:00<br>Approximately 168 h (1 week) after last IMP administration	X	X
16/EOT[1]	365 (+/-7)	168:00<br>Approximately 168 h (1 week) after last IMP administration	X	X
ETD	-	168:00<br>Approximately 168 h (1 week) after last IMP administration	X	X
Follow Up	358 (+7)	672:00<br>Approximately 672 h (4 weeks) after last IMP administration	X	X

[1]Only applicable for participants who agreed to enter the optional extended treatment period

Proprietary confidential information © 2025 Boehringer Ingelheim International GmbH or one or more of its affiliated companies

10.5 CLINICAL STAGING OF OBESITY

Staging of obesity will be assessed by the investigator using the following staging system based on the medical history at screening.

Edmonton obesity staging system

Stage	Short description
**Stage 0**	**No apparent risk factors**
**Stage 1**	**Subclinical risk factors**: Presence of obesity-related subclinical risk factors (e.g. borderline hypertension, impaired fasting glucose, elevated liver enzymes, etc.), mild physical symptoms (e.g. dyspnoea on moderate exertion, occasional aches and pains, fatigue, etc.), mild psychopathology, mild functional limitations and/or mild impairment of well being
**Stage 2**	**Established complications**: Presence of established obesity-related chronic disease (e.g. hypertension, T2DM, sleep apnoea, osteoarthritis, reflux disease, polycystic ovary syndrome, anxiety disorder, etc.), moderate limitations in activities of daily living and/or well being
**Stage 3**	**End organ damage:** Presence of end organ damage such as MI, HF, diabetic complications, incapacitating osteoarthritis, significant psychopathology, significant functional limitations and/or impairment of well being
**Stage 4**	**End stage**: Having disabilities from obesity-related chronic diseases, severe disabling psychopathology, severe functional limitations and/or severe impairment of well being

Proprietary confidential information © 2025 Boehringer Ingelheim International GmbH or one or more of its affiliated companies

11. DESCRIPTION OF GLOBAL AMENDMENT(S)

11.1 GLOBAL AMENDMENT 1

This amendment is considered to be substantial based on the criteria set forth in Regulation (EU) No 536/2014 of the European Parliament and the Council of the European Union.

Overall rationale for the amendment:

Request from Health Authority

**Date of amendment**		20 May 2024
**EU CT number**<br>**Universal Trial number**		2023-505303-23-00<br>U1111-1299-9925
**BI Trial Number**		1404-0056
**BI Investigational Medicinal Product(s)**		Survodutide (BI 456906)
**Title of protocol**		Multicentre, randomised, double-blind, placebo-controlled, 48-week, Phase III trial to evaluate the efficacy and safety of survodutide administered subcutaneously in participants with overweight or obesity and presumed or confirmed non-alcoholic steatohepatitis (NASH)
**Global Amendment due to urgent safety reasons**		☐
**Global Amendment**		☒
**Section to be changed**		Synopsis
**Description of change**		1. Addition of "History of at least one self-reported unsuccessful dietary effort to lose body weight" to key inclusion criteria<br>2. Clarification that the primary objective will be addressed by the treatment-regimen estimand and that the efficacy estimand is included for supplementary analyses
**Rationale for change**		Ad 1 and 2: Request from Health Authority
**Section to be changed**		Schedule of assessments
**Description of change**		Footnote 14: Clarification that MRI scheduled prior to Visit 2 will be performed to measure MRI-PDFF, cT1 and MRE
**Rationale for change**		Request from Health Authority
**Section to be changed**		Section 3.3.2

Proprietary confidential information © 2025 Boehringer Ingelheim International GmbH or one or more of its affiliated companies

**Date of amendment**		20 May 2024
**Description of change**		History of at least one self-reported unsuccessful dietary effort to lose body weight was added to inclusion criteria
**Rationale for change**		Request from Health Authority
**Section to be changed**		Section 4.1.5.1
**Description of change**		Addition of ADA/Nab samples to the text regarding the cases in which randomisation codes will be provided to the external bioanalytical laboratory during the trial
**Rationale for change**		Request from Health Authority
**Section to be changed**		Section 4.2.2.2
**Description of change**		1. Recommendation that participants abstain from alcohol consumption during the trial was added<br>2. Addition of "for dietary and physical activity counselling" when referring to Section 4.2.1.2.
**Rationale for change**		Ad 1 and 2: Request from Health Authority
**Section to be changed**		Section 6.2.1
**Description of change**		Clarification that MRI that should be performed in case that VCTE <20 kPa will measure MRI-PDFF, cT1 and MRE
**Rationale for change**		Request from Health Authority
**Section to be changed**		Section 7.2.2
**Description of change**		Clarification that the primary efficacy analysis will be conducted using treatment-regimen estimand was added
**Rationale for change**		Request from Health Authority
**Section to be changed**		Section 7.2.3.1
**Description of change**		1. Clarification was added that the analysis of the occurrence of "Relative reduction in liver fat content of at least 30% from baseline to Week 48 assessed by MRI-PDFF (yes/no)" will include calculation and testing of odds ratio<br>2. Text related to analysis relative to the efficacy estimand was moved to Section 7.2.3.4<br>3. Clarification that further details will be provided in the TSAP and the TSAP will be finalised prior to DBL was added

Proprietary confidential information © 2025 Boehringer Ingelheim International GmbH or one or more of its affiliated companies

**Date of amendment**		20 May 2024
**Rationale for change**		Ad 1, 2, and 3: Request from Health Authority
**Section to be changed**		Section 7.2.3.4
**Description of change**		1. Text related to the efficacy estimand was moved here from Section 7.2.3.1<br>2. Clarification was made that significance tests will be based on contrasts at the primary endpoint time point (Week 48).<br>3. Explanation that unstructured covariance will be assumed to model the relationship between pairs of measurements taken at different weeks on the same patient was added
**Rationale for change**		Ad 1,2, and 3: Request from Health Authority

Proprietary confidential information © 2025 Boehringer Ingelheim International GmbH or one or more of its affiliated companies

11.2 GLOBAL AMENDMENT 2

This amendment is considered to be substantial based on the criteria set forth in Regulation (EU) No 536/2014 of the European Parliament and the Council of the European Union.

Overall rationale for the amendment:

Alignment with other Phase III studies

**Date of amendment**	22 July 2024	
**EU CT number**<br>**Universal Trial number**	2023-505303-23-00<br>U1111-1299-9925	
**BI Trial Number**	1404-0056	
**BI Investigational Medicinal Product(s)**	Survodutide (BI 456906)	
**Title of protocol**	Multicentre, randomised, double-blind, placebo-controlled, Phase III trial to evaluate the efficacy and safety of survodutide administered subcutaneously in participants with overweight or obesity and presumed or confirmed non-alcoholic steatohepatitis (NASH)	
**Global Amendment due to urgent safety reasons**		☐
**Global Amendment**		☒
**Section to be changed**	Synopsis, Section 7.2.3.1	
**Description of change**	Added clarification that the responder/non-responder (yes/no) status will be based on the continuous liver fat measurements, or, in the case of missing liver fat values, on the imputed continuous values	
**Rationale for change**	To clarify the methods of imputation of missing continuous values of MRI-PDFF	
**Section to be changed**	Synopsis, Section 3.1	
**Description of change**	The number of participants was changed to at least 104 in survodutide group and at least 52 in placebo group	
**Rationale for change**	Correction	
**Section to be changed**	Synopsis, Section 3.3.3	
**Description of change**	Added clarification of requirements for a negative HCV RNA test	
**Rationale for change**	To clarify that HCV RNA should be also performed for patients with positive HCV antibody but who were not treated	

Proprietary confidential information © 2025 Boehringer Ingelheim International GmbH or one or more of its affiliated companies

**Section to be changed**	Synopsis, Section 3.3.3
**Description of change**	History of cirrhosis added to key exclusion criteria
**Rationale for change**	To clarify that patients with the history of cirrhosis (e.g. patients that previously had liver fibrosis stage F4 that improved to F3) are to be excluded
**Section to be changed**	SoA, Section 3.1
**Description of change**	A column for ETD assessments was added, and the relevant footnotes were updated
**Rationale for change**	To clarify that for the patients that discontinued the IMP earlier there would be an ETD visit, and to specify the assessments performed at the ETD visit
**Section to be changed**	SoA
**Description of change**	A row for substance usage was added, and footnote 5 was updated
**Rationale for change**	To clarify that data on substance usage is to be collected on V2, V10 and EOT/ETD
**Section to be changed**	SoA, Section 5.2.3, Section 5.4.1
**Description of change**	A row for Hs Troponin T was added, Changed Hs Troponin T from biomarker to safety evaluation; at V2, V7, V11, and EOT/ETD troponin T will be measured always and not as reflex in case of elevated CK. Where missing, information was added to clarify that troponin test is Hs troponin T.
**Rationale for change**	To guarantee the timely report of Hs Troponin T values at the specified timepoints.
[redacted]	
**Section to be changed**	SoA
**Description of change**	A specification of parameters to be measured as part of the lipid tests was added
**Rationale for change**	To improve clarity
**Section to be changed**	SoA
**Description of change**	Row for ELF score was added
**Rationale for change**	Correction, the ELF score should be calculated at screening
**Section to be changed**	SoA

Proprietary confidential information © 2025 Boehringer Ingelheim International GmbH or one or more of its affiliated companies

**Description of change**	[redacted]
**Rationale for change**	
**Section to be changed**	SoA
**Description of change**	Clarification was added to Footnote 2 that a separate informed consent is required for biobanking samples [redacted]
**Rationale for change**	Clarification
**Section to be changed**	SoA
**Description of change**	Clarification was added to Footnote 6 that the eye examination is to be performed in patients with diabetes
**Rationale for change**	Clarification
**Section to be changed**	SoA, Section 5.2.4
**Description of change**	Clarification was added to Footnote 8 that ECG screening should be recorded in triplicates with interval of 180 s at the screening
**Rationale for change**	Clarification to specify the timing of measurements
**Section to be changed**	SoA, footnotes 10 and 11
**Description of change**	Edited text on the timing of EOT, ETD, FU and EOS visits. Replaced extensive description on options and assessments for participants discontinuing IMP prematurely with a cross-reference to applicable sections.
**Rationale for change**	To clarify the timing of newly added ETD visit and to decrease redundancy
**Section to be changed**	SoA (footnote 14), Section 5.1.1
**Description of change**	Clarification that prior to MRE measurements 4 h of fasting are required was added
**Rationale for change**	To avoid confusion with other assessments that require 3 h of fasting
**Section to be changed**	SoA, footnote 15
**Description of change**	Text edited
**Rationale for change**	To clarify that CAP™ is optional
**Section to be changed**	SoA, footnote 20
**Description of change**	Clarification was added that biomarker samples are not to be collected after IMP discontinuation

Proprietary confidential information © 2025 Boehringer Ingelheim International GmbH or one or more of its affiliated companies

**Rationale for change**	Clarification
**Section to be changed**	SoA, footnotes 19 and 20
**Description of change**	The footnote was updated to clarify that the schedule of assessments should be followed even if one sample was omitted
**Rationale for change**	Clarification
**Section to be changed**	SoA, footnote 21
**Description of change**	Clarification was added that DNA samples will be collected prior to IMP administration
**Rationale for change**	Clarification
**Section to be changed**	SoA (footnote 23), Section 5.2.3. (Table 10)
**Description of change**	Removed FSH testing requirement for women >55 years old with amenorrhea for at least 12 months without alternative medical cause other than being postmenopausal. Requirement to pause HRT to allow FSH testing.
**Rationale for change**	To avoid unnecessary assessments
**Section to be changed**	SoA (footnote 25)
**Description of change**	Text regarding the participation of patients with type 4 or 5 suicidal ideation was removed and reference to Sections 3.3.4.1 and 5.2.5.1 was provided instead
**Rationale for change**	Correction: for participants that answered "yes" to specific C-SSRS questions, the IMP should be discontinued regardless of the investigator's opinion. Reference was provided to the sections that state this
**Section to be changed**	Section 1 Introduction
**Description of change**	A footnote was added that for the purpose of this trial the terms NAFLD, NAFL and NASH are to be considered interchangeable to MASLD, MASL and MASH, respectively.
**Rationale for change**	Clarification, as new terms are now used in clinical practice and to avoid changing the title of the trial.
**Section to be changed**	Section 1.2 Drug profile
**Description of change**	References were updated
**Rationale for change**	To reflect recent publications
**Section to be changed**	Section 1.4.2 Benefit-Risk Assessment
**Description of change**	The mitigation strategy for the risks of acute pancreatitis, pancreatic cancer, and Medullary Thyroid Carcinoma (MTC) now includes the exclusion of certain patients. This is in line

Proprietary confidential information © 2025 Boehringer Ingelheim International GmbH or one or more of its affiliated companies

	with the exclusion criteria that were already present in the previous version of the CTP
**Rationale for change**	Completeness
**Section to be changed**	Section 1.4.2 Benefit-Risk Assessment
**Description of change**	Updated safety profile on GI disorders and cardiac disorders
**Rationale for change**	New information available from clinical trials 1404-0010 and 1404-0043
**Section to be changed**	Section 1.4.2 Benefit-Risk Assessment
**Description of change**	In acute kidney injury (pre-renal) due to dehydration risk, diarrhoea was added as an example of one of the possible causes of dehydration. Mitigation strategy was updated
**Rationale for change**	New information available from clinical trials 1404-0010 and 1404-0043
**Section to be changed**	Section 1.4.3 Discussion
**Description of change**	Survodutide ADRs such as nausea, vomiting, diarrhoea, constipation, abdominal pain, dyspepsia, and dizziness were removed from the section.
**Rationale for change**	New ADRs may be added during ongoing development of survodutide. The current ADRs of survodutide are listed in the IB.
**Section to be changed**	Section 2.1.3 Secondary Endpoints
**Description of change**	Absolute/relative change from baseline to Week 48 in liver fat content assessed by MRI-PDF was split to 2 endpoints
**Rationale for change**	To comply with guidelines
**Section to be changed**	Section 2.1.3 Secondary Endpoints
**Description of change**	Where missing, measurement units were specified
**Rationale for change**	Correction
**Section to be changed**	[redacted]
**Description of change**	
**Rationale for change**	
**Section to be changed**	
**Description of change**	
**Rationale for change**	
**Section to be changed**	

Proprietary confidential information © 2025 Boehringer Ingelheim International GmbH or one or more of its affiliated companies

**Description of change**	[redacted]
**Rationale for change**	
**Section to be changed**	
**Description of change**	
**Rationale for change**	
**Section to be changed**	
**Description of change**	
**Rationale for change**	
**Section to be changed**	
**Description of change**	
**Rationale for change**	
**Section to be changed**	
**Description of change**	
**Rationale for change**	
**Section to be changed**	Section 3.1 Trial Design
**Description of change**	Body weight was added to necessary measurements to be performed at Week 48
**Rationale for change**	Alignment across several NASH trials
**Section to be changed**	Section 3.3.2 Inclusion Criteria, Section 3.3.3 Exclusion Criteria
**Description of change**	Addition: "For an eligible participant, all inclusion criteria must be answered “yes” (Section 3.3.2)/"no"(Section 3.3.3) at screening and re-confirmed at baseline (i.e. before randomisation), except for laboratory tests for which screening results are used for randomisation"
**Rationale for change**	Clarification
**Section to be changed**	Section 3.3.2 Inclusion Criteria
**Description of change**	Correction of the MAST score formula
**Rationale for change**	Correction
**Section to be changed**	Section 3.3.3 Exclusion Criteria

Proprietary confidential information © 2025 Boehringer Ingelheim International GmbH or one or more of its affiliated companies

**Description of change**	Clarification that PHQ9 assessment may be repeated once within the screening window
**Rationale for change**	To clarify how the exclusion of patients with PHQ9 ≥15 during screening period may be achieved
**Section to be changed**	Section 3.3.3 Exclusion Criteria
**Description of change**	Removed the acceptance to include participants with a history of other malignancies treated with curative intent.
**Rationale for change**	Clarify exclusion to be applicable to malignancy with reasonably recent biological activity.
**Section to be changed**	Section 3.3.3 Exclusion Criteria
**Description of change**	Addition of any condition that may interfere with trial objectives
**Rationale for change**	Increase in stringency of exclusion criteria
**Section to be changed**	Section 3.3.3 Exclusion Criteria
**Description of change**	Clarification on what “screening period” means (in mental health assessments) was added
**Rationale for change**	Clarification
**Section to be changed**	Section 3.3.4.1 Discontinuation of trial treatment
**Description of change**	Clarification that if changes in **mean** QTcF are seen in local reading, IMP should be stopped
**Rationale for change**	To clarify the discontinuation criteria for QTcF interval prolongation
**Section to be changed**	Section 3.3.4.1 Discontinuation of trial treatment
**Description of change**	Detailed clarification was added regarding the timing of ETD and FU for the patients that discontinued the treatment early, and the scenarios in which ETD/FU may be combined with other trial visits
**Rationale for change**	Clarify and reduce patient burden
**Section to be changed**	Section 3.3.4.1 Discontinuation of trial treatment
**Description of change**	Clarification on which type of data should be collected at minimum during remote visits/telephone calls/Week 51
**Rationale for change**	Clarification
**Section to be changed**	Section 3.3.4.1 Discontinuation of trial treatment
**Description of change**	Clarification regarding obtaining MRI-PDFF data from patients that discontinued early

Proprietary confidential information © 2025 Boehringer Ingelheim International GmbH or one or more of its affiliated companies

**Rationale for change**		Clarification to assist data collection for primary data analysis
**Section to be changed**		Section 3.3.4.1 Discontinuation of trial treatment
**Description of change**		Added and modified different follow-up modalities for participants discontinuing the IMP prematurely
**Rationale for change**		Allow flexibility for participants willing to undergo further assessments after IMP discontinuation prematurely.
**Section to be changed**		Section 3.3.4.1 Discontinuation of trial treatment
**Description of change**		Clarification that Boehringer will pause and potentially stop the trial if hepatic injury is found in 3 or more patients
**Rationale for change**		Health Authority request
**Section to be changed**		Section 3.3.4.2
**Description of change**		Added clarification on the meaning of consent withdrawal and that the investigator must explain the meaning to trial participants
**Rationale for change**		Clarification
**Section to be changed**		Section 4.1.4 Drug Assignment and Administration
**Description of change**		Specification of the maintenance dose (6.0 mg) from which the participants may downgrade to a lower dose was added, Clarification that if measures to mitigate tolerability issues did not work, a reduction of dose (or delay in dose escalation), with the intent to resume escalation, is possible (exception: reduction or delay not allowed for participants on 2.4 mg dose).
**Rationale for change**		Clarification
**Section to be changed**		Section 4.1.4 Drug Assignment and Administration, Section 4.1.4.1
**Description of change**		Instructions regarding the use of spare IMP kits were added. Clarification was added that for any changes in dose outside of the regularly scheduled steps, IMP of the new dose is dispensed during an unscheduled visit, and the participant must return all kits of the old dose. Instructions were added regarding the documentation of the dose reduction.
**Rationale for change**		To ensure the correct use of the kits. To ensure proper documentation of the dose reduction
**Section to be changed**		Section 4.1.4.1
**Description of change**		Replaced “should” with “must” in the length description of the entire dose escalation period

Proprietary confidential information © 2025 Boehringer Ingelheim International GmbH or one or more of its affiliated companies

**Rationale for change**		To stress the importance of keeping a fixed length of dose escalation period
**Section to be changed**		Section 4.2.1.1.3
**Description of change**		Plasma glucose values that should be reported were corrected
**Rationale for change**		Correction
**Section to be changed**		Section 4.2.1.2
**Description of change**		Clarification on assessment of dietary compliance was added
**Rationale for change**		Clarification for the sites
**Section to be changed**		Section 4.2.2 Restrictions
**Description of change**		Text added clarifying that oral medications with an NTI should not be administered until at least 28 days after last dose of IMP. Replaced the term survodutide with IMP when referring to the restrictions during their treatment period.
**Rationale for change**		Improve clarity. Corrected error (as blinding will not allow to distinguish between IMP and survodutide treatment).
**Section to be changed**		Section 4.2.2.1
**Description of change**		Clarification of the restrictions regarding Vitamin E treatment was added
**Rationale for change**		To clarify that 400 UI of Vitamin E per day is allowed if was taken at a stable dose for more than 6 months
**Section to be changed**		Section 4.2.2.1
**Description of change**		Addition of the ursodeoxycholic acid to the list of restricted medications
**Rationale for change**		Addition
**Section to be changed**		Section 4.2.2.1
**Description of change**		Text regarding restricted anti-obesity treatments was amended
**Rationale for change**		Clarification
**Section to be changed**		Section 4.2.2.1
**Description of change**		Footnote that the list of medications in Table 8 is not exhaustive was added, and mirtazapine removed from SSRI list
**Rationale for change**		Correction
**Section to be changed**		Section 5.1.2 Fibroscan

Proprietary confidential information © 2025 Boehringer Ingelheim International GmbH or one or more of its affiliated companies

**Description of change**	Clarification regarding the use of SmartExam during FibroScan(R) measurements and the need for 3 h of fasting was added
**Rationale for change**	Clarification
**Section to be changed**	Section 5.2.6.1.4 AESI
**Description of change**	Clarification that liver enzymes should be retested in case of potential DILI, and IMP should be interrupted if ALT remains elevated was added, DILI was added to events requiring adjudication
**Rationale for change**	To ensure participants' safety
**Section to be changed**	Section 5.2.6.1.4 AESI, Table 11 and Table 12
**Description of change**	Rash and eosinophilia were added to liver-related symptoms (second row, Table 11), and glucose levels for hypoglycaemia Level 1 were amended (Table 12)
**Rationale for change**	Correction
**Section to be changed**	Section 5.2.6.1.7 AESI
**Description of change**	Edited the text on acute pancreatitis, including suspicion of pancreatitis as a criterion to perform CT scan or MRI. Edited text allowing MRI with contrast as an alternative to CT scan with contrast.
**Rationale for change**	To clarify that CT scan needs to be performed only when there is suspicion of pancreatitis and not for enzyme elevations of amylase or lipase >3x ULN
**Section to be changed**	Section 5.2.6.1.7
**Description of change**	Added management of acute severe renal events by hydration and information on diagnosis and staging based on KDIGO classification of AKI
**Rationale for change**	Health Authority Request
**Section to be changed**	Section 5.2.6.1.7
**Description of change**	Pancreatitis suspicion was added as a requirement to perform CT scan or MRI in case of elevated amylase/lipase >3x ULN
**Rationale for change**	Performing CT scan/MRI for all patients with elevated amylase/lipase >3x ULN without symptoms of pancreatitis deemed not necessarily
**Section to be changed**	[redacted]
**Description of change**	
**Rationale for change**	

Proprietary confidential information © 2025 Boehringer Ingelheim International GmbH or one or more of its affiliated companies

**Section to be changed**	Section 5.6.1.1 Timing of immunogenicity measures
**Description of change**	Text added regarding ADA and NAb assessment in case of systematic hypersensitivity
**Rationale for change**	Addition
**Section to be changed**	Section 6.2.1 Screening Period
**Description of change**	MAST, FAST, and ELF score were added to screening
**Rationale for change**	Needed for the inclusion criteria
**Section to be changed**	Section 6.2.2 Treatment Period
**Description of change**	[redacted]
**Rationale for change**	Addition
**Section to be changed**	Section 6.2.2 Treatment Period
**Description of change**	Order of sequence of assessments was changed and clarification regarding which assessment requires fasted state was added
**Rationale for change**	To increase clarity and reduce patient burden
**Section to be changed**	Section 7.1 Null and Alternative Hypotheses
**Description of change**	Correction of a typo in H02 formula
**Rationale for change**	Correction
**Section to be changed**	Section 7.2.2
**Description of change**	The definition of null hypothesis for both estimands was amended
**Rationale for change**	Correction
**Section to be changed**	Section 7.2.3.1
**Description of change**	Text regarding analysis of treatment-regimen estimand was amended
**Rationale for change**	To specify the primary endpoint
**Section to be changed**	Section 7.2.3.4
**Description of change**	Text added regarding covariate-adjusted estimator
**Rationale for change**	Request from Health Authorities
**Section to be changed**	Section 10.1 Appendix
**Description of change**	Section on trial participant feedback was added
**Rationale for change**	New addition from the sponsor

Proprietary confidential information © 2025 Boehringer Ingelheim International GmbH or one or more of its affiliated companies

11.3 GLOBAL AMENDMENT 3

This amendment is considered to be substantial based on the criteria set forth in Regulation (EU) No 536/2014 of the European Parliament and the Council of the European Union.

Overall rationale for the amendment:

Alignment with other Phase III studies

Date of amendment	24 Jan 2025
EU CT number<br>Universal Trial number	2023-505303-23-00<br>U1111-1299-9925
BI Trial Number	1404-0056
BI Investigational Medicinal Product(s)	Survodutide (BI 456906)
Title of protocol	Multicentre, randomised, double-blind, placebo-controlled, 48-week, Phase III trial to evaluate the efficacy and safety of survodutide administered subcutaneously in participants with overweight or obesity and presumed or confirmed non-alcoholic steatohepatitis (NASH)
Global Amendment due to urgent safety reasons	☐
Global Amendment	☒
Section to be changed	Title page, Synopsis, Section 3.1, Section 11.2
Description of change	Length of the trial (48 weeks) deleted
Rationale for change	To reflect an optional extended treatment duration of 4 weeks that changes the trial length to 52 weeks in some participants
Section to be changed	Synopsis, Section 7.2.3.1
Description of change	"Continuous liver fat" was deleted from items to be imputed based on "retrieved dropouts". It was clarified that fixed categorical effects refer to baseline, and text on MMRM was re-arranged. Missing values will be imputed using predicted values (instead of imputed).
Rationale for change	Clarification and correction of a mistake
Section to be changed	Synopsis, Section 2.2.2, Section 3.3.2, Section 5.2.3
Description of change	Edited text to clarify HDL, LDL, and VLDL refers to HDL, LDL, and VLDL cholesterol
Rationale for change	Clarification and consistency throughout the protocol.
Section to be changed	Synopsis, Section 3.1

Proprietary confidential information © 2025 Boehringer Ingelheim International GmbH or one or more of its affiliated companies

Description of change		Clarification was added that the final number of randomised participants was 220 instead of planned 160.
Rationale for change		To explain why the number of participants in the trial is higher than planned
Section to be changed		Synopsis, Section 3.1, Section 4.1.4, Section 6.1, Section 6.2.2
Description of change		Option to extend treatment period by 4 weeks was added to the trial design and duration of treatment. Timepoints of procedures during optional extended treatment period are added to the SoA and clarified in Section 3.1. Footnote added to Figure 2
Rationale for change		To provide safety and tolerability assessments for up to 1 year (52 weeks)
Section to be changed		SoA, Section 3.1
Description of change		A column for "Extended treatment period (ETP)" was added. A timepoint at EOT was added for IRT call and dispense IMP, with clarification in the footnote 35 that this is only applicable for participants in extended treatment period. Footnote 36 was added to clarify ETP. Footnote 10 was updated.
Rationale for change		To introduce the procedures during ETP.
Section to be changed		SoA, Section 10.4
Description of change		Timepoint for sampling for PK, ADA, and NAb at V16/EOT was added, instructions added to Table 14
Rationale for change		To clarify PKA, ADA and NAb sampling procedures at ETP
Section to be changed		SoA
Description of change		Footnote 6 was updated to clarify that eye examination can be performed up to 14 days after EOT/ETD visit
Rationale for change		To decrease burden to the sites as in USA it is difficult to perform an eye examination on the same day as EOT
Section to be changed		SoA
Description of change		Note with clarification on in-clinic/remote visits was added
Rationale for change		Clarification
Section to be changed		SoA, footnote 3
Description of change		Amended
Rationale for change		Clarification
Section to be changed		SoA, footnote 5

Proprietary confidential information © 2025 Boehringer Ingelheim International GmbH or one or more of its affiliated companies

Description of change	Shortened
Rationale for change	Increase readability
Section to be changed	SoA, footnotes 14 and 15
Description of change	Footnotes 14 and 15 were expanded to add the clarification that liver assessments at FU visit will be performed for patients that discontinued the treatment early
Rationale for change	Clarification
Section to be changed	SoA, footnote 21
Description of change	Shortened
Rationale for change	Increase readability
Section to be changed	SoA, footnote 23
Description of change	Clarification on pausing HRT was added
Rationale for change	Clarification
Section to be changed	SoA, footnote 26
Description of change	Replaced "are" with "may" in the sentence “The remote visits (if allowed per local regulations) may be performed”
Rationale for change	Alignment with the rest of the protocol
Section to be changed	Section 1.4.2
Description of change	Amylase changed to serum pancreatic amylase
Rationale for change	Clarification
Section to be changed	Section 1.4.3
Description of change	A cross reference to the latest version of the IB is added and examples of known ADRs were listed.
Rationale for change	New ADRs may be added during the ongoing development of survodutide. The updated list of ADRs for survodutide is provided in the current version of the IB
Section to be changed	[redacted]
Description of change	
Rationale for change	
Section to be changed	
Description of change	

Proprietary confidential information © 2025 Boehringer Ingelheim International GmbH or one or more of its affiliated companies

Rationale for change	[redacted]
Section to be changed	
Description of change	
Rationale for change	
Section to be changed	Section 3.3.4.1
Description of change	If the specified QTcF cut-offs, which meet discontinuation criteria, are observed in a local ECG reading the IMP should be temporarily interrupted. If these findings are subsequently confirmed by a central ECG reading, the IMP should be discontinued permanently
Rationale for change	To clarify the discontinuation criteria for QTcF interval prolongation
Section to be changed	Section 3.3.4.1
Description of change	Clarification was added to discontinuation in case of QTcF prolongation, broad QRS complex, and additional scenarios
Rationale for change	To clarify that in conditions with a broad QRS complex such as intermittent bundle branch block or intermittent interventricular pacing, any potential QTcF prolongation >60 ms from baseline should be evaluated by the Investigator and after consultation with a cardiologist to determine if it fulfills discontinuation criteria
Section to be changed	Section 3.3.4.1
Description of change	Clarification regarding diabetic rethinopathy/maculopathy was added
Rationale for change	To clarify that if a worsening or new onset of diabetic retinopathy or maculopathy is considered clinically significant by the investigator, it will lead to treatment discontinuation
Section to be changed	Section 3.3.4.1
Description of change	Text regarding diagnosis of any malignancy was amended
Rationale for change	To clarify that cancers in situ do not lead to discontinuation of treatment and that cancers that can be considered cured after surgical resection, such as prostate cancer, should be discussed with the sponsor
Section to be changed	Section 3.3.4.1, Section 4.1.5.2

Proprietary confidential information © 2025 Boehringer Ingelheim International GmbH or one or more of its affiliated companies

Description of change	Addition of discontinuation criterion: "emergency unblinding of participant´s treatment assignment by the investigator"
Rationale for change	Clarification on wording of safety-related unblinding: participants cannot continue IMP treatment but can stay in the trial
Section to be changed	Section 3.3.4.1
Description of change	Added descriptive information on cross reference to Section 5.2.5.1
Rationale for change	Clarification
Section to be changed	Section 3.3.4.1
Description of change	Discontinuation criterion "worsening or new onset of diabetic retinopathy or maculopathy" added
Rationale for change	Rapid improvement in HbA1c can lead to worsening of diabetic retinopathy, which is a class risk for GLP1RAs. Patients with worsening of pre-existing diabetic retinopathy should be discontinued to prevent further exacerbation and complications of the event. New onset is also considered a worsening in this context.
Section to be changed	Section 3.3.4.1
Description of change	Description of procedures following early treatment discontinuation was reviewed and shortened. Details on follow-up for participants who chose Option 3 or 4 were added
Rationale for change	Clarification
Section to be changed	Section 3.3.4.2
Description of change	The text was revised to more clearly describe the procedures to be followed if a participant withdraws consent for trial participation or treatment with IMP
Rationale for change	To clarify procedures required in the event of withdrawal of consent and to ensure consistency with clarifications in footnotes to the SoA
Section to be changed	Section 4.1.4
Description of change	Revised text to note that participants may receive extra kits as a back-up
Rationale for change	Improve clarity regarding procedures to be followed during the in-clinic visits
Section to be changed	Section 4.1.4
Description of change	Change of the text for dose-reduction being allowed only after consultation with the sponsor. Investigators should

Proprietary confidential information © 2025 Boehringer Ingelheim International GmbH or one or more of its affiliated companies

	consult with the sponsor representative for any IMP dose adjustments
Rationale for change	To be consistent with how IMP dose adjustments are managed by investigators in consultation with the sponsor
Section to be changed	Section 4.1.4
Description of change	Visit numbers were added to table 4
Rationale for change	To increase clarity
Section to be changed	Section 4.1.4
Description of change	In Table 6 allowed to resume treatment after missing 3 consecutive doses of IMP without contacting the sponsor
Rationale for change	To reduce the burden on sites and avoid further delays in dosing
Section to be changed	Section 4.1.4
Description of change	Added the possibility for assessments being performed remotely by a specialized vendor/HCP, if agreed with the sponsor, if available and if acceptable by local law and regulation.
Rationale for change	To allow flexibility for collecting these assessments remotely
Section to be changed	Section 4.1.5.2
Description of change	Clarification was added that once unblinded, the trial treatment needs to be discontinued for the participant. However, the participant should remain in the trial if willing to continue follow up
Rationale for change	Clarification
Section to be changed	Section 4.1.8
Description of change	Revised text to note that sharps container with used syringes should be returned to the site whenever a container replacement is needed and latest at EOT visit.
Rationale for change	To reduce operational burden
Section to be changed	Section 4.2.1.2
Description of change	Text regarding food diary completion requirements was amended
Rationale for change	To allow more flexibility for trial participants
Section to be changed	Section 5.1.1 and Section 5.1.2
Description of change	Clarification was added that in case of technical issues or inadequate quality of individual scans, MRI including MRE

Proprietary confidential information © 2025 Boehringer Ingelheim International GmbH or one or more of its affiliated companies

	assessments or Fibroscan may be repeated with approval from the sponsor.
Rationale for change	Clarification
Section to be changed	Section 5.2.1, Section 5.2.2
Description of change	Clarification was added that in case of technical issues or inadequate quality of individual scans, MRI assessments may be repeated.
Rationale for change	Clarification
Section to be changed	Section 5.2.3, Table 10
Description of change	Added pancreatic amylase test and instructions when to be performed and clarified that CK-MB test is a reflex test
Rationale for change	Clarification
Section to be changed	Section 5.2.3, Table 10
Description of change	Inclusion of INR as a reflex test
Rationale for change	INR will be tested as part of reflex testing when there is suspicion of DILI. It is added to this table for consistency with DILI monitoring requirement
Section to be changed	Section 5.2.3, Table 10
Description of change	Clarification was added that segmented neutrophils should be tested when the differential is abnormal
Rationale for change	Clarification
Section to be changed	Section 5.2.4
Description of change	Correction of time window that qualifies ECG abnormalities as baseline condition
Rationale for change	Correction
Section to be changed	Section 5.2.5.1
Description of change	It was noted that if the trial participant refuses to be referred to an MHP, the investigator will decide if the participant can remain in the trial
Rationale for change	Improve clarity regarding procedures to be followed in case of refusal to see an MHP
Section to be changed	Section 5.2.6.1.1
Description of change	Correction of time window that qualifies various abnormalities as baseline condition
Rationale for change	Correction

Proprietary confidential information © 2025 Boehringer Ingelheim International GmbH or one or more of its affiliated companies

Section to be changed	Section 4.2.1.1.3, Section 5.2.6.1.4
Description of change	Changed limit of blood glucose values which should be reported as a hypoglycaemic episode from ≤3.9 mmol/L to <3.9 mmol/L (≤70 mg/dL to <70 mg/dL).
Rationale for change	To correct a discrepancy to Table 12
Section to be changed	Section 5.2.6.1.4
Description of change	Text regarding potential DILI was amended
Rationale for change	To clarify the definition of DILI and necessary actions. AST was added to the row/scenario 4 of the Table 11 to meet the definition of Hy's Law. Text regarding hepatic injury alert was clarified
Section to be changed	Section 5.2.6.1.7
Description of change	Clarification that if acute pancreatitis is suspected in a patient, the lab test of pancreatic amylase and lipase should be done either via central labs OR local labs (if needed)
Rationale for change	To avoid any delay in the management of such patients.
Section to be changed	Section 5.2.6.1.7
Description of change	Acute severe renal events text was amended
Rationale for change	Alignment with Sections 5.2.3 and 5.2.6 (laboratory results) and clarification
Section to be changed	Section 5.2.6.2.3
Description of change	Added clarification, that for some countries, a separate pregnancy informed consent must be given to follow-up pregnancy outcome associated with drug exposure
Rationale for change	Address request of some local Ethics Committees
Section to be changed	[redacted]
Description of change	
Rationale for change	
Section to be changed	Section 6.2.2
Description of change	Removed mandatory use of video conferencing via the Study Hub application for remote visits, allowing instead video calls as an option
Rationale for change	Allowing flexibility in case Study Hub application cannot be used
Section to be changed	Section 6.2.3

Proprietary confidential information © 2025 Boehringer Ingelheim International GmbH or one or more of its affiliated companies

Description of change		Aligned the definition of trial completion with the trial design
Rationale for change		Correction
Section to be changed		Section 7.2.3.1
Description of change		Text was amended to improve clarity and application of ANCOVA clarified. Clarification was added that Statistical inference over imputed data will be guided by Rubin's rule
Rationale for change		To improve wording, improve model description, and explicitly mention how missing values will be handled by the model.
Section to be changed		Section 7.2.3.4
Description of change		Text regarding analysis relative to the efficacy estimand was amended
Rationale for change		To improve wording, improve model description, and explicitly mention how missing values will be handled by the model.
Section to be changed		Section 7.2.4, 7.2.5
Description of change		Reference to TSAP for secondary and further endpoints was added
Rationale for change		Clarification
Section to be changed		Section 7.3
Description of change		Clarification regarding handling missing data for the efficacy estimand was added
Rationale for change		Clarification

Proprietary confidential information © 2025 Boehringer Ingelheim International GmbH or one or more of its affiliated companies

11.4 GLOBAL AMENDMENT 4

This amendment is considered to be substantial based on the criteria set forth in Regulation (EU) No 536/2014 of the European Parliament and the Council of the European Union.

Overall rationale for the amendment:
To provide clarification of assessments and visit procedures for participants who prematurely discontinue IMP.

Date of amendment	03 Jul 2025
EU CT number<br>Universal Trial number	2023-505303-23-00<br>U1111-1299-9925
BI Trial Number	1404-0056
BI Investigational Medicinal Product(s)	Survodutide (BI 456906)
Title of protocol	Multicentre, randomised, double-blind, placebo-controlled, 48-week, Phase III trial to evaluate the efficacy and safety of survodutide administered subcutaneously in participants with overweight or obesity and presumed or confirmed non-alcoholic steatohepatitis (NASH)
Global Amendment due to urgent safety reasons	☐
Global Amendment	☒
Section to be changed	Clinical trial protocol synopsis
Description of change	Updates to align the synopsis with the revised protocol.
Rationale for change	To ensure consistency with the changes made in the protocol.
Section to be changed	SoA Footnote 10
Description of change	Enable the option for the ETD visit and Week 48/Visit 15, for participants who prematurely discontinue IMP, to be performed by a site's HCP or vendor at the participant's home, if available and acceptable according to local law and regulations.
Rationale for change	To enhance participant retention in the trial by offering greater flexibility.
Section to be changed	SoA Footnote 11
Description of change	Added: for participants who discontinued the IMP prematurely and are willing to continue in the trial, the EOS at W51 should only include collection of vital status, AEs/AESIs/SAEs, concomitant medication and samples for ADA/Nab.

Proprietary confidential information © 2025 Boehringer Ingelheim International GmbH or one or more of its affiliated companies

Rationale for change	To minimise patient burden by collecting only the information pertaining to safety.
Section to be changed	SoA, Footnotes 14 and 15
Description of change	Added: Patients who discontinued IMP due to pregnancy are not to undergo any imaging assessments for trial purposes.
Rationale for change	To ensure patient safety.
Section to be changed	Section 2.1.2
Description of change	Added: Absolute **and relative** change from baseline to Week 48 in HOMA-IR (FPI [mlU/L]·FPG [mmol/L]/22.5)
Rationale for change	HOMA-IR will be analysed in the log-scale; therefore this endpoint should also include a relative change from baseline.
Section to be changed	[redacted]
Description of change	
Rationale for change	
Section to be changed	
Description of change	
Rationale for change	
Section to be changed	
Description of change	
Rationale for change	
Section to be changed	Section 2.2.1
Description of change	Further endpoint removed: • High-sensitivity C-reactive protein (hsCRP) [ng/L]
Rationale for change	This endpoint has been added to the safety section.
Section to be changed	[redacted]
Description of change	

Proprietary confidential information © 2025 Boehringer Ingelheim International GmbH or one or more of its affiliated companies

Rationale for change	[redacted]
Section to be changed	
Description of change	
Rationale for change	
Section to be changed	Section 3.3
Description of change	Reduce the minimum number of participating countries from 4 to 2.
Rationale for change	Two of the initially planned countries were not initiated.
Section to be changed	Section 3.3.3.1
Description of change	Added: In case of pregnancy, the trial participant will be expected to attend the regularly scheduled study visits as outlined above, **with the exception of any imaging assessments.**
Rationale for change	To ensure patient safety
Section to be changed	Section 3.3.3.1
Description of change	Change from <u>intermittent</u> to <u>newly</u> developed bundle branch block or ventricular pacing, as follows:<br>In the case of conditions with broad QRS complex, such as newly developed bundle branch block (BBB) or ventricular pacing, an increase in QTcF interval >60 ms compared to baseline should be evaluated by the Investigator after

Proprietary confidential information © 2025 Boehringer Ingelheim International GmbH or one or more of its affiliated companies

	consultation with a cardiologist to determine whether there is a true QTcF prolongation.
Rationale for change	The wording has been updated to align with the overall survodutide program
Section to be changed	Section 3.3.3.1
Description of change	• Enable the option for the ETD visit and Week 48/Visit 15, for participants who prematurely discontinue IMP, to be performed by a site's HCP or vendor at the participant's home, if available and acceptable according to local law and regulations.<br>• Specify assessments to be done at ETD/Week 48 and FU/EOS in case participants prematurely discontinue the IMP.<br>Revised text:<br>If the participant is not willing or able to attend the clinic, the ETD and/or Week 48 visit can be performed by a site's HCP or vendor at the participant's home, if available and acceptable according to local law and regulations. In this case the assessments will be restricted to collection of weight, waist/hip circumference, vital signs, information on AE and substance usage. The FU/EOS Visit may be conducted via a remote visit (if allowed per local regulations) or over the phone for review of medical records. For participants who prematurely discontinued the IMP and are willing to continue in the trial and attend clinic visits, the FU/EOS at Week 51 should only include collection of vital status, AEs/AESIs/SAEs, concomitant medication and samples for ADA/Nab.
Rationale for change	• To enhance participant retention in the trial by offering greater flexibility.<br>• To minimise patient burden by collecting only the information pertaining to safety.
Section to be changed	Section 3.3.3.1
Description of change	Clarify that participants who prematurely discontinue IMP due to pregnancy are not to undergo any imaging assessments for trial purposes.
Rationale for change	To ensure patient safety.
Section to be changed	[redacted]
Description of change	

Proprietary confidential information © 2025 Boehringer Ingelheim International GmbH or one or more of its affiliated companies

Rationale for change	[redacted]
Section to be changed	Section 5.1.1
Description of change	Clarify that participants who prematurely discontinue IMP due to pregnancy are not to undergo any imaging assessments for trial purposes.
Rationale for change	To ensure patient safety.
Section to be changed	Section 5.1.2.5
Description of change	Clarify that the requirement for blood pressure measurements to be conducted using the same method and device applies only to on-site visits.
Rationale for change	To enhance flexibility in remote settings.
Section to be changed	[redacted]
Description of change	
Rationale for change	
Section to be changed	Throughout the document
Description of change	Minor editorial and formatting adjustments
Rationale for change	To maintain a consistent style and improve overall readability.

Signature Page for VV-TMF-981278 v5.0

Reason for signing: Approved	Name: [redacted]<br>Role: A[redacted]<br>Date of signature: 07-Jul-2025 15:15:47 GMT+0000
Reason for signing: Approved	Name: [redacted]<br>Role: A[redacted]<br>Date of signature: 08-Jul-2025 06:43:21 GMT+0000
Reason for signing: Approved	Name: [redacted]<br>Role: A[redacted]<br>Date of signature: 08-Jul-2025 09:42:58 GMT+0000
Reason for signing: Approved	Name: [redacted]<br>Role: A[redacted]<br>Date of signature: 09-Jul-2025 16:00:07 GMT+0000
Reason for signing: Approved	Name: [redacted]<br>Role: A[redacted]<br>Date of signature: 10-Jul-2025 17:18:39 GMT+0000
Reason for signing: Approved	Name: [redacted]<br>Role: V[redacted]Paper Signature Completion<br>Date of signature: 14-Jul-2025 12:41:58 GMT+0000

TRIAL STATISTICAL ANALYSIS PLAN

**Global ID Version:**	**228892_303524_1.0**
**BI Trial No.:**	**1404-0056**
**Title:**	Multicenter, randomised, double-blind, placebo-controlled, 48 week, Phase III trial to evaluate the efficacy and safety of survodutide administered subcutaneously in participants with overweight or obesity and presumed or confirmed non-alcoholic steatohepatitis (NASH)
**Investigational Product(s):**	Survodutide (BI 456906)
**Responsible trial statistician(s):**	[redacted]<br>Tel.: [redacted]<br>Email: [redacted]
**Date of statistical analysis plan:**	23JAN2026
**Version:**	1.0

Proprietary confidential information
© 2026 Boehringer Ingelheim International GmbH or one or more of its affiliated companies. All rights reserved.
This document may not - in full or in part - be passed on, reproduced, published or otherwise used without prior written permission.

Proprietary confidential information © 2026 Boehringer Ingelheim International GmbH or one or more of its affiliated companies.

1. TABLE OF CONTENTS

TITLE PAGE......1

1. TABLE OF CONTENTS......2

LIST OF TABLES......4

2. LIST OF ABBREVIATIONS......5

3. INTRODUCTION......9

4. CHANGES IN THE PLANNED ANALYSIS OF THE STUDY......9

5. ENDPOINTS......10
5.1 PRIMARY ENDPOINT(S)......10
5.2 SECONDARY ENDPOINT(S)......11
5.2.1 Key secondary endpoint(s)......11
5.2.2 Secondary endpoint(s)......11
5.3 FURTHER ENDPOINT(S)......13
5.4 OTHER VARIABLE(S)......25
5.4.1 Safety......25
5.4.2 Demographics and other baseline characteristics......29
5.4.3 Treatment exposure and compliance......29
[redacted]
6. GENERAL ANALYSIS DEFINITIONS......31
6.1 TREATMENT(S)......31
6.1.1 Study Periods......31
6.1.2 Definition of Treatment Groups......32
6.1.3 Definition of On-Treatment......32
6.2 IMPORTANT PROTOCOL DEVIATIONS......33
6.3 INTERCURRENT EVENTS......33
6.4 SUBJECT SETS ANALYSED......34
[redacted]
6.6 HANDLING OF MISSING DATA AND OUTLIERS......37
6.7 BASELINE, TIME WINDOWS AND CALCULATED VISITS......39

7. PLANNED ANALYSIS......44
7.1 DEMOGRAPHIC AND OTHER BASELINE CHARACTERISTICS......45
7.2 CONCOMITANT DISEASES AND MEDICATION......46
7.3 TREATMENT COMPLIANCE......47
7.4 PRIMARY OBJECTIVE ANALYSIS......47
7.4.1 Relative reduction in liver fat content of at least 30% from baseline to Week 48 assessed by MRI-PDFF (yes/no)......47
7.4.1.1 Main analysis......47
7.4.1.2 Sensitivity analysis......51
[redacted]

Proprietary confidential information © 2026 Boehringer Ingelheim International GmbH or one or more of its affiliated companies.

7.4.2 Relative change in body weight from baseline to Week 48 (%) 56
7.4.2.1 Main analysis 56
7.4.2.2 Sensitivity analysis 59
[redacted]
7.5 SECONDARY OBJECTIVE ANALYSIS 63
7.5.1 Key secondary objective analysis 63
7.5.2 Secondary objective analysis 63
7.6 FURTHER OBJECTIVE ANALYSIS 67
7.7 EXTENT OF EXPOSURE 72
7.8 SAFETY ANALYSIS 73
7.8.1 Adverse Events 73
7.8.1.1 Assignment of AEs to analysis phases 74
7.8.1.2 AE summaries 74
7.8.1.3 Adverse events of special interest (AESIs) 75
7.8.1.4 Specific AEs 78
7.8.1.5 Adjudicated events 79
7.8.1.6 Possible cases of IMP abuse or overuse 80
7.8.1.7 Adverse event incidence rates 80
7.8.2 Laboratory data 81
7.8.2.1 General analysis 81
[redacted]
7.8.3 Vital signs 84
7.8.4 ECG 84
7.8.4.1 Quantitative variables 84
7.8.4.2 Categorical variables 85
7.8.4.3 Binary variables 85
7.8.5 Mental health questionnaires 86
[redacted]
8. TIMEPOINT OF RELEASE OF TREATMENT INFORMATION 90
9. REFERENCES 91
10. ADDITIONAL SECTIONS 93
10.1 PATTERN MERGING ALGORITHM FOR RETRIEVED DROPOUT MULTIPLE IMPUTATION ANALYSIS OF BODY WEIGHT 93
10.2 VARIABLES TO BE ANALYSED AFTER LOGARITHMIC TRANSFORMATION 94
10.3 DEFINITION OF SPECIAL ADVERSE EVENTS 95
11. HISTORY TABLE 97

Proprietary confidential information © 2026 Boehringer Ingelheim International GmbH or one or more of its affiliated companies.

LIST OF TABLES

Table 1 New onset derivation for QT....27
Table 2 New onset derivation for QTcF....28
Table 3 Aggregation rules for overall ECG interpretation....28
Table 4 Study periods....31
Table 5 Subject sets analysed....35
[redacted]
Table 7 Visit windows for variables scheduled to be assessed at all clinic and remote visits.42
Table 8 Visit windows for variables with a sparse clinic visit schedule....43
[redacted]
Table 10 Starting patterns for retrieved dropout (MI3) analysis of MRI-PDFF....50
[redacted]
Table 12 History table....97

Proprietary confidential information © 2026 Boehringer Ingelheim International GmbH or one or more of its affiliated companies.

2. LIST OF ABBREVIATIONS

Term	Definition / description
[redacted]	
AE	Adverse Event
[redacted]	
ALT	Alanine aminotransferase (SGPT)
ANCOVA	Analysis of covariance
AP	Alkaline phosphatase
[redacted]	
AST	Aspartate aminotransferase (SGOT)
ATC	Anatomical Therapeutic Chemical
BI	Boehringer Ingelheim
BIcMQ	Boehringer Ingelheim customized MedDRA Query
BITS	Boehringer Ingelheim Tailored Search
BMI	Body Mass Index
BP	Blood pressure
CAP	Control Attenuation Parameter
CI	Confidence interval
CKD-EPI	Chronic Kidney Disease Epidemiology Collaboration
[redacted]	
C-SSRS	Columbia – Suicide Severity Rating Scale
cT1	Iron corrected T1
CTIS	Clinical Trials Information System
[redacted]	
eCRF	electronic Case report form
CTC	Common Terminology Criteria
CTP	Clinical Trial Protocol
CTR	Clinical Trial Report
CV	Cardiovascular
DBL	Database Lock
DILI	Drug-Induced Liver Injury

Proprietary confidential information © 2026 Boehringer Ingelheim International GmbH or one or more of its affiliated companies.

Term	Definition / description
DMC	Data Monitoring Committee
DTA	Data Transfer Agreement
ECG	Electrocardiogram
EDMS	Electronic Document Management System
eGFR	Estimated Glomerular Filtration Rate
ELF	Enhanced Liver Fibrosis
ENMO	Euclidean Norm Minus One
EOSS	Edmonton Obesity Staging System
EOT	End of treatment
FAS	Full Analysis Set
[redacted]	
FCS	Fully conditional specification
[redacted]	
FPG	Fasting plasma glucose
FPI	Fasting plasma insulin
FTR	Follow the reference
[redacted]	
HDL	High Density Lipoprotein
HF	Heart Failure
HFE	Heart Failure Event
HHF	Hospitalisation for Heart Failure
HLT	Higher Level Term
HOMA-IR	Homeostasis Model Assessment – Insulin Resistance
[redacted]	
HR	Heart rate
[redacted]	
ICE	Intercurrent event
ICH	International Conference on Harmonisation

Proprietary confidential information © 2026 Boehringer Ingelheim International GmbH or one or more of its affiliated companies.

Term	Definition / description
IMP	Investigational Medicinal Product
IPD	Important protocol deviation
IRT	Interactive Response Technology
[redacted]	
LDL	Low Density Lipoprotein
LLQ	Lower Limit of Quantification
MACE	Major Adverse Cardiovascular Events
MAR	Missing at random
MASH	Metabolic dysfunction-associated steatohepatitis
MCAR	Missing completely at random
MedDRA	Medical Dictionary for Regulatory Activities
MET	Metabolic Equivalent of Task
mg	milligram
MI	Myocardial infarction
miRNA	Micro ribonucleic acid
MMRM	Mixed model for repeated measures
MNAR	Missing not at random
MRE	Magnetic Resonance Elastography
MRI	Magnetic Resonance Imaging
MRI-PDFF	Magnetic Resonance Imaging - Proton Density Fat Fraction
MVPA	Moderately to Vigorous Physical Activity
NA	Not applicable
[redacted]	
NAFLD	Non-Alcoholic Fatty Liver Disease
NAS	NAFLD Activity Score
NASH	Non-Alcoholic SteatoHepatitis
NYHA	New York Heart Association
PD	Protocol deviation
PK	Pharmacokinetics
[redacted]	

Proprietary confidential information © 2026 Boehringer Ingelheim International GmbH or one or more of its affiliated companies.

Term	Definition / description
PMM	Predictive mean matching
[redacted]	
PT	Preferred term
Q1	Lower quartile
Q3	Upper quartile
RD	Retrieved dropout
REP	Residual effect period
RPM	Report Planning Meeting
RS&I	Random slope and intercept
SAP	Statistical analysis plan
SD	Standard deviation
SDG	Standardised Drug Grouping
SOC	System organ class
SRD	Standardised risk difference
T2DM	Type 2 diabetes mellitus
[redacted]	
TMF	Trial Master File
TS	Treated Set
[redacted]	
ULN	Upper limit of normal
ULQ	Upper Limit of Quantification
VLDL	Very Low Density Lipoprotein
[redacted]	
WHO	World Health Organisation

Proprietary confidential information © 2026 Boehringer Ingelheim International GmbH or one or more of its affiliated companies.

3. INTRODUCTION

As per ICH E9, the purpose of this document is to provide a more technical and detailed elaboration of the principal features of the analysis described in the protocol, and to include detailed procedures for executing the statistical analysis of the primary and secondary variables and other data.

This TSAP assumes familiarity with the Clinical Trial Protocol (CTP), including Protocol Amendments. In particular, the TSAP is based on the planned analysis specification as written in CTP Section 7 "Statistical Methods and Determination of Sample Size". Therefore, TSAP readers may consult the CTP for more background information on the study, e.g., on study objectives, study design and population, treatments, definition of measurements and variables, planning of sample size, randomisation.

This document describes one final analysis of study 1404-0056. No interim analyses are planned.

This TSAP is based on the 1404-0056 CTP (Version 5.0, 03JUL2025), together with the latest versions of the eCRF and relevant Data Transfer Agreements (DTAs) for external vendor data.

All statistical analyses defined in this TSAP will be performed by the trial statistician (TSTAT) with support from other trial team members following unblinding of the trial at database lock (DBL). This TSAP will be finalized and signed prior to DBL.

A DBL meeting will be held prior to taking the final database snapshot. It will be decided at this meeting whether any open cleaning issues will be accepted or not. Important protocol deviations (IPDs) will also be finalized prior to DBL.

SAS® version 9.4 or later will be used for all analysis. Where statistical analyses require the use of R instead of SAS®, R version 4.2.2 or later will be used.

The analyses and outputs specified in this TSAP will be included in the clinical trial report (CTR) unless specified otherwise.

This document will use the wording "non-alcoholic steatohepatitis (NASH)" for consistency with the CTP, which will not be changed for this, including in the study title. The newer term "metabolic dysfunction-associated steatohepatitis (MASH)" is expected to be used in other documents describing this trial, and is acknowledged here to mean the same thing.

4. CHANGES IN THE PLANNED ANALYSIS OF THE STUDY

The analysis models specified in the CTP included fixed categorical effects for region. Since only 1 participant is expected to be randomised outside USA, this will no longer be done.

Proprietary confidential information © 2026 Boehringer Ingelheim International GmbH or one or more of its affiliated companies.

[redacted]

5. ENDPOINTS

5.1 PRIMARY ENDPOINT(S)

Two co-primary endpoints are defined in this trial:

1) **Relative reduction in liver fat content of at least 30% from baseline to Week 48 assessed by MRI-PDFF (yes/no)**

For the purposes of this primary endpoint, liver fat content (median PDFF) in % is defined as the whole liver median obtained from a technically acceptable image reading. The median will be calculated by the vendor using all available pixels and will be provided in the data transfer. (Other percentile PDFF values which are also provided in the data transfer are primarily for assessing the quality of the imaging procedure, and will not be used directly in any analysis.)

All non-missing values of liver fat content will be used for analysis, with the following exception. If a (non-missing) median PDFF is provided in the data transfer for which another (non-missing) median PDFF value was provided at the same visit because of a re-scan, the latter will be used for analysis. The original value will be assumed to reflect a technically inadequate PDFF measurement, even if other technically adequate imaging data were obtained from the original scan.

Baseline liver fat content (median PDFF) is expected to be a screening value. Participants with a liver fat content (median PDFF) value within the Week 48 visit window who meet the endpoint definition are defined as responders, and participants with a value within the Week 48 visit window who do not meet the endpoint definition are defined as non-responders. For the primary (treatment regimen) estimand, this statement applies regardless of the occurrence of any of the intercurrent events which were pre-specified in the protocol prior to the Week 48 MRI scan date (except for death, if any deaths occur on study, which would anyway preclude the availability of subsequent data). Further details regarding definition of intercurrent events, and primary and supplementary estimands with regard to these, are given in Section 7.4.1 and

Proprietary confidential information © 2026 Boehringer Ingelheim International GmbH or one or more of its affiliated companies.

Section 7.4.2. Further details on baseline and visit window definitions are provided in Section 6.7.

It is not anticipated that there will be a need to perform single imputation of participants without a value in the Week 48 visit window as non-responders in the analysis datasets for this trial, and that this topic will be addressed entirely through the analyses specified in Section 7.

2) Relative change in body weight from baseline to Week 48 (%)

For the purposes of this primary endpoint, body weight measured at the clinic will be used, as recorded on the eCRF in kg. All body weight data recorded on the eCRF will be considered eligible for primary endpoint purposes. For the primary (treatment regimen) estimand, if the participant does not return to the clinic after early treatment discontinuation and only a remote visit is possible, it is anticipated that a study nurse will attempt to weigh the participant at home using standard calibrated equipment, and that this weight measurement will be entered into the eCRF (and not any other type of home measurement).

Baseline body weight is expected to be the value from the randomisation visit. Visit windows will be used to determine what is considered to be Week 48. Further details on baseline and visit window definitions are provided in Section 6.7. For the primary (treatment regimen) estimand, Week 48 body weight data will be used regardless of the occurrence of any of the intercurrent events which were pre-specified in the protocol prior to the Week 48 clinic visit date (except for death, if any deaths occur on study, which would anyway preclude the availability of subsequent data). Further details regarding definition of intercurrent events, and primary and supplementary estimands with regard to these, are given in Section 7.4.1 and Section 7.4.2.

5.2 SECONDARY ENDPOINT(S)

5.2.1 Key secondary endpoint(s)

There are no key secondary endpoints defined in this trial.

5.2.2 Secondary endpoint(s)

Secondary endpoints are defined as follows. All laboratory values used in any secondary endpoint definitions are non-normalised values:

- Absolute change from baseline to Week 48 in liver fat content assessed by MRI-PDFF (%)

- Relative change from baseline to Week 48 in liver fat content assessed by MRI-PDFF (%)

Liver fat content (MRI-PDFF) data selection is the same as what is described for the primary endpoint in Section 5.1.

Proprietary confidential information © 2026 Boehringer Ingelheim International GmbH or one or more of its affiliated companies.

- Absolute reduction in cT1 levels of at least 80 ms from baseline to Week 48 (yes/no)

cT1 levels will be selected from MRI similarly to liver fat content (see Section 5.1), using only the median cT1 (calculated across the whole liver) for analysis and not other percentiles in the vendor data transfer, and with similar handling of any re-scans. Note that it is possible that cT1 may be selected from the original scan and liver fat content (MRI-PDFF) from a re-scan, or vice versa.

- Absolute change from baseline to Week 48 in ALT (U/L)

- Relative change from baseline to Week 48 in ALT (%)

- Absolute change from baseline to Week 48 in waist circumference (cm)

- Relative change from baseline to Week 48 in waist circumference (%)

- Absolute change from baseline to Week 48 in HOMA-IR

- Relative change from baseline to Week 48 in HOMA-IR

Insulin resistance using HOMA-IR is calculated from fasting plasma glucose (FPG) (in mmol/L) and fasting plasma insulin (FPI) (in mIU/L) as follows:

HOMA-IR = (FPI x FPG) / 22.5.

HOMA-IR will be calculated by the central laboratory and used directly for analysis. All visits after screening are expected to be performed fasted. Baseline is expected to be the value from the randomisation visit; if this is missing, then baseline is missing, since data are not expected to be available at screening, which is anyway not fasted. Further details on baseline definitions are provided in Section 6.7. Any available HOMA-IR values will be assumed to be fasted and used for analysis, unless there is clear information in the data transfer to indicate that specific values were obtained non-fasted or the fasting status is missing, in which case they will be considered missing for analysis. Any participant who is receiving background insulin at the time of study treatment start will be excluded from HOMA-IR analysis.

- Absolute change from baseline to Week 48 in liver stiffness assessed by MRE (kPa)

- Relative change from baseline to Week 48 in liver stiffness assessed by MRE (%)

Liver stiffness in kPa is defined as the mean liver stiffness over a number of different slice locations from a technically acceptable image reading. The minimum number of slice locations is expected to be 2. Values provided in the data transfer for individual slice locations

Proprietary confidential information © 2026 Boehringer Ingelheim International GmbH or one or more of its affiliated companies.

will not be used for analysis. Handling of any re-scans will be similar to what is specified for liver fat content (MRI-PDFF) in Section 5.1. Note that it is possible that liver stiffness may be selected from the original scan and liver fat content (MRI-PDFF) from a re-scan, or vice versa. Any liver stiffness value which did not clearly meet the 4-hour fasting requirement will be excluded from analysis (this includes any situation in which the fasting information remains missing/unknown and cannot be resolved).

- Absolute change from baseline to Week 48 in liver volume measured by MRI (mL)

- Relative change from baseline to Week 48 in liver volume measured by MRI (%)

Liver volume is measured from the MRI in units of mm^3 which will be converted to mL by the vendor prior to data transfer. Handling of any re-scans will be similar to what is specified for liver fat content (MRI-PDFF) in Section 5.1. Note that it is possible that liver volume may be selected from the original scan and liver fat content (MRI-PDFF) from a re-scan, or vice versa.

5.3 FURTHER ENDPOINT(S)

Further endpoints are defined as follows. [redacted]

Further endpoints from MRI or MRE

- Absolute change from baseline to Week 28 in liver fat content assessed by MRI-PDFF (%)

- Relative change from baseline to Week 28 in liver fat content assessed by MRI-PDFF (%)

- Absolute change from baseline to Week 28 in liver stiffness assessed by MRE (kPa)

- Relative change from baseline to Week 28 in liver stiffness assessed by MRE (%)

- Absolute change from baseline to Week 28 in liver volume measured by MRI (mL)

- Relative change from baseline to Week 28 in liver volume measured by MRI (%)

- Absolute change from baseline to Week 28 in cT1 (ms)

Proprietary confidential information © 2026 Boehringer Ingelheim International GmbH or one or more of its affiliated companies.

- Relative change from baseline to Week 28 in cT1 (%)
- Absolute change from baseline to Week 48 in cT1 (ms)
- Relative change from baseline to Week 48 in cT1 (%)
- Relative reduction in liver fat content of at least 30% from baseline to Week 28 assessed by MRI-PDFF (yes/no)
- Relative reduction in liver fat content of at least 50% from baseline to Week 48 assessed by MRI-PDFF (yes/no)
- Relative reduction in liver fat content of at least 70% from baseline to Week 48 assessed by MRI-PDFF (yes/no)
- Liver fat content of less than 5% at Week 48 assessed by MRI-PDFF (yes/no)

[redacted].

Data for the MRI and MRE endpoints at Week 28 will be selected similarly to their Week 48 counterparts, as described in Section 5.1 and Section 5.2.2.

Further endpoints from clinic measurements

- Absolute change from baseline to Week 48 in BMI (kg/m^2)

BMI will be calculated as weight (kg) / height ^{2} (m^2).

- Absolute change from baseline to Week 48 in body weight (kg)
- Absolute change from baseline to Week 48 in systolic blood pressure (mmHg)
- Absolute change from baseline to Week 48 in diastolic blood pressure (mmHg)

Blood pressure measurements are captured pre-dose at the dosing visits in this trial, not also post-dose. It is expected that 3 blood pressure measurements will be captured on the eCRF at scheduled visits. For the systolic and diastolic blood pressure endpoints, the mean of these 3 measurements (or however many available) will be used.

The following additional responder variables will be evaluated for body weight:

Proprietary confidential information © 2026 Boehringer Ingelheim International GmbH or one or more of its affiliated companies.

- Weight reduction of ≥ 5% from baseline at Week 48 (yes/no)
- Weight reduction of ≥ 10% from baseline at Week 48 (yes/no)
- Weight reduction of ≥ 15% from baseline at Week 48 (yes/no)
- Weight reduction of ≥ 20% from baseline at Week 48 (yes/no)

[redacted]

<u>Further endpoints from central laboratory data</u>

- Relative change from baseline to Week 48 in total cholesterol (mg/dL)
- Relative change from baseline to Week 48 in high density lipoprotein cholesterol (HDL-C) (mg/dL)
- Relative change from baseline to Week 48 in low density lipoprotein cholesterol (LDL-C) (mg/dL)
- Relative change from baseline to Week 48 in very low density lipoprotein cholesterol (VLDL-C) (mg/dL)
- Relative change from baseline to Week 48 in triglycerides (mg/dL)

[redacted]

- Absolute change from baseline to Week 48 in fasting plasma glucose (FPG) (mg/dL)
- Relative change from baseline to Week 48 in fasting plasma insulin (FPI) (mIU/L)

Proprietary confidential information © 2026 Boehringer Ingelheim International GmbH or one or more of its affiliated companies.

[redacted]

- Absolute change from baseline to Week 48 in HbA1c (%)

- Absolute change from baseline to Week 48 in HbA1c (mmol/mol)

This will be derived from HbA1c in % using the following formula:

HbA1c (mmol/mol) = (HbA1c (%) – 2.15) x 10.929

[redacted]

Proprietary confidential information © 2026 Boehringer Ingelheim International GmbH or one or more of its affiliated companies.

[redacted]

- Absolute change from baseline to Week 48 in ELF score
- Relative change from baseline to Week 48 in ELF score (%)

ELF score will be calculated by the central laboratory and used directly for analysis. The ELF score will be calculated from measurements of hyaluronic acid, procollagen III amino terminal peptide and tissue inhibitor of metalloproteinase 1, which will also be provided individually for analysis (relative changes only).

[redacted]

Proprietary confidential information © 2026 Boehringer Ingelheim International GmbH or one or more of its affiliated companies.

[redacted]

- Absolute change from baseline to Week 48 in AST (U/L)
- Relative change from baseline to Week 48 in AST (%)

[redacted]

Proprietary confidential information © 2026 Boehringer Ingelheim International GmbH or one or more of its affiliated companies.

- Relative change from baseline to Week 48 in uric acid (%)

[redacted]

Further endpoints from laboratory biomarkers

[redacted]

Proprietary confidential information © 2026 Boehringer Ingelheim International GmbH or one or more of its affiliated companies.

[redacted]

Further endpoints from FibroScan®

- Absolute change from baseline to Week 48 in liver fat content assessed by FibroScan® CAP™ (dB/m)

Proprietary confidential information © 2026 Boehringer Ingelheim International GmbH or one or more of its affiliated companies.

- Relative change from baseline to Week 48 in liver fat content assessed by FibroScan® CAP™ (%)

[redacted]

Proprietary confidential information © 2026 Boehringer Ingelheim International GmbH or one or more of its affiliated companies.

[redacted]

Four different endpoints will be derived for analysis from the IWQOL-Lite-CT questionnaire, [redacted]

Proprietary confidential information © 2026 Boehringer Ingelheim International GmbH or one or more of its affiliated companies.

[redacted]

Proprietary confidential information © 2026 Boehringer Ingelheim International GmbH or one or more of its affiliated companies.

[redacted]

Proprietary confidential information © 2026 Boehringer Ingelheim International GmbH or one or more of its affiliated companies.

[redacted]

5.4 OTHER VARIABLE(S)

5.4.1 Safety

Safety will be assessed based on:

- Adverse events
  - Routine reporting using the Medical Dictionary for Regulatory Agencies (MedDRA) coding dictionary (version in force at DBL)
  - Adverse events of special interest (AESIs)
  - Adjudicated adverse events

Proprietary confidential information © 2026 Boehringer Ingelheim International GmbH or one or more of its affiliated companies.

  - Additional trial-specific AE data captured on the eCRF for: hypersensitivity/anaphylactic reactions; acute gall bladder disease; acute renal event; acute pancreatitis; pancreatic cancer; local tolerability and injection site reactions; hypoglycaemia; hyperglycaemia; hypotension; thyroid mass; arrythmias and cardiac conduction disorders.
  - Ophthalmic examination at specific visits

[redacted]

- Vital signs
  - Pulse rate
  - Systolic blood pressure
  - Diastolic blood pressure

- 12-lead ECG

  Further details regarding 12-lead ECG derivations are given below.

- Columbia - Suicide Severity Rating Scale (C-SSRS)

- Patient Health Questionnaire 9 item depression module (PHQ-9).

[redacted]

<u>12-lead ECG</u>

Single digital ECG recordings are planned to be collected at each relevant visit with the exception of screening, where triplicate ECG recordings are planned. Each of the recorded single ECGs will then be evaluated for cardiac intervals, which comprise the RR, PR and QT interval and the QRS duration. Measurements of these intervals will be made on four cardiac cycles from the lead chosen (details not specified here).

The measurements of the cardiac cycles will be stored in the database, i.e. four values per single ECG. The four cardiac cycles (or however many available) will be averaged, to give a mean value for each single ECG. This will be done prior to the calculation below of the heart rate (HR) and heart rate corrected QT interval (QTc).

Proprietary confidential information © 2026 Boehringer Ingelheim International GmbH or one or more of its affiliated companies.

For screening only, where a triplicate ECG is expected, the mean values obtained for each single ECG will be further averaged across the 3 ECGs (or however many available) to give a single mean value for each variable at screening.

The heart rate will be derived from the RR interval as:

$$\text{HR [beats/min]} = \frac{60\,000}{\overline{\text{RR}}}$$

where $\overline{RR}$ is the mean of the four RR intervals (measured in msec).

The QT interval corrected for HR according to Fridericia's formula (QTcF) for a single ECG will be derived as:

$$\overline{\text{QTcF}}\text{ [msec]} = \left(\frac{1000}{\overline{\text{RR}}}\right)^{1/3} * \overline{\text{QT}}\text{[msec]},$$

where $\overline{\text{QT}}$ is the mean of the four QT intervals and $\overline{\text{RR}}$ is the mean of the corresponding preceding RR intervals of the four cardiac cycles for this ECG.

In the case that more than one ECG is available (i.e. more than one group of cardiac cycles) at a visit where a single ECG was planned, then the (first) ECG on that day which is not labelled as "UNSCHEDULED" will be used, regardless of the reason for this.

Rules for the derivation of new onsets for categorical endpoints and aggregation rules for the overall ECG interpretation are described in the tables below:

Table 1 New onset derivation for QT

QT		
**On treatment**	**At baseline**	**"New onset of values above thresholds" (aggregated result presented in Table)**
≤ 500 msec (all available time points)	Any value	No new onset
> 500 msec (at least one time point)	> 500 msec	No new onset
	≤ 500 msec or missing	New onset > 500 msec

Proprietary confidential information © 2026 Boehringer Ingelheim International GmbH or one or more of its affiliated companies.

Table 2 New onset derivation for QTcF

QTcF		
**On treatment**	**At baseline**	**"New onset of values above thresholds" (aggregated result presented in Table)**
≤ 450 msec (all available time points)	Any value	No new onset
> 450 msec (at least one time point), ≤ 480 msec (all available time points)	> 450 msec	No new onset
	≤ 450 msec or missing	New onset > 450 msec
> 480 msec (at least one time point), ≤ 500 msec (all available time points)	> 480 msec	No new onset
	≤ 480 msec or missing	New onset > 480 msec
> 500 msec (at least one time point)	> 500 msec	No new onset
	≤ 500 msec or missing	New onset > 500 msec

Table 3 Aggregation rules for overall ECG interpretation

Overall ECG Interpretation		
**On treatment**	**At baseline**	**"New onset of values above thresholds" (aggregated result presented in Table)**
Normal (all available time points)	Normal, abnormal or missing/not evaluable	Normal
Abnormal (at least one time point)	Abnormal	Abnormal, and abnormal at baseline
	Normal or missing/not evaluable	Abnormal, and normal at baseline (new onset)

Proprietary confidential information © 2026 Boehringer Ingelheim International GmbH or one or more of its affiliated companies.

5.4.2 Demographics and other baseline characteristics

Standard demographic variables and baseline characteristics are used as recorded on the eCRF.

Baseline definitions are given in Section 6.7.

Time since NASH diagnosis will be derived as *(randomisation date – NASH diagnosis date).* Informed consent date will not be used, in anticipation that the diagnosis date for presumed NASH (according to one of the permitted methods in the protocol for this) might have been entered which is after the date of informed consent. Further information captured at baseline includes:

- Whether the diagnosis is presumed or confirmed
- If presumed, the method of presumption
- If confirmed, whether the confirmatory biopsy was within 3 years or not, NAS score and sub-scores, and fibrosis stage obtained from the biopsy
- Previous types of treatment for NASH.

The length of time the participant has suffered with overweight/obesity will be obtained directly from the eCRF. Further information captured at baseline includes:

- Family history
- Previous types of treatment for obesity or overweight.
- Average sleep hours and presence/absence of sleep apnea, insomnia and snoring
- Current stage of obesity using the Edmonton obesity staging system (EOSS)
- New York Heart Association (NYHA) functional classification
- Substance use (smoking and alcohol status)

5.4.3 Treatment exposure and compliance

Treatment exposure will be defined as the number of days from the first dose of study treatment to the last dose of study treatment inclusive. Any intermediate gaps in treatment in which the participant did not take medication will not be considered (any such gaps are expected to be short in this trial). The same exposure calculation will be performed regardless of whether the participant participated in the optional 4-week treatment extension or not. It is assumed that an End of Treatment eCRF page will be available and complete for all treated participants at DBL, and that no special rules are needed to handle any missing information here.

In addition, treatment exposure to planned maintenance dose will be defined as the number of days from the first dose of the planned maintenance dose of IMP to the last dose of the planned maintenance dose of IMP, excluding periods in which the participant did not take the planned maintenance dose of IMP. Planned maintenance dose is defined as 6.0 mg of survodutide (only). Participants who escalated to a maintenance dose which was lower than the planned dose will be included in this table as zero days.

Proprietary confidential information © 2026 Boehringer Ingelheim International GmbH or one or more of its affiliated companies.

Treatment exposure duration will be categorized as follows:
<4 weeks; 4-<8 weeks; 8-<12 weeks; 12-<16 weeks; 16-<20 weeks; 20-<24 weeks, 24-<28 weeks; 28-<32 weeks; 32-<36 weeks; 36-<40 weeks; 40-<44 weeks; 44-<48 weeks; 48-<52 weeks; ≥52 weeks.

Study duration (regardless of whether on-treatment) will also use the same category definitions.

Treatment compliance will be defined as the number of doses administered divided by the number of doses expected, multiplied by 100 to obtain a percentage.

The expected number of doses will be calculated as the number of weeks on treatment from date of first administration to date of last administration (since dosing is weekly).

[redacted]

Proprietary confidential information © 2026 Boehringer Ingelheim International GmbH or one or more of its affiliated companies.

6. GENERAL ANALYSIS DEFINITIONS

6.1 TREATMENT(S)

6.1.1 Study Periods

The following summarises the study periods defined in the protocol for a participant who completes all visits and remains on treatment as planned, including for the whole length of the treatment extension period:

Table 4 Study periods

Study Phase Description	Start	End
Screening period	Date of informed consent	Day before Visit 2 (Week 0)*
Treatment period – dose escalation	Date of Visit 2 (Week 0)*	Day before Visit 10 (Week 24)
Treatment period – maintenance	Date of Visit 10 (Week 24)	Date of Visit 15 (Week 48)
Treatment period – optional extension	Day after Visit 15 (Week 48)	Date of Visit 16 (Week 52)
Follow-up period	Day after Visit 16 (Week 52)	Date of end of study participation

* See also baseline definitions in Section 6.7.

Participants who discontinue treatment early are nevertheless encouraged to remain in the study as specified above, at least for the minimum assessment of key data (including body weight and ideally MRI-PDFF). The CTP section 3.3.4.1 outlines the preferred hierarchy of options to encourage continued study participation in these circumstances. Such participants are expected to provide data that are later than 4 weeks after last dose of study treatment, in some cases considerably later, if the protocol aim to retain these participants off-treatment up to Visit 15 (Week 48) is successful.

The follow-up period will not exist for any randomised participant who was lost to follow-up whilst still receiving treatment.

The definition of on-treatment for analysis purposes is given in Section 6.1.3. The use of a residual effect period (REP) means that data captured during the follow-up period specified in the protocol could be assigned to the treatment period for analysis.

Proprietary confidential information © 2026 Boehringer Ingelheim International GmbH or one or more of its affiliated companies.

6.1.2 Definition of Treatment Groups

Participants are not randomised to specific survodutide doses in this trial. However it is acknowledged that not all participants randomised to survodutide 6.0 mg will receive survodutide at this dose, either because

- the participant could not escalate successfully to the 6.0 mg dose and started maintenance treatment on a lower dose, or
- the participant did not reach the maintenance period and discontinued treatment early whilst receiving a lower dose.

Planned treatment groups will be used for all analysis. It is not anticipated that any analysis will present treatment groups split by survodutide dose, although the specific survodutide dose may be indicated on relevant data listings.

Any participant who received an incorrect treatment or an incorrect survodutide dose (if the latter is possible to identify from the data) will be listed separately.

6.1.3 Definition of On-Treatment

It is particularly noted here that the protocol encourages continued study participation for participants who have discontinued study treatment early. It is therefore expected that for some participants there will be off-treatment data captured during the planned treatment period.

In addition, even for participants who completed treatment according to the protocol, data captured during the follow-up period may be relevant for safety.

The definition of "on-treatment" for analysis purposes is as described below. It is assumed that all participants will have a date of last administration of study medication accurately recorded on the End of Treatment eCRF page, and the definition makes no distinction whether the last administration of study medication was in the optional treatment extension period or not.

"Off-treatment" refers to data which are captured after baseline but which are not "on-treatment" as defined below.

<u>Safety</u>

An AE start date or other safety assessment date will be considered "on-treatment" if the AE start date or assessment date is between the date of first dose and 28 days after the date of last dose of study treatment inclusive (the residual effect period (REP) is defined as 28 days in this trial).

The same on-treatment definition will be used for all types of safety data.

Proprietary confidential information © 2026 Boehringer Ingelheim International GmbH or one or more of its affiliated companies.

Efficacy

Efficacy data will also use the same 28 day definition of "on-treatment" as for safety.

The same on-treatment definition will be used for all types of efficacy data, [redacted]

6.2 IMPORTANT PROTOCOL DEVIATIONS

Handling of IPDs is included in the DV domain specifications and stored within the TMF in EDMS.

IPDs will not lead to exclusion from analysis sets.

6.3 INTERCURRENT EVENTS

Intercurrent events (ICEs) (as detailed in the CTP Section 7.2.2) fall into 3 types:

1) Premature treatment discontinuation (irrespective of reason)
2) Prohibited anti-obesity medications, which are expected to fall into 2 sub-categories:
    a. Those which contain GLP-1R agonists
    b. Those which do not contain GLP-1R agonists
3) Death from any cause (if any occur during the study)

It is possible that a participants may have more than one ICE in the above list, and it may also be possible that events (1) and (2) could occur in either time order.

Handling of these ICEs in estimand definitions for efficacy analyses is described in the relevant parts of Section 7.

Only anti-obesity medications were considered in the protocol as ICEs. If a NASH medication (e.g. resmetirom) was introduced for any participant after starting study treatment, then data after the introduction of such medication will be used in all analysis (i.e. a treatment policy approach will be used in all estimand definitions). This situation is expected to be rare, if it exists at all.

Premature treatment discontinuation will be defined directly from the End of Treatment eCRF page. The date of occurrence of this ICE will be the date of the last dose of study treatment plus 28 days (see also Section 6.1.3). The premature treatment discontinuation ICE is only defined where its occurrence is relevant to estimands and analysis of the primary endpoint time point (Week 48). Specifically, any participant who completed 48 weeks of treatment and subsequently discontinued during the optional treatment extension is not an ICE for premature treatment discontinuation.

The occurrence and date of any death on study will be taken directly from the End of Study eCRF page.

Proprietary confidential information © 2026 Boehringer Ingelheim International GmbH or one or more of its affiliated companies.

Prohibited anti-obesity medication (other than GLP-1R agonist containing medication) will be defined as restricted medication listed in CTP section 4.2.2.1 under “Anti-obesity medications”, that is, these medications are to be selected by BI-defined restricted medication BIcDQ GROUPCD = "62306201" (Restricted medications 1404 studies - Anti-obesity medications (BITS)), using both narrow and broad terms. The full list of BI restricted medications corresponding to this BIcDQ group will be displayed in a table in the CTR.

Prohibited medication containing GLP-1R agonists will be defined as restricted medication listed in CTP section 4.2.2.1 under “GLP-1R agonists”, that is, these medications are to be selected by the standardised drug grouping (SDG) code GROUPCD = "0000000168" (GLP-1R agonists used in diabetes), using both narrow and broad terms.

In both cases above, the date of occurrence of the prohibited medication ICE is the date on which the first qualifying medication was started.

A frequency table will be produced by treatment group which summarises the number and percentage of participants who had experienced each type of ICE at any time during the planned treatment period.

A cumulative incidence plot using Kaplan-Meier estimates will be presented by treatment group of time to first occurrence of non-GLP-1 and GLP-1 ICEs (separate plots for each). The graph will be truncated at Week 48 (i.e. any events which would have met the ICE criterion after this time point, notably during the optional treatment extension period, will not be considered, and participants will be censored at Week 48 in any such cases). Any event meeting the ICE criterion prior to Week 48 will be included as an uncensored event regardless of whether the participant remained on study treatment. Participants without the ICE prior to Week 48 will be censored at the earliest of last contact date and Week 48.

A cumulative incidence plot using Kaplan-Meier estimates will also be presented by treatment group of time to early treatment discontinuation (see also disposition section) to evaluate the extent to which this particular ICE is the dominant one with regard to both its occurrence and its timing.

6.4 SUBJECT SETS ANALYSED

The following analysis sets will be defined:

<u>Screened Set</u>

The screened set (SS) includes all participants for whom a signed informed consent is available at the time of DBL. It will be used only for display of participant disposition.

<u>Randomised Set</u>

The randomised set (RS) includes all screened participants who were randomised in the trial, regardless of whether any study drug was taken. It will be used only for display of participant disposition and IPDs.

Proprietary confidential information © 2026 Boehringer Ingelheim International GmbH or one or more of its affiliated companies.

Treated Set

The treated set (TS) is defined as all randomised participants who received at least one dose of study treatment.

The TS will be used for presentation of demographic/baseline data, concomitant medications/diagnoses, treatment exposure, efficacy and safety.

It is not expected on the database that the situation in which a participant could be treated without randomisation will exist. Documentation is in place by the Interactive Response Technology (IRT) vendor, which states that, should any participant be given treatment at the site in error without randomisation, such a participant will be subsequently randomised to a treatment which is compatible with the one given in error.

Per Protocol Set

A per protocol set (PPS) will not be defined in this study.

[redacted]

The table below summarises the analysis sets and their main intended purpose:

Table 5 Subject sets analysed

**Data type**	**Analysis set**			
	SS	RS	TS	[redacted]
Disposition	x	x	x	
Demographic and baseline characteristics			x	
Concomitant diseases and medications			x	
Exposure to study drug			x	
Efficacy endpoints (primary, secondary, further)			x	
[redacted]			[redacted]	

Proprietary confidential information © 2026 Boehringer Ingelheim International GmbH or one or more of its affiliated companies.

**Data type**	**Analysis set**			
Adverse events			x	
Vital signs			x	
Laboratory safety data			x	
12-lead ECG			x	
Other safety			x	
[redacted]				[redacted]

[redacted]

Proprietary confidential information © 2026 Boehringer Ingelheim International GmbH or one or more of its affiliated companies.

[redacted]

6.6 HANDLING OF MISSING DATA AND OUTLIERS

Efficacy

Descriptive summaries of efficacy endpoints make no special provision for missing data, i.e. it is ignored (which is implicitly missing completely at random (MCAR) in the summary statistics or frequency table which is presented. The approach taken in those endpoints for which statistical modelling is additionally performed is determined by the estimand used, and is summarised below. Full analysis details are given in the relevant parts of Section 7.

For the treatment regimen estimand, two different multiple imputation approaches will be used to handle missing data. In retrieved dropout MI analysis, the basic idea is that imputation of missing data at each time point considers whether the ICE has occurred (or not) and then imputes from participants within the same treatment group for whom the ICE has occurred (or not) and for whom data are available at that time point. In follow the reference MI analysis (also known as copy reference MI analysis), a more conservative approach is used, in which it

Proprietary confidential information © 2026 Boehringer Ingelheim International GmbH or one or more of its affiliated companies.

is assumed that participants on survodutide with missing data after the ICE has occurred follow a trajectory which slowly returns to that of placebo participants, and imputation is therefore done only using the available data for the placebo group. In both types of analysis, any imputed values will be overwritten by any actual values available after the ICE has occurred.

For the efficacy estimand, continuous endpoints will be analysed using MMRM, which handles missing values implicitly using a missing at random (MAR) assumption. MAR means that a participant with missing values is assumed to have behaved like a similar participant within the same treatment group (where “similar” means with regard to both baseline covariates in the model and to the observed trajectory of post-baseline values prior to the missing observation).

For the efficacy estimand, binary responder endpoints are derived from continuous variables. In these cases, an MI analysis will be performed in which imputation of missing data also uses an MAR assumption.

In both types of analysis using the efficacy estimand, any actual values available after the ICE has occurred will be set to missing, and treated in exactly the same way as genuinely missing values. Analyses which make more conservative assumptions than MAR will not be performed for the efficacy estimand.

For clarity, given especially earlier project precedent: for binary responder endpoints, no analysis will be performed in which simple imputation of missing values as non-responders will be performed.

For certain endpoint definitions in the relevant sections, it is planned to apply additional data selection criteria (e.g. based on fasting requirement or technical parameters of imaging procedures, where these data are available) in order to minimise the possibility that outliers or other implausible data are used for analysis. No further exclusion of possible outliers based on the values themselves is proposed.

[redacted]

Other

A completely missing concomitant medication start date will be assumed to mean present at screening. If only the month and year are available for the AE/concomitant medication start date, the first day of the month will be assumed. If only the year is available, then 1st January of that year will be assumed. In the case of AE start dates (only), if the stated imputation would make the AE become pre-treatment when this was not known from the incomplete date, it will be assumed instead that the AE started on the first day of treatment.

It is not expected to have a completely missing or partially missing date of first administration of IMP. A partially missing date of last administration of IMP will be imputed based on rules which generate a last possible date in the respective trial period. If an imputed date of last

Proprietary confidential information © 2026 Boehringer Ingelheim International GmbH or one or more of its affiliated companies.

administration of IMP is later than the date of End of Study or date of death, then the date of last administration of IMP will be set to End of Study date or death date, as applicable.

6.7 BASELINE, TIME WINDOWS AND CALCULATED VISITS

Definition of baseline

In general, baseline is defined as the last non-missing measurement on or prior to the first administration of study medication. In the case that the first dose of study medication is delayed beyond the date of randomisation, a post-randomisation value can be used as a baseline value provided that it meets the stated requirement.

Where clock times are available (expected to include laboratory safety data, vital signs/body weight, 12-lead ECG, plasma survodutide concentrations and biomarkers) they will be used in addition to dates to define baseline, since it is also expected that the time of first study drug administration will be accurately recorded on the eCRF for all participants.

Unscheduled and repeat assessments are eligible for use as baseline values provided that they meet the above criterion.

For blood pressure measurements, the baseline definition will make the additional requirement that the data records are pre-dose, although additional post-dose measurements are not expected according to the protocol. In practice, this will also be done using the clock times as well as the date. Three pre-dose measurements are expected to be captured at the baseline visit (Visit 2); the mean of these 3 measurements (or the mean of the available measurements if fewer) will be used as baseline. In any situation in which all of the measurements which are available at Visit 2 are clearly identified as post-dose and not pre-dose as expected, Visit 2 will not be used as baseline; the mean of the 3 measurements (or those available if fewer) at the screening visit (Visit 1) will be used as baseline instead.

For 12-lead ECG, if a screening value needs to be used for baseline, it will be the average of all available triplicate screening ECG measurements (see Section 5.4.1 for derivation details).

MRI-PDFF and [redacted] procedures are only scheduled to be performed at screening, and not also at the randomisation visit. Therefore, the baseline value is the screening value in these cases.

For laboratory variables which are supposed to be measured in a fasted state ([redacted], HOMA-IR, [redacted]), of the visits relevant to baseline definition, only Visit 2 is scheduled to be fasted according to the protocol. If the Visit 2 value is missing, then baseline is missing for these variables. Screening (Visit 1) values will not be used, even in the unlikely event that these data are available, since this visit is not scheduled to be fasted.

For endpoints derived across multiple data sources which involve imaging assessments (e.g. [redacted]), then the 14-day rule specified in Section 5.3 will be used. In the unlikely event that an on-treatment value might otherwise be selected using this 14-day rule, it will be

Proprietary confidential information © 2026 Boehringer Ingelheim International GmbH or one or more of its affiliated companies.

truncated to the date of first administration of study medication in the baseline definition. It should also be noted that in many cases (due to the protocol scheduling of imaging procedures at the screening visit only and not also at the randomisation visit, and the possibility of long screening periods spanning several weeks), it is expected in many cases that a laboratory measurement will be selected for this baseline derivation to be sufficiently close to the imaging date, which is not the same as the measurement selected for the regular baseline derivation of laboratory measurements.

Visit windows

All summaries and analysis which are presented by week will use a visit window to classify the data record. This approach allows appropriate classification of visits which may have occurred significantly earlier or later than the protocol assessment schedule, as well as the use of data captured at visits which have no fixed timing (notably the EOT visit). Relative day will be defined for this purpose as *(date of assessment – date of first administration of study medication) + 1.*

Nominal database visit labels will not be used in any summary or analysis by time point, unless otherwise stated.

Prior to applying the visit windows specified below, it will first be determined whether the data record is on-treatment, as defined in Section 6.1.3 for summaries or analysis which require this approach.

Unscheduled and repeat assessments (where relevant/available) are eligible for inclusion in visit windows.

For all variables except 12-lead ECG, if a participant has more than one non-missing value within the same visit window, the value closest to the target day will be selected. If two non-missing values are the same distance from the target day, the earlier of the two values will be selected.

For 12-lead ECG (only), scheduled visits take precedence over unscheduled visits. Once this rule has been applied, the measurement closest to the target day will be used. If there are two observations which have the same difference in days to the planned day (but are not on the same day), the later of the two values will be selected.

For all relevant variables, and for all analyses and summaries in which all data are included (i.e. irrespective of whether on- or off-treatment), if a participant has both an on-treatment and an off-treatment value within the same visit window, the on-treatment value will be selected in preference, even if it is not the value closest to the target day. If there is more than one on-treatment value in this situation, the on-treatment value closest to the target day will be selected.

Derivation of last, minimum and maximum values where relevant in safety analyses will consider all available values, regardless of whether they were selected in the visit window.

Proprietary confidential information © 2026 Boehringer Ingelheim International GmbH or one or more of its affiliated companies.

The derivation of the maximum value of an ECG interval (or worst case of the morphological assessment, if applicable) will consider all on-treatment values (whether or not selected in any visit window; see Section 6.1.3 for definition of on-treatment).

The following different situations are envisaged, which are described in the following tables:

- Visit windows for variables scheduled at all clinic visits (with/without remote visits)
- Visit windows for variables with a sparse clinic visit schedule.
- [redacted]

[redacted]

These visit windows will be used for all variables, including for variables which do not follow the full protocol clinic visit schedule, unless otherwise specified below. This includes e.g. safety laboratory variables which are not scheduled at Week 42, and ECG variables which are not scheduled at either Week 28 or Week 42.

For body weight (and BMI), the above table will be used, with one important adaptation. The Week 48 window will be defined as ≥ 316 days (i.e. with no upper bound), and no Week 52 window will be defined for analysis, regardless of whether the participant entered the optional treatment extension period or not. This is to avoid any possibility that late scheduling issues

Proprietary confidential information © 2026 Boehringer Ingelheim International GmbH or one or more of its affiliated companies.

produce a situation in which the participant would otherwise have a Week 52 body weight value available, but no Week 48 value available for primary endpoint purposes.

Table 7 Visit windows for variables scheduled to be assessed at all clinic and remote visits

**Period**	**Time Point**	**Target Day**	**Visit Window**
Dose escalation	Week 2	15	2-21
	Week 4	29	22-35
	Week 6	43	36-49
	Week 8	57	50-70
	Week 12	85	71-98
	Week 16	113	99-126
	Week 20	141	127-154
	Week 24	169	155-182
Maintenance	Week 28	197	183-210
	Week 32	225	211-238
	Week 36	253	239-273
	Week 42	295	274-315
	Week 48	337	≥ 316
	Week 52	N/A	N/A

The relevant variables are expected to include the C-SSRS questionnaire only, for which additional remote visits are scheduled at Week 2, Week 6 and Week 32.

The above visit windows will, however, be applied only using the relative day, regardless of whether selected records were actually done remotely or not.

The relevant variables are expected to include variables from the imaging procedures (MRI and FibroScan®) only.

Proprietary confidential information © 2026 Boehringer Ingelheim International GmbH or one or more of its affiliated companies.

Table 8 Visit windows for variables with a sparse clinic visit schedule

Period	Time Point	Target Day	Visit Window
Maintenance	Week 28	197	2-266
	Week 48	337	≥ 267

The use of such a wide window for imaging variables at Week 28 to handle the sparse protocol scheduling means that it will display and analyse as "Week 28" any imaging assessments which were done very early, e.g. as part of EOT assessments.

[redacted]

For variables derived across multiple data sources which involve an imaging component (e.g. FAST score), the same visit windows will be applied as for the variables from the imaging

Proprietary confidential information © 2026 Boehringer Ingelheim International GmbH or one or more of its affiliated companies.

procedures alone. Determination of relative day for visit window assignment will use the imaging date.

In addition to the above windows, separate outputs including only off-treatment measurements will be presented. Details on this, the analyses of participants from extended treatment periods and off-treatment measurements, as well as picking algorithms, will be given in the “ADS PK/PD/IS Data Transfer Specification For Study Datasets”.

7. PLANNED ANALYSIS

Descriptive statistics for continuous variables will be, unless otherwise stated: N (number of participants with non-missing values), arithmetic mean, standard deviation (SD), minimum, Q1 (lower quartile), median, Q3 (upper quartile) and maximum. Means, medians, other quartiles and SDs will be presented to one more decimal place than the raw data. Minimums and maximums will be presented to the same number of decimal places as the raw data.

For survodutide plasma concentrations, CV (arithmetic coefficient of variation), gMean (geometric mean) and gCV (geometric coefficient of variation) will also be presented. The data format for descriptive statistics of concentrations will be identical with the data format of the respective concentrations.

Tabulations of frequencies for categorical data will include all possible non-missing categories unless otherwise specified (e.g. AE terms), and will display the number of observations in a category as well as the percentage (%) relative to the respective treatment group (unless otherwise specified, all trial participants in the respective analysis set whether the trial participant has non-missing values or not).

The precision for percentages should be one decimal point, unless the denominator is smaller than 100 (in all treatment columns), in which case percentages are given in integer numbers. The category “missing” will be displayed only if there are actually missing values.

Variables which will be analysed on the log-scale will be reported on the original scale in terms of gMean and gSD for descriptive summaries, and in terms of gMean of the respective model estimate and its 95% confidence interval. The relative change from baseline in percentage will be reported as (gMean fold change -1)*100 %. Similarly, the estimates for “relative difference from baseline” and “relative difference from placebo” will be reported as:

Relative difference from baseline: Estimate = (exp(LSMEANS) -1)*100%,

Relative difference from placebo: Estimate = (exp(Difference of LSMEANS) -1)*100%,

with “exp” being the exponential function, and LSMEANS is the respective adjusted mean from the model.

The list of endpoints which will be analysed on the log-scale are listed in Section 10.1.

Proprietary confidential information © 2026 Boehringer Ingelheim International GmbH or one or more of its affiliated companies.

Disposition

The disposition of trial participants will be displayed. This table will present the number of trial participants enrolled, randomised, and treated. The number treated will be further presented separating the dose escalation and maintenance periods.
The number of trial participants who completed or who prematurely discontinued study treatment will be summarised, along with the categorization of the primary reason for treatment discontinuation. The number of trial participants and reasons for prematurely discontinuing treatment will also be displayed separately for the dose escalation and maintenance periods.

The number (%) of trial participants who completed treatment will be further summarised according to whether they participated in the optional 4-week treatment extension or not. This will be determined by the presence/absence of Visit 15 (Week 48) dispensing information on the eCRF.

The number of trial participants who completed or who prematurely withdrew from the study will be summarised, along with the categorization of the primary reason for study withdrawal.

Where shown on the disposition table, the percentages will be of the number of trial participants treated in each treatment group.

A cumulative incidence plot using Kaplan-Meier estimates will be presented by treatment group for time to early treatment discontinuation (defined as *treatment discontinuation date – treatment start date + 1*). In this analysis, participants who did not discontinue treatment early will be censored using the date on which they completed treatment.

The number (%) of trial participants who were randomised to survodutide and who did not reach the planned survodutide dose (6.0 mg) will be summarised by last survodutide dose reached. The frequency of participants in each of the different analysis sets (Section 6.4) will also be tabulated by treatment.

IPDs will be summarised and listed by treatment group for the randomised set.

7.1 DEMOGRAPHIC AND OTHER BASELINE CHARACTERISTICS

Descriptive summaries of the following demographic variables and characteristics measured at baseline will be presented by treatment group on the TS, to include: sex (including also childbearing potential and menopause status for female participants), race and ethnicity, age (years) (continuous and categories), height (cm), weight (kg) (continuous and categories), BMI (kg/m^2) (continuous and categories), systolic BP (mmHg), diastolic BP (mmHg), pulse rate (bpm), country and presence/absence of T2DM (from both the IRT stratification variable and from medical history).

Subgroup categories will be summarised as defined in Section 6.5.

Proprietary confidential information © 2026 Boehringer Ingelheim International GmbH or one or more of its affiliated companies.

The participant history of NASH will be summarised by treatment group on the TS, to include: time since NASH diagnosis, whether the diagnosis is presumed or confirmed, and the further information relevant to each of those 2 choices (see Section 5.4.2).

The participant history of obesity/overweight will be summarised by treatment group on the TS, to include: time since obesity/overweight diagnosis, family history, obesity staging (EOSS) and sleep quality (see Section 5.4.2).

NYHA functional classification and substance use will also be summarised by treatment group on the TS.

7.2 CONCOMITANT DISEASES AND MEDICATION

Only descriptive statistics are planned by treatment group (number and percentage of participants). Summaries of concomitant diseases (medical history and baseline conditions), concomitant medication and concomitant non-drug therapy will be based on the TS.

Baseline conditions, medical history and concomitant non-drug therapies will be coded according to the most recent version of MedDRA at the time of final DBL. This will include coding of trial-specific recording of medical history for the relevant summary table (see below).

Concomitant medications will be coded according to the most recent version of the World Health Organisation (WHO) Drug Dictionary at the time of final DBL.

Relevant baseline conditions and medical history will be summarised descriptively by MedDRA primary system organ class (SOC) and preferred term (PT), which will include coded trial-specific medical history.

In addition, trial-specific medical history will be summarised using reported term from the eCRF page (e.g. Type 2 Diabetes, Diabetic Retinopathy, Diabetic Maculopathy, Dyslipidaemia, Hypertension, etc).

A separate table will be produced of **ongoing** medical history as identified from the specific eCRF question, plus any related items recorded as baseline conditions (which will be assumed to be ongoing in the absence of data capture to identify otherwise).

A medication or non-drug therapy will be considered concomitant to treatment if either of the following applies:

- It is ongoing at the start of study treatment
- The start date occurs between the start of study treatment and 28 days after the end of study treatment inclusive.

A medication or non-drug therapy will be considered prior to treatment if the end date occurs before the start of study treatment.

Proprietary confidential information © 2026 Boehringer Ingelheim International GmbH or one or more of its affiliated companies.

Concomitant medication will be summarised descriptively by Anatomical Therapeutic Chemical classification level 3 (ATC3) and level 5 (ATC5). Summaries will be presented separately for all concomitant medication taken and concomitant medications newly introduced on treatment. Prior medication will not be summarised.

An additional table will be produced of any medications introduced (started or changed) on or after the date of early discontinuation of treatment, for the relevant subset of participants (regardless of whether these new medications were within or outside the 28-day period defined by the REP).

Furthermore, a table will be produced showing 'pre-defined medications of interest at baseline' (which were also ongoing at start of study treatment) by WHO Drug SDGs.

Concomitant non-drug therapy will be summarised by SOC and PT. Prior non-drug therapy will not be summarised.

7.3 TREATMENT COMPLIANCE

Compliance with Diet and Physical Activity

The answers to the following question: "Did the participant follow dietary and physical activity recommendations? (yes/no)" will be presented in a frequency table over time by treatment group using the TS.

Compliance with Study Treatment

Treatment compliance (as defined in Section 5.4.3) will be summarised descriptively by treatment group using the TS.

7.4 PRIMARY OBJECTIVE ANALYSIS

All efficacy analyses will be performed on the TS. Data selection will be determined according to the strategy for handling intercurrent events in the estimand being targeted.

The primary objective analysis is specified in the CTP Section 7.2.3. Details are not repeated here where the CTP statements are already sufficiently clear and comprehensive.

7.4.1 Relative reduction in liver fat content of at least 30% from baseline to Week 48 assessed by MRI-PDFF (yes/no)

7.4.1.1 Main analysis

Treatment regimen estimand

In all relevant descriptions of analysis using the treatment regimen estimand, a **retrieved dropout** is defined as a participant who prematurely discontinued study treatment (including

Proprietary confidential information © 2026 Boehringer Ingelheim International GmbH or one or more of its affiliated companies.

the 28-day REP, as defined in Section 6.3) and who subsequently provided an off-treatment endpoint value at Week 48 (according to the definition of Week 48 in Section 6.7).

In all relevant descriptions of analysis using the treatment regimen estimand, a **completer** is defined as a participant who provided an on-treatment endpoint value at Week 48. This could therefore include either a participant who did not discontinue study treatment at all, or a participant who discontinued study treatment less than 28 days before the Week 48 assessment.

The treatment regimen estimand is defined in the CTP Section 7.2.2. Intercurrent events are also summarised in Section 6.3. The main (co-primary) analysis estimates the treatment effect which includes the effects of all of the pre-defined ICEs other than death. Essentially a treatment policy strategy will be applied to all ICEs except for death. If any death occurs during the study then a hypothetical strategy will be used (i.e. as if death had not occurred).

Frequency tables will be produced of the liver fat content (MRI-PDFF) responder endpoint at Week 48, using both on- and off-treatment data. These tables will include the number (%) of responders and non-responders respectively. The table will be produced overall, and also separately by whether participants prematurely discontinued study treatment (including the 28-day REP) or not, i.e. in which those with non-missing values are the retrieved dropouts and completers respectively.

Descriptive summaries of the underlying continuous variable liver fat content from MRI-PDFF will be produced over time, using both on- and off-treatment data, and including absolute and percentage changes from baseline. The table will be produced overall, and also by whether participants prematurely discontinued study treatment (including the 28-day REP) or not.

For the main (co-primary) analysis targeting the treatment regimen estimand, missing values of the continuous liver fat content (MRI-PDFF) variable will be imputed using a retrieved dropout multiple imputation approach. In this approach, missing data after the ICE of premature treatment discontinuation will be imputed (from within the same treatment group) using an imputation model with a pattern indicator variable to denote the timing of treatment discontinuation, whilst missing data before the ICE of premature treatment discontinuation will be imputed using a standard MI-MAR approach.

In the event that there are insufficient observed data at Week 48 after the ICE of premature treatment discontinuation, then the primary analysis will instead be the reference-based multiple imputation (follow the reference) analysis described in Section 7.4.1.2. For this purpose, "insufficient observed data" is defined as one or both treatment groups in which fewer than 40% of participants who experienced the ICE of premature treatment discontinuation provide endpoint data at Week 48. Both analyses will still be done if both are possible.

[redacted]

Proprietary confidential information © 2026 Boehringer Ingelheim International GmbH or one or more of its affiliated companies.

[redacted]

Proprietary confidential information © 2026 Boehringer Ingelheim International GmbH or one or more of its affiliated companies.

[redacted]

Proprietary confidential information © 2026 Boehringer Ingelheim International GmbH or one or more of its affiliated companies.

[redacted]

7.4.1.2 Sensitivity analysis

Follow the reference multiple imputation analysis

As a sensitivity analysis targeting the treatment regimen estimand, missing values of the continuous liver fat content (MRI-PDFF) variable will be multiply imputed using a reference-based multiple imputation (follow the reference) approach, which makes a conservative missing not at random (MNAR) assumption to handling missing data after the presence of the ICE for treatment discontinuation. This assumes that participants in the active survodutide group lose any treatment effect such that their missing observations after the treatment discontinuation ICE will then follow a trajectory which slowly returns to that of the placebo (reference) group. Therefore all imputation after the treatment discontinuation ICE in both groups will be based solely on the placebo distribution.

If the retrieved dropout analysis specified in Section 7.4.1.1 is not selected as the primary analysis (because the 40% criterion defined in that section is not met), then the follow the reference analysis will be the primary analysis of the liver fat content (MRI-PDFF) co-primary endpoint. This is regardless of whether the retrieved dropout analysis specified in

Proprietary confidential information © 2026 Boehringer Ingelheim International GmbH or one or more of its affiliated companies.

Section 7.4.2.1 for the other body weight co-primary endpoint is selected as the primary analysis using a similar criterion. In other words, the 40% criterion will be applied independently to each co-primary endpoint.

[redacted]

Proprietary confidential information © 2026 Boehringer Ingelheim International GmbH or one or more of its affiliated companies.

[redacted]

Proprietary confidential information © 2026 Boehringer Ingelheim International GmbH or one or more of its affiliated companies.

[redacted]

Proprietary confidential information © 2026 Boehringer Ingelheim International GmbH or one or more of its affiliated companies.

[redacted]

Proprietary confidential information © 2026 Boehringer Ingelheim International GmbH or one or more of its affiliated companies.

7.4.2 Relative change in body weight from baseline to Week 48 (%)

7.4.2.1 Main analysis

Treatment regimen estimand

In all relevant descriptions of analysis using the treatment regimen estimand, a **retrieved dropout** and a **completer** are as defined in Section 7.4.1.1.

The treatment regimen estimand is defined in the CTP Section 7.2.2. Intercurrent events are also summarised in Section 6.3. The main (co-primary) analysis estimates the treatment effect which includes the effects of the all of the pre-defined ICEs other than death. Essentially a treatment policy strategy will be applied to all ICEs except for death. This includes any situation in which a participant discontinued treatment early due to pregnancy, but continued to be followed up for clinic measurement of body weight. If any death occurs during the study then a hypothetical strategy will be used (i.e. as if death had not occurred).

Descriptive summaries of body weight will be produced over time, using both on- and off-treatment data, and including absolute and percentage changes from baseline. The table will be produced overall, and also by whether participants prematurely discontinued study treatment (including the 28-day REP) or not.

Mean percentage change in body weight from baseline will be summarised graphically over time by missing data pattern. A separate graph for each treatment group will be created, displaying the following subgroups:

- Completers, as defined at the start of Section 7.4
- Retrieved dropouts, as also defined at the start of Section 7.4
- Participants with missing data patterns defined by whether they had their last non-missing value at Week X (where X < 48), and irrespective of whether this was on- or off-treatment.

The number of participants in each of the subgroups will be displayed on the graph.

For the main (co-primary) analysis targeting the treatment regimen estimand, missing values of the continuous body weight variable will be imputed using a retrieved dropout multiple imputation approach. In this approach, missing data after the ICE of premature treatment discontinuation will be imputed (from within the same treatment group) using an imputation model with a pattern indicator variable, whilst missing data before the ICE of premature treatment discontinuation will be imputed using a standard MI-MAR approach.

In the event that there are insufficient observed data at Week 48 after the ICE of premature treatment discontinuation, then the primary analysis will instead be the reference-based multiple imputation (follow the reference) analysis described in Section 7.4.2.2. For this purpose, “insufficient observed data” is defined as one or both treatment groups in which fewer than 40% of participants who experienced the ICE of premature treatment discontinuation provide endpoint data at Week 48. Both analyses will still be done if both are possible.

Proprietary confidential information © 2026 Boehringer Ingelheim International GmbH or one or more of its affiliated companies.

[redacted]

Proprietary confidential information © 2026 Boehringer Ingelheim International GmbH or one or more of its affiliated companies.

[redacted]

First off-treatment		Starting pattern
**Analysis** [redacted]	[redacted]	

[redacted]

Proprietary confidential information © 2026 Boehringer Ingelheim International GmbH or one or more of its affiliated companies.

[redacted]

7.4.2.2 Sensitivity analysis

Follow-reference multiple imputation analysis

As a sensitivity analysis targeting the treatment regimen estimand, missing values of the continuous body weight variable will be multiply imputed using a reference-based multiple imputation (follow the reference) approach, which makes a conservative MNAR assumption to handling missing data after the presence of the ICE for treatment discontinuation, similarly to what is described in Section 7.4.1.2.

If the retrieved dropout analysis specified in Section 7.4.2.1 is not selected as the primary analysis (because the 40% criterion defined in that section is not met), then the follow the reference analysis will be the primary analysis of the body weight co-primary endpoint. This is regardless of whether the retrieved dropout analysis specified in Section 7.4.1.1 for the other liver fat content (MRI-PDFF) co-primary endpoint is selected as the primary analysis using a similar criterion. In other words, the 40% criterion will be applied independently to each co-primary endpoint.

[redacted]

Proprietary confidential information © 2026 Boehringer Ingelheim International GmbH or one or more of its affiliated companies.

[redacted]

Proprietary confidential information © 2026 Boehringer Ingelheim International GmbH or one or more of its affiliated companies.

[redacted]

Proprietary confidential information © 2026 Boehringer Ingelheim International GmbH or one or more of its affiliated companies.

[redacted]

Proprietary confidential information © 2026 Boehringer Ingelheim International GmbH or one or more of its affiliated companies.

7.5 SECONDARY OBJECTIVE ANALYSIS

All efficacy analyses will be performed on the TS. Data selection will be determined according to the strategy for handling intercurrent events in the estimand being targeted.

7.5.1 Key secondary objective analysis

This section is not applicable as no key secondary endpoint has been specified in the protocol.

7.5.2 Secondary objective analysis

<u>Secondary endpoints from MRI or MRE</u>

Liver fat content (MRI-PDFF)

Descriptive summaries of continuous liver fat content from MRI-PDFF over time, and including both absolute and percentage changes from baseline, were already specified in Section 7.4.1.1, for all data and for on-treatment data, respectively.

Analysis of the continuous liver fat content (MRI-PDFF) secondary endpoints will be performed using both of the treatment regimen and efficacy estimands.

For analyses targeting the treatment regimen estimand, missing values of the continuous liver fat content (MRI-PDFF) variable will be multiply imputed using both a retrieved dropout approach and a reference-based (follow the reference) approach. Specifically, the same 1000 completely imputed datasets from Step 2 of Section 7.4.1.1 and Section 7.4.1.2, respectively, will be used. Step 3 in those sections will be replaced by derivation of the continuous absolute or relative change variable, as applicable. In Step 4, each of the 1000 completely imputed datasets will then be analysed using an ANCOVA on each of the two continuous endpoints directly, with treatment group and presence/absence of T2DM as factors, and baseline liver fat content (assessed by MRI-PDFF) as a continuous linear covariate. The model will be used to estimate the difference in means and its 95% CI and corresponding p-value for survodutide versus placebo. In Step 5, the estimate of difference in means and its 95% CI and corresponding p-value will be obtained from the 1000 imputed datasets using Rubin's rules, and displayed in a suitable summary table. The estimated mean in each treatment group and its 95% CI will also be displayed.

For analysis targeting the efficacy estimand, missing values of each of the continuous liver fat content (MRI-PDFF) endpoints will be implicitly handled using an MMRM, which makes a MAR assumption. Values recorded after premature discontinuation of study treatment (including the 28-day REP, as defined in Section 6.3) will be considered missing for this purpose. Further details regarding exclusion of data after the introduction of prohibited anti-obesity medications are given in CTP Table 13. Additional analyses which make other missing data assumptions will not be performed for this estimand. The MMRM will include fixed categorical effects for treatment at each week and presence of T2DM (yes/no), and the fixed continuous (linear) effect of baseline liver fat content (%) at each week. The Kenward-Roger approximation will be used to estimate the denominator degrees of freedom and adjust standard errors. Unstructured covariance will be assumed to model the relationship between pairs of measurements taken at different weeks on the same participant. Details in the event that the model fails to converge are as per those in Section 7.4.2.4.

Proprietary confidential information © 2026 Boehringer Ingelheim International GmbH or one or more of its affiliated companies.

Adjusted means will be calculated from the MMRM using the observed margins approach, in which the contribution of model factors to the estimate is weighted proportionally to the presence of these factors in the data.

A summary table will be produced of the MMRM results at each time point, including adjusted means and their standard errors for each treatment group, and including the adjusted mean difference from placebo for survodutide and its 95% confidence interval. Adjusted means from the MMRM will also be displayed graphically over time to assess the onset of effect over time.

An additional analysis will be performed on the efficacy estimand in which measured liver fat content (MRI-PDFF) values will be log-transformed prior to fitting the MMRM. Measured values at each scheduled week will be log-transformed prior to fitting the MMRM. Details for the MMRM are otherwise similar to those specified above. The dependent variable will then be the difference in log-transformed values at each scheduled week and the log-transformed value at baseline.

A summary table will be produced of the MMRM results at each time point. Adjusted means and their 95% CIs for each treatment group will be back-transformed by exponentiation before reporting (this gives estimates of the adjusted fold changes for post-baseline versus baseline for each treatment group). The adjusted mean difference (survodutide minus placebo) and its 95% CI will also be back-transformed by exponentiation before reporting (this gives an estimate of the ratio survodutide/placebo of the fold changes). The MMRM results will also be displayed graphically over time.

cT1 Levels

Descriptive summaries of continuous cT1 over time, and including both absolute and percentage changes from baseline, will be produced for all data and for on-treatment data, respectively.

Frequency tables will be produced of the cT1 responder secondary endpoint at Week 48 (as defined in Section 5.2.2), using all data and on-treatment data, respectively.

Analysis of the binary cT1 responder secondary endpoint at Week 48 (as defined in Section 5.2.2) will be performed using the efficacy estimand only.

For analysis targeting the efficacy estimand, missing values of the cT1 responder variable will be multiply imputed using a MAR assumption. Values recorded after premature discontinuation of study treatment (including the 28-day REP, as defined in Section 6.3) will be considered missing for this purpose. Further details regarding exclusion of data after the introduction of prohibited anti-obesity medications are given in CTP Table 13. Additional analyses which make other missing data assumptions will not be performed for this estimand.

The MI-MAR analysis will proceed according to the same steps as described for the similar analysis of the liver fat content (MRI-PDFF) responder endpoint on the efficacy estimand in Section 7.4.1.4, with differences as noted here. All MI steps will use the absolute value of the underlying continuous cT1 variable (measured in ms). Step 2 will use baseline cT1 instead of

Proprietary confidential information © 2026 Boehringer Ingelheim International GmbH or one or more of its affiliated companies.

baseline liver fat content (MRI-PDFF) (and the MI will also impute any missing baseline values which might occur here, although a similar situation is not expected for baseline liver fat content (MRI-PDFF)). The FCS method is not expected to need consideration of out of range cT1 values. Steps 3-5 will be the same, except for the derivation of the relevant endpoint in Step 3.

Liver stiffness (MRE)

Descriptive summaries of continuous liver stiffness from MRE over time, and including both absolute and percentage changes from baseline, will be produced for all data and for on-treatment data, respectively.

Analysis of the continuous liver stiffness secondary endpoints will be performed using the efficacy estimand only.

Missing values of each of the continuous liver stiffness variables will be implicitly handled using an MMRM, which makes a MAR assumption. Values recorded after premature discontinuation of study treatment (including the 28-day REP, as defined in Section 6.3) will be considered missing for this purpose. Further details regarding exclusion of data after the introduction of prohibited anti-obesity medications are given in CTP Table 13. Additional analyses which make other missing data assumptions will not be performed for this estimand. Details for the MMRM and presentation of the results are as specified above for the continuous liver fat content (MRI-PDFF) secondary endpoints, except that baseline liver stiffness (kPa) is used in the model.

Liver volume (MRI)

Descriptive summaries of continuous liver volume from MRI over time, and including both absolute and percentage changes from baseline, will be produced for all data and for on-treatment data, respectively.

Analysis of the continuous liver volume secondary endpoints will be performed using the efficacy estimand only.

Missing values of each of the continuous liver volume variables will be implicitly handled using an MMRM, which makes a MAR assumption. Values recorded after premature discontinuation of study treatment (including the 28-day REP, as defined in Section 6.3) will be considered missing for this purpose. Further details regarding exclusion of data after the introduction of prohibited anti-obesity medications are given in CTP Table 13. Additional analyses which make other missing data assumptions will not be performed for this estimand. Details for the MMRM and presentation of the results are as specified above for the continuous liver fat content (MRI-PDFF) secondary endpoints, except that baseline liver volume (mL) is used in the model.

Other secondary endpoints

ALT and HOMA-IR

Descriptive summaries of ALT and HOMA-IR over time, including absolute changes from baseline, and relative changes from baseline expressed using gMean and gSD, will be

Proprietary confidential information © 2026 Boehringer Ingelheim International GmbH or one or more of its affiliated companies.

produced for all data and for on-treatment data, respectively. The descriptive summaries for HOMA-IR will be presented by T2DM status (yes/no) as well as overall.

Analysis of the ALT and HOMA-IR secondary endpoints will be performed using the efficacy estimand only. Missing values will be implicitly handled using an MMRM, which makes a MAR assumption. Values recorded after premature discontinuation of study treatment (including the 28-day REP, as defined in Section 6.3) will be considered missing for this purpose. Further details regarding exclusion of data after the introduction of prohibited anti-obesity medications are given in CTP Table 13. Additional analyses which make other missing data assumptions will not be performed for this estimand. Measured values at each scheduled week will be log-transformed prior to fitting the MMRM. Details for the MMRM are otherwise similar to those specified above for the continuous liver fat content (MRI-PDFF) secondary endpoints, except that baseline ALT (U/L) and baseline HOMA-IR are used in the respective models, and these are also log-transformed prior to fitting them. The dependent variable will then be the difference in log-transformed values at each scheduled week and the log-transformed value at baseline.

A summary table will be produced of the MMRM results at each time point. Adjusted means and their 95% CIs for each treatment group will be back-transformed by exponentiation before reporting (this gives estimates of the adjusted fold changes for post-baseline versus baseline for each treatment group). The adjusted mean difference (survodutide minus placebo) and its 95% CI will also be back-transformed by exponentiation before reporting (this gives an estimate of the ratio survodutide/placebo of the fold changes). The MMRM results will also be displayed graphically over time.

Waist circumference

Descriptive summaries of waist circumference over time, and including both absolute and percentage changes from baseline, will be produced for all data and for on-treatment data, respectively.

Analysis of the waist circumference secondary endpoints will be performed using the efficacy estimand only. Missing values will be implicitly handled using an MMRM, which makes a MAR assumption. Values recorded after premature discontinuation of study treatment (including the 28-day REP, as defined in Section 6.3) will be considered missing for this purpose. Further details regarding exclusion of data after the introduction of prohibited anti-obesity medications are given in CTP Table 13. Additional analyses which make other missing data assumptions will not be performed for this estimand. Details for the MMRM and presentation of the results are as specified above for the continuous liver fat content (MRI-PDFF) secondary endpoints, except that baseline waist circumference (cm) is used in the model.

Proprietary confidential information © 2026 Boehringer Ingelheim International GmbH or one or more of its affiliated companies.

7.6 FURTHER OBJECTIVE ANALYSIS

Further endpoints from MRI or MRE

Liver fat content (MRI-PDFF)

Descriptive summaries of continuous liver fat content from MRI-PDFF over time, and including both absolute and percentage changes from baseline, were already specified in Section 7.4.1.1, for all data and for on-treatment data, respectively.

Analysis of the continuous liver fat content (MRI-PDFF) further endpoints will be performed using both of the treatment regimen and efficacy estimands.

For analyses targeting the treatment regimen estimand, missing values of the continuous liver fat content (MRI-PDFF) variable will be multiply imputed using both a retrieved dropout approach and a reference-based (follow the reference) approach. Specifically, the same 1000 completely imputed datasets from Step 2 of Section 7.4.1.1 and Section 7.4.1.2, respectively, will be used. Step 3 in those sections will be replaced by derivation of the continuous absolute or relative change variable, as applicable. Steps 4-5 will proceed similarly to what is described for the continuous MRI-PDFF secondary endpoints in Section 7.5.2.

For analysis targeting the efficacy estimand, missing values of each of the continuous liver fat content (MRI-PDFF) endpoints will be implicitly handled using an MMRM, which makes a MAR assumption. Values recorded after premature discontinuation of study treatment (including the 28-day REP, as defined in Section 6.3) will be considered missing for this purpose. Further details regarding exclusion of data after the introduction of prohibited anti-obesity medications are given in CTP Table 13. Details of the MMRM will be the same as those described for the continuous liver fat content (MRI-PDFF) secondary endpoints in Section 7.5.2.

An additional analysis will be performed on the efficacy estimand in which measured liver fat content (MRI-PDFF) values will be log-transformed prior to fitting the MMRM. Again, details of the MMRM will be the same as those described in Section 7.5.2.

Frequency tables will be produced of the liver fat content (MRI-PDFF) responder further endpoint at Week 28 and the three liver fat content (MRI-PDFF) responder further endpoints at Week 48 (all as defined in Section 5.3), using all data and on-treatment data, respectively.

Analysis of each of the four binary liver fat content (MRI-PDFF) responder further endpoints will be performed using both of the treatment regimen and efficacy estimands.

For analyses targeting the treatment regimen estimand, missing values of each of the liver fat content (MRI-PDFF) responder variables will be multiply imputed using both a retrieved dropout approach and a reference-based (follow the reference) approach. Specifically, the same 1000 completely imputed datasets from Step 2 of Section 7.4.1.1 and Section 7.4.1.2, respectively, will be used. Step 3 in those sections will be replaced by derivation of the relevant liver fat content (MRI-PDFF) responder variable. Steps 4-5 will proceed similarly to what is described for the liver fat content (MRI-PDFF) co-primary endpoint in Section 7.4.1.1.

Proprietary confidential information © 2026 Boehringer Ingelheim International GmbH or one or more of its affiliated companies.

For analysis targeting the efficacy estimand, missing values of each of the liver fat content (MRI-PDFF) responder variables will be multiply imputed using a MAR assumption. Values recorded after premature discontinuation of study treatment (including the 28-day REP, as defined in Section 6.3) will be considered missing for this purpose. Further details regarding exclusion of data after the introduction of prohibited anti-obesity medications are given in CTP Table 13. Specifically, the same 1000 completely imputed datasets from Step 2 of Section 7.4.1.4 will be used. Steps 3-5 will be the same, except for the derivation of the relevant endpoint in Step 3.

cT1 Levels

Descriptive summaries of continuous cT1 over time, and including both absolute and percentage changes from baseline, were already specified in Section 7.5.2, for all data and for on-treatment data, respectively.

Analysis of the continuous cT1 further endpoints will be performed using the efficacy estimand only.

Missing values of each of the continuous cT1 variables will be implicitly handled using an MMRM, which makes a MAR assumption. Values recorded after premature discontinuation of study treatment (including the 28-day REP, as defined in Section 6.3) will be considered missing for this purpose. Further details regarding exclusion of data after the introduction of prohibited anti-obesity medications are given in CTP Table 13. Details of the MMRM and presentation of the results will be similar to those described for the continuous liver fat content (MRI-PDFF) secondary endpoints in Section 7.5.2, except that the model will use baseline cT1 instead of baseline liver fat content (MRI-PDFF).

Liver stiffness (MRE)

Descriptive summaries of continuous liver stiffness from MRE over time, and including both absolute and percentage changes from baseline, were already specified in Section 7.5.2, for all data and for on-treatment data, respectively.

Analysis of the continuous liver stiffness further endpoints will be performed using the efficacy estimand only.

Missing values of each of the continuous liver stiffness variables will be implicitly handled using an MMRM, which makes a MAR assumption. Values recorded after premature discontinuation of study treatment (including the 28-day REP, as defined in Section 6.3) will be considered missing for this purpose. Further details regarding exclusion of data after the introduction of prohibited anti-obesity medications are given in CTP Table 13. Details of the MMRM and presentation of the results will be similar to those described for the continuous liver fat content (MRI-PDFF) secondary endpoints in Section 7.5.2, except that the model will use baseline liver stiffness (MRE) instead of baseline liver fat content (MRI-PDFF).

Proprietary confidential information © 2026 Boehringer Ingelheim International GmbH or one or more of its affiliated companies.

Liver volume (MRI)

Descriptive summaries of continuous liver volume from MRI over time, and including both absolute and percentage changes from baseline, were already specified in Section 7.5.2, for all data and for on-treatment data, respectively.

Analysis of the continuous liver volume further endpoints will be performed using the efficacy estimand only.

Missing values of each of the continuous liver volume variables will be implicitly handled using an MMRM, which makes a MAR assumption. Values recorded after premature discontinuation of study treatment (including the 28-day REP, as defined in Section 6.3) will be considered missing for this purpose. Further details regarding exclusion of data after the introduction of prohibited anti-obesity medications are given in CTP Table 13. Details of the MMRM and presentation of the results will be similar to those described for the continuous liver fat content (MRI-PDFF) secondary endpoints in Section 7.5.2, except that the model will use baseline liver volume instead of baseline liver fat content (MRI-PDFF).

Further endpoints from clinic measurements

Body weight and BMI

Descriptive summaries of continuous body weight over time, including absolute change from baseline, were already specified in Section 7.4.2.1, for all data and for on-treatment data, respectively. Descriptive summaries of BMI over time will be produced similarly.

Analysis of the continuous body weight and BMI further endpoints will be performed using both of the treatment regimen and efficacy estimands.

For analyses targeting the treatment regimen estimand, missing values of the continuous body weight and BMI variables will be multiply imputed using both a retrieved dropout approach and a reference-based (follow the reference) approach. Specifically, the same 1000 completely imputed datasets from Step 2 of Section 7.4.2.1 and Section 7.4.2.2, respectively, will be used. Step 3 in those sections will be replaced by derivation of the continuous absolute change variables (in the case of BMI, this will then also be divided by the square of the height at screening). Steps 4-5 will proceed similarly to what is described for the continuous liver fat content (MRI-PDFF) secondary endpoints in Section 7.5.2, except that baseline liver fat content (MRI-PDFF) is replaced by baseline body weight or baseline BMI, as applicable.

For analyses targeting the efficacy estimand, missing values of the continuous body weight and BMI variables will be implicitly handled using an MMRM, which makes a MAR assumption. Values recorded after premature discontinuation of study treatment (including the 28-day REP, as defined in Section 6.3) will be considered missing for this purpose. Further details regarding exclusion of data after the introduction of prohibited anti-obesity medications are given in CTP Table 13. Details of the MMRM and presentation of the results will be similar to those described for the continuous liver fat content (MRI-PDFF) secondary endpoints in Section 7.5.2, except that baseline liver fat content (MRI-PDFF) is replaced by baseline body weight or baseline BMI, as applicable.

Proprietary confidential information © 2026 Boehringer Ingelheim International GmbH or one or more of its affiliated companies.

Frequency tables will be produced of the various body weight responder endpoints at Week 48, using all data and on-treatment data, respectively.

No further analysis of the various body weight responder endpoints will be performed.

Blood pressure

Descriptive summaries of systolic and diastolic blood pressure will be produced over time, using on-treatment data only, and including absolute change from baseline.

Analysis of the systolic and diastolic BP further endpoints will be performed using the efficacy estimand only.

Missing values of the blood pressure variables will be implicitly handled using an MMRM, which makes a MAR assumption. Values recorded after premature discontinuation of study treatment (including the 28-day REP, as defined in Section 6.3) will be considered missing for this purpose. Details of the MMRM and presentation of the results will be similar to those described for the continuous liver fat content (MRI-PDFF) secondary endpoints in Section 7.5.2, except that baseline liver fat content (MRI-PDFF) is replaced by baseline systolic BP or baseline diastolic BP, as applicable.

<u>Further endpoints from central laboratory data</u>

Analysis of the endpoints in this section will be performed using the efficacy estimand only.

Descriptive summaries of the following variables will be produced over time, using on-treatment data only, and including absolute change from baseline: FPG, HbA1c (both units), [redacted], ELF score, [redacted], AST, [redacted] uric acid. The descriptive summaries for FPG and HbA1c will be presented by T2DM status (yes/no) as well as overall.

Missing values of the variables listed above will be implicitly handled using an MMRM (on untransformed values), which makes a MAR assumption. Values recorded after premature discontinuation of study treatment ([redacted]) will be considered missing for this purpose. Details of the MMRM and presentation of the results will be similar to those described for the continuous liver fat content (MRI-PDFF) secondary endpoints in Section 7.5.2, except that baseline liver fat content (MRI-PDFF) is replaced by the relevant baseline value from the above list.

Descriptive summaries of the following variables will be produced over time, using on-treatment data only, and including relative changes from baseline expressed using gMean and gSD: Total cholesterol, HDL-C, LDL-C, VLDL-C, triglycerides, [redacted], [redacted] AST, [redacted], uric acid. The descriptive summaries for FPI and [redacted] will be presented by T2DM status (yes/no) as well as overall.

Missing values of the variables listed above will be implicitly handled using an MMRM , which makes a MAR assumption. Values recorded after premature discontinuation of study treatment ([redacted]) will be considered missing for

Proprietary confidential information © 2026 Boehringer Ingelheim International GmbH or one or more of its affiliated companies.

this purpose. Measured values at each scheduled week will be log-transformed prior to fitting the MMRM. Details of the MMRM and presentation of the results will be similar to those described for the continuous ALT secondary endpoint in Section 7.5.2, except that baseline ALT is replaced by the relevant baseline value from the above list (which is also log transformed prior to fitting the model). The dependent variable will then be the difference in log-transformed values at each scheduled week and the log-transformed value at baseline.

[redacted] categories as defined in Section 5.3.

[redacted]

Proprietary confidential information © 2026 Boehringer Ingelheim International GmbH or one or more of its affiliated companies.

[redacted]

7.7 EXTENT OF EXPOSURE

Exposure to study drug will be presented by treatment group on the TS.

Proprietary confidential information © 2026 Boehringer Ingelheim International GmbH or one or more of its affiliated companies.

Descriptive summary statistics will be produced of the extent of exposure, as defined in Section 5.4.3.

A frequency table will also be presented of the extent of exposure by categories. The total patient-years exposure will also be summarised; the 28-day REP after the last dose of study treatment (see Section 6.1.3) will not be included for this purpose.

A frequency table will be produced of duration on study (regardless of whether the participant remained on-treatment) using the same categories as extent of exposure.

A descriptive summary and frequency table will be produced of the extent of exposure for participants who discontinued treatment early. This table will be produced in the following 4 ways:

- All participants who prematurely discontinued treatment
- Participants who prematurely discontinued treatment due to AE
- Participants who prematurely discontinued treatment not due to AE
- Participants who prematurely discontinued treatment due to AE and at least one AE with SOC 'Gastrointestinal disorders' leading to treatment discontinuation.

[redacted]

7.8 SAFETY ANALYSIS

All safety analyses will be performed on the TS, including only on-treatment data (unless otherwise specified).

7.8.1 Adverse Events

Unless otherwise specified, the analyses of AEs will be descriptive in nature. All analyses of AEs will be based on the number of participants with AEs and not on the number of AEs.

AEs will be coded using the most recent version of the Medical Dictionary for Regulatory Activities (MedDRA) coding dictionary. Definitions of Boehringer Ingelheim customized MedDRA Queries (BIcMQs) and Boehringer Ingelheim Tailored Search (BITS) for new

Proprietary confidential information © 2026 Boehringer Ingelheim International GmbH or one or more of its affiliated companies.

MedDRA versions are maintained by the Boehringer Ingelheim dictionary maintenance group.

7.8.1.1 Assignment of AEs to analysis phases

The analysis of adverse events will be based on the concept of treatment emergent AEs. All AEs occurring between first intake of study treatment and 28 days after last intake of study treatment will be considered "on-treatment" (see Section 6.1.3). All AEs occurring before the first intake of study treatment will be assigned to "screening" and all AEs occurring after the last intake of study treatment + 28 days will be assigned to "follow-up".

While tables will generally display results by treatment group, listings will also reflect the actual dose level at the onset of the respective AE.

Appendix 16.1.13.1 will include an analysis (overall summary table, frequency of AEs by system organ class (SOC) / preferred term (PT), frequency of serious adverse events (SAEs) by SOC/PT) where AEs are assigned to the following study periods: screening; treatment period (by treatment group); follow-up period (also by treatment group).

Restart of study treatment after a period of temporary interruptions is not planned in in this trial. Participants will be assumed to remain on study treatment from first dose to last dose of study treatment, even if small interruptions occur in practice. Therefore this topic will not be considered further in the AE analysis.

7.8.1.2 AE summaries

An overall summary of adverse events will be presented for all participants in the TS. This summary will show the number and percent of participants with any AE, any AE with CTCAE grade 3-5, any investigator defined drug-related AE, any AE leading to the discontinuation of study medication, any AESI (as determined by the investigator), any serious AE (including reason for serious (death, life-threatening, disability/permanent damage, required or prolonged hospitalisation, congenital anomaly/birth defect and other medically important events)), and any drug-related serious AE.

The frequency of participants with all AEs will be summarised by treatment group, primary system organ class (SOC) and preferred term (PT). Incidence rates as defined in Section 7.8.1.7 will generally be included (that is: overall AE summaries, tables displaying AEs by maximum CTCAE grade, and tables presenting information from special AE eCRF pages will not include incidence rates). A participant with multiple on-treatment occurrences of the same preferred term meeting the criteria for inclusion in the table will be counted only once in these tabulations. SOCs will be sorted by descending overall frequency; PTs will be sorted by descending overall frequency of survodutide (within SOC).

The AE summary by treatment group, primary SOC and PT will also be repeated for each of the following:

- Investigator-defined drug-related AEs
- AEs leading to treatment discontinuation
- Serious AEs

Proprietary confidential information © 2026 Boehringer Ingelheim International GmbH or one or more of its affiliated companies.

- Investigator-defined drug-related serious AEs
- Serious AEs leading to treatment discontinuation
- Any fatal AE
- AEs leading to treatment interruption
- AEs leading to dose reduction
- AEs requiring treatment
- Any AE reported by the investigator as AESI.

An additional table will present the frequency of participants with AEs by maximum CTCAE grade (1 to 5) and by treatment group, primary SOC and PT. When no events meet the criteria for inclusion, the table will be shown as indicating no events of that type have occurred.

[redacted]

For disclosure of AE data on ClinicalTrials.gov, the frequency of participants with non-serious AEs occurring with an incidence of greater than 5% (PT occurring in more than 5% of treated participants in one or more treatment groups) will be summarised by treatment group, primary SOC and PT. The frequency of participants with serious AEs will also be summarised. The frequency of participants with the reason death due to ‘all-causes’ will also be summarised.

Adverse event information for each treatment arm will be summarised for disclosure of AE data in the CTIS register. Frequency of participants with non-serious AEs with an incidence of greater than 5% (AEs occurring in more than 5% of treated participants in one or more treatment groups) and the frequency of participants with serious AEs will also be summarised, together with the occurrences and treatment-related occurrences for these serious AEs. The frequency of participants with the reason death due to “all-causes” will also be summarised. Furthermore, the total number of enrolled (screened) participants by country and by age group will be summarised. Moreover, for results disclosure in CTIS, for the set for which the baseline characteristics will be reported, ‘age by group’ will also be summarised.

7.8.1.3 Adverse events of special interest (AESIs)

The CTP pre-specifies the following AESIs:

Proprietary confidential information © 2026 Boehringer Ingelheim International GmbH or one or more of its affiliated companies.

- Potential severe Drug-Induced Liver Injury (DILI)
- Thyroid malignancies and C-cell hyperplasia
- Systemic and serious cutaneous hypersensitivity or anaphylactic reactions
- Acute gallbladder disease
- Severe hypoglycaemia requiring assistance (Level 3)

AESIs as recorded by the investigator on the eCRF AE page will already be summarised as part of the overall AE summary, and the AE summary by treatment group, primary SOC and PT, as described in Section 7.8.1.2.

A listing of all investigator reported AESIs will be prepared. Additionally, these events will be analysed using standardised or customized MedDRA queries as follows:

Liver-related adverse events

A table will be produced presenting the frequency of participants with liver-related adverse events as defined by the four MedDRA SMQs:

- Liver related investigations, signs and symptoms (narrow SMQ)
- Cholestasis and jaundice of hepatic origin (narrow SMQ)
- Hepatitis, non-infectious (narrow SMQ)
- Hepatic failure, fibrosis and cirrhosis and other liver damage-related conditions (narrow SMQ).

This table will be summarised by treatment group, MedDRA SMQ, and PT. Any PT which is classified under more than one SMQ will be included under each relevant SMQ.

Participants with liver-related adverse events will be listed.

Thyroid malignancies and C-cell hyperplasia

All associated adverse events reported by the investigator on the eCRF “AE: Thyroid Mass” page will be summarised by treatment group, primary SOC and PT.

Thyroid neoplasm adverse events defined by the narrow BIcMQ Thyroid neoplasms and the PT Thyroid C-cell hyperplasia will be summarised by treatment group, primary SOC and PT.

Participants with thyroid malignancies and C-cell hyperplasia will be listed. Details of all adverse events reported by the investigator on the eCRF “AE: Thyroid Mass” page will also be listed.

Proprietary confidential information © 2026 Boehringer Ingelheim International GmbH or one or more of its affiliated companies.

Systemic and serious cutaneous hypersensitivity or anaphylactic reactions

All associated adverse events reported by the investigator on the eCRF "AE: Hypersensitivity/Anaphylactic Reactions" page will be summarised by treatment group, primary SOC and PT.

In addition, hypersensitivity reaction adverse events defined by the SMQs
- SMQ (Narrow) Hypersensitivity
- SMQ (Narrow) Severe cutaneous adverse reactions
- SMQ (Narrow) Anaphylactic reaction
- SMQ (Narrow) Angioedema

will be summarised by treatment group, MedDRA SMQ and PT. Any PT which is classified under more than one SMQ will be included under each relevant SMQ.

Participants with hypersensitivity reactions will be listed. Details of all adverse events reported by the investigator on the eCRF "AE: Hypersensitivity/Anaphylactic Reactions" page will also be listed.

Acute gallbladder disease

All associated adverse events reported by the investigator on the eCRF "AE: Acute Gall Bladder Disease " page will be summarised by treatment group, primary SOC and PT.

In addition, acute gallbladder disease adverse events defined by the SMQs
- SMQ (Narrow) Biliary tract disorders
- SMQ (Narrow) Gallstone related disorders
- SMQ (Narrow) Infectious biliary disorders
- SMQ (Narrow) Gallbladder related disorders

will be summarised by treatment group, SMQ and PT. Any PT which is classified under more than one SMQ will be included under each relevant SMQ.

Participants with acute gallbladder disease will be listed. Details of all adverse events reported by the investigator on the eCRF "AE: Acute Gall Bladder Disease " page will be listed.

Severe hypoglycaemia requiring assistance

All associated adverse events reported by the investigator on the eCRF "Hypoglycaemia" page will be summarised by treatment group, primary SOC and PT.

In addition, all adverse events defined by SMQ (Narrow) Hypoglycaemia will be summarised by treatment group, primary SOC and PT.

Both of the above summaries will be presented overall and by presence of T2DM (yes/no).

Proprietary confidential information © 2026 Boehringer Ingelheim International GmbH or one or more of its affiliated companies.

Participants with hypoglycaemia adverse events will be listed. Details of all adverse events and events not qualified as adverse events reported by the investigator on the eCRF Hypoglycaemia page will also be listed.

7.8.1.4 Specific AEs

All events reported by the investigator as acute severe renal event, acute pancreatitis, persistent and unacceptable hyperglycaemia, hypotension/orthostatic hypotension/syncopal events, pancreatic cancer, arrhythmias and cardiac conduction disorders, local tolerability and injection site reactions and diabetic retinopathy or maculopathy will be summarised by treatment group, primary SOC and PT.

A listing will be prepared for each event type presenting all participants with the respective event.

Additionally, treatment emergent AEs defined by the following definitions will be summarised by treatment group, primary SOC (or SMQ as applicable) and PT:

- Acute renal event: SMQ (Narrow) Acute renal failure
- Acute pancreatitis: SMQ (Narrow) Acute pancreatitis
- Hyperglycaemia: SMQ (Narrow) Hyperglycaemia/new onset diabetes mellitus
- Hypotension:
  - Preferred terms: Hypotension, Hypotensive crisis, Orthostatic hypotension, Blood pressure decreased, Diastolic hypotension, Blood pressure diastolic decreased, Blood pressure systolic decreased, Blood pressure ambulatory decreased, Blood pressure orthostatic decreased, Mean arterial pressure decreased, Postprandial hypotension, Syncope, Presyncope
- Pancreatic cancer: BIcMQ (Narrow) Pancreatic neoplasm
- Arrhythmias and cardiac conduction disorders (by SMQ/PT):
  - SMQ (Narrow) Arrhythmia related investigations, signs and symptoms
  - SMQ (Broad) Cardiac arrhythmia terms (incl bradyarrhythmias and tachyarrhythmias)
  - SMQ (Broad) Torsade de pointes/QT prolongation
- Injection site reactions: HLT Injection site reactions
- Major depressive disorder: SMQ (Narrow) Depression (excl suicide and self injury)
- Suicidal ideation and behaviour: SMQ (Narrow) Suicide/self injury
- Diabetic retinopathy or maculopathy: For list of PTs see Appendix 10.3.

Proprietary confidential information © 2026 Boehringer Ingelheim International GmbH or one or more of its affiliated companies.

All of the specific AE summaries above will be presented overall, except for the items relating to diabetic retinopathy or maculopathy, which will also be presented by presence of T2DM (yes/no).

For derivations based on HLT, PTs assigned to the HLT via primary and secondary path are to be considered.

For each topic separately, participants with the respective event will be listed.

A frequency table will be produced for participants with injection site reaction overall and presence of symptoms of injection site reaction (swelling, induration, heat, redness, pain, other) for the subset of participants with the event.

Changes in eye examination for diabetic retinopathy/maculopathy from baseline will be listed.

7.8.1.5 Adjudicated events

Frequency tables summarising the adjudication results for each of the following events (as defined in the CTP) with onset on-treatment will be presented:

- Major adverse CV events (5P-MACE) including
  - Non-fatal MI
  - Non-fatal stroke
  - Ischaemia related coronary revascularisation
  - Heart failure event (HFE) (including hospitalisation for heart failure (HHF), emergency room visit, urgent care visit, or urgent outpatient heart failure (HF) visit)
  - CV death
- Non-CV death
- Acute pancreatitis
- Thyroid malignancies and C-cell hyperplasia
- Pancreatic cancer
- DILI

Thyroid malignancies and C-cell hyperplasia and pancreatic cancer will additionally be presented for the whole on-trial period.

Listings will be provided, that show the trigger events and result of adjudication for each event type.

Proprietary confidential information © 2026 Boehringer Ingelheim International GmbH or one or more of its affiliated companies.

7.8.1.6 Possible cases of IMP abuse or overuse

The frequency of trial participants with adverse events "drug abuse" or "drug overdose" and frequency of trial participants who answered the questions about drug accountability and discrepancies with 'yes' will be tabulated. For AE "drug abuse", a table will be generated summarising the frequency of trial participants with associated AE by primary SOC and PT, starting with frequency of trial participants with "Any AE of drug abuse". For AE "drug overuse", a table will be generated summarising the frequency of trial participants with associated AE by primary SOC and PT, starting with frequency of trial participants with "Any AE of drug overuse".

A listing for "drug abuse" which presents all associated AEs together (with all information from the eCRF AE page) with 'additional information', a listing for "drug overuse" which lists all associated AEs (with all information from the eCRF AE page), and a listing for the two questions on 'drug accountability discrepancies' and other discrepancies' will be produced. The listings will appear under the same section as the specified tables.

Adverse events possible related to drug abuse will be summarized by treatment group, this will be defined by CNS depressant effects (BITS), Dissociation (BITS), Drug abuse, dependence, withdrawal and substance-related disorders, incl. diversion (BITS), Euphoria-related adverse events (BITS), Mood disorders and disturbances (BITS), Perceptual disturbances/ psychotomimetic effects (BITS) and Drug abuse, dependence and withdrawal (SMQ-narrow).

7.8.1.7 Adverse event incidence rates

Generally, exposure-adjusted adverse event analyses will be performed for all AEs, investigator defined drug-related AEs, AEs leading to discontinuation, serious AEs, adverse events of special interest and specific adverse events by primary SOC and PT.

The time at risk in patient years for the on-treatment period is derived as follows:

- Participants with AE:

time at risk (AE) in days = date of start of AE of interest – IMP start date + 1

- Participants without AE:

time at risk (AE) in days = end date of time at risk – IMP start date + 1, where end date of time at risk is the minimum of date of last IMP intake + x days and date of death, if applicable.

The standard approach will be x=28 days, but for selected events the time until trial end will also be considered.

Total AE-specific time at risk per treatment group is then derived as:

Time at risk (AE) [years] = Sum of time at risk [days] over all participants in a treatment group/365.25

For each row of a table (e.g. displaying a SOC), time at risk is calculated using start of first AE summarised in this row, e.g. for participant with AE in a specified SOC,

time at risk = date of start of AE with specified PT in this SOC – IMP start date + 1.

Proprietary confidential information © 2026 Boehringer Ingelheim International GmbH or one or more of its affiliated companies.

The AE incidence rate per 100 patient-years will then be calculated as follows:

Incidence rate per 100 patient-years = 100 x number of participants with AE / time at risk (AE) [years].

7.8.2 Laboratory data

The analyses of laboratory data will be descriptive in nature, considering laboratory measurements which are on-treatment only.

Only laboratory parameters routinely collected at the clinic visits as listed in the CTP flow chart will be included in the descriptive statistics. Laboratory values only assessed at screening or as a result of a reflex test will not be displayed in the descriptive statistics table.

Derivation of Lower Limit of Quantification (LLQ) and Upper Limit of Quantification (ULQ) into numeric values for analysis is as follows:

If the original laboratory value is reported as a character string ">= ULQ" or "> ULQ", the character part will be dropped, “ULQ” converted to a numeric value which will be kept as the corresponding derived numeric laboratory value. E.g., “>= 60” or “> 60” will be translated to 60 (numeric value).

If the original laboratory value is reported as a character string "< LLQ" or "<= LLQ", the character part will be dropped and (0.5 x numeric value of LLQ) will be kept as the corresponding derived numeric laboratory value. E.g., “<= 3.0” or “< 3.0” will be translated to 1.5 (numeric value).

7.8.2.1 General analysis

All of the following displays will be produced by treatment group.

<u>Safety laboratory data specified in CTP Table 10</u>

For continuous safety laboratory variables where reference ranges exist, normalised values will be derived (i.e. transformation to both a standard unit and to a standard reference range). Descriptive statistics will be presented over time for absolute values and changes from baseline in each of the laboratory variables where normalised values are possible. All scheduled in-clinic weeks will be presented as well as last value, minimum value and maximum value on-treatment.

Laboratory parameters will be shown in SI units.

For continuous safety laboratory variables where reference ranges exist, a shift table will be produced summarising the shifts from baseline to the last value on treatment, and displaying categories “Low”, “Normal” and “High” with respect to the relevant reference range.

Proprietary confidential information © 2026 Boehringer Ingelheim International GmbH or one or more of its affiliated companies.

For categorical safety laboratory variables, a shift table will be produced summarising the shifts from baseline to the last value on treatment, and displaying each relevant category. For any semi-quantitative variables (i.e. those recorded as either "Normal" or with a continuous numerical value otherwise), the shift table will display categories "Normal" and "Abnormal" only, without further categorisation of the continuous values into different degrees of abnormality.

Safety laboratory data specified in CTP Table 10 and additional laboratory data assessed as e.g. reflex-tests

Potentially clinically significant abnormalities will be identified using BI standard rules. A listing containing these rules (including for each variable whether a low range applies, or a high range applies, or both) will be included in the CTR. A frequency table will be used to summarise the number and percentage of participants with potentially clinically significant abnormalities, for each variable where a BI standard rule exists. Participants with an abnormal value at baseline will be presented separately on this table.

A separate listing will present potentially clinically significant abnormal laboratory values; for each functional laboratory group all laboratory values of the participant will be listed, if there exists at least one value with clinically significant abnormality within the group. Additionally, a listing with pregnancy test results will be produced.

[redacted]

Proprietary confidential information © 2026 Boehringer Ingelheim International GmbH or one or more of its affiliated companies.

[redacted]

Proprietary confidential information © 2026 Boehringer Ingelheim International GmbH or one or more of its affiliated companies.

A shift table of calcitonin will be produced by treatment group, summarising the shifts from [redacted]

7.8.3 Vital signs

Descriptive statistics for body weight, waist circumference, systolic BP, diastolic BP and BMI will be displayed in the respective section for efficacy analyses.

Descriptive statistics for pulse rate and hip circumference will be presented in this section.

7.8.4 ECG

All derivations relating to 12-lead ECG variables are given in Section 5.4.1.

For RR interval, only listings will be provided.

Abnormal findings will be reported as baseline conditions (at screening) or as AEs (during the trial) if judged clinically relevant by the investigator.

The ECG recordings will be centrally evaluated and interpretated as normal, abnormal, or not evaluable.

7.8.4.1 Quantitative variables

Descriptive statistics will be presented over time for absolute values and absolute changes from baseline for PR, QRS, uncorrected QT, HR, and QTcF. Figures including mean and standard deviation over time by treatment group will be provided. Only scheduled weeks will be included.

For HR a restricted maximum likelihood (REML) based approach using a MMRM comparing the change from baseline over time of treatment will be performed. The model will include fixed, categorical effects of treatment at each visit and presence of T2DM (yes/no), and the fixed continuous effects of baseline at each visit, using visit as repeated measures, and unstructured covariance matrix to model the relationship between pairs of measurements

Proprietary confidential information © 2026 Boehringer Ingelheim International GmbH or one or more of its affiliated companies.

taken at different visits on the same participant. The Kenward-Roger approximation will be used to estimate denominator degrees of freedom and adjust standard errors. Significance tests will be based on least-squares means using a two-sided α = 0.10 (two-sided 90% confidence intervals).

7.8.4.2 Categorical variables

Frequency tables will be provided for the categorical variables described below, which are determined from the specified quantitative ECG variables:

- New onset (meaning that this or a higher category was not present any time at baseline) of maximum QTcF interval > 450 to 480 msec, > 480 to 500 msec, or > 500 msec on treatment
- New onset (not present any time at baseline) of uncorrected QT interval > 500 msec at any time on treatment
- Maximum change from baseline of QTcF ≤ 30 msec, > 30 to ≤ 60 msec, or > 60 msec on treatment
- Maximum change from baseline of QT ≤ 60 msec, or > 60 msec on treatment
- Increase in heart rate (> 5 bpm, > 10 bpm, > 15 bpm, and > 20 bpm) at any time on treatment

For assignment of a participant to one of the above categories, all available time points on-treatment will be considered.

For QTcF, a shift table will summarize shift from baseline to maximum value on treatment by the categories "≤ 450 msec", "> 450 to 480 msec","> 480 to 500 msec and "> 500 msec".

A scatter plot of QTcF at baseline versus the maximum change from baseline will be produced, including diagonal reference lines for absolute QTcF equal to 450 msec, 480 msec and 500 msec, as well as horizontal reference lines for QTcF maximum changes from baseline equal to 30 msec and 60 msec.

7.8.4.3 Binary variables

The occurrence of any of the following will be considered as "notable findings" and summarised using frequency tables:

- New onset (not present any time at baseline) of uncorrected QT interval > 500 msec at any time on treatment
- New onset of QTcF interval > 500 msec at any time on treatment
- Change from baseline of QTcF > 60 msec at any time on treatment
- An increase in HR ≥ 25% from baseline, when corresponding on-treatment value of HR is > 100 beats/min, or decrease in HR ≥ 25% from baseline, when corresponding on-treatment value of HR is < 50 beats/min, at any time on-treatment
- An increase in PR ≥ 25% from baseline , when corresponding on-treatment value of PR interval is > 200 msec, at any time on treatment
- An increase in QRS ≥ 10% from baseline, when corresponding on-treatment value of QRS duration is > 110 msec, at any time on treatment.

Proprietary confidential information © 2026 Boehringer Ingelheim International GmbH or one or more of its affiliated companies.

Frequency tables will also include ECG morphological findings (determined and categorized based on SDTM terminology) that might be attributable to treatment:

- ECG findings pre-treatment
- ECG findings on treatment
- New onset of ECG findings. New onsets of findings not present at baseline will be explored. A morphological finding observed on treatment that was not reported at baseline will be categorized as a 'new onset' of this finding.

Tables will be provided showing the proportion of participants in the following categories:

- with an increase in heart rate (separately for > 5 bpm, > 10 bpm, > 15 bpm, and > 20 bpm) from the baseline at each scheduled week
- With a heart rate >100 bpm recorded at 2 consecutive visits, interpreted as two measurements, taken at least one day apart, at any time on treatment

7.8.5 Mental health questionnaires

Frequency tables will be produced for C-SSRS summarising the information collected at screening, and the worst outcome on treatment. A shift table will present the change from baseline to the worst suicidal ideation observed on-treatment.

For the PHQ-9 total score (units on a scale), a shift table will be produced summarising the shifts from baseline to each analysis visit and additionally the maximum value on-treatment, displaying the PHQ-9 total score categories <15 and >= 15.

[redacted]

Proprietary confidential information © 2026 Boehringer Ingelheim International GmbH or one or more of its affiliated companies.

[redacted]

Proprietary confidential information © 2026 Boehringer Ingelheim International GmbH or one or more of its affiliated companies.

[redacted]

Proprietary confidential information © 2026 Boehringer Ingelheim International GmbH or one or more of its affiliated companies.

[redacted]

Proprietary confidential information © 2026 Boehringer Ingelheim International GmbH or one or more of its affiliated companies.

[redacted]

8. TIMEPOINT OF RELEASE OF TREATMENT INFORMATION

The treatment information will be released to unblind the trial database after the last participant has completed their End-of-Study/Follow-up visit and all data has been entered and cleaned as defined in the "Data Ready to be Unblinded and / or Final Trial Closure Notification" (RUN) form.

Proprietary confidential information © 2026 Boehringer Ingelheim International GmbH or one or more of its affiliated companies.

9. REFERENCES

9.1	*CPMP/ICH/363/96:* "Statistical Principles for Clinical Trials", ICH Guideline Topic E9, Note For Guidance on Statistical Principles for Clinical Trials, current version.
9.2	*EMA/CHMP/ICH/43622/2017:* "Addendum on Estimands and Sensitivity Analysis in Clinical Trials" to the Guideline on Statistical Principles for Clinical Trials, ICH E9 (R1), current version.
9.3	*CPMP/ICH/137/95*: "Structure and Content of Clinical Study Reports", ICH Guideline Topic E3; Note For Guidance on Structure and Content of Clinical Study Reports, current version.
9.4	Bell J, Drury T, Muetze T, Pipper CB, Guizzaro L, Mitroiu M, et al. Estimation methods for estimands using the treatment policy strategy; a simulation study based on the PIONEER 1 trial. Pharm Stat 2025, 24: 1539-1604 [R24-0906]
9.5	Firth D. Bias reduction of maximum likelihood estimates. Biometrika 1993, 80: 27–38 [R20-2291]
9.6	Ge M, Durham LK, Meyer RD, et al. Covariate-adjusted difference in proportions from clinical trials using logistic regression and weighted risk differences. Ther Innov Regul Sci 2011, 45: 481-493 [R16-5360]
9.7	Steingrimsson JA, Hanley DF, Rosenblum M. Improving precision by adjusting for prognostic baseline variables in randomized trials with binary outcomes, without regression model assumptions. Contemp Clin Trials 2017, 54: 18-24 [P17-13765]
9.8	Maruish, ME. User's manual for the SF-36v2 Health Survey (3rd ed. 2011). Lincoln, RI: QualityMetric Incorporated [R23-1385]
9.9	Kolotkin RL, Williams VSL, Ervin CM, et al. Validation of a new measure of quality of life in obesity trials: Impact of Weight on Quality of Life-Lite Clinical Trials Version. Clin. Obes. 2019 Jun, 9(3): e12310 [R19-0163]
9.10	Younossi ZM, Guyatt G, Kiwi M, et al. Development of a disease specific questionnaire to measure health related quality of life in patients with chronic liver disease. Gut. 1999 Aug, 45(2): 295-300 [R17-0283]
9.11	Buysse DJ, Reynolds CF, Monk TH, et al. The Pittsburgh Sleep Quality Index: a new instrument for psychiatric practice and research. Psychiatry Res. 1989 May, 28(2): 193-213 [R00-0292]

Proprietary confidential information © 2026 Boehringer Ingelheim International GmbH or one or more of its affiliated companies.

9.12	Wang Y, Tu W, Kim Y, et al. Statistical methods for handling missing data to align with treatment policy strategy. Pharm Stat 2023, 22: 650-670 [R25-3827]
9.13	[redacted]

Proprietary confidential information © 2026 Boehringer Ingelheim International GmbH or one or more of its affiliated companies.

10. ADDITIONAL SECTIONS

10.1 PATTERN MERGING ALGORITHM FOR RETRIEVED DROPOUT MULTIPLE IMPUTATION ANALYSIS OF BODY WEIGHT

For each analysis week, it will be checked whether a pattern category used in the retrieved dropout imputation model for that week has at least 5 participants in each treatment group with an observed value at that week. This condition will be called the “observation criterion”.

The notation P_on_treatment below represents those participants who were on-treatment at the analysis week described. Unlike the starting pattern categories described in Section 7.4.2.1 (which describe mutually exclusive groups of participants according to the first off-treatment analysis week), P_on_treatment varies according to the analysis week being described (because the number of participants is expected to decrease with increasing week).

The rules for merging pattern categories are described below. It should be understood that if the observation criterion is not met in one treatment group, then the stated merging rule will be applied in both treatment groups.

- **For analysis visits 1, 2 and 3 (weeks 4, 8 and 12)**:
  1. If P1_3 does not fulfil the observation criterion
     then merge with P_on_treatment
     Resulting pattern: P_{1_3 and on_treatment}

- **For analysis visits 4, 5 and 6 (weeks 16, 20 and 24):**
  1. If P1_3 or P4_6 do not fulfil the observation criterion
     then merge P1_3 and P4_6
     Resulting pattern:P1_6
  2. If P1_6 does not fulfil the observation criterion
     then merge with P_on_treatment
     Resulting pattern: P_{1_6 and on_treatment}

- **For analysis visits 7 and 8 (weeks 28 and 36)**:
  1. If P1_3 or P4_6 do not fulfil the observation criterion
     then merge P1_3 and P4_6
     Resulting pattern:P1_6
  2. If P1_6 does not fulfil the observation criterion
     then merge with P7_8
     Resulting pattern: P1_8
  3. If P7_8 does not fulfil the observation criterion
     then merge with P4_6 or P1_6
     Resulting pattern: P4_8 or P1_8
  4. If P1_8 does not fulfil the observation criterion
     then merge with P_on_treatment
     Resulting pattern: P_{1_8 and on_treatment}

Proprietary confidential information © 2026 Boehringer Ingelheim International GmbH or one or more of its affiliated companies.

Note: P4_8 must fulfil the observation criterion if P4_6 does.

- **For analysis visits 9 and 10 (weeks 42 and 48):**
  1. If P1_3 or P4_6 do not fulfil the observation criterion then merge P1_3 and P4_6
     Resulting pattern:P1_6
  2. If P7_8 or P9_10 do not fulfil the observation criterion then merge P7_8 and P9_10
     Resulting pattern:P7_10
  3. If P1_6 does not fulfil the observation criterion then merge with P7_8 or P7_10
     Resulting pattern:P1_8 or P1_10
  4. If P7_10 does not fulfil the observation criterion then merge with either P4_6 or P1_6
     Resulting pattern:P4_10 or P1_10
  5. If P1_10 does not fulfil the observation criterion then merge with P_on_treatment
     Resulting pattern: P_{1_10 and on_treatment}

Note: P4_10 must fulfil the observation criterion if P4_6 does.

10.2 VARIABLES TO BE ANALYSED AFTER LOGARITHMIC TRANSFORMATION

Variable	Type of endpoint	Endpoint
ALT	Secondary	Relative CfB to Week 48
HOMA-IR	Secondary	Relative CfB to Week 48
Total cholesterol	Further	Relative CfB to Week 48
HDL cholesterol	Further	Relative CfB to Week 48
LDL cholesterol	Further	Relative CfB to Week 48
Triglycerides	Further	Relative CfB to Week 48
VLDL cholesterol	Further	Relative CfB to Week 48
Free fatty acids	Further	Relative CfB to Week 48
ApolipoproteB (ApoB)	Further	Relative CfB to Week 48
Fasting C-peptide	Further	Relative CfB to Week 48
Fasting plasma insulin (FPI)	Further	Relative CfB to Week 48
HOMA-β	Further	Relative CfB to Week 48
Glucagon	Further	Relative CfB to Week 48
GLP-1 total	Further	Relative CfB to Week 48
Alanine	Further	Relative CfB to Week 48
Glycine	Further	Relative CfB to Week 48
Threonine	Further	Relative CfB to Week 48

Proprietary confidential information © 2026 Boehringer Ingelheim International GmbH or one or more of its affiliated companies.

[redacted]

Proprietary confidential information © 2026 Boehringer Ingelheim International GmbH or one or more of its affiliated companies.

Topic	Preferred Terms
	Retinal aneurysm<br>Retinal artery occlusion<br>Retinal artery stenosis<br>Retinal collateral vessels<br>Retinal cryoablation<br>Retinal detachment<br>Retinal exudates<br>Retinal fibrosis<br>Retinal haemorrhage<br>Retinal laser coagulation<br>Retinal microangiopathy<br>Retinal neovascularization<br>Retinal oedema<br>Retinal thickening<br>Retinal vascular disorder<br>Retinal vascular occlusion<br>Retinal vein occlusion<br>Retinopathy<br>Retinopathy haemorrhagic<br>Retinopathy proliferative<br>Sudden visual loss<br>Tractional retinal detachment<br>Vision acuity reduced<br>Vision acuity reduced transiently<br>Vision blurred<br>Vision impairment<br>Vitrectomy<br>Vitreous floaters<br>Vitreous haemorrhage

Proprietary confidential information © 2026 Boehringer Ingelheim International GmbH or one or more of its affiliated companies.

11. HISTORY TABLE

Table 12 History table

**Version**	**Date (DD-MMM-YY)**	**Author**	**Sections changed**	**Brief description of change**
1.0	**23-JAN-2026**	[redacted]	None	This is the final TSAP.
