# Supplementary material for: Survodutide in adults with obesity and metabolic dysfunction-associated steatotic liver disease: SYNCHRONIZE-MASLD, a randomized, double-blind, placebo-controlled phase 3 trial
Source: Nat Med. 2026 Jun 7;32(8):2948–58. doi: 10.1038/s41591-026-04479-3 (PMC13472939; doi:10.1038/s41591-026-04479-3)
Supplement: Supplementary file 2 [file 41591_2026_4479_MOESM2_ESM.pdf]

nature portfolio

Corresponding author(s): Lee Kaplan

Last updated by author(s): May 21, 2026

Reporting Summary

Nature Portfolio wishes to improve the reproducibility of the work that we publish. This form provides structure for consistency and transparency in reporting. For further information on Nature Portfolio policies, see our Editorial Policies and the Editorial Policy Checklist.

Statistics

For all statistical analyses, confirm that the following items are present in the figure legend, table legend, main text, or Methods section.

n/a	Confirmed	
☐	☒	The exact sample size (*n*) for each experimental group/condition, given as a discrete number and unit of measurement
☒	☐	A statement on whether measurements were taken from distinct samples or whether the same sample was measured repeatedly
☐	☒	The statistical test(s) used AND whether they are one- or two-sided<br>*Only common tests should be described solely by name; describe more complex techniques in the Methods section.*
☐	☒	A description of all covariates tested
☐	☒	A description of any assumptions or corrections, such as tests of normality and adjustment for multiple comparisons
☐	☒	A full description of the statistical parameters including central tendency (e.g. means) or other basic estimates (e.g. regression coefficient) AND variation (e.g. standard deviation) or associated estimates of uncertainty (e.g. confidence intervals)
☐	☒	For null hypothesis testing, the test statistic (e.g. *F*, *t*, *r*) with confidence intervals, effect sizes, degrees of freedom and *P* value noted<br>*Give P values as exact values whenever suitable.*
☒	☐	For Bayesian analysis, information on the choice of priors and Markov chain Monte Carlo settings
☒	☐	For hierarchical and complex designs, identification of the appropriate level for tests and full reporting of outcomes
☒	☐	Estimates of effect sizes (e.g. Cohen's *d*, Pearson's *r*), indicating how they were calculated

Our web collection on statistics for biologists contains articles on many of the points above.

Software and code

Policy information about availability of computer code

Data collection	This study did not utilize custom algorithms or bespoke software; code deposition is not applicable.
Data analysis	SAS® version 9.4 or later was used for all analysis. Where statistical analyses require the use of R instead of SAS®, R version 4.2.2 or later was used.

For manuscripts utilizing custom algorithms or software that are central to the research but not yet described in published literature, software must be made available to editors and reviewers. We strongly encourage code deposition in a community repository (e.g. GitHub). See the Nature Portfolio guidelines for submitting code & software for further information.

Data

Policy information about availability of data

All manuscripts must include a data availability statement. This statement should provide the following information, where applicable:

- Accession codes, unique identifiers, or web links for publicly available datasets
- A description of any restrictions on data availability
- For clinical datasets or third party data, please ensure that the statement adheres to our policy

To ensure independent interpretation of clinical study results and enable authors to fulfill their role and obligations under the International Committee of Medical Journal Editors criteria, Boehringer Ingelheim grants all external authors access to relevant clinical study data. In adherence with the Boehringer Ingelheim policy on transparency and publication of clinical study data, scientific and medical researchers can request access to clinical study data, typically 1 year after the approval has

been granted by major regulatory authorities or after termination of the development program. Initial feedback is provided within 21 days of receipt of the data sharing request. Upon approval, and governed by a Legal Agreement, data are shared in a secured data-access system for a limited period of 1 year, which may be extended upon request. Prior to providing access, clinical study documents and data will be examined, and, if necessary, redacted and de-identified, to protect the personal data of study participants and personnel, and to respect the boundaries of the informed consent of the study participants. Researchers should use the https://vivli.org/ link to request access to study data and visit www.mystudywindow.com/msw/datasharing for further information.

Research involving human participants, their data, or biological material

Policy information about studies with human participants or human data. See also policy information about sex, gender (identity/presentation), and sexual orientation and race, ethnicity and racism.

Reporting on sex and gender	Sex was recorded and reported in the baseline characteristics. Gender identity was recorded but not reported in the manuscript. Subgroup analysis for the co-primary endpoints included sex but is not reported in the manuscript. To ensure that enough male and female trial participants were enrolled and randomized, a cap of approximately 70% was set for the number of female trial participants randomized.
Reporting on race, ethnicity, or other socially relevant groupings	Race and ethnicity were recorded according to local regulations. Subgroup analysis for the co-primary endpoints included race and ethnicity but is not reported in the manuscript.
Population characteristics	The study population comprised adult participants with at risk MASLD and obesity. Characteristics are reported in Table 1.
Recruitment	Recruitment was conducted at multiple international clinical sites in accordance with the study protocol. Enrolment was predefined on inclusion and exclusion criteria. Recruitment timelines and enrolment numbers are reported in the manuscript
Ethics oversight	The protocol was approved by the Advarra central institutional review board and the European Medicines Agency, and the trial was conducted in accordance with the principles of the Declaration of Helsinki and Good Clinical Practice guidelines of the International Council for Harmonisation. All participants provided written informed consent before enrollment.

Note that full information on the approval of the study protocol must also be provided in the manuscript.

Field-specific reporting

Please select the one below that is the best fit for your research. If you are not sure, read the appropriate sections before making your selection.

☒ Life sciences ☐ Behavioural & social sciences ☐ Ecological, evolutionary & environmental sciences

For a reference copy of the document with all sections, see nature.com/documents/nr-reporting-summary-flat.pdf

Life sciences study design

All studies must disclose on these points even when the disclosure is negative.

Sample size	A sample size of 156 participants (104 randomized to survodutide, 52 to placebo) was calculated to provide 90% overall power to demonstrate superiority of survodutide 6.0 mg over placebo for both co-primary endpoints, each at a two-sided significance level of 0.05, assuming an early treatment discontinuation rate of 15%. During the course of the trial, the number of participants increased to approximately 220 owing to an unexpected sharp drop in the screening failure percentage following recruitment closure.
Data exclusions	No participants were excluded following treatment initiation. Procedures for handling missing data are reported in the manuscript, with further details in the protocol (provided as Supporting Information). Analyses were based on available samples and evaluable measurements as reported in the manuscript.
Replication	Clinical and laboratory assessments were performed according to the protocol and prespecified assay procedures. This is a single study.
Randomization	Participants were randomly assigned in a 2:1 ratio to receive once-weekly subcutaneous injections of survodutide 6.0 mg (maintenance dose) or placebo. Details on randomization procedures are reported in the protocol (provided as Supporting Information).
Blinding	Trial participants, investigators, central reviewers, and everyone involved in the trial conduct were blinded until after database lock.

Reporting for specific materials, systems and methods

We require information from authors about some types of materials, experimental systems and methods used in many studies. Here, indicate whether each material, system or method listed is relevant to your study. If you are not sure if a list item applies to your research, read the appropriate section before selecting a response.

Materials & experimental systems

n/a	Involved in the study
☒	☐ Antibodies
☒	☐ Eukaryotic cell lines
☒	☐ Palaeontology and archaeology
☒	☐ Animals and other organisms
☐	☒ Clinical data
☒	☐ Dual use research of concern
☒	☐ Plants

Methods

n/a	Involved in the study
☒	☐ ChIP-seq
☒	☐ Flow cytometry
☒	☐ MRI-based neuroimaging

Clinical data

Policy information about clinical studies

All manuscripts should comply with the ICMJE guidelines for publication of clinical research and a completed CONSORT checklist must be included with all submissions.

Clinical trial registration	ClinicalTrials.gov registration: NCT06309992.
Study protocol	The study protocol has been provided as Supplementary Information
Data collection	Trial-site investigators were responsible for data collection.
Outcomes	The trial aimed to provide evidence of efficacy, safety, and tolerability of survodutide. Primary and secondary endpoints are described in the manuscript.

Plants

Seed stocks	*Report on the source of all seed stocks or other plant material used. If applicable, state the seed stock centre and catalogue number. If plant specimens were collected from the field, describe the collection location, date and sampling procedures.*
Novel plant genotypes	*Describe the methods by which all novel plant genotypes were produced. This includes those generated by transgenic approaches, gene editing, chemical/radiation-based mutagenesis and hybridization. For transgenic lines, describe the transformation method, the number of independent lines analyzed and the generation upon which experiments were performed. For gene-edited lines, describe the editor used, the endogenous sequence targeted for editing, the targeting guide RNA sequence (if applicable) and how the editor was applied.*
Authentication	*Describe any authentication procedures for each seed stock used or novel genotype generated. Describe any experiments used to assess the effect of a mutation and, where applicable, how potential secondary effects (e.g. second site T-DNA insertions, mosiacism, off-target gene editing) were examined.*
